# Enantioselective Synthesis of α‑Oxygenated Ketones via Organocatalytic Formal O–H Bond Insertion of Sulfonium Ylides

**DOI:** 10.1021/jacs.5c10842

**Published:** 2025-08-20

**Authors:** Chenxiao Qian, Ziwei Zhong, Qingzheng Xu, Pengfei Li, Chaoshen Zhang, Jianwei Sun

**Affiliations:** † Department of Chemistry and the Hong Kong Branch of Chinese National Engineering Research Centre for Tissue Restoration & Reconstruction, 58207 The Hong Kong University of Science and Technology, Clear Water Bay, Kowloon, Hong Kong SAR 999077, China; ‡ Department of Chemistry, Guangdong Provincial Key Laboratory of Catalysis, College of Science, 255310 Southern University of Science and Technology, Shenzhen, Guangdong 518055, China

## Abstract

Described here is an example of organocatalytic enantioselective formal O–H bond insertion of α-carbonyl sulfur ylides, providing efficient access to highly enantioenriched ketones bearing a tertiary α-oxygenated stereocenter. This metal-free approach is complementary to the well-established metal-based diazo carbonyl chemistry, which has gained broad success for esters but not for ketones. The proper choice of a chiral thiourea catalyst in combination with *N*-hydroxyl phthalimides proved to be crucial to success. Mechanistic studies indicate that the reaction is likely initiated by activation of sulfonium ylides by hydrogen bonding, thus distinct from previous related cases using chiral phosphoric acid catalysis that involves initial protonation of the basic sulfonium ylide functionality. DFT studies provided important insights into the key C–O bond-forming intermediates and transition states.

## Graphical abstract

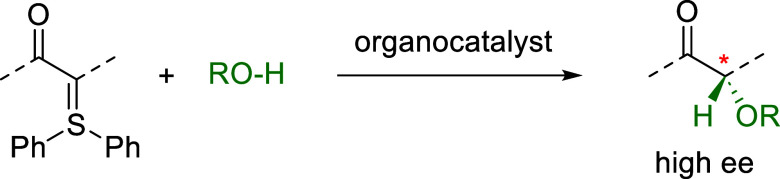

## Introduction

The chiral α-oxygenated carbonyl functionality is widely present in biologically important molecules and natural products (Scheme 1A).^1^ Specifically, enantioenriched chiral ketones bearing an α-oxygenated tertiary carbon stereogenic center are particularly useful, as they are also valuable precursors to other important chiral building blocks, such as diols and amino alcohols bearing vicinal stereocenters.^2–4^ Among the various methods to introduce a C–O bond at the carbonyl α position with concomitant enantiocontrol, catalytic enantioselective O–H bond insertion to metal carbenes generated *in situ* from the corresponding α-diazo carbonyl compounds represents one of the most established approaches (Scheme 1B).^5–9^ However, although this type of process has been intensively studied over the past two decades, a literature survey indicated a stunning fact—almost all the cases so far have dealt with α-diazo esters but rarely with other types of carbonyl compounds.^5,6^ Specifically, catalytic enantioselective insertion of an α-diazo ketone from α-diazo ketones remained scarce. Liu and co-workers pioneered the first example using carboxylic acids as the reaction partner, with suboptimal enantioselectivity.^7^ However, for the reaction with alcohols, a mechanistically distinct strategy requiring metal and chiral phosphoric acid cocatalysis had to be employed, as pioneered by Zhou and co-workers.^8^ Both of the leading examples employed a special type of substrate, α-aryl-α-diazo ketones. The challenges of such reactions from α-diazo ketones are associated with the limited stability of these substrates and the diverse possibilities of side reactions. Moreover, the enantiodetermining step—protonation of the ketone enolates bound to the metal center bearing a chiral ligand—is often complicated by uncontrolled protonation of the free enolate due to its labile coordination with the metal center, thus causing loss or compromise of enantiocontrol.^8^ In this context, the development of alternative strategies would be desirable and expected to provide complementary solutions to the above challenges.

**Scheme 1:**
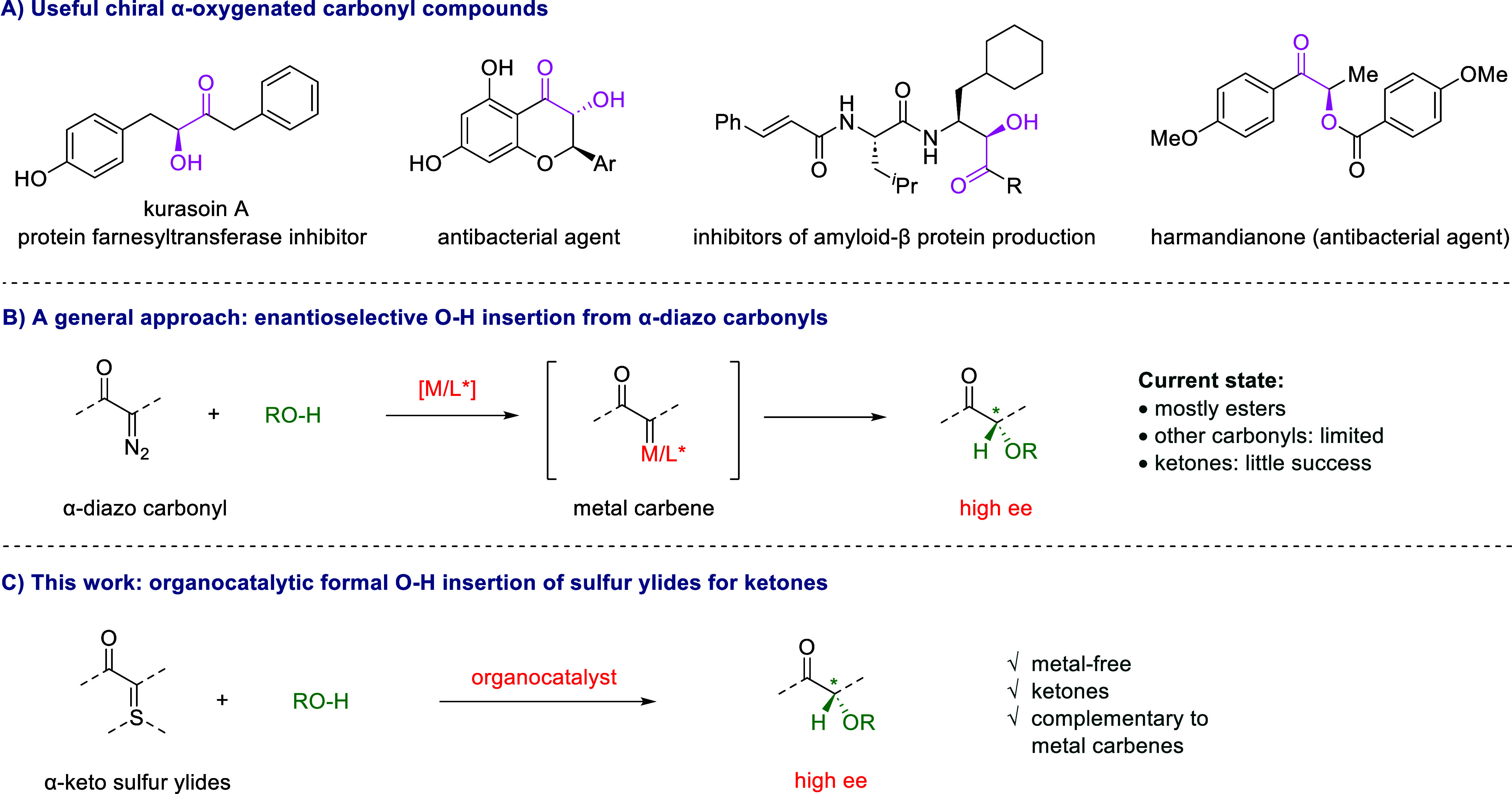
Introduction to α-Oxygenated Carbonyl Compounds and Reaction Design

α-Carbonyl sulfur ylides represent an important family of safe surrogates of α-diazo carbonyl compounds.^10–13^ Importantly, they have been demonstrated to exhibit complementary utility, particularly in view of their superior performance in generating chiral α-functionalized ketones, as well as their extraordinary compatibility with organocatalysis. However, currently the asymmetric formal H–X (X = heteroatom) bond insertion reactions have only been limited to certain strong nucleophiles, such as amines and thiols, as pioneered by the Burtoloso and our laboratories.^11–13^ Notably, weak oxygen nucleophiles have remained unsuccessful so far. Here we report our progress in addressing this unsolved challenge by developing a metal-free protocol to access chiral α-oxygenated ketones with high enantioselectivity, thus complementary to metal-catalyzed diazo chemistry (Scheme 1C).

## Results and Discussion

We began our study with α-keto sulfonium ylide **1a** as the model substrate. Common oxygen nucleophiles, phenol and benzyl alcohol, were employed as the reaction partners (Scheme 2). Chiral phosphoric acid **A**, the catalyst of choice previously identified for the formal N–H insertion,^12^ was first examined for this reaction. Both PhOH and BnOH showed the desired reactivity, leading to the corresponding α-oxygenated ketones in 89% and 17% yields, respectively. However, no enantioselectivity was observed in either case (0% ee). These preliminary results suggested that stereocontrol in the formal insertion of sulfonium ylides might be a completely different scenario from previously established insertions.

**Scheme 2:**
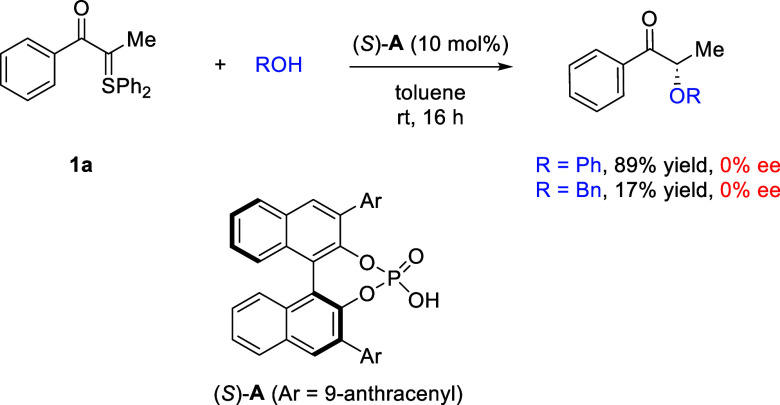
Preliminary Results

We reasoned that a different mechanistic approach might be needed to allow for successful enantiomeric induction. The previous approach with a chiral phosphoric acid catalyst involves initial rapid protonation of the sulfonium ylide substrate. However, with relatively weak oxygen-based nucleophiles, a less acidic catalyst might be suitable. Thus, considerable efforts were devoted to the search for a good balance between nucleophiles and catalysts. Gratifyingly, *N*-hydroxyphthalimide (**2a**) was identified as a promising oxygen nucleophile when combined with chiral hydrogen bond donor (HBD) catalysts.^14^ For example, the reaction between **1a** and **2a** in the presence of chiral thiourea **B1** (10 mol %) in toluene proceeded smoothly at room temperature to form the desired product **3a** quantitatively with promising enantioselectivity (42% ee, Table 1, entry 1).^15^ Notably, in the absence of a catalyst, the reaction also proceeded to almost completeness, suggesting a strong background reaction that may lead to difficulty in enantiocontrol (entry 2). For comparison, chiral phosphoric acid **A** was also evaluated, which resulted in a racemic product as well, suggesting the importance of matching the acidity of the catalyst and nucleophile (entry 3). Aiming to further improve the enantioselectivity, we screened various other HBD catalysts. Varying the hydrogen-bond functionality to urea (**B2**) or squaramide (**B3**) resulted in inferior enantioselectivity (entries 4–5). Replacing the *tert*-butyl group in **B1** with a bulkier hydroxyladamantyl group (**B4**) did not further improve the selectivity (entry 6). Finally, modification of the amide functionality also led to lower enantioselectivity (entry 7).

**Table 1:**
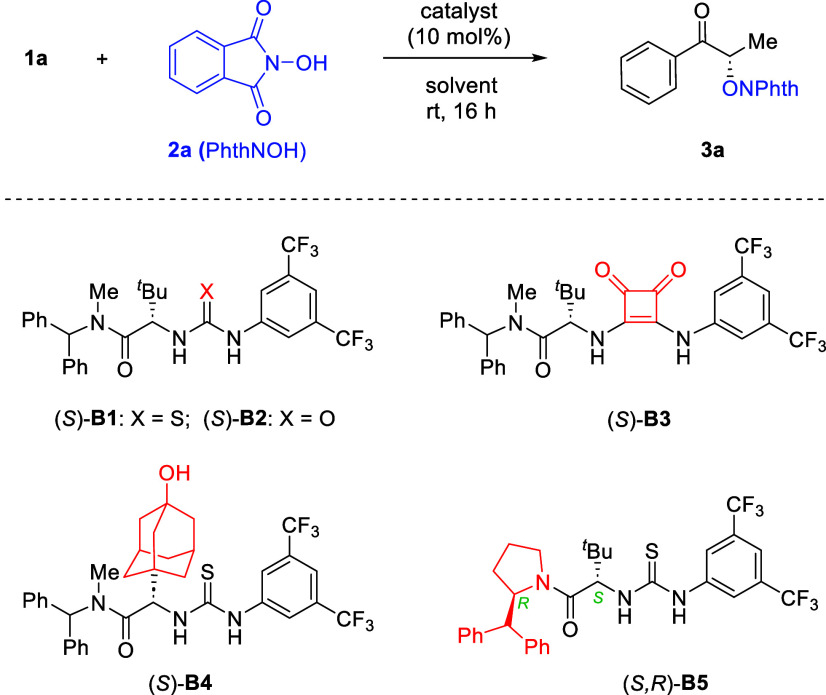
Evaluation of Conditions^a^

entry	catalyst	solvent	yield (%)	ee (%)
1	(*S*)–**B1**	toluene	>95	42
2^b^		toluene	74	0
3	(*S*)-**A**	toluene	>95	0
4	(*S*)–**B2**	toluene	87	13
5	(*S*)–**B3**	toluene	94	4
6	(*S*)–**B4**	toluene	93	38
7	(*S,R*)-**B5**	toluene	97	27
8^c^	(*S*)–**B1**	toluene	>95	85
9^c^	(*S*)–**B1**	CH_2_Cl_2_	>95	48
10^c^	(*S*)–**B1**	THF	>95	64
11^c^	(*S*)–**B1**	EtOAc	>95	75
12^c^	(*S*)–**B1**	*o*-xylene	>95	89
13^c^	(*S*)–**B1**	*m*-xylene	>95	90
14^c^	(*S*)–**B1**	*p*-xylene	>95	82
15^d^	(*S*)–**B1**	*m*-xylene	>95	93

a Reaction conditions: **1a** (0.05 mmol), **2a** (0.06 mmol), catalyst (10 mol %), solvent (0.5 mL), rt, 16 h. Yield was determined to be >95% for all the cases based on the ^1^H NMR spectrum of the crude mixture using CH_2_Br_2_ as the internal standard. Ee was determined by chiral HPLC analysis.

b Clean background reaction.

c Run at −10 °C.

d Run at −30 °C for 48 h.

We further reasoned that the background reaction might be retarded by a decrease in temperature. Moreover, the lower solubility of **2a** at a lower temperature may also benefit enantiocontrol by inhibiting the background reaction. Indeed, as expected, the reaction at −10 °C provided a dramatically improved enantioselectivity (85% ee; entry 8). Next, we screened other reaction solvents. DCM led to substantially lower enantioselectivity (entry 9), which might be due to the high solubility of the reactants and thus a stronger background reaction. Polar solvents, such as EtOAc and THF, were also inferior (entries 10–11). However, *o*- and *m*-xylene could slightly improve the enantioselectivity, but *p*-xylene gave lower enantioselectivity (entries 12–14). Finally, with *m*-xylene as the solvent, further decreasing the temperature to −30 °C provided **3a** with both excellent chemical efficiency and enantioselectivity (entry 15).

Under the optimized conditions, a range of α-keto sulfonium ylides participated smoothly in the intermolecular formal O–H insertion reactions with **2a**, providing access to diverse α-oxygenated ketones in high yield with excellent enantioselectivity (Scheme 3). In all cases, clean conversion was observed with essentially no byproducts. The excellent reactivity and enantioselectivity were so robust that almost no influence was observed by electron-donating or electron-withdrawing groups on the benzene ring directly linked to the carbonyl group. The mild conditions also tolerated diverse functional groups including ether, silyl-protected phenol, halide, nitrile, and silane. Heteroaryl ketones (**3p**–**3q**) were also suitable substrates. The absolute configuration of **3a** was unambiguously confirmed to be *S* by X-ray crystallography.

**Scheme 3:**
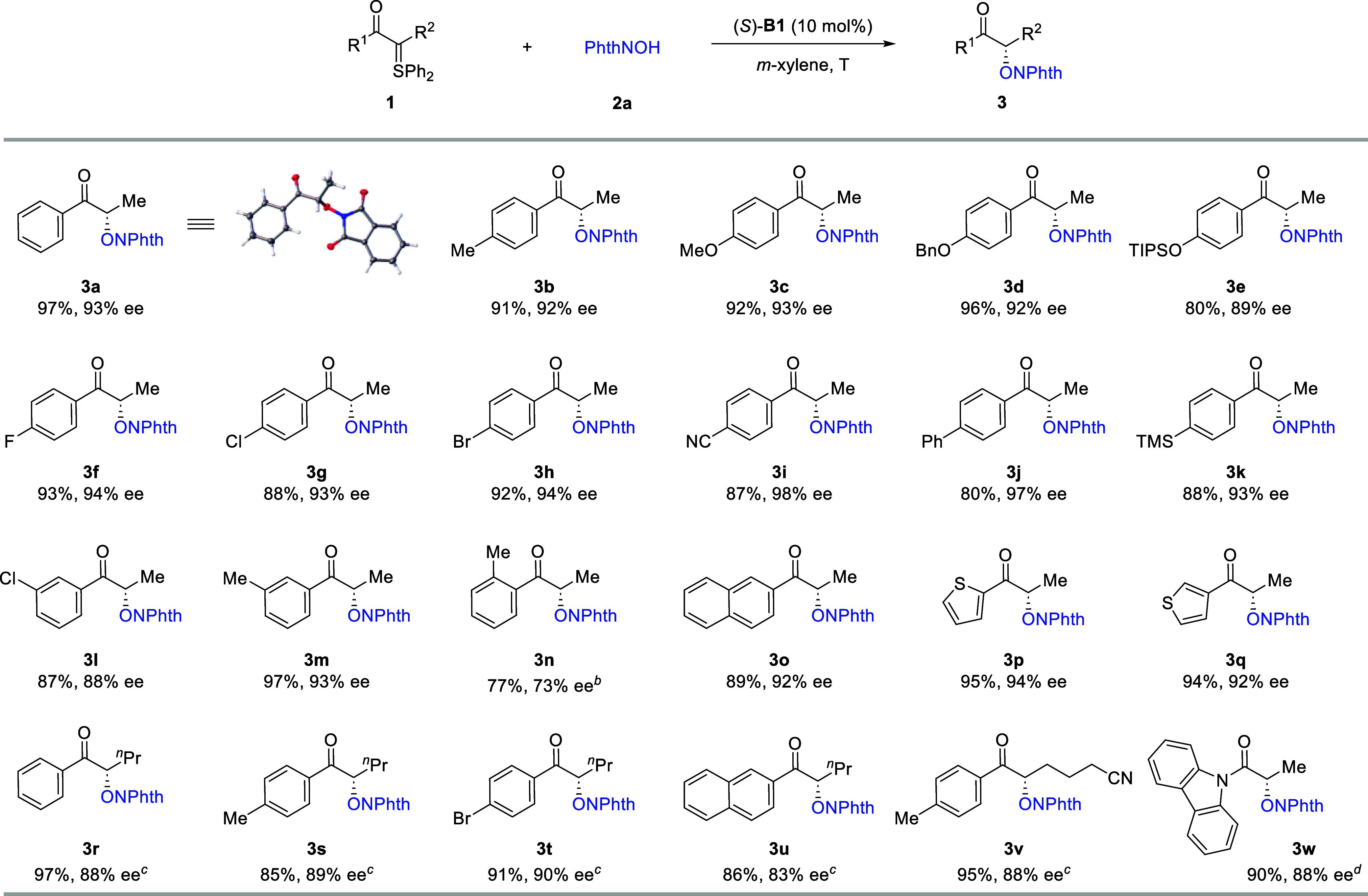
Reaction Scope of Sulfur Ylides[Fn s3fn1]

a
**1** (0.4 mmol), **2a** (0.48 mmol), (*S*)–**B1** (10 mol %), *m*-xylene (4.0 mL), −30 °C, 96 h. Isolated yield.
b Run at −15 °C, 72 h.
c Run with (*S*)–**B4** (10 mol %) as a catalyst in a mixed solvent: *m*-xylene/*c*-hexane = 2:3 (8.0 mL), −20 °C, 72 h.
d Run at −15 °C.

While the standard protocol was highly successful for methyl ketones, it gave relatively low enantioselectivity for substrates bearing a higher alkyl chain. To expand the applicability of this protocol, we performed additional optimizations (see the Supporting Information for details). After considerable efforts, we identified thiourea **B4** as the best catalyst for this type of substrate. Furthermore, a mixture of *m*-xylene and *c*-hexane served as the best solvent. With these modified conditions, the corresponding α-oxygenated ketones **3r**–**3v** were obtained with good to high enantioselectivity. Finally, in addition to ketones, this protocol can also be applied to the synthesis of α-oxygenated amides with high efficiency and enantioselectivity (**3w**). We also evaluated an α-alkyl acyl-substituted sulfur ylide, but it resulted in a very low yield and enantioselectivity (see the Supporting Information for details).

We also explored other oxygen nucleophiles for this process (Scheme 4). Some commercially available substituted *N*-hydroxylphthalimides were examined, which led to equally good results as **2a**, suggesting that the electronic properties of the arene of the nucleophile did not have an obvious impact on the reactivity and enantioselectivity. In addition, 1-hydroxybenzotriazole (HOBt) is another suitable reaction partner for this formal O–H insertion reaction. Under the standard conditions, the corresponding product **4a** was obtained with good efficiency and enantioselectivity. Substitution by a chlorine atom did not influence the outcome. It is worth noting that the N–O bond in the products is expected to be easily cleavable, giving rise to ketones bearing a free hydroxyl group at the α-position that permits further diversifications. Phenol could also serve as a nucleophile, but it showed a relatively low reactivity. At room temperature, the reaction proceeded efficiently to afford the desired product **4c** in good yield but with moderate enantioselectivity.

**Scheme 4:**
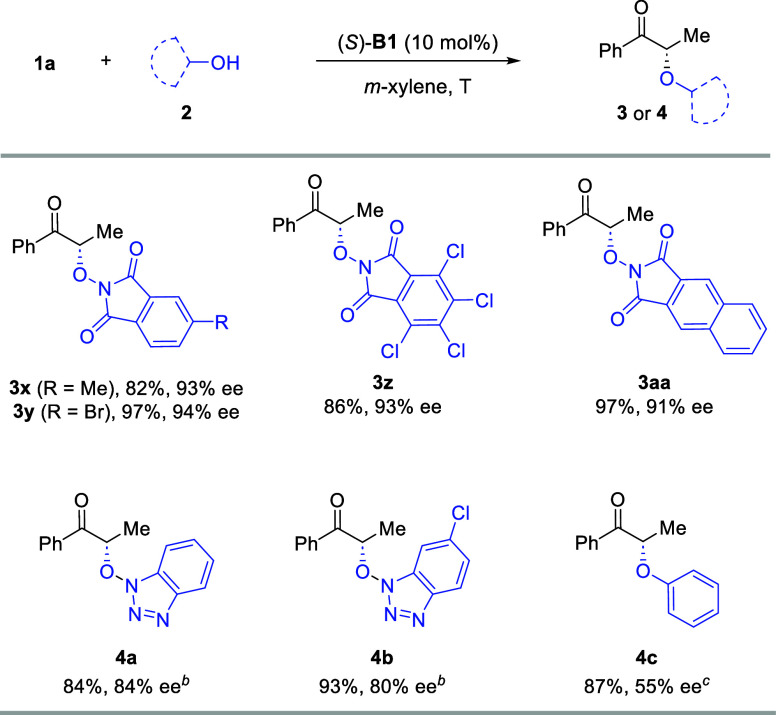
Scope of the Oxygen Nucleophiles^a^

a Reaction Conditions: **1a** (0.4 mmol), **2** (0.48 mmol), (*S*)–**B1** (10 mol %), *m*-xylene (4.0 mL), −30 °C, 96 h. Isolated yield.
b Run for 72 h.
c Run at rt for 48 h.

Without modification, the standard protocol was successfully employed for a gram-scale synthesis of **3a** without compromise of chemical efficiency and enantioselectivity (Scheme 5). The leaving group Ph_2_S and catalyst **B1** could both be recovered in a high yield. The highly enantioenriched α-oxygenated ketone **3a** could be easily transformed into other chiral building blocks. For example, Baeyer–Villiger oxidation by *m*-CPBA afforded ester **5**, a derivative of *l* ­(+)-lactic acid, in high yield. Notably, it was also possible to directly synthesize compound **5** via the enantioselective formal O–H bond insertion of ester sulfonium ylide **1ab**. However, a moderate yield and low enantioselectivity were observed under the standard conditions. Furthermore, the O–N bond could be easily cleaved by iron powder under acidic conditions, leading to free α-hydroxyl ketone **6**, which was a key intermediate in the synthesis of a type of HIF-1α inhibitors called Manassantin analogues.^16^ Finally, the enantioenriched ketone **6** was also demonstrated as a useful precursor to different chiral 1,2-diols upon treatment with different nucleophiles such as Grignard reagents, organolithium reagents, and hydrides. In all these transformations, no obvious erosion in the high enantiopurity was observed.

**Scheme 5:**
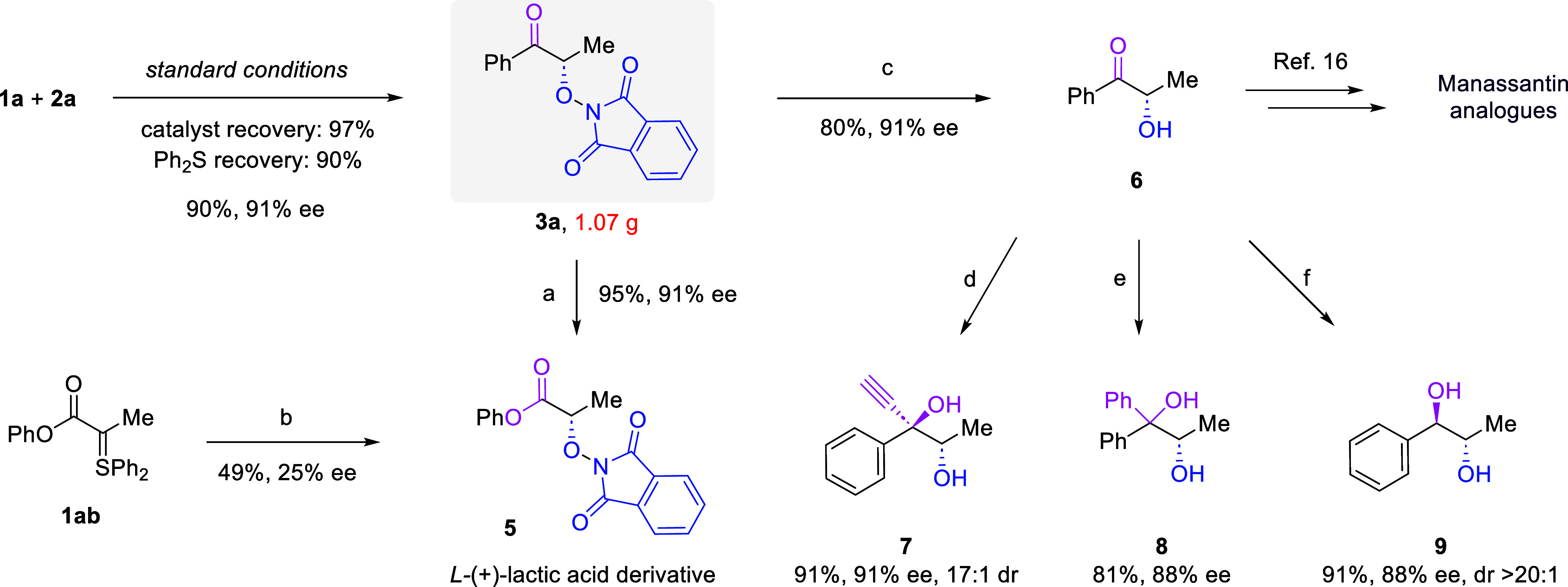
Scale-up Reaction and Product Transformations^a^

a (a) *m-*CPBA, TFA, CH_2_Cl_2_, rt, 16 h. (b) **1ab** (0.4 mmol), **2a** (0.48 mmol), (*S*)–**B4** (10 mol %), *m*-xylene (4.0 mL), −15 °C, 72 h. (c) Fe, NH_4_Cl, EtOH/H_2_O, 80 °C, 4 h. (d) HC≡CMgBr, THF, −78 °C → 0 °C, 1.5 h. (e) PhLi, THF, −78 °C → 0 °C, 40 min. (f) Zn­(BH_4_)_2_, THF, −78 °C → 0 °C, 40 min.

To gain more information about the reaction mechanism, we performed some experiments. First of all, product enantiomeric excess (ee) values showed a linear correlation with the *ee* values of the catalyst, which is consistent with the involvement of one catalyst molecule in the enantiodetermining transition state (see the Supporting Information for more details). Next, to know more about the catalytic activation mode, the possible interaction between the catalyst and each reactant was investigated (Figure 1). It is worth noting that both substrates had a limited solubility in CDCl_3_. When catalyst **B1** was mixed with nucleophile **2a**, no change in the ^1^H NMR signals was observed. In contrast, when **B1** was treated with sulfonium ylide **1a**, the two N–H signals in the thiourea motif were dramatically shifted downfield. Moreover, a higher loading of **1a** led to a more pronounced shift, suggesting a reversible interaction between **B1** and **1a**.

**Figure 1:**
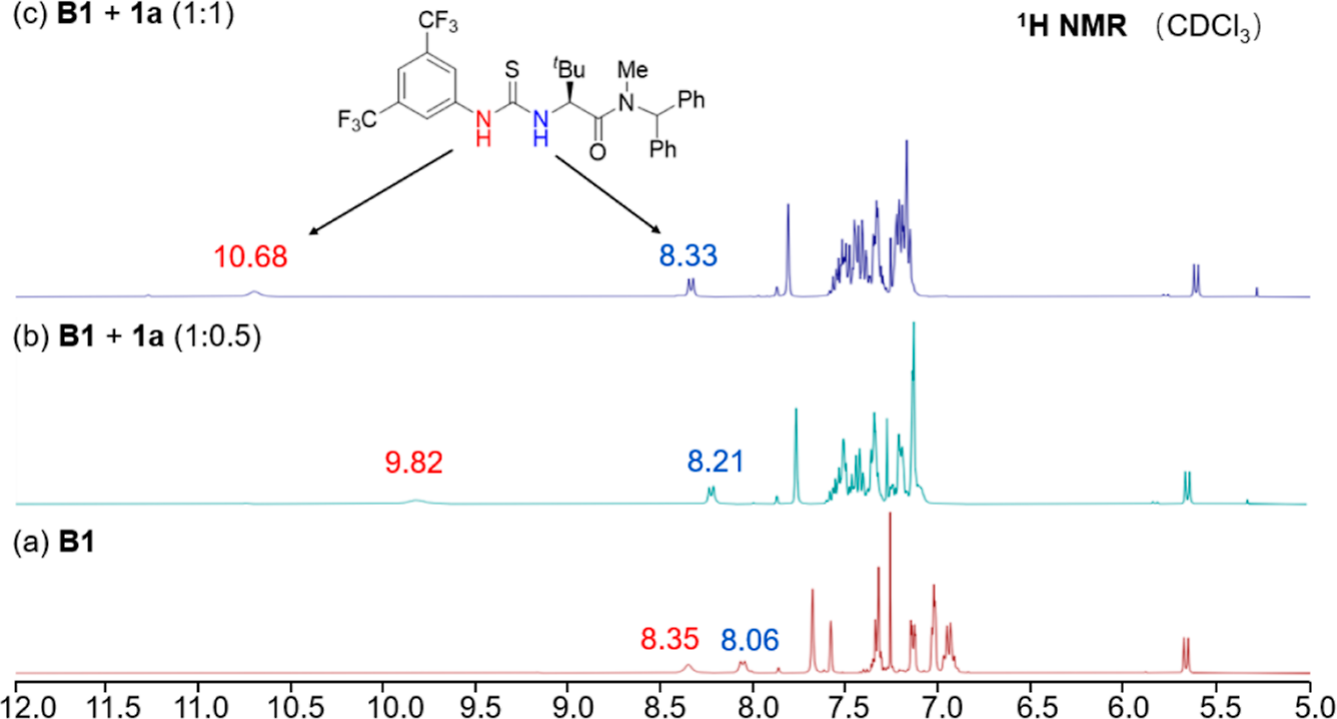
^1^H NMR study of the interaction between **B1** and **1a**.

Based on the above results, we proposed a possible mechanism using **1a** and **2a** as model substrates (Figure 2). The reaction begins with activation of the sulfonium ylide **1a** by the thiourea catalyst **B1** via hydrogen bonding. The activated form **IM1** is then protonated by the acidic nucleophile **2a** to form chiral ion pair **IM2A**, which has an increased solubility in organic solvent relative to both reactants. This explains the rate acceleration of the catalytic pathway since the background reaction is suppressed by the low solubility of both substrates. Next, the counteranion serves as the nucleophile and undergoes substitution of the sulfonium motif to form the desired product, with the enantioselectivity controlled by the chiral catalyst, possibly via dynamic kinetic resolution. Nevertheless, although catalyst activation of sulfonium **1a** is preferred in the first step, this does not exclude the possibility of another scenario indicated by **IM2B**. After the protonation, the increased solubility and basicity of the oxygen anion (conjugate base of **2a**) becomes a better hydrogen bonding acceptor (than carbonyl) and causes an equilibrium toward the formation of a different chiral ion pair **IM2B**. In this case, the HBD-bound chiral anion serves as the nucleophile for the substitution and controls the stereochemistry in a similar dynamic kinetic resolution pathway.^17,18^

**Figure 2:**
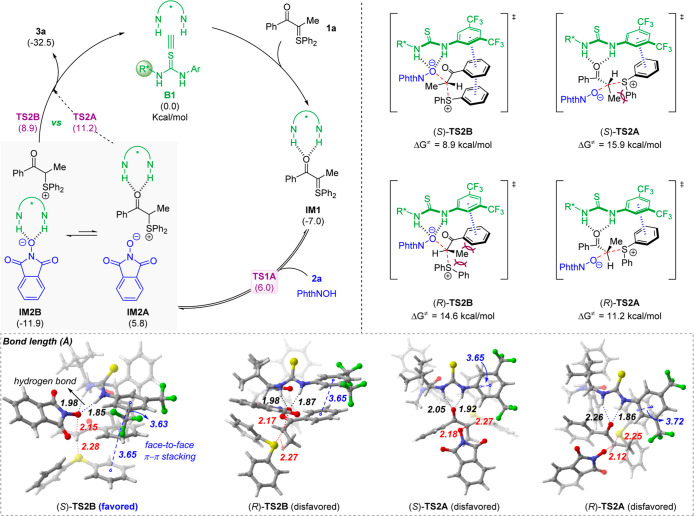
Proposed mechanism and computed transition states.

It is difficult to differentiate the two pathways by experiments. Therefore, density functional theory (DFT) studies were performed to gain additional insights. Indeed, complex **IM1** is thermodynamically more stable than the complex **IM1′** from **B1** and **2a** (see Figure S1 for details), in agreement with the observation from NMR experiments. The protonation leading to chiral ion pair **IM2A** is a reversible step. More importantly, **IM2A** can easily equilibrate to the more stable chiral ion pair **IM2B**, as initially hypothesized. Notably, the facile epimerization of the α-chirality of this type of sulfonium salt was experimentally observed before.^17a^ Nevertheless, the relative stability of these species does not determine the key C–O-bond-forming pathway, which is the rate-determining step. Indeed, all four possible transition states were calculated, corresponding to both pathways (via **IM2A** and **IM2B**) and two enantiomers in each pathway. The calculation results suggest that C–O formation via **IM2B** is more favorable for the major enantiomer. Specifically, in the enantiomerically favored transition state (*S*)-**TS2B**, a stronger hydrogen bond in the donor-bound chiral anion and a favorable π–π interaction are present, which facilitate the substitution process when compared with **TS2A**. Notably, for the pathway leading to the minor enantiomer, the transition state (*R*)-**TS2A** from **IM2A** appears to be more favored. Overall, the calculated energy difference of 2.3 kcal/mol between (*S*)-**TS2B** and (*R*)-**TS2A** agrees with the observed experimental enantioselectivity.

## Conclusion

In summary, we have developed an example of organocatalytic enantioselective formal O–H bond insertion of α-carbonyl sulfur ylides, leading to highly efficient synthesis of the valuable ketones bearing a tertiary α-oxygenated stereocenter. This mild and metal-free approach is complementary to the well-established metal-catalyzed diazo carbonyl chemistry via metal carbenes that has gained broad success for esters but not for ketones. The proper choice of a suitable chiral thiourea catalyst in combination with *N*-hydroxyl phthalimides and analogues as nucleophiles proved to be crucial to success. This is also distinct from previous enantioselective X–H insertion reactions of sulfonium ylides, featuring the use of chiral phosphoric acids that involve protonation as a key step. The mild conditions in this protocol tolerated a range of functional groups, and the products could be useful precursors to other chiral building blocks. Mechanistic studies indicate that the reaction is likely initiated by activation of sulfonium ylides by hydrogen bonding, which is distinct from previous related cases using chiral phosphoric acid catalysis that involves facile protonation of the basic sulfonium ylide functionality. DFT studies also provided important insights into differentiating the possible pathways.

## Supplementary Material



## Notes

Additional experimental and computational details and spectroscopic data of all compounds (PDF)
