# Supplementary material for: Enantioselective Synthesis of α‑Oxygenated Ketones via Organocatalytic Formal O–H Bond Insertion of Sulfonium Ylides
Source: J Am Chem Soc. 2025 Aug 20;147(35):32110–7. doi: 10.1021/jacs.5c10842 (PMC12412150; doi:10.1021/jacs.5c10842)
Supplement: Supplementary file 1 [file ja5c10842_si_001.pdf]

Supporting Information

Enantioselective Synthesis of α-Oxygenated Ketones via Organocatalytic Formal O–H Bond Insertion of Sulfonium Ylides

Chenxiao Qian,[ab] Ziwei Zhong,[a] Qingzheng Xu,[a] Pengfei Li,[b*]
Chaoshen Zhang,[a*] and Jianwei Sun[a*]

[a]*Department of Chemistry and the Hong Kong Branch of Chinese National Engineering Research Centre for Tissue Restoration & Reconstruction, The Hong Kong University of Science and Technology, Clear Water Bay, Kowloon, Hong Kong SAR 999077, China*

[b]*Department of Chemistry, Guangdong Provincial Key Laboratory of Catalysis, College of Science, Southern University of Science and Technology, Shenzhen, Guangdong, 518055, China*

Table of Contents

I. General Information S-2

II. Substrate Preparation S-3

III. Synthesis of Catalyst B4 .... S-15

IV. Synthesis of Chiral α-Oxygenated Ketones and Amides S-17

V. Scale-up Reaction and Product Derivatizations S-41

VI. Mechanistic Study S-47

VII. DFT Calculations S-50

VIII. Determination of the Stereochemistry S-91

HPLC Traces and NMR Spectra

I. General Information

Flash column chromatography was performed over silica gel (200-300 mesh) purchased from Qingdao Puke Co., China. All air or moisture sensitive reactions were conducted in oven-dried glassware under a nitrogen atmosphere using anhydrous solvents. Anhydrous dichloromethane, toluene, diethyl ether, and tetrahydrofuran were purified by the Innovative® solvent purification system. Reagents were purchased from commercial suppliers and used without further purification unless otherwise stated. All heating was achieved by an oil bath. The single crystal was obtained by slowly volatilizing of saturated CH_2Cl_2 solution at room temperature. ^{1}H, ^{13}C and ^{19}F spectra were collected on a Bruker AV 400 MHz or 600 MHz NMR spectrometer using residue solvent peaks as an internal standard (^{1}H NMR: $CDCl_3$ at 7.26 ppm, DMSO-d_6 at 2.50 ppm, acetone-d_6 at 2.05 ppm; ^{13}C NMR: $CDCl_3$ at 77.16 ppm, DMSO-d_6 at 39.52 ppm, acetone-d_6 at 29.84 ppm). Data for ^{1}H NMR were recorded as follows: chemical shift (δ, ppm), multiplicity (s = singlet; d = doublet; t = triplet; q = quarter; p = pentet; m = multiplet; br = broad), coupling constant (Hz), integration. Mass spectra were collected on an Agilent GC/MS 5975C system, a MALDI Micro MX mass spectrometer, or an API QSTAR XL System. Optical rotations were measured on JASCO P-2000 polarimeter with $[\alpha]_D$ values reported in degrees; concentration (c) is in 2.0 mg/mL. The enantiomeric excess values were determined by chiral HPLC using an Agilent 1200 LC instrument with a Daicel CHIRALCEL® OD-3, CHIRALPAK® IC-3, CHIRALPAK® ID-3 or CHIRALPAK® IG-3 columns. The instrumentation used for the crystal measurement was Bruker D8 VENTUR.

II. Substrate Preparation

α-Keto sulfur ylides **1a**-**1c**, **1f**, **1h**, **1i**, **1l**, **1o**, **1p**, **1s**, and **1v** are known compounds.[1] The α-keto sulfur ylides **1** were prepared according to the reported procedure.[1]

General Procedure A.

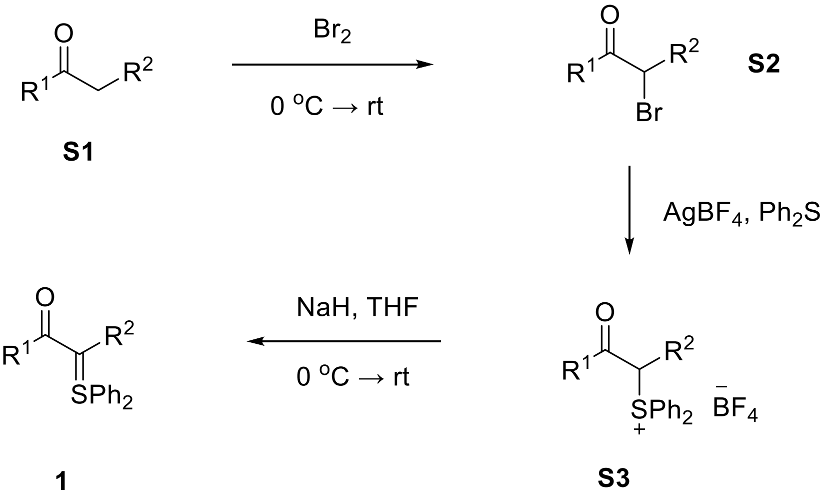

At 0 °C, to a solution of ketone **S1** (10.0 mmol) in the solvent (30 mL, Et_2O for **1d**, **1e**, **1n** and **1q** to avoid over-bromination, and CH_2Cl_2 for the others) was slowly added liquid bromine (0.51 mL, 1.0 equiv, 10.0 mmol). The mixture was stirred at room temperature for 1–3 h until the color of bromine dissipated. Upon completion, the mixture was quenched by a saturated aqueous $NaHCO_3$ solution (30 mL). The solution was extracted with the corresponding solvent (10 mL× 3 times, Et_2O or CH_2Cl_2). The combined organic layers were washed by brine (30 mL) and dried over anhydrous Na_2SO_4, filtered, and concentrated under reduced pressure. The crude product was used for the next step without purification.

---

(1) Guo, W.; Luo, Y.; Herman; Williams, I. D.; Li, P.; Sun, J. Chiral Phosphoric Acid Catalyzed Enantioselective Synthesis of α-Tertiary Amino Ketones from Sulfonium Ylides. *J. Am. Chem. Soc.* **2020**, *142*, 14384–14390.

Under N_2, to a 100-mL round-bottom flask were added the above crude product, diphenyl sulfide (3.72 g, 2.0 equiv, 20.0 mmol), $AgBF_4$ (1.95 g, 1.0 equiv, 10.0 mmol), and dry DCE (40 mL). The mixture was stirred at 80 °C for 24 h before it was cooled to room temperature and filtered through a short pad of celite to remove the generated silver bromide. The celite was washed with CH_2Cl_2 (100 mL), and the filtrate was concentrated *in vacuo*. The residue was purified by flash column chromatography on silica gel to afford the desired sulfonium salt (eluent: CH_2Cl_2 followed by EtOAc).

Under N_2 at 0 °C, to an over-dried 100-mL flask charged with the sulfonium salt (1.0 equiv) and dry THF (0.1 M) was added NaH (2.0 equiv, 60 wt% in mineral oil) portionwise. The resulting suspension was slowly warmed to room temperature and stirred for 3 h. Then, the reaction mixture was filtered through a short pad of celite, which was washed with CH_2Cl_2 (5 mL per mmol of substrate). The filtrate was concentrated *in vacuo*, and the residue was washed with *n*-hexane/CH_2Cl_2 (10:1, 5.0 mL per mmol of substrate). The solid was dried *in vacuo* to give the pure sulfur ylide **1**, which was used directly without further purification.

Note: All the obtained ylides are mixtures of s-cis and s-trans isomers (shown below). It is impossible to isolate the pure form of either isomer since they are in equilibrium.

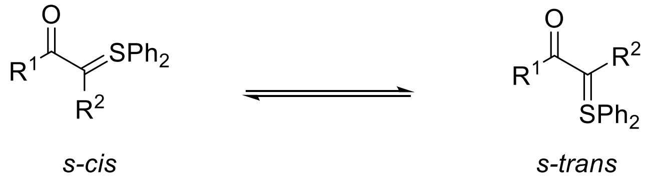

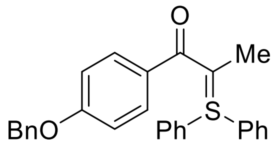

1d

1-(4-(Benzyloxy)phenyl)-2-(diphenyl-λ⁴-sulfaneylidene)propan-1-one (1d) was prepared as a white solid from 1-(4-(benzyloxy)phenyl)propan-1-one (2.4 g, 10.0 mmol) according to the General Procedure A (in Et_2O for 3 h for the first step) in 37% overall yield (1.5 g, *s-cis*/*trans* = 5:1).

Major isomer:

^{1}H NMR (400 MHz, $CDCl_3$) δ 7.57 – 7.50 (m, 7H), 7.41 – 7.35 (m, 9H), 7.33 – 7.29 (m, 1H), 6.95 – 6.91 (m, 2H), 5.06 (s, 2H), 1.90 (s, 3H) ppm.

^{13}C NMR (151 MHz, $CDCl_3$) δ 186.9, 158.9, 137.1, 135.9, 132.0, 131.3, 131.2, 130.0, 130.0, 129.7, 129.5, 129.2, 128.9, 128.6, 128.0, 127.5, 114.2, 70.1, 65.0, 10.3 ppm.

HRMS (ES+) Calcd for $C_{28}H_{25}O_2S^+$ $[M+H]^+$: 425.1570, Found: 425.1571.

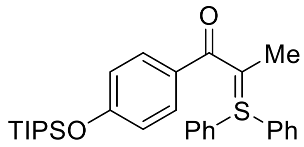

1e

2-(Diphenyl-λ⁴-sulfaneylidene)-1-(4-((triisopropylsilyl)oxy)phenyl)propan-1-one (1e) was prepared as a pale-yellow solid from 1-(4-((triisopropylsilyl)oxy)phenyl)propan-1-one (3.1 g, 10.0 mmol) according to the General Procedure A (in Et_2O for 3 h for the first step) in 33% overall yield (1.62 g,*s-cis*/*trans* = 5:1).

Major isomer:

^{1}H NMR (400 MHz, $CDCl_3$) δ 7.50 – 7.48 (m, 7H), 7.36 – 7.35 (m, 3H), 7.31 – 7.26 (m, 2H), 6.82 – 6.80 (m, 2H), 1.88 (s, 3H), 1.25 – 1.19 (m, 3H), 1.08 – 1.02 (m, 18H) ppm.

^{13}C NMR (151 MHz, $CDCl_3$) δ 187.1, 156.2, 135.9, 132.0, 131.3, 131.1, 129.9, 129.5, 129.2, 129.1, 128.8, 127.1, 119.3, 65.1, 18.0, 12.7, 10.2 ppm.

HRMS (ES+) Calcd for $C_{30}H_{39}O_2SSi^+$ $[M+H]^+$: 491.2435, Found: 491.2442.

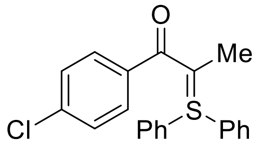

1g

1-(4-Chlorophenyl)-2-(diphenyl-λ[4]-sulfaneylidene)propan-1-one (1g) was prepared as a white solid from 1-(4-chlorophenyl)propan-1-one (1.7 g, 10.0 mmol) according to the General Procedure A (in CH_2Cl_2 for 2 h for the first step) in 51% overall yield (1.8 g, *s-cis/trans* = 5:1).

Major isomer:

^{1}H NMR (400 MHz, $CDCl_3$) δ 7.55 – 7.48 (m, 7H), 7.37 – 7.26 (m, 7H), 1.89 (s, 3H) ppm.

^{13}C NMR (151 MHz, $CDCl_3$) δ 185.8, 141.5, 133.9, 131.6, 131.5, 130.1, 130.1, 129.5, 129.4, 129.0, 128.8, 128.2, 128.0, 66.2, 10.0 ppm.

HRMS (ES+) Calcd for $C_{21}H_{18}ClOS^+$ $[M+H]^+$: 353.0762, Found: 353.0763.

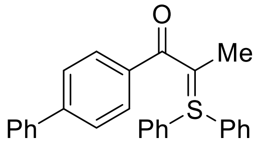

1j

1-([1,1'-Biphenyl]-4-yl)-2-(diphenyl-λ[4]-sulfaneylidene)propan-1-one (1j) was prepared as a yellowish brown solid from 1-([1,1'-biphenyl]-4-yl)propan-1-one (2.1 g, 10.0 mmol) according to the General Procedure A (in CH_2Cl_2 for 2 h for the first step) in 46% overall yield (1.8 g, *s-cis/trans* = 6:1).

Major isomer:

^{1}H NMR (400 MHz, $CDCl_3$) δ 7.64 – 7.47 (m, 13H), 7.41 – 7.39 (m, 5H), 7.33 – 7.30 (m, 1H), 1.93 (s, 3H) ppm.

^{13}C NMR (151 MHz, $CDCl_3$) δ 186.9, 142.1, 141.0, 140.9, 131.8, 131.4, 131.3, 130.0, 129.5, 128.9, 128.8, 128.5, 128.1, 127.3, 127.1, 126.8, 126.6, 65.7, 10.1 ppm.

HRMS (ES+) Calcd for $C_{27}H_{23}OS^+$ $[M+H]^+$: 395.1465, Found: 395.1471.

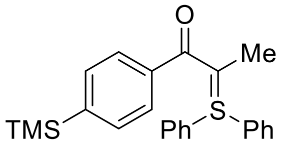

1k

2-(Diphenyl-λ^4-sulfaneylidene)-1-(4-(trimethylsilyl)phenyl)propan-1-one (1k) was prepared as a yellow solid from 1-(4-(trimethylsilyl)phenyl)propan-1-one (1.05 g, 5.0 mmol) according to the General Procedure A (in CH_2Cl_2 for 2 h for the first step) in 50% overall yield (0.98 g, *s-cis/trans* = 6.5:1).

Major isomer:

^{1}H NMR (400 MHz, $CDCl_3$) δ 7.54 – 7.45 (m, 9H), 7.39 – 7.36 (m, 5H), 1.90 (s, 3H), 0.24 (s, 9H) ppm.

^{13}C NMR (151 MHz, $CDCl_3$) δ 187.3, 143.5, 140.2, 133.0, 132.9, 131.9, 131.4, 131.3, 130.0, 129.5, 128.9, 127.1, 126.8, 65.5, 10.0, -1.0 ppm.

HRMS (ES+) Calcd for $C_{24}H_{27}OSSi^+$ $[M+H]^+$: 391.1547, Found: 391.1551.

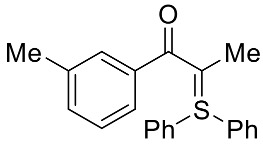

1m

2-(Diphenyl-λ^4-sulfaneylidene)-1-(*m*-tolyl)propan-1-one (1m) was prepared as a white solid from 1-(*m*-tolyl)propan-1-one (1.5 g, 10.0 mmol) according to the General Procedure A (in CH_2Cl_2 for 2 h for the first step) in 51% overall yield (1.7 g, *s-cis/trans* = 6:1).

Major isomer:

^{1}H NMR (400 MHz, $CDCl_3$) δ 7.54 – 7.47 (m, 6H), 7.38 – 7.25 (m, 4H), 7.19 – 7.12 (m, 4H), 2.31 (s, 3H), 1.90 (s, 3H) ppm.

^{13}C NMR (151 MHz, $CDCl_3$) δ 187.2, 143.0, 137.5, 131.7, 131.3, 131.2, 129.9, 129.3, 128.71, 128.67, 128.4, 128.2, 127.6, 127.5, 124.5, 65.4, 21.4, 9.9 ppm.

HRMS (ES+) Calcd for $C_{22}H_{21}OS^+$ $[M+H]^+$: 333.1308, Found: 333.1315.

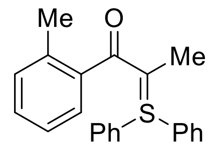

1n

2-(Diphenyl-λ⁴-sulfaneylidene)-1-(*o*-tolyl)propan-1-one (1n) was prepared as a brown solid from 1-(*o*-tolyl)propan-1-one (1.5 g, 10.0 mmol) according to the General Procedure A (in Et_2O for 2 h for the first step) in 54% overall yield (1.8 g, *s-cis/trans* = 8:1).

Major isomer:

^{1}H NMR (400 MHz, $CDCl_3$) δ 7.52 – 7.33 (m, 10H), 7.20 – 7.08 (m, 4H), 2.35 (s, 3H), 1.94 (s, 3H) ppm.

^{13}C NMR (151 MHz, $CDCl_3$) δ 187.1, 143.0, 135.2, 131.9, 131.41, 131.37, 130.3, 130.1, 130.0, 129.5, 128.94, 128.89, 127.6, 127.3, 125.2, 67.1, 19.4, 9.4 ppm.

HRMS (ES+) Calcd for $C_{22}H_{21}OS^+$ $[M+H]^+$: 333.1308, Found: 333.1301.

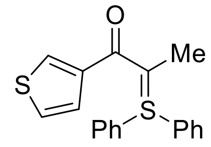

1q

2-(Diphenyl-λ⁴-sulfaneylidene)-1-(thiophen-3-yl)propan-1-one (1q) was prepared as a pale-yellow solid from 1-(thiophen-3-yl)propan-1-one (0.7 g, 5.0 mmol) according to the General Procedure A (in Et_2O for 3 h for the first step) in 20% overall yield (0.32 g, *s-cis/trans* = 4:1).

Major isomer:

^{1}H NMR (400 MHz, DMSO-d_6) δ 7.64 – 7.60 (m, 6H), 7.52 – 7.31 (m, 6H), 7.27 – 7.10 (m, 1H), 1.70 (s, 3H) ppm.

^{13}C NMR (151 MHz, $CDCl_3$) δ 181.5, 143.7, 131.7, 131.4, 131.2, 130.0, 129.9, 129.3, 128.7, 128.2, 125.4, 124.8, 123.7, 66.0, 10.2 ppm.

HRMS (ES+) Calcd for $C_{19}H_{17}OS_2^+$ [M+H]$^+$: 325.0716, Found: 325.0726.

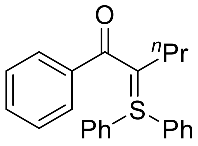

1r

2-(Diphenyl-λ^4-sulfaneylidene)-1-phenylpentan-1-one (1r) was prepared as a pale-yellow solid from 1-phenylpentan-1-one (1.6 g, 10.0 mmol) according to the General Procedure A (in CH_2Cl_2 for 2 h for the first step) in 46% overall yield (1.6 g, *s-cis/trans* = 5:1).

Major isomer:

^{1}H NMR (400 MHz, DMSO-*d*$_6$) δ 7.61 – 7.25 (m, 15H), 2.30 (t, *J* = 7.8 Hz, 2H), 0.96 – 0.91 (m, 2H), 0.57 (t, *J* = 7.2 Hz, 3H) ppm.

^{13}C NMR (151 MHz, $CDCl_3$) δ 187.4, 143.6, 133.3, 131.3, 131.2, 129.9, 129.7, 129.6, 128.8, 127.9, 127.8, 127.6, 127.2, 70.6, 28.9, 22.3, 14.1 ppm.

HRMS (ES+) Calcd for $C_{23}H_{23}OS^+$ [M+H]$^+$: 347.1465, Found: 347.1471.

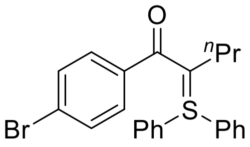

1t

1-(4-Bromophenyl)-2-(diphenyl-λ^4-sulfaneylidene)pentan-1-one (1t) was prepared as a pale-yellow solid from 1-(4-bromophenyl)pentan-1-one (2.4 g, 10.0 mmol) according to the General Procedure A (in CH_2Cl_2 for 2 h for the first step) in 45% overall yield (1.9 g, *s-cis/trans* = 5:1).

Major isomer:

^{1}H NMR (400 MHz, DMSO-*d*$_6$) δ 7.61 – 7.45 (m, 12H), 7.36 – 7.21 (m, 2H), 2.29 (t, *J* = 7.7 Hz, 2H), 0.95 – 0.89 (m, 2H), 0.55 (t, *J* = 7.1 Hz, 3H) ppm.

^{13}C NMR (151 MHz, $CDCl_3$) δ 185.9, 142.3, 132.9, 131.5, 131.3, 131.0, 130.0, 129.8, 129.6, 129.3, 129.1, 128.8, 121.9, 71.2, 28.9, 22.2, 14.1 ppm.

HRMS (ES+) Calcd for $C_{23}H_{22}BrOS^+$ $[M+H]^+$: 425.0570, Found: 425.0576.

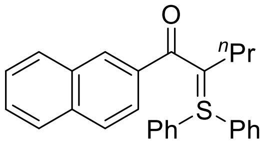

1u

2-(Diphenyl-λ⁴-sulfaneylidene)-1-(naphthalen-2-yl)pentan-1-one (1u) was prepared as a yellowish brown solid from 1-(naphthalen-2-yl)pentan-1-one (2.1 g, 10.0 mmol) according to the General Procedure A (in CH_2Cl_2 for 2 h for the first step) in 30% overall yield (1.2 g, *s-cis/trans* = 4:1).

Major isomer:

^{1}H NMR (400 MHz, $CDCl_3$) δ 7.61 – 7.28 (m, 15H), 7.16 – 7.12 (m, 2H), 2.43 (t, *J* = 7.1 Hz, 2H), 1.13-1.03 (m, 2H), 0.68 (t, *J* = 6.2 Hz, 3H) ppm.

^{13}C NMR (151 MHz, $CDCl_3$) δ 187.5, 140.6, 137.62, 137.55, 133.4, 131.6, 131.2, 131.1, 131.0, 130.0, 129.62, 129.57, 129.2, 128.76, 128.75, 128.5, 128.4, 127.6, 127.3, 70.2, 29.0, 22.2, 14.1 ppm.

HRMS (ES+) Calcd for $C_{27}H_{25}OS^+$ $[M+H]^+$: 397.1621, Found: 397.1627.

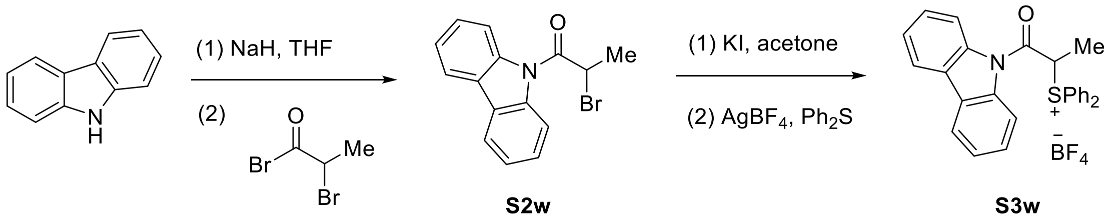

Synthesis of (1-(9*H*-carbazol-9-yl)-1-oxopropan-2-yl)diphenylsulfonium tetrafluoroborate (S3w). Under N_2 at 0 °C, to an over-dried 100-mL flask charged with carbazole (1.67 g, 1.0 equiv, 10.0 mmol) and dry THF (50 mL) was added NaH (0.60 g, 1.5 equiv, 60 wt% in mineral oil, 15.0 mmol) portionwise. The suspension was slowly warmed to room temperature and stirred for 2 h before 2-bromopropionyl bromide (2.1 mL, 4.31 g, 2.0 equiv, 20.0 mmol) was added. Then, the reaction mixture was stirred for an additional 3 h. Upon

completion, it was quenched by a saturated aqueous $NaHCO_3$ solution (50 mL) and extracted with EtOAc (30 mL × 3). The combined organic layers were washed by an aqueous $NaHCO_3$ solution (50 mL) and brine (50 mL), and dried over anhydrous Na_2SO_4, filtered, and concentrated under reduced pressure. The residue was redissolved in CH_2Cl_2 (20 mL) and filtered through a short pad of silica gel. The silica gel was washed with CH_2Cl_2 (50 mL). The filtrate was concentrated *in vacuo*, and the crude bromide **S2w** was used for the next step without purification. (*Note: The residue was the mixture of carbazole and the desired bromide. Unfortunately, it was difficult to separate the desired bromide from carbazole, but this did not influence the subsequent reactions.*)

To a 100-mL round-bottom flask were added the crude **S2w**, KI (1.66 g, 1.0 equiv, 10.0 mmol), and acetone (20 mL). The mixture was stirred at room temperature for 24 h and then filtered through a short pad of celite to remove the generated KBr. The celite pad was washed with CH_2Cl_2 (30 mL), and the filtrate was concentrated *in vacuo*. The residue was redissolved in CH_2Cl_2 (30 mL), to which were added diphenyl sulfide (3.72 g, 2.0 equiv, 20.0 mmol) and $AgBF_4$ (1.95 g, 1.0 equiv, 10.0 mmol). The mixture was stirred at room temperature for 24 h before it was filtered through a short pad of celite to remove the generated silver iodide. The celite pad was washed with CH_2Cl_2 (50 mL), and the filtrate was concentrated *in vacuo*. The residue was purified by flash column chromatography on silica gel to afford the desired sulfonium salt **S3w** as a white solid (eluent: CH_2Cl_2/EtOAc = 20:1 → 1:1, 0.92g, 19% yield over 4 steps).

^{1}H NMR (400 MHz, $CDCl_3$) δ 8.29 – 8.27 (m, 4H), 8.20 – 8.17 (m, 2H), 7.92 (d, *J* = 4.2 Hz, 2H), 7.85 – 7.58 (m, 8H), 7.42 (t, *J* = 7.5 Hz, 2H), 6.88 (q, *J* = 7.2 Hz, 1H), 1.92 (d, *J* = 7.2 Hz, 3H) ppm.

^{13}C NMR (151 MHz, $CDCl_3$) δ 166.1, 135.6, 134.9, 132.8, 132.2, 131.7, 130.8, 127.06, 127.05, 125.7, 124.8, 121.4, 120.1, 63.5, 15.3 ppm.

HRMS (ES+) Calcd for $C_{27}H_{22}NOS^+$ $[M-BF_4]^+$: 408.1417, Found: 408.1425.

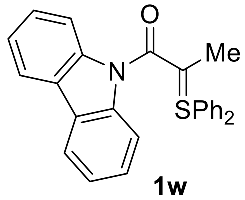

1w

1-(9*H*-Carbazol-9-yl)-2-(diphenyl-λ^4-sulfaneylidene)propan-1-one (1w) was prepared as a pale-yellow solid from **S3w** (495 mg, 1.0 mmol) according to the last step of the General Procedure A (384 mg, 94% yield, *s-cis*/*trans* = 2:1).

Major isomer:

^{1}H NMR (400 MHz, $CDCl_3$) δ 8.11 – 8.06 (m, 2H), 7.68 – 7.35 (m, 11H), 7.29 – 7.22 (m, 5H), 2.13 (s, 3H) ppm.

^{13}C NMR (151 MHz, $CDCl_3$) δ 165.5, 140.6, 131.7, 131.6, 131.1, 130.3, 130.0, 129.3, 128.9, 125.8, 123.4, 120.2, 119.9, 119.7, 111.3, 61.7, 10.8 ppm.

HRMS (ES+) Calcd for $C_{27}H_{22}NOS^+$ $[M+H]^+$: 408.1417, Found: 408.1425.

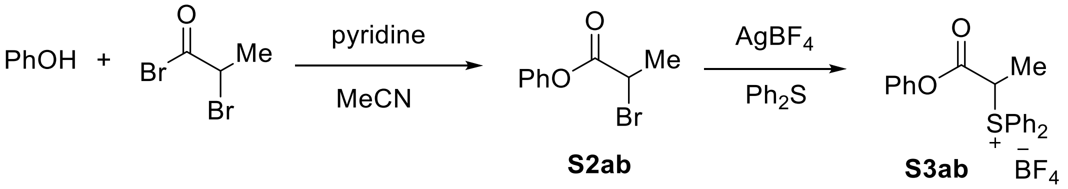

(1-Oxo-1-phenoxypropan-2-yl)diphenylsulfonium tetrafluoroborate (S3ab). Under N_2 at 0 °C, to an oven-dried 100-mL flask charged with phenol (0.94 g, 1.0 equiv, 10.0 mmol), pyridine (1.58 g, 2.0 equiv, 20.0 mmol), and dry MeCN (10 mL) was added 2-bromopropionyl bromide (2.1 mL, 4.31 g, 2.0 equiv, 20.0 mmol) dropwise. The mixture was warmed slowly to room temperature and stirred for 30 min. Upon completion, it was quenched by a saturated aqueous $NaHCO_3$ solution (20 mL) and extracted with EtOAc (10 mL × 3). The combined organic layers were washed by brine (20 mL), dried over anhydrous Na_2SO_4, filtered, and concentrated under reduced pressure. The residue was redissolved in CH_2Cl_2 (10 mL) and filtered through a short pad of silica gel,

which was washed with CH_2Cl_2 (50 mL). The filtrate was concentrated *in vacuo*, and the crude bromide **S2ab** was used for the next step without purification.

Under N_2, the crude bromide was redissolved in dry DCE (30 mL), to which were added diphenyl sulfide (2.79 g, 1.5 equiv, 15.0 mmol) and $AgBF_4$ (1.95 g, 1.0 equiv, 10.0 mmol). The mixture was stirred at 80 °C for 12 h before it was cooled to room temperature and filtered through a short pad of celite to remove the generated silver bromide. The celite was washed with CH_2Cl_2 (50 mL), and the filtrate was concentrated *in vacuo*. The residue was purified by flash column chromatography on silica gel to afford the desired sulfonium salt **S3ab** as a white solid (eluent: $CH_2Cl_2 \rightarrow$ EtOAc, 2.1 g, 50% yield over 2 steps).

^{1}H NMR (400 MHz, acetone-d_6) δ 8.39 (d, *J* = 8.0 Hz, 2H), 8.32 (d, *J* = 8.1 Hz, 2H), 7.98 – 7.84 (m, 6H), 7.41 (t, *J* = 7.7 Hz, 2H), 7.30 (t, *J* = 7.2 Hz, 1H), 7.01 (d, *J* = 7.8 Hz, 2H), 6.28 (q, *J* = 7.2 Hz, 1H), 1.98 (d, *J* = 7.1 Hz, 3H) ppm.

^{13}C NMR (101 MHz, acetone-d_6) δ 166.2, 150.9, 136.4, 136.1, 133.1, 132.64, 132.58, 131.9, 130.6, 127.8, 125.4, 123.5, 121.8, 57.6, 14.9 ppm.

HRMS (ES+) Calcd for $C_{21}H_{19}O_2S^+$ $[M-BF_4]^+$: 335.1101, Found: 335.1096.

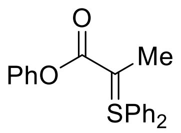

1ab

Phenyl 2-(diphenyl-λ⁴-sulfaneylidene)propanoate (1ab). Under N_2 at 0 °C, to an oven-dried 100-mL flask charged with the sulfonium salt **S3ab** (1.43 g, 1.0 equiv, 3.4 mmol) and dry THF (34.0 mL, 0.1 M) was added NaH (272 mg, 2.0 equiv, 60 wt% in mineral oil) portionwise. The resulting suspension was slowly warmed to room temperature and stirred for 7 h. Then, it was filtered through a short pad of celite, which was washed with CH_2Cl_2 (20 mL). The filtrate was concentrated *in vacuo* to give the pure sulfur ylide **1ab** as a yellow oil, which

was used directly without further purification (1.1 g, *s-cis/trans* = 2:1, 97% yield). (*Note: This sulfur ylide will deteriorate when exposed to the air for a long time. Therefore, a small amount mineral oil introduced by NaH was not completely removed, but it has no detrimental effect on the reaction.*)

Major isomer:

^{1}H NMR (400 MHz, $CDCl_3$) δ 7.54 – 7.47 (m, 10H), 7.33 – 7.28 (m, 2H), 7.17 – 7.06 (m, 3H), 1.79 (s, 3H) ppm.

^{13}C NMR (151 MHz, $CDCl_3$) δ 167.9, 153.4, 132.5, 131.2, 131.15, 131.12, 129.9, 129.2, 129.1, 129.0, 128.9, 123.7, 121.8, 41.7, 10.7 ppm.

HRMS (ES+) Calcd for $C_{21}H_{19}O_2S^+$ $[M-BF_4]^+$: 335.1101, Found: 335.1095.

III. Synthesis of Catalyst B4

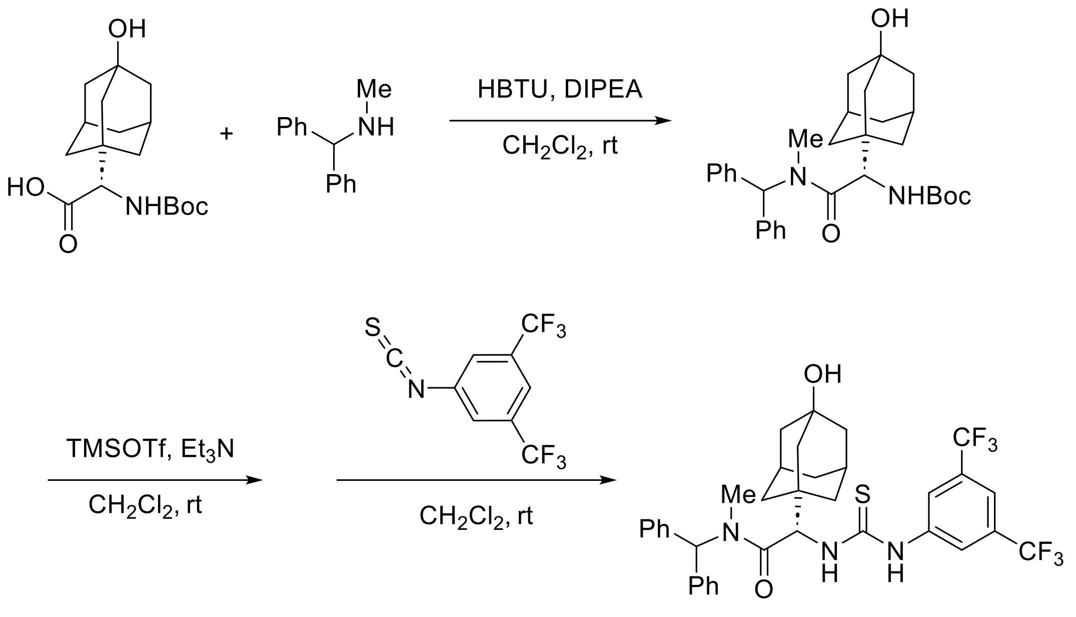

(2*S*)-*N*-Benzhydryl-2-(3-(3,5-bis(trifluoromethyl)phenyl)thioureido)-2-(3-hydroxyadamantan-1-yl)-*N*-methylacetamide (B4) was synthesized according to a reported procedure[2] from the commercially available Boc-3-hydroxy-1-adamantyl-D-glycine (3.25g, 1 equiv, 10 mmol). Purification by flash column chromatography on silica gel provided the catalyst **B4** as a white solid (eluent: *n*-hexane/EtOAc = 10:1 → 1:1, 2.1 g, 31% yield over 3 steps).

$[\alpha]_D^{25}$: -56.0 (c = 0.2, CH_2Cl_2).

Major conformational rotamer:

^{1}H NMR (400 MHz, DMSO-d_6) δ 10.5 (br, 1H), 8.38 (s, 2H), 8.28 (d, J = 8.8 Hz, 1H), 7.76 (br, 1H), 7.40 (t, J = 7.5 Hz, 2H), 7.36 – 7.24 (m, 4H), 7.17 (d, J = 7.2 Hz, 1H), 7.12 (d, J = 7.4 Hz, 2H), 6.99 (s, 1H), 5.34 (d, J = 8.8 Hz, 1H), 4.49 (s, 1H), 3.01 (s, 3H), 2.09 (br, 1H), 1.71 – 1.36 (m, 14H) ppm.

All observed signals for both isomers:

---

(2) Zuend, S. J.; Coughlin, M. P.; Lalonde, M. P.; Jacobsen, E. N. Scaleable Catalytic Asymmetric Strecker Syntheses of Unnatural α-Amino Acids. *Nature* **2009**, *461*, 968–970.

^{13}**C NMR** (101 MHz, DMSO-d_6) δ 180.5, 170.6, 141.7, 139.2, 138.5, 130.4, 130.1, 129.2, 128.6, 128.4, 127.7, 127.1, 124.6, 121.9, 121.6, 66.6, 60.6, 60.3, 46.1, 44.6, 44.3, 40.6, 37.6, 37.1, 35.1, 33.1, 29.74, 29.68 ppm.

^{19}**F NMR** (376 MHz, $CDCl_3$) δ -62.9 ppm.

HRMS (ES+) Calcd for $C_{35}H_{35}F_6N_3NaO_2S^+$ $[M+Na]^+$: 698.2247, Found: 698.2252.

IV. Synthesis of Chiral α-Oxygenated Ketones and Amides

Table S1. Condition Optimization for Sulfonium Ylide 1r[a]

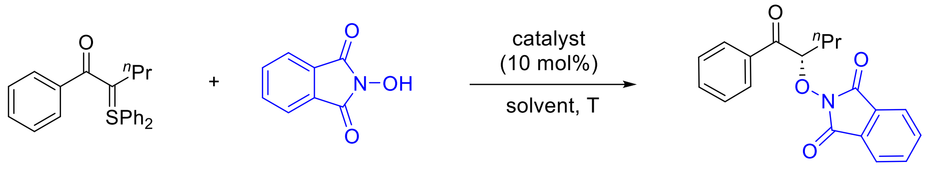

1r **2a** **3r**

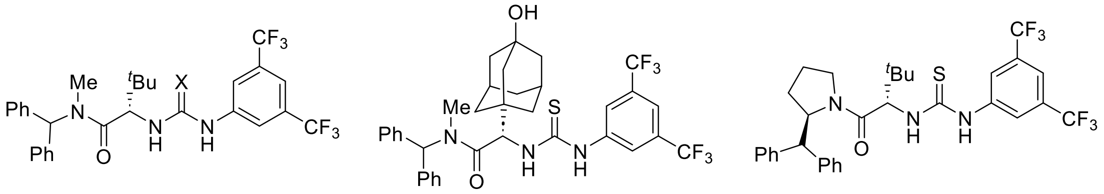

(*S*)-**B1**: X = S; (*S*)-**B2**: X = O (*S*)-**B4** (*S*,*R*)-**B5**

entry	catalyst	T/	t/h	solvent	Yield/%	ee /%[b]
1	(*S*)-**B1**	-10	24	*m*-xylene	>95	73
2	(*S*)-**B2**	-10	24	*m*-xylene	>95	50
3	(*S*)-**B4**	-10	24	*m*-xylene	>95	78
4	(*S*,*R*)-**B5**	-10	24	*m*-xylene	>95	56
5	(*S*)-**B4**	-20	48	*m*-xylene	>95	85
6	(*S*)-**B4**	-30	72	*m*-xylene	>95	85
7	(*S*)-**B4**	-40	72	*m*-xylene	<5%	—
8	(*S*)-**B4**	-20	48	mesitylene	>95	84
9	(*S*)-**B4**	-20	48	*o*-xylene	>95	84
10[c]	(*S*)-**B4**	-20	48	*m*-xylene + EtOAc	>95	72
11[c]	(*S*)-**B4**	-20	48	*m*-xylene + CH_2Cl_2	>95	48
12[c]	(*S*)-**B4**	-20	48	*m*-xylene + *n*-hexane	>95	86
13[c]	(*S*)-**B4**	-20	48	*m*-xylene + *c*-hexane	>95	87
14[d]	(*S*)-**B4**	-20	48	*m*-xylene + *c*-hexane	>95	88

[a]Unless noted, a mixture of **1r** (0.05 mmol), **2a** (0.06 mmol) and catalyst (10.0 mol%) in the solvent (0.5 mL) was stirred at corresponding temperature. NMR yields were determined by ^1H NMR with CH_2Br_2 as internal standard.

[b]Determined by chiral HPLC analysis. [c]Ratio of the two solvents is 1:1. [d]Solvent: *m*-xylene/*c*-hexane = 2:3, 1.0 mL.

General Procedure B (for Methyl Ketones).

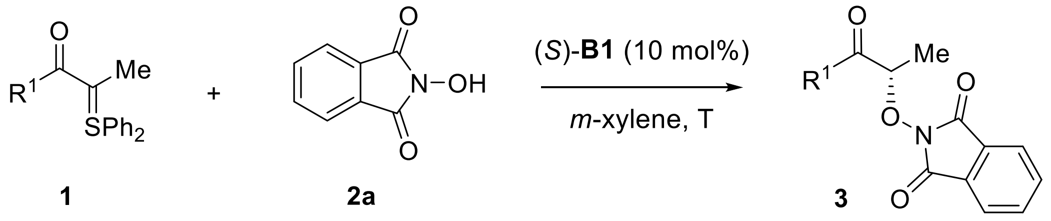

1 *s-cis* & *s-trans* mixture   **2a**   **3**

A 20-mL vial equipped with a magnetic stirring bar was charged with the sulfonium ylide **1** (0.4 mmol, 1.0 equiv) and the thiourea catalyst (*S*)-**B1** (23.3 mg, 10.0 mol%, 0.04 mmol). Next, *m*-xylene (4.0 mL) was added to the vial. The mixture was stirred at -15 °C (for **1n**, **1w**) or -30 °C (for others) for 5 min. *N*-Hydroxyphthalimide **2a** (78.2 mg, 1.2 equiv, 0.48 mmol) was then added to the reaction mixture. The mixture was stirred for 96 h at the same temperature. Upon completion, it was directly subjected to silica gel column chromatography to afford the desired product.

General Procedure C (for Ketones with Long Alkyl Chain).

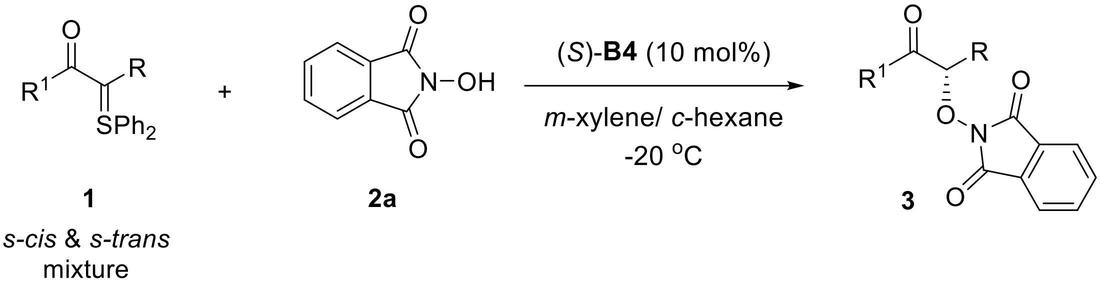

A 20-mL vial equipped with a magnetic stirring bar was charged with the sulfonium ylide **1** (0.4 mmol, 1.0 equiv) and the thiourea catalyst (*S*)-**B4** (27.0 mg, 10.0 mol%, 0.04 mmol). Next, mixed solvent (*m*-xylene/*c*-hexane = 2:3, 8.0 mL) was added to the vial. The mixture was stirred at -20 °C for 5 min. *N*-Hydroxyphthalimide **2a** (78.2 mg, 1.2 equiv, 0.48 mmol) was then added to the reaction mixture. The mixture was stirred for 72 h at the same temperature. Upon completion, it was directly subjected to silica gel column chromatography to afford the desired product.

General Procedure D (for Other Nucleophiles).

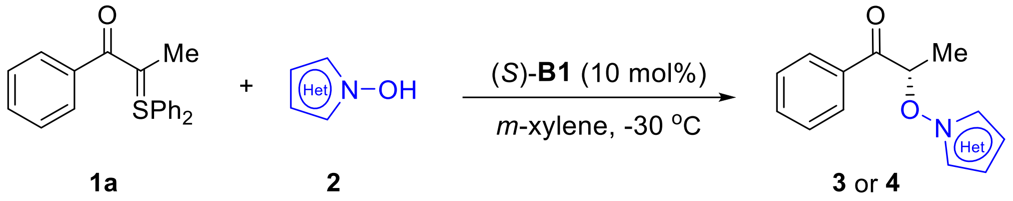

A 20-mL vial equipped with a magnetic stirring bar was charged with the sulfonium ylide **1a** (127.2 mg, 0.4 mmol, 1.0 equiv) and the thiourea catalyst (*S*)-**B1** (23.3 mg, 10.0 mol%, 0.04 mmol). Next, *m*-xylene (4.0 mL) was added to the vial. The mixture was stirred at -30 °C for 5 min. **2** (1.2 equiv, 0.48 mmol) was then added to the reaction mixture. The mixture was stirred for corresponding time at the same temperature. Upon completion, it was directly subjected to silica gel column chromatography to afford the desired product.

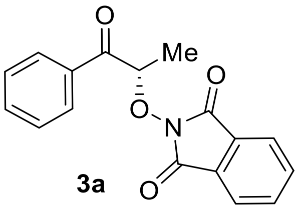

3a

(*S*)-2-((1-Oxo-1-phenylpropan-2-yl)oxy)isoindoline-1,3-dione (3a) was prepared as a white solid from **1a** (127.2 mg, 0.40 mmol) according to the General Procedure B (purified by flash column chrom1atography: *n*-hexane/CH_2Cl_2 = 5:1 → *n*-hexane/EtOAc = 5:1 → CH_2Cl_2) in 97% yield (114.4 mg, 93% ee).

$[\alpha]_D^{25}$: -84.1 (*c* = 0.2, CH_2Cl_2). HPLC analysis of the product: Daicel CHIRALPAK® IC-3 column; 30% *i*-PrOH in hexanes; 1.0 mL/min; retention times: 23.7 min (major), 33.8 min (minor).

^{1}H NMR (400 MHz, $CDCl_3$) δ 8.13 (d, *J* = 7.3 Hz, 2H), 7.80 (dd, *J* = 5.5, 3.1 Hz, 2H), 7.72 (dd, *J* = 5.5, 3.1 Hz, 2H), 7.57 (t, *J* = 7.4 Hz, 1H), 7.47 (t, *J* = 7.7 Hz, 2H), 5.73 (q, *J* = 6.7 Hz, 1H), 1.66 (d, *J* = 6.8 Hz, 3H) ppm.

^{13}C NMR (101 MHz, $CDCl_3$) δ 195.4, 163.7, 134.7 (2C), 133.8, 129.3, 128.8, 128.7, 123.7, 83.6, 16.3 ppm

HRMS (ES+) Calcd for $C_{17}H_{13}NNaO_4^+$ $[M+Na]^+$: 318.0737, Found: 318.0746.

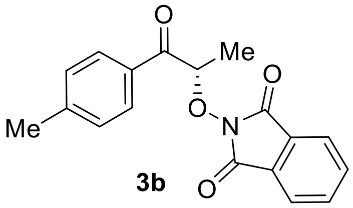

3b

(*S*)-2-((1-Oxo-1-(*p*-tolyl)propan-2-yl)oxy)isoindoline-1,3-dione (3b) was prepared as a white solid from **1b** (132.8 mg, 0.40 mmol) according to the General Procedure B (purified by flash column chromatography: *n*-hexane/CH_2Cl_2 = 5:1 → *n*-hexane/EtOAc = 5:1 → CH_2Cl_2) in 91% yield (112.5 mg, 92% ee).

$[\alpha]_D^{25}$: -110.5 (*c* = 0.2, CH_2Cl_2). HPLC analysis of the product: Daicel

CHIRALPAK® IC-3 column; 50% *i*-PrOH in hexanes; 1.0 mL/min; retention times: 20.0 min (major), 29.3 min (minor).

^{1}H NMR (400 MHz, $CDCl_3$) δ 8.05 (d, *J* = 8.1 Hz, 2H), 7.82 (dd, *J* = 5.5, 3.1 Hz, 2H), 7.74 (dd, *J* = 5.5, 3.1 Hz, 2H), 7.28 (d, *J* = 8.1 Hz, 2H), 5.72 (q, *J* = 6.7 Hz, 1H), 2.41 (s, 3H), 1.67 (d, *J* = 6.8 Hz, 3H) ppm.

^{13}C NMR (101 MHz, $CDCl_3$) δ 195.1, 163.8, 144.9, 134.7, 132.3, 129.5, 129.4, 128.9, 123.8, 83.7, 21.9, 16.4 ppm.

HRMS (ES+) Calcd for $C_{18}H_{15}NNaO_4^+$ $[M+Na]^+$: 332.0894, Found: 332.0902.

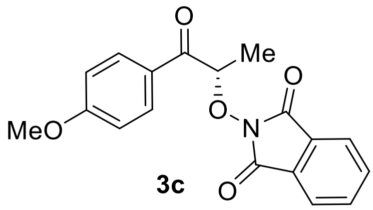

3c

(*S*)-2-((1-(4-Methoxyphenyl)-1-oxopropan-2-yl)oxy)isoindoline-1,3-dione (3c) was prepared as a white solid from **1c** (139.2 mg, 0.40 mmol) according to the General Procedure B (purified by flash column chromatography: *n*-hexane/CH_2Cl_2 = 5:1 → *n*-hexane/EtOAc = 5:1 → CH_2Cl_2) in 92% yield (119.9 mg, 93% ee).

$[\alpha]_D^{25}$: -81.3 (*c* = 0.2, CH_2Cl_2). HPLC analysis of the product: Daicel CHIRALPAK® IC-3 column; 50% *i*-PrOH in hexanes; 1.0 mL/min; retention times: 27.5 min (major), 39.1 min (minor).

^{1}H NMR (400 MHz, $CDCl_3$) δ 8.17 (d, *J* = 8.9 Hz, 2H), 7.82 (dd, *J* = 5.5, 3.1 Hz, 2H), 7.74 (dd, *J* = 5.5, 3.1 Hz, 2H), 6.96 (d, *J* = 8.9 Hz, 2H), 5.69 (q, *J* = 6.7 Hz, 1H), 3.87 (s, 3H), 1.66 (d, *J* = 6.7 Hz, 3H) ppm.

^{13}C NMR (101 MHz, $CDCl_3$) δ 193.8, 164.1, 163.8, 134.8, 131.8, 128.9, 127.8, 123.8, 114.0, 83.7, 55.6, 16.4 ppm.

HRMS (ES+) Calcd for $C_{18}H_{15}NNaO_5^+$ $[M+Na]^+$: 348.0843, Found: 348.0851.

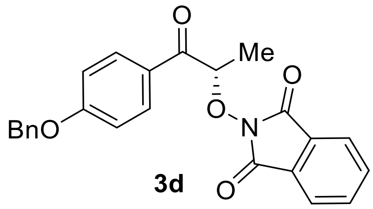

3d

(*S*)-2-((1-(4-(Benzyloxy)phenyl)-1-oxopropan-2-yl)oxy)isoindoline-1,3-dione (3d) was prepared as a white solid from **1d** (158.4 mg, 0.40 mmol) according to the General Procedure B (purified by flash column chromatography: *n*-hexane/CH_2Cl_2 = 5:1 → *n*-hexane/EtOAc = 5:1 → CH_2Cl_2) in 96% yield (154.0 mg, 92% ee).

$[\alpha]_D^{25}$: -84.1 (*c* = 0.2, CH_2Cl_2). HPLC analysis of the product: Daicel CHIRALPAK® IC-3 column; 50% *i*-PrOH in hexanes; 1.0 mL/min; retention times: 30.1 min (major), 43.2 min (minor).

^{1}H NMR (400 MHz, $CDCl_3$) δ 8.17 (d, *J* = 8.9 Hz, 2H), 7.83 (dd, *J* = 5.5, 3.1 Hz, 2H), 7.74 (dd, *J* = 5.5, 3.0 Hz, 2H), 7.46 – 7.31 (m, 5H), 7.04 (d, *J* = 9.0 Hz, 2H), 5.70 (q, *J* = 6.7 Hz, 1H), 5.14 (s, 2H), 1.67 (d, *J* = 6.7 Hz, 3H) ppm.

^{13}C NMR (101 MHz, $CDCl_3$) δ 193.8, 163.8, 163.3, 136.2, 134.8, 131.8, 128.9, 128.8, 128.4, 128.0, 127.6, 123.8, 114.8, 83.7, 70.3, 16.4 ppm.

HRMS (ES+) Calcd for $C_{24}H_{19}NNaO_5^+$ $[M+Na]^+$: 424.1156, Found: 424.1164.

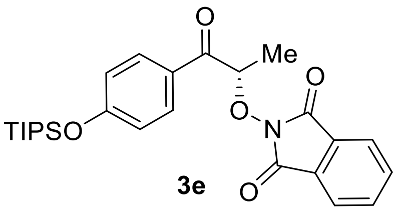

3e

(*S*)-2-((1-Oxo-1-(4-((triisopropylsilyl)oxy)phenyl)propan-2-yl)oxy)iso indoline-1,3-dione (3e) was prepared as a white wax from **1e** (196.0 mg, 0.40 mmol) according to the General Procedure B (purified by flash column chromatography: *n*-hexane/CH_2Cl_2 = 5:1 → 1:1) in 80% yield (149.9 mg, 89% ee).

$[\alpha]_D^{25}$: -52.0 (*c* = 0.2, CH_2Cl_2). HPLC analysis of the product: Daicel CHIRALPAK® IC-3 column; 30% *i*-PrOH in hexanes; 1.0 mL/min; retention

times: 16.8 min (major), 23.9 min (minor).

^{1}H NMR (400 MHz, $CDCl_3$) δ 8.09 (d, *J* = 8.8 Hz, 2H), 7.82 (dd, *J* = 5.5, 3.1 Hz, 2H), 7.74 (dd, *J* = 5.5, 3.1 Hz, 2H), 6.92 (d, *J* = 8.8 Hz, 2H), 5.72 (q, *J* = 6.7 Hz, 1H), 1.66 (d, *J* = 6.7 Hz, 3H), 1.35 – 1.22 (m, 3H), 1.09 (d, *J* = 7.4 Hz, 18H) ppm.

^{13}C NMR (101 MHz, $CDCl_3$) δ 193.6, 163.7, 161.4, 134.6, 131.6, 128.9, 128.0, 123.7, 119.9, 83.3, 17.9, 16.3, 12.7 ppm.

HRMS (ES+) Calcd for $C_{26}H_{33}NNaO_5Si^+$ $[M+Na]^+$: 490.2021, Found: 490.2025.

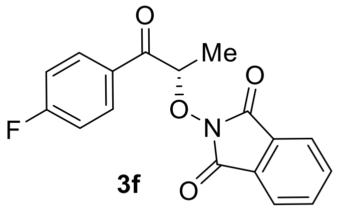

3f

(*S*)-2-((1-(4-Fluorophenyl)-1-oxopropan-2-yl)oxy)isoindoline-1,3-dione (3f) was prepared as a white solid from **1f** (134.4 mg, 0.40 mmol) according to the General Procedure B (purified by flash column chromatography: *n*-hexane/CH_2Cl_2 = 5:1 → *n*-hexane/EtOAc = 5:1 → CH_2Cl_2) in 93% yield (116.3 mg, 94% ee).

$[\alpha]_D^{25}$: -145.1 (*c* = 0.2, CH_2Cl_2). HPLC analysis of the product: Daicel CHIRALPAK® IC-3 column; 30% *i*-PrOH in hexanes; 1.0 mL/min; retention times: 18.2 min (major), 24.2 min (minor).

^{1}H NMR (400 MHz, $CDCl_3$) δ 8.26 (dd, *J* = 8.9, 5.4 Hz, 2H), 7.83 (dd, *J* = 5.4, 3.2 Hz, 2H), 7.76 (dd, *J* = 5.5, 3.1 Hz, 2H), 7.17 (t, *J* = 8.6 Hz, 2H), 5.64 (q, *J* = 6.7 Hz, 1H), 1.67 (d, *J* = 6.7 Hz, 3H) ppm.

^{13}C NMR (101 MHz, $CDCl_3$) δ 193.8, 166.2 (d, *J* = 254.4 Hz), 163.8, 134.9, 132.3 (d, *J* = 9.4 Hz), 131.2 (d, *J* = 2.9 Hz), 128.9, 123.9, 116.0 (d, *J* = 21.8 Hz), 84.1, 16.1 ppm.

^{19}F NMR (376 MHz, $CDCl_3$) δ -103.7 ppm.

HRMS (ES+) Calcd for $C_{17}H_{12}FNNaO_4^+$ $[M+Na]^+$: 336.0643, Found: 336.0651.

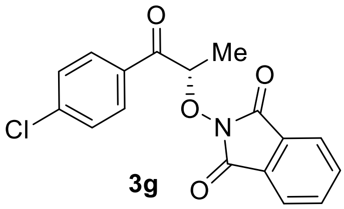

3g

(*S*)-2-((1-(4-Chlorophenyl)-1-oxopropan-2-yl)oxy)isoindoline-1,3-dione (3g) was prepared as a white solid from **1g** (141.2 mg, 0.40 mmol) according to the General Procedure B (purified by flash column chromatography: *n*-hexane/CH_2Cl_2 = 5:1 → *n*-hexane/EtOAc = 5:1 → CH_2Cl_2) in 88% yield (115.5 mg, 93% ee).

$[\alpha]_D^{25}$: -110.5 (c = 0.2, CH_2Cl_2). HPLC analysis of the product: Daicel CHIRALPAK® IC-3 column; 30% *i*-PrOH in hexanes; 1.0 mL/min; retention times: 17.9 min (major), 25.0 min (minor).

^{1}H NMR (400 MHz, $CDCl_3$) δ 8.15 (d, J = 8.6 Hz, 2H), 7.82 (dd, J = 5.4, 3.1 Hz, 2H), 7.75 (dd, J = 5.5, 3.1 Hz, 2H), 7.46 (d, J = 8.6 Hz, 2H), 5.62 (q, J = 6.7 Hz, 1H), 1.66 (d, J = 6.8 Hz, 3H) ppm.

^{13}C NMR (101 MHz, $CDCl_3$) δ 194.3, 163.8, 140.4, 134.9, 133.1, 130.9, 129.1, 128.8, 123.9, 84.1, 16.0 ppm

HRMS (ES+) Calcd for $C_{17}H_{12}ClNNaO_4^+$ $[M+Na]^+$: 352.0348, Found: 352.0352.

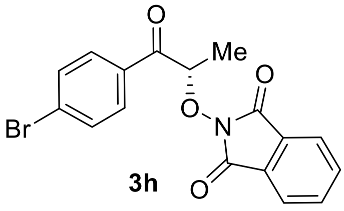

3h

(*S*)-2-((1-(4-Bromophenyl)-1-oxopropan-2-yl)oxy)isoindoline-1,3-dione (3h) was prepared as a white solid from **1h** (158.8 mg, 0.40 mmol) according to the General Procedure B (purified by flash column chromatography: *n*-hexane/CH_2Cl_2 = 5:1 → *n*-hexane/EtOAc = 5:1 → CH_2Cl_2) in 92% yield (138.1 mg, 94% ee).

$[\alpha]_D^{25}$: -98.8 (c = 0.2, CH_2Cl_2). HPLC analysis of the product: Daicel

CHIRALPAK® IC-3 column; 30% *i*-PrOH in hexanes; 1.0 mL/min; retention times: 18.8 min (major), 26.7 min (minor).

^{1}H NMR (400 MHz, $CDCl_3$) δ 8.06 (d, *J* = 8.6 Hz, 2H), 7.81 (dd, *J* = 5.5, 3.1 Hz, 2H), 7.74 (dd, *J* = 5.5, 3.1 Hz, 2H), 7.62 (d, *J* = 8.6 Hz, 2H), 5.61 (q, *J* = 6.7 Hz, 1H), 1.65 (d, *J* = 6.7 Hz, 3H) ppm.

^{13}C NMR (101 MHz, $CDCl_3$) δ 194.4, 163.7, 134.8, 133.5, 132.1, 131.0, 129.2, 128.8, 123.8, 84.0, 16.0 ppm.

HRMS (ES+) Calcd for $C_{17}H_{12}BrNNaO_4^+$ $[M+Na]^+$: 395.9842, Found: 395.9851.

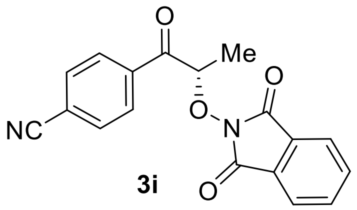

3i

(*S*)-4-(2-((1,3-Dioxoisoindolin-2-yl)oxy)propanoyl)benzonitrile (3i) was prepared as a yellow solid from **1i** (137.2 mg, 0.40 mmol) according to the General Procedure B (purified by flash column chromatography: *n*-hexane/CH_2Cl_2 = 5:1 → *n*-hexane/EtOAc = 5:1 → CH_2Cl_2/EtOAc = 5:1) in 87% yield (111.2 mg, 98% ee).

$[\alpha]_D^{25}$: -134.4 (*c* = 0.2, CH_2Cl_2). HPLC analysis of the product: Daicel CHIRALPAK® IC-3 column; 30% *i*-PrOH in hexanes; 1.0 mL/min; retention times: 43.3 min (major), 47.8 min (minor).

^{1}H NMR (400 MHz, $CDCl_3$) δ 8.34 (d, *J* = 8.5 Hz, 2H), 7.87 – 7.73 (m, 6H), 5.57 (q, *J* = 6.7 Hz, 1H), 1.67 (d, *J* = 6.7 Hz, 3H) ppm.

^{13}C NMR (101 MHz, $CDCl_3$) δ 194.3, 163.8, 137.9, 135.0, 132.5, 130.0, 128.7, 123.9, 118.0, 116.8, 84.5, 15.6 ppm.

HRMS (ES+) Calcd for $C_{18}H_{12}N_2NaO_4^+$ $[M+Na]^+$: 343.0690, Found: 343.0695.

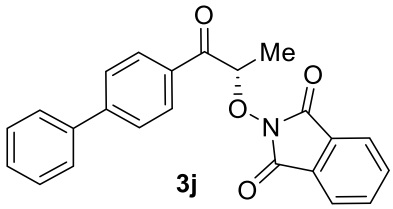

3j

(*S*)-4-(2-((1,3-Dioxoisoindolin-2-yl)oxy)propanoyl)benzonitrile (3j) was prepared as a white solid from **1j** (157.6 mg, 0.40 mmol) according to the General Procedure B (purified by flash column chromatography: *n*-hexane/CH_2Cl_2 = 5:1 → *n*-hexane/EtOAc = 5:1 → CH_2Cl_2) in 80% yield (119.1 mg, 97% ee).

$[\alpha]_D^{25}$: -67.1 (c = 0.2, CH_2Cl_2). HPLC analysis of the product: Daicel CHIRALPAK® IC-3 column; 50% *i*-PrOH in hexanes; 1.0 mL/min; retention times: 21.6 min (major), 33.2 min (minor).

^{1}H NMR (400 MHz, $CDCl_3$) δ 8.25 (d, J = 8.1 Hz, 2H), 7.88 – 7.58 (m, 8H), 7.51 – 7.36 (m, 3H), 5.77 (q, J = 6.7 Hz, 1H), 1.71 (d, J = 6.6 Hz, 3H) ppm.

^{13}C NMR (101 MHz, $CDCl_3$) δ 195.0, 163.8, 146.5, 139.8, 134.8, 133.5, 130.0, 129.1, 128.9, 128.5, 127.4 (2C), 123.8, 83.8, 16.3 ppm.

HRMS (ES+) Calcd for $C_{23}H_{17}NNaO_4^+$ $[M+Na]^+$: 394.1050, Found: 394.1060.

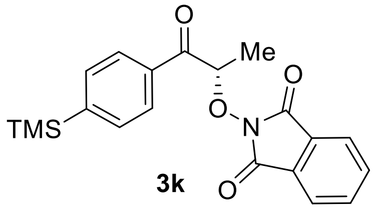

3k

(*S*)-2-((1-Oxo-1-(4-(trimethylsilyl)phenyl)propan-2-yl)oxy)isoindoline-1,3-dione (3k) was prepared as a yellow wax from **1k** (156.0 mg, 0.40 mmol) according to the General Procedure B (purified by flash column chromatography: *n*-hexane/CH_2Cl_2 = 5:1 → 1:1) in 88% yield (129.8 mg, 93% ee).

$[\alpha]_D^{25}$: -53.5 (c = 0.2, CH_2Cl_2). HPLC analysis of the product: Daicel CHIRALPAK® IC-3 column; 30% *i*-PrOH in hexanes; 1.0 mL/min; retention times: 16.7 min (major), 33.2 min (minor).

^{1}H NMR (400 MHz, $CDCl_3$) δ 8.11 (d, *J* = 8.1 Hz, 2H), 7.82 (dd, *J* = 5.5, 3.1 Hz, 2H), 7.74 (dd, *J* = 5.5, 3.1 Hz, 2H), 7.65 (d, *J* = 8.2 Hz, 2H), 5.75 (q, *J* = 6.7 Hz, 1H), 1.68 (d, *J* = 6.8 Hz, 3H), 0.28 (s, 9H) ppm.

^{13}C NMR (101 MHz, $CDCl_3$) δ 195.6, 163.8, 148.3, 134.9, 134.8, 133.7, 128.9, 128.1, 123.8, 83.7, 16.3, -1.3 ppm.

HRMS (ES+) Calcd for $C_{20}H_{21}NNaO_4Si^+$ $[M+Na]^+$: 390.1133, Found: 390.1139.

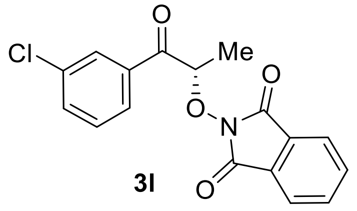

3l

(*S*)-2-((1-(3-Chlorophenyl)-1-oxopropan-2-yl)oxy)isoindoline-1,3-dione (3l) was prepared as a white solid from **1l** (141.2 mg, 0.40 mmol) according to the General Procedure B (purified by flash column chromatography: *n*-hexane/CH_2Cl_2 = 5:1 → *n*-hexane/EtOAc = 5:1 → CH_2Cl_2) in 87% yield (115.3 mg, 88% ee).

$[\alpha]_D^{25}$: -109.8 (*c* = 0.2, CH_2Cl_2). HPLC analysis of the product: Daicel CHIRALPAK® IC-3 column; 30% *i*-PrOH in hexanes; 1.0 mL/min; retention times: 15.4 min (major), 25.2 min (minor).

^{1}H NMR (400 MHz, $CDCl_3$) δ 8.18 (t, *J* = 1.8 Hz, 1H), 8.09 (d, *J* = 7.8 Hz, 1H), 7.83 (dd, *J* = 5.4, 3.1 Hz, 2H), 7.76 (dd, *J* = 5.5, 3.1 Hz, 2H), 7.59 – 7.54 (m, 1H), 7.45 (t, *J* = 7.9 Hz, 1H), 5.62 (q, *J* = 6.7 Hz, 1H), 1.68 (d, *J* = 6.8 Hz, 3H) ppm.

^{13}C NMR (101 MHz, $CDCl_3$) δ 194.3, 163.8, 136.3, 135.1, 134.9, 133.8, 130.1, 129.5, 128.9, 127.6, 123.9, 84.1, 16.0 ppm.

HRMS (ES+) Calcd for $C_{17}H_{12}ClNNaO_4^+$ $[M+Na]^+$: 352.0348, Found: 352.0353.

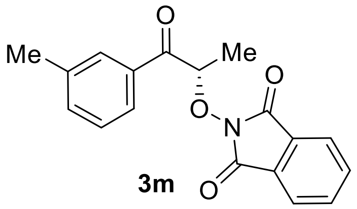

(*S*)-2-((1-Oxo-1-(*m*-tolyl)propan-2-yl)oxy)isoindoline-1,3-dione (3m) was prepared as a white solid from **1m** (132.8 mg, 0.40 mmol) according to the General Procedure B (purified by flash column chromatography: *n*-hexane/CH_2Cl_2 = 5:1 → *n*-hexane/EtOAc = 5:1 → CH_2Cl_2) in 97% yield (119.3 mg, 93% ee).

$[\alpha]_D^{25}$: -88.4 (c = 0.2, CH_2Cl_2). HPLC analysis of the product: Daicel CHIRALPAK® IC-3 column; 50% *i*-PrOH in hexanes; 1.0 mL/min; retention times: 16.1 min (major), 26.0 min (minor).

^{1}H NMR (400 MHz, $CDCl_3$) δ 7.95 – 7.92 (m, 2H), 7.82 (dd, J = 5.4, 3.1 Hz, 2H), 7.74 (dd, J = 5.5, 3.1 Hz, 2H), 7.43 – 7.34 (m, 2H), 5.75 (q, J = 6.8 Hz, 1H), 2.41 (s, 3H), 1.67 (d, J = 6.8 Hz, 3H) ppm.

^{13}C NMR (101 MHz, $CDCl_3$) δ 195.6, 163.8, 138.7, 134.8, 134.74, 134.69, 129.8, 128.9, 128.7, 126.5, 123.8, 83.6, 21.5, 16.4 ppm.

HRMS (ES+) Calcd for $C_{18}H_{15}NNaO_4^+$ $[M+Na]^+$: 332.0894, Found: 332.0897.

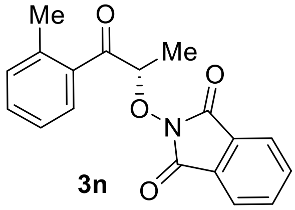

(*S*)-2-((1-Oxo-1-(*o*-tolyl)propan-2-yl)oxy)isoindoline-1,3-dione (3n) was prepared as a white solid from **1n** (132.8 mg, 0.40 mmol) according to the General Procedure B (reaction temperature = -15 °C, 72 h, purified by flash column chromatography: petroleum ether/CH_2Cl_2 = 5:1 → petroleum ether/EtOAc = 5:1 → CH_2Cl_2) in 77% yield (95.2 mg, 75% ee).

$[\alpha]_D^{25}$: -44.6 (c = 0.2, CH_2Cl_2). HPLC analysis of the product: Daicel

CHIRALPAK® IC-3 column; 50% *i*-PrOH in hexanes; 1.0 mL/min; retention times: 9.0 min (major), 10.6 min (minor).

^{1}H NMR (400 MHz, $CDCl_3$) δ 7.86 (d, *J* = 7.6 Hz, 1H), 7.82 – 7.78 (m, 2H), 7.76 – 7.71 (m, 2H), 7.38 (t, *J* = 7.6 Hz, 1H), 7.26 (t, *J* = 7.7 Hz, 2H), 5.68 (q, *J* = 6.8 Hz, 1H), 2.50 (s, 3H), 1.61 (d, *J* = 6.8 Hz, 3H) ppm.

^{13}C NMR (101 MHz, $CDCl_3$) δ 199.0, 163.7, 139.3, 135.3, 134.7, 132.01, 131.98, 129.2, 128.8,125.6, 123.7, 83.3, 21.1, 16.0 ppm.

HRMS (ES+) Calcd for $C_{18}H_{15}NNaO_4^+$ $[M+Na]^+$: 332.0894, Found: 332.0888.

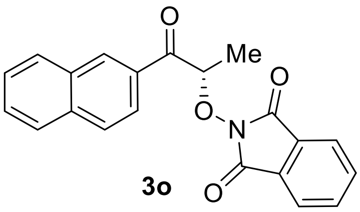

3o

(*S*)-2-((1-(Naphthalen-2-yl)-1-oxopropan-2-yl)oxy)isoindoline-1,3-dione (3o) was prepared as a white solid from **1o** (147.2 mg, 0.40 mmol) according to the General Procedure B (purified by flash column chromatography: *n*-hexane/CH_2Cl_2 = 5:1 → *n*-hexane/EtOAc = 5:1 → CH_2Cl_2) in 89% yield (122.9 mg, 92% ee).

$[\alpha]_D^{25}$: -69.4 (*c* = 0.2, CH_2Cl_2). HPLC analysis of the product: Daicel CHIRALPAK® IC-3 column; 50% *i*-PrOH in hexanes; 1.0 mL/min; retention times: 18.7 min (major), 30.4 min (minor).

^{1}H NMR (400 MHz, $CDCl_3$) δ 8.85 (s, 1H), 8.14 (dd, *J* = 8.6, 1.6 Hz, 1H), 8.02 (d, *J* = 8.1 Hz, 1H), 7.92 – 7.79 (m, 4H), 7.77 – 7.71 (m, 2H), 7.64 – 7.53 (m, 2H), 5.89 (q, *J* = 6.7 Hz, 1H), 1.75 (d, *J* = 6.7 Hz, 3H) ppm.

^{13}C NMR (101 MHz, $CDCl_3$) δ 195.1, 163.8, 135.9, 134.7, 132.5, 132.0, 131.7, 129.9, 128.9, 128.8, 128.6, 127.8, 126.9, 124.4, 123.7, 83.7, 16.2 ppm.

HRMS (ES+) Calcd for $C_{21}H_{15}NNaO_4^+$ $[M+Na]^+$: 368.0894, Found: 368.0899.

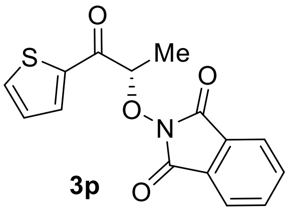

3p

(*S*)-2-((1-Oxo-1-(thiophen-2-yl)propan-2-yl)oxy)isoindoline-1,3-dione (3p) was prepared as a white solid from **1p** (129.6 mg, 0.40 mmol) according to the General Procedure B (purified by flash column chromatography: *n*-hexane/CH_2Cl_2 = 5:1 → *n*-hexane/EtOAc = 5:1 → CH_2Cl_2) in 95% yield (114.5 mg, 94% ee).

$[\alpha]_D^{25}$: -134.3 (*c* = 0.2, CH_2Cl_2). HPLC analysis of the product: Daicel CHIRALPAK® IC-3 column; 30% *i*-PrOH in hexanes; 1.0 mL/min; retention times: 30.7 min (major), 35.2 min (minor).

^{1}H NMR (400 MHz, $CDCl_3$) δ 8.22 (dd, *J* = 3.9, 1.1 Hz, 1H), 7.83 (dd, *J* = 5.5, 3.1 Hz, 2H), 7.75 (dd, *J* = 5.5, 3.1 Hz, 2H), 7.72 (d, *J* = 4.9 Hz, 1H), 7.21 – 7.16 (m, 1H), 5.48 (q, *J* = 6.7 Hz, 1H), 1.71 (s, 3H) ppm.

^{13}C NMR (101 MHz, $CDCl_3$) δ 188.4, 163.6, 141.3, 135.1, 134.7, 134.5, 128.8, 128.5, 123.8, 85.1, 16.6 ppm.

HRMS (ES+) Calcd for $C_{15}H_{11}NNaO_4S^+$ $[M+Na]^+$: 324.0301, Found: 324.0306.

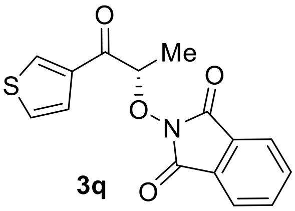

3q

(*S*)-2-((1-Oxo-1-(thiophen-3-yl)propan-2-yl)oxy)isoindoline-1,3-dione (3q) was prepared as a white solid from **1q** (129.6 mg, 0.40 mmol) according to the General Procedure B (purified by flash column chromatography: *n*-hexane/CH_2Cl_2 = 5:1 → *n*-hexane/EtOAc = 5:1 → CH_2Cl_2) in 94% yield (113.3 mg, 92% ee).

$[\alpha]_D^{25}$: -132.5 (*c* = 0.2, CH_2Cl_2). HPLC analysis of the product: Daicel

CHIRALPAK® IC-3 column; 30% *i*-PrOH in hexanes; 1.0 mL/min; retention times: 25.7 min (major), 30.3 min (minor).

^{1}H NMR (400 MHz, $CDCl_3$) δ 8.64 (d, *J* = 1.9 Hz, 1H), 7.83 (dd, *J* = 5.5, 3.1 Hz, 2H), 7.76 (dd, *J* = 5.5, 3.1 Hz, 2H), 7.72 (d, *J* = 5.1 Hz, 1H), 7.33 (dd, *J* = 5.1, 2.9 Hz, 1H), 5.45 (q, *J* = 6.7 Hz, 1H), 1.66 (d, *J* = 6.7 Hz, 3H) ppm.

^{13}C NMR (101 MHz, $CDCl_3$) δ 189.6, 163.8, 139.1, 135.2, 134.8, 128.9, 127.9, 126.2, 123.9, 85.5, 16.3 ppm.

HRMS (ES+) Calcd for $C_{15}H_{11}NNaO_4S^+$ [M+Na]$^+$: 324.0301, Found: 324.0305.

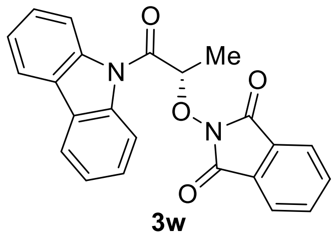

3w

(*S*)-2-((1-(9*H*-Carbazol-9-yl)-1-oxopropan-2-yl)oxy)isoindoline-1,3-dione (3w) was prepared as a white solid from **1w** (162.8 mg, 0.40 mmol) according to the General Procedure B (reaction temperature = -15°C, purified by flash column chromatography: *n*-hexane/CH_2Cl_2 = 5:1 → *n*-hexane/EtOAc = 5:1 → CH_2Cl_2) in 90% yield (138.7 mg, 88% ee).

$[\alpha]_D^{25}$: -59.8 (*c* = 0.2, CH_2Cl_2). HPLC analysis of the product: Daicel CHIRALPAK® IC-3 column; 50% *i*-PrOH in hexanes; 1.0 mL/min; retention times: 19.4 min (major), 34.5 min (minor).

^{1}H NMR (400 MHz, $CDCl_3$) δ 8.30 (d, *J* = 8.4 Hz, 2H), 8.02 – 7.93 (m, 2H), 7.80 (dd, *J* = 5.5, 3.1 Hz, 2H), 7.71 (dd, *J* = 5.5, 3.1 Hz, 2H), 7.50 (t, *J* = 7.2 Hz, 2H), 7.45 – 7.36 (m, 2H), 6.06 (q, *J* = 6.6 Hz, 1H), 1.85 (d, *J* = 6.6 Hz, 3H) ppm.

^{13}C NMR (101 MHz, $CDCl_3$) δ 168.9, 163.8, 138.3, 134.7, 128.8, 127.7, 126.7, 124.3, 123.8, 119.9, 116.5, 80.4, 16.2 ppm.

HRMS (ES+) Calcd for $C_{23}H_{16}N_2NaO_4^+$ [M+Na]$^+$: 407.1003, Found: 407.1006.

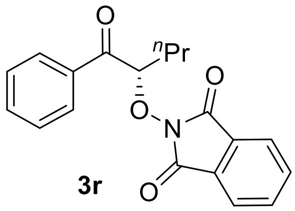

3r

(*S*)-2-((1-Oxo-1-phenylpentan-2-yl)oxy)isoindoline-1,3-dione (3r) was prepared as a yellow oil from **1r** (139.2 mg, 0.40 mmol) according to the General Procedure C (purified by flash column chromatography: *n*-hexane/CH_2Cl_2 = 5:1 → CH_2Cl_2) in 97% yield (125.6 mg, 88% ee).

$[\alpha]_D^{25}$: -80.8 (c = 0.2, CH_2Cl_2). HPLC analysis of the product: Daicel CHIRALPAK® IC-3 column; 20% *i*-PrOH in hexanes; 1.0 mL/min; retention times: 24.5 min (major), 27.7 min (minor).

^{1}H NMR (400 MHz, $CDCl_3$) δ 8.06 (d, *J* = 7.7 Hz, 2H), 7.88 – 7.65 (m, 4H), 7.58 (t, *J* = 7.4 Hz, 1H), 7.47 (t, *J* = 7.6 Hz, 2H), 5.58 (t, *J* = 6.5 Hz, 1H), 2.11 – 1.94 (m, 2H), 1.67 – 1.57 (m, 2H), 0.98 (t, *J* = 7.4 Hz, 3H) ppm.

^{13}C NMR (101 MHz, $CDCl_3$) δ 196.1, 163.5, 135.1, 134.7, 133.8, 129.0, 128.9, 128.8, 123.7, 88.2, 33.6, 18.7, 13.9 ppm.

HRMS (ES+) Calcd for $C_{19}H_{17}NNaO_4^+$ $[M+Na]^+$: 346.1050, Found: 346.1056.

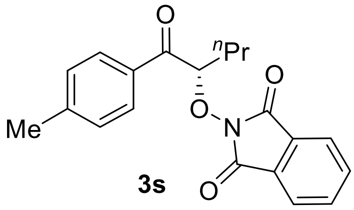

3s

(*S*)-2-((1-Oxo-1-(*p*-tolyl)pentan-2-yl)oxy)isoindoline-1,3-dione (3s) was prepared as a yellow oil from **1s** (144.0 mg, 0.40 mmol) according to the General Procedure C (purified by flash column chromatography: *n*-hexane/CH_2Cl_2 = 5:1 → CH_2Cl_2) in 85% yield (114.3 mg, 89% ee).

$[\alpha]_D^{25}$: -60.1 (c = 0.2, CH_2Cl_2). HPLC analysis of the product: Daicel CHIRALPAK® IC-3 column; 30% *i*-PrOH in hexanes; 1.0 mL/min; retention times: 21.6 min (major), 25.7 min (minor).

^{1}**H NMR** (400 MHz, $CDCl_3$) δ 7.96 (d, *J* = 8.1 Hz, 2H), 7.77 (dd, *J* = 5.5, 3.1 Hz, 2H), 7.70 (dd, *J* = 5.5, 3.1 Hz, 2H), 7.26 (d, *J* = 8.1 Hz, 2H), 5.55 (dd, *J* = 7.4, 5.6 Hz, 1H), 2.39 (s, 3H), 2.11 – 1.91 (m, 2H), 1.60 (h, *J* = 7.4 Hz, 2H), 0.98 (t, *J* = 7.4 Hz, 3H) ppm.

^{13}**C NMR** (101 MHz, $CDCl_3$) δ 195.7, 163.5, 144.7, 134.6, 132.6, 129.5, 129.1, 128.9, 123.7, 88.2, 33.7, 21.8, 18.7, 13.9 ppm.

HRMS (ES+) Calcd for $C_{20}H_{19}NNaO_4^+$ $[M+Na]^+$: 360.1207, Found: 360.1212.

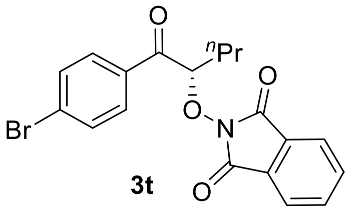

3t

(*S*)-2-((1-(4-Bromophenyl)-1-oxopentan-2-yl)oxy)isoindoline-1,3-dione (3t) was prepared as a pale-yellow solid from **1t** (169.6 mg, 0.40 mmol) according to the General Procedure C (purified by flash column chromatography: *n*-hexane/CH_2Cl_2 = 5:1 → CH_2Cl_2) in 91% yield (145.7 mg, 90% ee).

$[\alpha]_D^{25}$: -88.3 (*c* = 0.2, CH_2Cl_2). HPLC analysis of the product: Daicel CHIRALPAK® IC-3 column; 30% *i*-PrOH in hexanes; 1.0 mL/min; retention times: 12.7 min (major), 14.4 min (minor).

^{1}**H NMR** (400 MHz, $CDCl_3$) δ 7.98 (d, *J* = 8.6 Hz, 2H), 7.82 – 7.76 (m, 2H), 7.76 – 7.70 (m, 2H), 7.62 (d, *J* = 8.6 Hz, 2H), 5.39 (dd, *J* = 7.4, 5.9 Hz, 1H), 2.13 – 1.85 (m, 2H), 1.66 – 1.52 (m, 2H), 0.98 (t, *J* = 7.3 Hz, 3H) ppm.

^{13}**C NMR** (101 MHz, $CDCl_3$) δ 195.3, 163.5, 134.8, 133.7, 132.1, 130.8, 129.1, 128.8, 123.8, 89.2, 33.6, 18.7, 13.9 ppm.

HRMS (ES+) Calcd for $C_{19}H_{16}BrNNaO_4^+$ $[M+Na]^+$: 424.0155, Found: 424.0161.

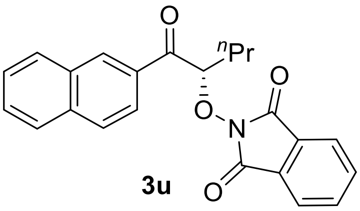

3u

(*S*)-2-((1-(Naphthalen-2-yl)-1-oxopentan-2-yl)oxy)isoindoline-1,3-dione (3u) was prepared as a pale-yellow solid from **1u** (158.4 mg, 0.40 mmol) according to the General Procedure C (purified by flash column chromatography: *n*-hexane/CH_2Cl_2 = 5:1 → CH_2Cl_2) in 86% yield (128.5 mg, 83% ee).

$[\alpha]_D^{25}$: -27.3 (*c* = 0.2, CH_2Cl_2). HPLC analysis of the product: Daicel CHIRALPAK® IC-3 column; 30% *i*-PrOH in hexanes; 1.0 mL/min; retention times: 21.3 min (major), 27.4 min (minor).

^{1}H NMR (400 MHz, $CDCl_3$) δ 8.68 (s, 1H), 8.10 (dd, *J* = 8.7, 1.8 Hz, 1H), 7.99 (d, *J* = 8.0 Hz, 1H), 7.90 (d, *J* = 8.7 Hz, 1H), 7.86 (d, *J* = 8.1 Hz, 1H), 7.78 (dd, *J* = 5.5, 3.1 Hz, 2H), 7.70 (dd, *J* = 5.5, 3.1 Hz, 2H), 7.63 – 7.51 (m, 2H), 5.74 – 5.59 (m, 1H), 2.28 – 1.96 (m, 2H), 1.66 (h, *J* = 7.4 Hz, 2H), 1.01 (t, *J* = 7.4 Hz, 3H) ppm.

^{13}C NMR (101 MHz, $CDCl_3$) δ 196.0, 163.6, 135.9, 134.7, 132.5, 132.4, 131.2, 129.9, 129.0, 128.9, 128.7, 127.9, 127.0, 124.4, 123.7, 88.4, 33.7, 18.8, 14.0 ppm.

HRMS (ES+) Calcd for $C_{23}H_{19}NNaO_4^+$ $[M+Na]^+$: 396.1207, Found: 396.1211.

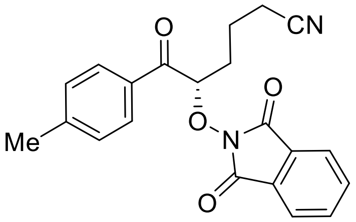

3v

(*S*)-5-((1,3-Dioxoisoindolin-2-yl)oxy)-6-oxo-6-(*p*-tolyl)hexanenitrile (3v) was prepared as a yellow wax from **1v** (154.0 mg, 0.40 mmol) according to the General Procedure C (purified by flash column chromatography: *n*-hexane/CH_2Cl_2 = 5:1 → CH_2Cl_2) in 95% yield (137.2 mg, 88% ee).

$[\alpha]_D^{25}$: -83.7 (*c* = 0.2, CH_2Cl_2). HPLC analysis of the product: Daicel

CHIRALPAK® IC-3 column; 30% *i*-PrOH in hexanes; 1.0 mL/min; retention times: 45.1 min (major), 56.7 min (minor).

^{1}H NMR (400 MHz, $CDCl_3$) δ 7.94 (d, *J* = 8.3 Hz, 2H), 7.78 (dd, *J* = 5.5, 3.1 Hz, 2H), 7.72 (dd, *J* = 5.6, 3.1 Hz, 2H), 7.26 (d, *J* = 8.2 Hz, 2H), 5.60 (dd, *J* = 7.4, 4.8 Hz, 1H), 2.65 – 2.46 (m, 2H), 2.39 (s, 3H), 2.27 – 1.93 (m, 4H) ppm.

^{13}C NMR (101 MHz, $CDCl_3$) δ 194.5, 163.5, 145.2, 134.7, 132.1, 129.6, 129.1, 128.7, 123.7, 119.2, 86.6, 30.0, 21.8, 21.1, 16.8 ppm.

HRMS (ES+) Calcd for $C_{21}H_{18}N_2NaO_4^+$ $[M+Na]^+$: 385.1159, Found: 385.1166.

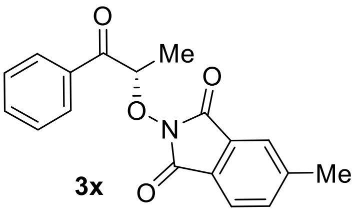

3x

(*S*)-5-Methyl-2-((1-oxo-1-phenylpropan-2-yl)oxy)isoindoline-1,3-dione (3x) was prepared as a white solid from **1a** with 4-methyl-*N*-hydroxyphthalimide (85.0 mg, 1.2 equiv, 0.48 mmol) according to the General Procedure D (96h, purified by flash column chromatography: *n*-hexane/CH_2Cl_2 = 5:1 → CH_2Cl_2) in 82% yield (101.4 mg, 93% ee).

$[\alpha]_D^{25}$: -82.6 (*c* = 0.2, CH_2Cl_2). HPLC analysis of the product: Daicel CHIRALPAK® IC-3 column; 50% *i*-PrOH in hexanes; 1.0 mL/min; retention times: 22.3 min (major), 32.5 min (minor).

^{1}H NMR (400 MHz, $CDCl_3$) δ 8.16 (d, *J* = 7.2 Hz, 2H), 7.69 (d, *J* = 7.6 Hz, 1H), 7.62 – 7.56 (m, 2H), 7.53 – 7.46 (m, 3H), 5.72 (q, *J* = 6.8 Hz, 1H), 2.49 (s, 3H), 1.68 (d, *J* = 6.8 Hz, 3H) ppm.

^{13}C NMR (101 MHz, $CDCl_3$) δ 195.5, 164.0, 164.0, 146.2, 135.2, 134.9, 133.8, 129.4, 129.2, 128.8, 126.2, 124.3, 123.8, 83.7, 22.2, 16.3 ppm.

HRMS (ES+) Calcd for $C_{18}H_{15}NNaO_4^+$ $[M+Na]^+$: 332.0894, Found: 332.0899.

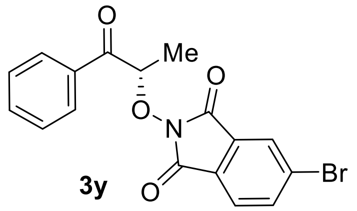

3y

(*S*)-5-Bromo-2-((1-oxo-1-phenylpropan-2-yl)oxy)isoindoline-1,3-dione (3y) was prepared as a white solid from **1a** with 4-bromo-*N*-hydroxyphthalimide (115.7 mg, 1.2 equiv, 0.48 mmol) according to the General Procedure D (96h, purified by flash column chromatography: *n*-hexane/CH_2Cl_2 = 5:1 → CH_2Cl_2) in 97% yield (145.0 mg, 94% ee).

$[\alpha]_D^{25}$: -78.7 (*c* = 0.2, CH_2Cl_2). HPLC analysis of the product: Daicel CHIRALPAK® IC-3 column; 30% *i*-PrOH in hexanes; 1.0 mL/min; retention times: 30.6 min (major), 40.9 min (minor).

^{1}H NMR (400 MHz, $CDCl_3$) δ 8.11 (d, *J* = 7.2 Hz, 2H), 7.94 (d, *J* = 1.5 Hz, 1H), 7.87 (dd, *J* = 8.0, 1.7 Hz, 1H), 7.68 (d, *J* = 8.0 Hz, 1H), 7.59 (t, *J* = 7.4 Hz, 1H), 7.48 (t, *J* = 7.9 Hz, 2H), 5.74 (q, *J* = 6.8 Hz, 1H), 1.67 (d, *J* = 6.8 Hz, 3H) ppm.

^{13}C NMR (101 MHz, $CDCl_3$) δ 195.3, 163.0, 162.4, 137.8, 134.7, 133.9, 130.4, 129.7, 129.2, 128.8, 127.3, 127.1, 125.2, 83.7, 16.4 ppm.

HRMS (ES+) Calcd for $C_{17}H_{12}BrNNaO_4^+$ $[M+Na]^+$: 395.9842, Found: 395.9847.

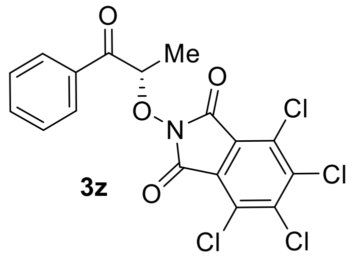

3z

(*S*)-4,5,6,7-Tetrachloro-2-((1-oxo-1-phenylpropan-2-yl)oxy)isoindoline-1,3-dione (3z) was prepared as a white solid from **1a** with 4,5,6,7-tetrachloro-*N*-hydroxyphthalimide (144.5 mg, 1.2 equiv, 0.48 mmol) according to the General Procedure D (96h, purified by flash column chromatography: *n*-hexane/CH_2Cl_2 = 5:1 → CH_2Cl_2) in 86% yield (148.5 mg, 93% ee).

$[\alpha]_D^{25}$: -89.2 (*c* = 0.2, CH_2Cl_2). HPLC analysis of the product: Daicel

CHIRALPAK® IC-3 column; 50% *i*-PrOH in hexanes; 1.0 mL/min; retention times: 32.3 min (major), 44.6 min (minor).

^{1}H NMR (400 MHz, $CDCl_3$) δ 8.12 (d, *J* = 7.1 Hz, 2H), 7.68 – 7.57 (m, 1H), 7.50 (t, *J* = 7.7 Hz, 2H), 5.74 (q, *J* = 6.8 Hz, 1H), 1.68 (d, *J* = 6.8 Hz, 3H) ppm.

^{13}C NMR (101 MHz, $CDCl_3$) δ 194.9, 159.3, 141.0, 134.6, 134.1, 130.3, 129.3, 128.9, 124.6, 84.1, 16.4 ppm.

HRMS (ES+) Calcd for $C_{17}H_9Cl_4NNaO_4^+$ [M+Na]$^+$: 455.9149, Found: 455.9156.

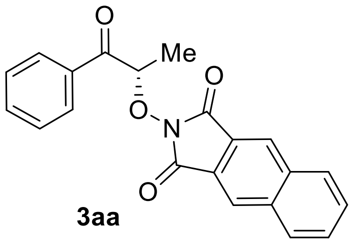

3aa

(*S*)-2-((1-Oxo-1-phenylpropan-2-yl)oxy)-1*H*-benzo[*f*]isoindole-1,3(2*H*)-dione (3aa) was prepared as a white solid from **1a** with *N*-hydroxy-1*H*-benzo[*f*]isoindole-1,3(2*H*)-dione (102.2 mg, 1.2 equiv, 0.48 mmol) according to the General Procedure D (96h, purified by flash column chromatography: *n*-hexane/CH_2Cl_2 = 5:1 → *n*-hexane/EtOAc = 5:1 → CH_2Cl_2) in 97% yield (133.2 mg, 91% ee).

$[\alpha]_D^{25}$: -122.6 (*c* = 0.2, CH_2Cl_2). HPLC analysis of the product: Daicel CHIRALPAK® IC-3 column; 70% *i*-PrOH in hexanes; 1.0 mL/min; retention times: 26.0 min (major), 43.4 min (minor).

^{1}H NMR (400 MHz, $CDCl_3$) δ 8.28 (s, 2H), 8.18 (d, *J* = 7.7 Hz, 2H), 8.02 (dd, *J* = 6.1, 2.8 Hz, 2H), 7.71 – 7.68 (m, 2H), 7.59 (t, *J* = 7.4 Hz, 1H), 7.49 (t, *J* = 7.6 Hz, 2H), 5.82 (q, *J* = 6.7 Hz, 1H), 1.72 (d, *J* = 6.7 Hz, 3H) ppm.

^{13}C NMR (101 MHz, $CDCl_3$) δ 195.5, 163.4, 135.6, 134.9, 133.9, 130.5, 129.7, 129.4, 128.8, 125.6, 124.5, 83.8, 16.4 ppm.

HRMS (ES+) Calcd for $C_{21}H_{15}NNaO_4^+$ [M+Na]$^+$: 368.0894, Found: 368.0898.

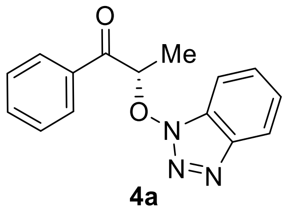

4a

(*S*)-2-((1*H*-Benzo[*d*][1,2,3]triazol-1-yl)oxy)-1-phenylpropan-1-one (4a) was prepared as a white solid from **1a** with 1-hydroxybenzotriazole (HOBt, 64.8 mg, 1.2 equiv, 0.48 mmol) according to the General Procedure D (72h, purified by flash column chromatography: *n*-hexane/CH_2Cl_2 = 5:1 → CH_2Cl_2) in 84% yield (90.1 mg, 84% ee).

$[\alpha]_D^{25}$: +22.9 (*c* = 0.2, CH_2Cl_2). HPLC analysis of the product: Daicel CHIRALPAK® IC-3 column; 30% *i*-PrOH in hexanes; 1.0 mL/min; retention times: 11.3 min (minor), 13.7 min (major).

^{1}H NMR (400 MHz, $CDCl_3$) δ 8.02 – 7.91 (m, 3H), 7.82 (d, *J* = 8.4 Hz, 1H), 7.65 – 7.56 (m, 1H), 7.54– 7.50 (m, 1H), 7.47 (t, *J* = 7.7 Hz, 2H), 7.39 – 7.31 (m, 1H), 6.37 (q, *J* = 6.9 Hz, 1H), 1.79 (d, *J* = 6.9 Hz, 3H) ppm.

^{13}C NMR (101 MHz, $CDCl_3$) δ 195.6, 143.4, 134.4, 134.2, 129.1, 128.8, 128.4, 128.3, 124.9, 120.0, 110.3, 85.5, 17.2 ppm.

HRMS (ES+) Calcd for $C_{15}H_{13}N_3NaO_2^+$ $[M+Na]^+$: 290.0900, Found: 290.0906.

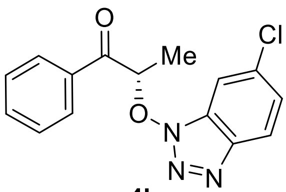

4b

(*S*)-2-((6-Chloro-1*H*-benzo[*d*][1,2,3]triazol-1-yl)oxy)-1-phenylpropan-1-one (4b) was prepared as a white solid from **1a** with 6-chloro1-hydroxybenzotriazole (81.4 mg, 1.2 equiv, 0.48 mmol) according to the General Procedure D (72 h, purified by flash column chromatography: *n*-hexane/CH_2Cl_2 = 5:1 → CH_2Cl_2) in 93% yield (111.8 mg, 80% ee).

$[\alpha]_D^{25}$: +162.6 (*c* = 0.2, CH_2Cl_2). HPLC analysis of the product: Daicel

CHIRALPAK® IC-3 column; 20% *i*-PrOH in hexanes; 1.0 mL/min; retention times: 10.2 min (minor), 12.9 min (major).

^{1}H NMR (400 MHz, $CDCl_3$) δ 7.91 (d, *J* = 7.3 Hz, 2H), 7.85 (d, *J* = 1.7 Hz, 1H), 7.82 (d, *J* = 8.9 Hz, 1H), 7.56 (t, *J* = 7.4 Hz, 1H), 7.43 (t, *J* = 7.6 Hz, 2H), 7.27 (dd, *J* = 8.8, 1.8 Hz, 1H), 6.37 (q, *J* = 7.0 Hz, 1H), 1.77 (d, *J* = 7.0 Hz, 3H) ppm.

^{13}C NMR (101 MHz, $CDCl_3$) δ 195.4, 141.8, 134.8, 134.3, 133.9, 129.0, 128.7, 128.6, 126.1, 120.8, 110.2, 85.6, 17.1 ppm.

HRMS (ES+) Calcd for $C_{15}H_{12}ClN_3NaO_2^+$ [M+Na]$^+$: 324.0511, Found: 324.0516.

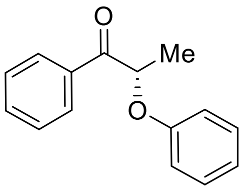

4c

(*S*)-2-Phenoxy-1-phenylpropan-1-one (4c) was prepared as a colorless oil from **1a** with phenol (45.2 mg, 1.2 equiv, 0.48 mmol) according to the General Procedure D (room temperature, 48 h, purified by flash column chromatography: *n*-hexane/EtOAc = 20:1) in 87% yield (78.6 mg, 55% ee).

$[\alpha]_D^{25}$: -11.7 (*c* = 0.1, CH_2Cl_2). HPLC analysis of the product: Daicel CHIRALPAK® IG-3 column; 10% *i*-PrOH in hexanes; 1.0 mL/min; retention times: 8.6 min (major), 10.1 min (minor).

^{1}H NMR (400 MHz, $CDCl_3$) δ 8.08 (d, *J* = 7.2 Hz, 2H), 7.59 (t, *J* = 7.4 Hz, 1H), 7.47 (t, *J* = 7.9 Hz, 2H), 7.26 – 7.21 (m, 2H), 6.95 – 6.90 (m, 1H), 6.89 – 6.85 (m, 2H), 5.48 (q, *J* = 6.9 Hz, 1H), 1.72 (d, *J* = 6.9 Hz, 3H) ppm.

^{13}C NMR (101 MHz, $CDCl_3$) δ 199.1, 157.6, 134.3, 133.8, 131.2, 129.7, 129.0, 128.9, 121.6, 115.3, 18.9 ppm.

HRMS (ES+) Calcd for $C_{15}H_{14}NaO_2^+$ [M+Na]$^+$: 249.0886, Found: 249.0903.

An unsuccessful example with α-alkyl acyl-substituted sulfur ylide:

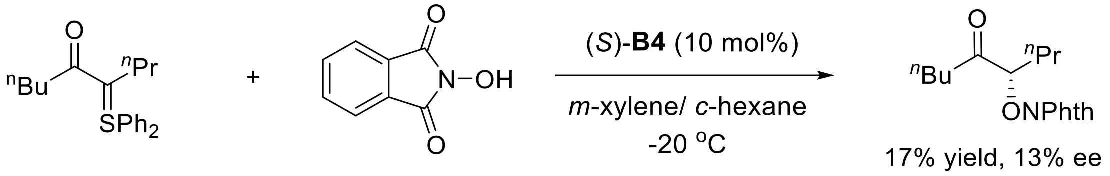

Under the standard conditions, α-alkyl acyl-substituted sulfur ylides were unsuccessful. As an example, we evaluated 4-(diphenyl-λ^4-sulfaneylidene)nonan-5-one as the model substrate. Unfortunately, under the conditions of General Procedure C, a low yield of the desired product (17% yield) and poor enantioselectivity (13% ee) were obtained. This substrate appeared to undergo facile decomposition under the standard conditions.

V. Scale-up Reaction and Product Derivatizations

Reaction at 4-mmol Scale

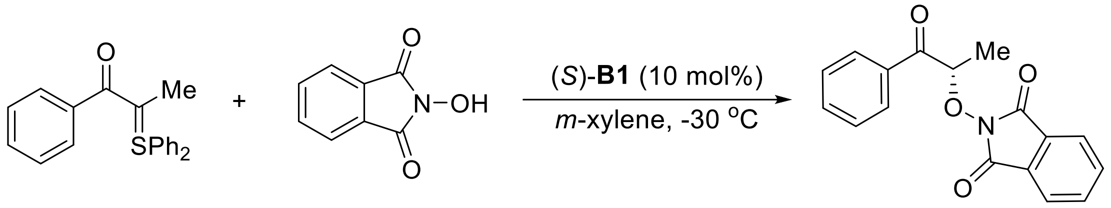

1a 4 mmol | **2a** 4.8 mmol | **3a** 90% yield, 91% ee

A 100-mL flask equipped with a magnetic stirring bar was charged with the sulfonium ylide **1a** (1.27 g, 1.0 equiv, 4 mmol) and catalyst (*S*)-**B1** (232.4 mg, 10.0 mol%, 0.4 mmol), to which was added *m*-xylene (40 mL). The mixture was stirred at -30 °C for 5 min. *N*-Hydroxyphthalimide **2a** (0.78 g, 1.2 equiv, 0.48 mmol) was added to the reaction mixture. Then, the resulting mixture was stirred for 108 h at -30 °C. Upon completion, the reaction mixture was directly subjected to silica gel column chromatography (eluent: *n*-hexane/CH_2Cl_2 = 5:1 → *n*-hexane/EtOAc = 3:1 → CH_2Cl_2) to afford the desired **3a** as a white solid in 91% yield (1.07 g, 91% ee). Ph_2S was recycled in 90% yield (0.67 g), and catalyst **B1** was recovered in 97% yield (225.4 mg). The recovered catalyst showed equally good catalytic activity.

Product Derivatizations

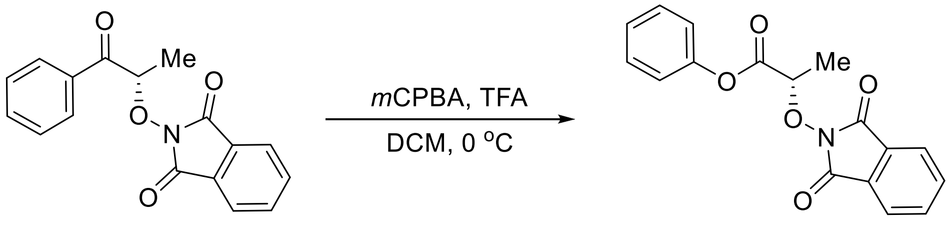

3a 91% ee | **5** 95% yield, 91% ee

Phenyl (*S*)-2-((1,3-dioxoisoindolin-2-yl)oxy)propanoate (5). At 0 °C, to a 5-mL vial charged with **3a** (88.5 mg, 1 equiv, 0.3 mmol), 3-chloroperoxybenzoic acid (75%, 207.1 mg, 3 equiv, 0.9 mmol), trifluoroacetic acid (50 μL, 0.65 mmol) was

added CH_2Cl_2 (2.0 mL). The mixture was stirred at room temperature for 16 h before it was quenched by a saturated aqueous $NaHCO_3$ solution (20 mL). The layers were separated, and the aqueous layer was extracted with CH_2Cl_2 (10.0 mL× 3). The combined organic layers were washed by brine (10 mL), dried over anhydrous Na_2SO_4, filtered, and concentrated. The residue was subjected to silica gel column chromatography (eluent: *n*-hexane/CH_2Cl_2 = 2:1 → 1:3) to afford ester **5** as a white solid in 95% yield (88.7 mg, 91% ee).

$[\alpha]_D^{25}$: -153.1 (*c* = 0.2, CH_2Cl_2). HPLC analysis of the product: Daicel CHIRALPAK® IC-3 column; 20% *i*-PrOH in hexanes; 1.0 mL/min; retention times: 12.7 min (major), 15.5 min (minor).

^{1}H NMR (400 MHz, $CDCl_3$) δ 8.08 – 8.03 (m, 2H), 7.81 (dd, *J* = 5.5, 3.1 Hz, 2H), 7.74 – 7.70 (m, 2H), 7.60 – 7.54 (m, 1H), 7.44 (t, *J* = 7.8 Hz, 2H), 6.72 (q, *J* = 5.5 Hz, 1H), 1.80 (d, *J* = 5.5 Hz, 3H) ppm.

^{13}C NMR (101 MHz, $CDCl_3$) δ 165.6, 163.3, 134.7, 133.7, 130.2, 129.1, 128.9, 128.6, 123.9, 99.4, 18.6 ppm.

HRMS (ES+) Calcd for $C_{17}H_{13}NNaO_5^+$ $[M+Na]^+$: 334.0686, Found: 334.0692.

The ester sulfonium ylide **1ab** also underwent the enantioselective formal O−H bond insertion. Unfortunately, under the standard conditions, the reaction gave moderate yield and low enantioselectivity.

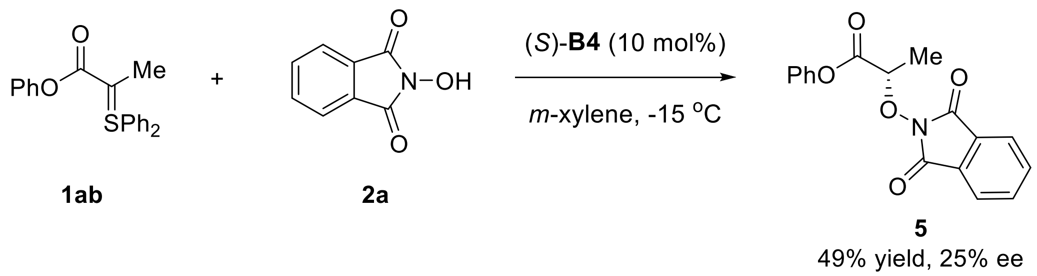

Phenyl (*S*)-2-((1,3-dioxoisoindolin-2-yl)oxy)propanoate (5). A 20-mL vial equipped with a magnetic stirring bar was charged with sulfonium ylide **1ab** (0.4 mmol, 1.0 equiv) and thiourea catalyst (*S*)-**B4** (27.0 mg, 10.0 mol%, 0.04 mmol). Next, *m*-xylene (4.0 mL) was added to the vial, and the mixture was

stirred at -15 °C for 5 min. *N*-Hydroxyphthalimide **2a** (78.2 mg, 1.2 equiv, 0.48 mmol) was then added to the reaction mixture. The mixture was stirred for 72 h at the same temperature. Upon completion, it was directly subjected to silica gel column chromatography to afford the desired product (eluent: *n*-hexane/CH_2Cl_2 = 2:1 → 1:3) to afford ester **5** as a white solid in 49% yield (60.7 mg, 25% ee).

$[\alpha]_D^{25}$: -30.9 (*c* = 0.2, CH_2Cl_2). HPLC analysis of the product: Daicel CHIRALPAK® IC-3 column; 15% *i*-PrOH in hexanes; 1.0 mL/min; retention times: 18.2 min (major), 20.5 min (minor).

HRMS (ES+) Calcd for $C_{17}H_{13}NNaO_5^+$ $[M+Na]^+$: 334.0686, Found: 334.0677.

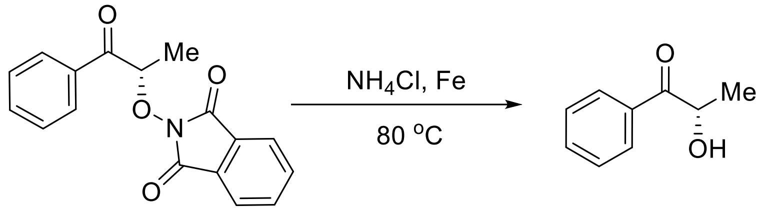

3a
91% ee

6
80% yield, 91% ee

(*S*)-2-Hydroxy-1-phenylpropan-1-one (6). To a 20-mL vial charged with **3a** (147.5 mg, 1 equiv, 0.5 mmol), iron powder (140 mg, 5 equiv, 2.5 mmol), ammonium chloride (134 mg, 5 equiv, 2.5 mmol) was added a mixed solvent (EtOH/H_2O = 1:1, 5.0 mL). The mixture was stirred at 80 °C for 4 h before it was filtered through a short pad of celite, which was washed with EtOAc (5 mL). The filtrate was extracted by EtOAc (5 mL × 2). The combined organic layers were washed by brine (10 mL), dried over anhydrous Na_2SO_4, filtered, and concentrated. The residue was subjected to silica gel column chromatography (eluent: *n*-hexane/EtOAc = 10:1 → 5:1) to afford alcohol **6** as a pale-yellow oil in 80% yield (59.8 mg, 91% ee).

$[\alpha]_D^{25}$: -97.2 (*c* = 0.2, CH_2Cl_2). HPLC analysis of the product: Daicel CHIRALCEL® OD-3 column; 10% *i*-PrOH in hexanes; 1.0 mL/min; retention times: 6.1 min (major), 6.9 min (minor).

^{1}**H NMR** (400 MHz, $CDCl_3$) δ 7.93 (dd, *J* = 8.4, 1.4 Hz, 2H), 7.66 – 7.59 (m, 1H), 7.55 – 7.48 (m, 2H), 5.16 (q, *J* = 7.0 Hz, 1H), 3.80 (br, 1H), 1.45 (d, *J* = 7.0 Hz, 3H) ppm.

^{13}**C NMR** (101 MHz, $CDCl_3$) δ 202.4, 134.0, 133.3, 128.9, 128.7, 69.3, 22.3 ppm.

HRMS (CI+) Calcd for $C_9H_{11}O_2^+$ $[M+H]^+$: 151.0754, Found: 151.0762.

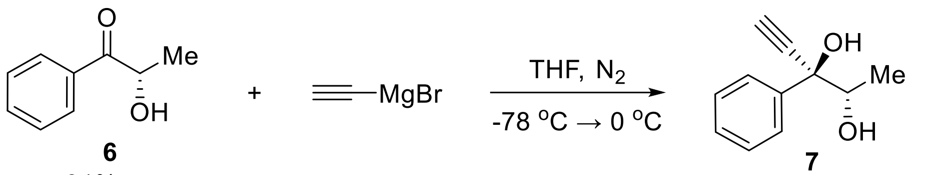

6
91% ee

7
91% yield
91% ee, dr = 17:1

(2*S*,3*R*)-3-Phenylpent-4-yne-2,3-diol (7). At -78 °C under N_2, to a 10-mL vial charged with **6** (30.0 mg, 1 equiv, 0.2 mmol) and anhydrous THF (3.0 mL) was slowly added ethynylmagnesium bromide (1.6 mL, 0.5 M in THF, 4 equiv, 0.8 mmol). The mixture was stirred at -78 °C for 30 min and then at 0 °C for 60 min. Upon completion, the reaction mixture was quenched by a saturated aqueous NH_4Cl solution (5 mL). The mixture was extracted with EtOAc (5 mL × 3). The combined organic layers were washed by brine (10 mL), dried over anhydrous Na_2SO_4, filtered, and concentrated. The residue was subjected to silica gel column chromatography (eluent: *n*-hexane/EtOAc = 5:1 → 2:1) to afford alcohol **7** as a yellow oil in 91% yield (32.2 mg, 91% ee, 17:1 dr). The relative configuration of **7** was determined by analogy with the literature.[3]

$[\alpha]_D^{25}$: +4.2 (*c* = 0.2, CH_2Cl_2). HPLC analysis of the product: Daicel CHIRALCEL® OD-3 column; 3% *i*-PrOH in hexanes; 1.0 mL/min; retention times: 23.7 min (minor), 27.1 min (major).

Major isomer:

(3) Niu, S.; Zhang, H.; Xu, W.; Bagdi, P. R.; Zhang, G.; Liu, J.; Yang, S.; Fang, X. Access to Enantioenriched Compounds Bearing Challenging Tetrasubstituted Stereocenters via Kinetic Resolution of Auxiliary Adjacent Alcohols. *Nat. Commun.* **2021**, *12*, 3735.

^{1}H NMR (400 MHz, $CDCl_3$) δ 7.64 – 7.58 (m, 2H), 7.40 – 7.29 (m, 3H), 4.02 (q, *J* = 6.3 Hz, 1H), 3.25 (br, 1H), 2.68 (s, 1H), 2.44 (br, 1H), 1.05 (d, *J* = 6.3 Hz, 3H) ppm.

^{13}C NMR (101 MHz, $CDCl_3$) δ 140.0, 128.3, 128.3, 126.3, 85.5, 75.9, 74.9, 74.7, 16.2 ppm.

HRMS (CI-) Calcd for $C_{11}H_{11}O_2^-$ $[M-H]^-$: 175.0754, Found: 175.0756.

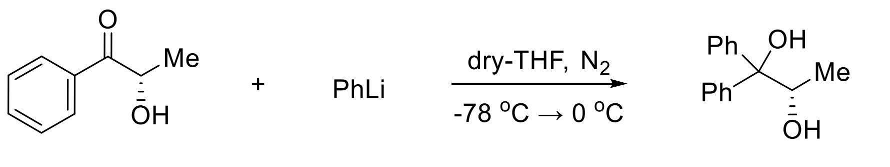

6
91% ee

8
81% yield, 88% ee

(*S*)-1,1-Diphenylpropane-1,2-diol (8). At -78 °C under N_2, to a 10-mL vial charged with **6** (30.0 mg, 1 equiv, 0.2 mmol) and anhydrous THF (1.0 mL) was slowly added phenyllithium (0.6 mL, 1.0 M in Et_2O, 3.0 equiv, 0.6 mmol). The mixture was stirred at -78 °C for 10 min and then at 0 °C for 30 min. Upon completion, the reaction mixture was quenched by a saturated aqueous NH_4Cl solution (3 mL). The mixture was extracted with EtOAc (3 mL× 3). The combined organic layers were washed by brine (10 mL), dried over anhydrous Na_2SO_4, filtered, and concentrated. The residue was subjected to silica gel column chromatography (eluent: *n*-hexane/EtOAc = 5:1 → 3:1) to afford alcohol **8** as a white solid in 81% yield (36.9 mg, 88% ee).

$[\alpha]_D^{25}$: -56.2 (*c* = 0.2, CH_2Cl_2). HPLC analysis of the product: Daicel CHIRALCEL® OD-3 column; 10% *i*-PrOH in hexanes; 1.0 mL/min; retention times: 8.1 min (minor), 13.1 min (major).

^{1}H NMR (400 MHz, $CDCl_3$) δ 7.62 (d, *J* = 7.5 Hz, 2H), 7.45 (d, *J* = 7.5 Hz, 2H), 7.37 (d, *J* = 7.5 Hz, 2H), 7.32 – 7.17 (m, 4H), 4.82 (q, *J* = 6.2 Hz, 1H), 3.08 (br, 1H), 1.92 (br, 1H), 1.11 (d, *J* = 6.2 Hz, 3H) ppm.

^{13}C NMR (101 MHz, $CDCl_3$) δ 145.7, 144.0, 128.8, 128.3, 127.4, 126.9, 126.4, 125.7, 80.0, 71.7, 16.8 ppm.

HRMS (ES+) Calcd for $C_{15}H_{16}NaO_2^+$ $[M+Na]^+$: 251.1043, Found: 251.1046.

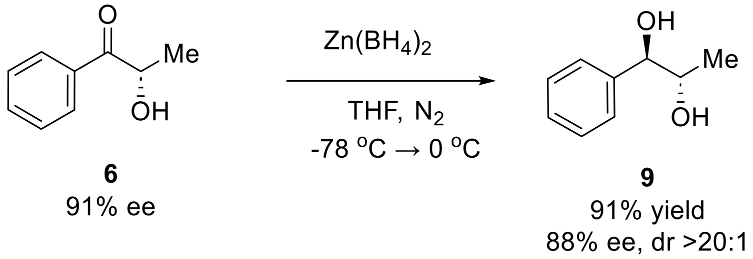

(1*R*,2*S*)-1-Phenylpropane-1,2-diol (9). At -78 °C under N_2, to a 10-mL vial charged with **6** (30.0 mg, 1 equiv, 0.2 mmol) and anhydrous THF (1.0 mL) was slowly added zinc borohydride (0.4 mL, 1.0 M in THF, 2 equiv, 0.4 mmol). The mixture was stirred at -78 °C for 10 min and then at 0 °C for 30 min. Upon completion, the reaction mixture was quenched by a saturated aqueous NH_4Cl solution (5 mL). The mixture was extracted with EtOAc (5 mL × 3). The combined organic layers were washed by brine (10 mL), dried over anhydrous Na_2SO_4, filtered, and concentrated. The residue was subjected to silica gel column chromatography (eluent: *n*-hexane/EtOAc = 3:1 → 1:1) to afford the **9** as white wax in 91% yield (27.8 mg, 88% ee, dr>20:1). The relative configuration of **9** was determined by an analogy with the literature.[4]

$[\alpha]_D^{25}$: -27.7 (*c* = 0.2, CH_2Cl_2). HPLC analysis of the product: Daicel CHIRALPAK® ID-3 column; 10% *i*-PrOH in hexanes; 1.0 mL/min; retention times: 10.7 min (minor), 11.8 min (major).

^{1}H NMR (400 MHz, $CDCl_3$) δ 7.37 – 7.26 (m, 5H), 4.65 (d, *J* = 4.2 Hz, 1H), 4.01 – 3.94 (m, 1H), 2.49 (br, 2H), 1.05 (d, *J* = 6.4 Hz, 3H) ppm.

^{13}C NMR (101 MHz, $CDCl_3$) δ 140.4, 128.4, 127.9, 126.7, 77.5, 71.4, 17.2 ppm.

HRMS (CI-) Calcd for $C_9H_{11}O_2^-$ $[M-H]^-$: 151.0754, Found: 151.0753.

---

(4) Husain, S. M.; Stillger, T.; Dünkelmann, P.; Lödige, M.; Walter, L.; Breitling, E.; Pohl, M.; Bürchner, M.; Krossing, I.; Müller, M.; Romano, D.; Molinari, F. Stereoselective Reduction of 2-Hydroxy Ketones towards *Syn*- and *Anti*-1,2-Diols. *Adv. Synth. Catal.* **2011**, *353*, 2359–2362.

VI. Mechanistic Study

(1) Non-linear effect

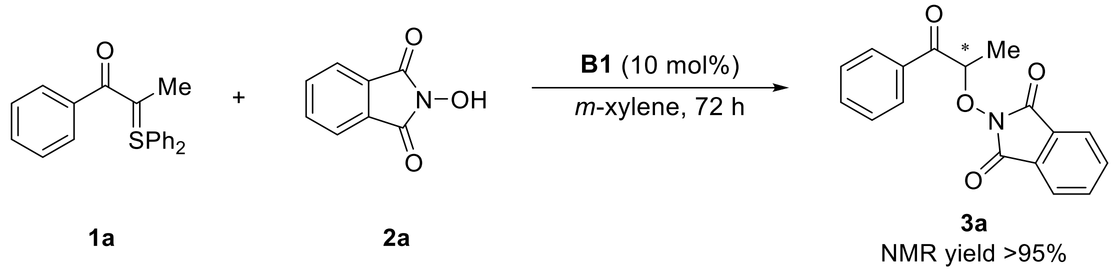

A 5-mL vial equipped with a magnetic stirring bar was charged with the sulfonium ylide **1a** (15.9 mg, 0.05 mmol, 1.0 equiv) and catalyst **B1** solution (0.5 mL, 0.01M in *m*-xylene, 10.0 mol%, 0.005 mmol. The mixture was stirred at -30 °C for 5 min. *N*-Hydroxyphthalimide **2a** (9.6 mg, 1.2 equiv, 0.06 mmol) was added to the reaction mixture, then the resulted mixture was stirred for 72 h at -30°C. Upon completion, the reaction mixture was directly filtered through a short pad of silica gel, which was washed with CH_2Cl_2 (5.0 mL). The filtrate was concentrated in vacuo. CH_2Br_2 (0.7 μL, 0.1 mmol) was added to the mixture as internal standard, the conversion was observed by ^{1}H NMR analysis. The ee was determined by HPLC with a chiral stationary phase.

Preparation of a **B1** solution:

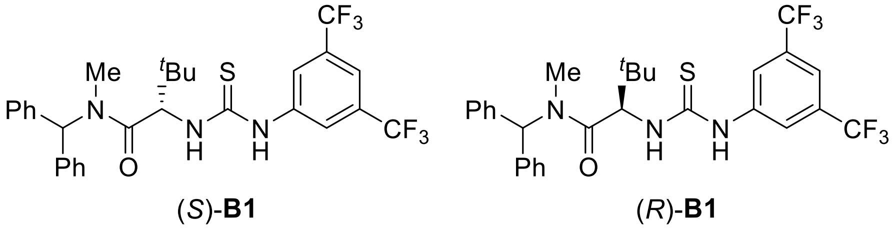

(*S*)-Solution: (*S*)-**B1** (17.4 mg, 0.03 mmol) was dissolved in *m*-xylene (3.0 mL).

(*R*)-Solution: (*R*)-**B1** (8.7 mg, 0.015 mmol) was dissolved in *m*-xylene (1.5 mL).

ee 0% solution: 0.25 mL (*S*)-solution + 0.25 mL (*R*)-solution.

ee 20% solution: 0.30 mL (*S*)-solution + 0.20 mL (*R*)-solution.

ee 40% solution: 0.35 mL (*S*)-solution + 0.15 mL (*R*)-solution.

ee 60% solution: 0.40 mL (*S*)-solution + 0.10 mL (*R*)-solution.

ee 80% solution: 0.45 mL (*S*)-solution + 0.05 mL (*R*)-solution.

ee 100% solution: 0.50 mL (*S*)-solution.

Entry	ee of (*S*)-**B1** (%)	ee of **3a** (%)
1	0	-1.6
2	20	13.3
3	40	33.2
4	60	56.1
5	80	67.6
6	100	93.6

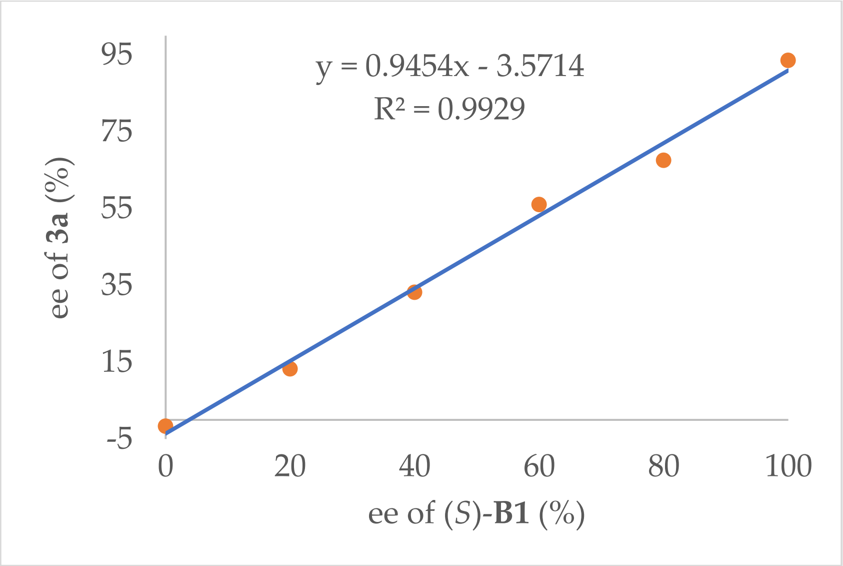

(2) *In situ* ^{1}H NMR analysis of the reaction species involved

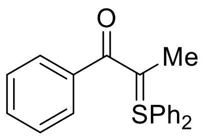

1a

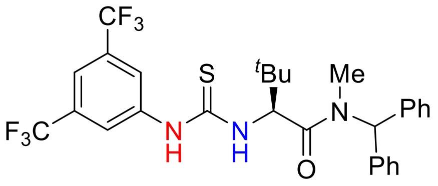

B1

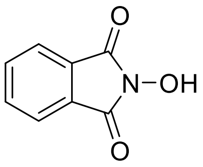

2a

Interaction between B1 with 1a:

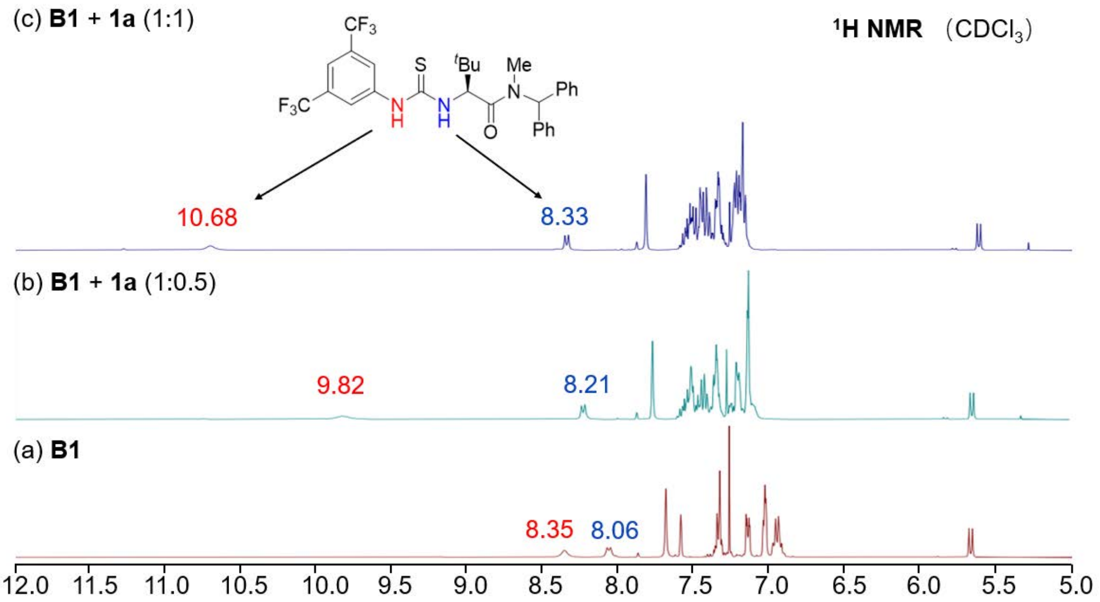

(a) **B1** in $CDCl_3$ (0.05 mL)

(b) **B1** (0.02 mmol) + **1a** (0.01 mmol) in $CDCl_3$ (0.05 mL)

(c) **B1** (0.02 mmol) + **1a** (0.02 mmol) in $CDCl_3$ (0.05 mL)

Interaction between B1 with 2a:

2a is insoluble in the reaction system or $CDCl_3$, and no significant effect on the signals of **B1** was found in ^{1}H NMR spectroscopy after addition of **2**.

VII. DFT Calculations

Computational Details

All structures were optimized and characterized in *m*-xylene with the SMD[5] solvent model (SCRF = SMD) at M06-2X[6]/6-31G(d) level. Harmonic frequency analysis calculations at the same level were performed to verify the optimized geometries to be minima (no imaginary frequency) or transition states (TSs, having unique one imaginary frequency). The energies were further improved by M06-2X/6-311++G(d,p)//M06-2X/6-31G(d) single-point calculations with solvent effects accounted by the SMD solvent model, using the experimental solvent (*m*-xylene). All DFT calculations were carried out using Gaussian 09

(5) Marenich, A. V.; Cramer, C. J.; Truhlar, D. G. Universal Solvation Model Based on Solute Electron Density and on a Continuum Model of the Solvent Defined by the Bulk Dielectric Constant and Atomic Surface Tensions. *J. Phys. Chem. B.* **2009**, *113*, 6378–6396.

(6) (a) Zhao, Y.; Truhlar, D. G. Benchmark Energetic Data in a Model System for Grubbs II Metathesis Catalysis and Their Use for the Development, Assessment, and Validation of Electronic Structure Methods. *J. Chem. Theory Comput.* **2009**, *5*, 324–333; (b) Zhao, Y.; Truhlar, D. G. The M06 suite of density functionals for main group thermochemistry, thermochemical kinetics, noncovalent interactions, excited states, and transition elements: two new functionals and systematic testing of four M06-class functionals and 12 other functionals. *Theor. Chem. Acc.* **2008**, *120*, 215–241; (c) Zhao, Y.; Truhlar, D. G. Density Functionals with Broad Applicability in Chemistry. *Acc. Chem. Res.* **2008**, *41*, 157–167.

program.[7] Computed structures are illustrated using the CYLview.[8]

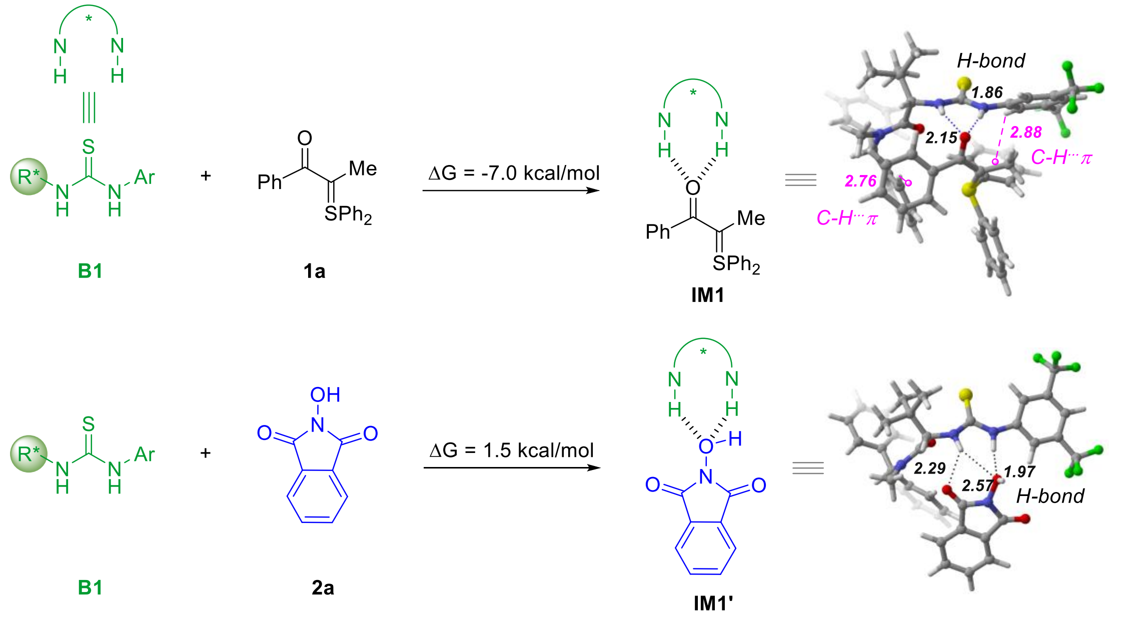

Figure S1. Comparison of free energies (in kcal/mol) for the possible complexation modes of **B1**, **1a** and **2a**. Bond length in Å. The complex **IM1** is 8.5 kcal/mol more favorable than **IM1'**, which is stabilized by more favorable hydrogen bonds and C···H interactions.

---

(7) Frisch, M. J.; Trucks, G. W.; Schlegel, H. B.; Scuseria, G. E.; Robb, M. A.; Cheeseman, J. R.; Scalmani, G.; Barone, V.; Mennucci, B.; Petersson, G. A.; Nakatsuji, H.; Caricato, M.; Li, X.; Hratchian, H. P.; Izmaylov, A. F.; Bloino, J.; Zheng, G.; Sonnenberg, J. L.; Hada, M.; Ehara, M.; Toyota, K.; Fukuda, R.; Hasegawa, J.; Ishida, M.; Nakajima, T.; Honda, Y.; Kitao, O.; Nakai, H.; Vreven, T.; Montgomery, J. A., Jr.; Peralta, J. E.; Ogliaro, F.; Bearpark, M.; Heyd, J. J.; Brothers, E.; Kudin, K. N.; Staroverov, V. N.; Keith, T.; Kobayashi, R.; Normand, J.; Raghavachari, K.; Rendell, A.; Burant, J. C.; Iyengar, S. S.; Tomasi, J.; Cossi, M.; Rega, N.; Millam, J. M.; Klene, M.; Knox, J. E.; Cross, J. B.; Bakken, V.; Adamo, C.; Jaramillo, J.; Gomperts, R.; Stratmann, R. E.; Yazyev, O.; Austin, A. J.; Cammi, R.; Pomelli, C.; Ochterski, J. W.; Martin, R. L.; Morokuma, K.; Zakrzewski, V. G.; Voth, G. A.; Salvador, P.; Dannenberg, J. J.; Dapprich, S.; Daniels, A. D.; Farkas, O.; Foresman, J. B.; Ortiz, J. V.; Cioslowski, J.; Fox, D. J. Gaussian 09, Rev. A.01; Gaussian, Inc.: Wallingford, CT, 2010.

(8) Legault, C. Y. *CYLView, version 1.0 b*; Université de Sherbrooke: Sherbrooke, Quebec, Canada, 2009. http://www.cylview.org.

Cartesian Coordinates in Å, SCF Energies and Free Energies (in a.u.) at 298.15 K and 1 atm for the Optimized Structures [BSI= 6-31G(d), BSII=6-311++G(d,p)]

B1

M06-2X/BSI SCF energy in *m*-xylene: -2358.509998 a.u.
M06-2X/BSII SCF energy in *m*-xylene: -2359.135975 a.u.
M06-2X/BSII free energy in *m*-xylene: -2358.660693 a.u.

C	-2.69105600	3.30021400	-0.50553700
C	-2.15276000	2.53342100	0.74350600
C	-2.33016400	2.62239900	-1.83313700
N	-0.78012500	2.82974800	1.20353800
C	-2.27541700	1.00167100	0.57853400
H	-2.75808900	2.88167200	1.58432500
C	-2.18167100	4.74328400	-0.45537200
C	-4.22549400	3.33242400	-0.41806800
H	-2.71065800	3.23239600	-2.66046600
H	-2.79500900	1.63196900	-1.91146800
H	-1.25502700	2.50441300	-1.96541200
C	0.38938700	2.42722500	0.55248100
H	-0.72120800	2.57245600	2.18670100
N	-3.44323800	0.35160900	0.84366500
O	-1.29497600	0.38111000	0.18147800
H	-1.09779500	4.78975500	-0.57494700
H	-2.44387300	5.21737800	0.49843500
H	-2.64792900	5.32304600	-1.26000100
H	-4.62013900	3.91876900	-1.25466600
H	-4.57413200	3.80350400	0.50950400
H	-4.66031200	2.33001600	-0.49254000
N	1.07541500	1.47660300	1.27449100
C	-3.48812000	-1.09992000	0.58617700
C	-4.53779600	0.85062200	1.66514900
C	2.14040800	0.73933800	0.67122900
H	0.44528200	0.86437000	1.78726100
C	-3.15942100	-1.42779300	-0.86957700
C	-2.71994300	-1.91279900	1.62473900
H	-4.54706100	-1.35786200	0.71674100
H	-4.61541800	0.24610700	2.57701200
H	-4.38438800	1.88778000	1.95371000
H	-5.48648400	0.78615500	1.12257700
C	3.46616900	1.11139500	0.85851800
C	1.81692500	-0.38090600	-0.08822900

C	-4.11768400	-1.13236600	-1.83988600
C	-1.94279200	-1.98409400	-1.26804000
C	-2.72817300	-3.30810100	1.52721900
C	-2.06373900	-1.32514300	2.70605500
C	4.47017400	0.34215700	0.27966800
H	3.70230500	1.99313300	1.44446500
C	2.83581400	-1.14368400	-0.64814400
H	0.76982800	-0.62683000	-0.24641300
C	-3.86416400	-1.37514300	-3.18858600
H	-5.07105200	-0.70255300	-1.53704400
C	-1.68597800	-2.22937000	-2.61276400
H	-1.18666800	-2.20831100	-0.52253600
C	-2.08338200	-4.09340300	2.47652400
H	-3.23628000	-3.78262400	0.69149700
C	-1.41924400	-2.10963000	3.66153300
H	-2.05790900	-0.24395600	2.81362300
C	5.91186700	0.68786000	0.51901900
C	4.16850500	-0.79115300	-0.47170300
C	2.46529300	-2.32435500	-1.49952000
C	-2.64503500	-1.92340700	-3.57690300
H	-4.61986300	-1.14004400	-3.93202000
H	-0.72575700	-2.64268800	-2.90652700
C	-1.42323100	-3.49585600	3.54882600
H	-2.09694000	-5.17456100	2.37819500
H	-0.91399100	-1.63253600	4.49609700
F	6.06838600	1.96904400	0.87468300
F	6.43962200	-0.06093100	1.50235900
F	6.66044800	0.47290400	-0.57168000
H	4.96135900	-1.38520100	-0.91439800
F	2.10623700	-1.95394000	-2.73810400
F	1.42152600	-2.99625500	-0.98483600
F	3.48078500	-3.18970100	-1.62496500
H	-2.44148100	-2.11201600	-4.62656100
H	-0.91833500	-4.10689800	4.29050200
S	0.99586100	3.09369700	-0.83804400

1a

M06-2X/BSI SCF energy in *m*-xylene: -1284.010731 a.u.
M06-2X/BSII SCF energy in *m*-xylene: -1284.290157 a.u.
M06-2X/BSII free energy in *m*-xylene: -1284.008113 a.u.

C	-2.68705800	-1.29913700	-0.51431600

C	-2.79520300	-0.21215100	0.35680400
C	-3.84417800	0.69464000	0.18291600
C	-4.74366300	0.54579400	-0.86659000
C	-4.62105500	-0.53302000	-1.74108700
C	-3.59797500	-1.46004500	-1.55678700
H	-1.90289600	-2.03718600	-0.36394000
H	-3.94253300	1.51368300	0.88874200
H	-5.54519500	1.26637100	-0.99910800
H	-3.51214300	-2.31410900	-2.22203300
C	-1.88411500	0.01212000	1.54301500
O	-2.41121300	0.31267000	2.61948300
C	-0.46036400	-0.12463600	1.42555100
S	0.22118300	-0.05482600	-0.12364700
C	1.21968900	1.44405000	-0.28366700
C	0.65375400	2.46630500	-1.04171200
C	2.43496300	1.62023800	0.37743400
C	1.32096000	3.68391300	-1.14869700
H	-0.29911900	2.31017500	-1.54027600
C	3.09106700	2.84234900	0.26567000
H	2.86849500	0.81210600	0.95995100
C	2.53694300	3.87049400	-0.49637600
H	0.89016500	4.48294900	-1.74351500
H	4.03746600	2.99198500	0.77566300
H	3.05556400	4.82035200	-0.58092300
C	1.50979800	-1.32329100	-0.22869900
C	2.48108000	-1.23281600	-1.22435900
C	1.44444100	-2.41998500	0.62273700
C	3.41352000	-2.25693100	-1.35258200
H	2.51515700	-0.37220700	-1.88643000
C	2.38703900	-3.43853300	0.48572600
H	0.66610500	-2.46559500	1.38065700
C	3.37006100	-3.35672300	-0.49628700
H	4.17576000	-2.19326600	-2.12275400
H	2.35125600	-4.29440900	1.15231400
H	4.10331500	-4.15048500	-0.59834800
C	0.40022300	0.14677200	2.62722100
H	0.63009700	1.21462100	2.75196700
H	-0.14433400	-0.17211400	3.51960700
H	1.35197500	-0.39697700	2.59158200
H	-5.32791300	-0.65648400	-2.55615000

2a
M06-2X/BSI SCF energy in *m*-xylene: -588.0219717 a.u.
M06-2X/BSII SCF energy in *m*-xylene: -588.2032034 a.u.
M06-2X/BSII free energy in *m*-xylene: -588.1162784 a.u.

C	-2.87843500	0.69826400	-0.00226200
C	-2.87852200	-0.69834100	-0.00222800
C	-1.68193100	-1.42169700	0.00265300
C	-0.50390600	-0.69698600	0.00368000
C	-0.50385400	0.69665800	0.00368200
C	-1.68175800	1.42149700	0.00259000
H	-3.82449000	1.23008900	-0.00719900
H	-3.82463500	-1.23005200	-0.00722400
H	-1.67184200	-2.50686000	0.00275600
H	-1.67151700	2.50665800	0.00274400
C	0.91027900	-1.17776500	0.00628700
C	0.91040600	1.17765100	0.00658300
O	1.35040000	2.29725300	-0.02316900
O	1.35119400	-2.29689900	-0.02305300
N	1.66826100	0.00024800	0.07253300
O	3.01674700	-0.00010000	-0.12710900
H	3.41424300	0.00071000	0.76194200

IM1
M06-2X/BSI SCF energy in *m*-xylene: -3642.565752 a.u.
M06-2X/BSII SCF energy in *m*-xylene: -3643.468506 a.u.
M06-2X/BSII free energy in *m*-xylene: -3642.680016 a.u.

C	2.45830400	-3.50534700	1.79549200
C	1.98418700	-2.00207600	1.73408600
C	2.85138000	-4.07889400	0.42726000
N	0.54636500	-1.72041500	1.68742200
C	2.71337400	-1.22992100	0.61965500
H	2.25388600	-1.58178800	2.70545500
C	1.36853400	-4.34901700	2.46238100
C	3.71209300	-3.57951600	2.68308000
H	3.06776100	-5.14820900	0.53732400
H	3.76112200	-3.59660700	0.04826000
H	2.06079000	-3.95337800	-0.31084800
C	-0.45167700	-2.04307600	0.82270400
H	0.26305100	-1.04930300	2.39896300
N	3.97977500	-0.75832000	0.83385900

O	2.18586300	-1.11488300	-0.47603000
H	0.48210500	-4.43351100	1.83127200
H	1.07097300	-3.92239600	3.42802500
H	1.75400600	-5.35826000	2.64577700
H	4.00012000	-4.62866800	2.81000500
H	3.53870000	-3.16032700	3.68232500
H	4.56479200	-3.06882400	2.22626000
N	-1.59938200	-1.35352700	1.13110600
C	4.74148500	-0.29058000	-0.34174200
C	4.63643500	-0.53044600	2.11049000
C	-2.88043300	-1.52287300	0.58472700
H	-1.56446300	-0.72650200	1.94743600
C	5.05309800	-1.46652500	-1.25896800
C	4.16778700	0.96995400	-0.97934700
H	5.70775500	0.01445700	0.07846300
H	4.85464900	0.53799500	2.22564300
H	4.00949400	-0.83930500	2.94323400
H	5.57921200	-1.08658200	2.16664600
C	-3.96648700	-1.48196300	1.45801900
C	-3.11240500	-1.61439000	-0.79393900
C	6.16983100	-2.24666900	-0.94377500
C	4.24590300	-1.84952800	-2.33334000
C	4.51645400	1.32962600	-2.28426400
C	3.40668900	1.86151800	-0.21808900
C	-5.26689100	-1.54250300	0.96227600
H	-3.79236200	-1.39659500	2.52800800
C	-4.41629100	-1.70784300	-1.26017300
H	-2.27458600	-1.60416200	-1.47900800
C	6.47070200	-3.39578100	-1.66930200
H	6.80959700	-1.95180000	-0.11416000
C	4.54944600	-2.99527400	-3.06501600
H	3.37120800	-1.25943700	-2.58033200
C	4.10008100	2.54433100	-2.82380300
H	5.12109700	0.65693500	-2.88505600
C	3.00939600	3.08527900	-0.75138900
H	3.12324900	1.60254600	0.80105300
C	-6.39660600	-1.47156500	1.94680000
C	-5.51043400	-1.67078200	-0.39636600
C	-4.67712500	-1.78191900	-2.73665600
C	5.65604900	-3.77400000	-2.73312800
H	7.34077300	-3.98991200	-1.40688700
H	3.91044300	-3.28609300	-3.89321900
C	3.34765900	3.43043200	-2.05799500

H	4.37371100	2.79977500	-3.84322600
H	2.43476100	3.77377500	-0.13722400
F	-6.34217400	-2.47266300	2.83908400
F	-6.35700300	-0.32959700	2.65443600
F	-7.59493400	-1.53006300	1.35158000
H	-6.52298800	-1.74015800	-0.77784000
F	-3.61699600	-2.22766500	-3.42148400
F	-4.99691100	-0.57812400	-3.25022000
F	-5.70869000	-2.59436900	-3.01514500
H	5.88413600	-4.66935500	-3.30340700
H	3.03446700	4.38551000	-2.46985500
C	1.30696600	3.28883500	2.48766300
C	0.88425600	1.98644500	2.77839900
C	1.71436700	1.18003800	3.56578400
C	2.95147000	1.64104700	4.00407400
C	3.38520300	2.92026700	3.65844100
C	2.55394200	3.74637000	2.90730400
H	0.64632900	3.97716100	1.96848000
H	1.35218800	0.20313800	3.87291600
H	3.57633800	1.00699900	4.62652100
H	2.86302100	4.75892400	2.66599100
C	-0.47365500	1.44724300	2.39448400
O	-0.95750900	0.55633700	3.14450100
C	-1.19181700	1.95651400	1.28728800
S	-0.27600100	2.52811700	-0.04268300
C	-0.82637700	1.61580900	-1.48975100
C	-0.00601700	0.56191300	-1.87985300
C	-2.02096800	1.90797600	-2.14933500
C	-0.39567100	-0.21963100	-2.96714900
H	0.91652700	0.34057200	-1.34892900
C	-2.39147600	1.12220100	-3.23548000
H	-2.64420600	2.73711500	-1.82661100
C	-1.57837900	0.06328800	-3.64523600
H	0.22976800	-1.05243700	-3.27088900
H	-3.31894100	1.33054000	-3.75758100
H	-1.87777800	-0.54759300	-4.49032700
C	-0.80184400	4.20758800	-0.45123200
C	-0.40360800	4.75761900	-1.66941000
C	-1.48460100	4.95888500	0.49812000
C	-0.71447800	6.08631500	-1.93974600
H	0.13402800	4.15503300	-2.39710300
C	-1.79113600	6.28915000	0.21306500
H	-1.76577300	4.50511200	1.44575900

C	-1.40828300	6.85055500	-1.00145700
H	-0.41189000	6.52536000	-2.88516600
H	-2.33068900	6.88259700	0.94422000
H	-1.64788700	7.88645300	-1.21927800
C	-2.66273100	1.71972100	1.10101500
H	-2.89138200	1.03370900	0.27740900
H	-3.06920500	1.29431200	2.02305900
H	-3.19973000	2.65645000	0.89976900
H	4.35129100	3.28046800	3.99864500
S	-0.38946500	-3.18311700	-0.40911000

IM1′

M06-2X/BSI SCF energy in *m*-xylene: -2946.553492 a.u.
M06-2X/BSII SCF energy in *m*-xylene: -2947.358508 a.u.
M06-2X/BSII free energy in *m*-xylene: -2946.774582 a.u.

C	-2.11430900	-2.79167800	-2.32275100
C	-1.63061900	-1.37635200	-1.84586400
C	-2.48309700	-3.74417500	-1.17828800
N	-0.19258500	-1.11808300	-1.70333100
C	-2.36306600	-0.93559100	-0.56807600
H	-1.88028100	-0.70564200	-2.66809700
C	-1.04836300	-3.40373700	-3.23498800
C	-3.39064000	-2.58039200	-3.15501100
H	-2.73645300	-4.72389300	-1.60082300
H	-3.36490900	-3.38226800	-0.63564700
H	-1.66822700	-3.86842900	-0.46634700
C	0.77472300	-1.62524800	-0.89079800
H	0.01899200	-0.20009800	-2.08467700
N	-3.60144100	-0.35260900	-0.67816100
O	-1.87576300	-1.18150600	0.52136000
H	-0.14605700	-3.66502900	-2.67793800
H	-0.77358300	-2.71319800	-4.04187900
H	-1.44259500	-4.31728400	-3.69427100
H	-3.73302900	-3.54397800	-3.54769300
H	-3.21908100	-1.91317600	-4.00934200
H	-4.20385100	-2.17324700	-2.54486500
N	1.81966100	-0.73015800	-0.78839600
C	-4.41777700	-0.24005600	0.54395000
C	-4.12836100	0.35729100	-1.83215200
C	3.09689900	-0.82804600	-0.22520500
H	1.61342400	0.21238900	-1.10109800

C	-4.76459800	-1.62498800	1.07751300
C	-3.87626100	0.75874100	1.56101600
H	-5.36701100	0.18058300	0.18848500
H	-4.32590500	1.40123300	-1.55772500
H	-3.42210800	0.36529400	-2.65800000
H	-5.06761600	-0.09070600	-2.17680000
C	3.74234600	0.38864300	0.03956300
C	3.78515100	-2.01992000	0.02926200
C	-5.82436900	-2.30515100	0.47034500
C	-4.03979700	-2.26322300	2.08564200
C	-4.48022100	0.86731200	2.81689000
C	-2.84965100	1.64334000	1.23264600
C	5.02823300	0.40899200	0.55943900
H	3.23484900	1.32828600	-0.16822100
C	5.07023300	-1.96787800	0.55952300
H	3.32237500	-2.97460000	-0.17613800
C	-6.14983200	-3.60469800	0.84727200
H	-6.39733600	-1.81143200	-0.31256700
C	-4.36840400	-3.56154100	2.46869400
H	-3.20729700	-1.74831200	2.55154300
C	-4.05597000	1.83161600	3.72718200
H	-5.28332900	0.18705700	3.08843600
C	-2.42628700	2.61154000	2.14010100
H	-2.37025100	1.56062500	0.26128700
C	5.65713200	1.74672000	0.82435200
C	5.71317900	-0.76813700	0.83636400
C	5.78449000	-3.27137200	0.78155100
C	-5.41811300	-4.23694700	1.84936300
H	-6.97399600	-4.11910500	0.36230100
H	-3.79310300	-4.05133100	3.24840500
C	-3.02574100	2.70739700	3.39267200
H	-4.53107900	1.89669400	4.70157600
H	-1.61975600	3.28785500	1.86802700
F	5.66604700	2.51041700	-0.28026000
F	4.98394100	2.43581800	1.75877500
F	6.92392200	1.63479600	1.24501700
H	6.71350700	-0.74984100	1.25099700
F	6.17308900	-3.82331000	-0.38002400
F	5.00031100	-4.17083700	1.39020400
F	6.88455800	-3.12044500	1.53293700
H	-5.66600700	-5.25125500	2.14742000
H	-2.68941300	3.45510500	4.10447000
C	-1.62987000	6.29100500	0.24767500

C	-2.71375800	5.51314600	-0.16359800
C	-2.52271900	4.30890800	-0.84824000
C	-1.21848300	3.92227000	-1.09714300
C	-0.13407500	4.70461000	-0.69134800
C	-0.31331900	5.89659400	-0.01379800
H	-1.81350800	7.21710400	0.78256800
H	-3.72252700	5.84403200	0.06020800
H	-3.36277000	3.69721900	-1.15997500
H	0.53505500	6.49449200	0.30225100
C	-0.69774600	2.69692800	-1.76341700
C	1.12706700	4.02366700	-1.09912700
O	2.27859200	4.28439100	-0.88514600
O	-1.26597700	1.69725800	-2.13967600
N	0.67828700	2.91626200	-1.85857800
S	0.77753200	-3.12610600	-0.15784700
O	1.51836300	1.88102800	-2.15149200
H	1.88232700	2.05981100	-3.03852300

TS1A

M06-2X/BSI SCF energy in *m*-xylene: -4230.589719 a.u.
M06-2X/BSII SCF energy in *m*-xylene: -4231.670569 a.u.
M06-2X/BSII free energy in *m*-xylene: -4230.775456 a.u.

C	3.52467200	-2.13496200	-3.09654500
C	2.27963600	-1.81567500	-2.18233400
C	4.87339700	-2.03189200	-2.37139300
N	1.71776100	-0.46079000	-2.21961000
C	2.49704900	-2.23605300	-0.71403300
H	1.46258900	-2.40056200	-2.61365900
C	3.47612200	-1.22665900	-4.32904800
C	3.40744300	-3.58670700	-3.59001500
H	5.67712500	-2.22831500	-3.09092500
H	4.95191400	-2.78810300	-1.58100200
H	5.03512300	-1.05060700	-1.92959800
C	2.20910500	0.76270400	-1.87628200
H	0.70392900	-0.48985000	-2.28434500
N	2.40844900	-3.55383400	-0.35925300
O	2.81603700	-1.38884900	0.10670900
H	3.63425000	-0.17897100	-4.06787700
H	2.51336900	-1.31704900	-4.84661800
H	4.26278100	-1.52679400	-5.03025100
H	4.19865800	-3.77921600	-4.32240400

H	2.44661800	-3.78239800	-4.08225200
H	3.54456800	-4.30497400	-2.77740800
N	1.18172500	1.65719900	-1.69520100
C	2.94337400	-3.94355800	0.96352900
C	1.70582200	-4.61038800	-1.06996000
C	1.16812600	3.05054600	-1.54537800
H	0.24023300	1.26752900	-1.73504200
C	4.45309600	-3.73807200	0.98961800
C	2.13382300	-3.38979500	2.13176500
H	2.79509000	-5.03013600	0.99346900
H	0.90431600	-5.00597600	-0.43537600
H	1.25676700	-4.24157800	-1.99014200
H	2.38410700	-5.43294400	-1.32292000
C	-0.02835500	3.68603800	-1.90149100
C	2.19650900	3.82276400	-0.99578000
C	5.25309200	-4.76837200	0.48705500
C	5.06628200	-2.55068200	1.40027200
C	2.71099300	-3.21386700	3.39174700
C	0.75608100	-3.18666600	1.99412200
C	-0.20936100	5.04143200	-1.66533800
H	-0.83089800	3.10711000	-2.35273500
C	1.99532600	5.18351700	-0.78825700
H	3.13316400	3.36034100	-0.72101500
C	6.63283100	-4.61787000	0.37769200
H	4.78755200	-5.69960400	0.17010100
C	6.44654500	-2.40026700	1.29633500
H	4.45636100	-1.74157100	1.78391800
C	1.93869300	-2.81438900	4.48083800
H	3.77217400	-3.39339800	3.53017300
C	-0.01957100	-2.81083800	3.08827600
H	0.27706500	-3.32636700	1.02631000
C	-1.55217000	5.63000400	-1.98285500
C	0.79790800	5.81523600	-1.10186100
C	3.08392700	5.94529900	-0.09073600
C	7.23278500	-3.42780500	0.78033600
H	7.23661800	-5.42913400	-0.01765500
H	6.90747400	-1.46926400	1.61218300
C	0.57018600	-2.61144000	4.33541200
H	2.41218700	-2.67293600	5.44777700
H	-1.09057000	-2.68207300	2.96232200
F	-1.95425400	5.31111300	-3.22140400
F	-2.49430800	5.16507000	-1.14282200
F	-1.55710300	6.96558300	-1.88531800

H	0.65444900	6.87363300	-0.91899100
F	4.30169900	5.59620800	-0.52245400
F	3.06846900	5.70603400	1.23872700
F	2.95928300	7.26934600	-0.24549300
H	8.30802800	-3.30220000	0.69600600
H	-0.03589200	-2.31798400	5.18739900
C	-2.73981900	-2.39983100	0.71146500
C	-1.96025600	-1.84722500	-0.31315400
C	-1.40026600	-2.69500200	-1.27812800
C	-1.60860800	-4.06701900	-1.21731000
C	-2.36107900	-4.61271500	-0.17693200
C	-2.92894400	-3.77777400	0.78171500
H	-3.27483700	-1.76515200	1.41426300
H	-0.84025000	-2.25687800	-2.09893600
H	-1.19823700	-4.71424300	-1.98626300
H	-3.55848900	-4.18673700	1.56495500
C	-1.77086900	-0.37094600	-0.48379300
O	-1.22656800	0.05463100	-1.49773900
C	-2.33897200	0.58850400	0.52991500
S	-1.52015900	0.26566100	2.10592400
C	-0.00541300	1.21950600	2.08449200
C	1.13826300	0.52136100	1.70758200
C	0.01609400	2.58786000	2.36467200
C	2.34288800	1.21648000	1.60596700
H	1.11251700	-0.54475700	1.49720900
C	1.22785700	3.26099100	2.27398900
H	-0.89088400	3.11208900	2.65005700
C	2.38639000	2.57603500	1.89966800
H	3.22740500	0.68077300	1.27997000
H	1.26818000	4.32644800	2.47567400
H	3.32498500	3.11714500	1.82442000
C	-2.50243700	1.08187200	3.36983900
C	-1.91697400	1.13727400	4.63691800
C	-3.78781300	1.55653400	3.13058600
C	-2.63107800	1.70870400	5.68207000
H	-0.91429000	0.75122500	4.80104900
C	-4.48825200	2.12232600	4.19682800
H	-4.25953100	1.48283200	2.15657900
C	-3.91641000	2.20276600	5.46153900
H	-2.18304500	1.76459600	6.66866500
H	-5.49181100	2.49695200	4.02503500
H	-4.47223400	2.64799300	6.28049300
C	-2.28099800	2.03571500	0.09075700

H	-1.25918300	2.37816400	-0.09560200
H	-2.86564000	2.11728100	-0.82970500
H	-2.73352000	2.69555200	0.83695700
H	-3.54227800	0.24651000	0.70723600
H	-2.52674000	-5.68469300	-0.13029600
C	-5.83947000	-3.16637500	-4.11091300
C	-6.31797900	-4.12763000	-3.22064000
C	-6.33261700	-3.89041800	-1.84012600
C	-5.85914300	-2.66940000	-1.40260400
C	-5.37970700	-1.71041500	-2.29108100
C	-5.35685700	-1.93303700	-3.65380600
H	-5.84046600	-3.37964200	-5.17537700
H	-6.68466000	-5.07414700	-3.60556800
H	-6.70024700	-4.63193700	-1.13787000
H	-4.97819400	-1.18106800	-4.33866900
C	-5.71700500	-2.12832600	-0.01354300
C	-4.92185900	-0.53005000	-1.49204600
O	-4.38316600	0.48528500	-1.88543900
O	-5.96633200	-2.67299800	1.03837700
N	-5.19445600	-0.84751600	-0.17037300
O	-4.87381000	-0.06097600	0.87027000
S	3.82593700	1.19025700	-1.77523000

IM2A

M06-2X/BSI SCF energy in *m*-xylene: -4230.595145 a.u.
M06-2X/BSII SCF energy in *m*-xylene: -4231.675914 a.u.
M06-2X/BSII free energy in *m*-xylene: -4230.775773 a.u.

C	-2.84202000	3.49282400	-2.78766500
C	-1.76448500	2.65096000	-2.00103800
C	-4.07824100	3.84266700	-1.94819800
N	-1.74996700	1.19764300	-2.20337200
C	-1.73735900	2.95283500	-0.48701400
H	-0.80187600	2.94158100	-2.43317000
C	-3.22639100	2.74230500	-4.06762300
C	-2.21356700	4.82839100	-3.22105400
H	-4.78695100	4.39882200	-2.57303700
H	-3.80698100	4.48875900	-1.10476500
H	-4.57726300	2.95775600	-1.55731500
C	-2.65138100	0.21326100	-1.92160600
H	-0.79732100	0.85758100	-2.29190900
N	-1.21371300	4.13674600	-0.04484200

O	-2.24069700	2.15969900	0.29424500
H	-3.76609200	1.81832200	-3.85450600
H	-2.33904700	2.49807300	-4.66385600
H	-3.87475300	3.38019300	-4.67824200
H	-2.90933100	5.34731300	-3.88881700
H	-1.27333600	4.68638600	-3.76934200
H	-2.03331600	5.49336200	-2.37281400
N	-2.03719000	-1.01490800	-1.97381700
C	-1.46829800	4.51079800	1.36123300
C	-0.22630700	4.96255000	-0.72357000
C	-2.48568300	-2.31556200	-1.72575500
H	-1.02510000	-0.98627800	-2.06090600
C	-2.95381100	4.76969500	1.57809000
C	-0.75206800	3.61352900	2.36480500
H	-0.98805600	5.49236500	1.45752600
H	0.70573400	4.97392200	-0.14552700
H	-0.00139700	4.57844400	-1.71552900
H	-0.58358600	5.99327400	-0.82348100
C	-1.46603200	-3.27679400	-1.63075900
C	-3.81046000	-2.72708100	-1.54809900
C	-3.43331000	6.04705200	1.27397300
C	-3.86140300	3.79136300	1.99314400
C	-1.18223200	3.53145100	3.69092000
C	0.44267100	2.97911200	2.00900600
C	-1.75804200	-4.58841400	-1.30018800
H	-0.43607900	-2.97756300	-1.80410900
C	-4.07239700	-4.05575900	-1.21972400
H	-4.62302200	-2.02187800	-1.64148800
C	-4.79013500	6.34462700	1.36275200
H	-2.73336100	6.81776400	0.95675400
C	-5.21848700	4.08904400	2.08819900
H	-3.50286300	2.79584300	2.22758700
C	-0.44976500	2.81311000	4.63435300
H	-2.09567900	4.03394100	3.99325600
C	1.19234600	2.29215300	2.95982800
H	0.80199300	3.02437900	0.98251800
C	-0.62756300	-5.54629900	-1.06480800
C	-3.06846200	-5.00174300	-1.07418800
C	-5.49462100	-4.42803800	-0.91361800
C	-5.68788200	5.36103300	1.76878100
H	-5.14256600	7.34224800	1.11868100
H	-5.91339500	3.31799100	2.40679500
C	0.74307900	2.19296200	4.27546700

H	-0.81056500	2.75219300	5.65680000
H	2.13551000	1.83959800	2.66881600
F	0.43334800	-5.28103500	-1.83785400
F	-0.19934200	-5.48030200	0.21362800
F	-0.98786300	-6.81666500	-1.28315100
H	-3.29516700	-6.02781700	-0.80786000
F	-6.36112800	-3.84593500	-1.75127400
F	-5.83660300	-4.03775400	0.32989800
F	-5.69536600	-5.75145100	-0.97482300
H	-6.74797300	5.58507300	1.83954700
H	1.32590800	1.64963500	5.01336300
C	3.18471400	1.39113200	0.07963600
C	2.26082700	0.89727600	-0.84928000
C	1.83831200	1.71644000	-1.90687600
C	2.31072300	3.01554700	-2.02261000
C	3.20287000	3.51824600	-1.07151000
C	3.63976300	2.70531100	-0.03005900
H	3.61335200	0.74510200	0.84729900
H	1.18444200	1.30312600	-2.66899800
H	2.00571600	3.63137200	-2.86388400
H	4.36031700	3.07943800	0.69065800
C	1.71692300	-0.48859900	-0.78678600
O	0.84087200	-0.84871500	-1.55778200
C	2.23321700	-1.48326800	0.26061400
S	1.44622700	-0.96432200	1.84694700
C	-0.25871100	-1.47921500	1.73052800
C	-1.18200300	-0.46311700	1.48718800
C	-0.64002700	-2.81545300	1.86993800
C	-2.53017600	-0.80035600	1.37936600
H	-0.87462800	0.57581900	1.39123200
C	-1.99020600	-3.12982400	1.77250700
H	0.09710900	-3.58964800	2.05695100
C	-2.93092400	-2.12603800	1.53085600
H	-3.24969600	-0.01722300	1.16440700
H	-2.30441300	-4.16376500	1.87466100
H	-3.98152800	-2.38667400	1.44331200
C	2.16452300	-2.04346900	3.09490800
C	1.42026000	-2.25888700	4.25597500
C	3.46466100	-2.51981600	2.94520900
C	1.98457100	-3.01409000	5.27772100
H	0.41797000	-1.85522500	4.36192300
C	4.00531700	-3.27519400	3.98542000
H	4.07001700	-2.29471700	2.06875400

C	3.27312400	-3.52767700	5.14055900
H	1.41377700	-3.19819500	6.18201800
H	5.01520100	-3.65812300	3.88267800
H	3.70885400	-4.11737600	5.94079400
C	1.96592400	-2.92311900	-0.11770200
H	0.90187000	-3.12343900	-0.24173600
H	2.47675100	-3.10683200	-1.06715400
H	2.37545200	-3.60942400	0.62833600
H	3.31011000	-1.29144600	0.45702100
H	3.57700500	4.53369500	-1.16220100
C	7.38428700	0.92651900	-4.10753200
C	8.26118400	1.61383500	-3.27084900
C	8.13078900	1.54705900	-1.87603900
C	7.10704400	0.77173300	-1.37151000
C	6.23020700	0.08623600	-2.20770300
C	6.34511300	0.14536300	-3.58159600
H	7.50958100	0.99657000	-5.18374700
H	9.05663500	2.21029400	-3.70716100
H	8.80716100	2.08095000	-1.21564400
H	5.65717300	-0.39406400	-4.22520600
C	6.69249800	0.50299000	0.04348500
C	5.24831700	-0.64065000	-1.34308300
O	4.28923100	-1.30952000	-1.70286000
O	7.16438200	0.95577800	1.06188800
N	5.61285900	-0.37957200	-0.03988500
O	4.96436300	-0.83417500	1.03336000
S	-4.28396000	0.42477100	-1.626510000

IM2A′

M06-2X/BSI SCF energy in *m*-xylene: -4230.595145 a.u.
M06-2X/BSII SCF energy in *m*-xylene: -4231.675914 a.u.
M06-2X/BSII free energy in *m*-xylene: -4230.775748 a.u.

C	2.84119500	-3.49262900	-2.78812300
C	1.76385000	-2.65078900	-2.00119000
C	4.07730000	-3.84307200	-1.94874000
N	1.74951300	-1.19744900	-2.20324500
C	1.73677100	-2.95301800	-0.48722100
H	0.80113500	-2.94119900	-2.43323400
C	3.22581800	-2.74174800	-4.06779700
C	2.21240600	-4.82783600	-3.22209900
H	4.78596200	-4.39906400	-2.57377600

H	3.80586700	-4.48947200	-1.10559600
H	4.57646600	-2.95841500	-1.55744700
C	2.65103100	-0.21323000	-1.92138400
H	0.79701400	-0.85721600	-2.29254700
N	1.21319700	-4.13711800	-0.04534300
O	2.23996100	-2.16003100	0.29425300
H	3.76577200	-1.81798300	-3.85436700
H	2.33857500	-2.49709000	-4.66400500
H	3.87404100	-3.37959100	-4.67861300
H	2.90803700	-5.34666400	-3.89007600
H	1.27224000	-4.68528700	-3.77036100
H	2.03190700	-5.49315300	-2.37419500
N	2.03715200	1.01509800	-1.97380900
C	1.46775800	-4.51146700	1.36063300
C	0.22556400	-4.96261400	-0.72414200
C	2.48599500	2.31562800	-1.72560400
H	1.02506000	0.98679000	-2.06105400
C	2.95327500	-4.77044700	1.57746400
C	0.75160800	-3.61440700	2.36444800
H	0.98752400	-5.49306000	1.45672200
H	-0.70631200	-4.97417200	-0.14583800
H	0.00041200	-4.57807600	-1.71587900
H	0.58276900	-5.99331500	-0.82455800
C	1.46659500	3.27707500	-1.63020500
C	3.81091800	2.72684000	-1.54822400
C	3.43274300	-6.04775400	1.27310600
C	3.86088300	-3.79222900	1.99274200
C	1.18173300	-3.53304500	3.69063800
C	-0.44287400	-2.97943700	2.00885700
C	1.75897300	4.58858200	-1.29947000
H	0.43650600	2.97816700	-1.80326000
C	4.07322000	4.05537700	-1.21962700
H	4.62327400	2.02146100	-1.64200300
C	4.78956300	-6.34537200	1.36181000
H	2.73278400	-6.81839800	0.95574800
C	5.21796500	-4.08993800	2.08769000
H	3.50234500	-2.79677500	2.22746700
C	0.44945900	-2.81490600	4.63434600
H	2.09500500	-4.03598700	3.99275600
C	-1.19236400	-2.29262500	2.95995400
H	-0.80215000	-3.02406700	0.98232500
C	0.62870700	5.54665900	-1.06383100
C	3.06951600	5.00156200	-1.07365300

C	5.49556300	4.42735500	-0.91368200
C	5.68733900	-5.36186800	1.76799400
H	5.14196700	-7.34295700	1.11755000
H	5.91288700	-3.31895400	2.40641800
C	-0.74315800	-2.19418200	4.27565300
H	0.81018100	-2.75458000	5.65685500
H	-2.13533400	-1.83957700	2.66907700
F	-0.43229600	5.28172300	-1.83686000
F	0.20054300	5.48055800	0.21460900
F	0.98925900	6.81698900	-1.28200600
H	3.29652100	6.02754000	-0.80720100
F	6.36189500	3.84460600	-1.75105200
F	5.83740200	4.03755600	0.33002100
F	5.69671200	5.75068700	-0.97547900
H	6.74743100	-5.58593500	1.83866200
H	-1.32583100	-1.65098900	5.01376900
C	-3.18488300	-1.39070700	0.07936400
C	-2.26106300	-0.89676600	-0.84957900
C	-1.83863700	-1.71585100	-1.90726900
C	-2.31098000	-3.01497700	-2.02304200
C	-3.20294600	-3.51780300	-1.07183000
C	-3.63980000	-2.70493000	-0.03031900
H	-3.61354400	-0.74477300	0.84710500
H	-1.18490700	-1.30244100	-2.66946200
H	-2.00604400	-3.63071800	-2.86440300
H	-4.36024800	-3.07911900	0.69046600
C	-1.71695400	0.48898100	-0.78683100
O	-0.84076400	0.84911200	-1.55764100
C	-2.23316300	1.48346200	0.26078900
S	-1.44595700	0.96416500	1.84693900
C	0.25889400	1.47928300	1.73053900
C	1.18232200	0.46333400	1.48706100
C	0.64003500	2.81554500	1.87016600
C	2.53046200	0.80075000	1.37938700
H	0.87507600	-0.57561900	1.39090500
C	1.99018100	3.13009700	1.77280700
H	-0.09722600	3.58958800	2.05731900
C	2.93103400	2.12646000	1.53108100
H	3.25009500	0.01773200	1.16437800
H	2.30426000	4.16406200	1.87511500
H	3.98161000	2.38724400	1.44362700
C	-2.16423800	2.04295200	3.09521200
C	-1.42000800	2.25795500	4.25637700

C	-3.46434300	2.51940600	2.94562200
C	-1.98436800	3.01277600	5.27838000
H	-0.41771000	1.85427400	4.36220900
C	-4.00507000	3.27434900	3.98611300
H	-4.06966000	2.29478200	2.06901800
C	-3.27294000	3.52637000	5.14138500
H	-1.41360600	3.19657800	6.18276100
H	-5.01496100	3.65728300	3.88345600
H	-3.70872800	4.11571700	5.94184800
C	-1.96589500	2.92338900	-0.11728600
H	-0.90185500	3.12366800	-0.24144700
H	-2.47682200	3.10722300	-1.06665400
H	-2.37532800	3.60957200	0.62891000
H	-3.31001800	1.29166700	0.45729600
H	-3.57697400	-4.53329400	-1.16247400
C	-7.38502000	-0.92384800	-4.10781300
C	-8.26175300	-1.61160500	-3.27131600
C	-8.13093000	-1.54576400	-1.87650400
C	-7.10692100	-0.77091500	-1.37177600
C	-6.23024600	-0.08498100	-2.20777900
C	-6.34558500	-0.14317700	-3.58167700
H	-7.51067800	-0.99312400	-5.18403300
H	-9.05744500	-2.20763000	-3.70778400
H	-8.80717800	-2.08001100	-1.21627100
H	-5.65780300	0.39665600	-4.22511600
C	-6.69224000	-0.50272400	0.04328000
C	-5.24830200	0.64160200	-1.34296100
O	-4.28923700	1.31059100	-1.70258400
O	-7.16387700	-0.95601000	1.06157400
N	-5.61285000	0.38019600	-0.03985200
O	-4.96450600	0.83467500	1.03351100
S	4.28347700	-0.42522300	-1.62580800

IM2B

M06-2X/BSI SCF energy in *m*-xylene: -4230.624518 a.u.
M06-2X/BSII SCF energy in *m*-xylene: -4231.700061 a.u.
M06-2X/BSII free energy in *m*-xylene: -4230.804092 a.u.

C	2.59640700	0.99424900	-3.04344600
C	2.11172800	0.47417900	-1.64284800
C	3.40025200	2.29938600	-2.98685900
N	0.76214800	0.80576700	-1.18338100

C	3.14938500	0.79249700	-0.55221800
H	2.02498500	-0.60614600	-1.76605900
C	1.38728400	1.14310800	-3.96992900
C	3.52578900	-0.08190900	-3.62914600
H	3.62616200	2.62126600	-4.01104900
H	4.35525200	2.15365700	-2.46969000
H	2.85257700	3.09312700	-2.47976800
C	0.14238900	1.96796400	-0.83898700
H	0.33491100	-0.02032500	-0.75973300
N	4.20581000	-0.06967300	-0.36769400
O	3.08474600	1.84569000	0.05643700
H	0.76403500	1.98590500	-3.65850200
H	0.77116900	0.23686300	-3.97421000
H	1.73163200	1.33187200	-4.99405100
H	3.86138500	0.22506100	-4.62595300
H	3.01927200	-1.05088600	-3.72861400
H	4.42073600	-0.21471900	-3.01127200
N	-0.92582600	1.68885200	-0.02279300
C	5.38863800	0.41490700	0.36340100
C	4.22176400	-1.49170200	-0.66716800
C	-2.03415300	2.42128800	0.38768700
H	-1.04607200	0.67794100	0.18059000
C	6.01605200	1.59766000	-0.36648700
C	5.19229200	0.61586400	1.86126300
H	6.11241000	-0.40432800	0.26419900
H	4.37222900	-2.05744900	0.26041100
H	3.27791600	-1.82015400	-1.09303500
H	5.03219700	-1.73672200	-1.36476300
C	-2.99351400	1.67476800	1.09655500
C	-2.29053000	3.77900900	0.15058100
C	6.84331700	1.33064900	-1.46073600
C	5.74814700	2.92503100	-0.02820300
C	6.28153800	1.05796500	2.61917000
C	3.99612000	0.31698000	2.51143200
C	-4.17546100	2.26021200	1.51938300
H	-2.81698000	0.61510300	1.28116600
C	-3.49196300	4.33340500	0.58334600
H	-1.57029500	4.38532600	-0.38068900
C	7.37856100	2.36602100	-2.22192800
H	7.06333300	0.29728000	-1.72332300
C	6.28600600	3.96228600	-0.78548800
H	5.09630900	3.13834500	0.81139500
C	6.17599100	1.21115300	3.99741400

H	7.21887300	1.29278600	2.11996900
C	3.88816400	0.47606400	3.89316500
H	3.14388300	-0.02886500	1.93355300
C	-5.18782800	1.38082800	2.18956200
C	-4.45559400	3.59839800	1.26220000
C	-3.77783000	5.75470200	0.19883000
C	7.09639700	3.68777100	-1.88525600
H	8.01509300	2.14047300	-3.07243100
H	6.05894700	4.99061100	-0.52118000
C	4.97353000	0.92332800	4.64032800
H	7.03180900	1.55917100	4.56847000
H	2.94430400	0.25699100	4.38434600
F	-5.65600800	0.43911200	1.34118300
F	-4.67951100	0.72350900	3.23774200
F	-6.25278200	2.07086800	2.62505900
H	-5.38559100	4.05033800	1.58572800
F	-4.16371500	5.84387100	-1.09057600
F	-2.70404400	6.54353000	0.32845700
F	-4.76446700	6.29169800	0.93292600
H	7.50798200	4.50086200	-2.47589100
H	4.88277200	1.05202700	5.71482300
C	-4.12944300	0.20190000	-1.64985900
C	-2.91018200	0.16613700	-2.33509200
C	-2.41662900	1.32907600	-2.94401500
C	-3.13978900	2.51057500	-2.86928200
C	-4.37403700	2.53284300	-2.21517400
C	-4.86858600	1.38139100	-1.60995900
H	-4.51162400	-0.67661300	-1.13781000
H	-1.45294500	1.29227600	-3.44349900
H	-2.73620700	3.41980300	-3.30349000
H	-5.81100900	1.40609500	-1.07315700
C	-2.09315900	-1.06406200	-2.45119600
O	-1.12526100	-1.13955300	-3.18627400
C	-2.50640700	-2.31201100	-1.66030700
S	-0.88889300	-3.15675800	-1.33359500
C	-1.11294600	-4.03194400	0.20883400
C	-2.34388700	-4.19517900	0.83691000
C	0.06792800	-4.50816000	0.78131900
C	-2.38403100	-4.84846700	2.06777900
H	-3.26020400	-3.81041800	0.40371700
C	0.00966000	-5.16432200	2.00577600
H	1.02321500	-4.34033000	0.29139200
C	-1.21525700	-5.33068400	2.65006800

H	-3.33613000	-4.96301700	2.57493200
H	0.92513200	-5.52137400	2.46588800
H	-1.25622900	-5.82467200	3.61549100
C	-0.83126400	-4.49543700	-2.53446800
C	-0.18619100	-4.19081100	-3.73294600
C	-1.36951000	-5.75800600	-2.29338100
C	-0.08731700	-5.17517700	-4.71218300
H	0.22054000	-3.19740300	-3.89605400
C	-1.25893100	-6.73185400	-3.28179200
H	-1.85681700	-5.98500100	-1.34996400
C	-0.62117300	-6.44168400	-4.48648000
H	0.41335800	-4.95057600	-5.64817500
H	-1.67202800	-7.72004300	-3.10762800
H	-0.53632100	-7.20779600	-5.25062200
H	-4.93125700	3.46245100	-2.14940300
C	0.58695100	-2.83381400	5.65760100
C	1.85006700	-2.84083400	5.06863000
C	2.03077000	-2.43600400	3.73773100
C	0.91002500	-2.02370500	3.04500500
C	-0.35360500	-2.01850100	3.63377100
C	-0.54470900	-2.42118600	4.93950300
H	0.47876800	-3.15321500	6.68949000
H	2.70862300	-3.16225800	5.64988900
H	3.01213500	-2.43598400	3.27287300
H	-1.53333300	-2.41694700	5.38789000
C	0.75300900	-1.59059700	1.62242500
C	-1.34260800	-1.57634700	2.60017100
O	-2.54582000	-1.46854500	2.68444700
O	1.59571900	-1.51727200	0.74284000
N	-0.58576600	-1.31406600	1.46021800
O	-1.13114000	-1.01047500	0.27413500
S	0.53297700	3.50533400	-1.39121900
C	-3.50730800	-3.15661200	-2.43781200
H	-3.13428200	-3.40338800	-3.43532800
H	-3.74802000	-4.08526600	-1.91190800
H	-4.43128200	-2.58139200	-2.55382300
H	-2.84235500	-2.03127800	-0.66153300

IM2B′

M06-2X/BSI SCF energy in *m*-xylene: -4230.608385 a.u.

M06-2X/BSII SCF energy in *m*-xylene: -4231.68288 a.u.

M06-2X/BSII free energy in *m*-xylene: -4230.785754 a.u.

C	3.11756900	0.99798300	-3.00842100
C	2.43564900	0.36976300	-1.73909000
C	4.19268500	2.04411500	-2.69019800
N	1.18602100	0.94173500	-1.23593600
C	3.45039100	0.22855600	-0.59237800
H	2.09234300	-0.61410700	-2.06052900
C	2.03080500	1.58254900	-3.91238900
C	3.81020200	-0.14019200	-3.77667400
H	4.58755500	2.44584900	-3.63156400
H	5.03206600	1.59506700	-2.14592800
H	3.79583000	2.86497500	-2.09401600
C	0.85408300	2.13666800	-0.67684300
H	0.50153700	0.20202000	-1.07201300
N	4.31270200	-0.84469700	-0.59577900
O	3.54761500	1.10460500	0.24827800
H	1.54443400	2.44066300	-3.43990600
H	1.27349200	0.82194400	-4.13511200
H	2.47624700	1.91490600	-4.85771600
H	4.22563400	0.25191900	-4.71188300
H	3.11093500	-0.94613200	-4.03259300
H	4.64296500	-0.56228400	-3.20460400
N	-0.28421000	1.99528000	0.07456900
C	5.46910900	-0.80626800	0.31415800
C	4.06041800	-2.14581700	-1.19496700
C	-1.20650100	2.90502500	0.57956700
H	-0.63161900	1.01984400	0.16081200
C	6.39154300	0.36163500	-0.02048300
C	5.11413000	-0.94363200	1.79104900
H	6.03692000	-1.70880300	0.05451300
H	3.98778300	-2.90559300	-0.40604600
H	3.12500200	-2.15914500	-1.74681500
H	4.87603100	-2.42614700	-1.87198900
C	-2.22149500	2.32955900	1.36336100
C	-1.26866800	4.27961100	0.31622200
C	7.30969700	0.20005700	-1.06133600
C	6.32652800	1.59059800	0.63922000
C	6.13192000	-0.87207500	2.74744300
C	3.81369500	-1.20687800	2.21591600
C	-3.28554000	3.09257400	1.81412400
H	-2.18611600	1.26369800	1.58313300
C	-2.34953400	5.01879400	0.78953700
H	-0.49929900	4.75678500	-0.27502900

C	8.14073100	1.24756100	-1.45065600
H	7.37129900	-0.75724300	-1.57618100
C	7.15856800	2.63779500	0.25375600
H	5.60345500	1.72766000	1.43474800
C	5.85083900	-1.04755700	4.09872400
H	7.15164400	-0.66523100	2.43152200
C	3.52854700	-1.38117500	3.56861800
H	3.01554600	-1.26454900	1.48209500
C	-4.38397300	2.37759200	2.53988300
C	-3.38025900	4.45121600	1.52873100
C	-2.44277100	6.45589200	0.37065800
C	8.06430800	2.47206800	-0.79212000
H	8.84903800	1.10552400	-2.26139300
H	7.08891400	3.59213600	0.76686500
C	4.54500100	-1.29947300	4.51534900
H	6.65261500	-0.98002400	4.82834900
H	2.50471400	-1.57017900	3.88094300
F	-5.01753900	1.50592700	1.71895700
F	-3.93460600	1.65440600	3.57152000
F	-5.32179000	3.21344300	3.00792400
H	-4.21952600	5.04308800	1.87389200
F	-2.86071400	6.56471900	-0.90827700
F	-1.26293900	7.08488100	0.43789200
F	-3.31481400	7.14998000	1.11762600
H	8.70765900	3.29415800	-1.09122700
H	4.32184600	-1.42628900	5.57047100
C	-3.34873900	0.87876900	-1.65168100
C	-2.21718600	0.66880100	-2.44651200
C	-1.57891900	1.76640100	-3.03700500
C	-2.08031400	3.04956600	-2.85701400
C	-3.22804200	3.24814100	-2.08959400
C	-3.85539800	2.16322300	-1.48106300
H	-3.81981600	0.05387100	-1.12900100
H	-0.68503400	1.59825300	-3.62776900
H	-1.56944200	3.89587800	-3.30562000
H	-4.72194600	2.31752500	-0.84604500
C	-1.62510900	-0.67178100	-2.69969100
O	-0.47485800	-0.81856200	-3.05443800
C	-2.49524300	-1.96169600	-2.70918400
S	-2.20932900	-2.87865500	-1.11910900
C	-3.79791500	-2.87586900	-0.27495800
C	-4.01306500	-1.82808300	0.62047000
C	-4.74763400	-3.87521100	-0.48634000

C	-5.21954400	-1.78577100	1.31688100
H	-3.25214400	-1.06590000	0.76811300
C	-5.94434100	-3.81361000	0.21987000
H	-4.56001500	-4.69495400	-1.17184700
C	-6.17976300	-2.77347400	1.11862000
H	-5.39172000	-0.97652800	2.01790100
H	-6.69165800	-4.58582200	0.07010000
H	-7.11472100	-2.73793400	1.66863700
C	-2.00508500	-4.58250200	-1.63274600
C	-1.23012800	-5.34976800	-0.76299700
C	-2.53684300	-5.12558100	-2.80160300
C	-1.00580000	-6.69066000	-1.06033300
H	-0.79892900	-4.90377100	0.12969700
C	-2.29478400	-6.46494300	-3.09140100
H	-3.13011200	-4.52617600	-3.48334100
C	-1.53637700	-7.24610200	-2.22152900
H	-0.40328700	-7.29429000	-0.39001000
H	-2.70172500	-6.89584600	-4.00010700
H	-1.35077900	-8.28937100	-2.45555700
C	-3.95370800	-1.82972300	-3.12258400
H	-4.59051200	-1.39944700	-2.35009900
H	-3.99892000	-1.17769500	-4.00079100
H	-4.37621200	-2.79901500	-3.40056900
H	-1.95732300	-2.59594600	-3.42024600
H	-3.60984900	4.25313700	-1.93690800
C	0.78573900	-3.96685500	4.45662600
C	1.66568800	-4.41687000	3.47356300
C	1.64143600	-3.87909500	2.17923100
C	0.70828700	-2.89440700	1.91950200
C	-0.17801700	-2.45204000	2.90051100
C	-0.15864800	-2.96739200	4.18125200
H	0.83479900	-4.39695700	5.45200400
H	2.38882000	-5.18855100	3.71815300
H	2.33398800	-4.21500000	1.41340400
H	-0.84923600	-2.61105000	4.93889000
C	0.42155600	-2.12935200	0.66357600
C	-1.07096600	-1.42175400	2.28229400
O	-1.97791000	-0.79907100	2.79138200
O	0.99441900	-2.18204300	-0.41034900
N	-0.66295700	-1.32344900	0.95363500
O	-1.26485900	-0.54441400	0.04119100
S	1.63804200	3.60373100	-0.91514400

(*S*)-**TS2A**

M06-2X/BSI SCF energy in *m*-xylene: -4230.57732 a.u.
M06-2X/BSII SCF energy in *m*-xylene: -4231.654625 a.u.
M06-2X/BSII free energy in *m*-xylene: -4230.759677 a.u.

C	4.28188600	-0.59050300	-3.17607000
C	3.07114800	-0.78233700	-2.19352300
C	5.39356100	0.31884600	-2.64140400
N	1.90196000	0.09085100	-2.31298000
C	3.53988100	-0.86183300	-0.73085900
H	2.64970300	-1.74410400	-2.49220400
C	3.74561300	-0.07659800	-4.51473400
C	4.90145300	-1.97997900	-3.40257200
H	6.16834300	0.42249800	-3.41074100
H	5.86782800	-0.11430600	-1.75415300
H	5.02147500	1.30931900	-2.38397400
C	1.69287800	1.42019200	-2.10745200
H	1.06935600	-0.46600600	-2.15302800
N	3.95357600	-2.08774400	-0.26890800
O	3.63111200	0.14900700	-0.05263900
H	3.36141800	0.94234300	-4.42504500
H	2.94140500	-0.71912800	-4.89314300
H	4.55350400	-0.07650200	-5.25551300
H	5.72162500	-1.90133100	-4.12437700
H	4.17098500	-2.69324800	-3.80431400
H	5.31801800	-2.38605600	-2.47456000
N	0.37596900	1.62808900	-1.76656300
C	4.86273100	-2.15791200	0.88620900
C	3.37428700	-3.36080800	-0.68555300
C	-0.38859900	2.76837400	-1.52830300
H	-0.10415500	0.78752900	-1.44760800
C	6.12975700	-1.34988300	0.63410900
C	4.19524500	-1.91021700	2.23238600
H	5.18565200	-3.20698700	0.89797700
H	3.13184900	-3.94534000	0.20902200
H	2.43666600	-3.21141500	-1.22155000
H	4.05944800	-3.94459500	-1.31239300
C	-1.70858500	2.50898200	-1.11667400
C	0.03458800	4.09820800	-1.61902500
C	7.14928500	-1.94117300	-0.11628500
C	6.28861000	-0.03552500	1.07646200
C	4.98343800	-1.88025000	3.38771000

C	2.81264200	-1.80025800	2.36005500
C	-2.55187500	3.54460500	-0.75778800
H	-2.06508000	1.48169200	-1.09349400
C	-0.83965600	5.11806600	-1.25004600
H	1.04083200	4.33214500	-1.93512400
C	8.30191500	-1.23015200	-0.43924900
H	7.03407300	-2.96858800	-0.45682300
C	7.44230200	0.67611900	0.75813100
H	5.49736000	0.43187800	1.65201100
C	4.40279000	-1.72747000	4.64211500
H	6.06322600	-1.96997100	3.30127500
C	2.22921400	-1.64618100	3.61725100
H	2.18987400	-1.83427700	1.46883000
C	-3.92240100	3.25271600	-0.22721000
C	-2.12905800	4.87120700	-0.80600000
C	-0.30010500	6.51711900	-1.19749000
C	8.44786200	0.08463200	-0.00372200
H	9.08405700	-1.70285900	-1.02560900
H	7.54984300	1.70155500	1.09828600
C	3.01996600	-1.60650400	4.76128100
H	5.03151300	-1.70059900	5.52696200
H	1.15044900	-1.54587000	3.70296100
F	-4.27695400	1.97228200	-0.39667400
F	-3.99352200	3.50241700	1.09881600
F	-4.85991800	4.01534400	-0.80520800
H	-2.78796400	5.68078000	-0.51016700
F	0.56549300	6.76840800	-2.18620000
F	0.35886700	6.73093800	-0.04037600
F	-1.27152600	7.43893300	-1.26399700
H	9.34298500	0.64591900	-0.25396300
H	2.56346200	-1.47990500	5.73811800
C	0.02337200	-3.63064600	1.38103000
C	-0.43542500	-3.14353500	0.15483500
C	-0.39097700	-3.95022000	-0.98385900
C	0.10709400	-5.24675100	-0.88757000
C	0.57686200	-5.73155600	0.33299600
C	0.53744100	-4.92263300	1.46703200
H	0.00139100	-2.99972300	2.26677300
H	-0.78707200	-3.56641900	-1.91788600
H	0.12202900	-5.88087000	-1.76857500
H	0.90734700	-5.29213400	2.41845400
C	-0.86421500	-1.71301400	0.02514700
O	-0.14107300	-0.91004000	-0.55287700

C	-2.11192900	-1.24829100	0.70180200
S	-1.18485500	-0.23634100	2.51122700
C	-0.39686600	1.29744200	2.01530500
C	0.89984200	1.18045600	1.50746000
C	-0.99295500	2.54729600	2.18976800
C	1.60831000	2.32980100	1.17339700
H	1.36869400	0.21187300	1.36940500
C	-0.26939900	3.68859300	1.85259100
H	-1.99783200	2.64194600	2.58520800
C	1.02535300	3.58372000	1.34808700
H	2.60591400	2.22245000	0.75969200
H	-0.72764700	4.66508500	1.97832900
H	1.57300700	4.48024900	1.07422000
C	-2.65591800	0.26855400	3.38261700
C	-2.68253100	0.09568200	4.76614000
C	-3.75396700	0.79959300	2.70200400
C	-3.82163300	0.46640200	5.47646100
H	-1.82078700	-0.31938200	5.27968400
C	-4.88183300	1.17561900	3.42397000
H	-3.74071400	0.92400500	1.62344900
C	-4.91636000	1.00767500	4.80776400
H	-3.84865300	0.33403300	6.55311000
H	-5.73105100	1.59987100	2.89818000
H	-5.80079200	1.29804800	5.36587600
H	0.96872600	-6.74182700	0.40225100
C	-7.45461500	-4.72650900	-2.63464100
C	-8.06200200	-3.67879400	-1.94238300
C	-7.30278700	-2.63758900	-1.39458700
C	-5.93376000	-2.69188700	-1.57028900
C	-5.32679700	-3.73784400	-2.26285600
C	-6.06532800	-4.77072100	-2.80617100
H	-8.07063500	-5.51927800	-3.04738000
H	-9.14132500	-3.67142800	-1.82776500
H	-7.76747800	-1.81766200	-0.85649300
H	-5.58289100	-5.58122400	-3.34288700
C	-4.86241900	-1.75877500	-1.10983100
C	-3.84865300	-3.50328800	-2.27422400
O	-2.98088800	-4.16523600	-2.79428100
O	-4.95452900	-0.74307700	-0.45383300
N	-3.66415500	-2.31897200	-1.55205100
O	-2.49553800	-1.70945300	-1.39435600
S	2.81985800	2.64053800	-2.31287000
H	-2.51613300	-0.31913100	0.32355100

C	-3.01390500	-2.22372300	1.39651700
H	-3.89897200	-1.72870900	1.79622300
H	-3.33809000	-2.98141000	0.67358600
H	-2.49955100	-2.75939200	2.20019700

(*R*)-**TS2A**

M06-2X/BSI SCF energy in *m*-xylene: -4230.587005 a.u.

M06-2X/BSII SCF energy in *m*-xylene: -4231.664421 a.u.

M06-2X/BSII free energy in *m*-xylene: -4230.767181 a.u.

C	2.65994600	-3.45753100	-2.85223000
C	1.65206100	-2.62761600	-1.96712300
C	3.93900800	-3.86661100	-2.11097300
N	1.65122000	-1.16836700	-2.09949800
C	1.68843500	-3.00524200	-0.46986600
H	0.66088000	-2.87729900	-2.35815700
C	2.98257200	-2.66528900	-4.12390300
C	1.97491300	-4.76289500	-3.29296300
H	4.56907900	-4.45777400	-2.78624600
H	3.70349300	-4.49686900	-1.24543500
H	4.50900000	-3.00710900	-1.76372200
C	2.59689100	-0.21437900	-1.87037900
H	0.70111700	-0.80399700	-2.09643100
N	1.16952500	-4.21495400	-0.07763600
O	2.22027300	-2.25940400	0.33605400
H	3.55297400	-1.76094800	-3.90649900
H	2.06819200	-2.38128900	-4.65817400
H	3.58350200	-3.29006700	-4.79396400
H	2.61448000	-5.26952400	-4.02346600
H	1.00494900	-4.58190200	-3.77359700
H	1.83479000	-5.45536500	-2.45883800
N	2.01960500	1.03098100	-1.90760000
C	1.41273100	-4.64636600	1.30897800
C	0.09040900	-4.94113000	-0.73439200
C	2.49809700	2.31229900	-1.62994100
H	1.00474400	1.02596300	-1.97378200
C	2.90278800	-4.79172900	1.59574700
C	0.59293600	-3.87744500	2.34073400
H	1.01822400	-5.67076200	1.33195300
H	-0.80692400	-4.92178400	-0.10326600
H	-0.16534800	-4.49808000	-1.69336600
H	0.37258700	-5.98633200	-0.90077100

C	1.49634200	3.28872100	-1.50057000
C	3.83171200	2.69499200	-1.45295400
C	3.53596700	-5.95441200	1.15011700
C	3.66093400	-3.81743200	2.24685800
C	0.82387900	-4.09614100	3.70269300
C	-0.47791400	-3.06271200	1.96637100
C	1.81419300	4.58575800	-1.13979300
H	0.46075900	3.00954600	-1.67453100
C	4.11974900	4.01018600	-1.09368000
H	4.62880900	1.97559600	-1.57080200
C	4.90256600	-6.14212100	1.33780600
H	2.95107300	-6.71867700	0.64113600
C	5.02579400	-4.00644700	2.44446900
H	3.18014400	-2.90528600	2.58279000
C	0.01447800	-3.50506900	4.66803200
H	1.64873800	-4.73330100	4.00964200
C	-1.30153500	-2.48856200	2.93329700
H	-0.68154100	-2.87304300	0.91383700
C	0.70052800	5.55427000	-0.87341500
C	3.13373000	4.97002300	-0.91547700
C	5.54992900	4.35280400	-0.79156800
C	5.65103500	-5.16494000	1.98815200
H	5.37846600	-7.05029400	0.98037400
H	5.60496600	-3.23810100	2.94748100
C	-1.05647900	-2.70028500	4.28666300
H	0.21830200	-3.68115800	5.72004100
H	-2.13980400	-1.87425700	2.62052600
F	-0.36502100	5.33123400	-1.65405800
F	0.26858400	5.45833200	0.40169900
F	1.08060200	6.82557200	-1.05395300
H	3.38093700	5.98523500	-0.62695500
F	6.40243200	3.76069500	-1.63687600
F	5.89222200	3.94986800	0.44793100
F	5.77592500	5.67331500	-0.84547700
H	6.71715100	-5.30501900	2.13933900
H	-1.69708600	-2.24615900	5.03659000
C	-3.30030200	-1.34223100	-0.01871300
C	-2.37801600	-0.85687900	-0.95044100
C	-1.96057500	-1.68853700	-1.99571200
C	-2.45109900	-2.98274900	-2.10610900
C	-3.35975400	-3.46719700	-1.16368800
C	-3.78181400	-2.64726900	-0.12098700
H	-3.65782700	-0.71720600	0.79625900

H	-1.30084300	-1.28142100	-2.75651700
H	-2.15021900	-3.60513100	-2.94406500
H	-4.49513700	-3.01564200	0.60933400
C	-1.83144700	0.53332800	-0.92413600
O	-0.78695400	0.79778500	-1.51921200
C	-2.44422700	1.56171000	-0.03073200
S	-1.44720800	0.93156100	1.88214100
C	0.25059200	1.45292900	1.78297000
C	1.17976200	0.45560700	1.48124000
C	0.64062700	2.77946300	1.97810900
C	2.52798300	0.79194000	1.39677900
H	0.87142200	-0.57607000	1.33088400
C	1.99321800	3.09699800	1.90097300
H	-0.09497100	3.54779200	2.19525700
C	2.93482200	2.10725000	1.61789000
H	3.24455300	0.01600500	1.14546100
H	2.30592100	4.12649400	2.04747400
H	3.98629800	2.36905100	1.54696100
C	-2.17765700	1.98532200	3.13256900
C	-1.48723800	2.19289200	4.32968800
C	-3.46394200	2.49427100	2.95078200
C	-2.08245800	2.94256200	5.33742800
H	-0.49389500	1.77712600	4.46836800
C	-4.04977400	3.23586100	3.97589300
H	-4.02267400	2.33136000	2.03295900
C	-3.36282500	3.46612200	5.16281300
H	-1.54436000	3.11155300	6.26471700
H	-5.04746000	3.63802300	3.83303200
H	-3.82387300	4.04928100	5.95349700
C	-2.00673500	2.98589600	-0.17320000
H	-0.92017300	3.07128700	-0.11739700
H	-2.33319500	3.33851100	-1.15511200
H	-2.46104200	3.61819800	0.59350900
H	-3.41905100	1.36078200	0.38661200
H	-3.74875200	-4.47711800	-1.25119700
C	-8.34151200	-1.92412500	-1.94371000
C	-8.70075500	-1.16050700	-0.83289000
C	-7.87048900	-0.13666900	-0.36051800
C	-6.68797600	0.08405300	-1.03981600
C	-6.32972700	-0.67721200	-2.14991700
C	-7.13936400	-1.69106000	-2.62351900
H	-9.00591300	-2.71134800	-2.28597800
H	-9.63955300	-1.36358500	-0.32722300

H	-8.14167000	0.46193400	0.50309800
H	-6.85085800	-2.28096300	-3.48754100
C	-5.59687000	1.06979000	-0.77615200
C	-4.99870700	-0.19741500	-2.63409700
O	-4.34253100	-0.58356900	-3.56828800
O	-5.51127900	1.87678100	0.13263400
N	-4.64524700	0.84635500	-1.75824100
O	-3.50800400	1.52820300	-1.86888900
S	4.23467900	-0.47290700	-1.64151500

(*S*)-**TS2B**
M06-2X/BSI SCF energy in *m*-xylene: -4230.592707 a.u.
M06-2X/BSII SCF energy in *m*-xylene: -4231.66822 a.u.
M06-2X/BSII free energy in *m*-xylene: -4230.770868 a.u.

C	2.17576400	-2.98762300	-3.34752100
C	1.62833600	-2.37238900	-1.98663200
C	3.70682800	-3.01368600	-3.44312600
N	0.92063300	-1.10863200	-2.12426400
C	2.57928500	-2.22128900	-0.75147600
H	0.83802500	-3.05606000	-1.64846600
C	1.61304800	-2.18196200	-4.52947300
C	1.63605400	-4.41821400	-3.53369400
H	3.99729900	-3.28021900	-4.46642500
H	4.14814900	-3.75664800	-2.77217900
H	4.13333100	-2.03492100	-3.20724400
C	1.47915600	0.12333700	-2.17211700
H	-0.05719300	-1.13809700	-1.83609400
N	3.39492300	-3.26685700	-0.38492300
O	2.59263000	-1.18604100	-0.11294000
H	1.99651100	-1.16038500	-4.53940400
H	0.51966000	-2.14663200	-4.49658800
H	1.91385500	-2.67079900	-5.46346500
H	1.92534800	-4.78749100	-4.52396200
H	0.54040500	-4.43327200	-3.48368300
H	2.02535800	-5.13233900	-2.80571500
N	0.58378300	1.09535800	-1.79992600
S	3.03526800	0.45624500	-2.72062500
C	4.48829500	-2.99894800	0.55968300
C	3.00992600	-4.66227600	-0.49092300
C	0.83810000	2.42001000	-1.44099100
H	-0.36889900	0.77114200	-1.59685800

C	5.31034600	-1.77952100	0.14803500
C	4.05871300	-3.00082800	2.02285800
H	5.15857200	-3.86171400	0.43723400
H	3.09852900	-5.13220800	0.49560700
H	1.96664000	-4.75261800	-0.79400700
H	3.64061100	-5.21801100	-1.19587700
C	-0.20785400	3.33761700	-1.57075900
C	2.03801900	2.83548400	-0.85062600
C	6.16318300	-1.89332100	-0.95004200
C	5.22463300	-0.55516000	0.81118000
C	5.04677300	-3.05962100	3.01109400
C	2.71981300	-2.97756900	2.40969500
C	-0.05337400	4.64408400	-1.11969000
H	-1.14819300	3.01706700	-2.01219000
C	2.17070800	4.14874100	-0.42197600
H	2.84097200	2.12347300	-0.70532900
C	6.91220600	-0.80307500	-1.38712600
H	6.23206200	-2.84394400	-1.47534100
C	5.97452700	0.53504200	0.38168200
H	4.54466100	-0.45224300	1.65068500
C	4.70553800	-3.08920800	4.35896900
H	6.09426600	-3.07471500	2.71709100
C	2.37674400	-3.00977000	3.76177300
H	1.93602000	-2.91686600	1.66147200
C	-1.23140000	5.56757200	-1.18965100
C	1.13581900	5.07266100	-0.54352500
C	3.42281400	4.53234600	0.31018900
C	6.82046400	0.41475000	-0.71934900
H	7.56641000	-0.90653700	-2.24762200
H	5.87948200	1.48900500	0.89221500
C	3.36415700	-3.06586700	4.73996500
H	5.48614000	-3.13472100	5.11283500
H	1.32727400	-2.99538600	4.04393600
F	-1.92078800	5.42158600	-2.33062800
F	-2.10251200	5.32971700	-0.18764600
F	-0.87642900	6.85602600	-1.09232100
H	1.25420800	6.09455600	-0.20154100
F	4.50910100	3.92509500	-0.18226000
F	3.35176200	4.19108400	1.61433800
F	3.64526400	5.85526300	0.27474700
H	7.39797100	1.26925500	-1.05871300
H	3.09493100	-3.09199500	5.79171300
C	-1.17771300	1.42909600	1.21934700

C	-0.37573900	0.28516900	1.33514600
C	0.92589300	0.39395200	1.84252600
C	1.40397200	1.62747300	2.26631600
C	0.58739600	2.75688700	2.19105400
C	-0.69651500	2.65894400	1.65782800
H	-2.16181500	1.37664400	0.76208400
H	1.54901300	-0.49340600	1.88334900
H	2.41714000	1.71437100	2.64661200
H	-1.31535100	3.54501300	1.55592000
C	-0.87128500	-1.07714400	1.00825300
O	-0.16124400	-2.06700100	1.01883400
C	-2.34340000	-1.27504700	0.70904900
H	-2.99772400	-0.42056700	0.63798300
C	-2.74971200	-2.60307900	0.13494900
H	-3.83103400	-2.67419200	-0.00176200
H	-2.25364300	-2.72372600	-0.83217900
H	-2.40495100	-3.42587400	0.76644700
S	-2.99983200	-1.65748600	2.85398200
C	-2.73940300	-0.11291000	3.71478100
C	-1.48270500	0.02893800	4.30877500
C	-3.67353400	0.91897400	3.75438700
C	-1.15410100	1.22866300	4.92860600
H	-0.76115200	-0.78310200	4.27166700
C	-3.33547700	2.10968500	4.39319200
H	-4.65428400	0.80372300	3.30643200
C	-2.07923000	2.26992800	4.97123400
H	-0.17203800	1.34745600	5.37443300
H	-4.06069600	2.91637000	4.43044600
H	-1.82034900	3.20553700	5.45650000
C	-4.76804600	-1.79168200	2.64196600
C	-5.42545900	-2.75618300	3.40664200
C	-5.46796100	-1.01339800	1.71500600
C	-6.79715600	-2.93608600	3.24948500
H	-4.86827800	-3.35936800	4.11666000
C	-6.83853700	-1.20403900	1.56827000
H	-4.96916600	-0.26319300	1.10729700
C	-7.50240700	-2.16193200	2.33226200
H	-7.31068700	-3.68555500	3.84274400
H	-7.38361300	-0.60147300	0.84873800
H	-8.57091100	-2.30744300	2.20884200
C	-5.95088400	-1.50897800	-5.19445000
C	-6.68511200	-0.59330700	-4.44193900
C	-6.14094500	0.00565600	-3.29836200

C	-4.85120900	-0.34557000	-2.95269600
C	-4.11579100	-1.26002200	-3.70734700
C	-4.64335900	-1.85993100	-4.83338900
H	-6.39998200	-1.95593700	-6.07562400
H	-7.69482500	-0.33920500	-4.74846500
H	-6.70538100	0.72158800	-2.70935700
H	-4.06396600	-2.57046600	-5.41402300
C	-3.99943000	0.10740200	-1.81083600
C	-2.77516000	-1.41710100	-3.06435900
O	-1.85638000	-2.15173000	-3.34799600
O	-4.28207400	0.84025600	-0.88059400
N	-2.78188300	-0.52646400	-1.98902700
O	-1.75075000	-0.37928700	-1.15408800
H	0.96539200	3.71953900	2.52190200

(*R*)-**TS2B**
M06-2X/BSI SCF energy in *m*-xylene: -4230.58563 a.u.
M06-2X/BSII SCF energy in *m*-xylene: -4231.659107 a.u.
M06-2X/BSII free energy in *m*-xylene: -4230.761867 a.u.

C	0.29216300	-1.81396000	3.67463100
C	0.14936500	-1.45207900	2.13454200
C	-0.77322400	-2.79268700	4.18256100
N	-0.17780100	-0.06248100	1.85989400
C	-0.74855800	-2.32610500	1.19779100
H	1.16052600	-1.53902500	1.71421300
C	0.22391800	-0.52807000	4.51362200
C	1.69223100	-2.39799900	3.93772500
H	-0.69724000	-2.87271500	5.27377400
H	-0.63980300	-3.79659500	3.76834400
H	-1.77986100	-2.44427000	3.93302900
C	-1.41516400	0.48687100	1.82290900
H	0.51676200	0.40427700	1.27888700
N	-0.62058400	-3.69705900	1.23131600
O	-1.54264500	-1.80692200	0.43757700
H	-0.75649300	-0.05333600	4.44659200
H	0.98587300	0.18944300	4.19189500
H	0.41432200	-0.78058900	5.56344100
H	1.82588600	-2.55856300	5.01388200
H	2.46360200	-1.69269500	3.60469500
H	1.86282700	-3.35975300	3.44817800
N	-1.42211200	1.63343700	1.06446900

S	-2.74795900	-0.06921900	2.68532100
C	-1.65693600	-4.50655700	0.57739000
C	0.64824700	-4.37140000	1.43600300
C	-2.51100300	2.26351100	0.45910100
H	-0.50653300	1.94416700	0.72053000
C	-3.05692700	-4.06735500	1.00133300
C	-1.46376700	-4.65455200	-0.92821200
H	-1.51798600	-5.50938100	1.00615100
H	0.83264000	-5.05193200	0.59582000
H	1.46762200	-3.65166600	1.44380100
H	0.66861200	-4.95487600	2.36558800
C	-2.37532500	3.61396200	0.12720500
C	-3.66357200	1.57252300	0.06237800
C	-3.46792600	-4.34997900	2.30393500
C	-3.92019900	-3.36727700	0.15849600
C	-2.23338400	-5.59459300	-1.62071800
C	-0.52029400	-3.90821800	-1.63232000
C	-3.37878800	4.26211800	-0.58608600
H	-1.47368800	4.14795800	0.41580600
C	-4.65184100	2.24656600	-0.64011800
H	-3.75916300	0.51501600	0.28015200
C	-4.71296200	-3.93076400	2.76591700
H	-2.79911000	-4.89382400	2.96817600
C	-5.16827600	-2.95342500	0.61291600
H	-3.59949400	-3.12019100	-0.84853000
C	-2.07054800	-5.77815700	-2.98979800
H	-2.97479800	-6.17986900	-1.08090700
C	-0.35656700	-4.09056000	-3.00575000
H	0.08084100	-3.16812200	-1.11343500
C	-3.16637300	5.68981600	-0.99070900
C	-4.53015100	3.59302800	-0.97756100
C	-5.82343300	1.47414800	-1.16907800
C	-5.56654500	-3.22933600	1.91947000
H	-5.01301500	-4.15212600	3.78585600
H	-5.82151200	-2.39158400	-0.04763500
C	-1.12912800	-5.02381100	-3.68961300
H	-2.67891800	-6.51070600	-3.51204800
H	0.37562200	-3.48958200	-3.53936400
F	-2.60624800	6.41504700	-0.01228500
F	-2.34304300	5.78459800	-2.05397500
F	-4.31234000	6.29420700	-1.33533600
H	-5.31380400	4.10394200	-1.52542000
F	-6.13342700	0.42097500	-0.40427700

F	-5.57406300	0.99502400	-2.40613500
F	-6.92282100	2.23663800	-1.27379800
H	-6.53529300	-2.89340900	2.27649100
H	-1.00280800	-5.16389200	-4.75906600
C	-0.22920700	2.00413900	-2.25314300
C	-0.12142400	0.64158900	-1.94420700
C	-1.20327000	-0.21383700	-2.20428500
C	-2.35568200	0.27933200	-2.80092700
C	-2.44538100	1.62998600	-3.13725000
C	-1.38715600	2.49034300	-2.85375300
H	0.55197100	2.69757800	-1.96979000
H	-1.13445600	-1.25502800	-1.90723200
H	-3.19368800	-0.38515700	-2.98666400
H	-1.47622700	3.55173700	-3.06264800
C	1.06690000	0.01456700	-1.30509400
O	1.01723200	-1.11079700	-0.83897800
C	2.42607600	0.70504200	-1.22170700
S	3.44152400	-1.01126600	-2.31003500
C	5.06557800	-0.42107700	-2.75690600
C	5.18143500	0.13015300	-4.03562400
C	6.15397800	-0.44286600	-1.88544600
C	6.39928700	0.66785600	-4.43978000
H	4.32904500	0.13219200	-4.70946200
C	7.36962600	0.08771500	-2.30749600
H	6.06136100	-0.88114600	-0.89737300
C	7.49332400	0.64614300	-3.57815000
H	6.49309600	1.09569200	-5.43254800
H	8.22211800	0.06667500	-1.63627700
H	8.44402900	1.05959800	-3.89880700
C	3.74750200	-2.27567900	-1.08073100
C	3.69675200	-3.59817900	-1.52088600
C	3.99219900	-1.97505600	0.26059500
C	3.91781600	-4.63067600	-0.61170100
H	3.48212800	-3.81929600	-2.56184700
C	4.21792000	-3.01549700	1.15759500
H	3.99803400	-0.95376800	0.62632400
C	4.18353700	-4.34048300	0.72367400
H	3.87725000	-5.66032500	-0.95184000
H	4.41201100	-2.78482600	2.20032800
H	4.35308700	-5.14580500	1.43174500
C	5.10773800	3.73841400	4.35415200
C	4.83545800	4.99304200	3.81016600
C	3.96339600	5.13773700	2.72344400

C	3.38738900	3.98954900	2.21735500
C	3.65893900	2.73500600	2.76269300
C	4.51938800	2.57968500	3.83135100
H	5.78539000	3.66032000	5.19831800
H	5.30468200	5.87252500	4.23940700
H	3.74509800	6.11074400	2.29518700
H	4.71979800	1.59956900	4.25229700
C	2.42458300	3.80836300	1.08696200
C	2.88206000	1.71783100	1.99357500
O	2.86564400	0.50397300	2.11012300
O	1.95164600	4.62630400	0.33431700
N	2.16829000	2.43260400	1.04920900
O	1.28995700	1.87963200	0.20577400
H	-3.35463000	2.01600100	-3.58853400
C	2.92655100	1.86183600	-2.03811400
H	2.53291700	1.84941900	-3.05808400
H	2.61628300	2.79324200	-1.55605100
H	4.01894500	1.85833700	-2.07595100
H	2.94526900	0.46814900	-0.30238200

3a
M06-2X/BSI SCF energy in *m*-xylene: -1010.847635 a.u.
M06-2X/BSII SCF energy in *m*-xylene: -1011.139171 a.u.
M06-2X/BSII free energy in *m*-xylene: -1010.914574 a.u.

C	-1.12441100	0.80956600	1.34532300
C	-2.01581400	0.79160900	0.26801200
C	-2.36296400	2.00174200	-0.34592200
C	-1.82336400	3.20104000	0.09864600
C	-0.93639200	3.20987300	1.17462600
C	-0.59397800	2.01461600	1.79968000
H	-0.84128200	-0.10515200	1.84948300
H	-3.04879100	1.97767500	-1.18576300
H	-2.08976500	4.12983500	-0.39605500
H	0.09027400	2.01479900	2.64237100
C	-2.64837500	-0.44659600	-0.29980900
O	-3.65663300	-0.34783600	-0.96992500
C	-2.10254400	-1.87802700	-0.09257700
H	-0.51487700	4.14687700	1.52593200
C	3.85355000	1.40655900	-0.52577500
C	4.28717700	0.47131100	0.41656100
C	3.49373800	-0.62562000	0.76735400

C	2.26685800	-0.74597400	0.13900100
C	1.83687900	0.18661400	-0.80367700
C	2.61123100	1.27767700	-1.15350600
H	4.49318900	2.24716700	-0.77488700
H	5.25827700	0.59929700	0.88404400
H	3.82468500	-1.35596600	1.49864800
H	2.26265200	1.99916700	-1.88510400
C	1.20835500	-1.78742700	0.30376600
C	0.48758000	-0.21698300	-1.29053400
O	-0.20688500	0.24994300	-2.15415500
O	1.21003400	-2.79427500	0.96521000
N	0.14535400	-1.32051100	-0.48588700
O	-0.89746300	-2.12031900	-0.84884500
H	-2.82244400	-2.49543300	-0.63388100
C	-1.99058300	-2.37864000	1.33948300
H	-1.93778300	-3.47008200	1.33172300
H	-1.09560700	-2.01699000	1.84573500
H	-2.87008300	-2.07575300	1.91675400

SPh_2

M06-2X/BSI SCF energy in *m*-xylene: -861.2515489 a.u.
M06-2X/BSII SCF energy in *m*-xylene: -861.408866 a.u.
M06-2X/BSII free energy in *m*-xylene: -861.261583 a.u.

S	-0.00002900	1.63624700	0.00003800
C	-1.39210000	0.52205400	-0.06309200
C	-2.52251800	0.84045500	0.69121400
C	-1.40100400	-0.60449200	-0.89119200
C	-3.65923800	0.03870700	0.61276200
H	-2.50890400	1.70991200	1.34166400
C	-2.53180000	-1.41202300	-0.94602200
H	-0.52344600	-0.84955200	-1.48225100
C	-3.66585300	-1.09070800	-0.20037100
H	-4.53559100	0.29401600	1.20053900
H	-2.53109300	-2.29015500	-1.58459300
H	-4.54875300	-1.71999400	-0.25176200
C	1.39209500	0.52212200	0.06312100
C	1.40118300	-0.60421600	0.89150800
C	2.52233600	0.84032600	-0.69152400
C	2.53199500	-1.41172100	0.94630600
H	0.52374700	-0.84911900	1.48281300
C	3.65908500	0.03860600	-0.61310700

H	2.50857100	1.70961100	-1.34220000
C	3.66588200	-1.09059200	0.20031800
H	2.53144700	-2.28968400	1.58511000
H	4.53530600	0.29376800	-1.20114400
H	4.54879600	-1.71985900	0.25169000

VIII. Determination of the Stereochemistry

The absolute stereochemistry of product **3a** was determined by X-ray diffraction (shown below). The X-ray data have been deposited at the Cambridge Crystallographic Data Center (CCDC 2379709 for **3a**). The stereochemistry of other products was assumed by analogy.

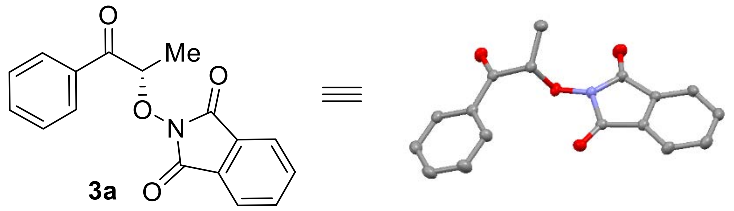

Table S2. Crystal Data and Structure Refinement for 3a.

Identification code	**3a**
Empirical formula	$C_{17}H_{13}NO_4$
Formula weight	295.28
Temperature/K	100.0(2)
Crystal system	orthorhombic
Space group	$P2_12_12_1$
a/Å	7.7844(4)
b/Å	10.1464(5)
c/Å	18.1495(9)
α/°	90
β/°	90
γ/°	90
Volume/Å^3	1433.51(12)
Z	4
ρ_{calc}g/cm^3	1.368
μ/mm^{-1}	0.520
F(000)	616.0
Crystal size/mm^3	0.22 × 0.18 × 0.16
Radiation	GaKα (λ = 1.34138)
2Θ range for data collection/°	8.476 to 146.818
Index ranges	$-9 \le h \le 11$, $-14 \le k \le 14$, $-25 \le l \le 25$
Reflections collected	42754

Independent reflections	4375 [R_{int} = 0.0380, R_{sigma} = 0.0173]
Data/restraints/parameters	4375/0/200
Goodness-of-fit on F^2	1.059
Final R indexes [I>=2σ (I)]	R_1 = 0.0280, wR_2 = 0.0787
Final R indexes [all data]	R_1 = 0.0284, wR_2 = 0.0791
Largest diff. peak/hole / e Å^{-3}	0.30/-0.17
Flack parameter	0.01(3)

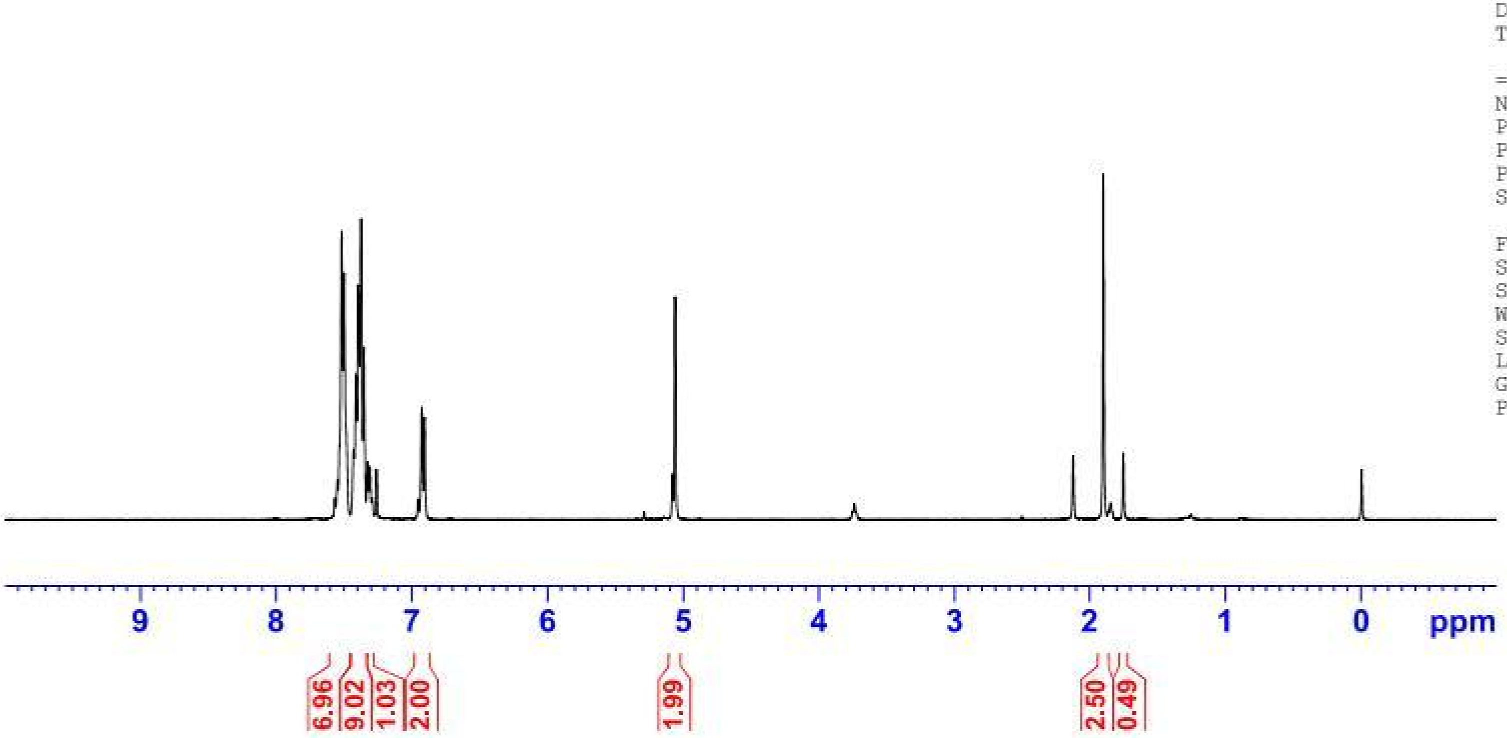

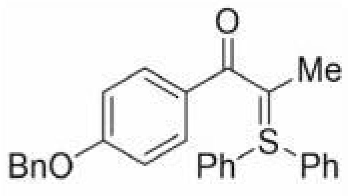

1d

```
Current Data Parameters
NAME          qcx-6-111b
EXPNO                  1
PROCNO                 1

F2 - Acquisition Parameters
Date_           20230421
Time               18.28
INSTRUM            spect
PROBHD   5 mm DUL 13C-1
PULPROG             zg30
TD                 65536
SOLVENT            CDCl3
NS                     7
DS                     2
SWH             8223.685 Hz
FIDRES          0.125483 Hz
AQ             3.9845889 sec
RG                   322
DW                60.800 usec
DE                  6.00 usec
TE                 292.8 K
D1            1.00000000 sec
TD0                    1

======== CHANNEL f1 ========
NUC1                  1H
P1                 15.80 usec
PL1                -1.00 dB
PL1W         12.17476940 W
SFO1         400.1324710 MHz

F2 - Processing parameters
SI                 32768
SF           400.1300104 MHz
WDW                   EM
SSB                    0
LB                  0.30 Hz
GB                     0
PC                  1.00
```

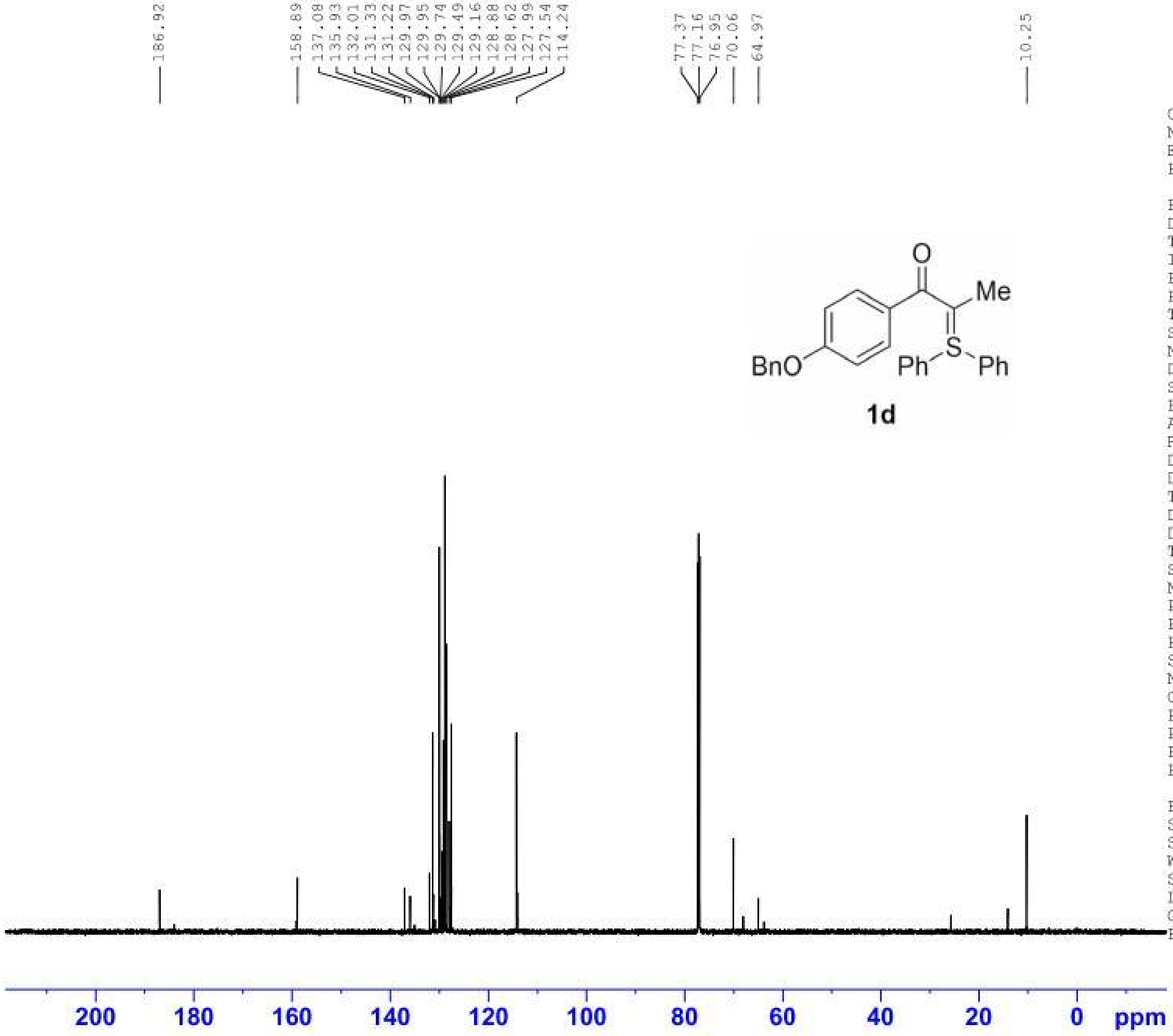

Current Data Parameters
NAME qcx-b-4
EXPNO 2
PROCNO 1

F2 - Acquisition Parameters
Date_ 20230528
Time 11.35 h
INSTRUM Avance NEO 600
PROBHD Z168773_0025 (
PULPROG zgpg30
TD 65536
SOLVENT CDCl3
NS 38
DS 4
SWH 35714.285 Hz
FIDRES 1.089913 Hz
AQ 0.9175040 sec
RG 3.56
DW 14.000 usec
DE 6.50 usec
TE 298.0 K
D1 2.00000000 sec
D11 0.03000000 sec
TD0 1
SFO1 150.9355021 MHz
NUC1 13C
P0 3.33 usec
P1 10.00 usec
PLW1 81.31300354 W
SFO2 600.2024008 MHz
NUC2 1H
CPDPRG[2 waltz65
PCPD2 70.00 usec
PLW2 18.05400085 W
PLW12 0.53056997 W
PLW13 0.26686999 W

F2 - Processing parameters
SI 32768
SF 150.9204004 MHz
WDW EM
SSB 0
LB 1.00 Hz
GB 0
PC 1.40

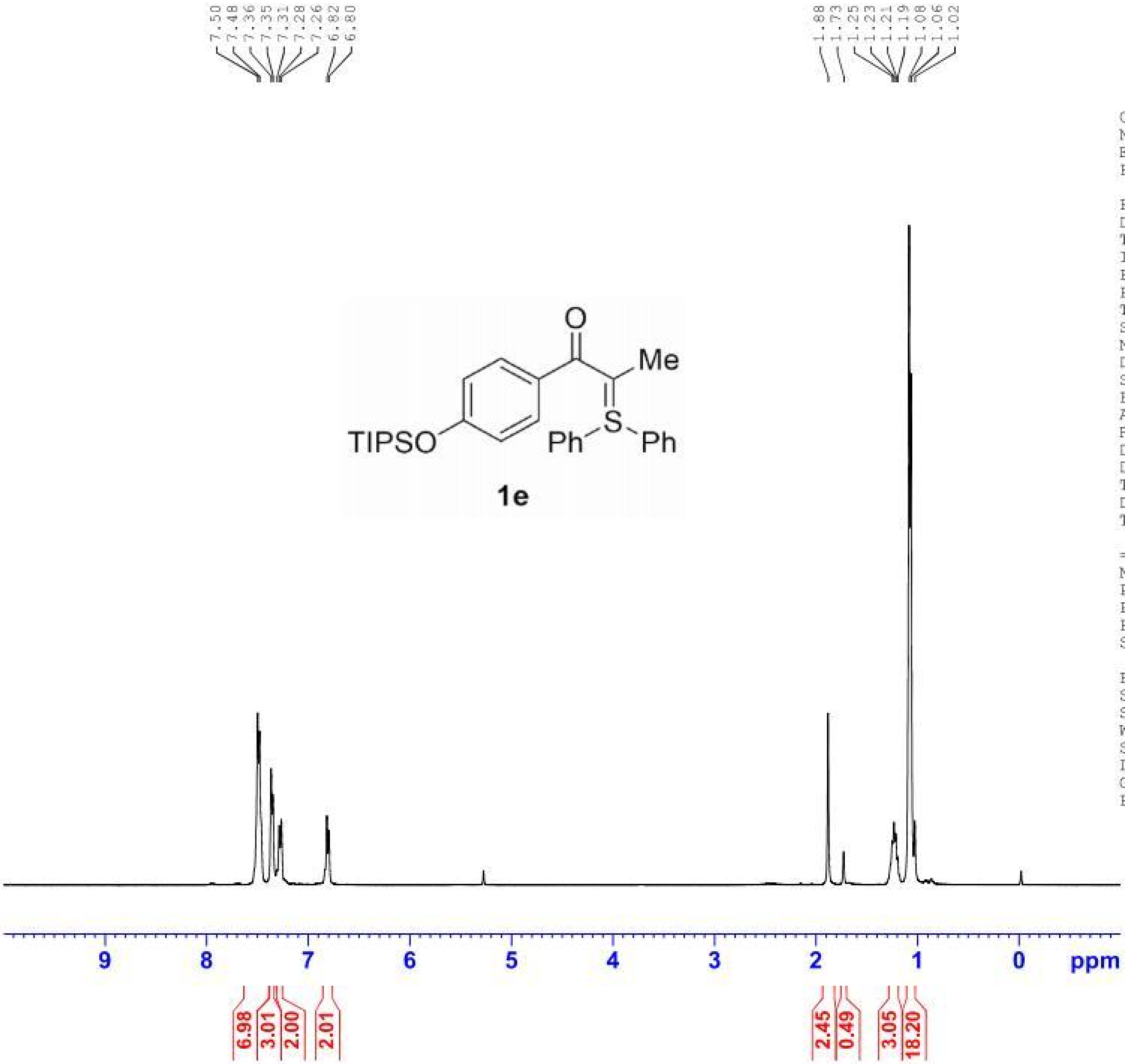


```
Current Data Parameters
NAME          qcx-6-111e
EXPNO                  1
PROCNO                 1

F2 - Acquisition Parameters
Date_           20230421
Time               19.09
INSTRUM            spect
PROBHD   5 mm DUL 13C-1
PULPROG             zg30
TD                 65536
SOLVENT            CDCl3
NS                     4
DS                     2
SWH             8223.685 Hz
FIDRES          0.125483 Hz
AQ             3.9845889 sec
RG                   144
DW                60.800 usec
DE                  6.00 usec
TE                 292.9 K
D1            1.00000000 sec
TD0                    1

======== CHANNEL f1 ========
NUC1                  1H
P1                 15.80 usec
PL1                -1.00 dB
PL1W         12.17476940 W
SFO1         400.1324710 MHz

F2 - Processing parameters
SI                 32768
SF           400.1300109 MHz
WDW                   EM
SSB                    0
LB                  0.30 Hz
GB                     0
PC                  1.00
```

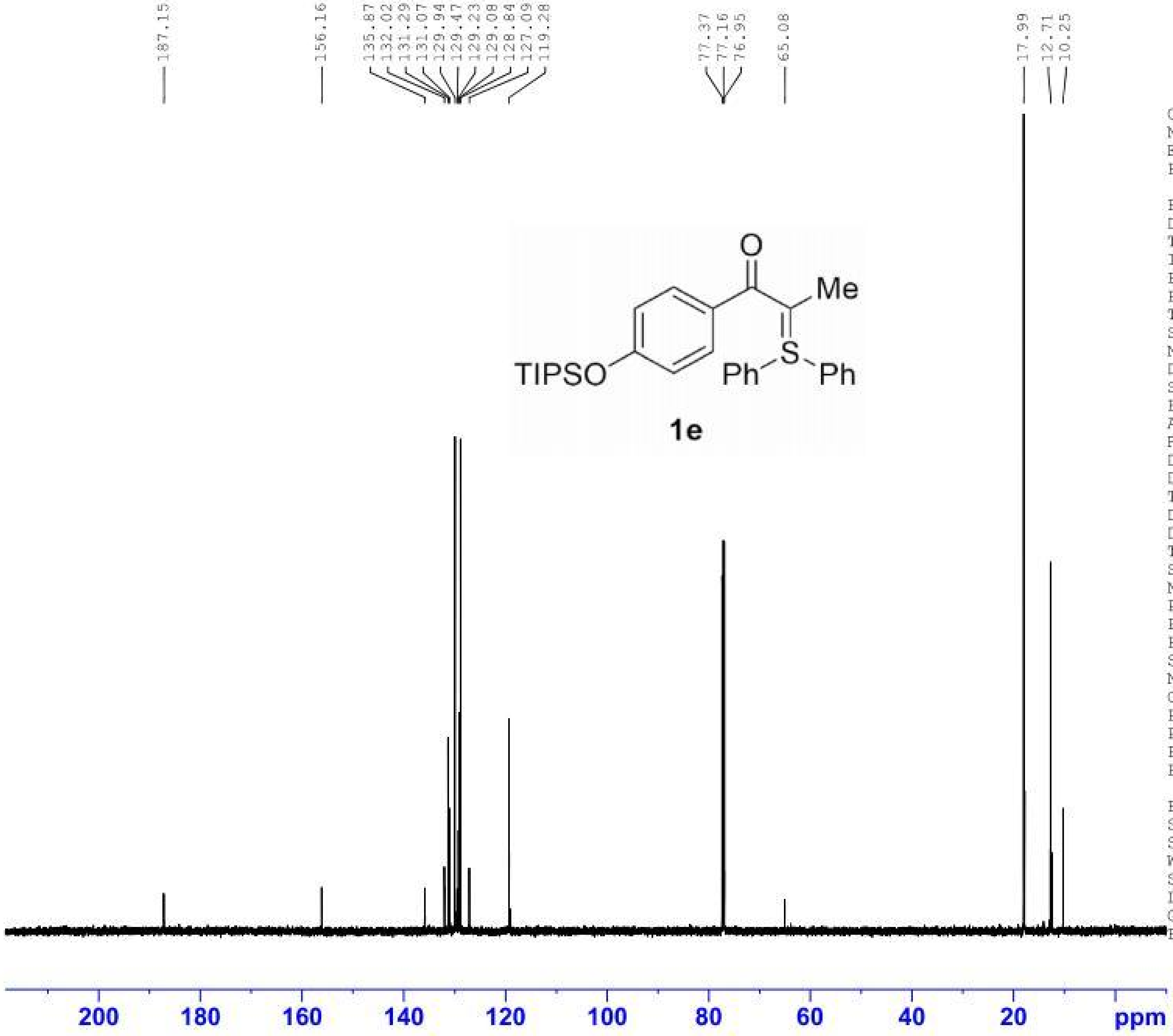

1e

```
Current Data Parameters
NAME            qcx-e
EXPNO               1
PROCNO              1

F2 - Acquisition Parameters
Date_        20230528
Time            11.57 h
INSTRUM  Avance NEO 600
PROBHD   Z168773_0025 (
PULPROG        zgpg30
TD              65536
SOLVENT         CDCl3
NS                 11
DS                  4
SWH         35714.285 Hz
FIDRES       1.089913 Hz
AQ          0.9175040 sec
RG                  4
DW             14.000 usec
DE               6.50 usec
TE              298.0 K
D1         2.00000000 sec
D11        0.03000000 sec
TD0                 1
SFO1      150.9355021 MHz
NUC1              13C
P0               3.33 usec
P1              10.00 usec
PLW1      81.31300354 W
SFO2      600.2024008 MHz
NUC2               1H
CPDPRG[2      waltz65
PCPD2           70.00 usec
PLW2      18.05400085 W
PLW12      0.53056997 W
PLW13      0.26686999 W

F2 - Processing parameters
SI              32768
SF        150.9204017 MHz
WDW                EM
SSB                 0
LB               1.00 Hz
GB                  0
PC               1.40
```

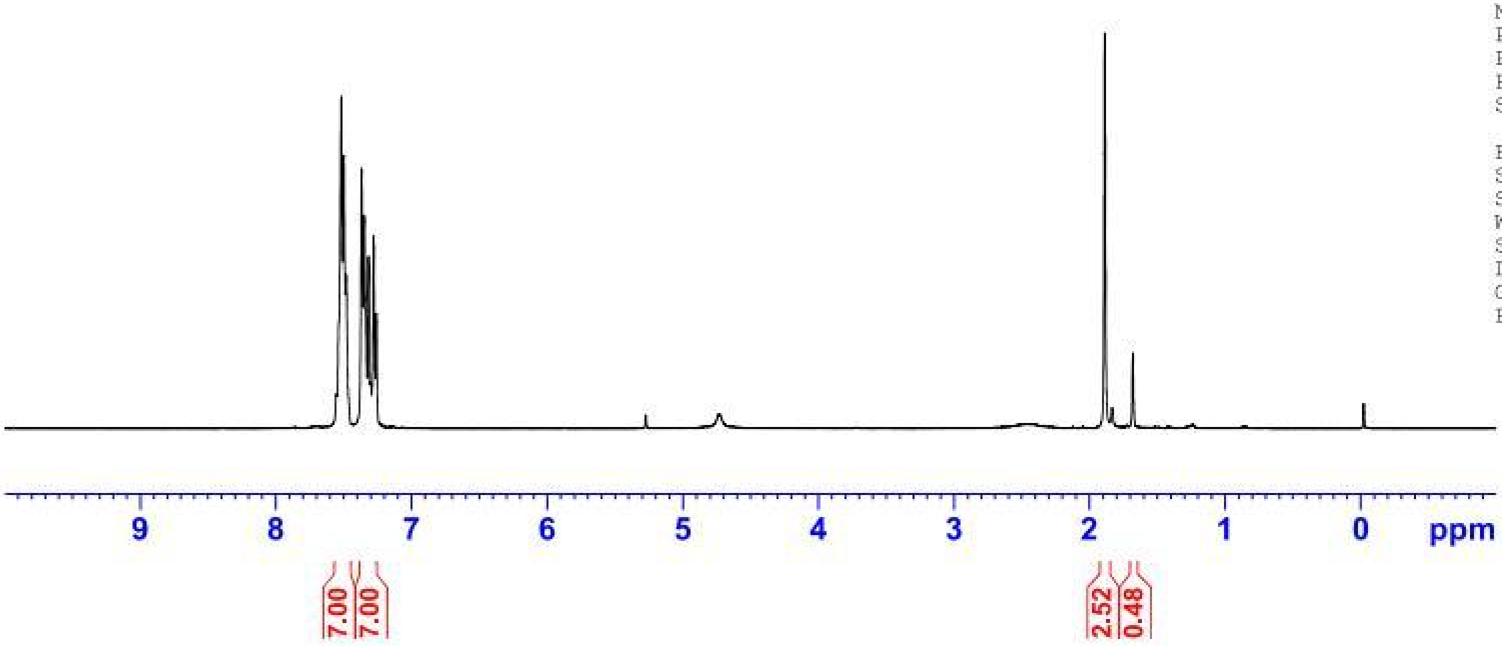

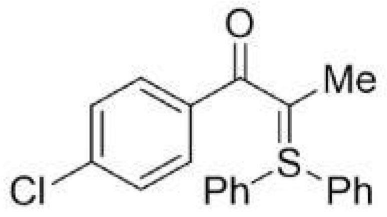

1g

Current Data Parameters
NAME qcx-6-111a
EXPNO 1
PROCNO 1

F2 - Acquisition Parameters
Date_ 20230421
Time 18.16
INSTRUM spect
PROBHD 5 mm DUL 13C-1
PULPROG zg30
TD 65536
SOLVENT CDCl3
NS 6
DS 2
SWH 8223.685 Hz
FIDRES 0.125483 Hz
AQ 3.9845889 sec
RG 228
DW 60.800 usec
DE 6.00 usec
TE 292.7 K
D1 1.00000000 sec
TD0 1

======== CHANNEL f1 ========
NUC1 1H
P1 15.80 usec
PL1 -1.00 dB
PL1W 12.17476940 W
SFO1 400.1324710 MHz

F2 - Processing parameters
SI 32768
SF 400.1300100 MHz
WDW EM
SSB 0
LB 0.30 Hz
GB 0
PC 1.00

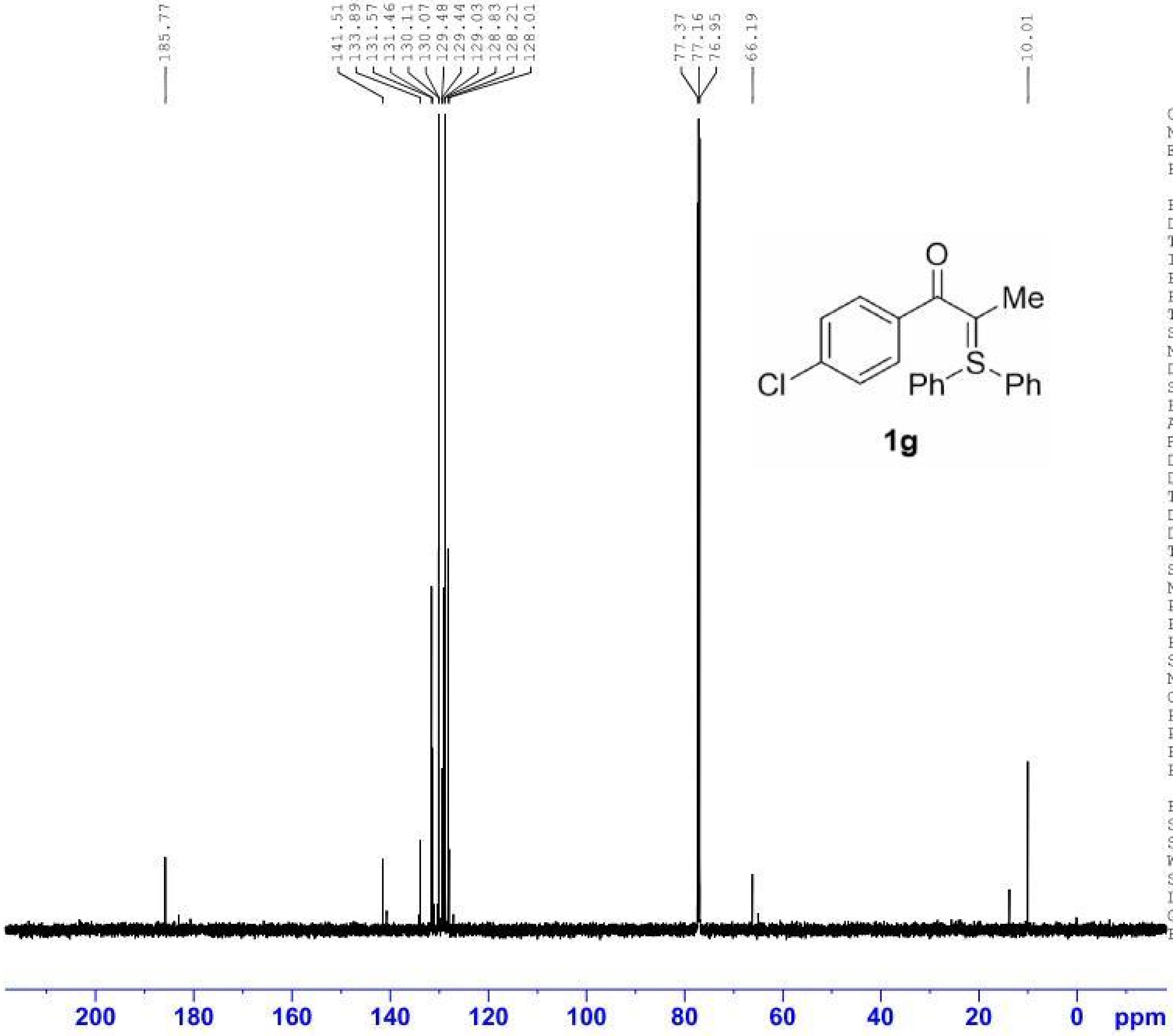

Current Data Parameters
NAME qcx-a
EXPNO 3
PROCNO 1

F2 - Acquisition Parameters
Date_ 20230528
Time 11.26 h
INSTRUM Avance NEO 600
PROBHD Z168773_0025 (
PULPROG zgpg30
TD 65536
SOLVENT CDCl3
NS 17
DS 4
SWH 35714.285 Hz
FIDRES 1.089913 Hz
AQ 0.9175040 sec
RG 3.2
DW 14.000 usec
DE 6.50 usec
TE 298.0 K
D1 2.00000000 sec
D11 0.03000000 sec
TD0 1
SFO1 150.9355021 MHz
NUC1 13C
P0 3.33 usec
P1 10.00 usec
PLW1 81.31300354 W
SFO2 600.2024008 MHz
NUC2 1H
CPDPRG[2 waltz65
PCPD2 70.00 usec
PLW2 18.05400085 W
PLW12 0.53056997 W
PLW13 0.26686999 W

F2 - Processing parameters
SI 32768
SF 150.9204006 MHz
WDW EM
SSB 0
LB 1.00 Hz
GB 0
PC 1.40

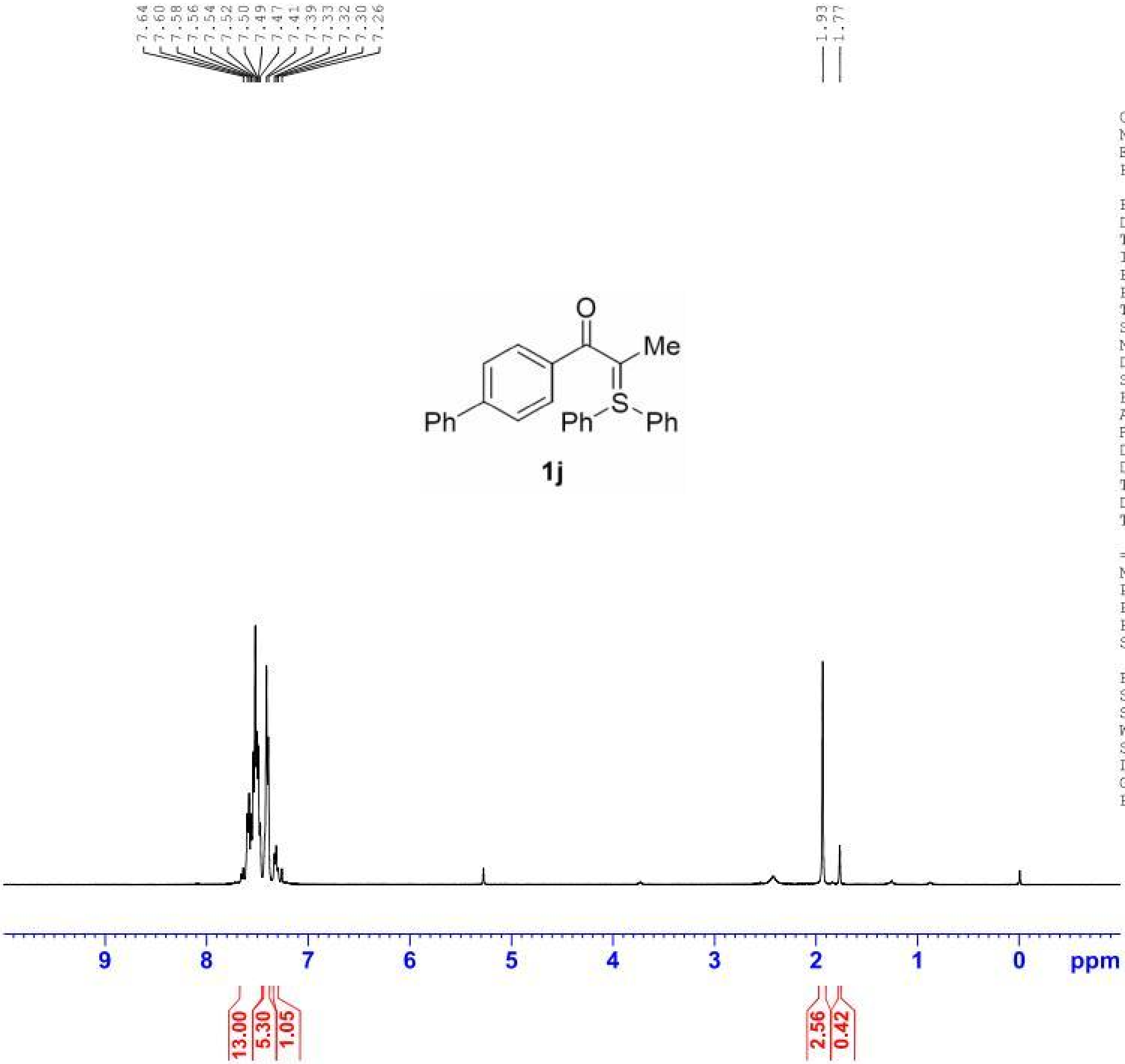

1j

```
Current Data Parameters
NAME          qcx-6-111d
EXPNO                  1
PROCNO                 1

F2 - Acquisition Parameters
Date_           20230421
Time               18.59
INSTRUM            spect
PROBHD   5 mm DUL 13C-1
PULPROG             zg30
TD                 65536
SOLVENT            CDCl3
NS                     5
DS                     2
SWH             8223.685 Hz
FIDRES          0.125483 Hz
AQ             3.9845889 sec
RG                   228
DW                60.800 usec
DE                  6.00 usec
TE                 292.9 K
D1            1.00000000 sec
TD0                    1

======== CHANNEL f1 ========
NUC1                  1H
P1                 15.80 usec
PL1                -1.00 dB
PL1W         12.17476940 W
SFO1         400.1324710 MHz

F2 - Processing parameters
SI                 32768
SF           400.1300105 MHz
WDW                   EM
SSB                    0
LB                  0.30 Hz
GB                     0
PC                  1.00
```

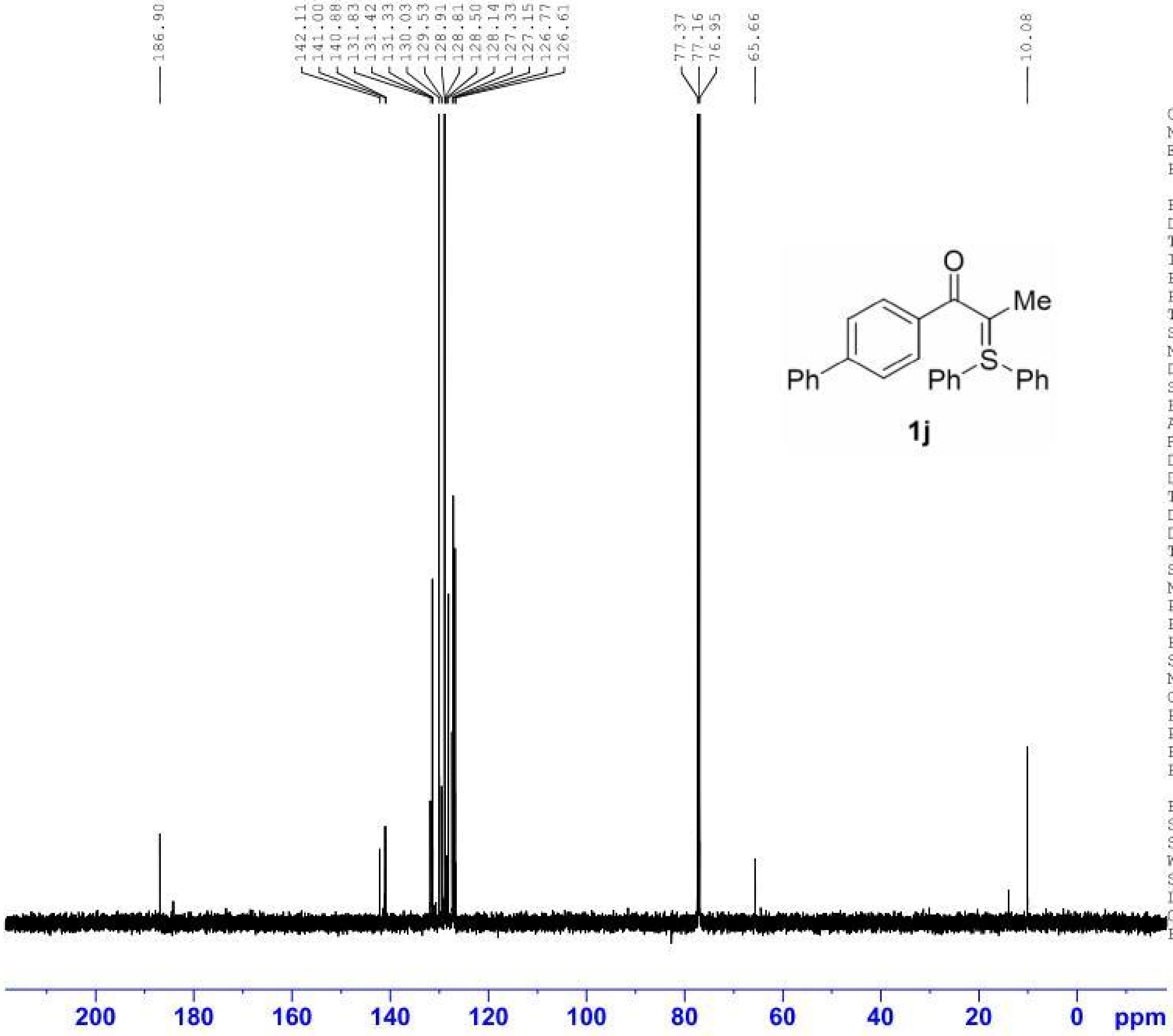

1j

Current Data Parameters
NAME qcx-d
EXPNO 3
PROCNO 1

F2 - Acquisition Parameters
Date_ 20230528
Time 11.52 h
INSTRUM Avance NEO 600
PROBHD Z168773_0025 (
PULPROG zgpg30
TD 65536
SOLVENT CDCl3
NS 11
DS 4
SWH 35714.285 Hz
FIDRES 1.089913 Hz
AQ 0.9175040 sec
RG 3.56
DW 14.000 usec
DE 6.50 usec
TE 298.0 K
D1 2.00000000 sec
D11 0.03000000 sec
TD0 1
SFO1 150.9355021 MHz
NUC1 13C
P0 3.33 usec
P1 10.00 usec
PLW1 81.31300354 W
SFO2 600.2024008 MHz
NUC2 1H
CPDPRG[2 waltz65
PCPD2 70.00 usec
PLW2 18.05400085 W
PLW12 0.53056997 W
PLW13 0.26686999 W

F2 - Processing parameters
SI 32768
SF 150.9203998 MHz
WDW EM
SSB 0
LB 1.00 Hz
GB 0
PC 1.40

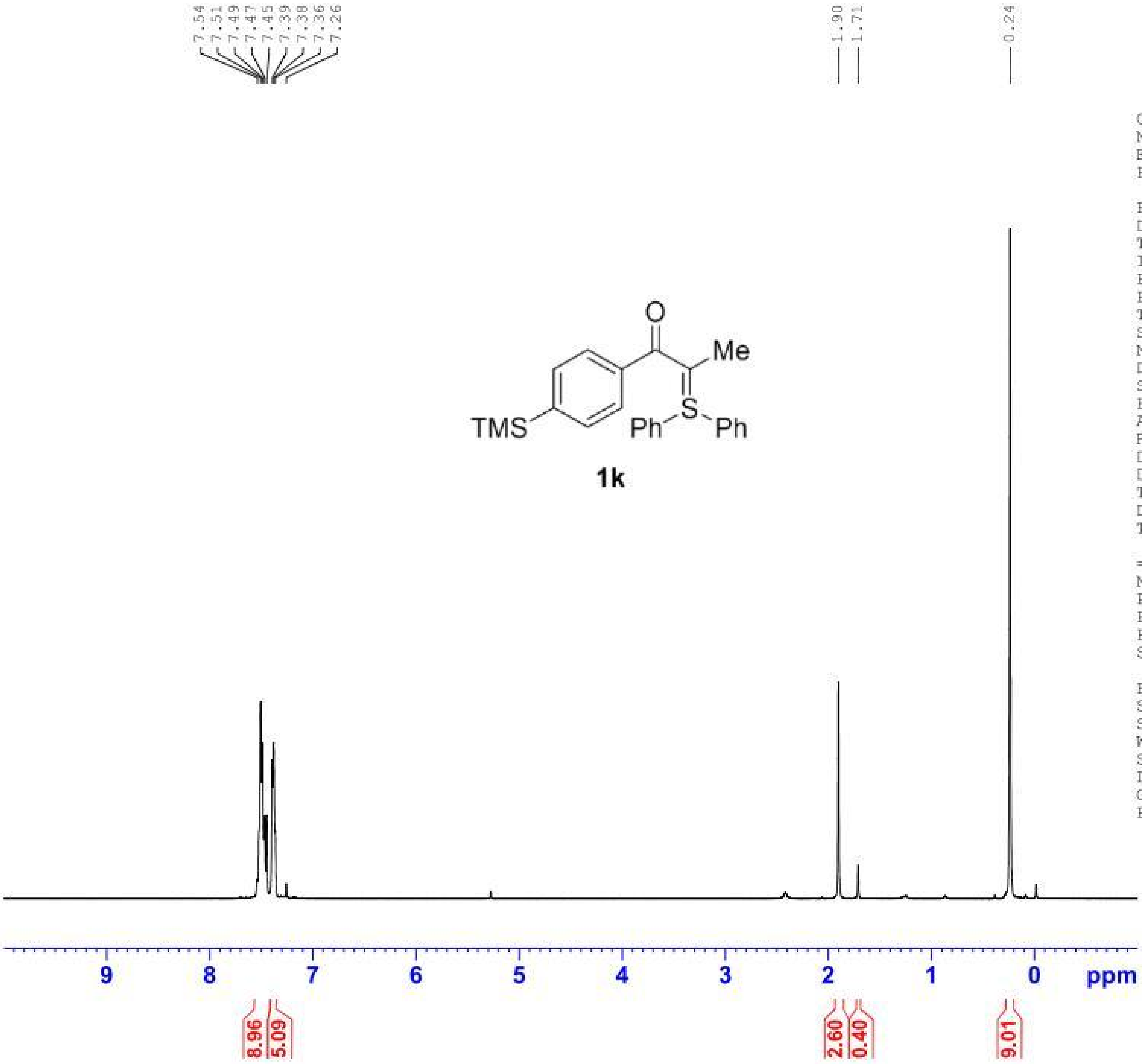


```
Current Data Parameters
NAME          qcx-6-111c
EXPNO                  1
PROCNO                 1

F2 - Acquisition Parameters
Date_           20230421
Time               18.50
INSTRUM            spect
PROBHD   5 mm DUL 13C-1
PULPROG             zg30
TD                 65536
SOLVENT            CDCl3
NS                     3
DS                     2
SWH             8223.685 Hz
FIDRES          0.125483 Hz
AQ             3.9845889 sec
RG                   203
DW                60.800 usec
DE                  6.00 usec
TE                 292.9 K
D1            1.00000000 sec
TD0                    1

======== CHANNEL f1 ========
NUC1                  1H
P1                 15.80 usec
PL1                -1.00 dB
PL1W         12.17476940 W
SFO1         400.1324710 MHz

F2 - Processing parameters
SI                 32768
SF           400.1300107 MHz
WDW                   EM
SSB                    0
LB                  0.30 Hz
GB                     0
PC                  1.00
```

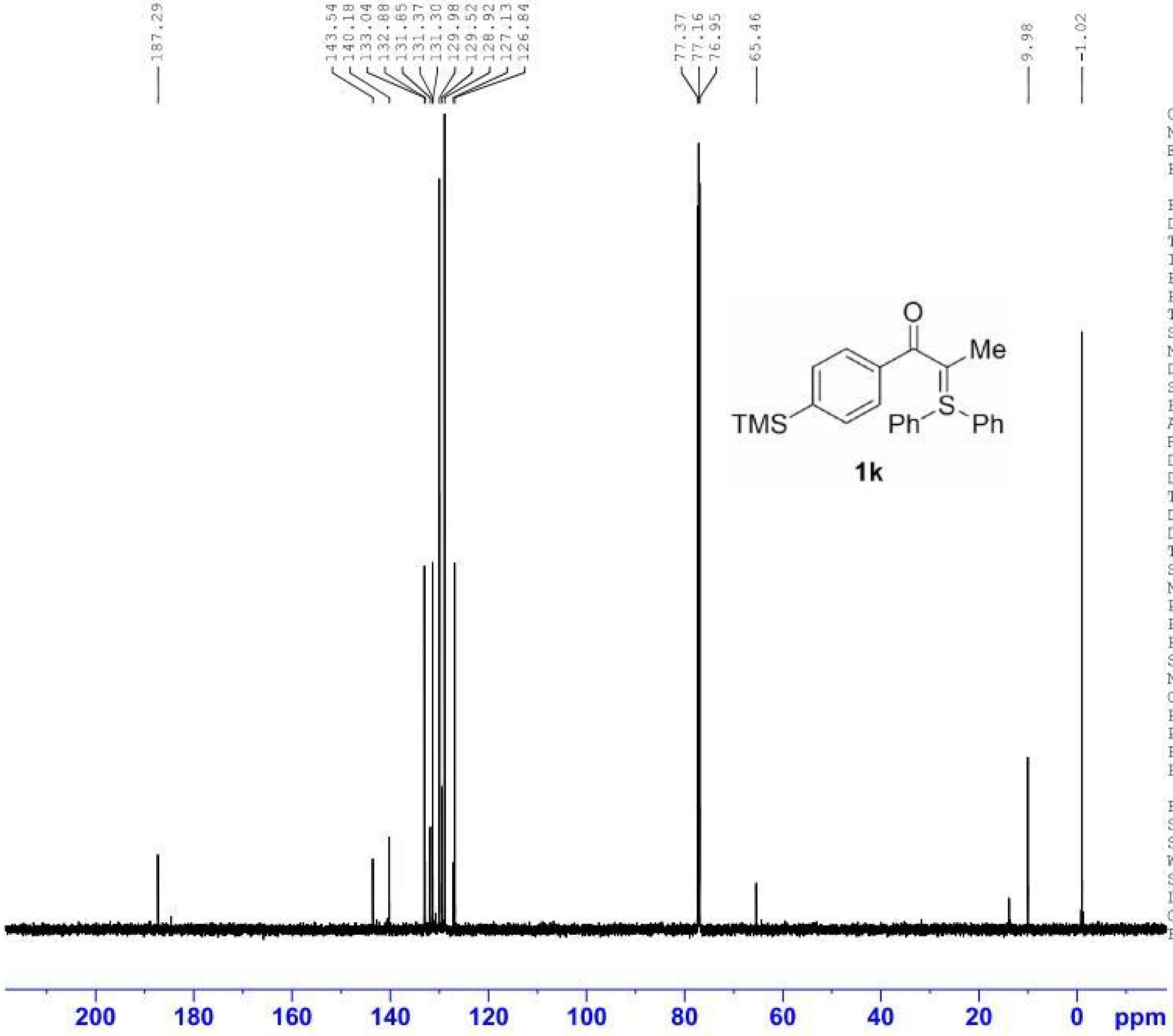

Current Data Parameters

NAME	qcx-c
EXPNO	2
PROCNO	1

F2 - Acquisition Parameters

Date_	20230528
Time	11.44 h
INSTRUM	Avance NEO 600
PROBHD	Z168773_0025 (
PULPROG	zgpg30
TD	65536
SOLVENT	CDCl3
NS	29
DS	4
SWH	35714.285 Hz
FIDRES	1.089913 Hz
AQ	0.9175040 sec
RG	3.56
DW	14.000 usec
DE	6.50 usec
TE	298.0 K
D1	2.00000000 sec
D11	0.03000000 sec
TD0	1
SFO1	150.9355021 MHz
NUC1	13C
P0	3.33 usec
P1	10.00 usec
PLW1	81.31300354 W
SFO2	600.2024008 MHz
NUC2	1H
CPDPRG[2	waltz65
PCPD2	70.00 usec
PLW2	18.05400085 W
PLW12	0.53056997 W
PLW13	0.26686999 W

F2 - Processing parameters

SI	32768
SF	150.9203984 MHz
WDW	EM
SSB	0
LB	1.00 Hz
GB	0
PC	1.40

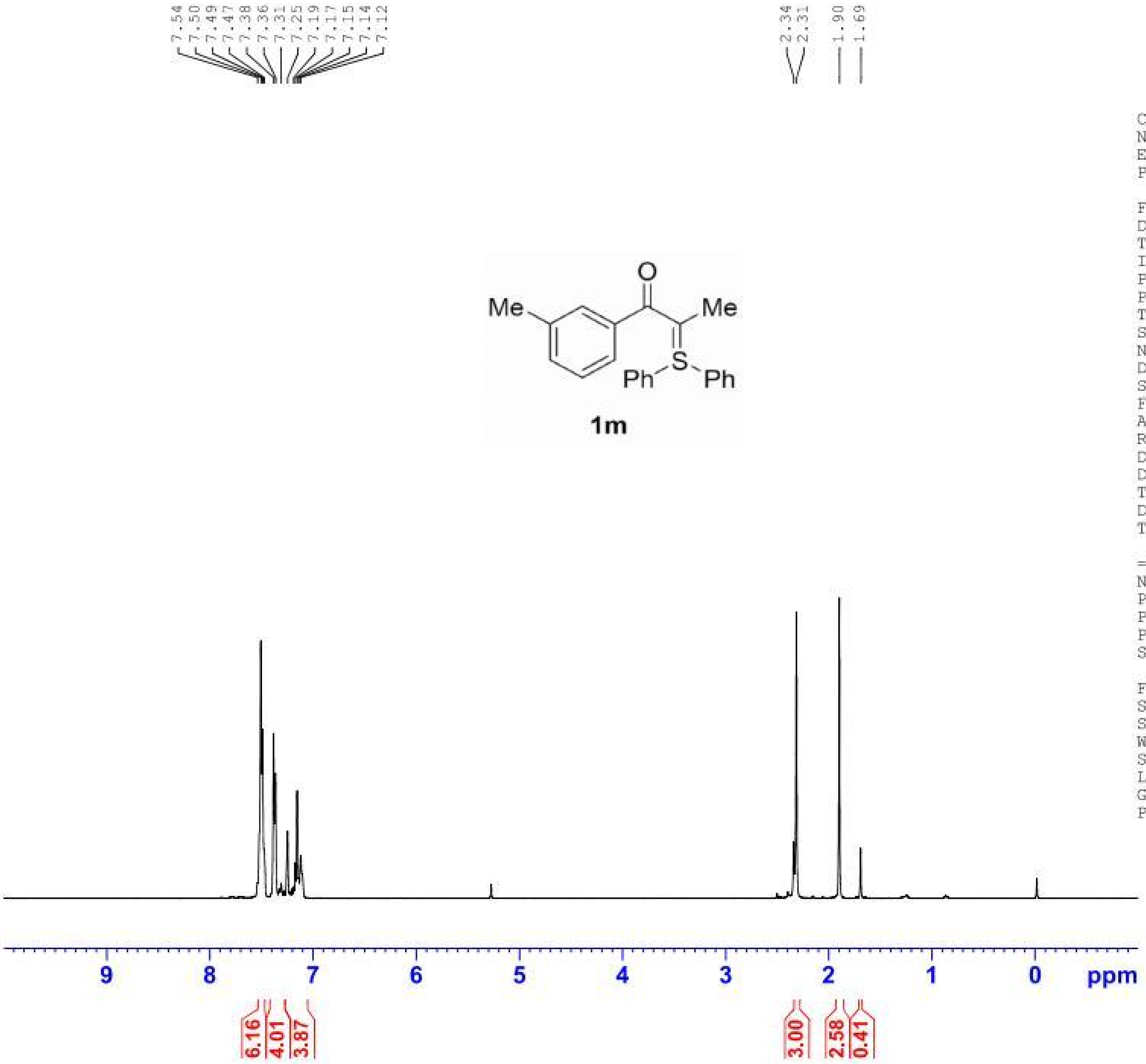

Current Data Parameters
NAME qcx-6-111f
EXPNO 1
PROCNO 1

F2 - Acquisition Parameters
Date_ 20230422
Time 20.20
INSTRUM spect
PROBHD 5 mm DUL 13C-1
PULPROG zg30
TD 65536
SOLVENT CDCl3
NS 2
DS 2
SWH 8223.685 Hz
FIDRES 0.125483 Hz
AQ 3.9845889 sec
RG 287
DW 60.800 usec
DE 6.00 usec
TE 294.9 K
D1 1.00000000 sec
TD0 1

======== CHANNEL f1 ========
NUC1 1H
P1 15.80 usec
PL1 -1.00 dB
PL1W 12.17476940 W
SFO1 400.1324710 MHz

F2 - Processing parameters
SI 32768
SF 400.1300116 MHz
WDW EM
SSB 0
LB 0.30 Hz
GB 0
PC 1.00

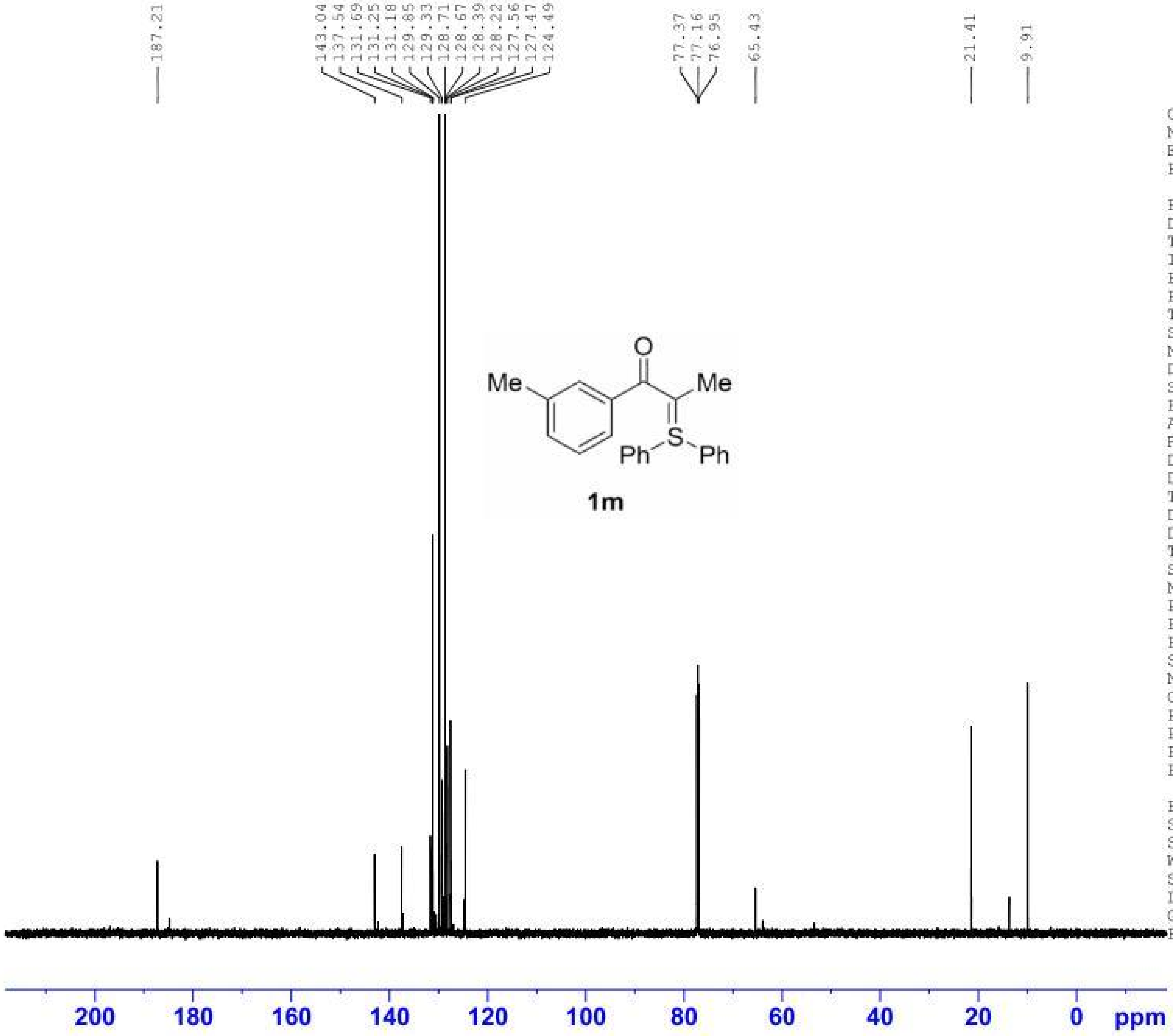

1m

```
Current Data Parameters
NAME              qcx-f
EXPNO                 1
PROCNO                1

F2 - Acquisition Parameters
Date_          20230528
Time              12.01 h
INSTRUM  Avance NEO 600
PROBHD   Z168773_0025 (
PULPROG         zgpg30
TD               65536
SOLVENT          CDCl3
NS                   7
DS                   4
SWH          35714.285 Hz
FIDRES        1.089913 Hz
AQ           0.9175040 sec
RG                 3.2
DW              14.000 usec
DE                6.50 usec
TE               298.0 K
D1          2.00000000 sec
D11         0.03000000 sec
TD0                  1
SFO1       150.9355021 MHz
NUC1               13C
P0                3.33 usec
P1               10.00 usec
PLW1       81.31300354 W
SFO2       600.2024008 MHz
NUC2                1H
CPDPRG[2       waltz65
PCPD2            70.00 usec
PLW2       18.05400085 W
PLW12       0.53056997 W
PLW13       0.26686999 W

F2 - Processing parameters
SI               32768
SF         150.9204190 MHz
WDW                 EM
SSB                  0
LB                1.00 Hz
GB                   0
PC                1.40
```

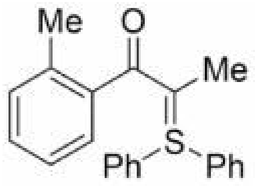

1n

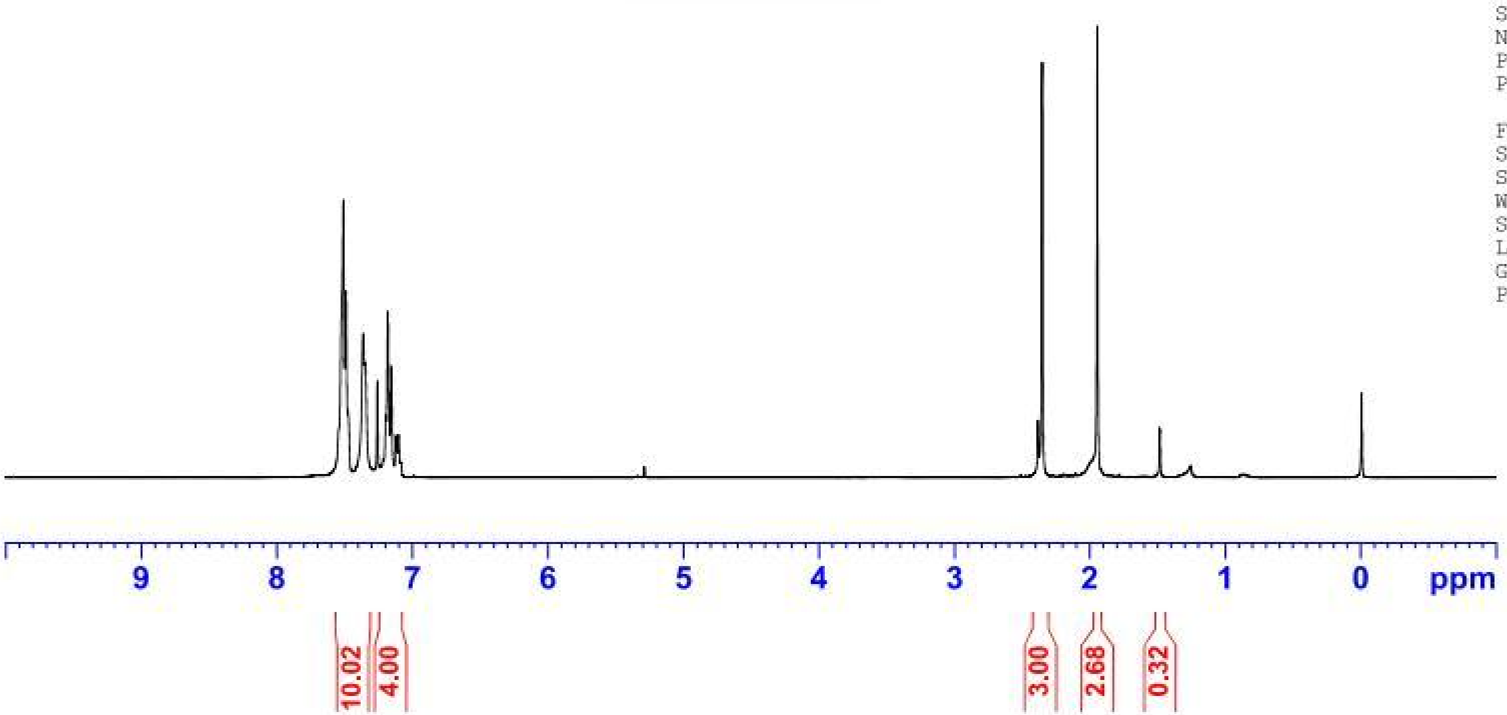

Current Data Parameters
NAME qcx-9-209p
EXPNO 1
PROCNO 1

F2 - Acquisition Parameters
Date_ 20250724
Time 15.11 h
INSTRUM spect
PROBHD Z116098_0761 (
PULPROG zg30
TD 65536
SOLVENT CDCl3
NS 16
DS 2
SWH 8012.820 Hz
FIDRES 0.244532 Hz
AQ 4.0894465 sec
RG 78.76
DW 62.400 usec
DE 6.50 usec
TE 299.0 K
D1 1.00000000 sec
TD0 1
SFO1 400.1324708 MHz
NUC1 1H
P1 10.00 usec
PLW1 16.24399948 W

F2 - Processing parameters
SI 65536
SF 400.1300105 MHz
WDW EM
SSB 0
LB 0.30 Hz
GB 0
PC 1.00

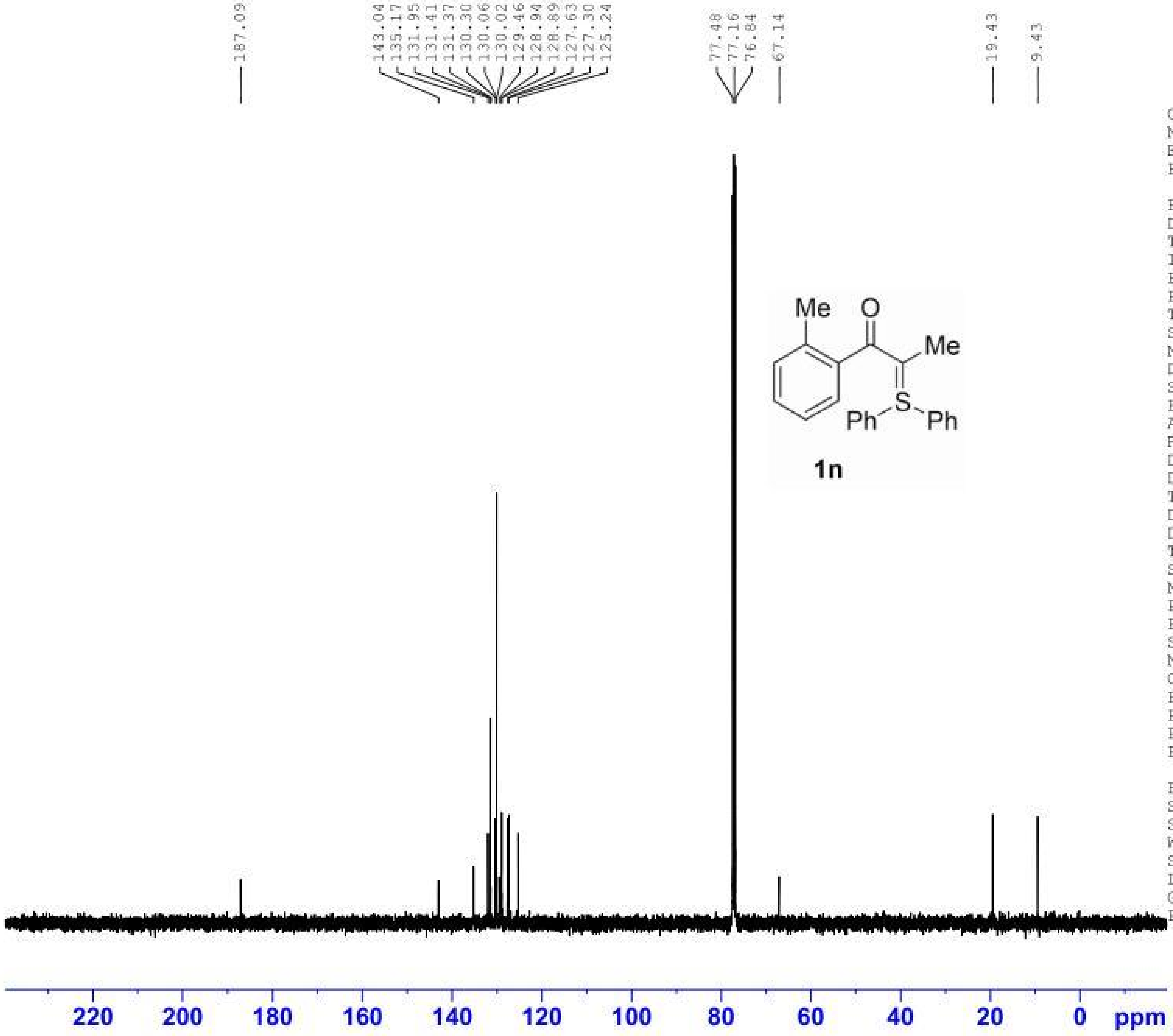


```
Current Data Parameters
NAME              qcx-9-209p
EXPNO                      2
PROCNO                     1

F2 - Acquisition Parameters
Date_               20250724
Time                   15.27 h
INSTRUM                spect
PROBHD    Z116098_0761 (
PULPROG              zgpg30
TD                     65536
SOLVENT                CDCl3
NS                       260
DS                         4
SWH                26041.666 Hz
FIDRES              0.794729 Hz
AQ                 1.2582912 sec
RG                    198.89
DW                    19.200 usec
DE                      6.50 usec
TE                     299.7 K
D1                2.00000000 sec
D11               0.03000000 sec
TD0                        1
SFO1             100.6238359 MHz
NUC1                     13C
P1                     10.00 usec
PLW1             79.28600311 W
SFO2             400.1316005 MHz
NUC2                      1H
CPDPRG[2             waltz16
PCPD2                  90.00 usec
PLW2             16.24399948 W
PLW12             0.20054001 W
PLW13             0.10087000 W

F2 - Processing parameters
SI                     32768
SF               100.6127568 MHz
WDW                       EM
SSB                        0
LB                      1.00 Hz
GB                         0
PC                      1.40
```

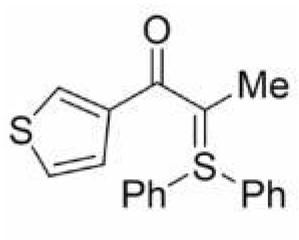

1q

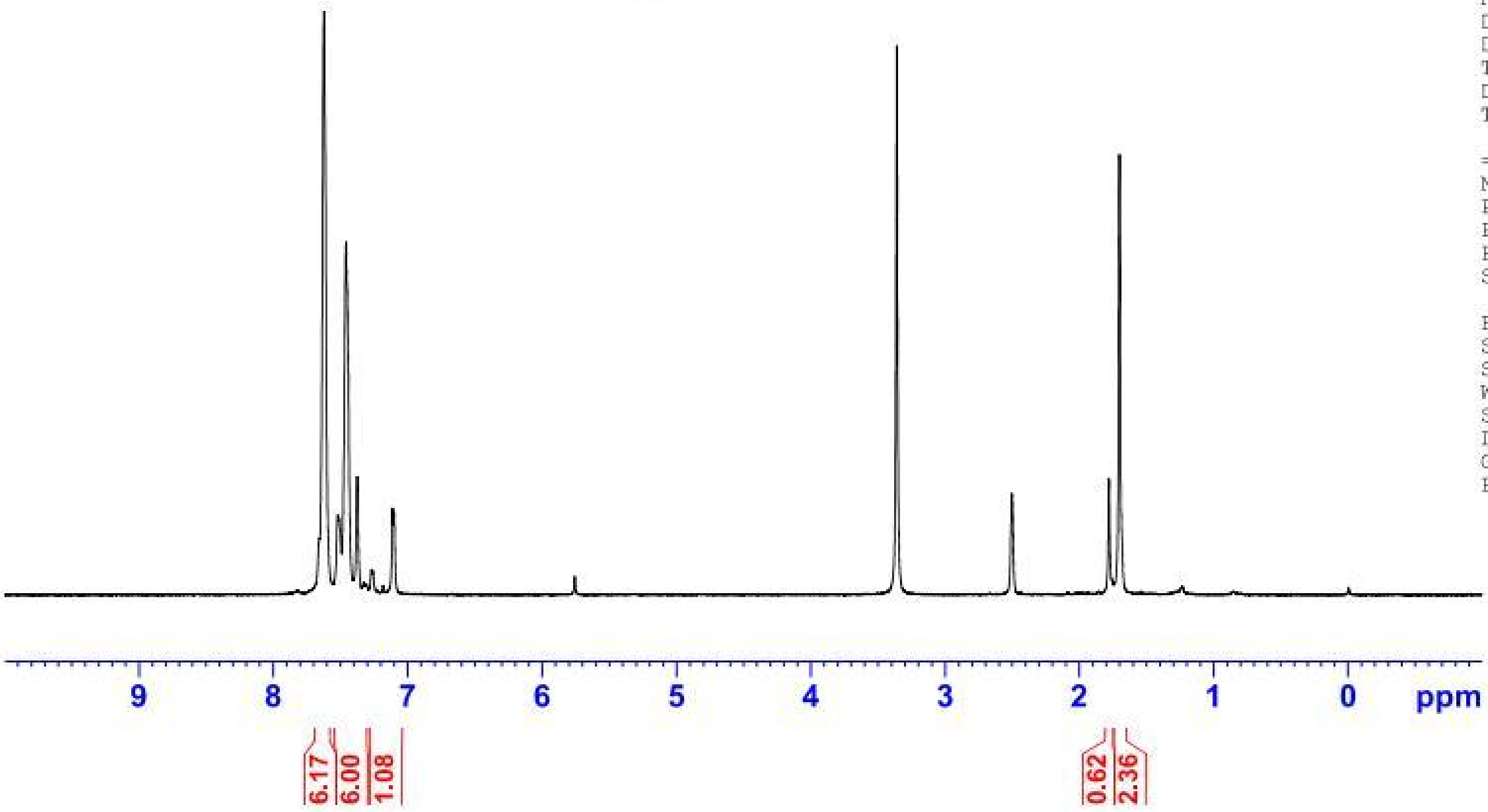


```
Current Data Parameters
NAME             qcx-6-111g
EXPNO                     1
PROCNO                    1

F2 - Acquisition Parameters
Date_              20230422
Time                  19.37
INSTRUM               spect
PROBHD    5 mm DUL 13C-1
PULPROG                zg30
TD                    65536
SOLVENT                DMSO
NS                        3
DS                        2
SWH                8223.685 Hz
FIDRES             0.125483 Hz
AQ                3.9845889 sec
RG                      362
DW                   60.800 usec
DE                     6.00 usec
TE                    294.6 K
D1               1.00000000 sec
TD0                       1

======== CHANNEL f1 ========
NUC1                     1H
P1                    15.80 usec
PL1                   -1.00 dB
PL1W            12.17476940 W
SFO1            400.1324710 MHz

F2 - Processing parameters
SI                    32768
SF              400.1300034 MHz
WDW                      EM
SSB                       0
LB                     0.30 Hz
GB                        0
PC                     1.00
```

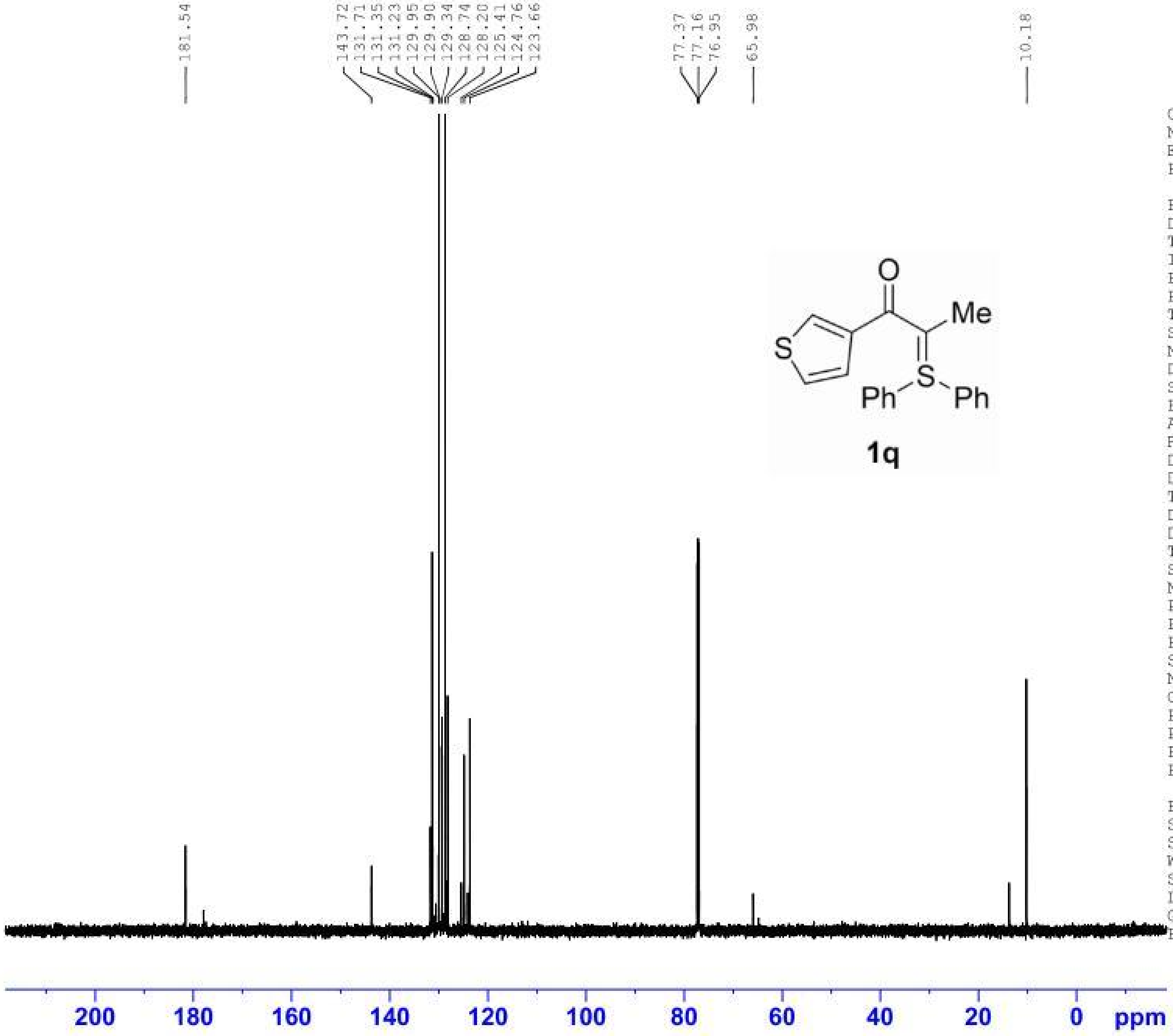

1q

```
Current Data Parameters
NAME                 qcx-g
EXPNO                    1
PROCNO                   1

F2 - Acquisition Parameters
Date_             20230528
Time                 12.05 h
INSTRUM     Avance NEO 600
PROBHD    Z168773_0025 (
PULPROG            zgpg30
TD                   65536
SOLVENT              CDCl3
NS                       7
DS                       4
SWH              35714.285 Hz
FIDRES            1.089913 Hz
AQ               0.9175040 sec
RG                    3.56
DW                  14.000 usec
DE                    6.50 usec
TE                   298.0 K
D1              2.00000000 sec
D11             0.03000000 sec
TD0                      1
SFO1           150.9355021 MHz
NUC1                   13C
P0                    3.33 usec
P1                   10.00 usec
PLW1           81.31300354 W
SFO2           600.2024008 MHz
NUC2                    1H
CPDPRG[2           waltz65
PCPD2                70.00 usec
PLW2           18.05400085 W
PLW12           0.53056997 W
PLW13           0.26686999 W

F2 - Processing parameters
SI                   32768
SF             150.9204148 MHz
WDW                     EM
SSB                      0
LB                    1.00 Hz
GB                       0
PC                    1.40
```

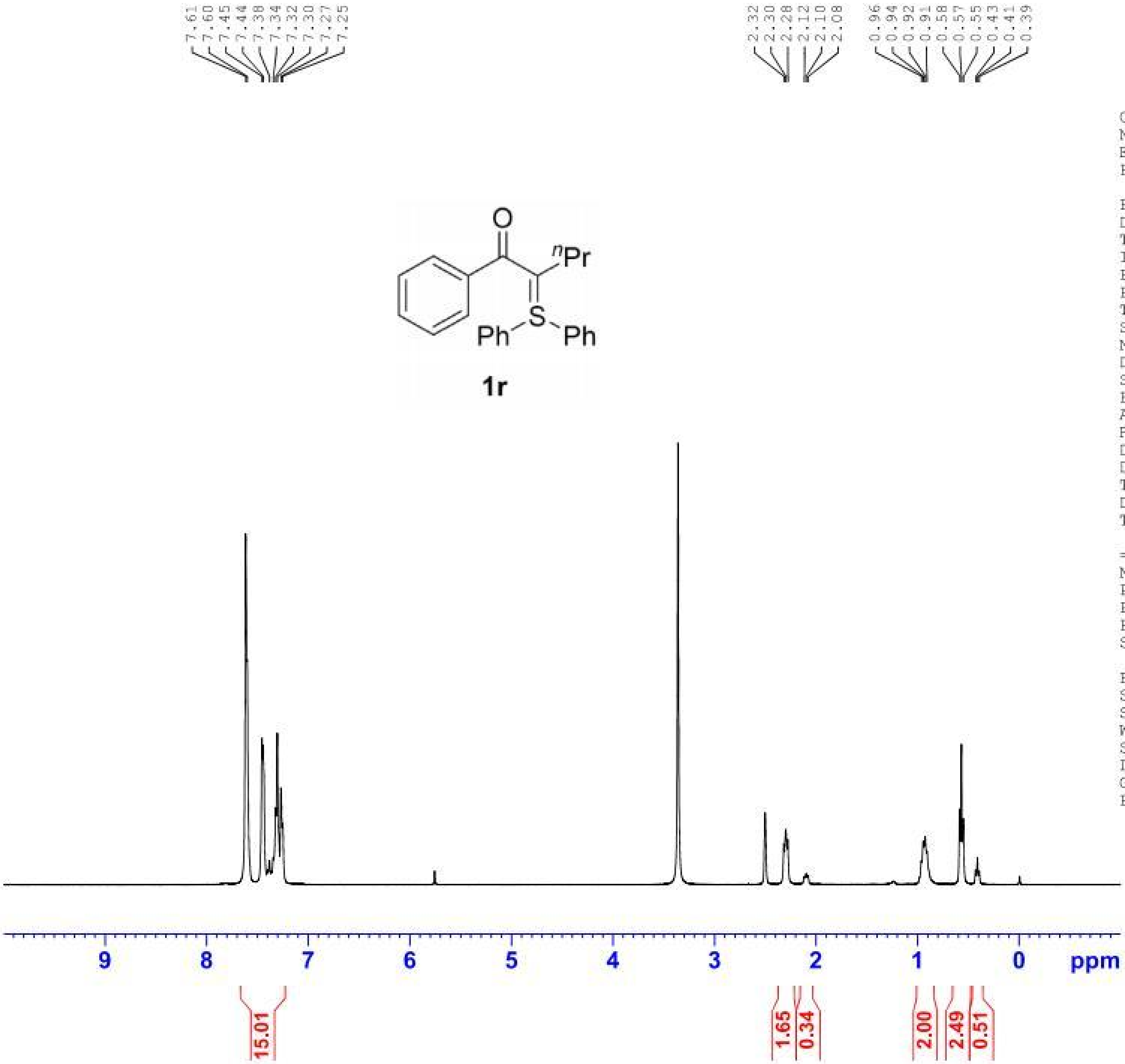


```
Current Data Parameters
NAME      qcx-6-111j-dmso
EXPNO                   1
PROCNO                  1

F2 - Acquisition Parameters
Date_            20230422
Time                19.06
INSTRUM             spect
PROBHD    5 mm DUL 13C-1
PULPROG              zg30
TD                  65536
SOLVENT              DMSO
NS                      5
DS                      2
SWH              8223.685 Hz
FIDRES           0.125483 Hz
AQ              3.9845889 sec
RG                    322
DW                 60.800 usec
DE                   6.00 usec
TE                  294.3 K
D1             1.00000000 sec
TD0                     1

======== CHANNEL f1 ========
NUC1                   1H
P1                  15.80 usec
PL1                 -1.00 dB
PL1W          12.17476940 W
SFO1          400.1324710 MHz

F2 - Processing parameters
SI                  32768
SF            400.1300036 MHz
WDW                    EM
SSB                     0
LB                   0.30 Hz
GB                      0
PC                   1.00
```

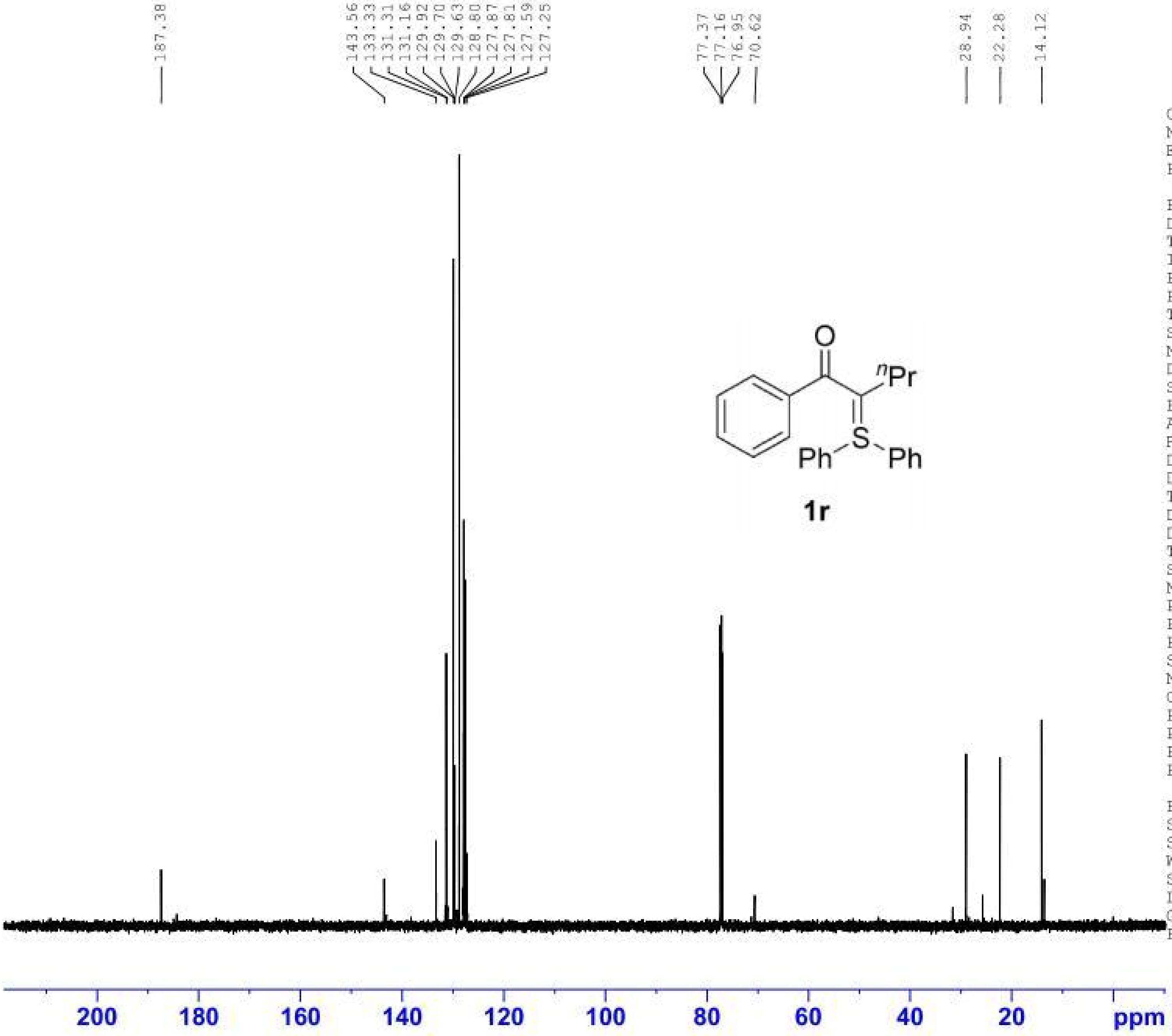


```
Current Data Parameters
NAME               qcx-j
EXPNO                  1
PROCNO                 1

F2 - Acquisition Parameters
Date_           20230528
Time               12.22 h
INSTRUM   Avance NEO 600
PROBHD   Z168773_0025 (
PULPROG           zgpg30
TD                 65536
SOLVENT            CDCl3
NS                     7
DS                     4
SWH            35714.285 Hz
FIDRES          1.089913 Hz
AQ             0.9175040 sec
RG                  3.56
DW                14.000 usec
DE                  6.50 usec
TE                 298.0 K
D1            2.00000000 sec
D11           0.03000000 sec
TD0                    1
SFO1         150.9355021 MHz
NUC1                 13C
P0                  3.33 usec
P1                 10.00 usec
PLW1         81.31300354 W
SFO2         600.2024008 MHz
NUC2                  1H
CPDPRG[2         waltz65
PCPD2              70.00 usec
PLW2         18.05400085 W
PLW12         0.53056997 W
PLW13         0.26686999 W

F2 - Processing parameters
SI                 32768
SF           150.9204048 MHz
WDW                   EM
SSB                    0
LB                  1.00 Hz
GB                     0
PC                  1.40
```

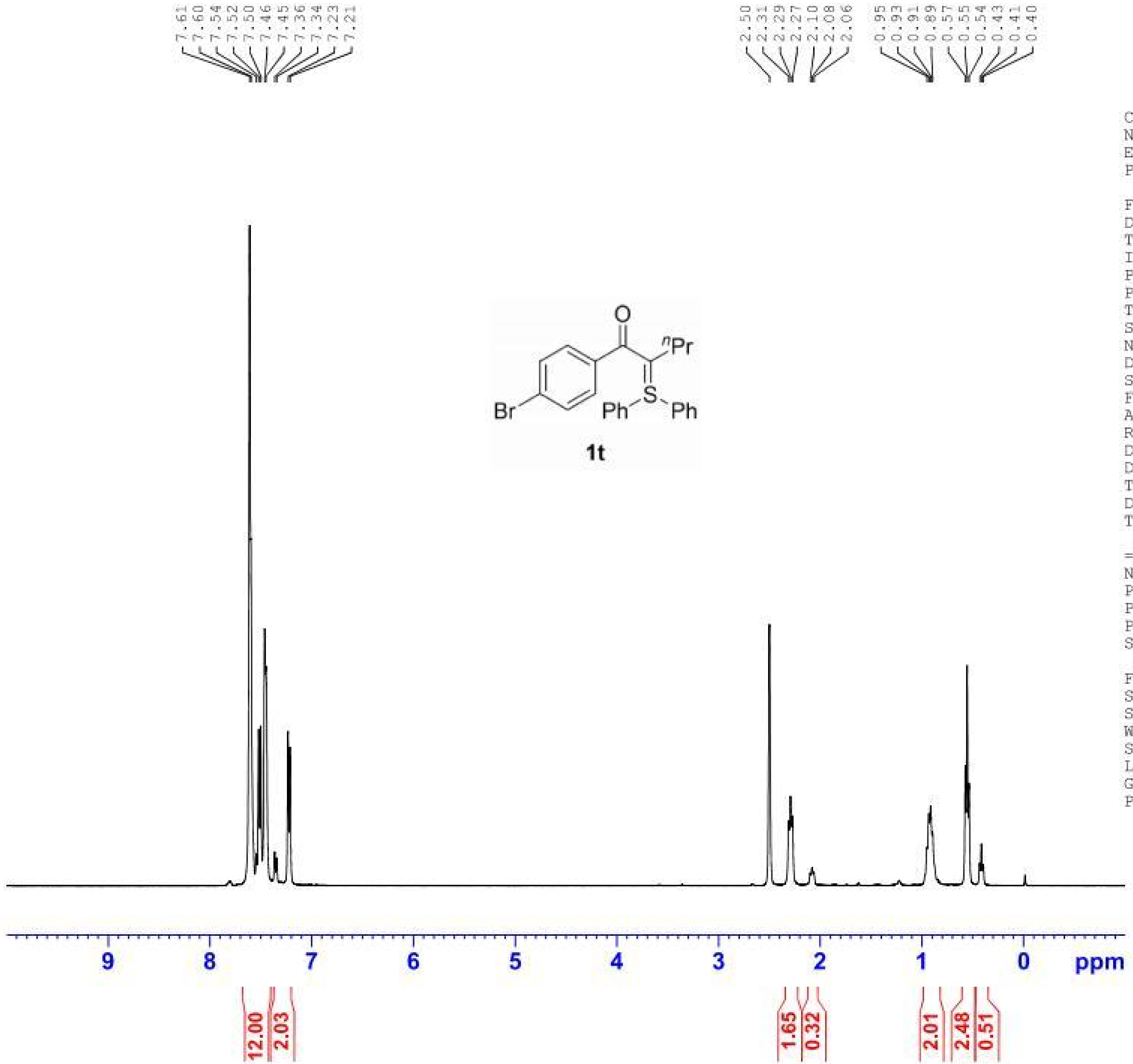


```
Current Data Parameters
NAME          qcx-6-111i
EXPNO                  1
PROCNO                 1

F2 - Acquisition Parameters
Date_           20230421
Time               20.34
INSTRUM            spect
PROBHD   5 mm DUL 13C-1
PULPROG             zg30
TD                 65536
SOLVENT             DMSO
NS                     3
DS                     2
SWH             8223.685 Hz
FIDRES          0.125483 Hz
AQ             3.9845889 sec
RG                   203
DW                60.800 usec
DE                  6.00 usec
TE                 292.6 K
D1            1.00000000 sec
TD0                    1

======== CHANNEL f1 ========
NUC1                  1H
P1                 15.80 usec
PL1                -1.00 dB
PL1W         12.17476940 W
SFO1         400.1324710 MHz

F2 - Processing parameters
SI                 32768
SF           400.1300041 MHz
WDW                   EM
SSB                    0
LB                  0.30 Hz
GB                     0
PC                  1.00
```

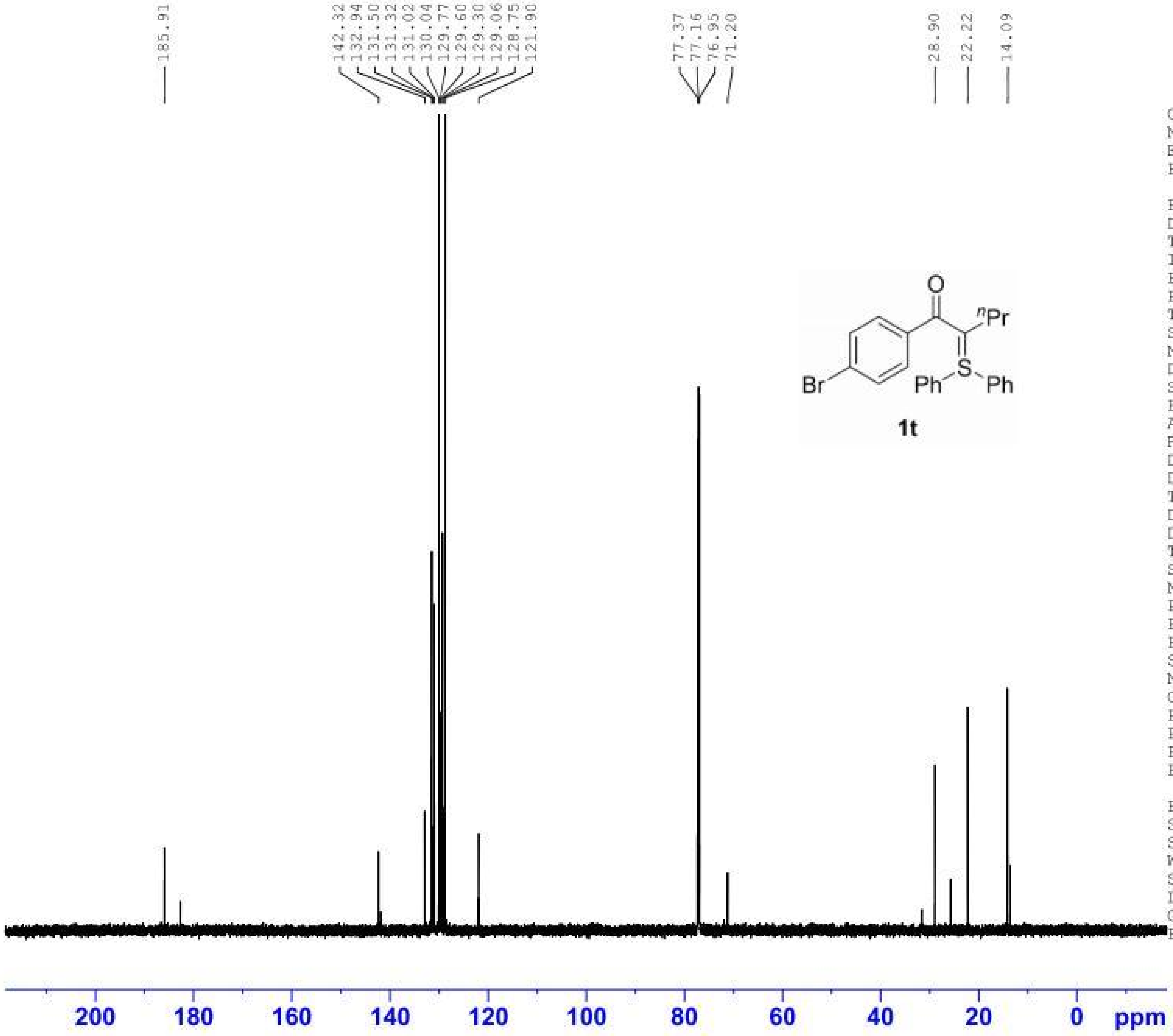

1t

```
Current Data Parameters
NAME              qcx-i
EXPNO                 1
PROCNO                1

F2 - Acquisition Parameters
Date_          20230528
Time              12.18 h
INSTRUM  Avance NEO 600
PROBHD   Z168773_0025 (
PULPROG          zgpg30
TD                65536
SOLVENT           CDCl3
NS                    8
DS                    4
SWH          35714.285 Hz
FIDRES        1.089913 Hz
AQ           0.9175040 sec
RG                 3.56
DW               14.000 usec
DE                 6.50 usec
TE                298.0 K
D1           2.00000000 sec
D11          0.03000000 sec
TD0                   1
SFO1        150.9355021 MHz
NUC1                13C
P0                 3.33 usec
P1                10.00 usec
PLW1        81.31300354 W
SFO2        600.2024008 MHz
NUC2                 1H
CPDPRG[2        waltz65
PCPD2             70.00 usec
PLW2        18.05400085 W
PLW12        0.53056997 W
PLW13        0.26686999 W

F2 - Processing parameters
SI                32768
SF          150.9204059 MHz
WDW                  EM
SSB                   0
LB                 1.00 Hz
GB                    0
PC                 1.40
```

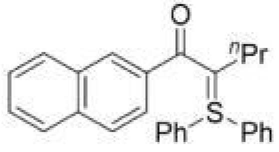

1u

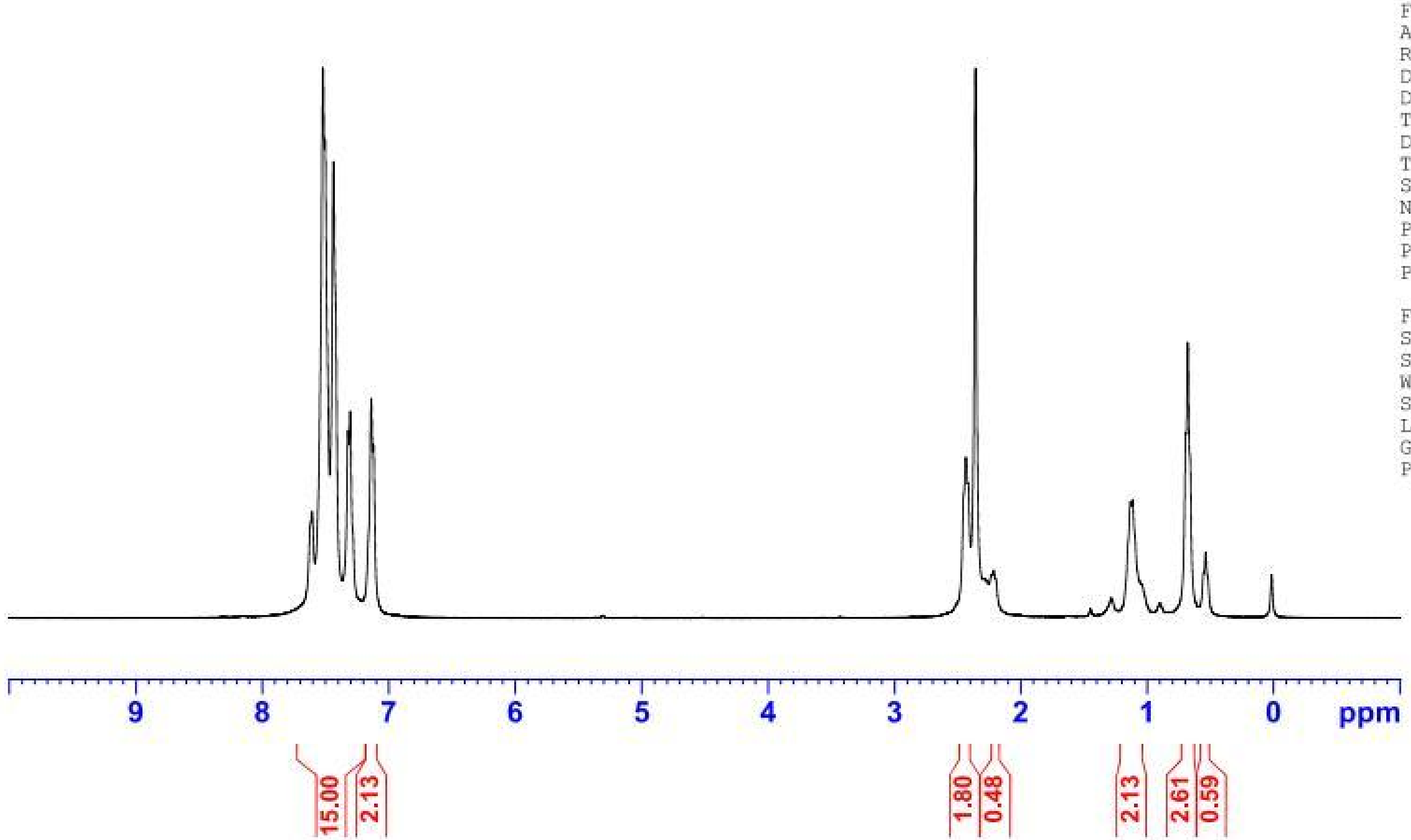

Current Data Parameters
NAME qcx-6-111n
EXPNO 1
PROCNO 1

F2 - Acquisition Parameters
Date_ 20230426
Time 22.21 h
INSTRUM AvanceNeo 400MHz
PROBHD Z163739_0629 (
PULPROG zg30
TD 65536
SOLVENT CDCl3
NS 5
DS 2
SWH 8196.722 Hz
FIDRES 0.250144 Hz
AQ 3.9976959 sec
RG 101
DW 61.000 usec
DE 13.89 usec
TE 297.6 K
D1 1.00000000 sec
TD0 1
SFO1 400.1824711 MHz
NUC1 1H
P0 2.67 usec
P1 8.00 usec
PLW1 21.26700020 W

F2 - Processing parameters
SI 65536
SF 400.1800000 MHz
WDW EM
SSB 0
LB 0.30 Hz
GB 0
PC 1.00

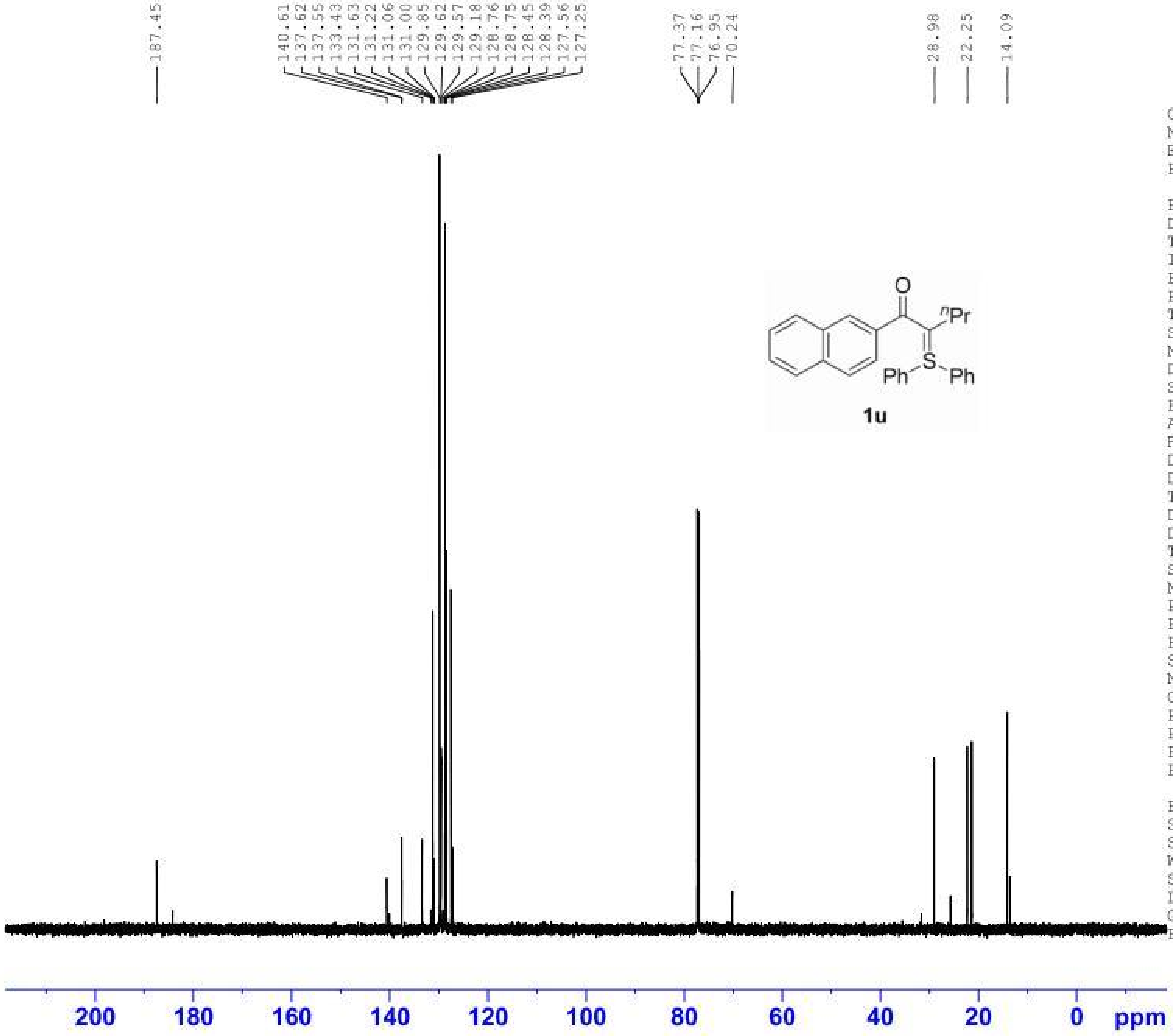

1u

```
Current Data Parameters
NAME                qcx-n
EXPNO                   1
PROCNO                  1

F2 - Acquisition Parameters
Date_            20230528
Time                12.32 h
INSTRUM    Avance NEO 600
PROBHD    Z168773_0025 (
PULPROG            zgpg30
TD                  65536
SOLVENT             CDCl3
NS                      7
DS                      4
SWH             35714.285 Hz
FIDRES           1.089913 Hz
AQ              0.9175040 sec
RG                      4
DW                 14.000 usec
DE                   6.50 usec
TE                  298.0 K
D1             2.00000000 sec
D11            0.03000000 sec
TD0                     1
SFO1          150.9355021 MHz
NUC1                  13C
P0                   3.33 usec
P1                  10.00 usec
PLW1          81.31300354 W
SFO2          600.2024008 MHz
NUC2                   1H
CPDPRG[2          waltz65
PCPD2               70.00 usec
PLW2          18.05400085 W
PLW12          0.53056997 W
PLW13          0.26686999 W

F2 - Processing parameters
SI                  32768
SF            150.9204118 MHz
WDW                    EM
SSB                     0
LB                   1.00 Hz
GB                      0
PC                   1.40
```

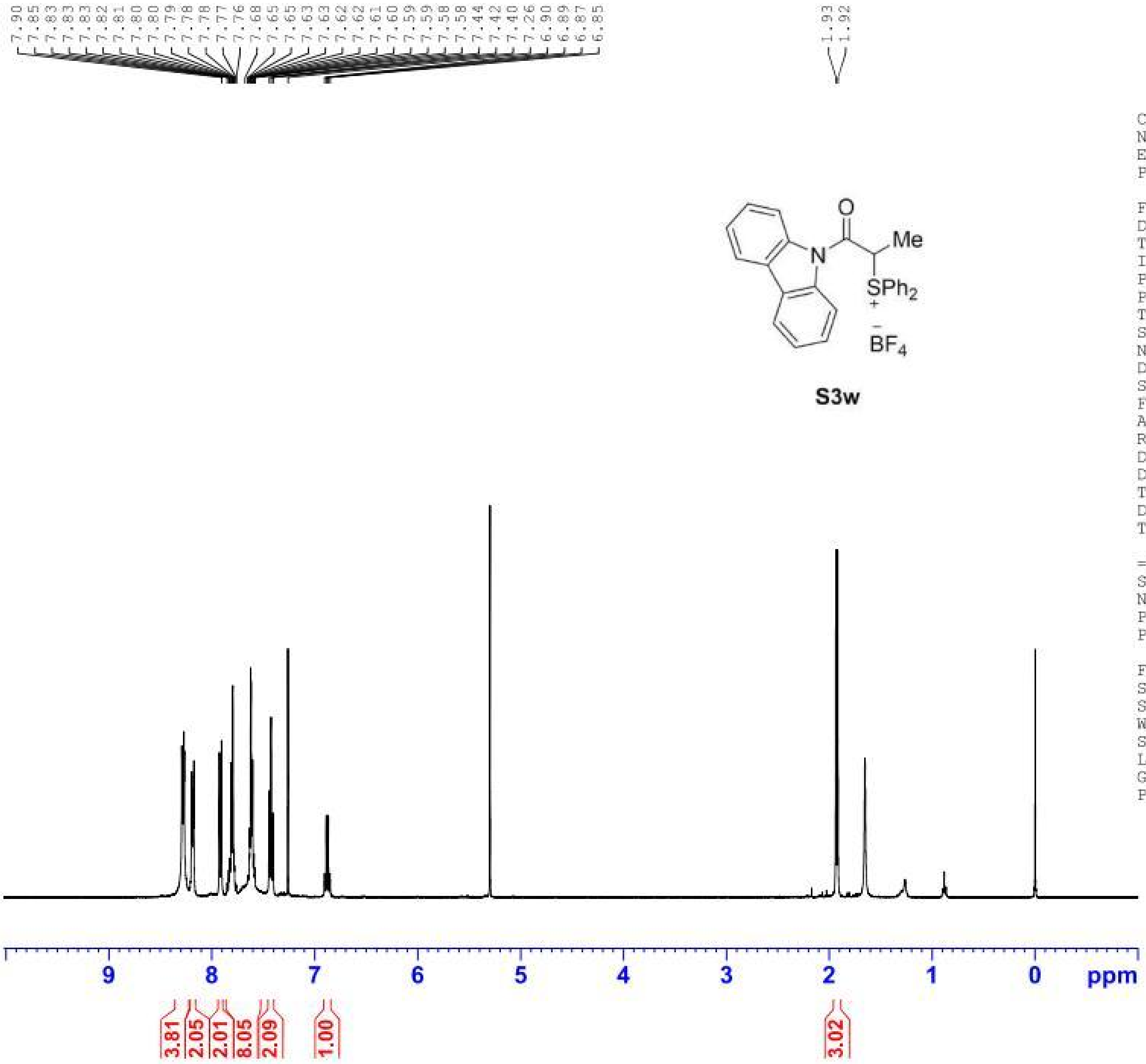

S3w

```
Current Data Parameters
NAME            qcx-6-111m
EXPNO                    1
PROCNO                   1

F2 - Acquisition Parameters
Date_             20230424
Time                 20.39
INSTRUM              spect
PROBHD    5 mm PABBO BB/
PULPROG               zg30
TD                   65536
SOLVENT              CDCl3
NS                       4
DS                       2
SWH               8012.820 Hz
FIDRES            0.122266 Hz
AQ               4.0894465 sec
RG                  112.31
DW                  62.400 usec
DE                    6.50 usec
TE                   296.3 K
D1              1.00000000 sec
TD0                      1

======== CHANNEL f1 ========
SFO1           400.1324710 MHz
NUC1                    1H
P1                   14.50 usec
PLW1           11.99499989 W

F2 - Processing parameters
SI                   65536
SF             400.1300101 MHz
WDW                     EM
SSB                      0
LB                    0.30 Hz
GB                       0
PC                    1.00
```

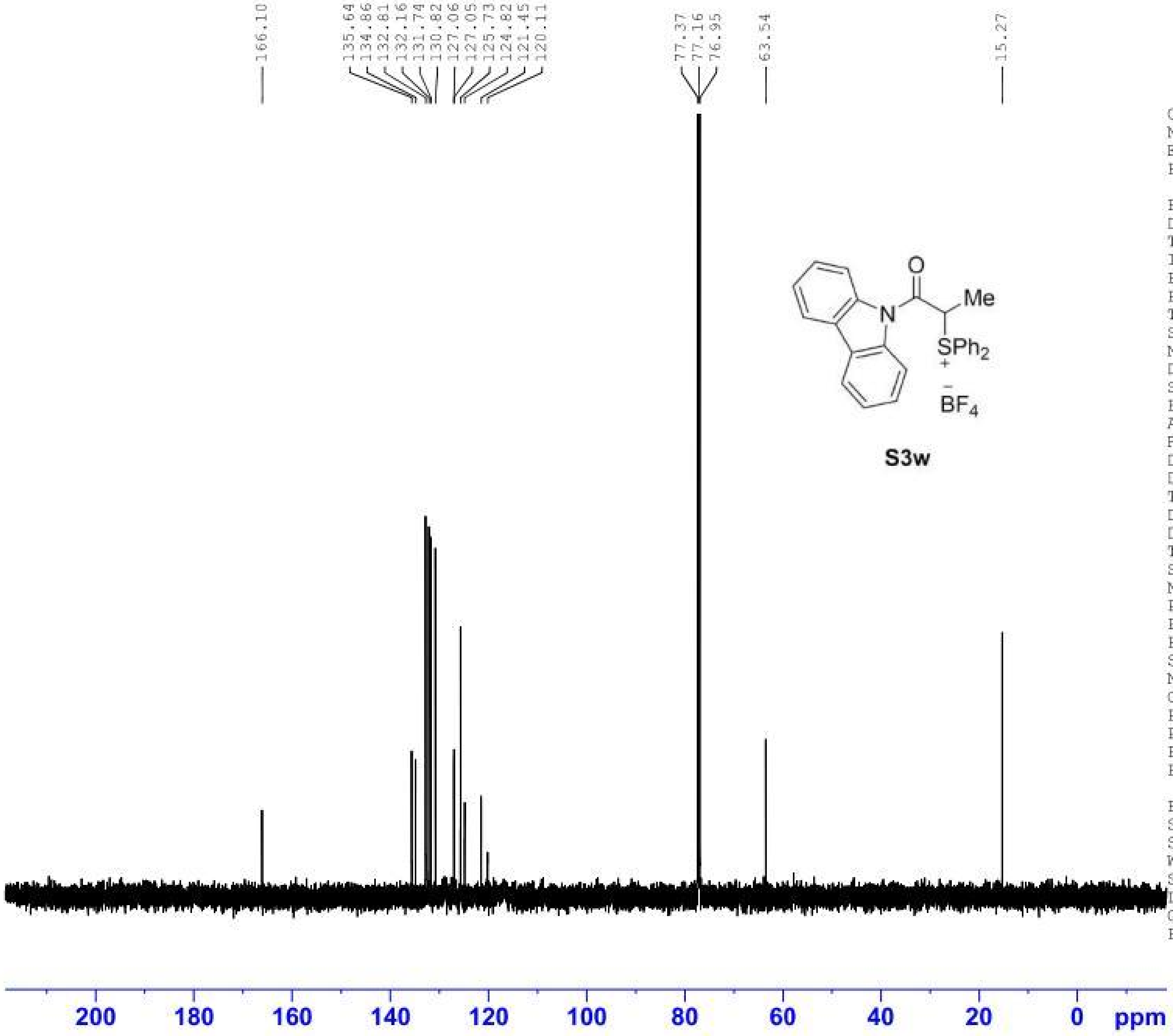

S3w

Current Data Parameters
NAME qcx-m
EXPNO 1
PROCNO 1

F2 - Acquisition Parameters
Date_ 20230528
Time 12.27 h
INSTRUM Avance NEO 600
PROBHD Z168773_0025 (
PULPROG zgpg30
TD 65536
SOLVENT CDCl3
NS 10
DS 4
SWH 35714.285 Hz
FIDRES 1.089913 Hz
AQ 0.9175040 sec
RG 3.56
DW 14.000 usec
DE 6.50 usec
TE 298.0 K
D1 2.00000000 sec
D11 0.03000000 sec
TD0 1
SFO1 150.9355021 MHz
NUC1 13C
P0 3.33 usec
P1 10.00 usec
PLW1 81.31300354 W
SFO2 600.2024008 MHz
NUC2 1H
CPDPRG[2 waltz65
PCPD2 70.00 usec
PLW2 18.05400085 W
PLW12 0.53056997 W
PLW13 0.26686999 W

F2 - Processing parameters
SI 32768
SF 150.9203945 MHz
WDW EM
SSB 0
LB 1.00 Hz
GB 0
PC 1.40

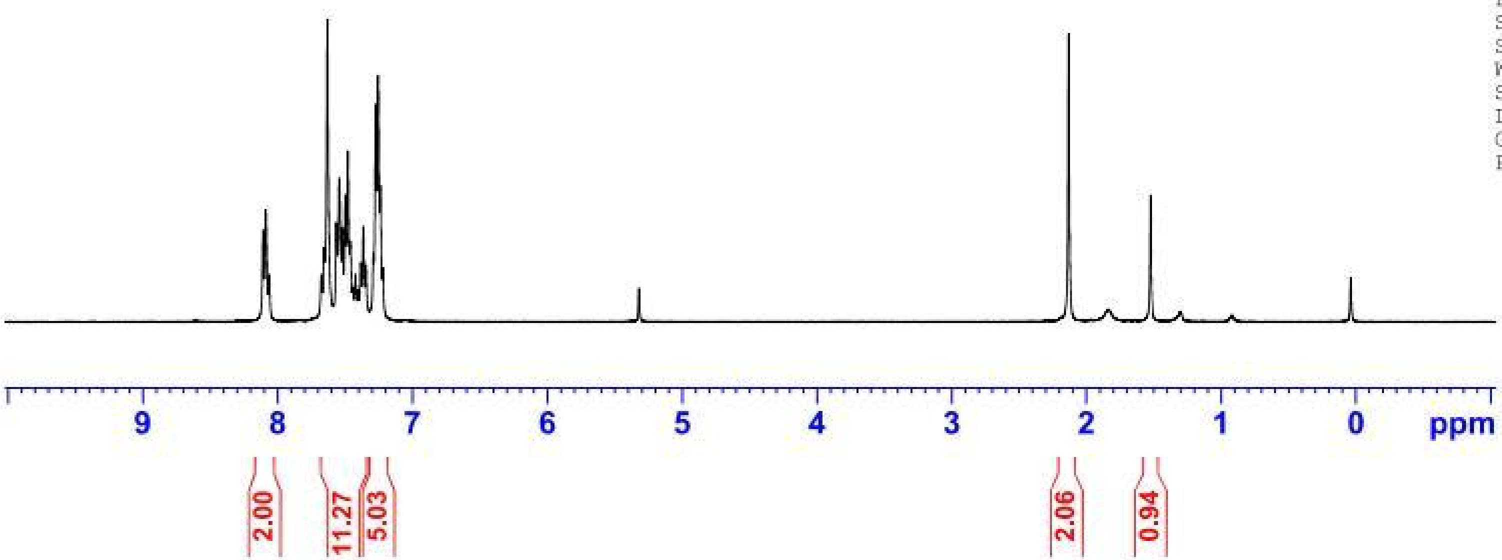

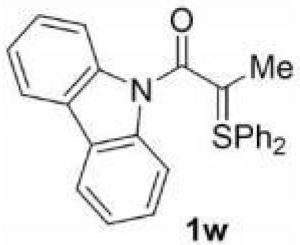

1w

Current Data Parameters
NAME qcx-6-111h-new
EXPNO 1
PROCNO 1

F2 - Acquisition Parameters
Date_ 20230424
Time 20.27 h
INSTRUM AvanceNeo 400MHz
PROBHD Z163739_0629 (
PULPROG zg30
TD 65536
SOLVENT CDCl3
NS 4
DS 2
SWH 8196.722 Hz
FIDRES 0.250144 Hz
AQ 3.9976959 sec
RG 101
DW 61.000 usec
DE 13.89 usec
TE 297.4 K
D1 1.00000000 sec
TD0 1
SFO1 400.1824711 MHz
NUC1 1H
P0 2.67 usec
P1 8.00 usec
PLW1 21.26700020 W

F2 - Processing parameters
SI 65536
SF 400.1799968 MHz
WDW EM
SSB 0
LB 0.30 Hz
GB 0
PC 1.00

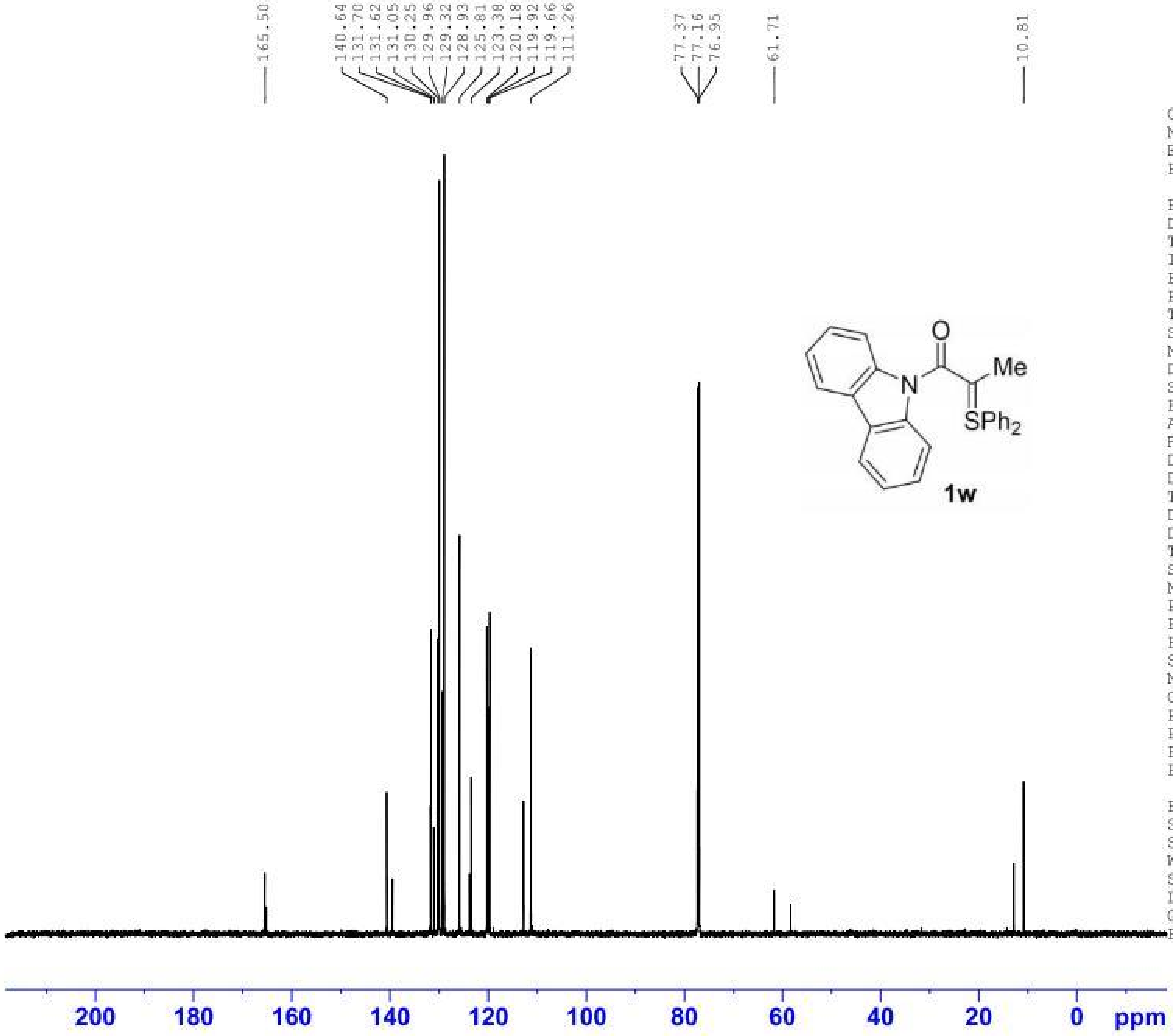

Current Data Parameters
NAME qcx-h
EXPNO 2
PROCNO 1

F2 - Acquisition Parameters
Date_ 20230528
Time 12.14 h
INSTRUM Avance NEO 600
PROBHD Z168773_0025 (
PULPROG zgpg30
TD 65536
SOLVENT CDCl3
NS 55
DS 4
SWH 35714.285 Hz
FIDRES 1.089913 Hz
AQ 0.9175040 sec
RG 4
DW 14.000 usec
DE 6.50 usec
TE 298.0 K
D1 2.00000000 sec
D11 0.03000000 sec
TD0 1
SFO1 150.9355021 MHz
NUC1 13C
P0 3.33 usec
P1 10.00 usec
PLW1 81.31300354 W
SFO2 600.2024008 MHz
NUC2 1H
CPDPRG[2 waltz65
PCPD2 70.00 usec
PLW2 18.05400085 W
PLW12 0.53056997 W
PLW13 0.26686999 W

F2 - Processing parameters
SI 32768
SF 150.9204064 MHz
WDW EM
SSB 0
LB 1.00 Hz
GB 0
PC 1.40

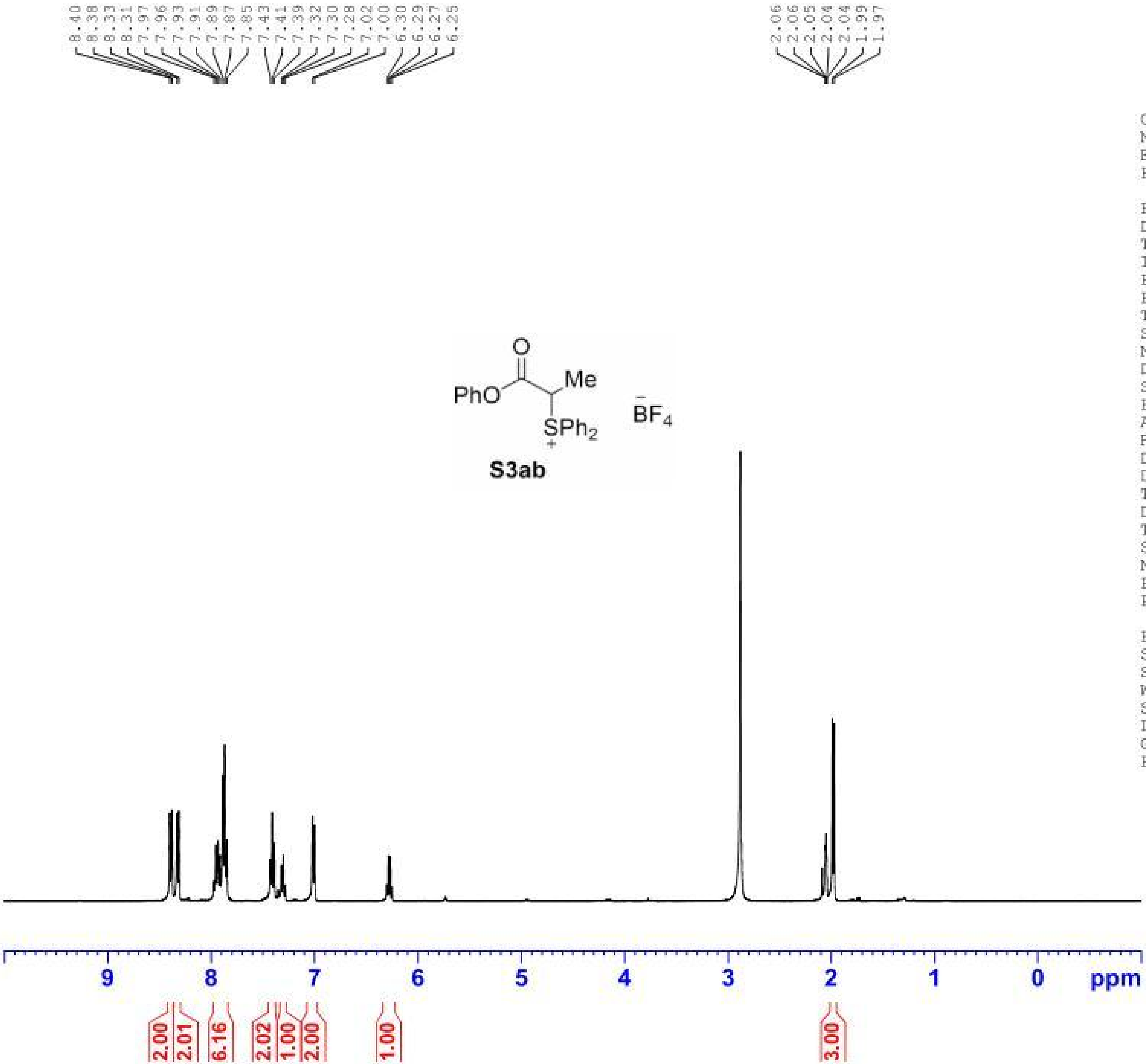

Current Data Parameters
NAME qcx-9-203-p
EXPNO 1
PROCNO 1

F2 - Acquisition Parameters
Date_ 20250722
Time 16.40 h
INSTRUM spect
PROBHD Z116098_0761 (
PULPROG zg30
TD 65536
SOLVENT Acetone
NS 11
DS 2
SWH 8012.820 Hz
FIDRES 0.244532 Hz
AQ 4.0894465 sec
RG 78.76
DW 62.400 usec
DE 6.50 usec
TE 299.7 K
D1 1.00000000 sec
TD0 1
SFO1 400.1324708 MHz
NUC1 1H
P1 10.00 usec
PLW1 16.24399948 W

F2 - Processing parameters
SI 65536
SF 400.1300066 MHz
WDW EM
SSB 0
LB 0.30 Hz
GB 0
PC 1.00

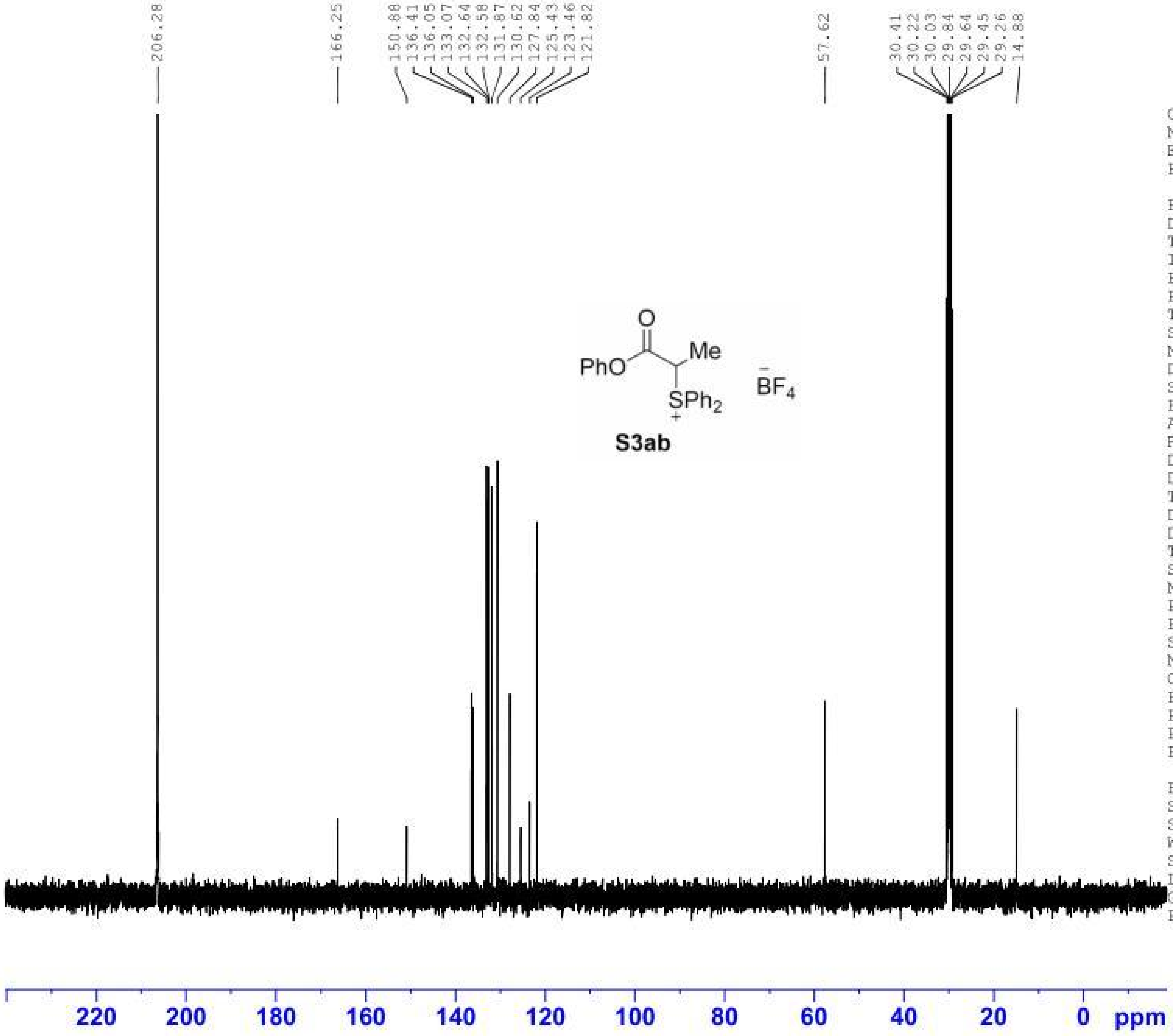

Current Data Parameters
NAME qcx-9-203-p
EXPNO 2
PROCNO 1

F2 - Acquisition Parameters
Date_ 20250722
Time 16.56 h
INSTRUM spect
PROBHD Z116098_0761 (
PULPROG zgpg30
TD 65536
SOLVENT Acetone
NS 260
DS 4
SWH 26041.666 Hz
FIDRES 0.794729 Hz
AQ 1.2582912 sec
RG 198.89
DW 19.200 usec
DE 6.50 usec
TE 300.2 K
D1 2.00000000 sec
D11 0.03000000 sec
TD0 1
SFO1 100.6238359 MHz
NUC1 13C
P1 10.00 usec
PLW1 79.28600311 W
SFO2 400.1316005 MHz
NUC2 1H
CPDPRG[2 waltz16
PCPD2 90.00 usec
PLW2 16.24399948 W
PLW12 0.20054001 W
PLW13 0.10087000 W

F2 - Processing parameters
SI 32768
SF 100.6126801 MHz
WDW EM
SSB 0
LB 1.00 Hz
GB 0
PC 1.40

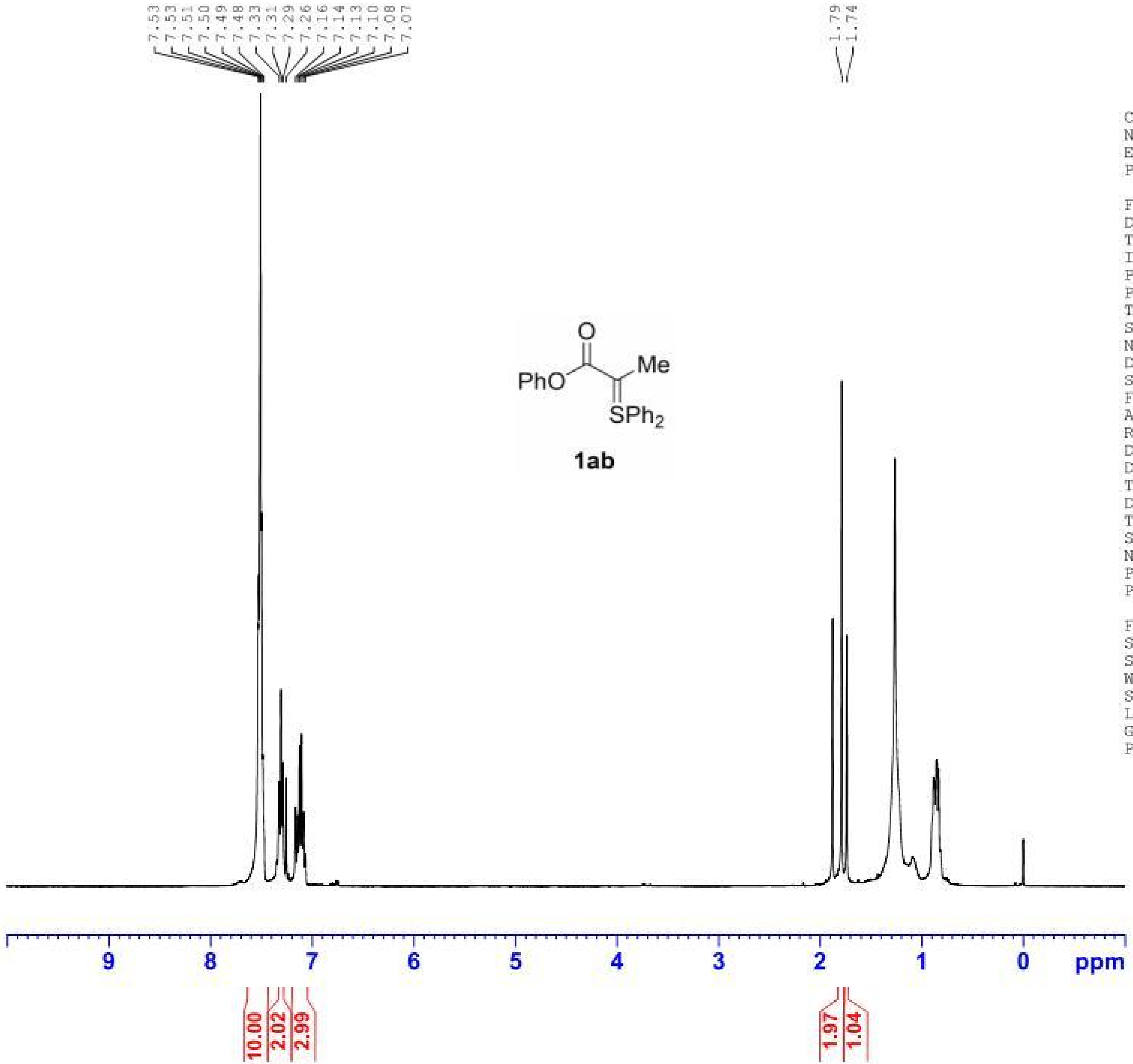

Current Data Parameters
NAME qcx-9-206-p
EXPNO 1
PROCNO 1

F2 - Acquisition Parameters
Date_ 20250722
Time 16.23 h
INSTRUM spect
PROBHD Z116098_0761 (
PULPROG zg30
TD 65536
SOLVENT CDCl3
NS 16
DS 2
SWH 8012.820 Hz
FIDRES 0.244532 Hz
AQ 4.0894465 sec
RG 55.51
DW 62.400 usec
DE 6.50 usec
TE 299.7 K
D1 1.00000000 sec
TD0 1
SFO1 400.1324708 MHz
NUC1 1H
P1 10.00 usec
PLW1 16.24399948 W

F2 - Processing parameters
SI 65536
SF 400.1300109 MHz
WDW EM
SSB 0
LB 0.30 Hz
GB 0
PC 1.00

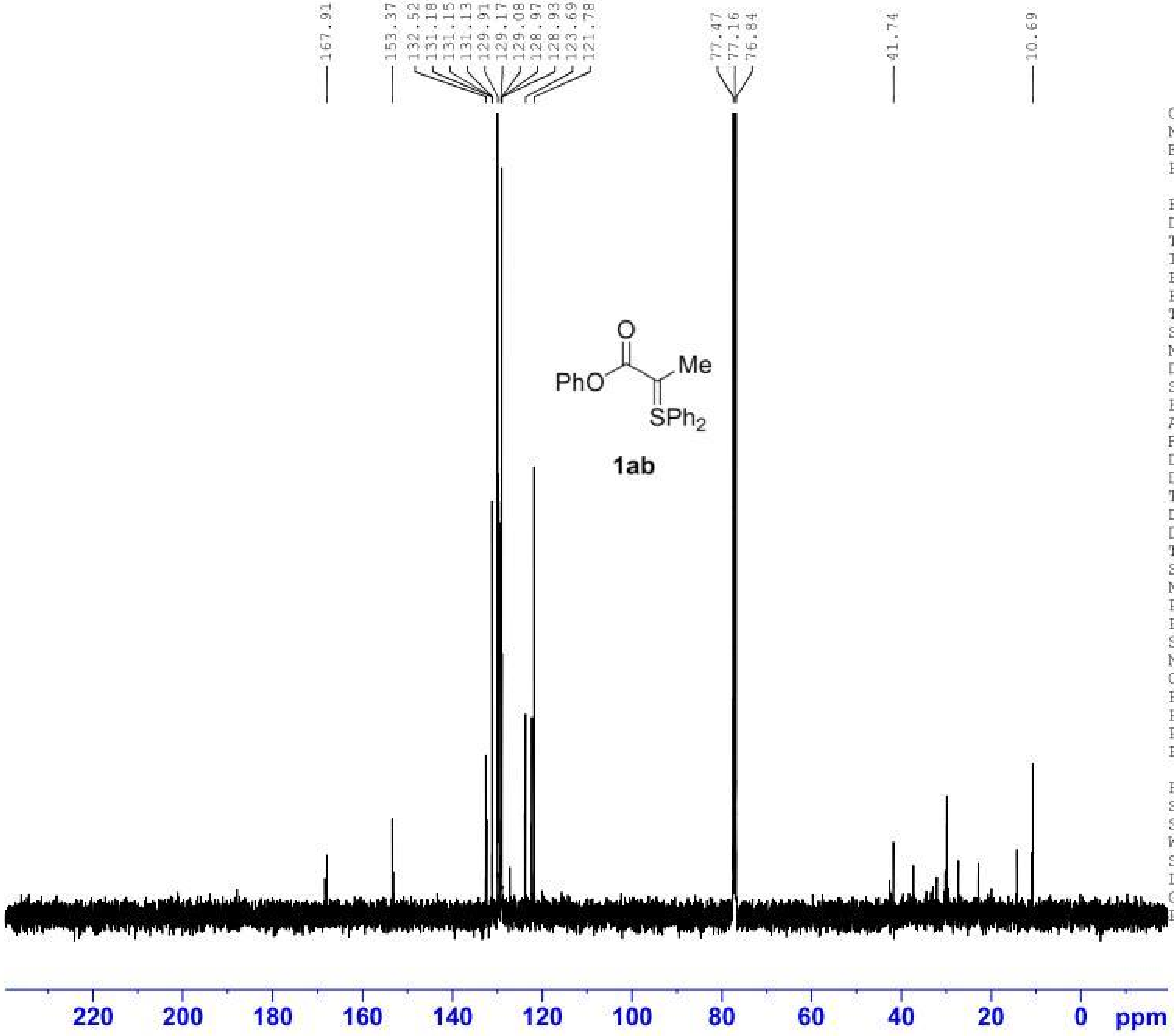

Current Data Parameters
NAME qcx-9-206-p
EXPNO 2
PROCNO 1

F2 - Acquisition Parameters
Date_ 20250722
Time 16.36 h
INSTRUM spect
PROBHD Z116098_0761 (
PULPROG zgpg30
TD 65536
SOLVENT CDCl3
NS 222
DS 4
SWH 26041.666 Hz
FIDRES 0.794729 Hz
AQ 1.2582912 sec
RG 198.89
DW 19.200 usec
DE 6.50 usec
TE 300.2 K
D1 2.00000000 sec
D11 0.03000000 sec
TD0 1
SFO1 100.6238359 MHz
NUC1 13C
P1 10.00 usec
PLW1 79.28600311 W
SFO2 400.1316005 MHz
NUC2 1H
CPDPRG[2 waltz16
PCPD2 90.00 usec
PLW2 16.24399948 W
PLW12 0.20054001 W
PLW13 0.10087000 W

F2 - Processing parameters
SI 32768
SF 100.6127579 MHz
WDW EM
SSB 0
LB 1.00 Hz
GB 0
PC 1.40

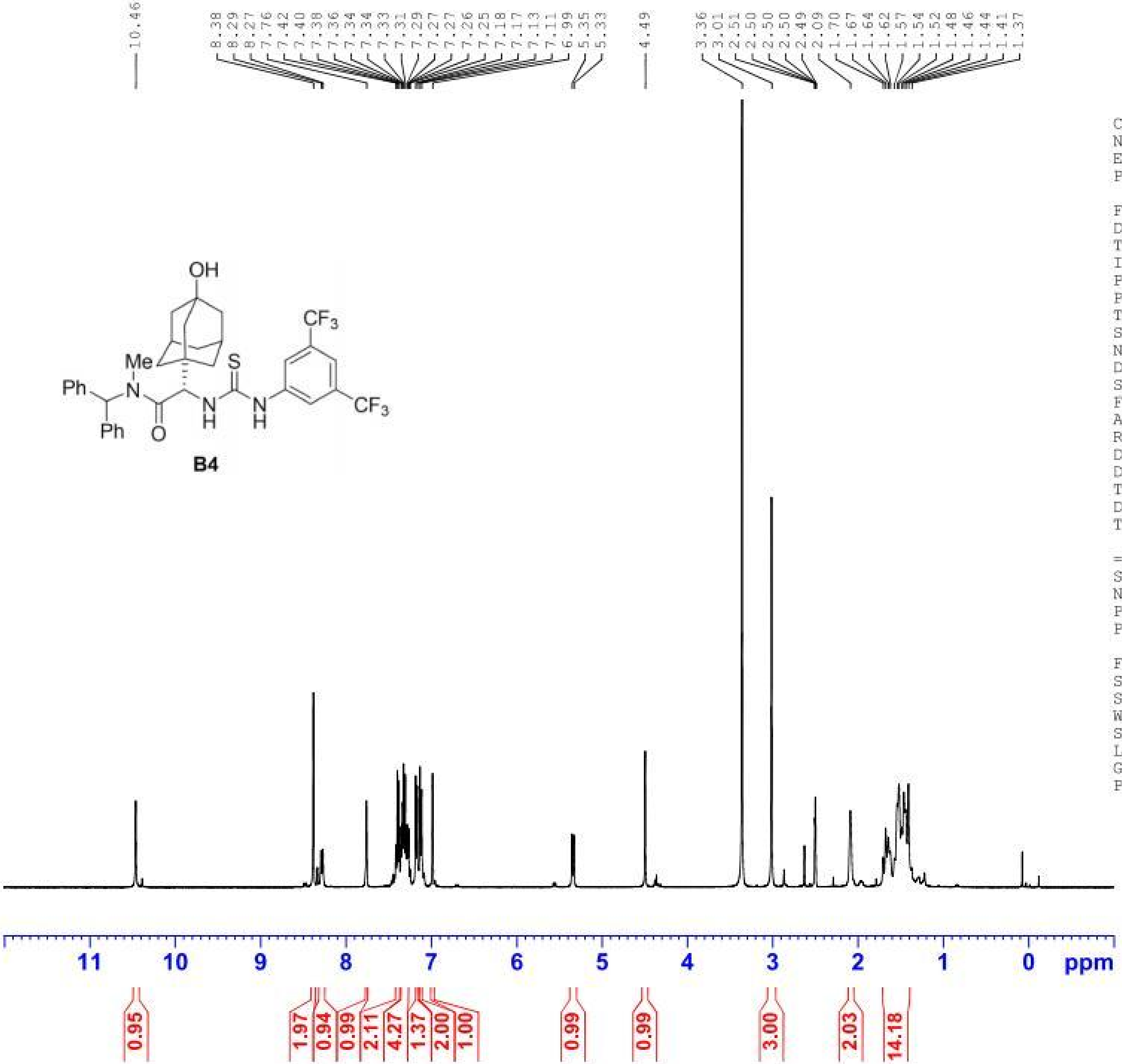


```
Current Data Parameters
NAME       qcx-6-136e-dmso
EXPNO                    1
PROCNO                   1

F2 - Acquisition Parameters
Date_             20230531
Time                 20.11
INSTRUM              spect
PROBHD    5 mm PABBO BB/
PULPROG               zg30
TD                   65536
SOLVENT               DMSO
NS                       5
DS                       2
SWH              8012.820 Hz
FIDRES           0.122266 Hz
AQ              4.0894465 sec
RG                   49.32
DW                  62.400 usec
DE                    6.50 usec
TE                   296.3 K
D1              1.00000000 sec
TD0                      1

======== CHANNEL f1 ========
SFO1         400.1324710 MHz
NUC1                    1H
P1                   14.50 usec
PLW1         11.99499989 W

F2 - Processing parameters
SI                   65536
SF           400.1300043 MHz
WDW                     EM
SSB                      0
LB                    0.30 Hz
GB                       0
PC                    1.00
```

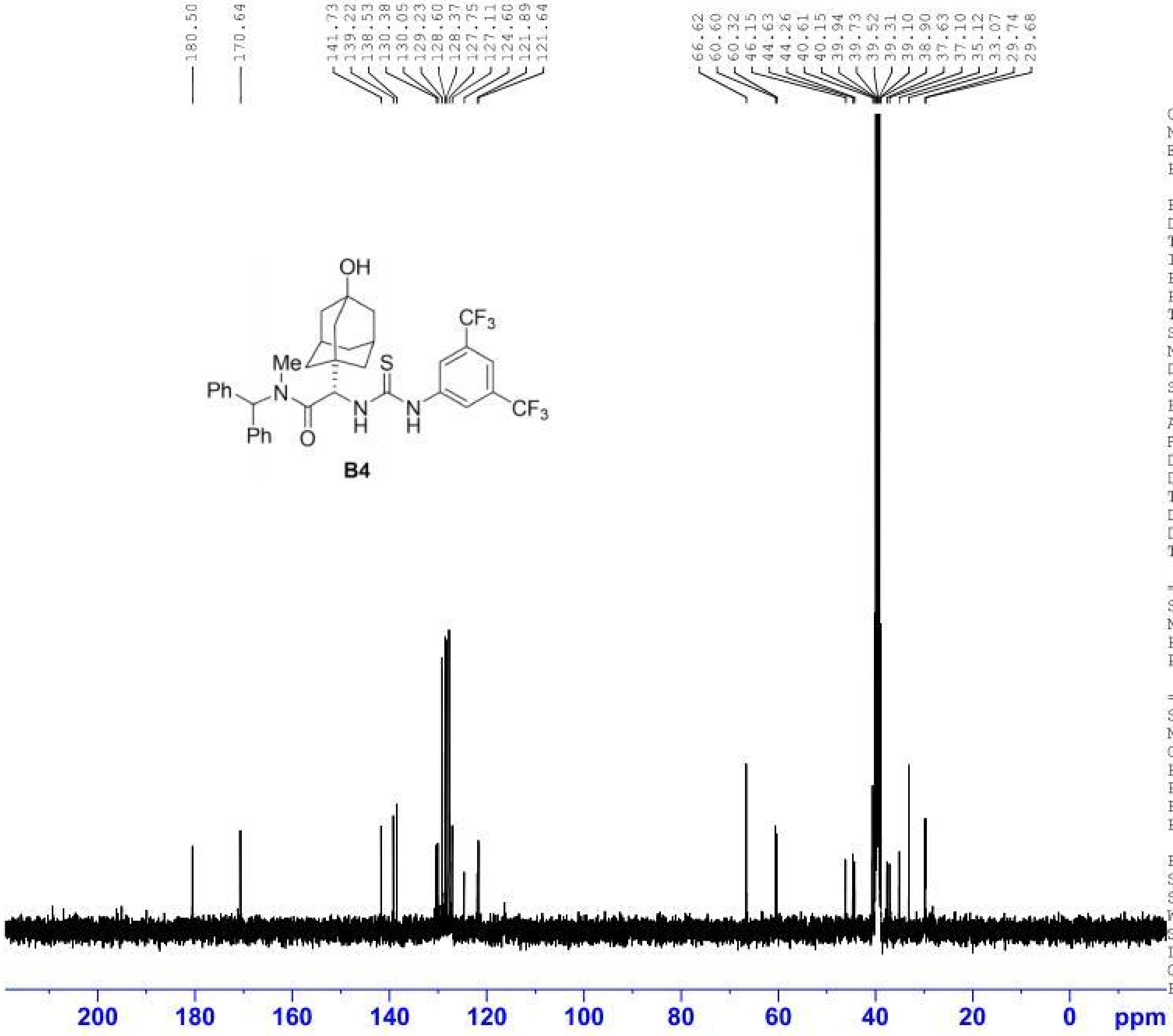

B4

Current Data Parameters
NAME qcx-6-136e-dmso
EXPNO 2
PROCNO 1

F2 - Acquisition Parameters
Date_ 20230531
Time 20.13
INSTRUM spect
PROBHD 5 mm PABBO BB/
PULPROG zgpg30
TD 65536
SOLVENT DMSO
NS 158
DS 2
SWH 24038.461 Hz
FIDRES 0.366798 Hz
AQ 1.3631488 sec
RG 196.92
DW 20.800 usec
DE 6.50 usec
TE 296.9 K
D1 2.00000000 sec
D11 0.03000000 sec
TD0 1

======== CHANNEL f1 ========
SFO1 100.6228298 MHz
NUC1 13C
P1 9.70 usec
PLW1 46.98899841 W

======== CHANNEL f2 ========
SFO2 400.1316005 MHz
NUC2 1H
CPDPRG[2 waltz16
PCPD2 90.00 usec
PLW2 11.99499989 W
PLW12 0.34213999 W
PLW13 0.27713001 W

F2 - Processing parameters
SI 32768
SF 100.6128139 MHz
WDW EM
SSB 0
LB 1.00 Hz
GB 0
PC 1.40

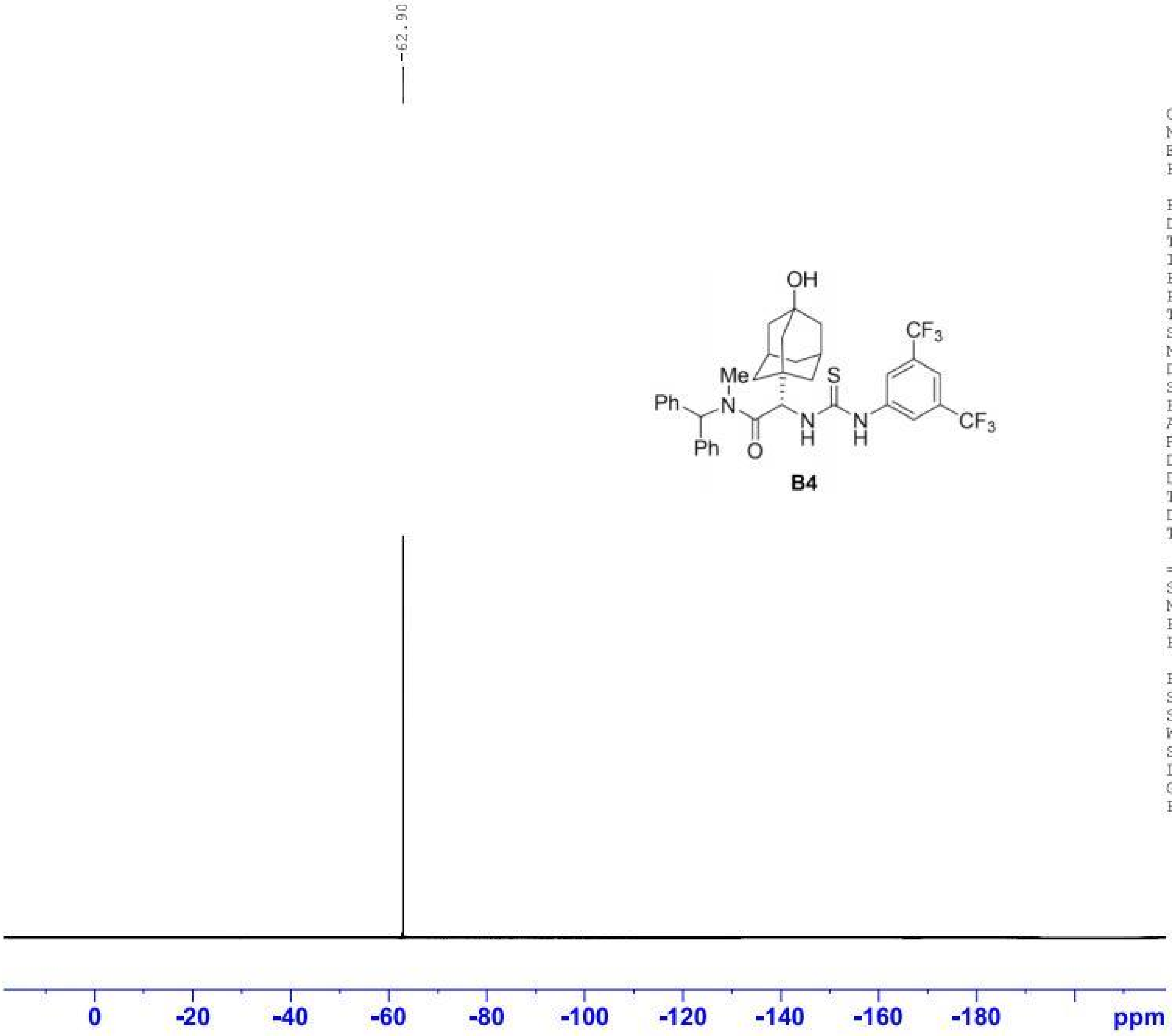

B4

```
Current Data Parameters
NAME          qcx-6-136e
EXPNO                  6
PROCNO                 1

F2 - Acquisition Parameters
Date_           20230530
Time               19.56
INSTRUM            spect
PROBHD   5 mm PABBO BB/
PULPROG           zgflqn
TD                131072
SOLVENT            CDCl3
NS                    16
DS                     4
SWH           89285.711 Hz
FIDRES         0.681196 Hz
AQ            0.7340032 sec
RG                196.92
DW                 5.600 usec
DE                  6.50 usec
TE                 296.5 K
D1            1.00000000 sec
TD0                    1

======== CHANNEL f1 ========
SFO1         376.4607164 MHz
NUC1                 19F
P1                 14.70 usec
PLW1         15.99600029 W

F2 - Processing parameters
SI                 65536
SF           376.4983660 MHz
WDW                   EM
SSB                    0
LB                  0.30 Hz
GB                     0
PC                  1.00
```

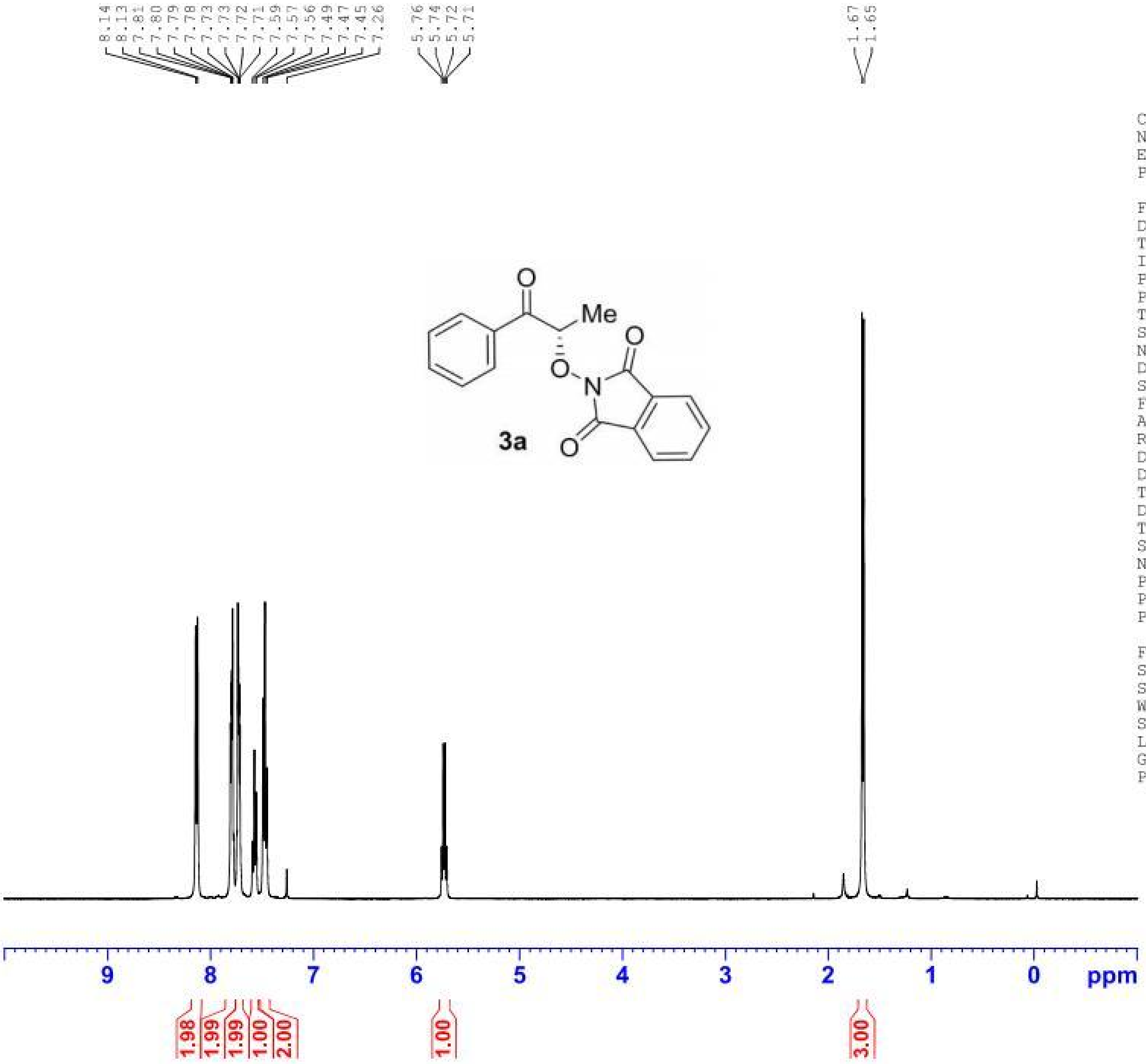

Current Data Parameters
NAME qcx-6-35a
EXPNO 1
PROCNO 1

F2 - Acquisition Parameters
Date_ 20230223
Time 20.12 h
INSTRUM AvanceNeo 400MHz
PROBHD Z163739_0629 (
PULPROG zg30
TD 65536
SOLVENT CDCl3
NS 4
DS 2
SWH 8196.722 Hz
FIDRES 0.250144 Hz
AQ 3.9976959 sec
RG 101
DW 61.000 usec
DE 13.89 usec
TE 294.6 K
D1 1.00000000 sec
TD0 1
SFO1 400.1824711 MHz
NUC1 1H
P0 2.67 usec
P1 8.00 usec
PLW1 21.26700020 W

F2 - Processing parameters
SI 65536
SF 400.1800089 MHz
WDW EM
SSB 0
LB 0.30 Hz
GB 0
PC 1.00

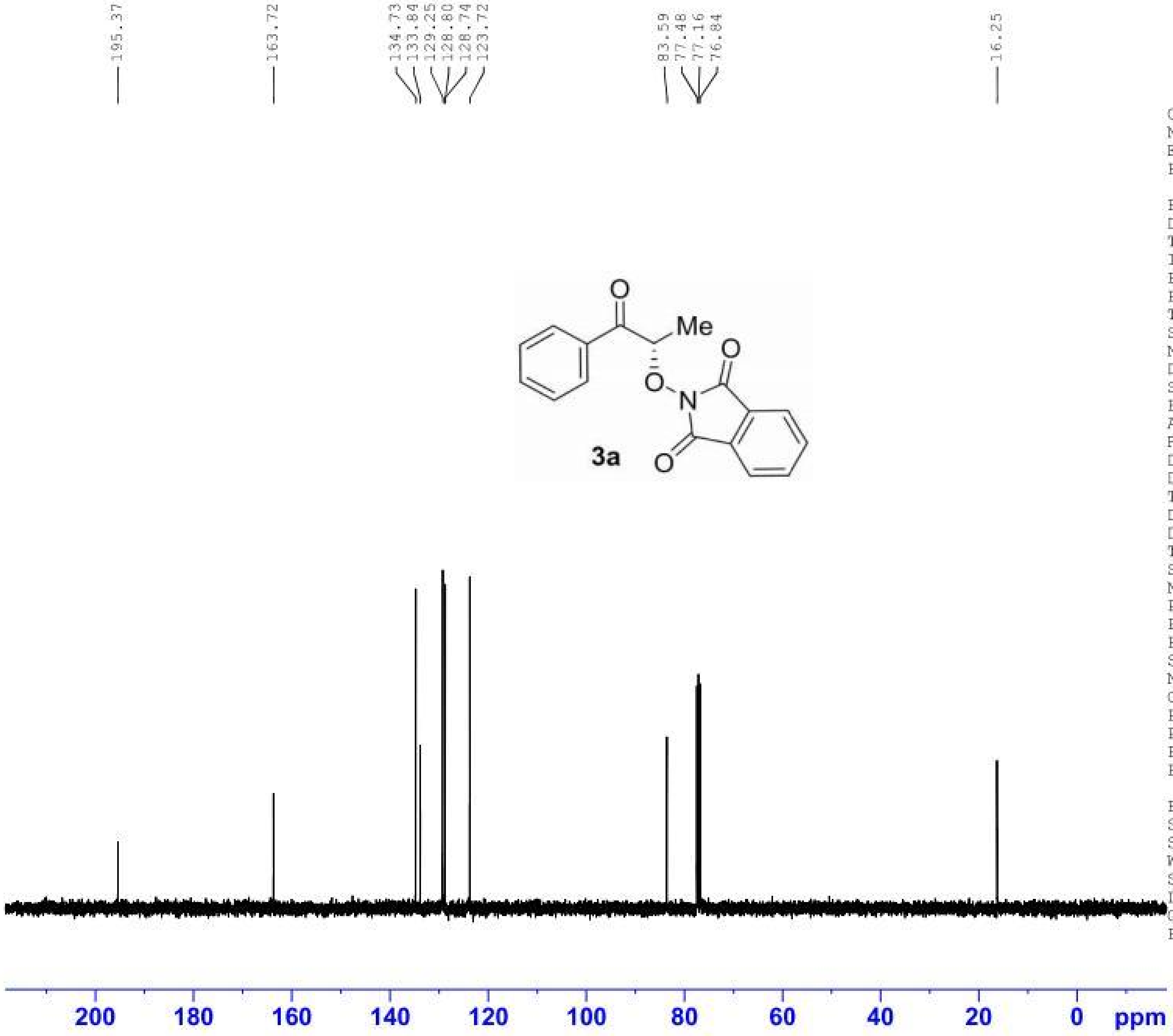

Current Data Parameters
NAME qcx-6-35a
EXPNO 2
PROCNO 1

F2 - Acquisition Parameters
Date_ 20230223
Time 20.16 h
INSTRUM AvanceNeo 400MHz
PROBHD Z163739_0629 (
PULPROG zgpg30
TD 65536
SOLVENT CDCl3
NS 26
DS 4
SWH 23809.523 Hz
FIDRES 0.726609 Hz
AQ 1.3762560 sec
RG 10
DW 21.000 usec
DE 6.50 usec
TE 294.9 K
D1 2.00000000 sec
D11 0.03000000 sec
TD0 1
SFO1 100.6354036 MHz
NUC1 13C
P0 2.67 usec
P1 8.00 usec
PLW1 85.25399780 W
SFO2 400.1816007 MHz
NUC2 1H
CPDPRG[2 waltz65
PCPD2 90.00 usec
PLW2 21.26700020 W
PLW12 0.16802999 W
PLW13 0.08452000 W

F2 - Processing parameters
SI 32768
SF 100.6253378 MHz
WDW EM
SSB 0
LB 1.00 Hz
GB 0
PC 1.40

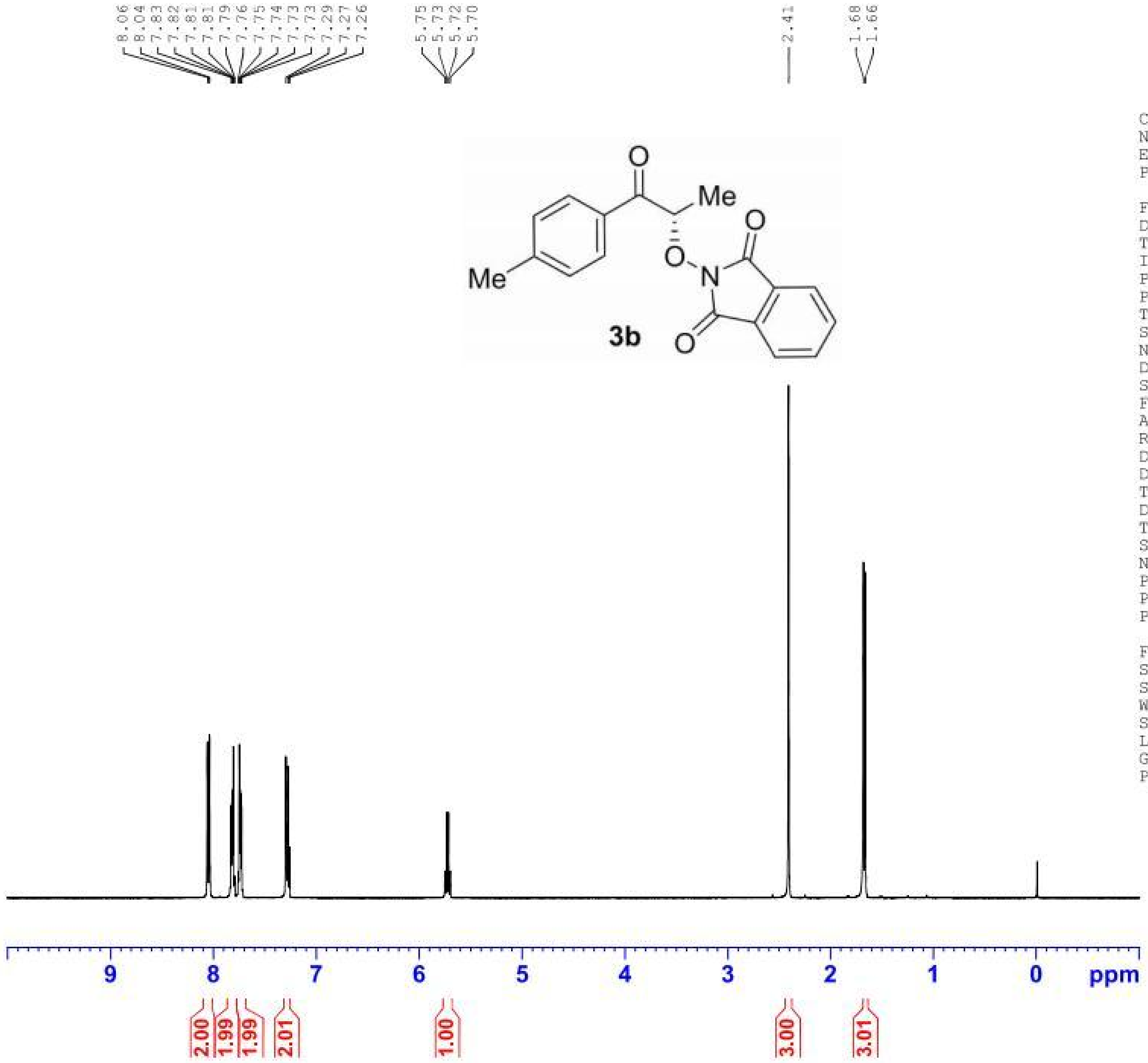


```
Current Data Parameters
NAME          qcx-6-48d
EXPNO                 1
PROCNO                1

F2 - Acquisition Parameters
Date_          20230308
Time              19.51 h
INSTRUM  AvanceNeo 400MHz
PROBHD   Z163739_0629 (
PULPROG            zg30
TD                65536
SOLVENT           CDCl3
NS                    4
DS                    2
SWH            8196.722 Hz
FIDRES         0.250144 Hz
AQ            3.9976959 sec
RG                  101
DW               61.000 usec
DE                13.89 usec
TE                296.1 K
D1           1.00000000 sec
TD0                   1
SFO1        400.1824711 MHz
NUC1                 1H
P0                 2.67 usec
P1                 8.00 usec
PLW1        21.26700020 W

F2 - Processing parameters
SI                65536
SF          400.1800095 MHz
WDW                  EM
SSB                   0
LB                 0.30 Hz
GB                    0
PC                 1.00
```

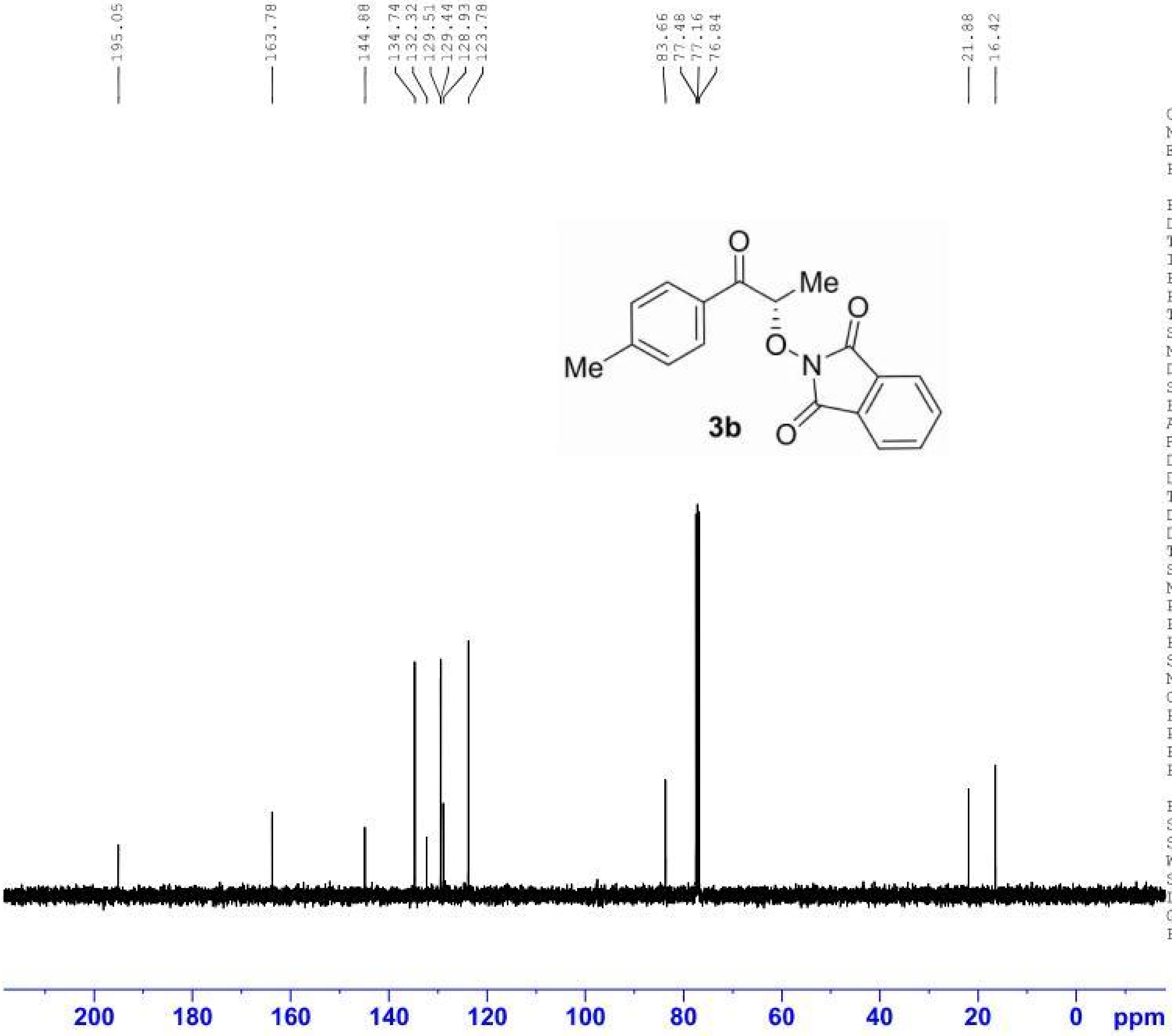


```
Current Data Parameters
NAME              qcx-6-48d
EXPNO                     2
PROCNO                    1

F2 - Acquisition Parameters
Date_              20230308
Time                  19.54 h
INSTRUM    AvanceNeo 400MHz
PROBHD     Z163739_0629 (
PULPROG             zgpg30
TD                    65536
SOLVENT               CDCl3
NS                       20
DS                        4
SWH               23809.523 Hz
FIDRES             0.726609 Hz
AQ                1.3762560 sec
RG                       10
DW                   21.000 usec
DE                     6.50 usec
TE                    296.4 K
D1               2.00000000 sec
D11              0.03000000 sec
TD0                       1
SFO1            100.6354036 MHz
NUC1                    13C
P0                     2.67 usec
P1                     8.00 usec
PLW1            85.25399780 W
SFO2            400.1816007 MHz
NUC2                     1H
CPDPRG[2            waltz65
PCPD2                 90.00 usec
PLW2            21.26700020 W
PLW12            0.16802999 W
PLW13            0.08452000 W

F2 - Processing parameters
SI                    32768
SF              100.6253311 MHz
WDW                      EM
SSB                       0
LB                     1.00 Hz
GB                        0
PC                     1.40
```

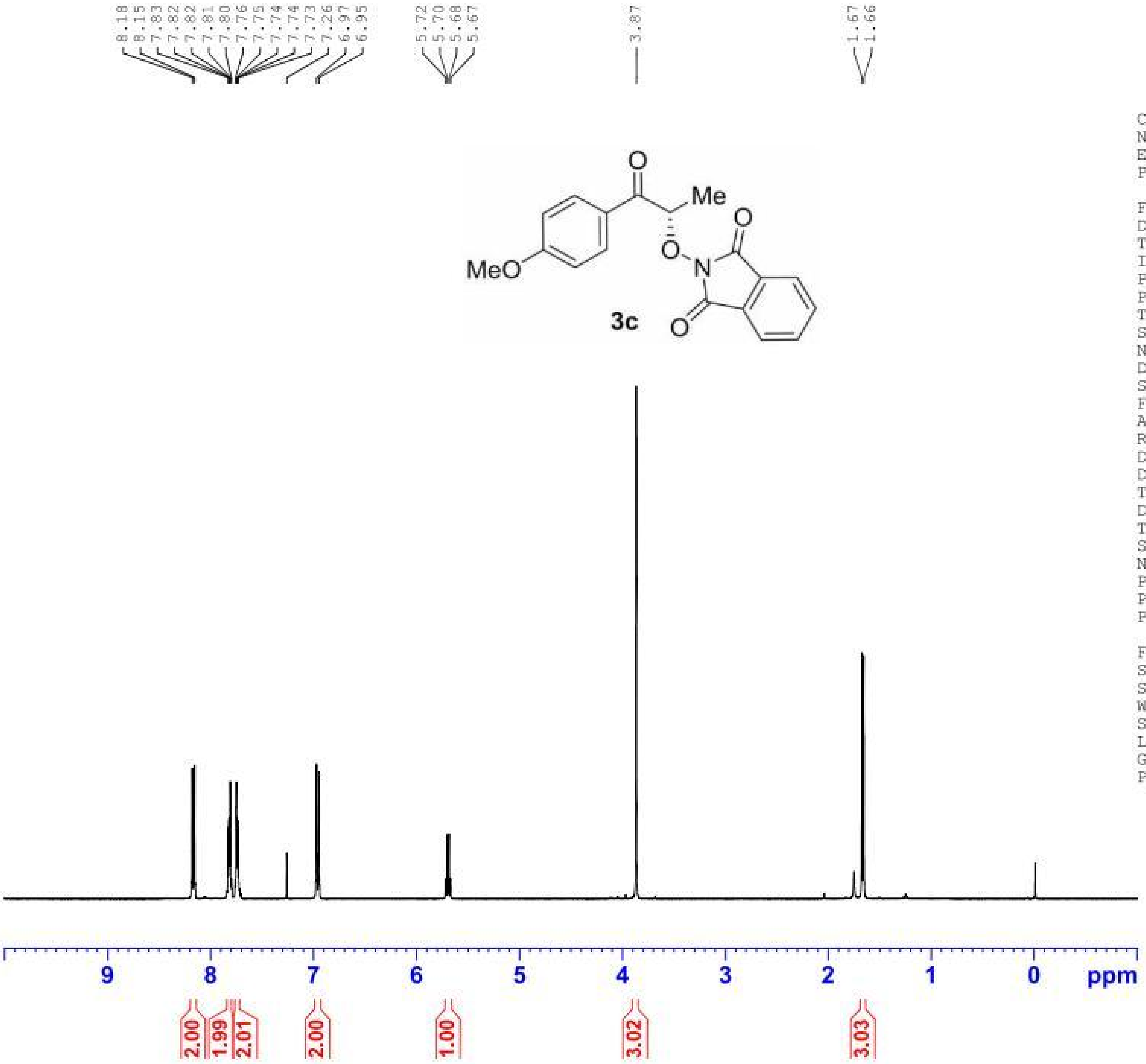

Current Data Parameters
NAME qcx-6-48c
EXPNO 1
PROCNO 1

F2 - Acquisition Parameters
Date_ 20230308
Time 19.27 h
INSTRUM AvanceNeo 400MHz
PROBHD Z163739_0629 (
PULPROG zg30
TD 65536
SOLVENT CDCl3
NS 4
DS 2
SWH 8196.722 Hz
FIDRES 0.250144 Hz
AQ 3.9976959 sec
RG 101
DW 61.000 usec
DE 13.89 usec
TE 295.8 K
D1 1.00000000 sec
TD0 1
SFO1 400.1824711 MHz
NUC1 1H
P0 2.67 usec
P1 8.00 usec
PLW1 21.26700020 W

F2 - Processing parameters
SI 65536
SF 400.1800094 MHz
WDW EM
SSB 0
LB 0.30 Hz
GB 0
PC 1.00

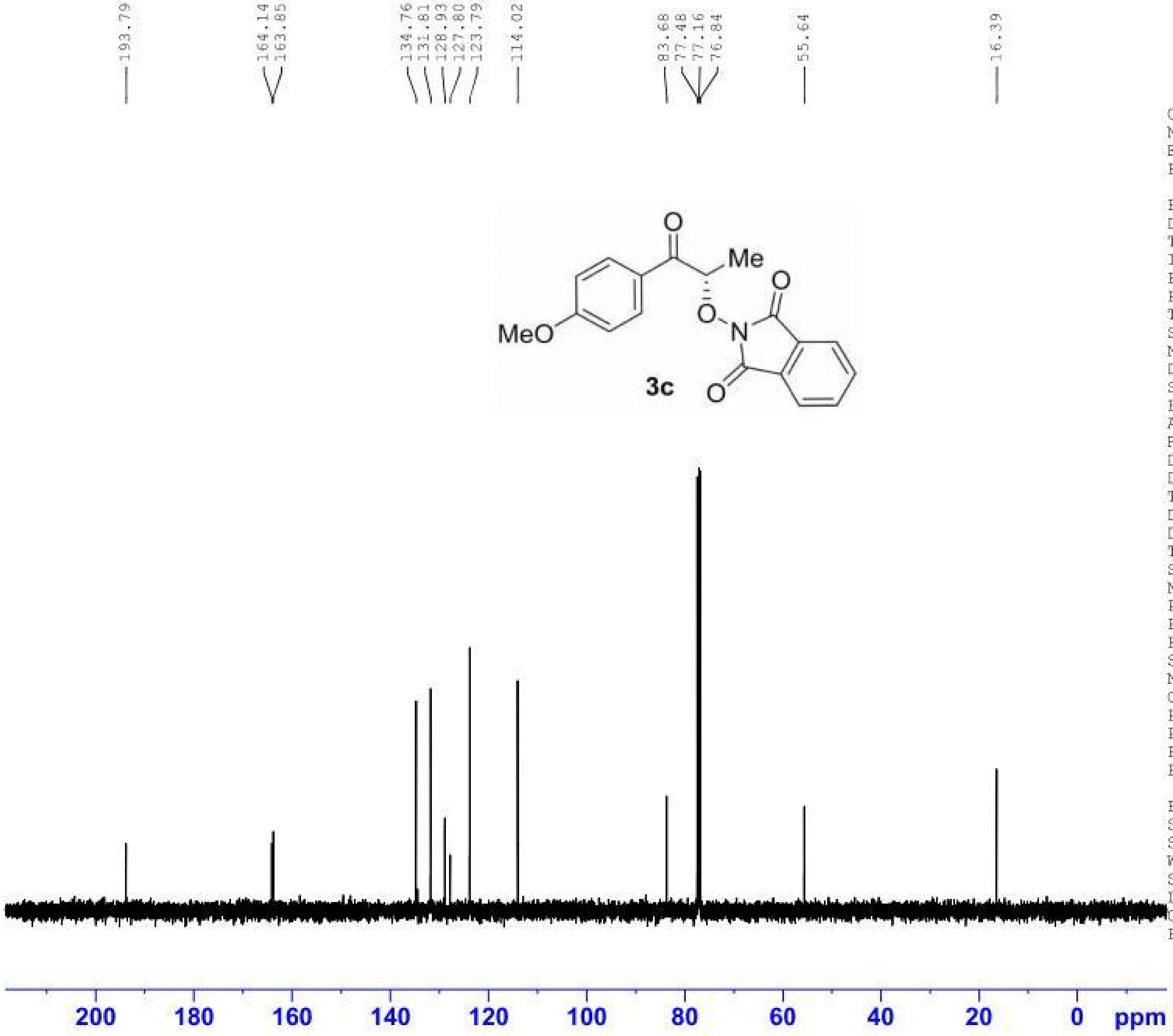

Current Data Parameters
NAME qcx-6-48c
EXPNO 2
PROCNO 1

F2 - Acquisition Parameters
Date_ 20230308
Time 19.30 h
INSTRUM AvanceNeo 400MHz
PROBHD Z163739_0629 (
PULPROG zgpg30
TD 65536
SOLVENT CDCl3
NS 25
DS 4
SWH 23809.523 Hz
FIDRES 0.726609 Hz
AQ 1.3762560 sec
RG 10
DW 21.000 usec
DE 6.50 usec
TE 296.0 K
D1 2.00000000 sec
D11 0.03000000 sec
TD0 1
SFO1 100.6354036 MHz
NUC1 13C
P0 2.67 usec
P1 8.00 usec
PLW1 85.25399780 W
SFO2 400.1816007 MHz
NUC2 1H
CPDPRG[2 waltz65
PCPD2 90.00 usec
PLW2 21.26700020 W
PLW12 0.16802999 W
PLW13 0.08452000 W

F2 - Processing parameters
SI 32768
SF 100.6253306 MHz
WDW EM
SSB 0
LB 1.00 Hz
GB 0
PC 1.40

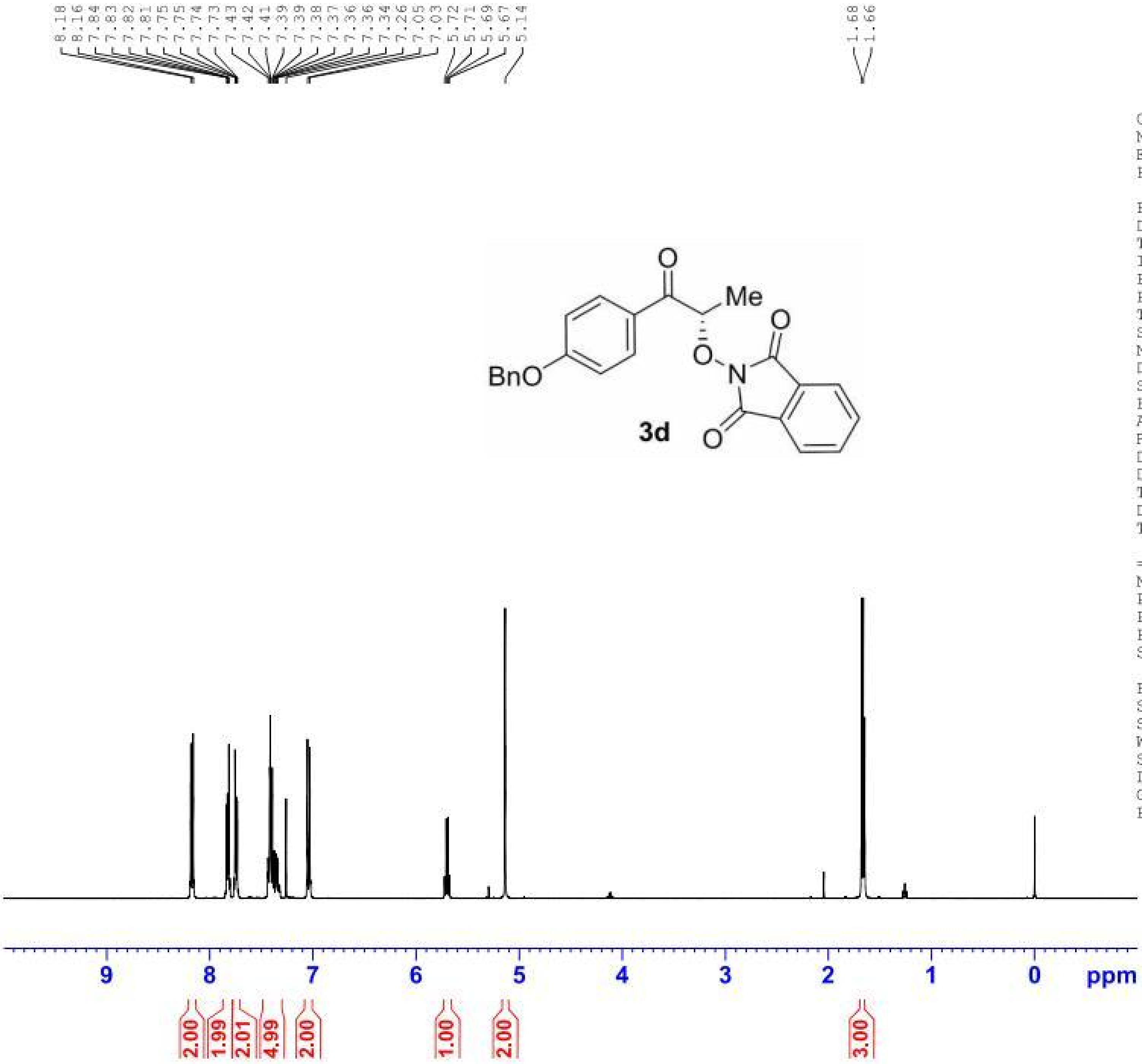

3d

```
Current Data Parameters
NAME          qcx-6-77a
EXPNO                 1
PROCNO                1

F2 - Acquisition Parameters
Date_          20230327
Time              16.13
INSTRUM           spect
PROBHD   5 mm DUL 13C-1
PULPROG            zg30
TD                65536
SOLVENT           CDCl3
NS                    5
DS                    2
SWH            8223.685 Hz
FIDRES         0.125483 Hz
AQ            3.9845889 sec
RG                  362
DW               60.800 usec
DE                 6.00 usec
TE                292.8 K
D1           1.00000000 sec
TD0                   1

======== CHANNEL f1 ========
NUC1                 1H
P1                15.80 usec
PL1               -1.00 dB
PL1W        12.17476940 W
SFO1        400.1324710 MHz

F2 - Processing parameters
SI                32768
SF          400.1300098 MHz
WDW                  EM
SSB                   0
LB                 0.30 Hz
GB                    0
PC                 1.00
```

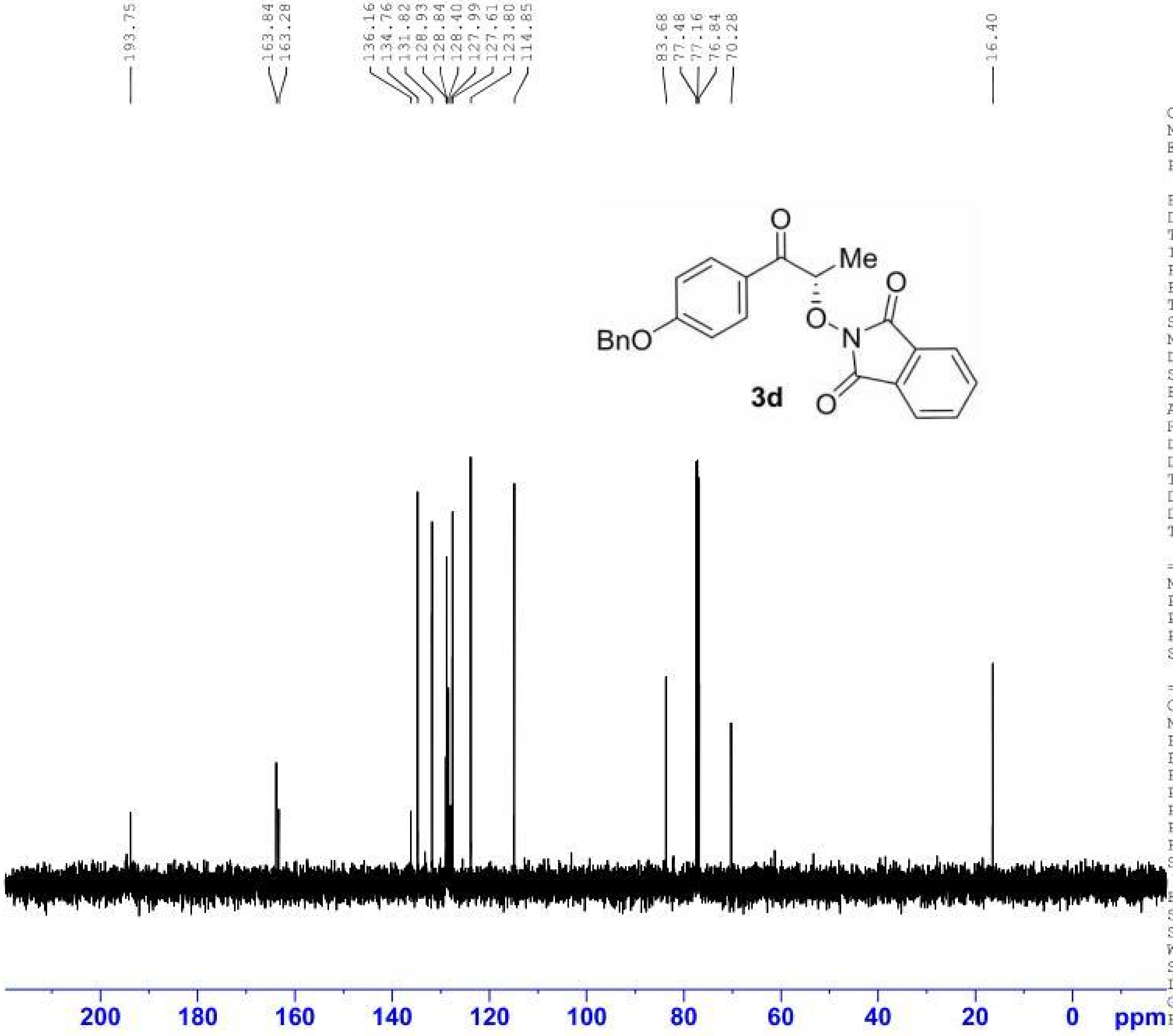

Current Data Parameters
NAME qcx-6-77a
EXPNO 3
PROCNO 1

F2 - Acquisition Parameters
Date_ 20230327
Time 16.19
INSTRUM spect
PROBHD 5 mm DUL 13C-1
PULPROG zgpg30
TD 65536
SOLVENT CDCl3
NS 35
DS 1
SWH 24038.461 Hz
FIDRES 0.366798 Hz
AQ 1.3631488 sec
RG 2050
DW 20.800 usec
DE 6.00 usec
TE 292.9 K
D1 2.00000000 sec
D11 0.03000000 sec
TD0 1

======== CHANNEL f1 ========
NUC1 13C
P1 40.00 usec
PL1 -3.00 dB
PL1W 60.64365387 W
SFO1 100.6228298 MHz

======== CHANNEL f2 ========
CPDPRG[2 waltz16
NUC2 1H
PCPD2 80.00 usec
PL2 -1.00 dB
PL12 14.39 dB
PL13 18.00 dB
PL2W 12.17476940 W
PL12W 0.35193357 W
PL13W 0.15327126 W
SFO2 400.1316005 MHz

F2 - Processing parameters
SI 32768
SF 100.6127585 MHz
WDW EM
SSB 0
LB 1.00 Hz
GB 0
PC 1.40

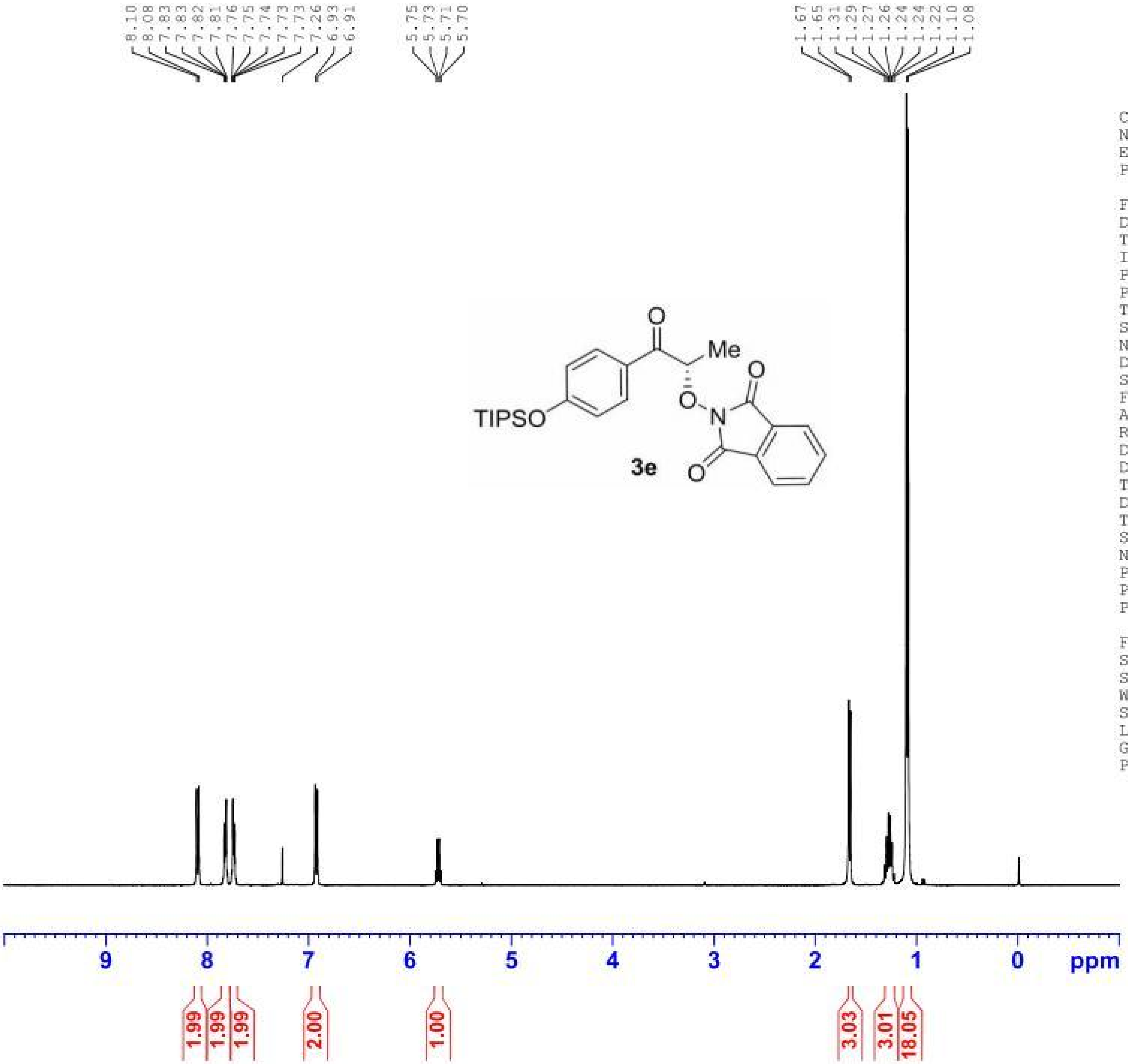


```
Current Data Parameters
NAME          qcx-6-56d-new
EXPNO                     1
PROCNO                    1

F2 - Acquisition Parameters
Date_              20230316
Time                  19.45 h
INSTRUM    AvanceNeo 400MHz
PROBHD     Z163739_0629 (
PULPROG                zg30
TD                    65536
SOLVENT               CDCl3
NS                        2
DS                        2
SWH                8196.722 Hz
FIDRES             0.250144 Hz
AQ                3.9976959 sec
RG                      101
DW                   61.000 usec
DE                    13.89 usec
TE                    296.1 K
D1               1.00000000 sec
TD0                       1
SFO1            400.1824711 MHz
NUC1                     1H
P0                     2.67 usec
P1                     8.00 usec
PLW1            21.26700020 W

F2 - Processing parameters
SI                    65536
SF              400.1800100 MHz
WDW                      EM
SSB                       0
LB                     0.30 Hz
GB                        0
PC                     1.00
```

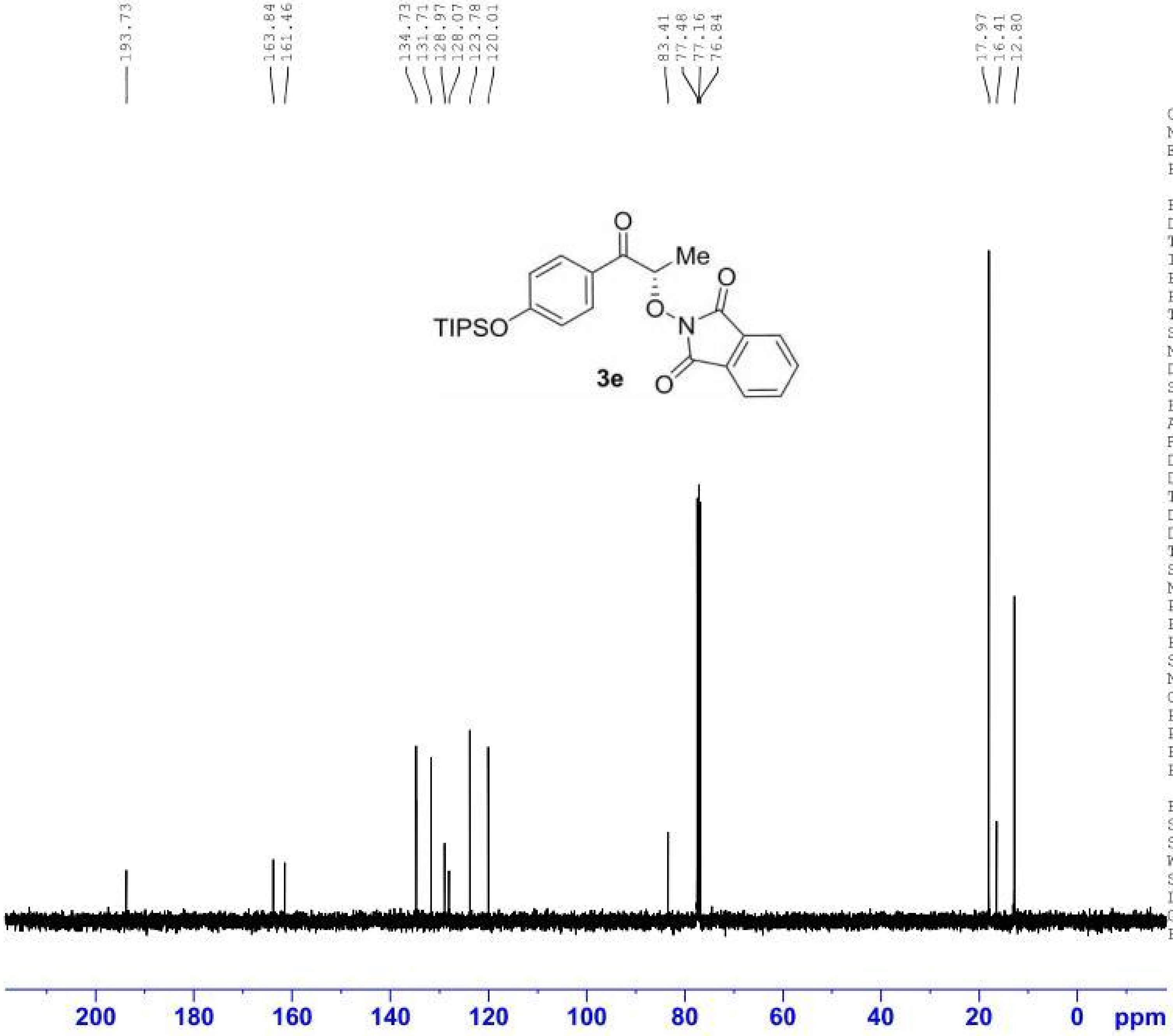

Current Data Parameters
NAME qcx-6-56d-new
EXPNO 2
PROCNO 1

F2 - Acquisition Parameters
Date_ 20230316
Time 19.48 h
INSTRUM AvanceNeo 400MHz
PROBHD Z163739_0629 (
PULPROG zgpg30
TD 65536
SOLVENT CDCl3
NS 24
DS 4
SWH 23809.523 Hz
FIDRES 0.726609 Hz
AQ 1.3762560 sec
RG 10
DW 21.000 usec
DE 6.50 usec
TE 296.3 K
D1 2.00000000 sec
D11 0.03000000 sec
TD0 1
SFO1 100.6354036 MHz
NUC1 13C
P0 2.67 usec
P1 8.00 usec
PLW1 85.25399780 W
SFO2 400.1816007 MHz
NUC2 1H
CPDPRG[2 waltz65
PCPD2 90.00 usec
PLW2 21.26700020 W
PLW12 0.16802999 W
PLW13 0.08452000 W

F2 - Processing parameters
SI 32768
SF 100.6253299 MHz
WDW EM
SSB 0
LB 1.00 Hz
GB 0
PC 1.40

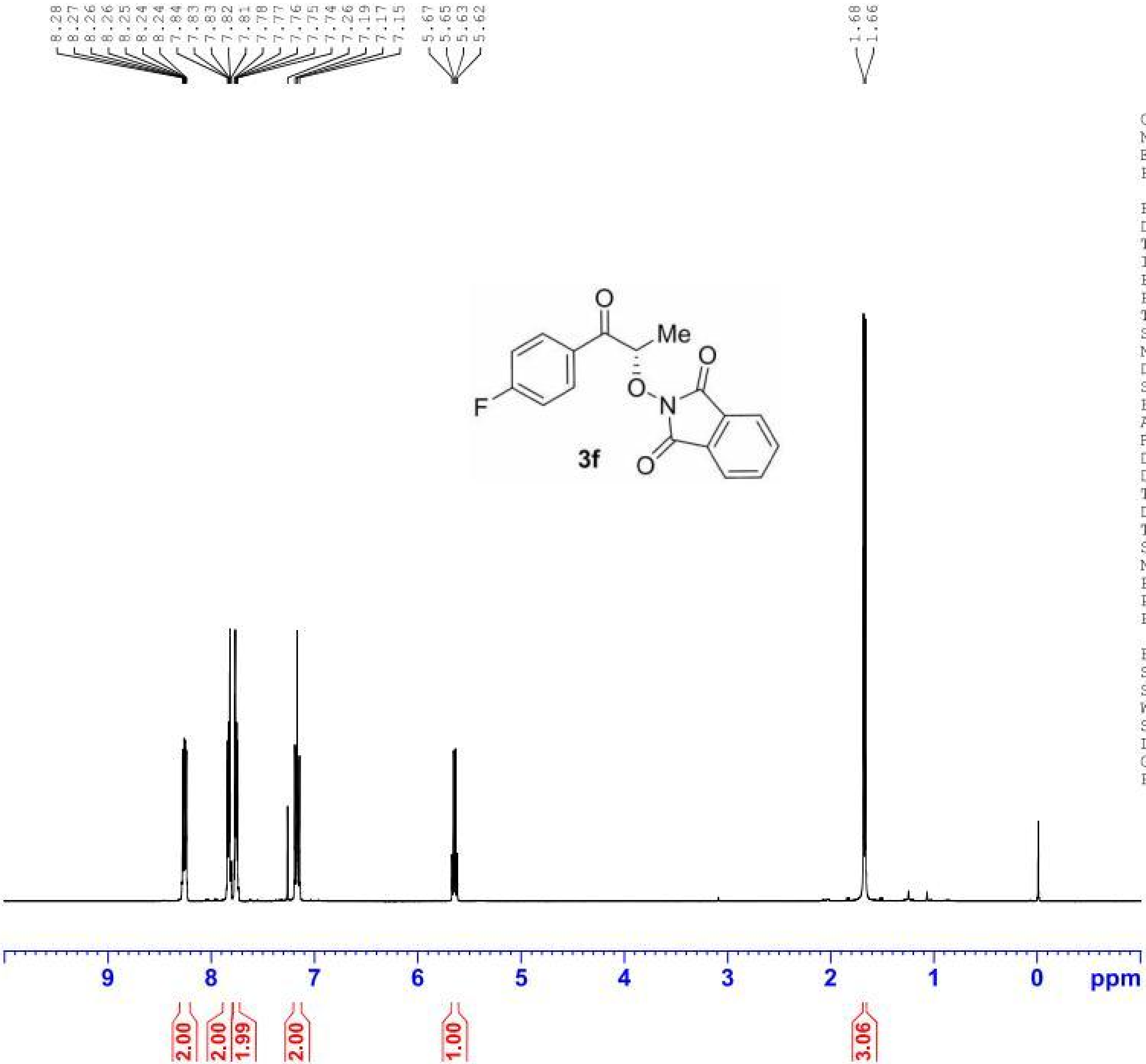

3f

```
Current Data Parameters
NAME          qcx-6-41a
EXPNO                 1
PROCNO                1

F2 - Acquisition Parameters
Date_          20230304
Time              19.48 h
INSTRUM  AvanceNeo 400MHz
PROBHD   Z163739_0629 (
PULPROG            zg30
TD                65536
SOLVENT           CDCl3
NS                    5
DS                    2
SWH            8196.722 Hz
FIDRES         0.250144 Hz
AQ            3.9976959 sec
RG                  101
DW               61.000 usec
DE                13.89 usec
TE                294.8 K
D1           1.00000000 sec
TD0                   1
SFO1        400.1824711 MHz
NUC1                 1H
P0                 2.67 usec
P1                 8.00 usec
PLW1        21.26700020 W

F2 - Processing parameters
SI                65536
SF          400.1800093 MHz
WDW                  EM
SSB                   0
LB                 0.30 Hz
GB                    0
PC                 1.00
```

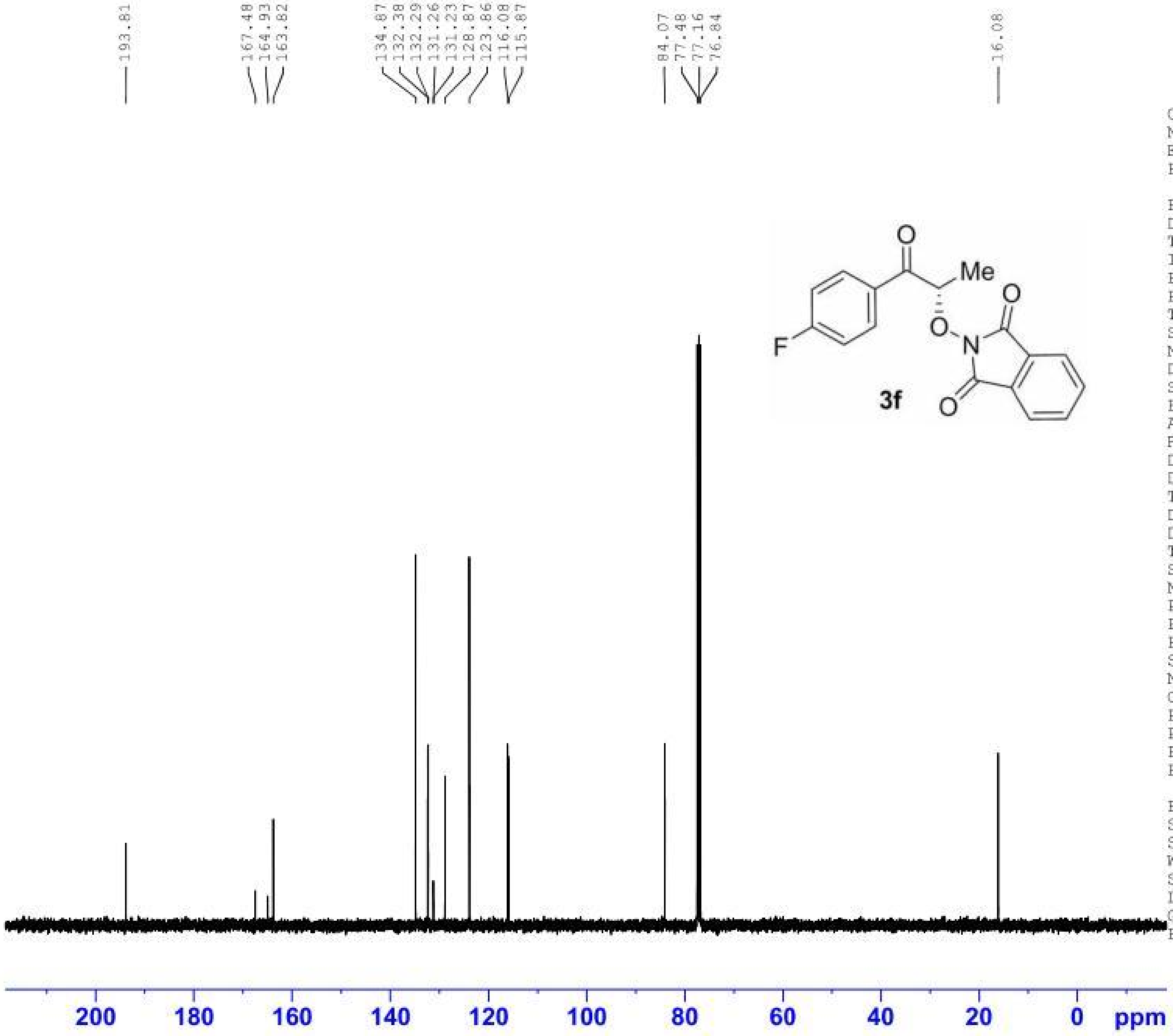

3f

Current Data Parameters
NAME qcx-6-41a
EXPNO 2
PROCNO 1

F2 - Acquisition Parameters
Date_ 20230304
Time 19.55 h
INSTRUM AvanceNeo 400MHz
PROBHD Z163739_0629 (
PULPROG zgpg30
TD 65536
SOLVENT CDCl3
NS 91
DS 4
SWH 23809.523 Hz
FIDRES 0.726609 Hz
AQ 1.3762560 sec
RG 10
DW 21.000 usec
DE 6.50 usec
TE 295.5 K
D1 2.00000000 sec
D11 0.03000000 sec
TD0 1
SFO1 100.6354036 MHz
NUC1 13C
P0 2.67 usec
P1 8.00 usec
PLW1 85.25399780 W
SFO2 400.1816007 MHz
NUC2 1H
CPDPRG[2 waltz65
PCPD2 90.00 usec
PLW2 21.26700020 W
PLW12 0.16802999 W
PLW13 0.08452000 W

F2 - Processing parameters
SI 32768
SF 100.6253302 MHz
WDW EM
SSB 0
LB 1.00 Hz
GB 0
PC 1.40

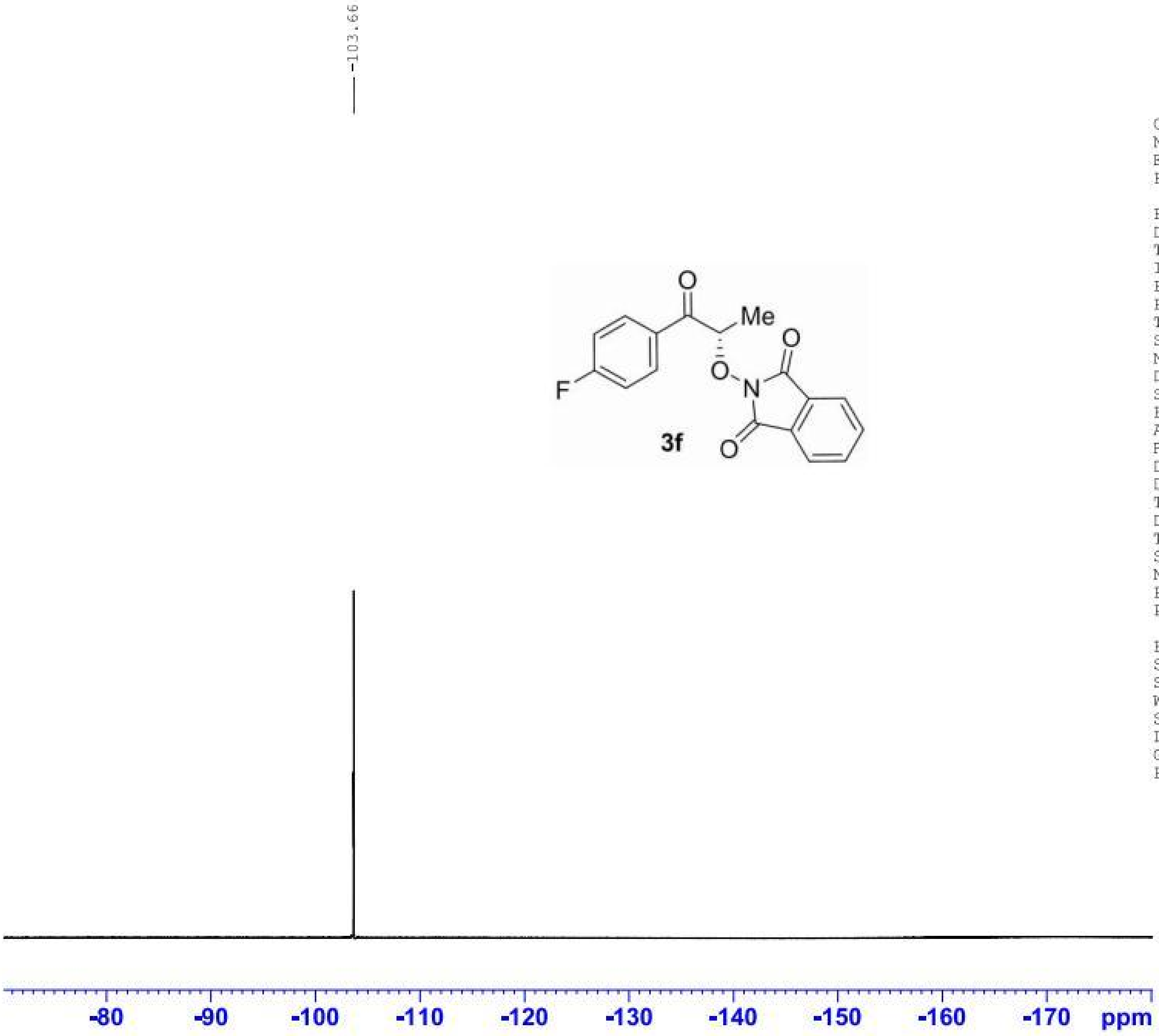

3f

Current Data Parameters
NAME qcx-6-41a
EXPNO 3
PROCNO 1

F2 - Acquisition Parameters
Date_ 20230304
Time 19.57 h
INSTRUM AvanceNeo 400MHz
PROBHD Z163739_0629 (
PULPROG zg
TD 131072
SOLVENT CDCl3
NS 16
DS 4
SWH 90909.094 Hz
FIDRES 1.387163 Hz
AQ 0.7208960 sec
RG 101
DW 5.500 usec
DE 6.50 usec
TE 295.1 K
D1 1.00000000 sec
TD0 1
SFO1 376.5077587 MHz
NUC1 19F
P1 12.00 usec
PLW1 33.72800064 W

F2 - Processing parameters
SI 65536
SF 376.5454132 MHz
WDW EM
SSB 0
LB 0.30 Hz
GB 0
PC 1.00

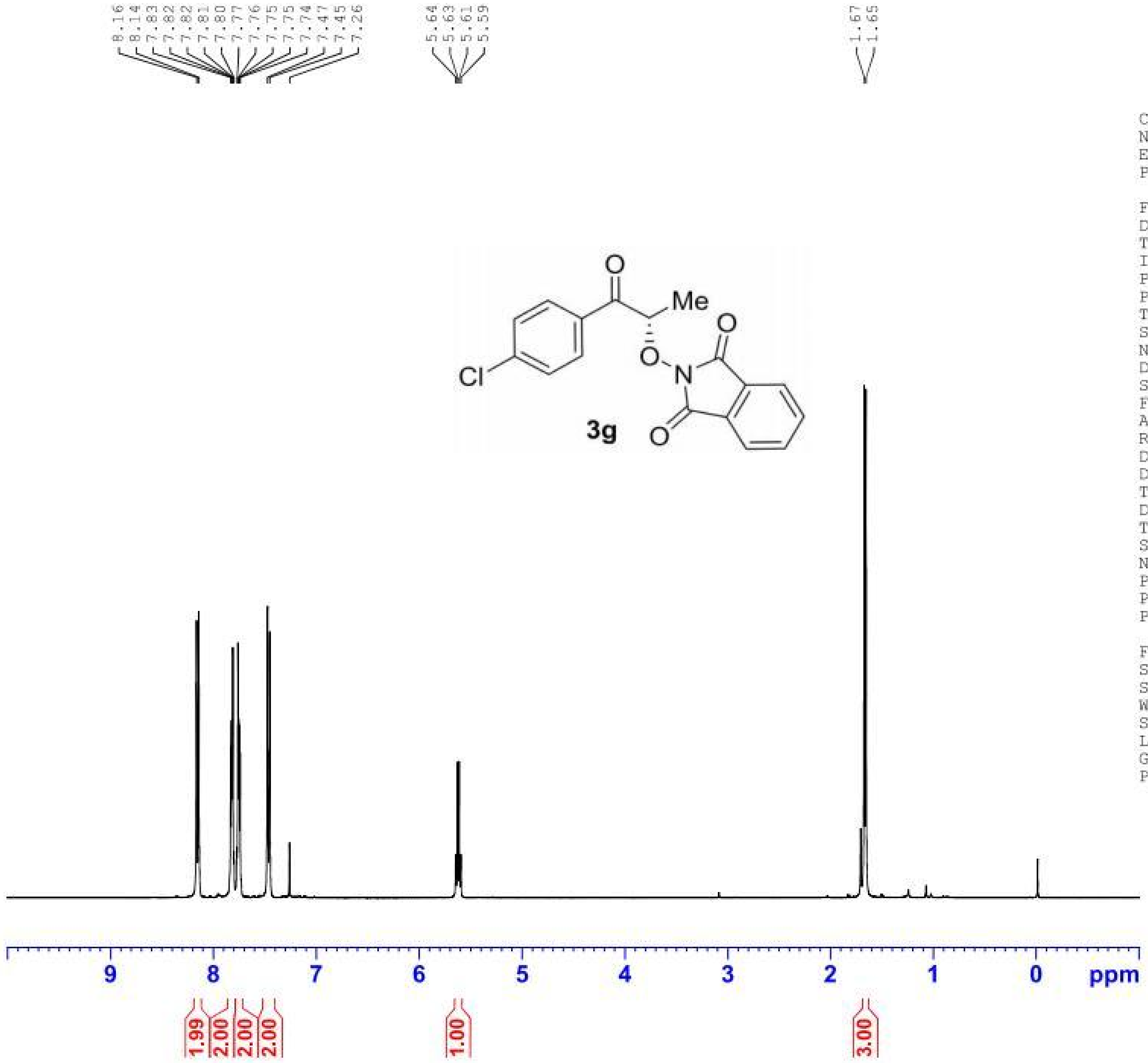


```
Current Data Parameters
NAME          qcx-6-41b
EXPNO                 1
PROCNO                1

F2 - Acquisition Parameters
Date_          20230304
Time              19.25 h
INSTRUM   AvanceNeo 400MHz
PROBHD    Z163739_0629 (
PULPROG            zg30
TD                65536
SOLVENT           CDCl3
NS                    3
DS                    2
SWH            8196.722 Hz
FIDRES         0.250144 Hz
AQ            3.9976959 sec
RG                  101
DW               61.000 usec
DE                13.89 usec
TE                294.8 K
D1           1.00000000 sec
TD0                   1
SFO1        400.1824711 MHz
NUC1                 1H
P0                 2.67 usec
P1                 8.00 usec
PLW1        21.26700020 W

F2 - Processing parameters
SI                65536
SF          400.1800091 MHz
WDW                  EM
SSB                   0
LB                 0.30 Hz
GB                    0
PC                 1.00
```

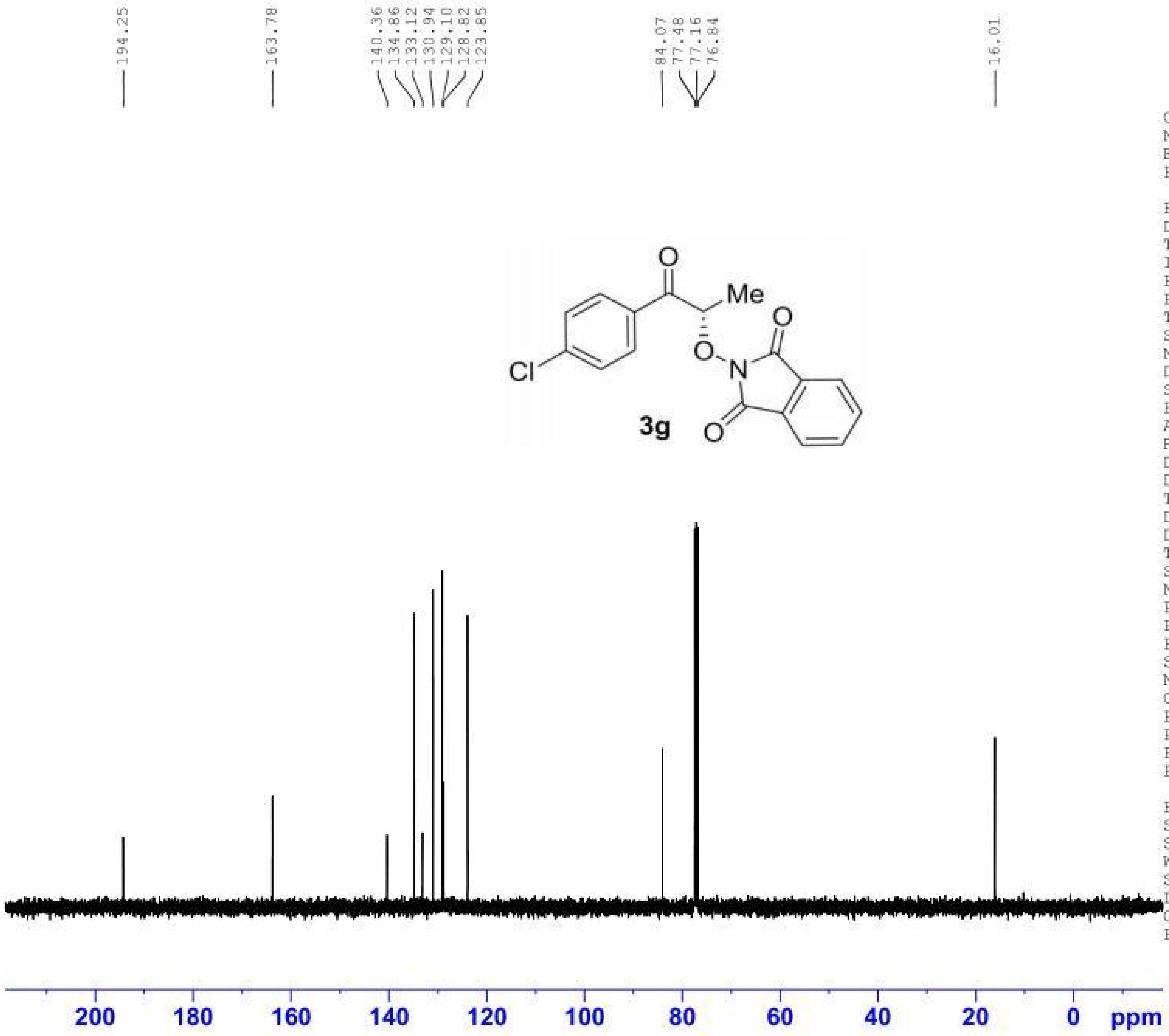

Current Data Parameters
NAME qcx-6-41b
EXPNO 2
PROCNO 1

F2 - Acquisition Parameters
Date_ 20230304
Time 19.28 h
INSTRUM AvanceNeo 400MHz
PROBHD Z163739_0629 (
PULPROG zgpg30
TD 65536
SOLVENT CDCl3
NS 23
DS 4
SWH 23809.523 Hz
FIDRES 0.726609 Hz
AQ 1.3762560 sec
RG 10
DW 21.000 usec
DE 6.50 usec
TE 295.1 K
D1 2.00000000 sec
D11 0.03000000 sec
TD0 1
SFO1 100.6354036 MHz
NUC1 13C
P0 2.67 usec
P1 8.00 usec
PLW1 85.25399780 W
SFO2 400.1816007 MHz
NUC2 1H
CPDPRG[2 waltz65
PCPD2 90.00 usec
PLW2 21.26700020 W
PLW12 0.16802999 W
PLW13 0.08452000 W

F2 - Processing parameters
SI 32768
SF 100.6253323 MHz
WDW EM
SSB 0
LB 1.00 Hz
GB 0
PC 1.40

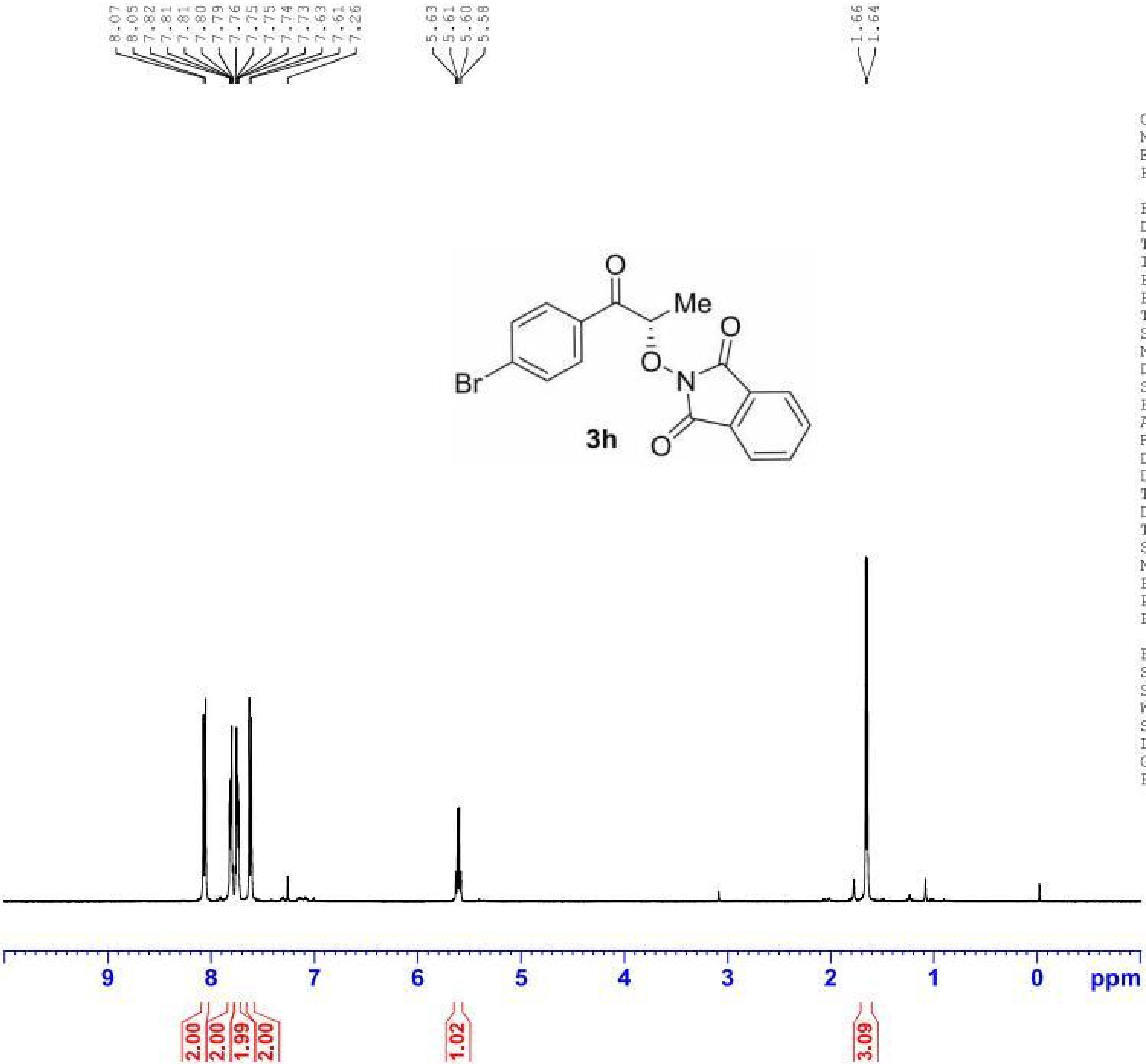

Current Data Parameters
NAME qcx-6-41c
EXPNO 1
PROCNO 1

F2 - Acquisition Parameters
Date_ 20230304
Time 19.34 h
INSTRUM AvanceNeo 400MHz
PROBHD Z163739_0629 (
PULPROG zg30
TD 65536
SOLVENT CDCl3
NS 6
DS 2
SWH 8196.722 Hz
FIDRES 0.250144 Hz
AQ 3.9976959 sec
RG 101
DW 61.000 usec
DE 13.89 usec
TE 294.8 K
D1 1.00000000 sec
TD0 1
SFO1 400.1824711 MHz
NUC1 1H
P0 2.67 usec
P1 8.00 usec
PLW1 21.26700020 W

F2 - Processing parameters
SI 65536
SF 400.1800092 MHz
WDW EM
SSB 0
LB 0.30 Hz
GB 0
PC 1.00

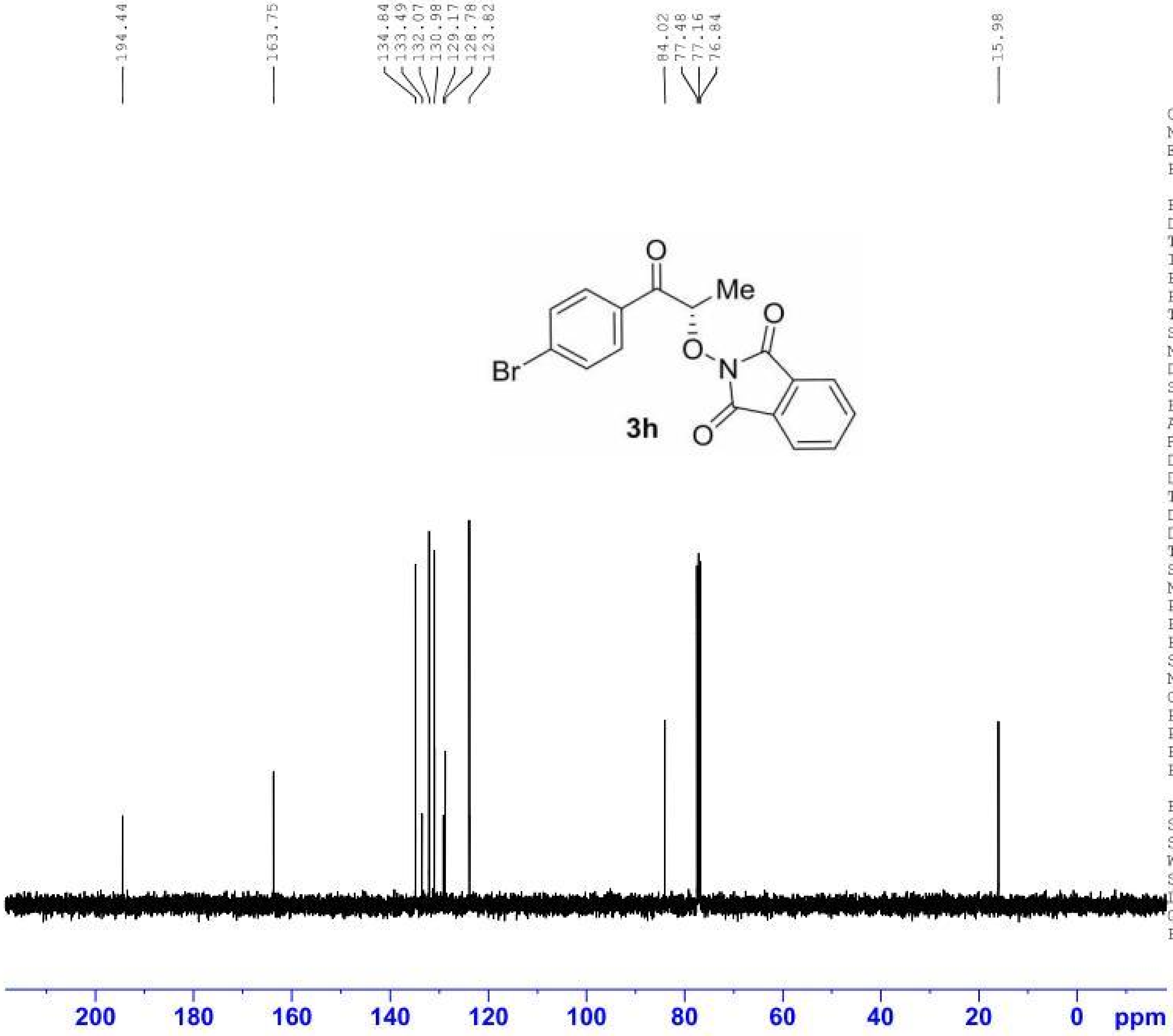

Current Data Parameters
NAME qcx-6-41c
EXPNO 2
PROCNO 1

F2 - Acquisition Parameters
Date_ 20230304
Time 19.36 h
INSTRUM AvanceNeo 400MHz
PROBHD Z163739_0629 (
PULPROG zgpg30
TD 65536
SOLVENT CDCl3
NS 11
DS 4
SWH 23809.523 Hz
FIDRES 0.726609 Hz
AQ 1.3762560 sec
RG 10
DW 21.000 usec
DE 6.50 usec
TE 295.1 K
D1 2.00000000 sec
D11 0.03000000 sec
TD0 1
SFO1 100.6354036 MHz
NUC1 13C
P0 2.67 usec
P1 8.00 usec
PLW1 85.25399780 W
SFO2 400.1816007 MHz
NUC2 1H
CPDPRG[2 waltz65
PCPD2 90.00 usec
PLW2 21.26700020 W
PLW12 0.16802999 W
PLW13 0.08452000 W

F2 - Processing parameters
SI 32768
SF 100.6253348 MHz
WDW EM
SSB 0
LB 1.00 Hz
GB 0
PC 1.40

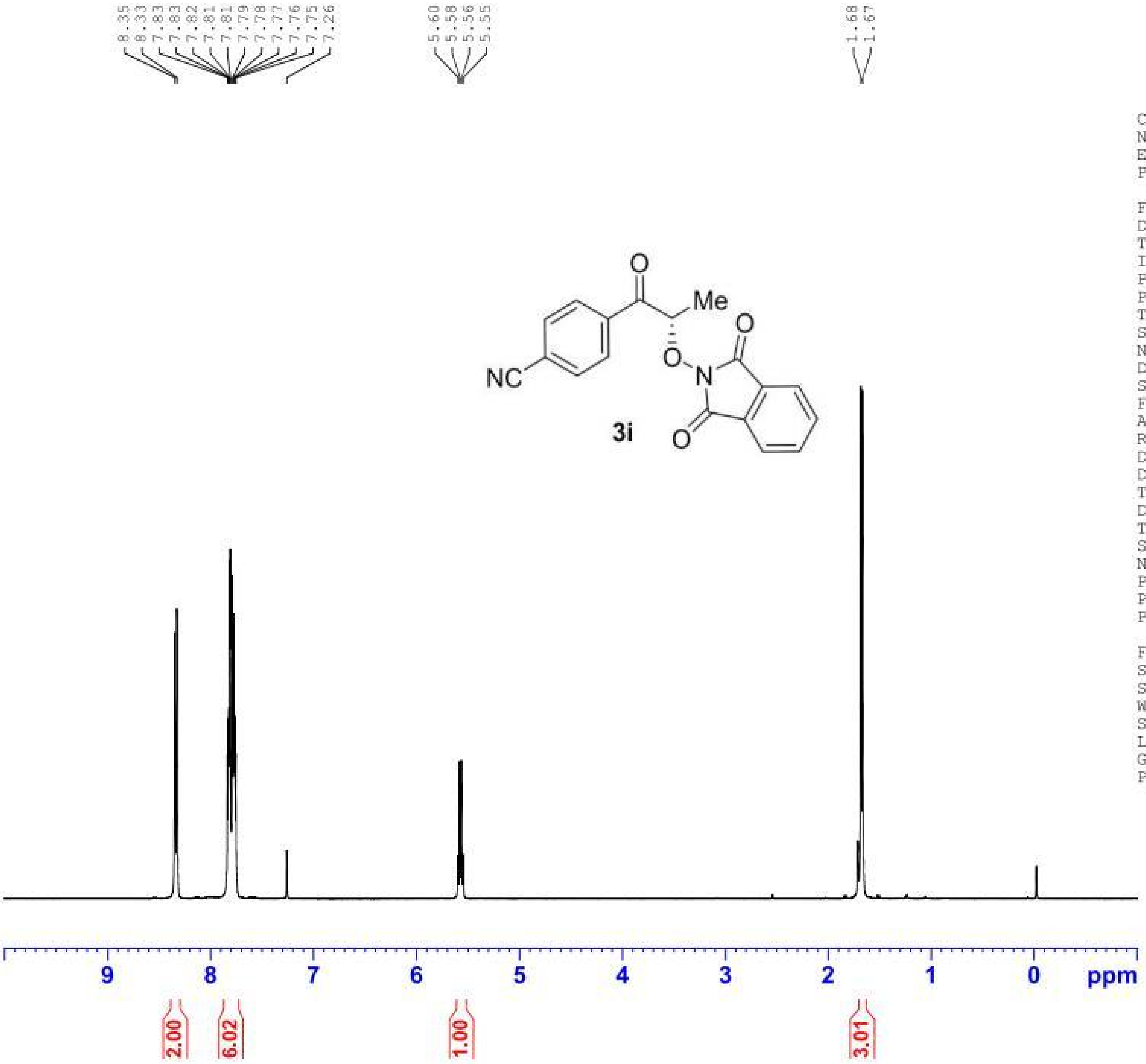


```
Current Data Parameters
NAME            qcx-6-41d
EXPNO                   1
PROCNO                  1

F2 - Acquisition Parameters
Date_            20230304
Time                19.41 h
INSTRUM   AvanceNeo 400MHz
PROBHD    Z163739_0629 (
PULPROG              zg30
TD                  65536
SOLVENT             CDCl3
NS                      5
DS                      2
SWH              8196.722 Hz
FIDRES           0.250144 Hz
AQ              3.9976959 sec
RG                    101
DW                 61.000 usec
DE                  13.89 usec
TE                  294.9 K
D1             1.00000000 sec
TD0                     1
SFO1          400.1824711 MHz
NUC1                   1H
P0                   2.67 usec
P1                   8.00 usec
PLW1          21.26700020 W

F2 - Processing parameters
SI                  65536
SF            400.1800092 MHz
WDW                    EM
SSB                     0
LB                   0.30 Hz
GB                      0
PC                   1.00
```

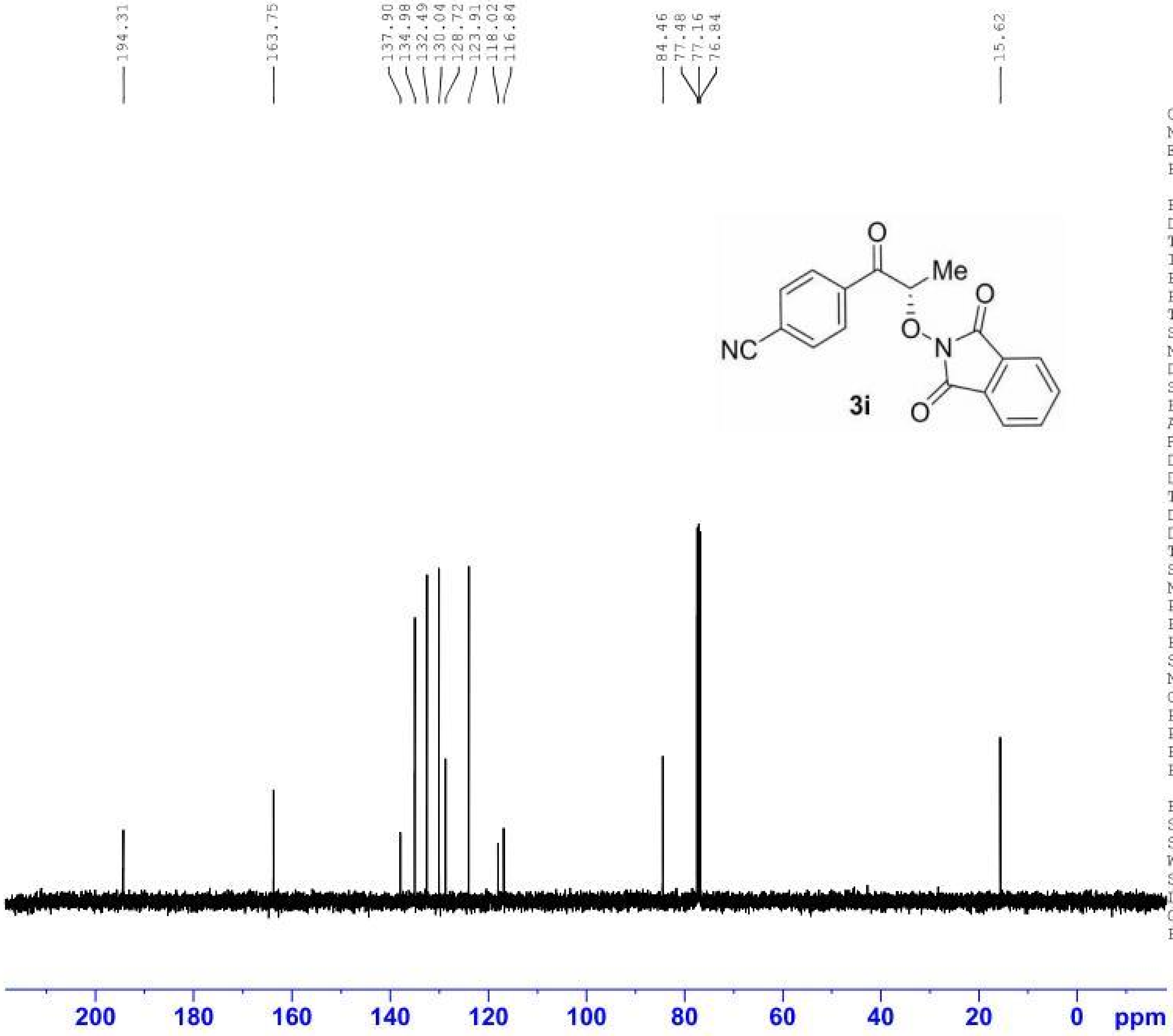

Current Data Parameters
NAME qcx-6-41d
EXPNO 2
PROCNO 1

F2 - Acquisition Parameters
Date_ 20230304
Time 19.43 h
INSTRUM AvanceNeo 400MHz
PROBHD Z163739_0629 (
PULPROG zgpg30
TD 65536
SOLVENT CDCl3
NS 21
DS 4
SWH 23809.523 Hz
FIDRES 0.726609 Hz
AQ 1.3762560 sec
RG 10
DW 21.000 usec
DE 6.50 usec
TE 295.2 K
D1 2.00000000 sec
D11 0.03000000 sec
TD0 1
SFO1 100.6354036 MHz
NUC1 13C
P0 2.67 usec
P1 8.00 usec
PLW1 85.25399780 W
SFO2 400.1816007 MHz
NUC2 1H
CPDPRG[2 waltz65
PCPD2 90.00 usec
PLW2 21.26700020 W
PLW12 0.16802999 W
PLW13 0.08452000 W

F2 - Processing parameters
SI 32768
SF 100.6253342 MHz
WDW EM
SSB 0
LB 1.00 Hz
GB 0
PC 1.40

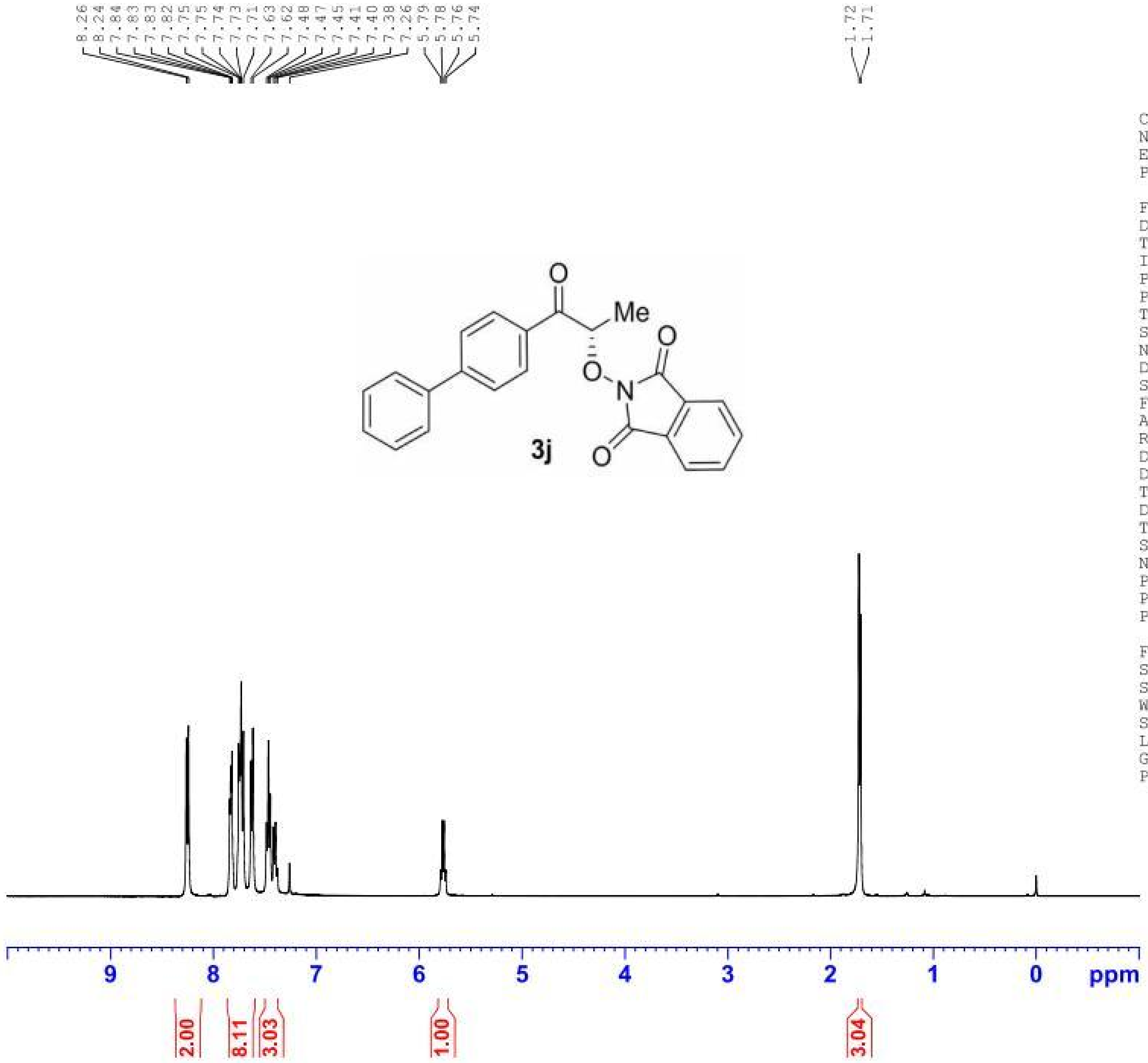


```
Current Data Parameters
NAME          qcx-6-56b
EXPNO                 1
PROCNO                1

F2 - Acquisition Parameters
Date_          20230315
Time              19.30 h
INSTRUM  AvanceNeo 400MHz
PROBHD   Z163739_0629 (
PULPROG            zg30
TD                65536
SOLVENT           CDCl3
NS                    4
DS                    2
SWH            8196.722 Hz
FIDRES         0.250144 Hz
AQ            3.9976959 sec
RG                  101
DW               61.000 usec
DE                13.89 usec
TE                296.0 K
D1           1.00000000 sec
TD0                   1
SFO1        400.1824711 MHz
NUC1                 1H
P0                 2.67 usec
P1                 8.00 usec
PLW1        21.26700020 W

F2 - Processing parameters
SI                65536
SF          400.1800090 MHz
WDW                  EM
SSB                   0
LB                 0.30 Hz
GB                    0
PC                 1.00
```

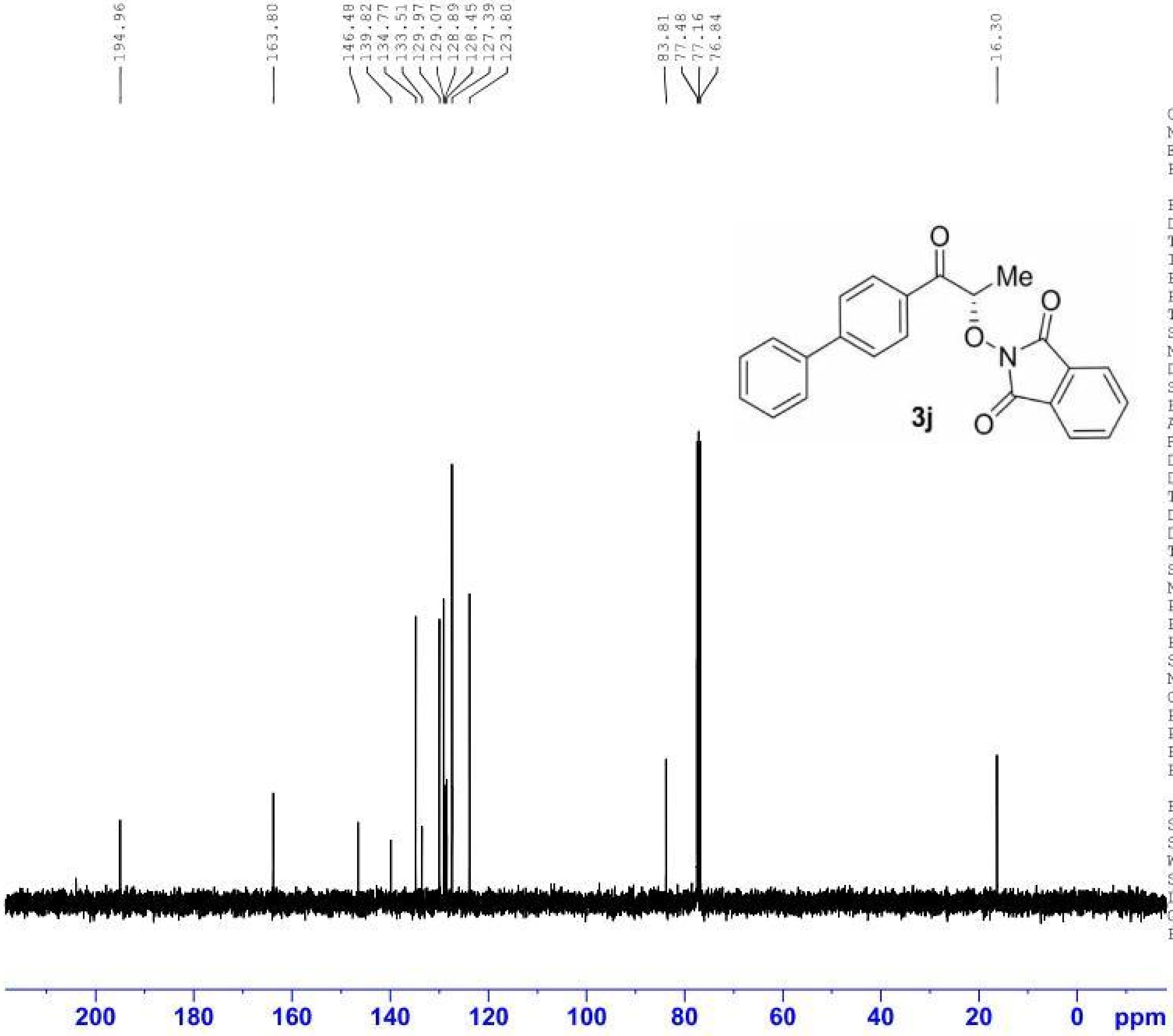

Current Data Parameters
NAME qcx-6-56b
EXPNO 2
PROCNO 1

F2 - Acquisition Parameters
Date_ 20230315
Time 19.32 h
INSTRUM AvanceNeo 400MHz
PROBHD Z163739_0629 (
PULPROG zgpg30
TD 65536
SOLVENT CDCl3
NS 16
DS 4
SWH 23809.523 Hz
FIDRES 0.726609 Hz
AQ 1.3762560 sec
RG 10
DW 21.000 usec
DE 6.50 usec
TE 296.2 K
D1 2.00000000 sec
D11 0.03000000 sec
TD0 1
SFO1 100.6354036 MHz
NUC1 13C
P0 2.67 usec
P1 8.00 usec
PLW1 85.25399780 W
SFO2 400.1816007 MHz
NUC2 1H
CPDPRG[2 waltz65
PCPD2 90.00 usec
PLW2 21.26700020 W
PLW12 0.16802999 W
PLW13 0.08452000 W

F2 - Processing parameters
SI 32768
SF 100.6253334 MHz
WDW EM
SSB 0
LB 1.00 Hz
GB 0
PC 1.40

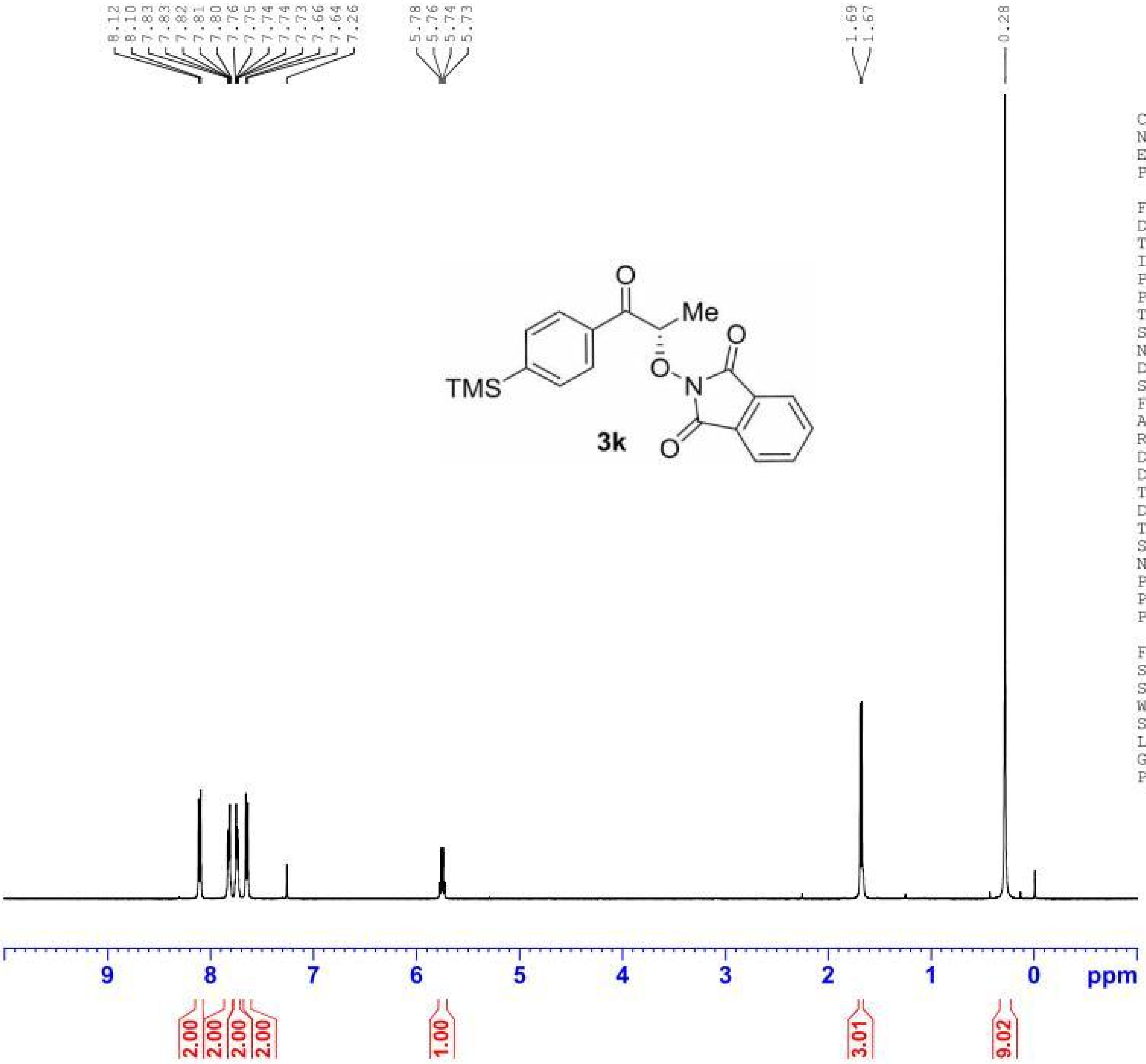


```
Current Data Parameters
NAME        qcx-6-56c-new
EXPNO                   1
PROCNO                  1

F2 - Acquisition Parameters
Date_            20230316
Time               19.39 h
INSTRUM   AvanceNeo 400MHz
PROBHD    Z163739_0629 (
PULPROG              zg30
TD                  65536
SOLVENT             CDCl3
NS                      5
DS                      2
SWH              8196.722 Hz
FIDRES           0.250144 Hz
AQ              3.9976959 sec
RG                    101
DW                 61.000 usec
DE                  13.89 usec
TE                  296.1 K
D1             1.00000000 sec
TD0                     1
SFO1          400.1824711 MHz
NUC1                   1H
P0                   2.67 usec
P1                   8.00 usec
PLW1          21.26700020 W

F2 - Processing parameters
SI                  65536
SF            400.1800097 MHz
WDW                    EM
SSB                     0
LB                   0.30 Hz
GB                      0
PC                   1.00
```

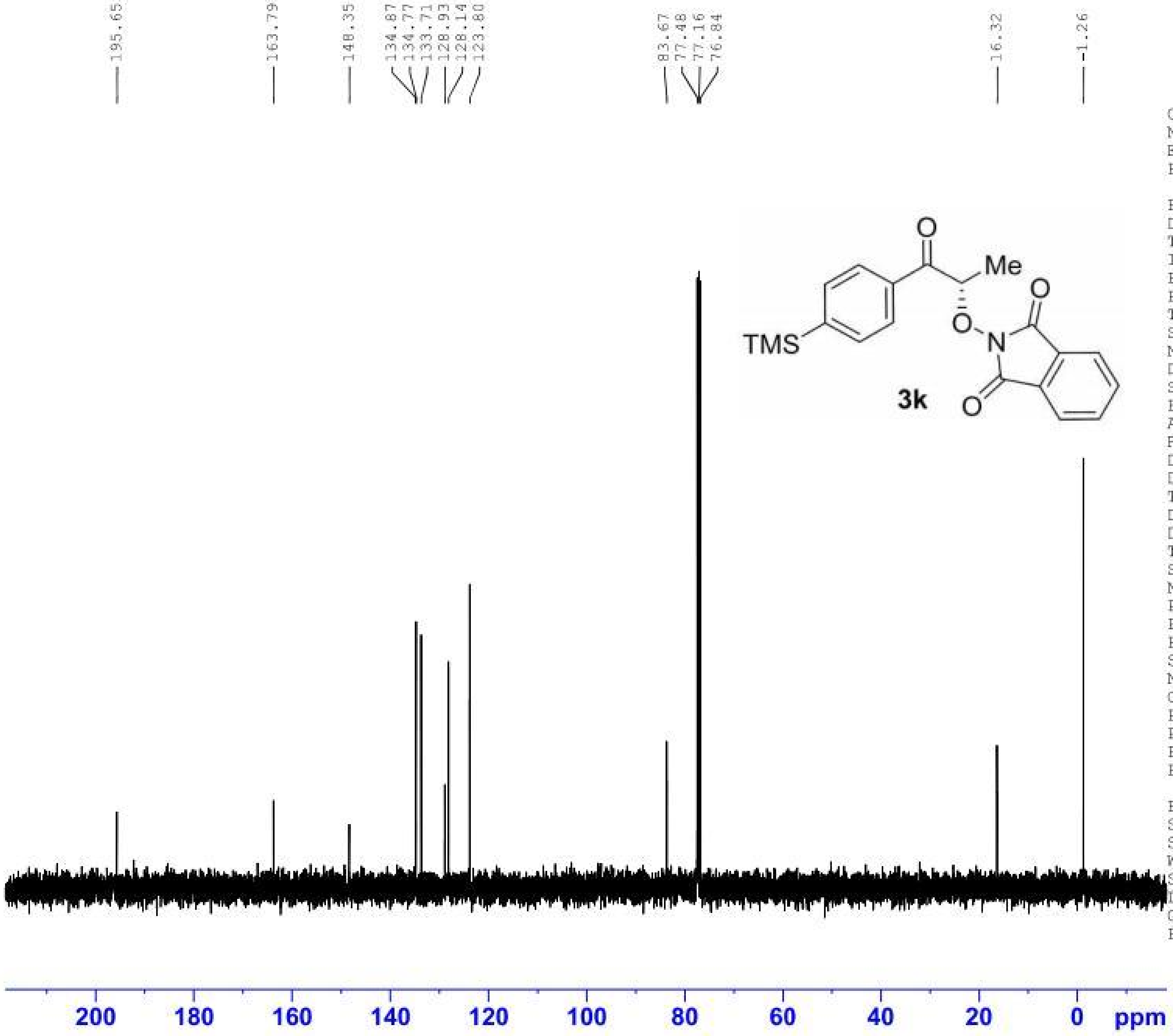

Current Data Parameters
NAME qcx-6-56c-new
EXPNO 2
PROCNO 1

F2 - Acquisition Parameters
Date_ 20230316
Time 19.41 h
INSTRUM AvanceNeo 400MHz
PROBHD Z163739_0629 (
PULPROG zgpg30
TD 65536
SOLVENT CDCl3
NS 13
DS 4
SWH 23809.523 Hz
FIDRES 0.726609 Hz
AQ 1.3762560 sec
RG 10
DW 21.000 usec
DE 6.50 usec
TE 296.4 K
D1 2.00000000 sec
D11 0.03000000 sec
TD0 1
SFO1 100.6354036 MHz
NUC1 13C
P0 2.67 usec
P1 8.00 usec
PLW1 85.25399780 W
SFO2 400.1816007 MHz
NUC2 1H
CPDPRG[2 waltz65
PCPD2 90.00 usec
PLW2 21.26700020 W
PLW12 0.16802999 W
PLW13 0.08452000 W

F2 - Processing parameters
SI 32768
SF 100.6253304 MHz
WDW EM
SSB 0
LB 1.00 Hz
GB 0
PC 1.40

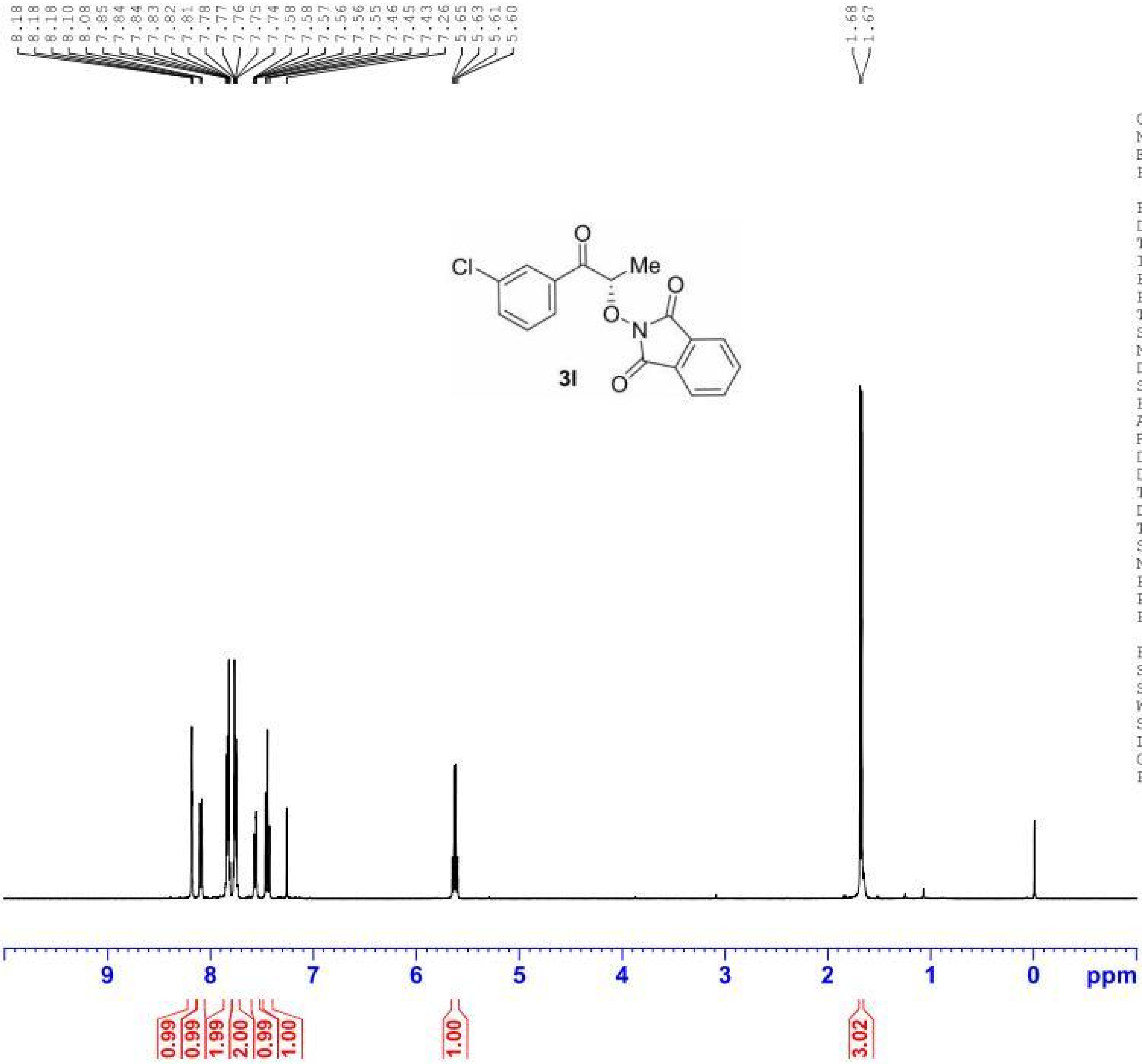

3l

Current Data Parameters
NAME qcx-6-50c
EXPNO 2
PROCNO 1

F2 - Acquisition Parameters
Date_ 20230310
Time 19.41 h
INSTRUM AvanceNeo 400MHz
PROBHD Z163739_0629 (
PULPROG zg30
TD 65536
SOLVENT CDCl3
NS 3
DS 2
SWH 8196.722 Hz
FIDRES 0.250144 Hz
AQ 3.9976959 sec
RG 101
DW 61.000 usec
DE 13.89 usec
TE 296.4 K
D1 1.00000000 sec
TD0 1
SFO1 400.1824711 MHz
NUC1 1H
P0 2.67 usec
P1 8.00 usec
PLW1 21.26700020 W

F2 - Processing parameters
SI 65536
SF 400.1800097 MHz
WDW EM
SSB 0
LB 0.30 Hz
GB 0
PC 1.00

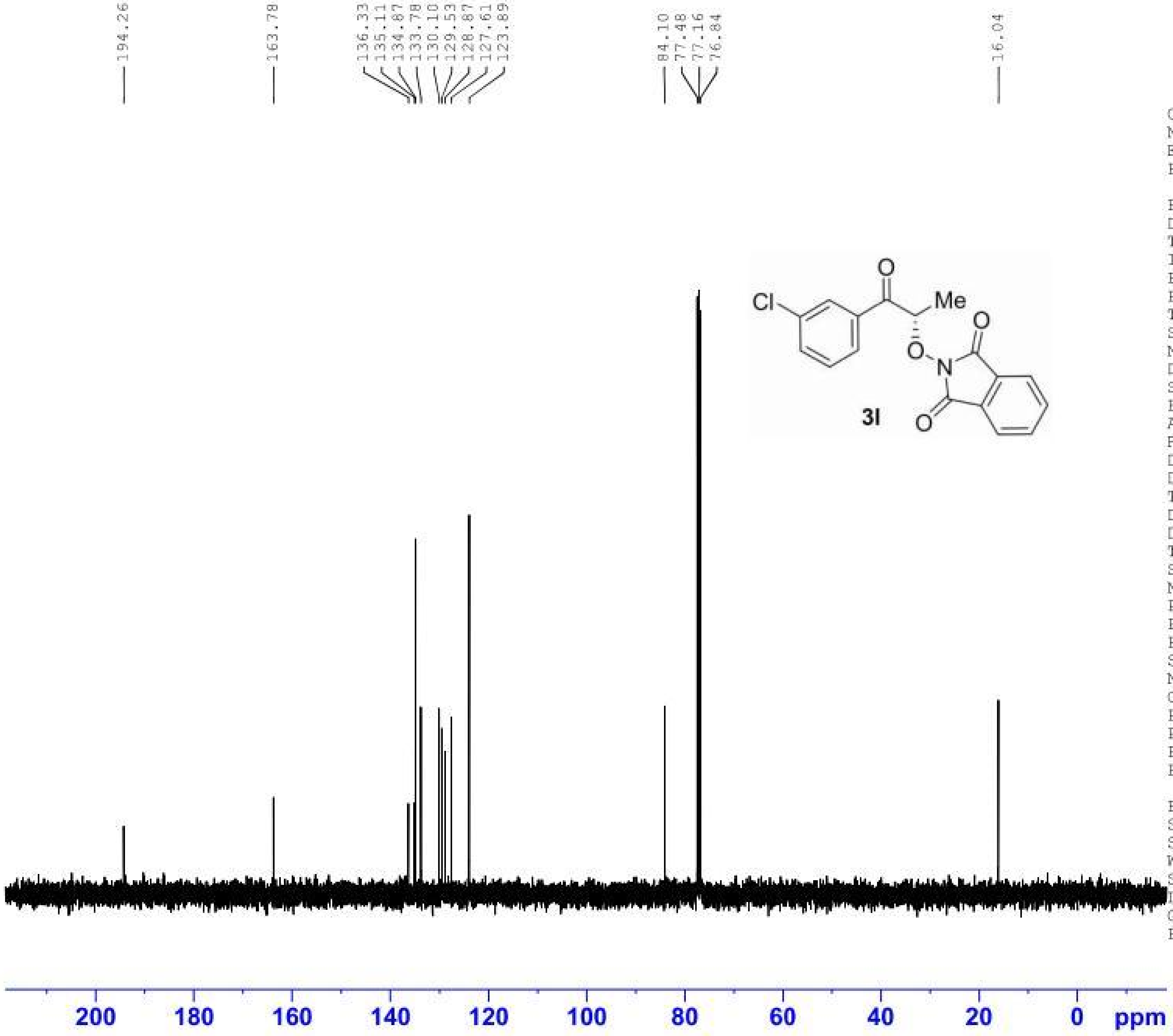

3l

Current Data Parameters
NAME qcx-6-50c
EXPNO 3
PROCNO 1

F2 - Acquisition Parameters
Date_ 20230310
Time 19.44 h
INSTRUM AvanceNeo 400MHz
PROBHD Z163739_0629 (
PULPROG zgpg30
TD 65536
SOLVENT CDCl3
NS 21
DS 4
SWH 23809.523 Hz
FIDRES 0.726609 Hz
AQ 1.3762560 sec
RG 11.3
DW 21.000 usec
DE 6.50 usec
TE 296.7 K
D1 2.00000000 sec
D11 0.03000000 sec
TD0 1
SFO1 100.6354036 MHz
NUC1 13C
P0 2.67 usec
P1 8.00 usec
PLW1 85.25399780 W
SFO2 400.1816007 MHz
NUC2 1H
CPDPRG[2 waltz65
PCPD2 90.00 usec
PLW2 21.26700020 W
PLW12 0.16802999 W
PLW13 0.08452000 W

F2 - Processing parameters
SI 32768
SF 100.6253299 MHz
WDW EM
SSB 0
LB 1.00 Hz
GB 0
PC 1.40

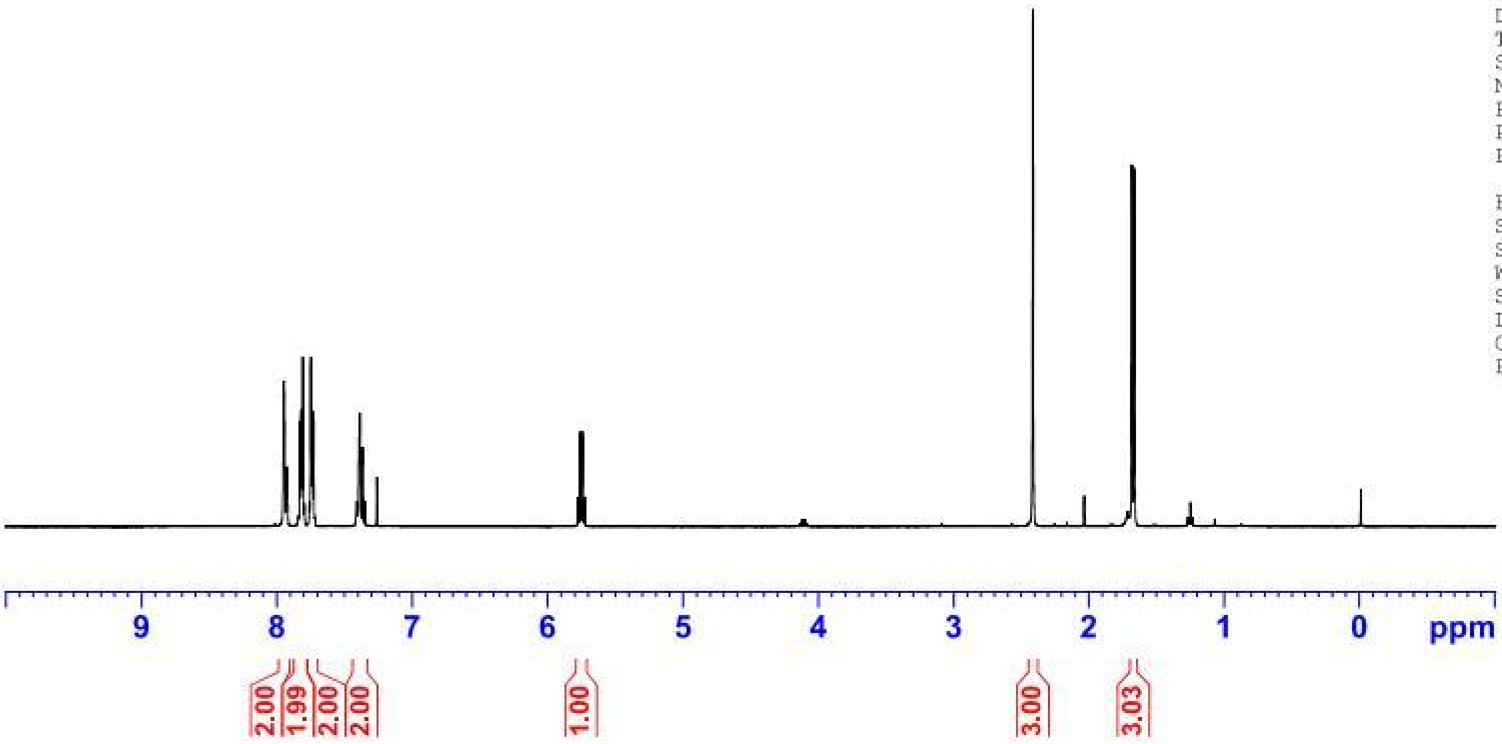

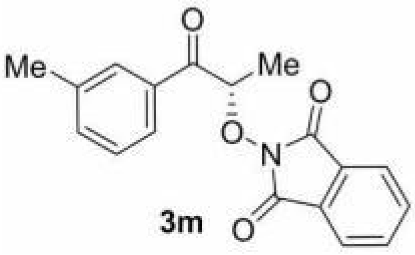

3m

Current Data Parameters
NAME qcx-6-50a
EXPNO 1
PROCNO 1

F2 - Acquisition Parameters
Date_ 20230310
Time 19.14 h
INSTRUM AvanceNeo 400MHz
PROBHD Z163739_0629 (
PULPROG zg30
TD 65536
SOLVENT CDCl3
NS 14
DS 2
SWH 8196.722 Hz
FIDRES 0.250144 Hz
AQ 3.9976959 sec
RG 101
DW 61.000 usec
DE 13.89 usec
TE 296.2 K
D1 1.00000000 sec
TD0 1
SFO1 400.1824711 MHz
NUC1 1H
P0 2.67 usec
P1 8.00 usec
PLW1 21.26700020 W

F2 - Processing parameters
SI 65536
SF 400.1800093 MHz
WDW EM
SSB 0
LB 0.30 Hz
GB 0
PC 1.00

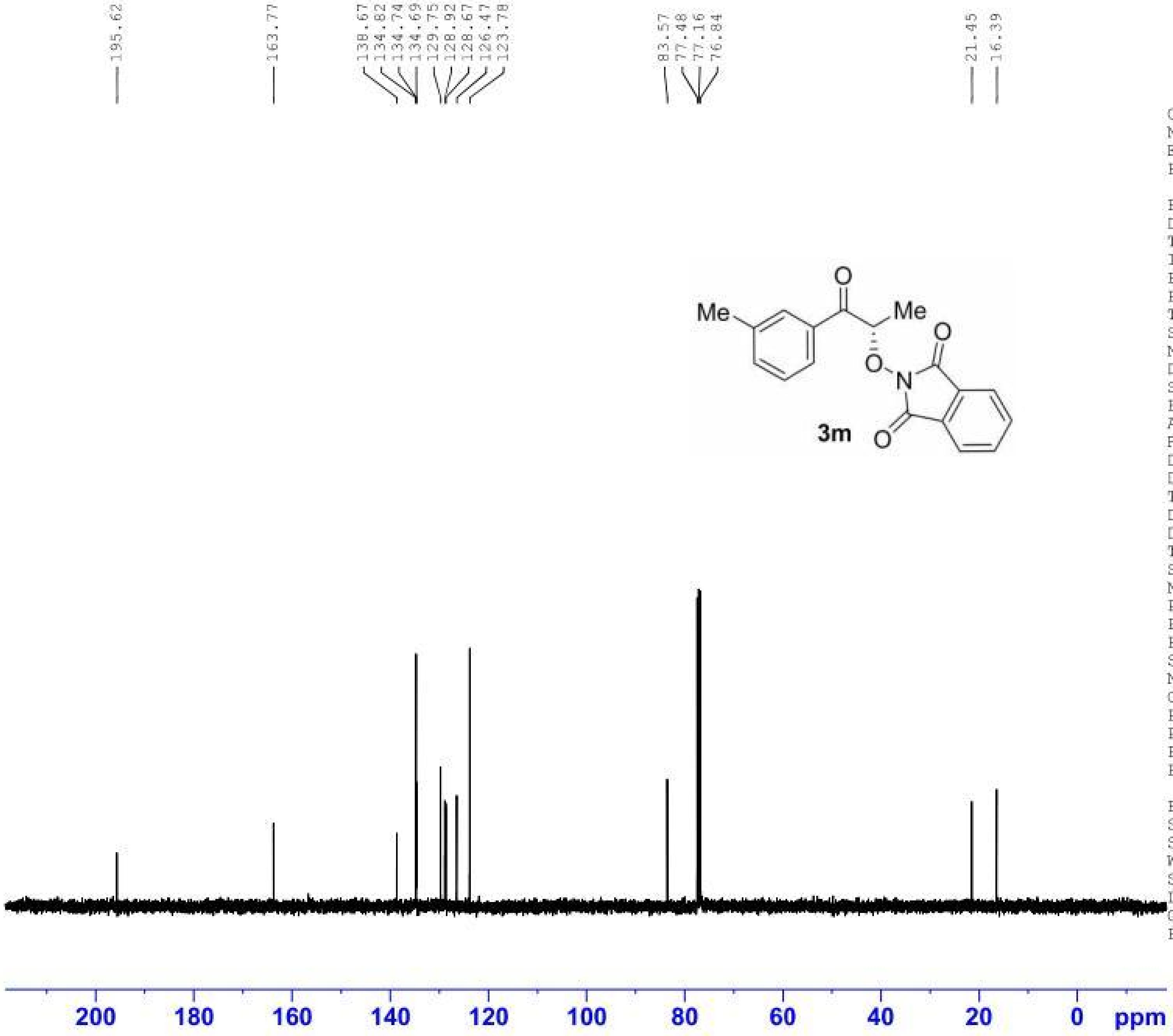

Current Data Parameters
NAME qcx-6-50a
EXPNO 2
PROCNO 1

F2 - Acquisition Parameters
Date_ 20230310
Time 19.18 h
INSTRUM AvanceNeo 400MHz
PROBHD Z163739_0629 (
PULPROG zgpg30
TD 65536
SOLVENT CDCl3
NS 25
DS 4
SWH 23809.523 Hz
FIDRES 0.726609 Hz
AQ 1.3762560 sec
RG 10
DW 21.000 usec
DE 6.50 usec
TE 296.5 K
D1 2.00000000 sec
D11 0.03000000 sec
TD0 1
SFO1 100.6354036 MHz
NUC1 13C
P0 2.67 usec
P1 8.00 usec
PLW1 85.25399780 W
SFO2 400.1816007 MHz
NUC2 1H
CPDPRG[2 waltz65
PCPD2 90.00 usec
PLW2 21.26700020 W
PLW12 0.16802999 W
PLW13 0.08452000 W

F2 - Processing parameters
SI 32768
SF 100.6253315 MHz
WDW EM
SSB 0
LB 1.00 Hz
GB 0
PC 1.40

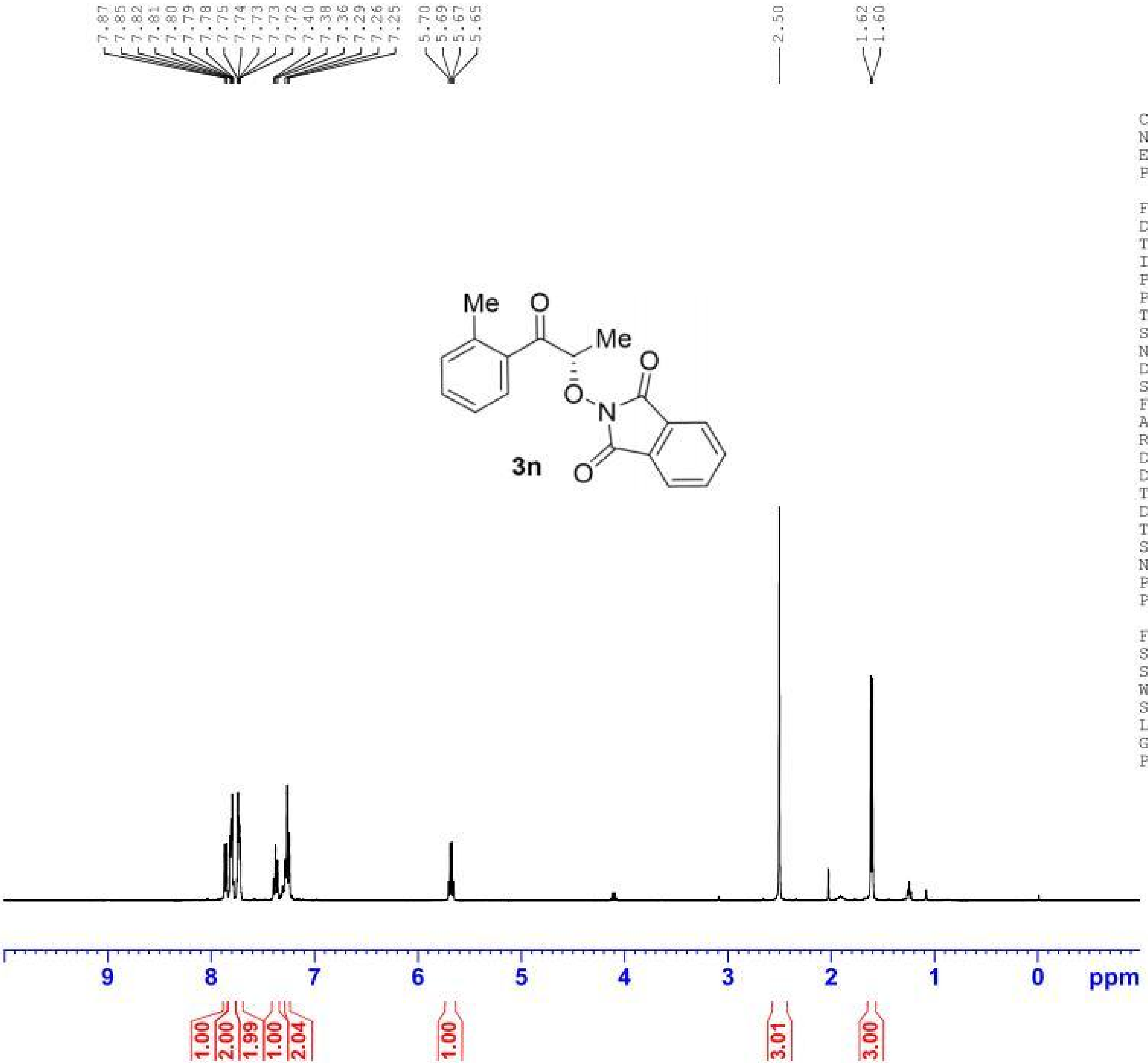

3n

```
Current Data Parameters
NAME          qcx-9-210b
EXPNO                  1
PROCNO                 1

F2 - Acquisition Parameters
Date_           20250725
Time               14.35 h
INSTRUM            spect
PROBHD   Z116098_0761 (
PULPROG             zg30
TD                 65536
SOLVENT            CDCl3
NS                     5
DS                     2
SWH             8012.820 Hz
FIDRES          0.244532 Hz
AQ             4.0894465 sec
RG                 31.57
DW                62.400 usec
DE                  6.50 usec
TE                 298.8 K
D1            1.00000000 sec
TD0                    1
SFO1         400.1324708 MHz
NUC1                  1H
P1                 10.00 usec
PLW1         16.24399948 W

F2 - Processing parameters
SI                 65536
SF           400.1300034 MHz
WDW                   EM
SSB                    0
LB                  0.30 Hz
GB                     0
PC                  1.00
```

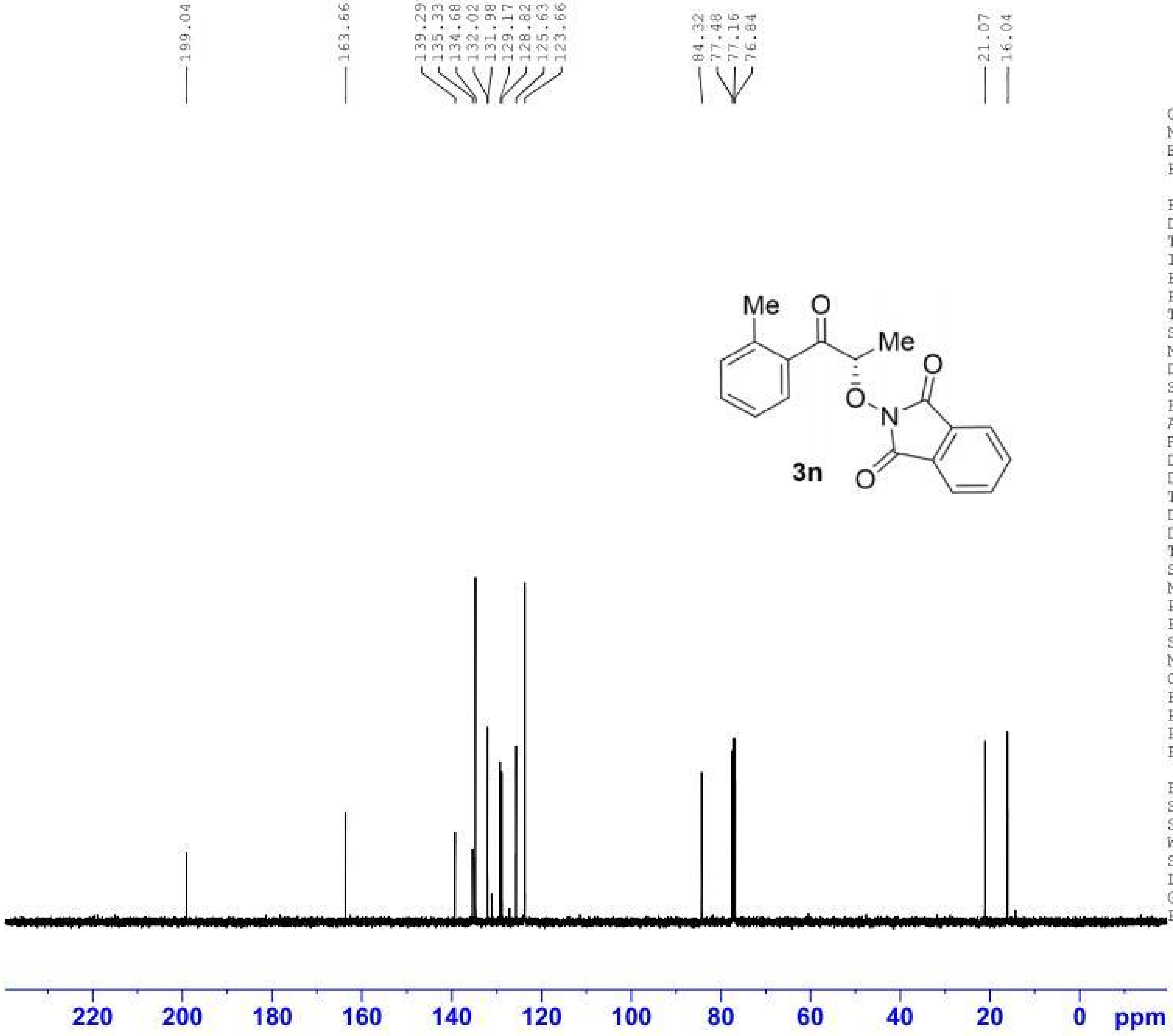

3n

Current Data Parameters
NAME qcx-9-210b
EXPNO 2
PROCNO 1

F2 - Acquisition Parameters
Date_ 20250725
Time 14.38 h
INSTRUM spect
PROBHD Z116098_0761 (
PULPROG zgpg30
TD 65536
SOLVENT CDCl3
NS 33
DS 4
SWH 26041.666 Hz
FIDRES 0.794729 Hz
AQ 1.2582912 sec
RG 198.89
DW 19.200 usec
DE 6.50 usec
TE 299.3 K
D1 2.00000000 sec
D11 0.03000000 sec
TD0 1
SFO1 100.6238359 MHz
NUC1 13C
P1 10.00 usec
PLW1 79.28600311 W
SFO2 400.1316005 MHz
NUC2 1H
CPDPRG[2 waltz16
PCPD2 90.00 usec
PLW2 16.24399948 W
PLW12 0.20054001 W
PLW13 0.10087000 W

F2 - Processing parameters
SI 32768
SF 100.6127648 MHz
WDW EM
SSB 0
LB 1.00 Hz
GB 0
PC 1.40

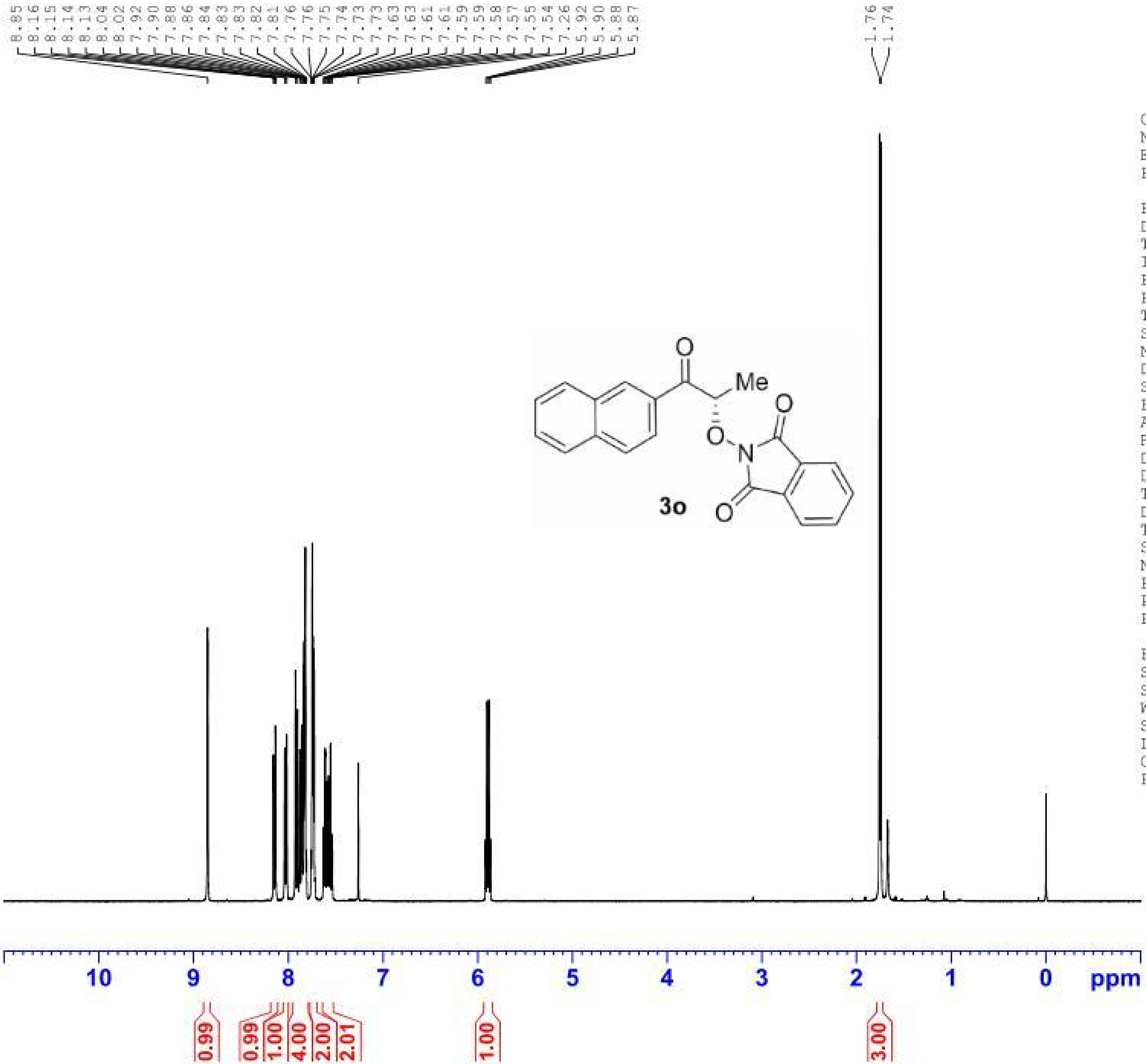

Current Data Parameters
NAME qcx-6-48a
EXPNO 1
PROCNO 1

F2 - Acquisition Parameters
Date_ 20230308
Time 19.35 h
INSTRUM AvanceNeo 400MHz
PROBHD Z163739_0629 (
PULPROG zg30
TD 65536
SOLVENT CDCl3
NS 7
DS 2
SWH 8196.722 Hz
FIDRES 0.250144 Hz
AQ 3.9976959 sec
RG 101
DW 61.000 usec
DE 13.89 usec
TE 295.8 K
D1 1.00000000 sec
TD0 1
SFO1 400.1824711 MHz
NUC1 1H
P0 2.67 usec
P1 8.00 usec
PLW1 21.26700020 W

F2 - Processing parameters
SI 65536
SF 400.1800092 MHz
WDW EM
SSB 0
LB 0.30 Hz
GB 0
PC 1.00

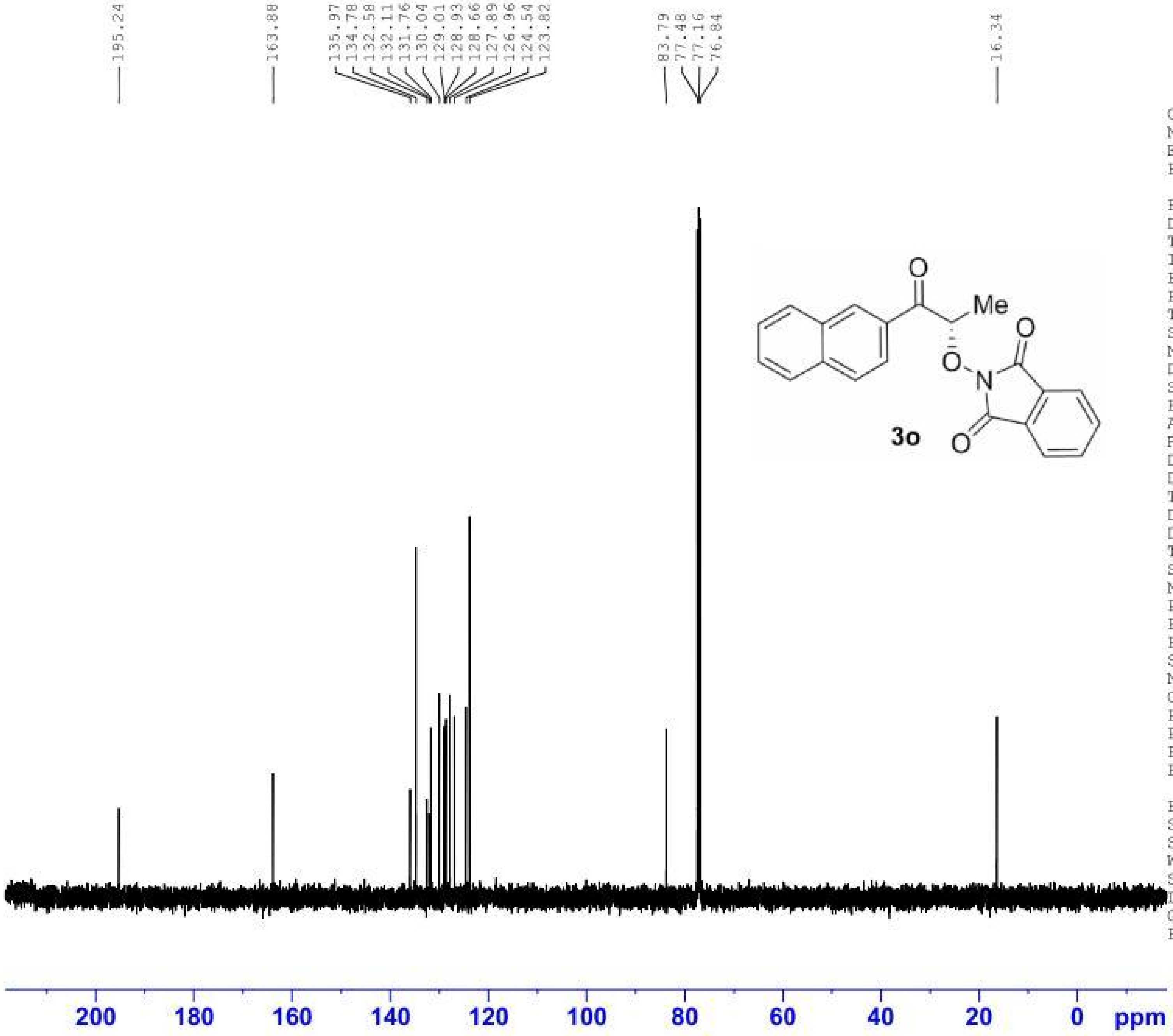

Current Data Parameters
NAME qcx-6-48a
EXPNO 2
PROCNO 1

F2 - Acquisition Parameters
Date_ 20230308
Time 19.38 h
INSTRUM AvanceNeo 400MHz
PROBHD Z163739_0629 (
PULPROG zgpg30
TD 65536
SOLVENT CDCl3
NS 33
DS 4
SWH 23809.523 Hz
FIDRES 0.726609 Hz
AQ 1.3762560 sec
RG 10
DW 21.000 usec
DE 6.50 usec
TE 296.3 K
D1 2.00000000 sec
D11 0.03000000 sec
TD0 1
SFO1 100.6354036 MHz
NUC1 13C
P0 2.67 usec
P1 8.00 usec
PLW1 85.25399780 W
SFO2 400.1816007 MHz
NUC2 1H
CPDPRG[2 waltz65
PCPD2 90.00 usec
PLW2 21.26700020 W
PLW12 0.16802999 W
PLW13 0.08452000 W

F2 - Processing parameters
SI 32768
SF 100.6253311 MHz
WDW EM
SSB 0
LB 1.00 Hz
GB 0
PC 1.40

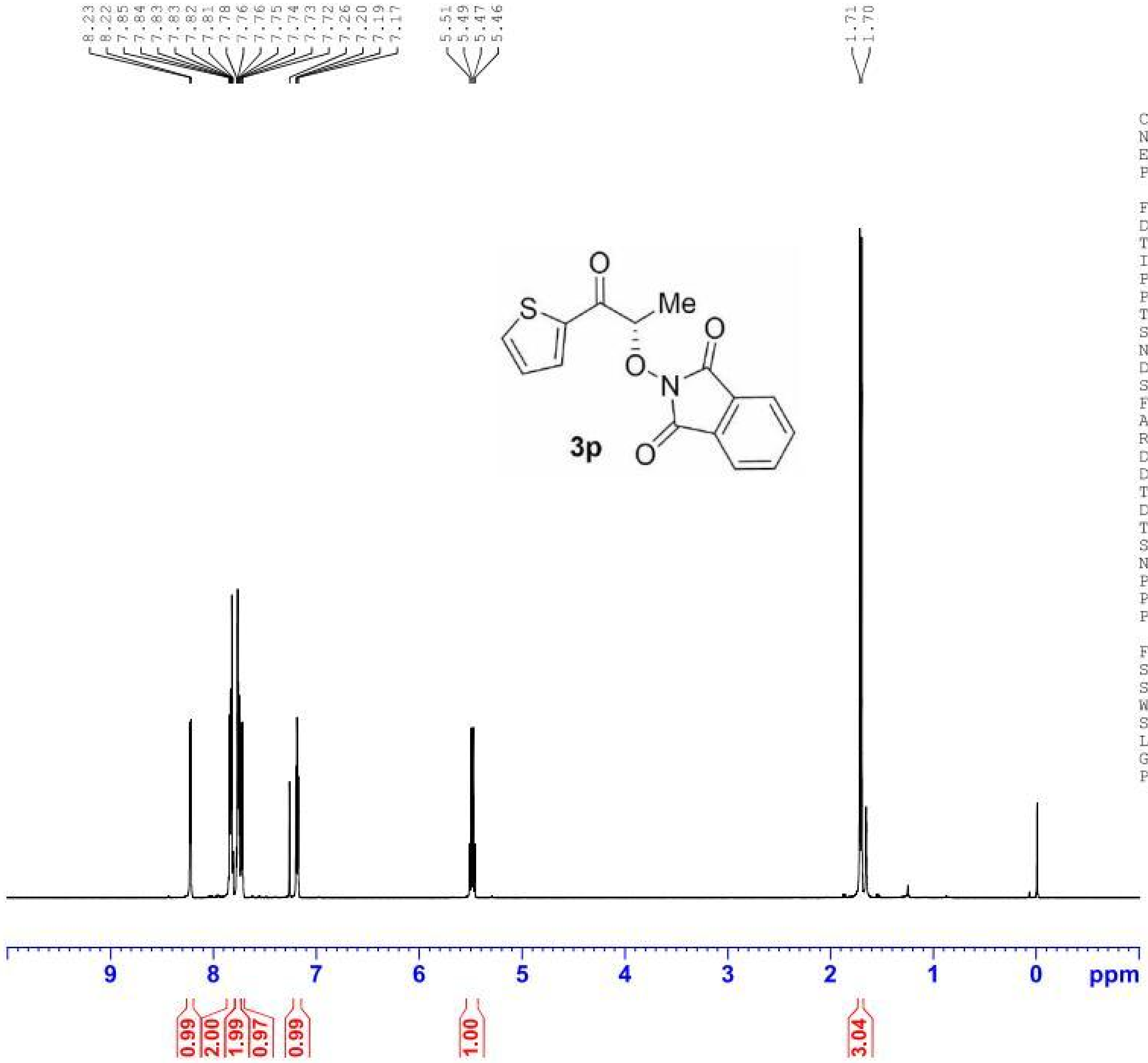

Current Data Parameters
NAME qcx-6-48b
EXPNO 1
PROCNO 1

F2 - Acquisition Parameters
Date_ 20230308
Time 19.42 h
INSTRUM AvanceNeo 400MHz
PROBHD Z163739_0629 (
PULPROG zg30
TD 65536
SOLVENT CDCl3
NS 4
DS 2
SWH 8196.722 Hz
FIDRES 0.250144 Hz
AQ 3.9976959 sec
RG 101
DW 61.000 usec
DE 13.89 usec
TE 295.9 K
D1 1.00000000 sec
TD0 1
SFO1 400.1824711 MHz
NUC1 1H
P0 2.67 usec
P1 8.00 usec
PLW1 21.26700020 W

F2 - Processing parameters
SI 65536
SF 400.1800093 MHz
WDW EM
SSB 0
LB 0.30 Hz
GB 0
PC 1.00

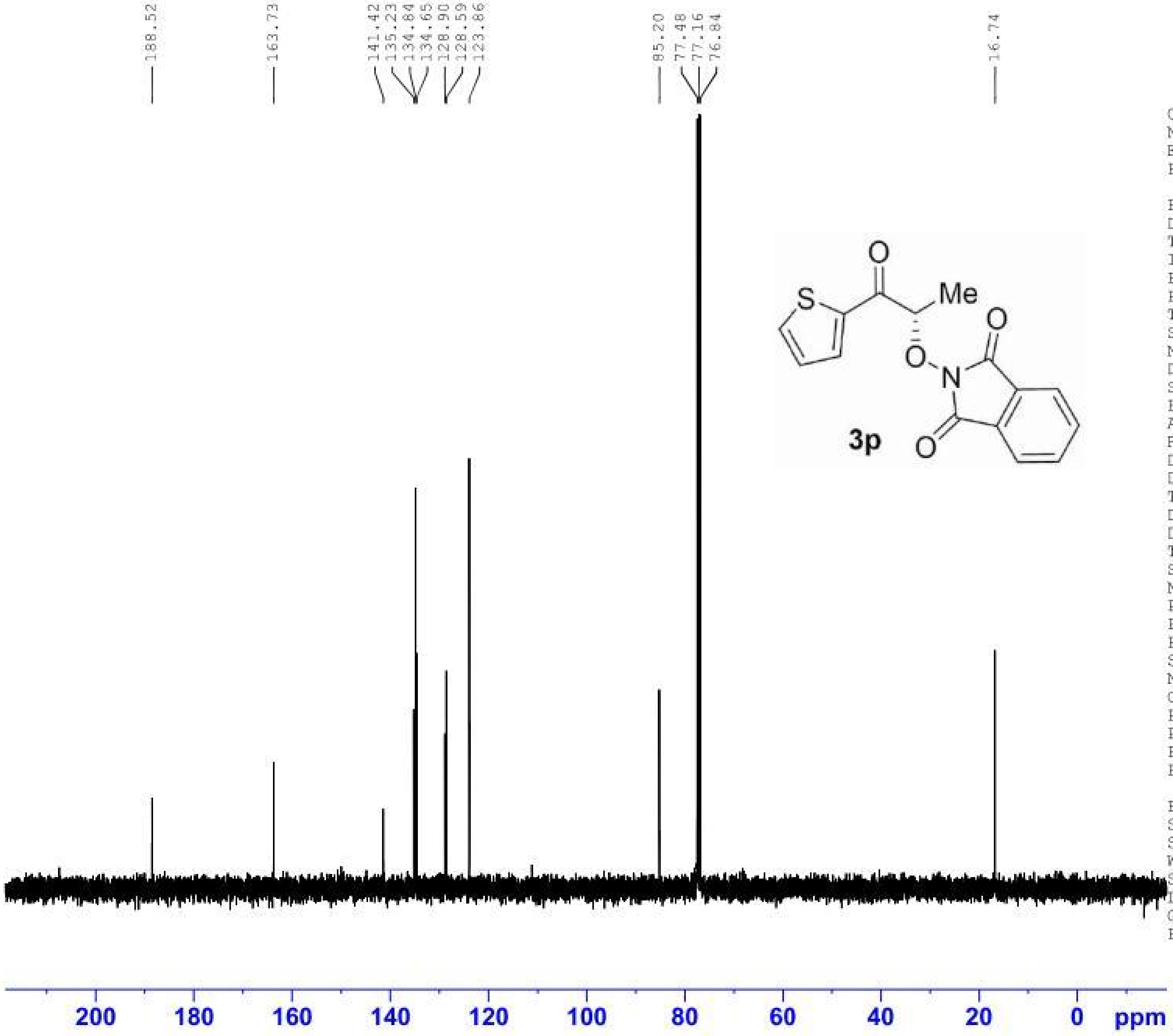

Current Data Parameters
NAME qcx-6-48b
EXPNO 2
PROCNO 1

F2 - Acquisition Parameters
Date_ 20230308
Time 19.46 h
INSTRUM AvanceNeo 400MHz
PROBHD Z163739_0629 (
PULPROG zgpg30
TD 65536
SOLVENT CDCl3
NS 37
DS 4
SWH 23809.523 Hz
FIDRES 0.726609 Hz
AQ 1.3762560 sec
RG 10
DW 21.000 usec
DE 6.50 usec
TE 296.1 K
D1 2.00000000 sec
D11 0.03000000 sec
TD0 1
SFO1 100.6354036 MHz
NUC1 13C
P0 2.67 usec
P1 8.00 usec
PLW1 85.25399780 W
SFO2 400.1816007 MHz
NUC2 1H
CPDPRG[2 waltz65
PCPD2 90.00 usec
PLW2 21.26700020 W
PLW12 0.16802999 W
PLW13 0.08452000 W

F2 - Processing parameters
SI 32768
SF 100.6253305 MHz
WDW EM
SSB 0
LB 1.00 Hz
GB 0
PC 1.40

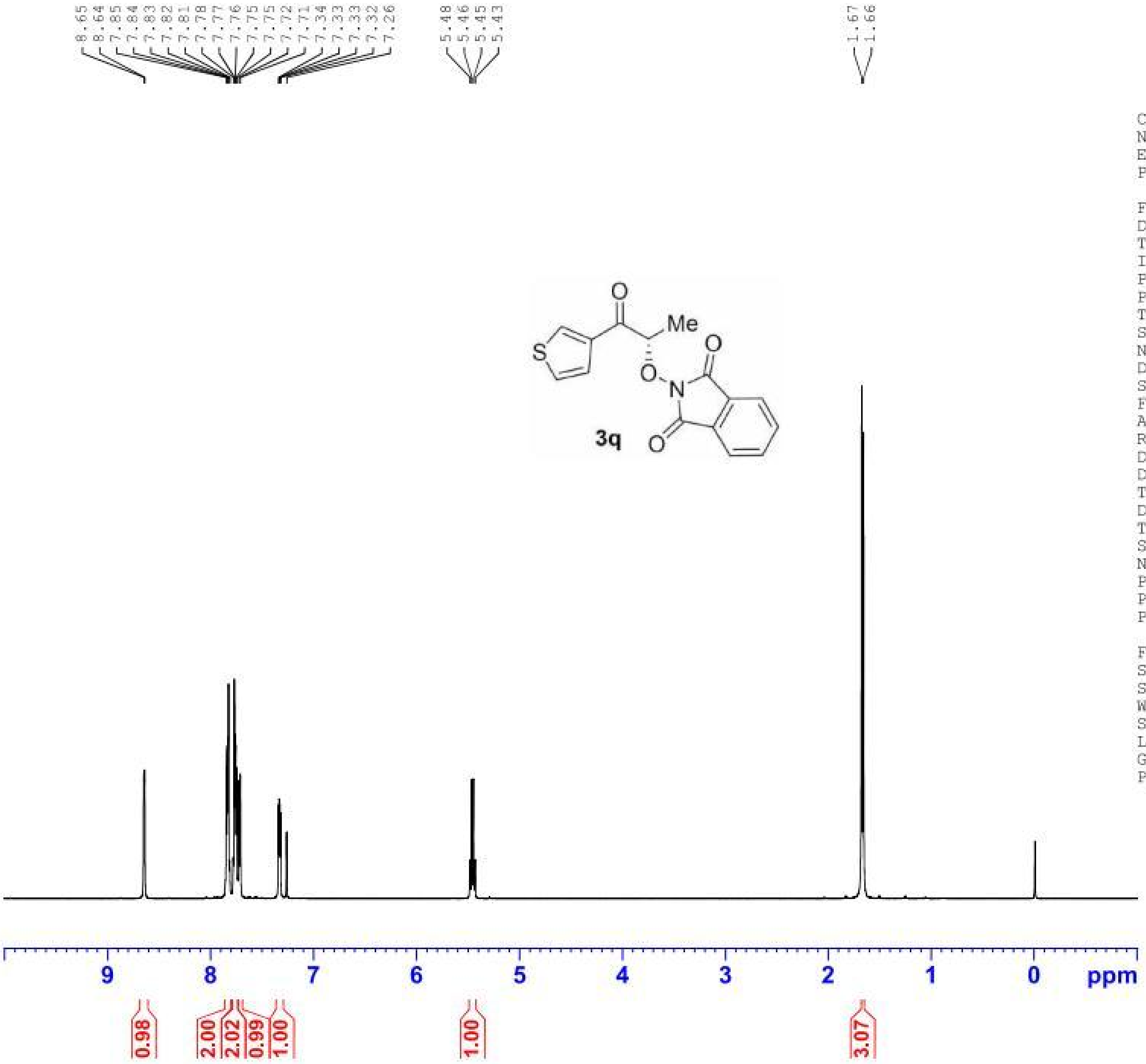


```
Current Data Parameters
NAME              qcx-6-60a
EXPNO                     1
PROCNO                    1

F2 - Acquisition Parameters
Date_              20230318
Time                  19.31 h
INSTRUM   AvanceNeo 400MHz
PROBHD    Z163739_0629 (
PULPROG               zg30
TD                   65536
SOLVENT              CDCl3
NS                       4
DS                       2
SWH               8196.722 Hz
FIDRES            0.250144 Hz
AQ               3.9976959 sec
RG                     101
DW                  61.000 usec
DE                   13.89 usec
TE                   297.0 K
D1              1.00000000 sec
TD0                      1
SFO1           400.1824711 MHz
NUC1                    1H
P0                    2.67 usec
P1                    8.00 usec
PLW1           21.26700020 W

F2 - Processing parameters
SI                   65536
SF             400.1800088 MHz
WDW                     EM
SSB                      0
LB                    0.30 Hz
GB                       0
PC                    1.00
```

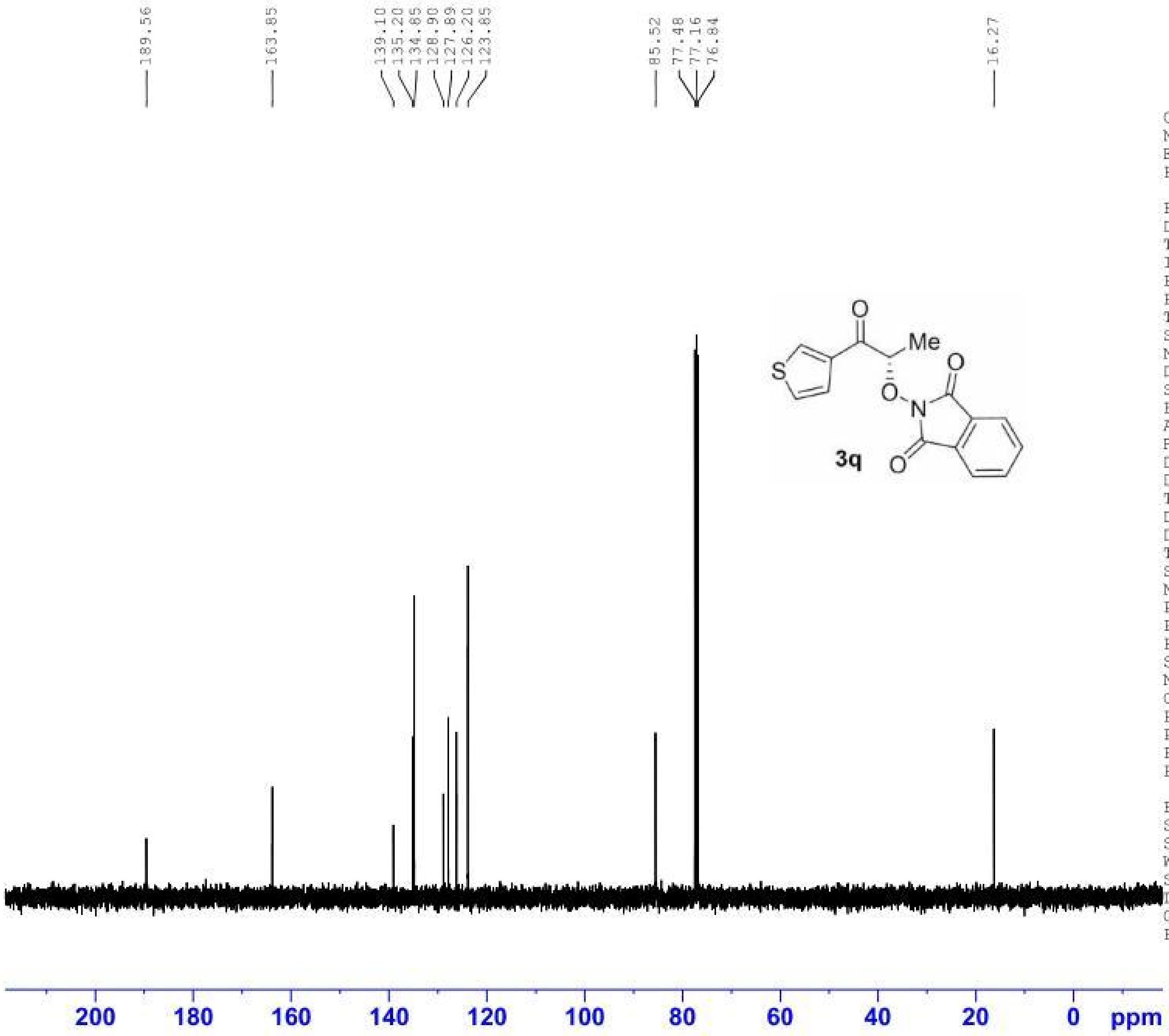

Current Data Parameters
NAME qcx-6-60a
EXPNO 2
PROCNO 1

F2 - Acquisition Parameters
Date_ 20230318
Time 19.33 h
INSTRUM AvanceNeo 400MHz
PROBHD Z163739_0629 (
PULPROG zgpg30
TD 65536
SOLVENT CDCl3
NS 25
DS 4
SWH 23809.523 Hz
FIDRES 0.726609 Hz
AQ 1.3762560 sec
RG 10
DW 21.000 usec
DE 6.50 usec
TE 297.3 K
D1 2.00000000 sec
D11 0.03000000 sec
TD0 1
SFO1 100.6354036 MHz
NUC1 13C
P0 2.67 usec
P1 8.00 usec
PLW1 85.25399780 W
SFO2 400.1816007 MHz
NUC2 1H
CPDPRG[2 waltz65
PCPD2 90.00 usec
PLW2 21.26700020 W
PLW12 0.16802999 W
PLW13 0.08452000 W

F2 - Processing parameters
SI 32768
SF 100.6253303 MHz
WDW EM
SSB 0
LB 1.00 Hz
GB 0
PC 1.40

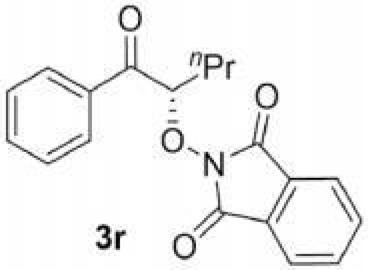

3r

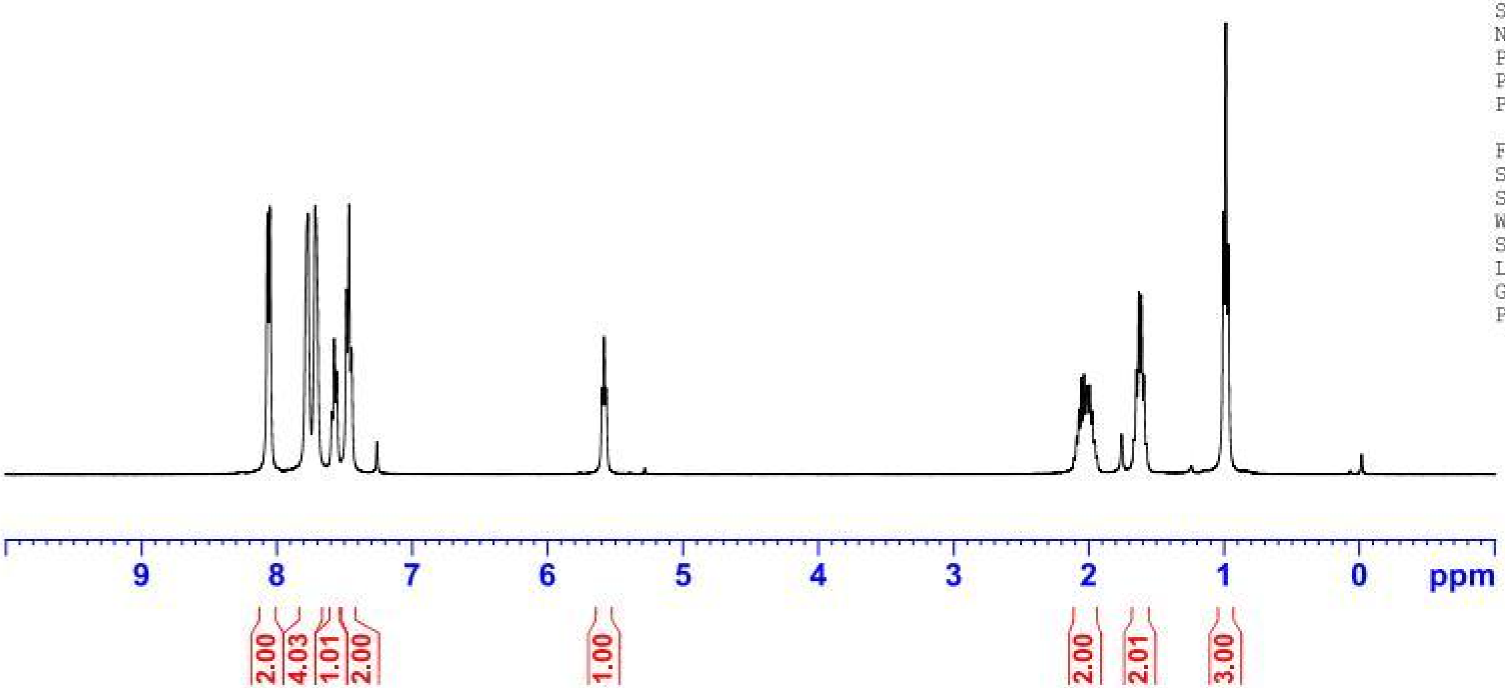


```
Current Data Parameters
NAME          qcx-6-28b
EXPNO                 1
PROCNO                1

F2 - Acquisition Parameters
Date_          20230224
Time              22.33 h
INSTRUM   AvanceNeo 400MHz
PROBHD   Z163739_0629 (
PULPROG            zg30
TD                65536
SOLVENT           CDCl3
NS                    4
DS                    2
SWH            8196.722 Hz
FIDRES         0.250144 Hz
AQ            3.9976959 sec
RG                  101
DW               61.000 usec
DE                13.89 usec
TE                295.7 K
D1           1.00000000 sec
TD0                   1
SFO1        400.1824711 MHz
NUC1                 1H
P0                 2.67 usec
P1                 8.00 usec
PLW1        21.26700020 W

F2 - Processing parameters
SI                65536
SF          400.1800087 MHz
WDW                  EM
SSB                   0
LB                 0.30 Hz
GB                    0
PC                 1.00
```

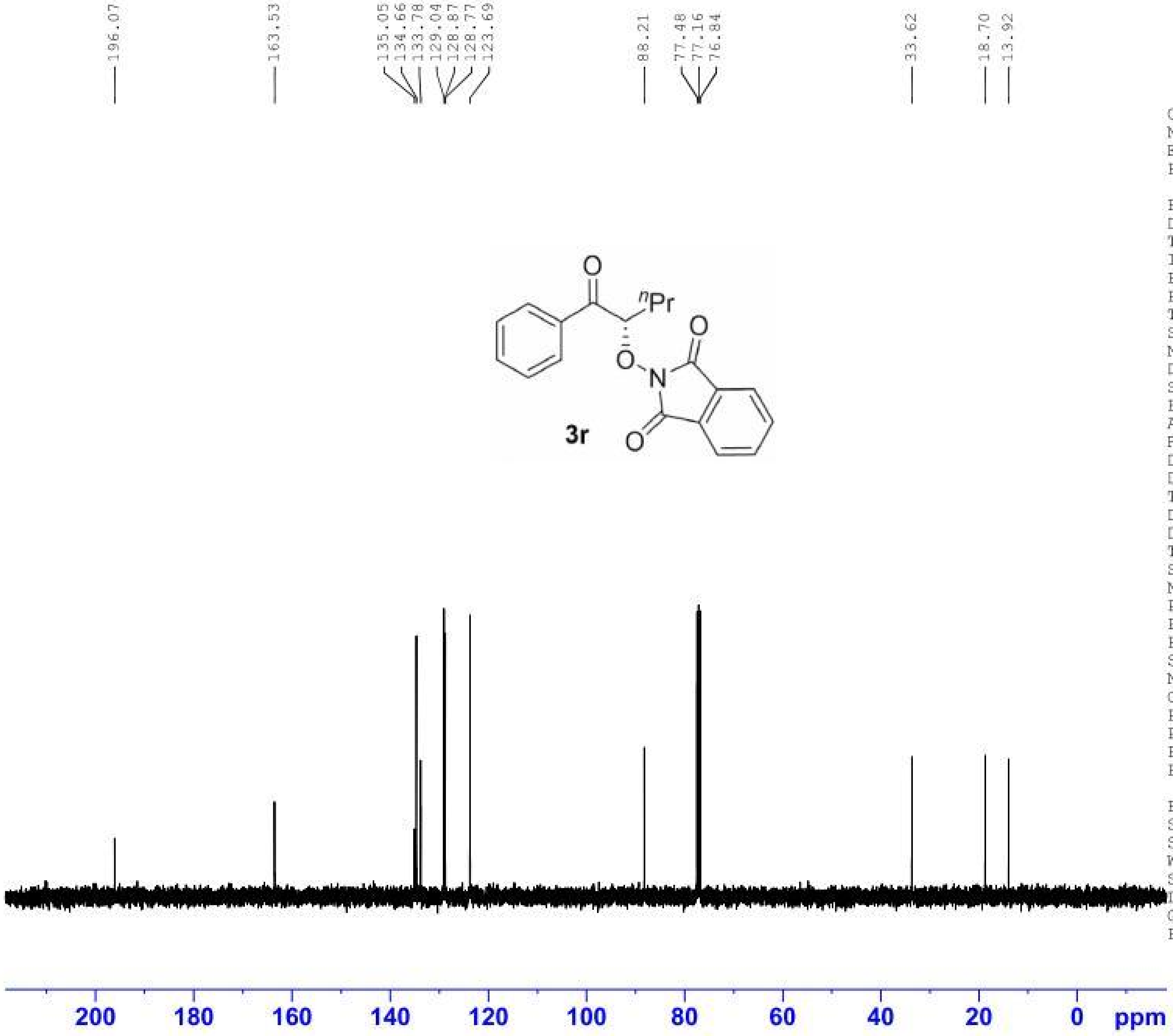


```
Current Data Parameters
NAME          qcx-6-28b
EXPNO                 2
PROCNO                1

F2 - Acquisition Parameters
Date_          20230224
Time              22.36 h
INSTRUM   AvanceNeo 400MHz
PROBHD    Z163739_0629 (
PULPROG          zgpg30
TD                65536
SOLVENT           CDCl3
NS                   16
DS                    4
SWH           23809.523 Hz
FIDRES         0.726609 Hz
AQ            1.3762560 sec
RG                   10
DW               21.000 usec
DE                 6.50 usec
TE                296.2 K
D1           2.00000000 sec
D11          0.03000000 sec
TD0                   1
SFO1        100.6354036 MHz
NUC1                13C
P0                 2.67 usec
P1                 8.00 usec
PLW1        85.25399780 W
SFO2        400.1816007 MHz
NUC2                 1H
CPDPRG[2         waltz65
PCPD2             90.00 usec
PLW2        21.26700020 W
PLW12        0.16802999 W
PLW13        0.08452000 W

F2 - Processing parameters
SI                32768
SF          100.6253339 MHz
WDW                  EM
SSB                   0
LB                 1.00 Hz
GB                    0
PC                 1.40
```

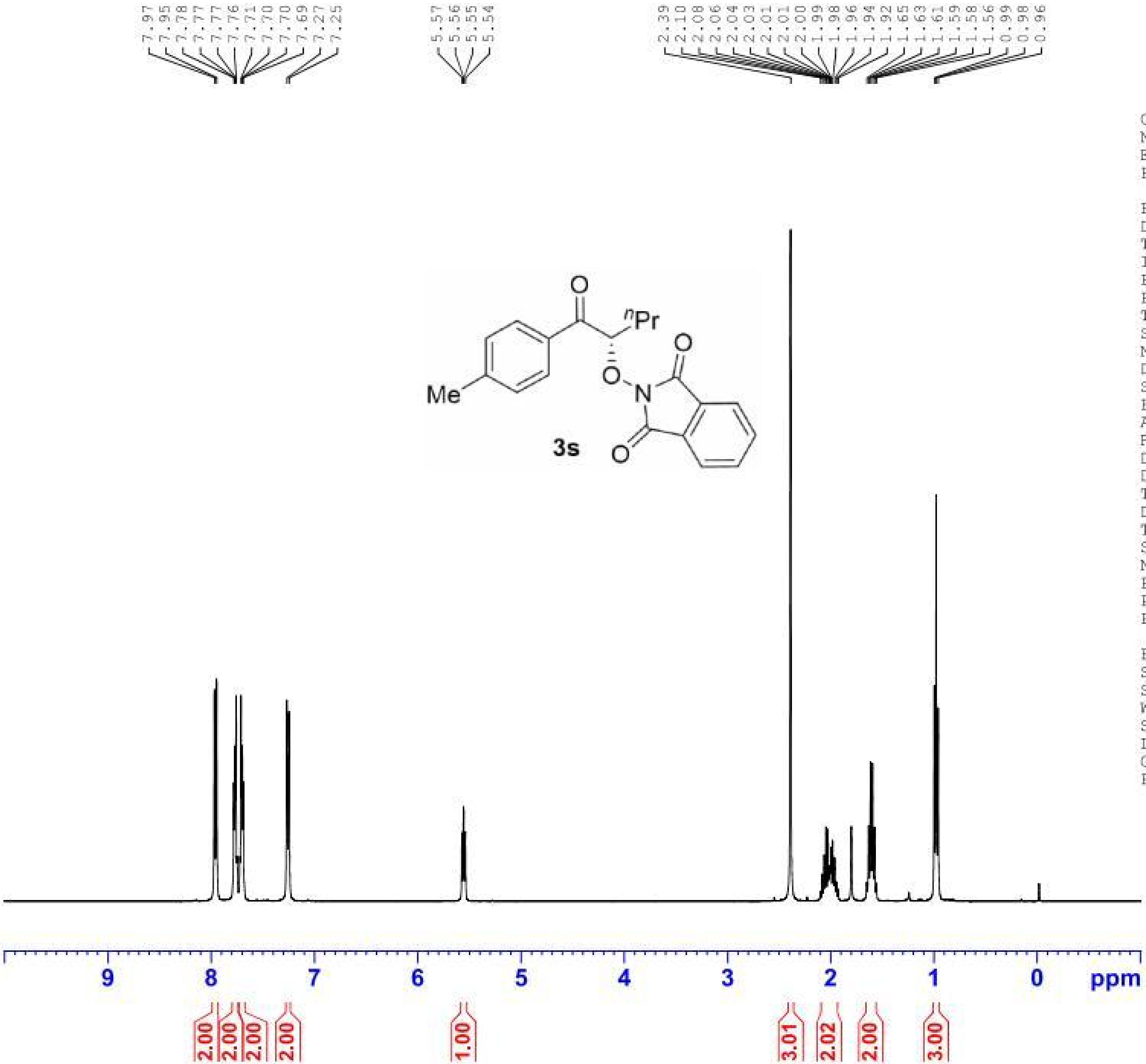

Current Data Parameters
NAME qcx-6-117a
EXPNO 1
PROCNO 1

F2 - Acquisition Parameters
Date_ 20230429
Time 19.37 h
INSTRUM AvanceNeo 400MHz
PROBHD Z163739_0629 (
PULPROG zg30
TD 65536
SOLVENT CDCl3
NS 9
DS 2
SWH 8196.722 Hz
FIDRES 0.250144 Hz
AQ 3.9976959 sec
RG 101
DW 61.000 usec
DE 13.89 usec
TE 298.4 K
D1 1.00000000 sec
TD0 1
SFO1 400.1824711 MHz
NUC1 1H
P0 2.67 usec
P1 8.00 usec
PLW1 21.26700020 W

F2 - Processing parameters
SI 65536
SF 400.1800093 MHz
WDW EM
SSB 0
LB 0.30 Hz
GB 0
PC 1.00

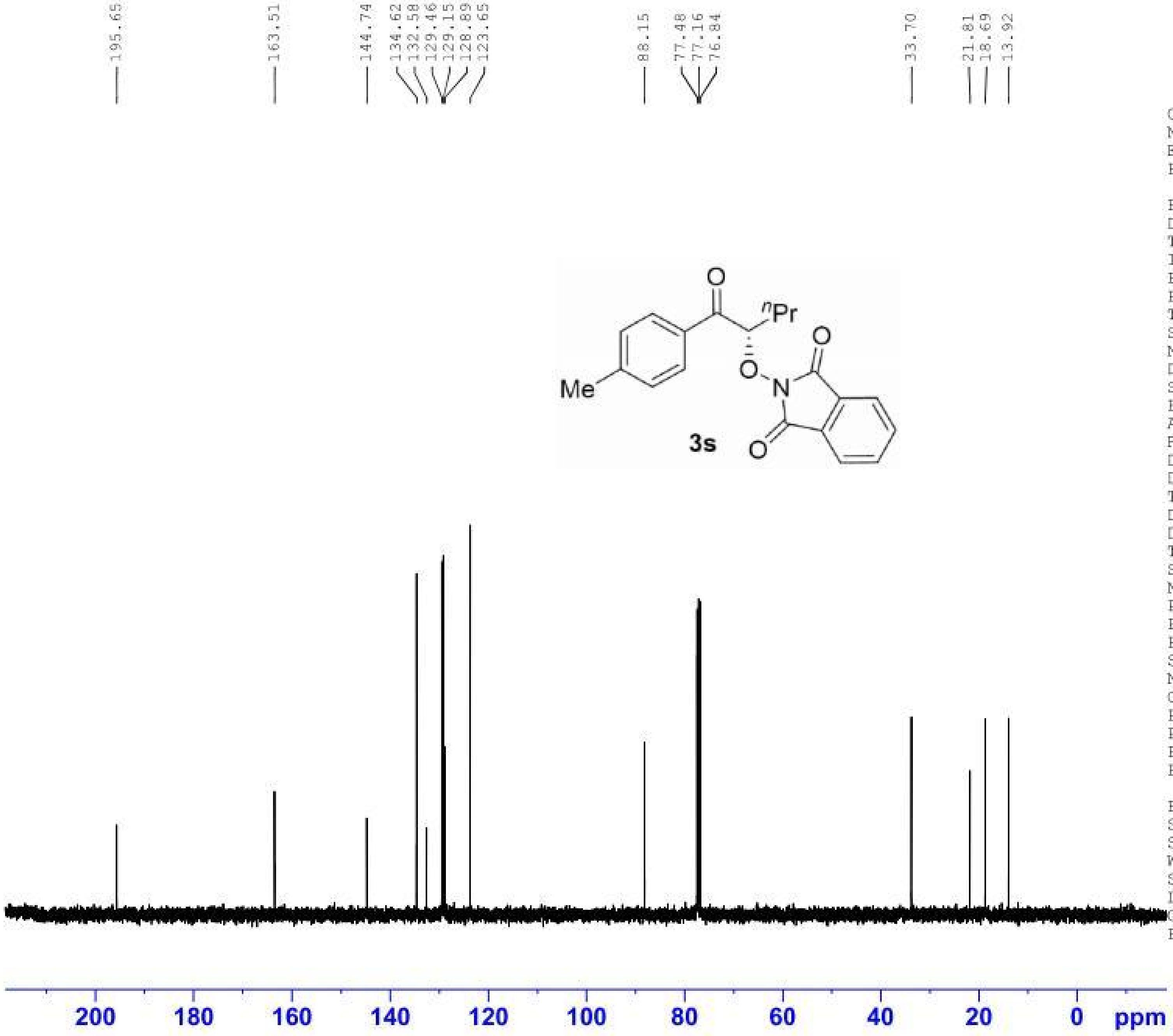

Current Data Parameters
NAME qcx-6-117a
EXPNO 2
PROCNO 1

F2 - Acquisition Parameters
Date_ 20230429
Time 19.40 h
INSTRUM AvanceNeo 400MHz
PROBHD Z163739_0629 (
PULPROG zgpg30
TD 65536
SOLVENT CDCl3
NS 19
DS 4
SWH 23809.523 Hz
FIDRES 0.726609 Hz
AQ 1.3762560 sec
RG 16
DW 21.000 usec
DE 6.50 usec
TE 298.7 K
D1 2.00000000 sec
D11 0.03000000 sec
TD0 1
SFO1 100.6354036 MHz
NUC1 13C
P0 2.67 usec
P1 8.00 usec
PLW1 85.25399780 W
SFO2 400.1816007 MHz
NUC2 1H
CPDPRG[2 waltz65
PCPD2 90.00 usec
PLW2 21.26700020 W
PLW12 0.16802999 W
PLW13 0.08452000 W

F2 - Processing parameters
SI 32768
SF 100.6253344 MHz
WDW EM
SSB 0
LB 1.00 Hz
GB 0
PC 1.40

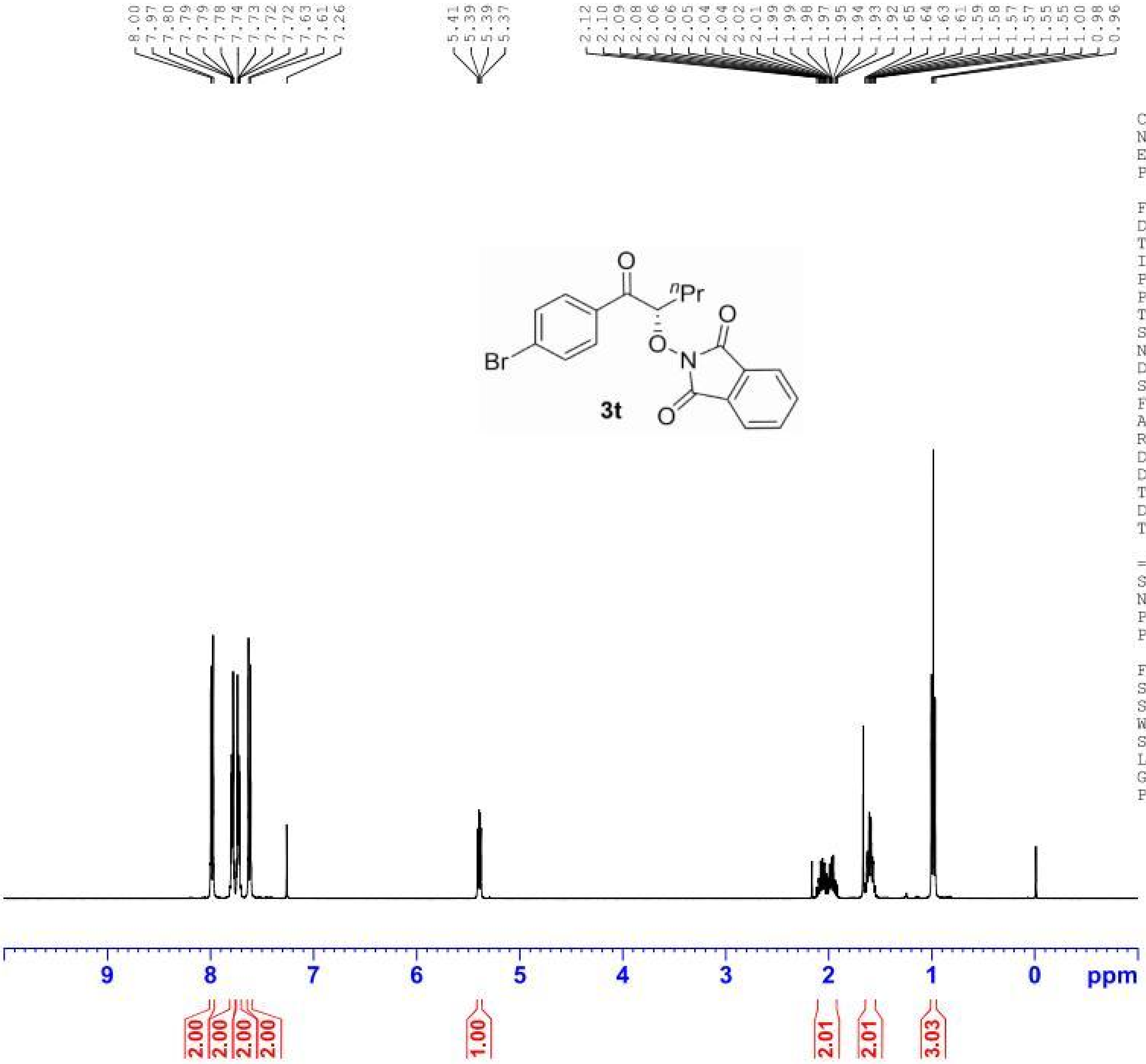

Current Data Parameters
NAME qcx-6-74-d
EXPNO 1
PROCNO 1

F2 - Acquisition Parameters
Date_ 20230325
Time 19.25
INSTRUM spect
PROBHD 5 mm PABBO BB/
PULPROG zg30
TD 65536
SOLVENT CDCl3
NS 2
DS 2
SWH 8012.820 Hz
FIDRES 0.122266 Hz
AQ 4.0894465 sec
RG 70.97
DW 62.400 usec
DE 6.50 usec
TE 296.4 K
D1 1.00000000 sec
TD0 1

======== CHANNEL f1 ========
SFO1 400.1324710 MHz
NUC1 1H
P1 14.50 usec
PLW1 11.99499989 W

F2 - Processing parameters
SI 65536
SF 400.1300101 MHz
WDW EM
SSB 0
LB 0.30 Hz
GB 0
PC 1.00

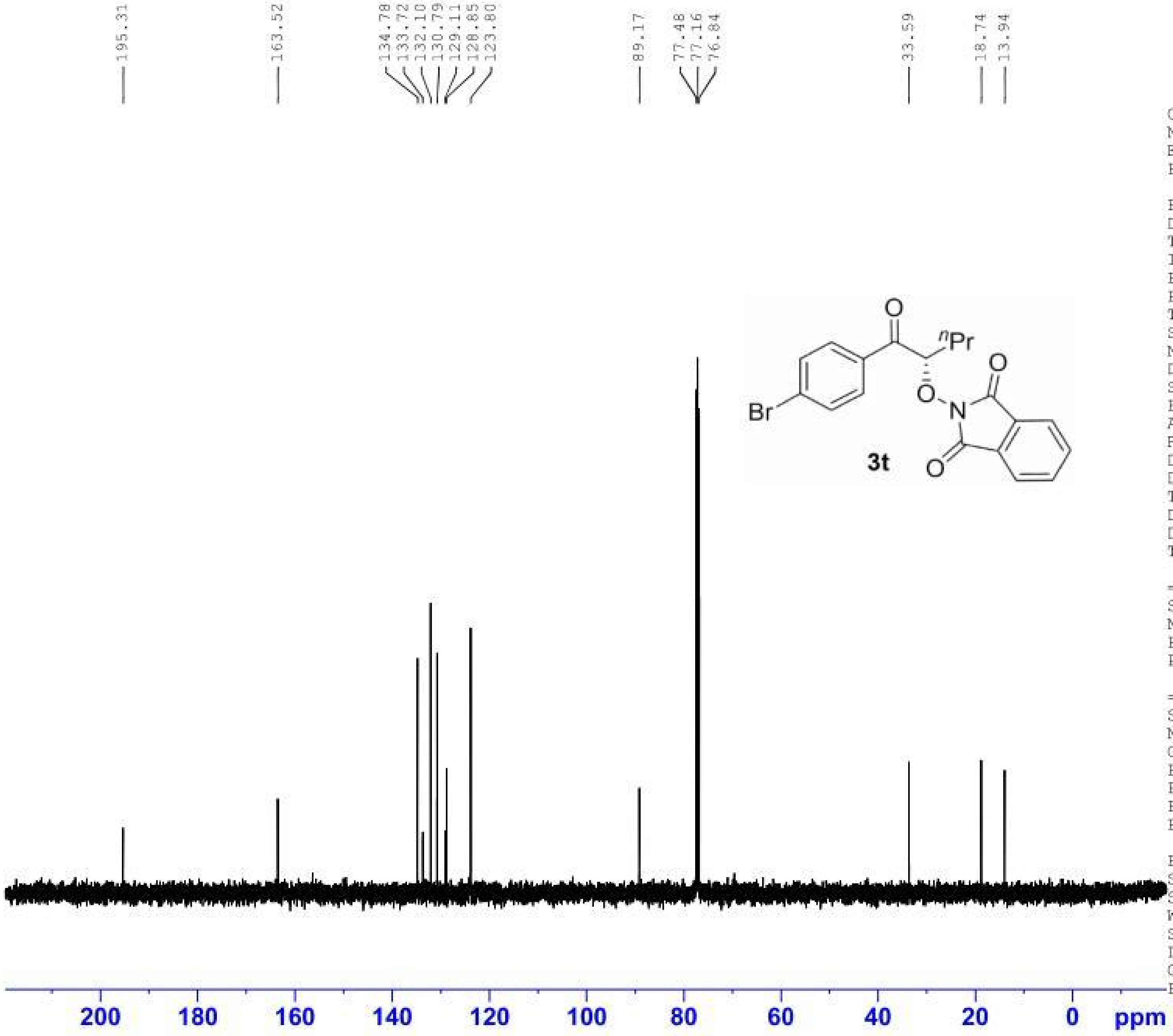


```
Current Data Parameters
NAME          qcx-6-74-d
EXPNO                  2
PROCNO                 1

F2 - Acquisition Parameters
Date_           20230325
Time               19.27
INSTRUM            spect
PROBHD   5 mm PABBO BB/
PULPROG          zgpg30
TD                 65536
SOLVENT            CDCl3
NS                    30
DS                     2
SWH            24038.461 Hz
FIDRES          0.366798 Hz
AQ             1.3631488 sec
RG                196.92
DW                20.800 usec
DE                  6.50 usec
TE                 297.0 K
D1            2.00000000 sec
D11           0.03000000 sec
TD0                    1

======== CHANNEL f1 ========
SFO1         100.6228298 MHz
NUC1                 13C
P1                  9.70 usec
PLW1         46.98899841 W

======== CHANNEL f2 ========
SFO2         400.1316005 MHz
NUC2                  1H
CPDPRG[2         waltz16
PCPD2              90.00 usec
PLW2         11.99499989 W
PLW12         0.34213999 W
PLW13         0.27713001 W

F2 - Processing parameters
SI                 32768
SF           100.6127584 MHz
WDW                   EM
SSB                    0
LB                  1.00 Hz
GB                     0
PC                  1.40
```

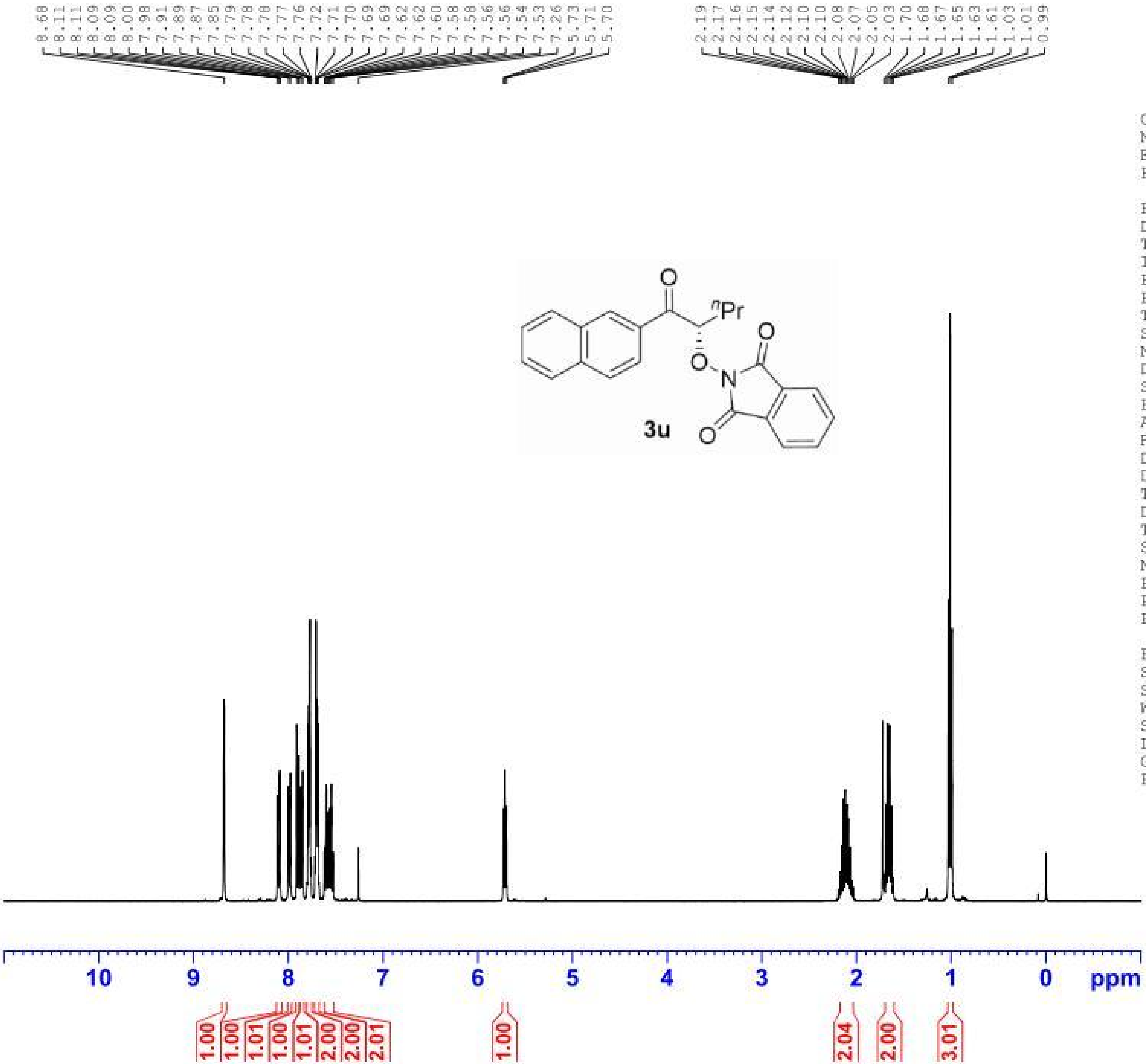


```
Current Data Parameters
NAME          qcx-6-117b
EXPNO                  1
PROCNO                 1

F2 - Acquisition Parameters
Date_           20230429
Time               19.29 h
INSTRUM   AvanceNeo 400MHz
PROBHD    Z163739_0629 (
PULPROG             zg30
TD                 65536
SOLVENT            CDCl3
NS                     4
DS                     2
SWH             8196.722 Hz
FIDRES          0.250144 Hz
AQ             3.9976959 sec
RG                   101
DW                61.000 usec
DE                 13.89 usec
TE                 298.6 K
D1            1.00000000 sec
TD0                    1
SFO1         400.1824711 MHz
NUC1                  1H
P0                  2.67 usec
P1                  8.00 usec
PLW1         21.26700020 W

F2 - Processing parameters
SI                 65536
SF           400.1800092 MHz
WDW                   EM
SSB                    0
LB                  0.30 Hz
GB                     0
PC                  1.00
```

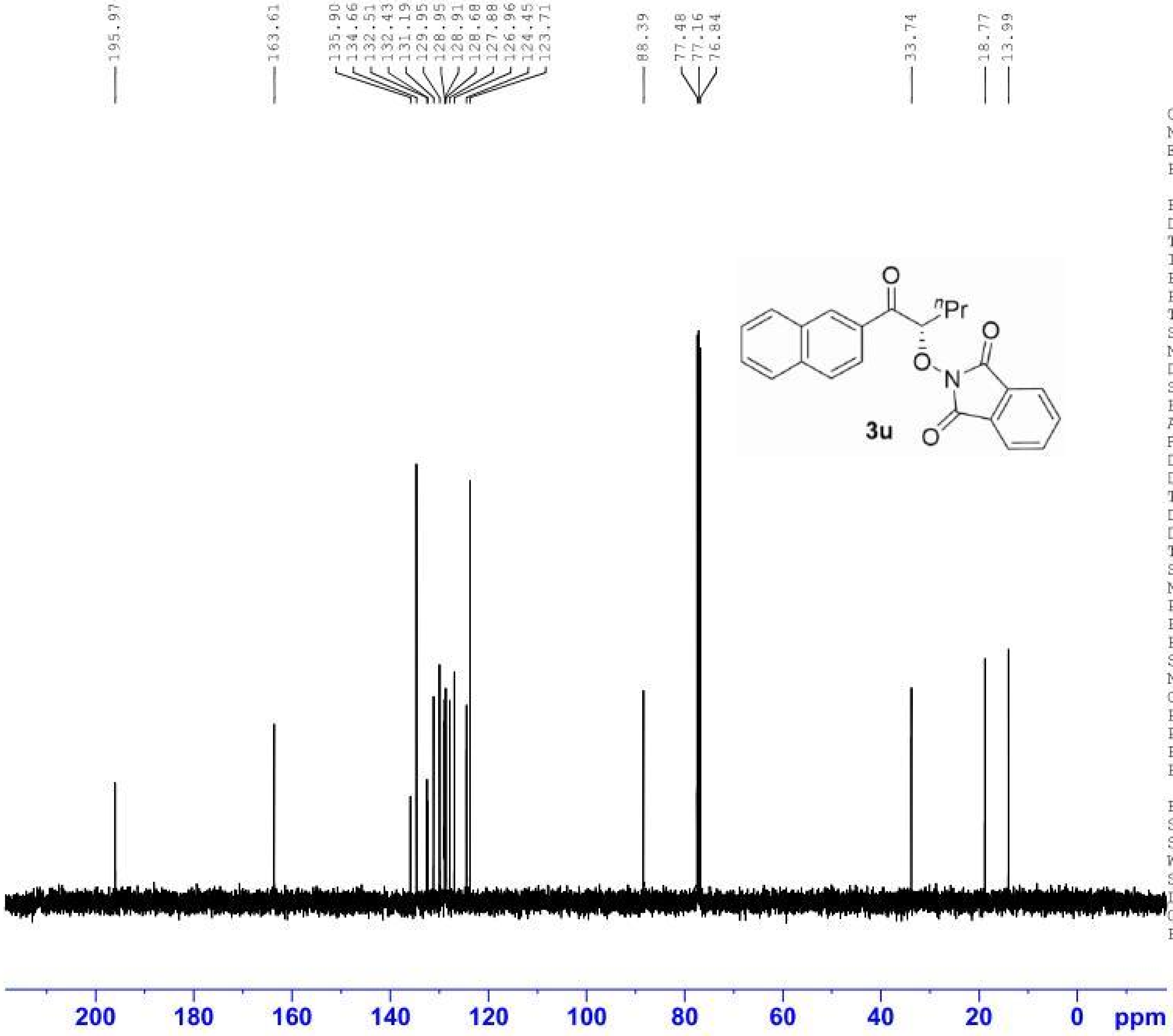

3u

Current Data Parameters
NAME qcx-6-117b
EXPNO 2
PROCNO 1

F2 - Acquisition Parameters
Date_ 20230429
Time 19.32 h
INSTRUM AvanceNeo 400MHz
PROBHD Z163739_0629 (
PULPROG zgpg30
TD 65536
SOLVENT CDCl3
NS 21
DS 4
SWH 23809.523 Hz
FIDRES 0.726609 Hz
AQ 1.3762560 sec
RG 10
DW 21.000 usec
DE 6.50 usec
TE 298.8 K
D1 2.00000000 sec
D11 0.03000000 sec
TD0 1
SFO1 100.6354036 MHz
NUC1 13C
P0 2.67 usec
P1 8.00 usec
PLW1 85.25399780 W
SFO2 400.1816007 MHz
NUC2 1H
CPDPRG[2 waltz65
PCPD2 90.00 usec
PLW2 21.26700020 W
PLW12 0.16802999 W
PLW13 0.08452000 W

F2 - Processing parameters
SI 32768
SF 100.6253325 MHz
WDW EM
SSB 0
LB 1.00 Hz
GB 0
PC 1.40

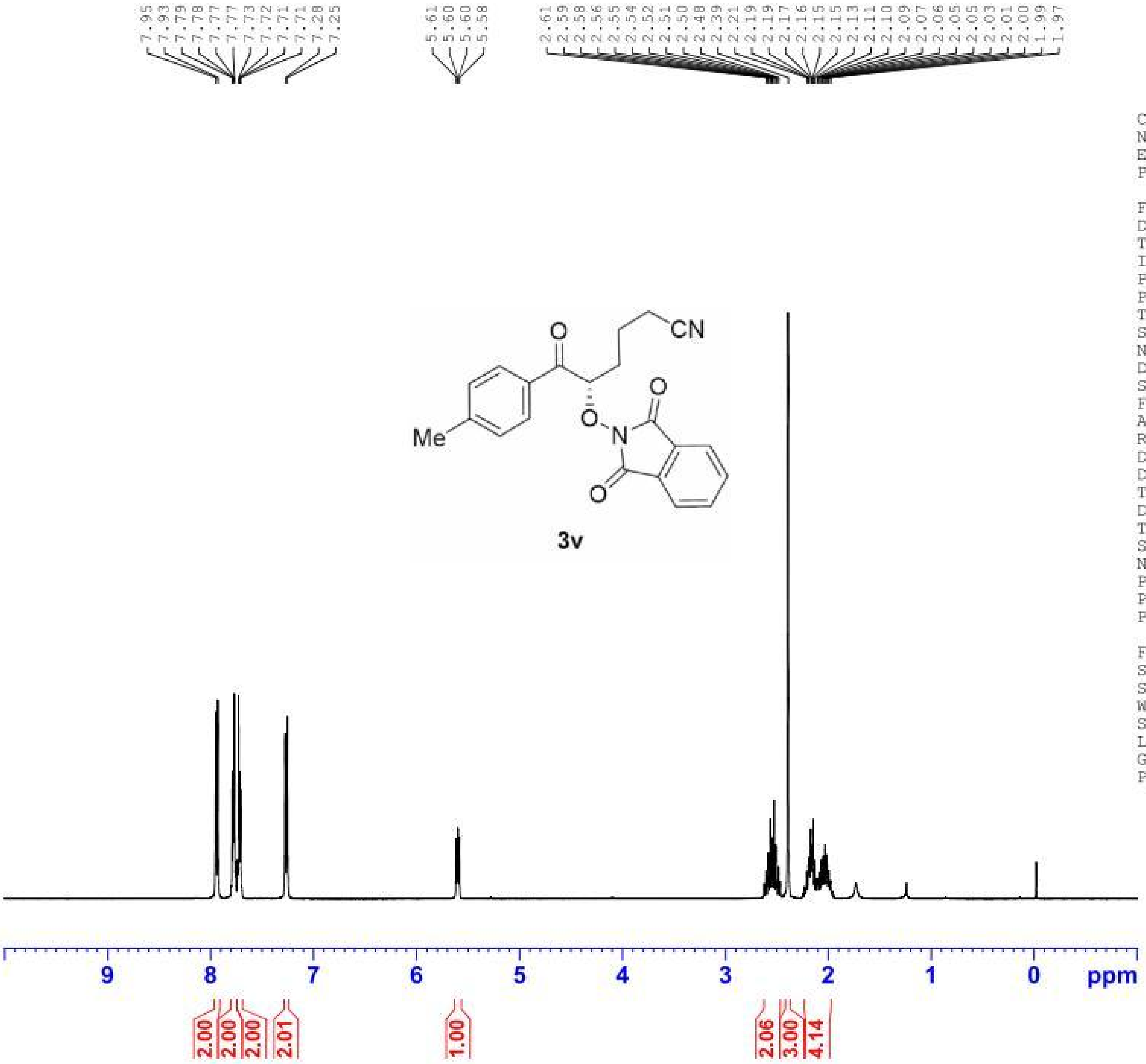

Current Data Parameters
NAME qcx-6-117c
EXPNO 1
PROCNO 1

F2 - Acquisition Parameters
Date_ 20230429
Time 19.21 h
INSTRUM AvanceNeo 400MHz
PROBHD Z163739_0629 (
PULPROG zg30
TD 65536
SOLVENT CDCl3
NS 2
DS 2
SWH 8196.722 Hz
FIDRES 0.250144 Hz
AQ 3.9976959 sec
RG 101
DW 61.000 usec
DE 13.89 usec
TE 299.2 K
D1 1.00000000 sec
TD0 1
SFO1 400.1824711 MHz
NUC1 1H
P0 2.67 usec
P1 8.00 usec
PLW1 21.26700020 W

F2 - Processing parameters
SI 65536
SF 400.1800117 MHz
WDW EM
SSB 0
LB 0.30 Hz
GB 0
PC 1.00

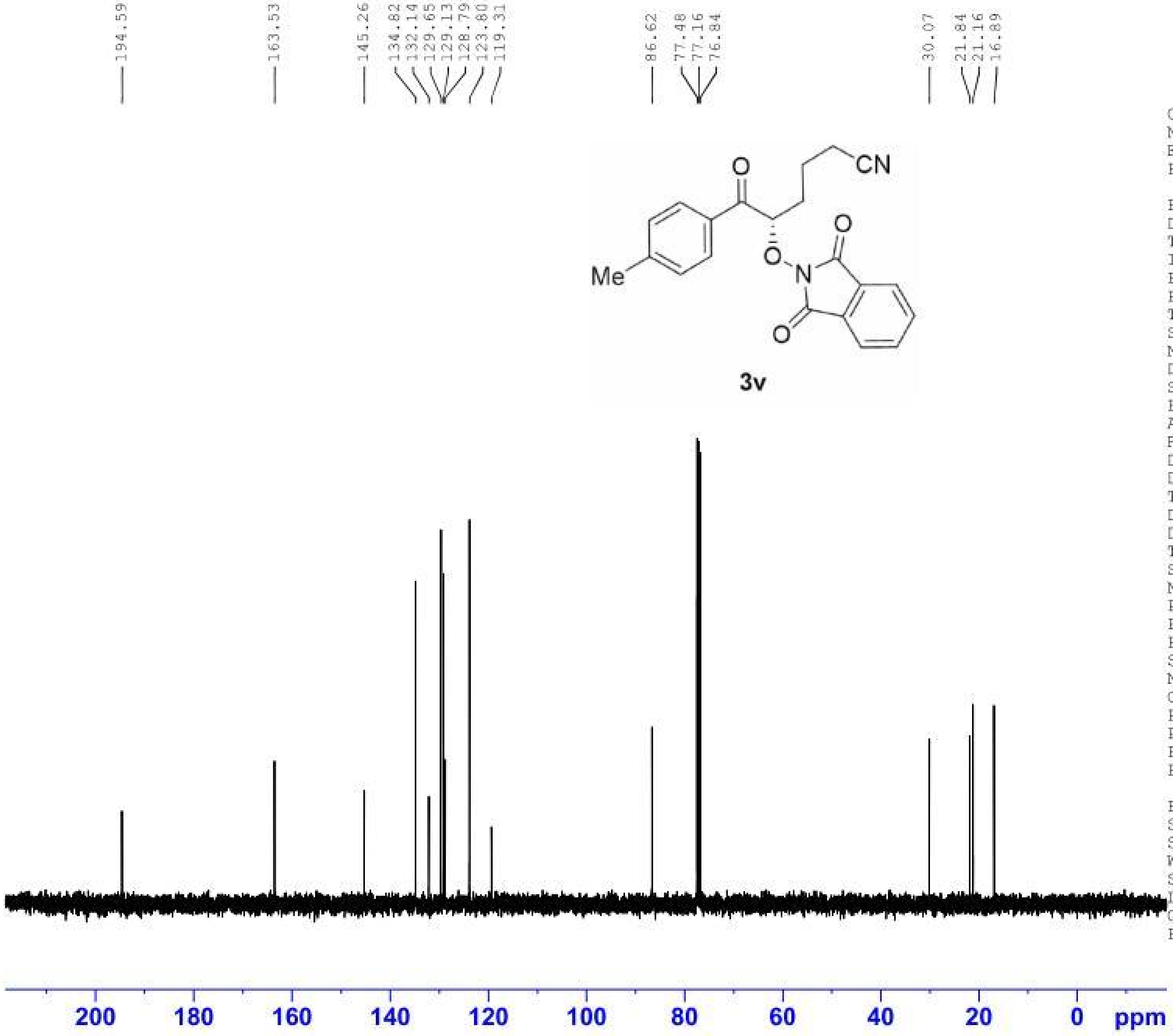

3v

Current Data Parameters
NAME qcx-6-117c
EXPNO 2
PROCNO 1

F2 - Acquisition Parameters
Date_ 20230429
Time 19.25 h
INSTRUM AvanceNeo 400MHz
PROBHD Z163739_0629 (
PULPROG zgpg30
TD 65536
SOLVENT CDCl3
NS 23
DS 4
SWH 23809.523 Hz
FIDRES 0.726609 Hz
AQ 1.3762560 sec
RG 10
DW 21.000 usec
DE 6.50 usec
TE 299.2 K
D1 2.00000000 sec
D11 0.03000000 sec
TD0 1
SFO1 100.6354036 MHz
NUC1 13C
P0 2.67 usec
P1 8.00 usec
PLW1 85.25399780 W
SFO2 400.1816007 MHz
NUC2 1H
CPDPRG[2 waltz65
PCPD2 90.00 usec
PLW2 21.26700020 W
PLW12 0.16802999 W
PLW13 0.08452000 W

F2 - Processing parameters
SI 32768
SF 100.6253339 MHz
WDW EM
SSB 0
LB 1.00 Hz
GB 0
PC 1.40

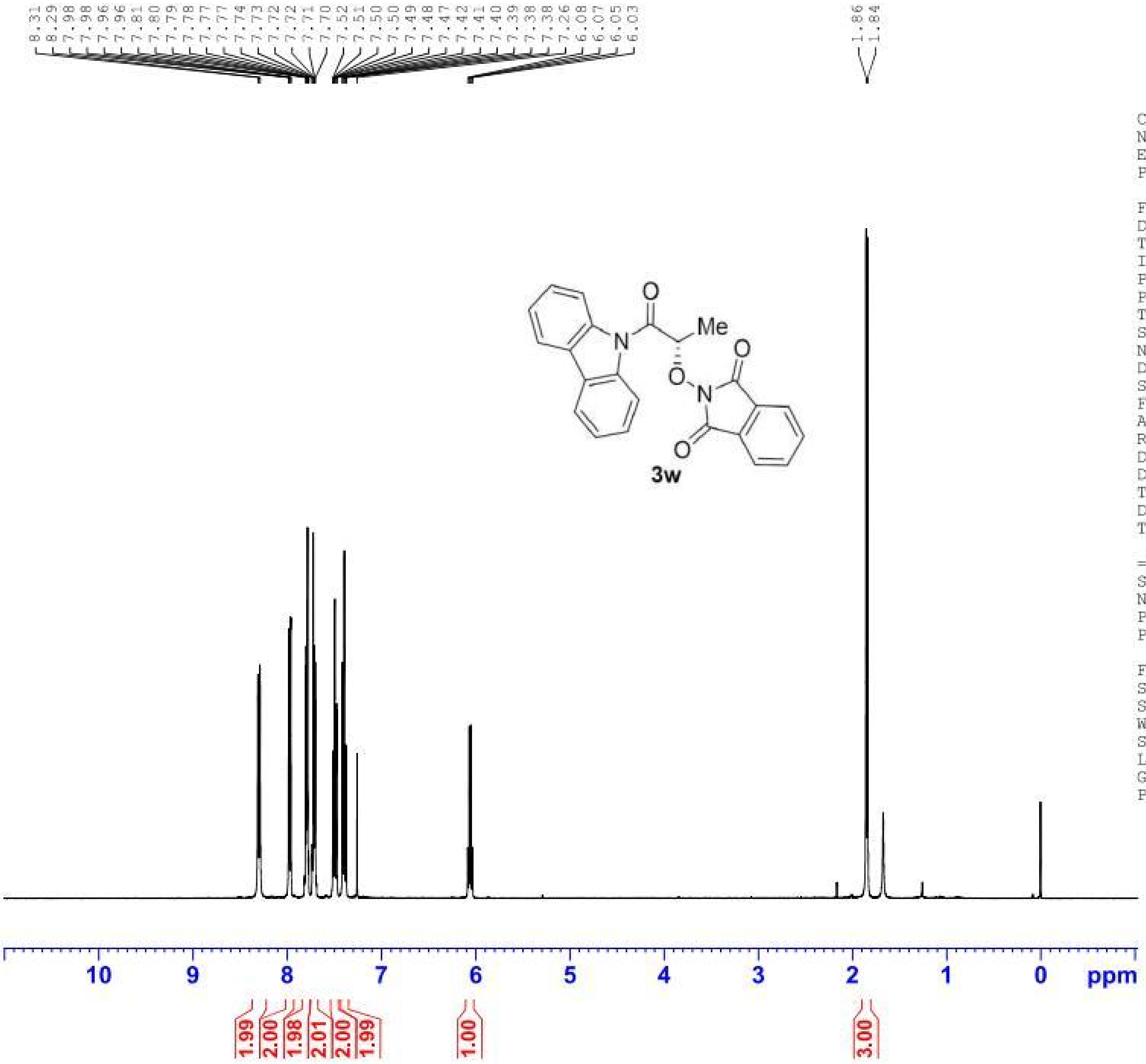

Current Data Parameters
NAME qcx-6-77-c
EXPNO 1
PROCNO 1

F2 - Acquisition Parameters
Date_ 20230325
Time 19.30
INSTRUM spect
PROBHD 5 mm PABBO BB/
PULPROG zg30
TD 65536
SOLVENT CDCl3
NS 2
DS 2
SWH 8012.820 Hz
FIDRES 0.122266 Hz
AQ 4.0894465 sec
RG 82.92
DW 62.400 usec
DE 6.50 usec
TE 296.4 K
D1 1.00000000 sec
TD0 1

======== CHANNEL f1 ========
SFO1 400.1324710 MHz
NUC1 1H
P1 14.50 usec
PLW1 11.99499989 W

F2 - Processing parameters
SI 65536
SF 400.1300101 MHz
WDW EM
SSB 0
LB 0.30 Hz
GB 0
PC 1.00

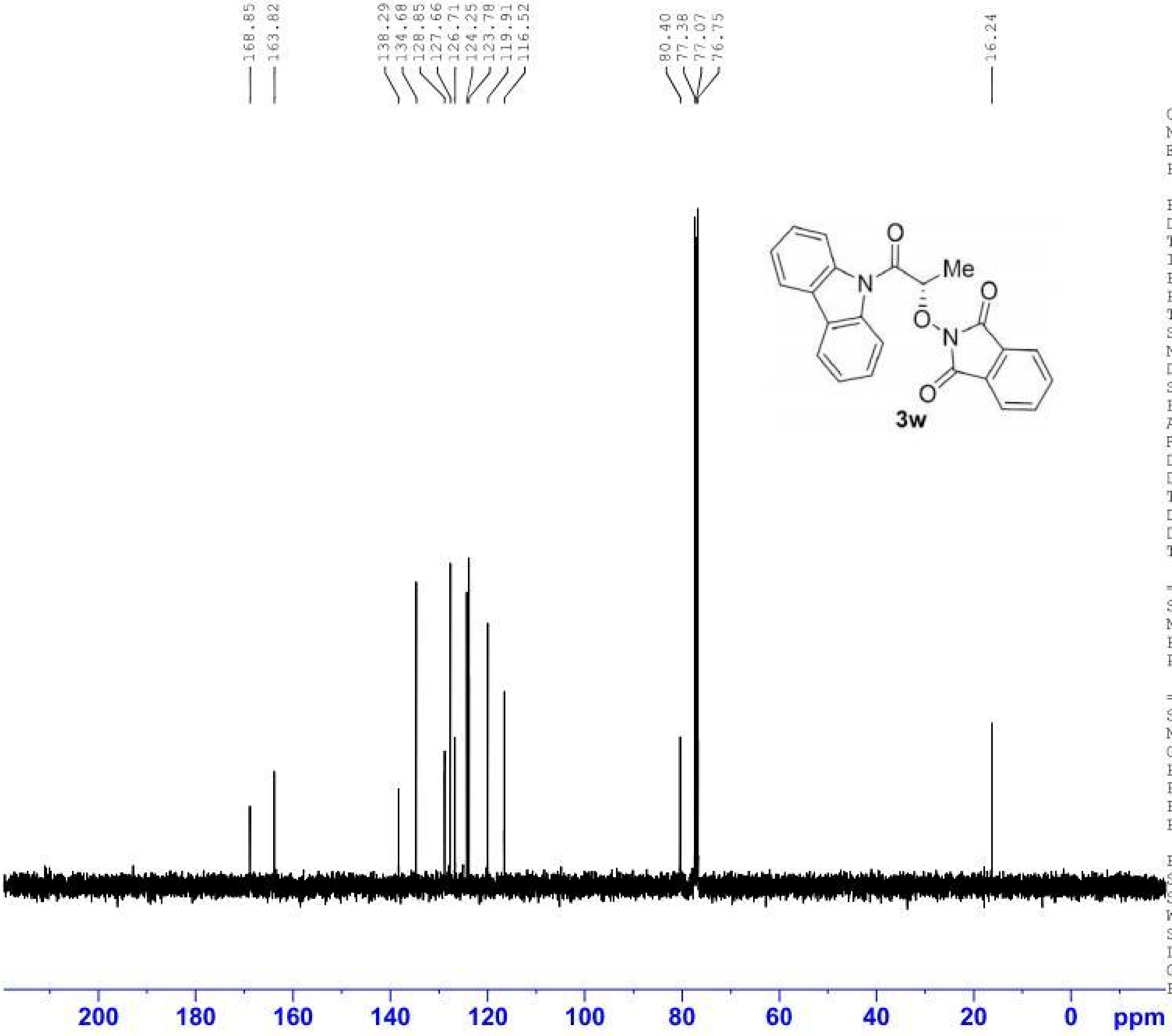


```
Current Data Parameters
NAME              qcx-6-77-c
EXPNO                      2
PROCNO                     1

F2 - Acquisition Parameters
Date_               20230325
Time                   19.33
INSTRUM                spect
PROBHD    5 mm PABBO BB/
PULPROG              zgpg30
TD                     65536
SOLVENT                CDCl3
NS                        30
DS                         2
SWH             24038.461 Hz
FIDRES           0.366798 Hz
AQ              1.3631488 sec
RG                    196.92
DW                  20.800 usec
DE                    6.50 usec
TE                    297.1 K
D1              2.00000000 sec
D11             0.03000000 sec
TD0                        1

======== CHANNEL f1 ========
SFO1         100.6228298 MHz
NUC1                     13C
P1                    9.70 usec
PLW1          46.98899841 W

======== CHANNEL f2 ========
SFO2         400.1316005 MHz
NUC2                      1H
CPDPRG[2             waltz16
PCPD2                90.00 usec
PLW2          11.99499989 W
PLW12          0.34213999 W
PLW13          0.27713001 W

F2 - Processing parameters
SI                     32768
SF           100.6127690 MHz
WDW                       EM
SSB                        0
LB                      1.00 Hz
GB                         0
PC                      1.40
```

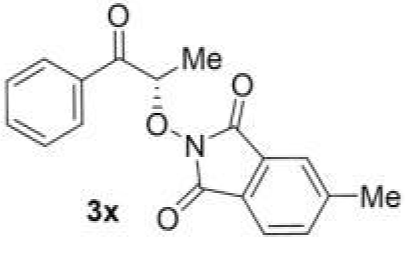

3x

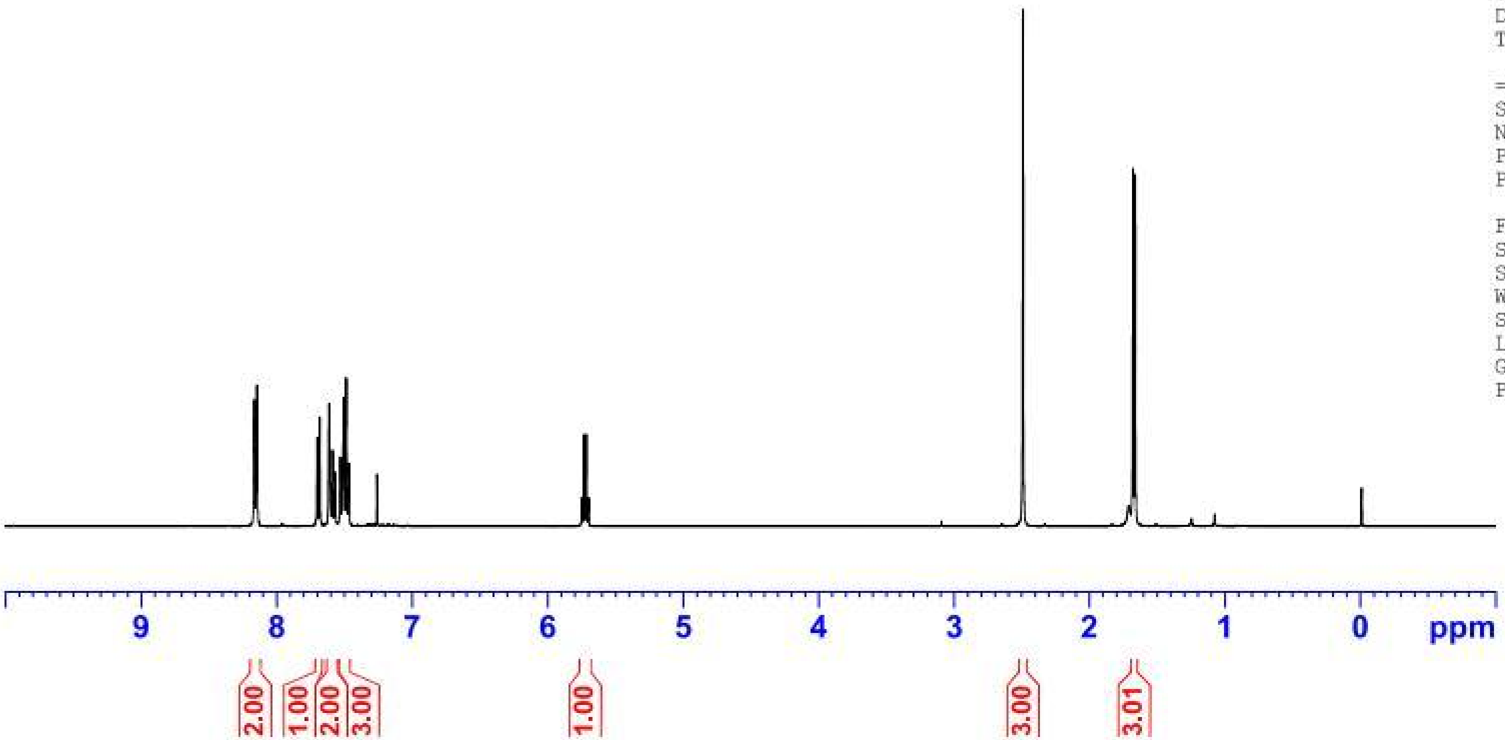


```
Current Data Parameters
NAME          qcx-6-131a
EXPNO                  1
PROCNO                 1

F2 - Acquisition Parameters
Date_           20230525
Time               19.57
INSTRUM            spect
PROBHD   5 mm PABBO BB/
PULPROG             zg30
TD                 65536
SOLVENT            CDCl3
NS                     3
DS                     2
SWH             8012.820 Hz
FIDRES          0.122266 Hz
AQ             4.0894465 sec
RG                 88.84
DW                62.400 usec
DE                  6.50 usec
TE                 296.0 K
D1            1.00000000 sec
TD0                    1

======== CHANNEL f1 ========
SFO1         400.1324710 MHz
NUC1                  1H
P1                 14.50 usec
PLW1         11.99499989 W

F2 - Processing parameters
SI                 65536
SF           400.1300101 MHz
WDW                   EM
SSB                    0
LB                  0.30 Hz
GB                     0
PC                  1.00
```

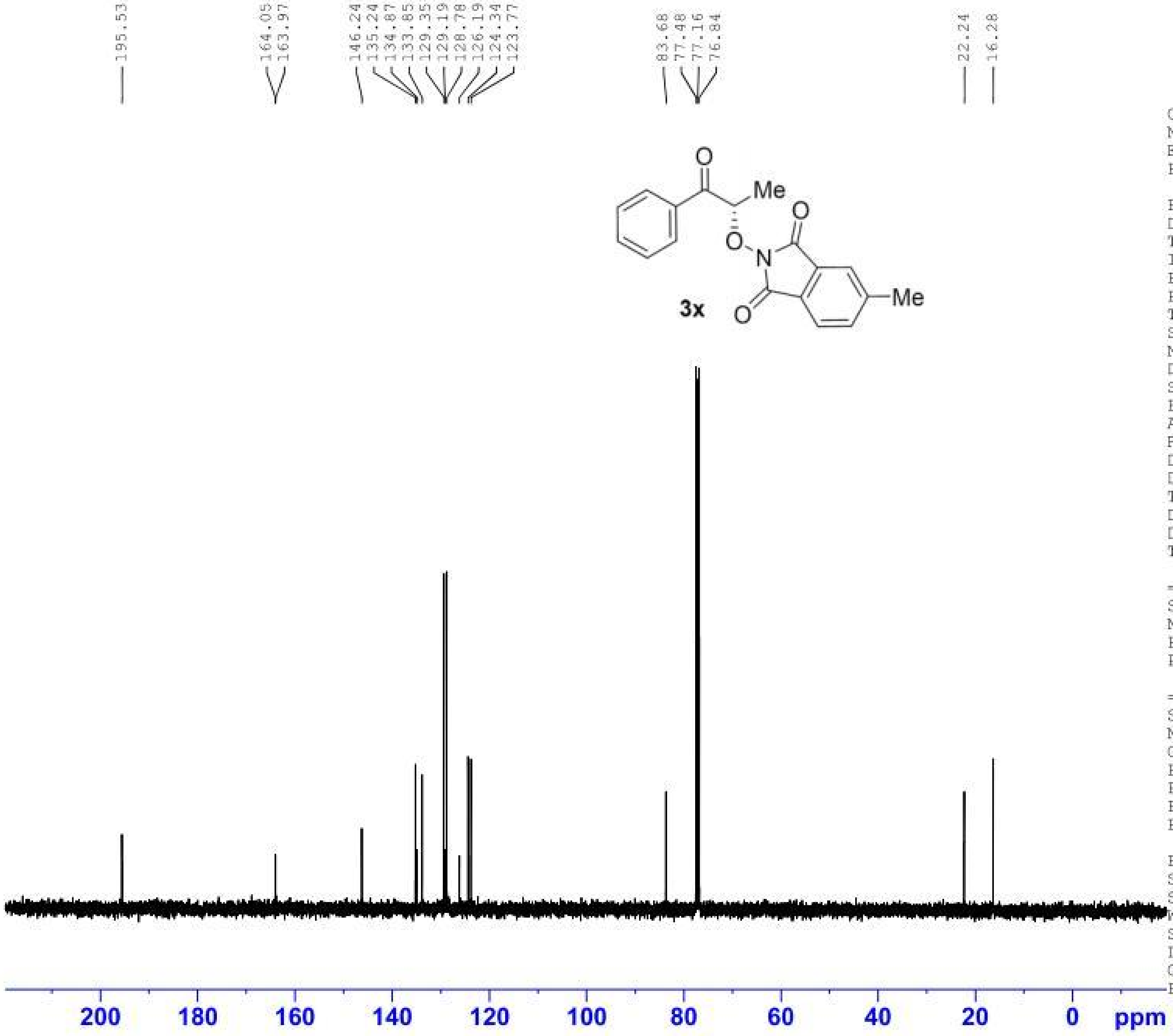


```
Current Data Parameters
NAME          qcx-6-131a
EXPNO                  2
PROCNO                 1

F2 - Acquisition Parameters
Date_           20230525
Time               19.59
INSTRUM            spect
PROBHD   5 mm PABBO BB/
PULPROG          zgpg30
TD                 65536
SOLVENT            CDCl3
NS                    58
DS                     2
SWH            24038.461 Hz
FIDRES          0.366798 Hz
AQ             1.3631488 sec
RG                196.92
DW                20.800 usec
DE                  6.50 usec
TE                 296.5 K
D1            2.00000000 sec
D11           0.03000000 sec
TD0                    1

======== CHANNEL f1 ========
SFO1         100.6228298 MHz
NUC1                 13C
P1                  9.70 usec
PLW1         46.98899841 W

======== CHANNEL f2 ========
SFO2         400.1316005 MHz
NUC2                  1H
CPDPRG[2         waltz16
PCPD2              90.00 usec
PLW2         11.99499989 W
PLW12         0.34213999 W
PLW13         0.27713001 W

F2 - Processing parameters
SI                 32768
SF           100.6127586 MHz
WDW                   EM
SSB                    0
LB                  1.00 Hz
GB                     0
PC                  1.40
```

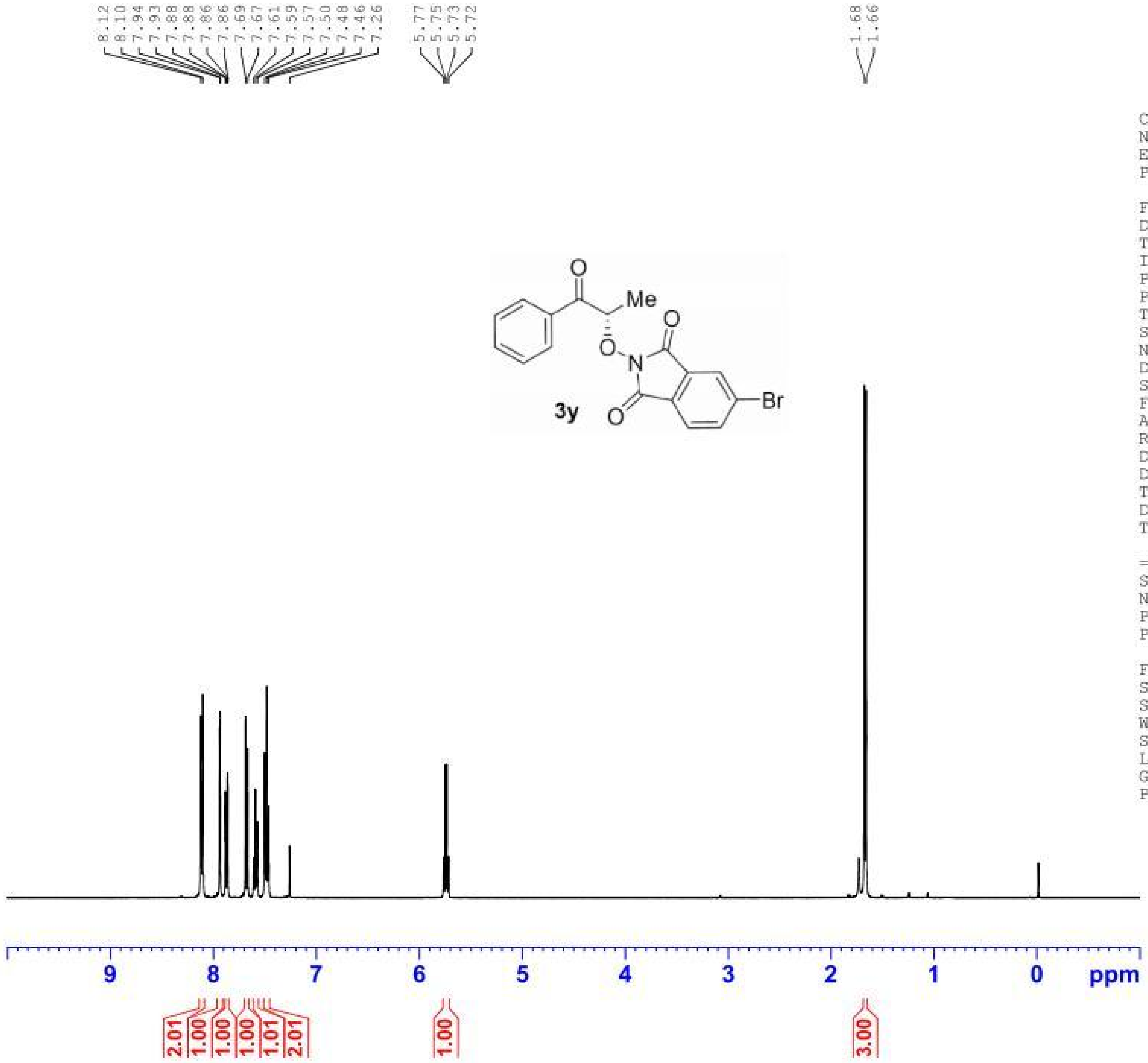

Current Data Parameters
NAME qcx-6-131b
EXPNO 1
PROCNO 1

F2 - Acquisition Parameters
Date_ 20230525
Time 20.05
INSTRUM spect
PROBHD 5 mm PABBO BB/
PULPROG zg30
TD 65536
SOLVENT CDCl3
NS 3
DS 2
SWH 8012.820 Hz
FIDRES 0.122266 Hz
AQ 4.0894465 sec
RG 70.97
DW 62.400 usec
DE 6.50 usec
TE 296.1 K
D1 1.00000000 sec
TD0 1

======== CHANNEL f1 ========
SFO1 400.1324710 MHz
NUC1 1H
P1 14.50 usec
PLW1 11.99499989 W

F2 - Processing parameters
SI 65536
SF 400.1300101 MHz
WDW EM
SSB 0
LB 0.30 Hz
GB 0
PC 1.00

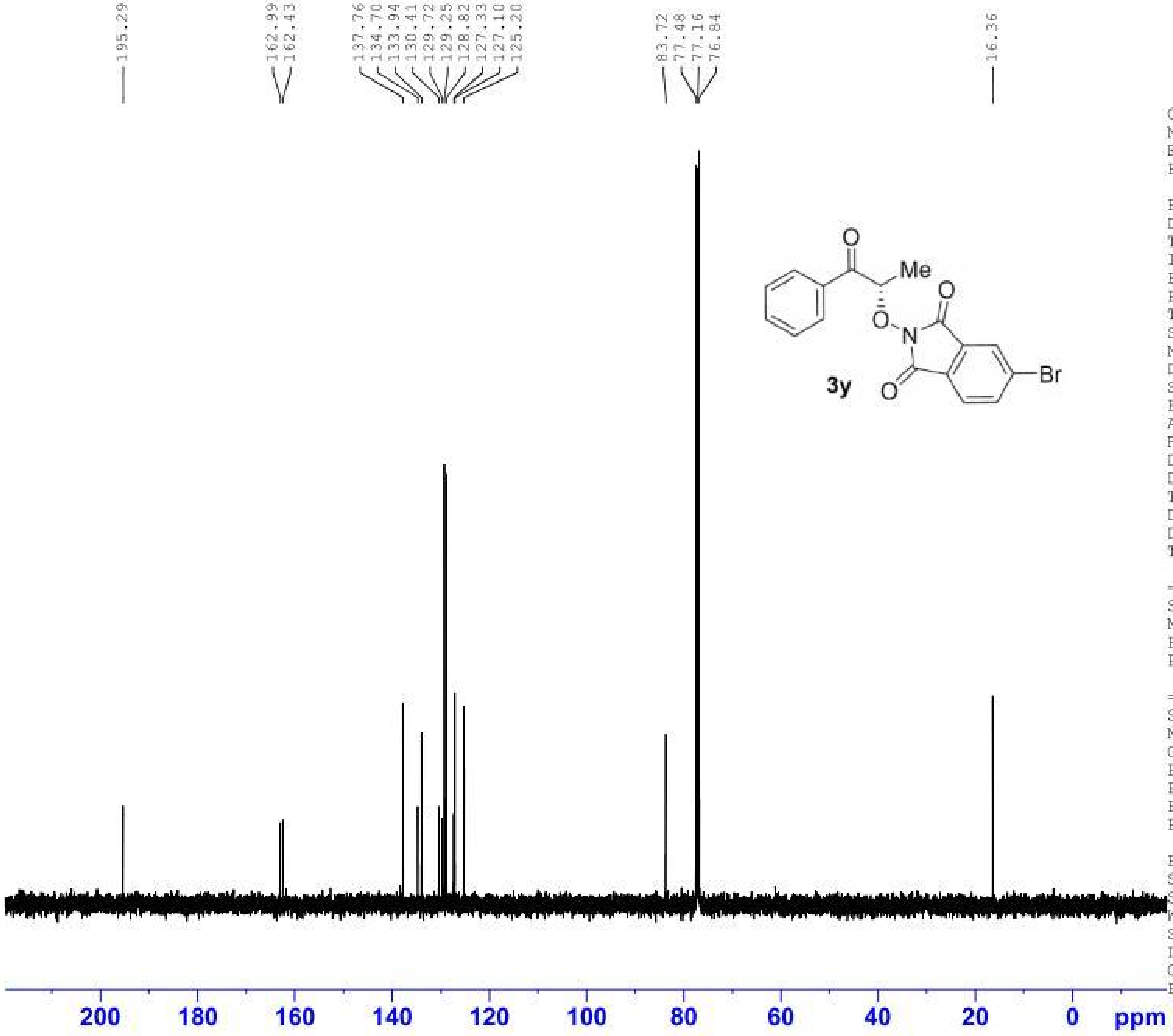


```
Current Data Parameters
NAME          qcx-6-131b
EXPNO                  2
PROCNO                 1

F2 - Acquisition Parameters
Date_           20230525
Time               20.07
INSTRUM            spect
PROBHD   5 mm PABBO BB/
PULPROG           zgpg30
TD                 65536
SOLVENT            CDCl3
NS                    57
DS                     2
SWH          24038.461 Hz
FIDRES        0.366798 Hz
AQ           1.3631488 sec
RG              196.92
DW              20.800 usec
DE                6.50 usec
TE               296.5 K
D1          2.00000000 sec
D11         0.03000000 sec
TD0                    1

======== CHANNEL f1 ========
SFO1       100.6228298 MHz
NUC1                 13C
P1                  9.70 usec
PLW1        46.98899841 W

======== CHANNEL f2 ========
SFO2       400.1316005 MHz
NUC2                  1H
CPDPRG[2         waltz16
PCPD2              90.00 usec
PLW2        11.99499989 W
PLW12        0.34213999 W
PLW13        0.27713001 W

F2 - Processing parameters
SI                 32768
SF          100.6127603 MHz
WDW                   EM
SSB                    0
LB                  1.00 Hz
GB                     0
PC                  1.40
```

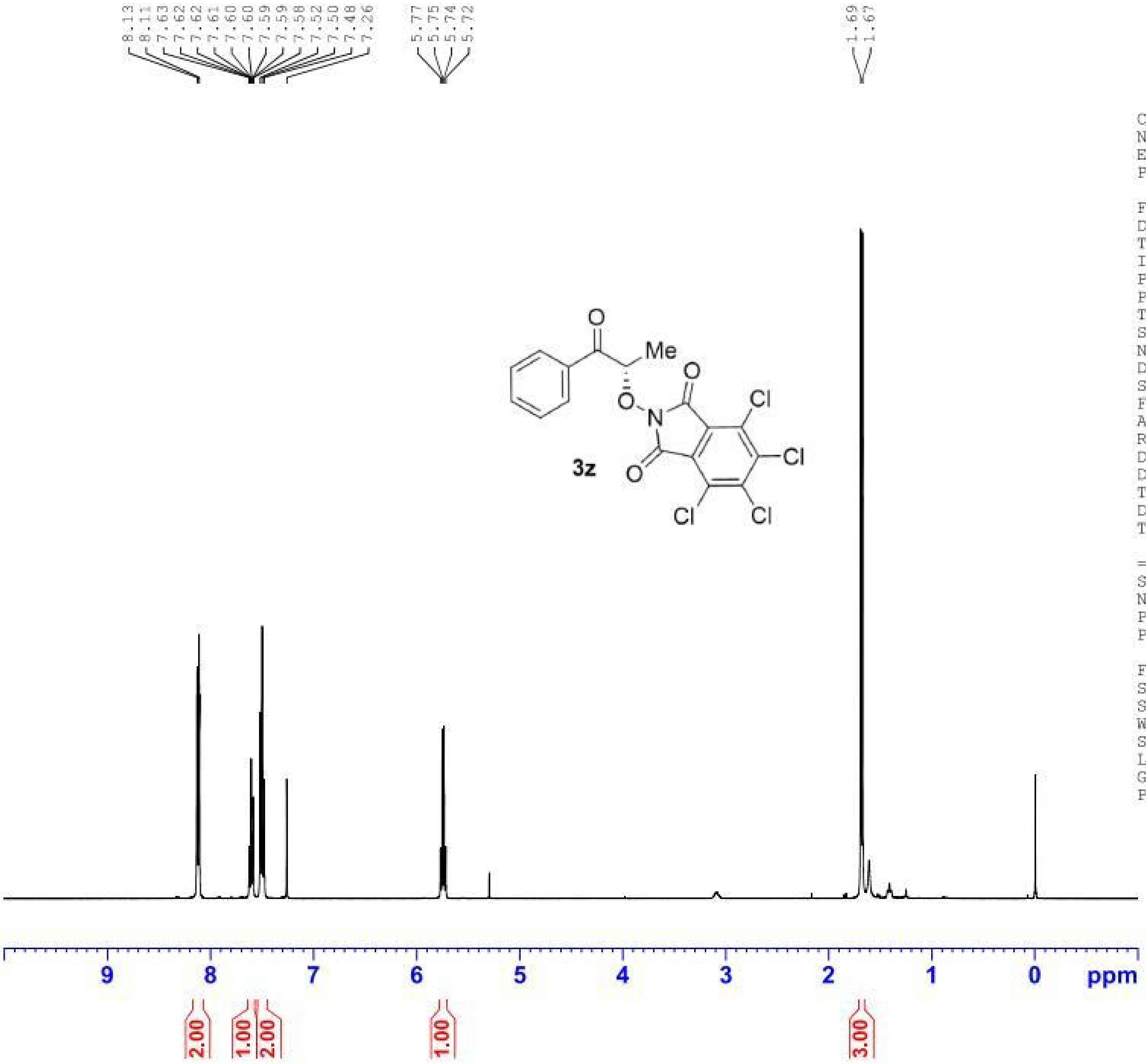

Current Data Parameters
NAME qcx-6-107a
EXPNO 1
PROCNO 1

F2 - Acquisition Parameters
Date_ 20230423
Time 18.14
INSTRUM spect
PROBHD 5 mm PABBO BB/
PULPROG zg30
TD 65536
SOLVENT CDCl3
NS 16
DS 2
SWH 8012.820 Hz
FIDRES 0.122266 Hz
AQ 4.0894465 sec
RG 126.97
DW 62.400 usec
DE 6.50 usec
TE 296.3 K
D1 1.00000000 sec
TD0 1

======== CHANNEL f1 ========
SFO1 400.1324710 MHz
NUC1 1H
P1 14.50 usec
PLW1 11.99499989 W

F2 - Processing parameters
SI 65536
SF 400.1300102 MHz
WDW EM
SSB 0
LB 0.30 Hz
GB 0
PC 1.00

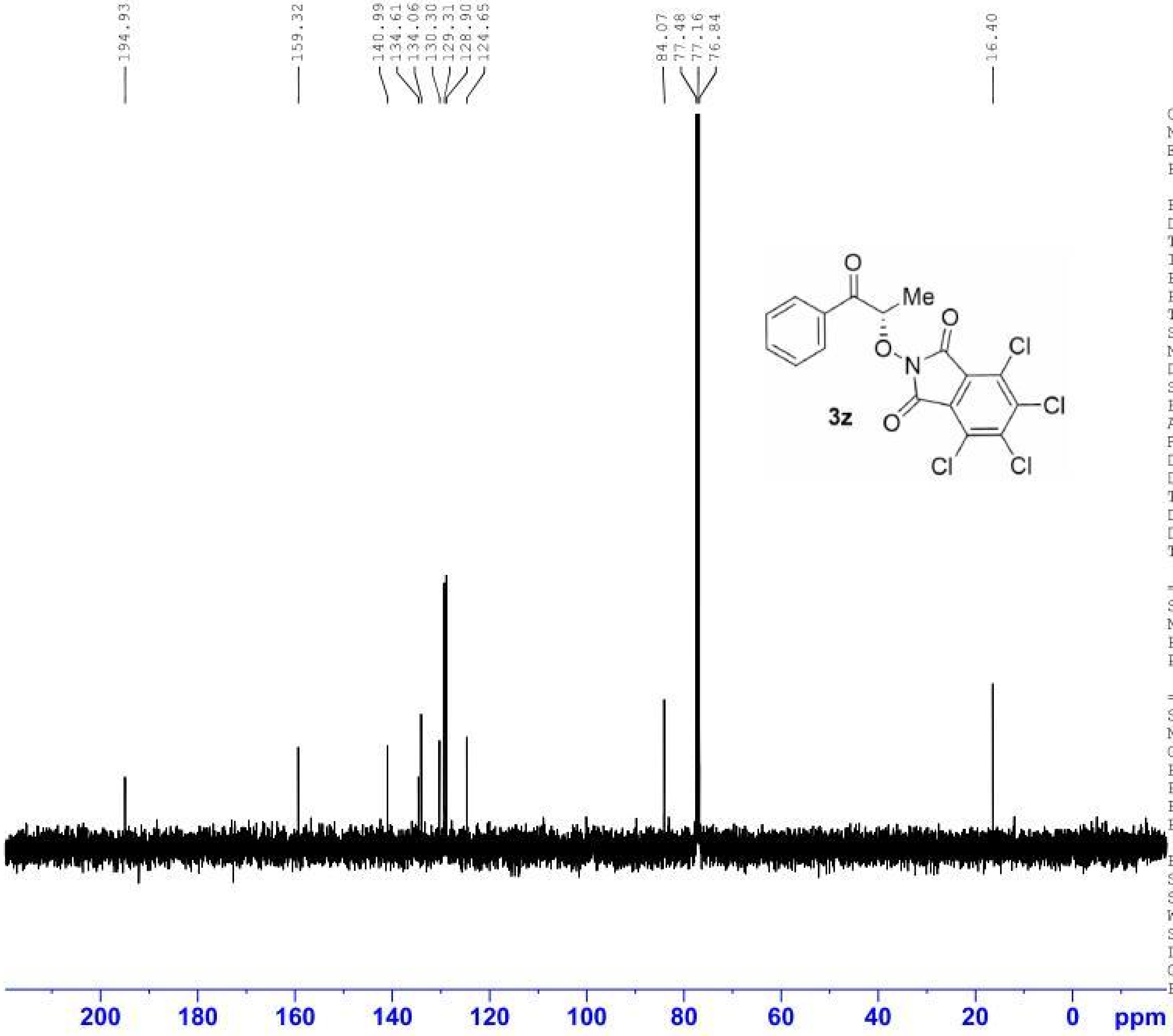

Current Data Parameters
NAME qcx-6-107a
EXPNO 2
PROCNO 1

F2 - Acquisition Parameters
Date_ 20230423
Time 18.19
INSTRUM spect
PROBHD 5 mm PABBO BB/
PULPROG zgpg30
TD 65536
SOLVENT CDCl3
NS 50
DS 2
SWH 24038.461 Hz
FIDRES 0.366798 Hz
AQ 1.3631488 sec
RG 196.92
DW 20.800 usec
DE 6.50 usec
TE 296.5 K
D1 2.00000000 sec
D11 0.03000000 sec
TD0 1

======== CHANNEL f1 ========
SFO1 100.6228298 MHz
NUC1 13C
P1 9.70 usec
PLW1 46.98899841 W

======== CHANNEL f2 ========
SFO2 400.1316005 MHz
NUC2 1H
CPDPRG[2 waltz16
PCPD2 90.00 usec
PLW2 11.99499989 W
PLW12 0.34213999 W
PLW13 0.27713001 W

F2 - Processing parameters
SI 32768
SF 100.6127563 MHz
WDW EM
SSB 0
LB 1.00 Hz
GB 0
PC 1.40

Current Data Parameters
NAME qcx-6-59a-new
EXPNO 1
PROCNO 1

F2 - Acquisition Parameters
Date_ 20230527
Time 21.22
INSTRUM spect
PROBHD 5 mm PABBO BB/
PULPROG zg30
TD 65536
SOLVENT CDCl3
NS 3
DS 2
SWH 8012.820 Hz
FIDRES 0.122266 Hz
AQ 4.0894465 sec
RG 62.93
DW 62.400 usec
DE 6.50 usec
TE 296.6 K
D1 1.00000000 sec
TD0 1

======== CHANNEL f1 ========
SFO1 400.1324710 MHz
NUC1 1H
P1 14.50 usec
PLW1 11.99499989 W

F2 - Processing parameters
SI 65536
SF 400.1300102 MHz
WDW EM
SSB 0
LB 0.30 Hz
GB 0
PC 1.00

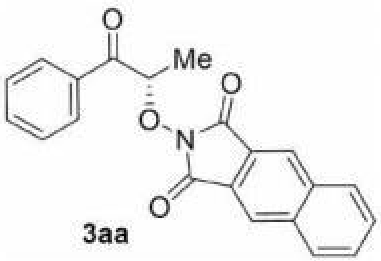

3aa

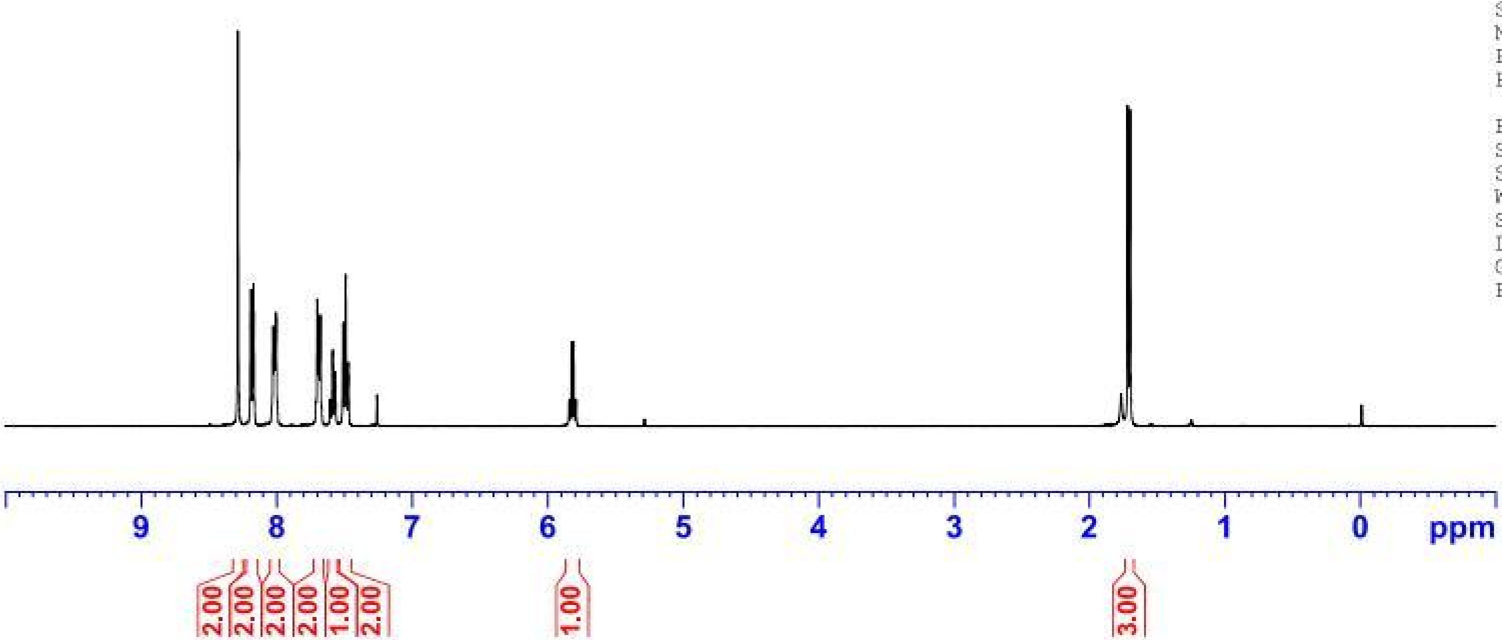

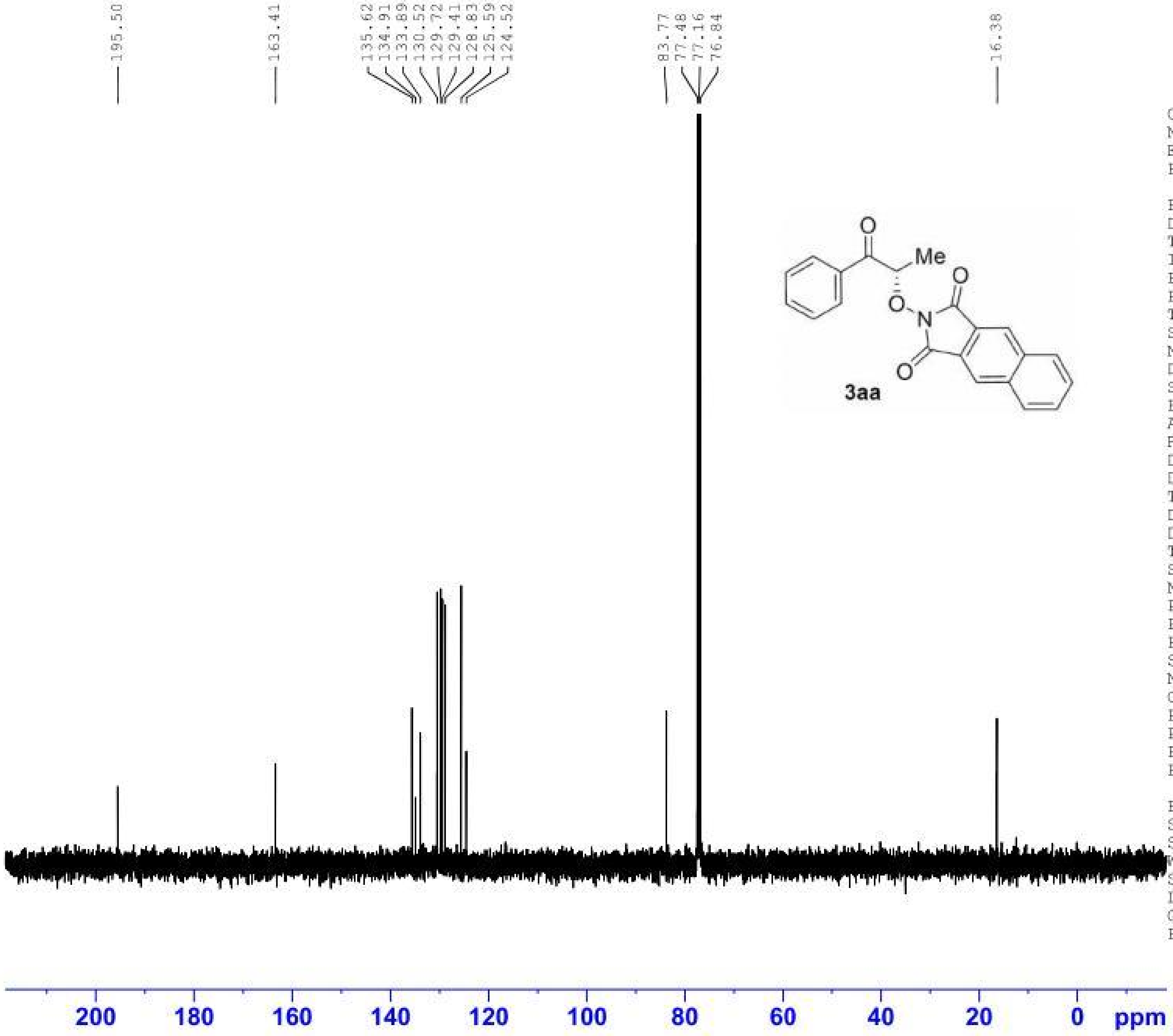

3aa

Current Data Parameters
NAME qcx-6-59a
EXPNO 2
PROCNO 1

F2 - Acquisition Parameters
Date_ 20230318
Time 19.27 h
INSTRUM AvanceNeo 400MHz
PROBHD Z163739_0629 (
PULPROG zgpg30
TD 65536
SOLVENT CDCl3
NS 79
DS 4
SWH 23809.523 Hz
FIDRES 0.726609 Hz
AQ 1.3762560 sec
RG 11.3
DW 21.000 usec
DE 6.50 usec
TE 297.2 K
D1 2.00000000 sec
D11 0.03000000 sec
TD0 1
SFO1 100.6354036 MHz
NUC1 13C
P0 2.67 usec
P1 8.00 usec
PLW1 85.25399780 W
SFO2 400.1816007 MHz
NUC2 1H
CPDPRG[2 waltz65
PCPD2 90.00 usec
PLW2 21.26700020 W
PLW12 0.16802999 W
PLW13 0.08452000 W

F2 - Processing parameters
SI 32768
SF 100.6253285 MHz
WDW EM
SSB 0
LB 1.00 Hz
GB 0
PC 1.40

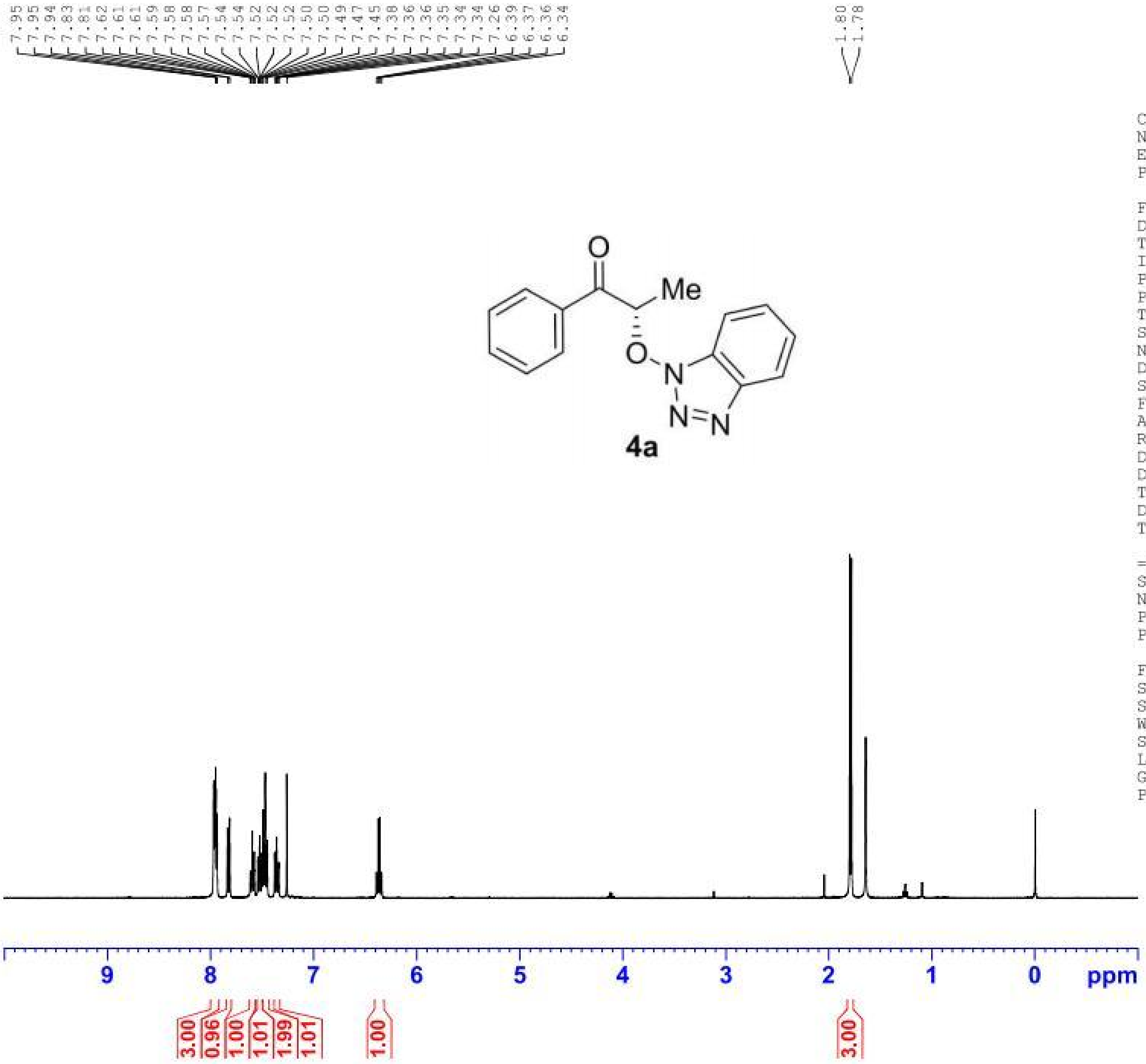


```
Current Data Parameters
NAME          qcx-6-59b-new
EXPNO                     1
PROCNO                    1

F2 - Acquisition Parameters
Date_              20230317
Time                  19.34
INSTRUM               spect
PROBHD   5 mm PABBO BB/
PULPROG                zg30
TD                    65536
SOLVENT               CDCl3
NS                        3
DS                        2
SWH               8012.820 Hz
FIDRES            0.122266 Hz
AQ               4.0894465 sec
RG                   164.33
DW                   62.400 usec
DE                     6.50 usec
TE                    296.0 K
D1               1.00000000 sec
TD0                       1

======== CHANNEL f1 ========
SFO1            400.1324710 MHz
NUC1                     1H
P1                    14.50 usec
PLW1            11.99499989 W

F2 - Processing parameters
SI                    65536
SF              400.1300101 MHz
WDW                      EM
SSB                       0
LB                     0.30 Hz
GB                        0
PC                     1.00
```

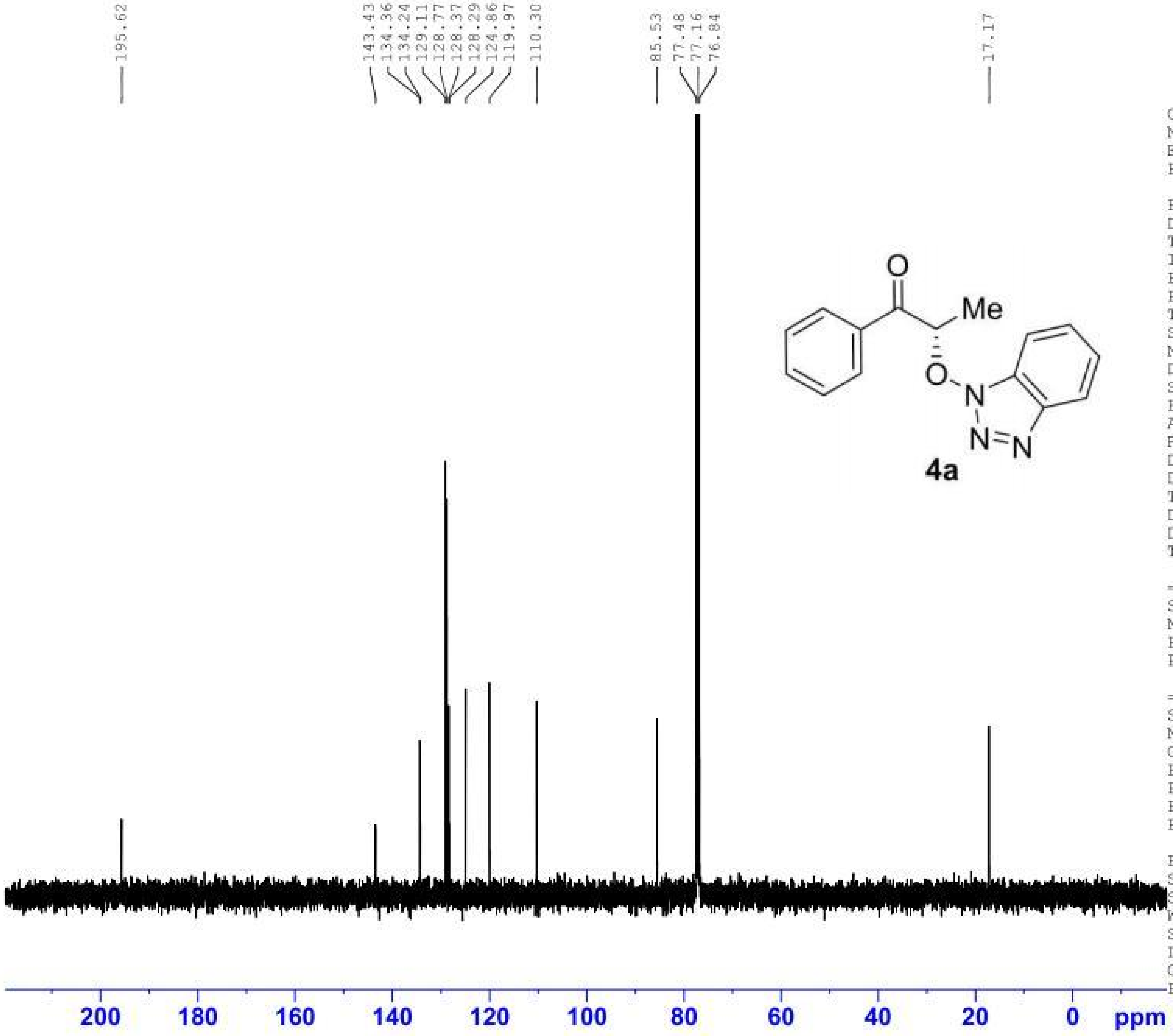


```
Current Data Parameters
NAME       qcx-6-59b-new
EXPNO                  2
PROCNO                 1

F2 - Acquisition Parameters
Date_           20230317
Time               19.37
INSTRUM            spect
PROBHD   5 mm PABBO BB/
PULPROG          zgpg30
TD                 65536
SOLVENT            CDCl3
NS                   134
DS                     2
SWH            24038.461 Hz
FIDRES          0.366798 Hz
AQ             1.3631488 sec
RG                196.92
DW                20.800 usec
DE                  6.50 usec
TE                 296.6 K
D1            2.00000000 sec
D11           0.03000000 sec
TD0                    1

======== CHANNEL f1 ========
SFO1         100.6228298 MHz
NUC1                 13C
P1                  9.70 usec
PLW1         46.98899841 W

======== CHANNEL f2 ========
SFO2         400.1316005 MHz
NUC2                  1H
CPDPRG[2          waltz16
PCPD2              90.00 usec
PLW2         11.99499989 W
PLW12         0.34213999 W
PLW13         0.27713001 W

F2 - Processing parameters
SI                 32768
SF           100.6127561 MHz
WDW                   EM
SSB                    0
LB                  1.00 Hz
GB                     0
PC                  1.40
```

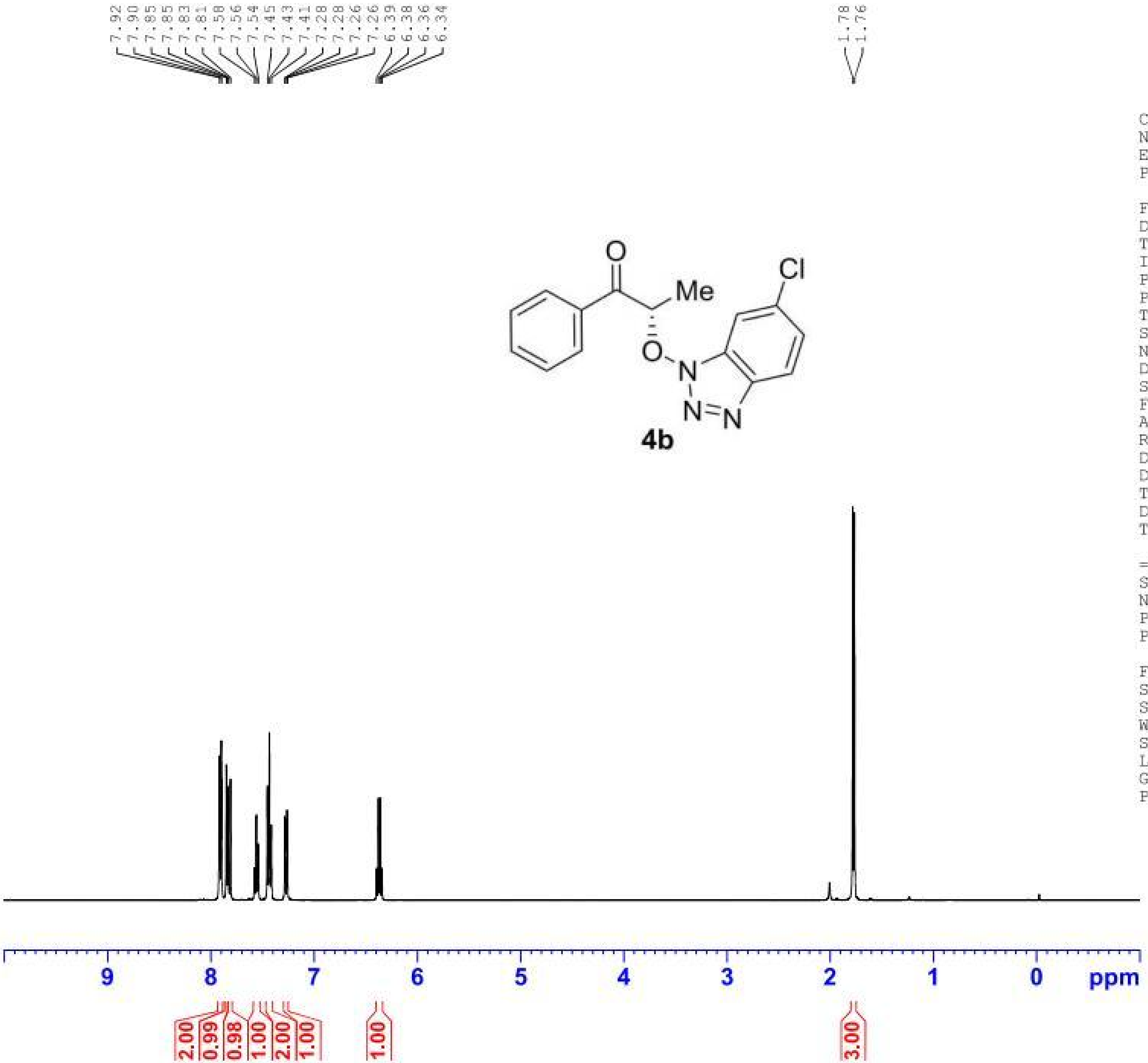

4b

Current Data Parameters
NAME qcx-6-136a
EXPNO 1
PROCNO 1

F2 - Acquisition Parameters
Date_ 20230530
Time 19.26
INSTRUM spect
PROBHD 5 mm PABBO BB/
PULPROG zg30
TD 65536
SOLVENT CDCl3
NS 3
DS 2
SWH 8012.820 Hz
FIDRES 0.122266 Hz
AQ 4.0894465 sec
RG 39.46
DW 62.400 usec
DE 6.50 usec
TE 296.2 K
D1 1.00000000 sec
TD0 1

======== CHANNEL f1 ========
SFO1 400.1324710 MHz
NUC1 1H
P1 14.50 usec
PLW1 11.99499989 W

F2 - Processing parameters
SI 65536
SF 400.1300092 MHz
WDW EM
SSB 0
LB 0.30 Hz
GB 0
PC 1.00

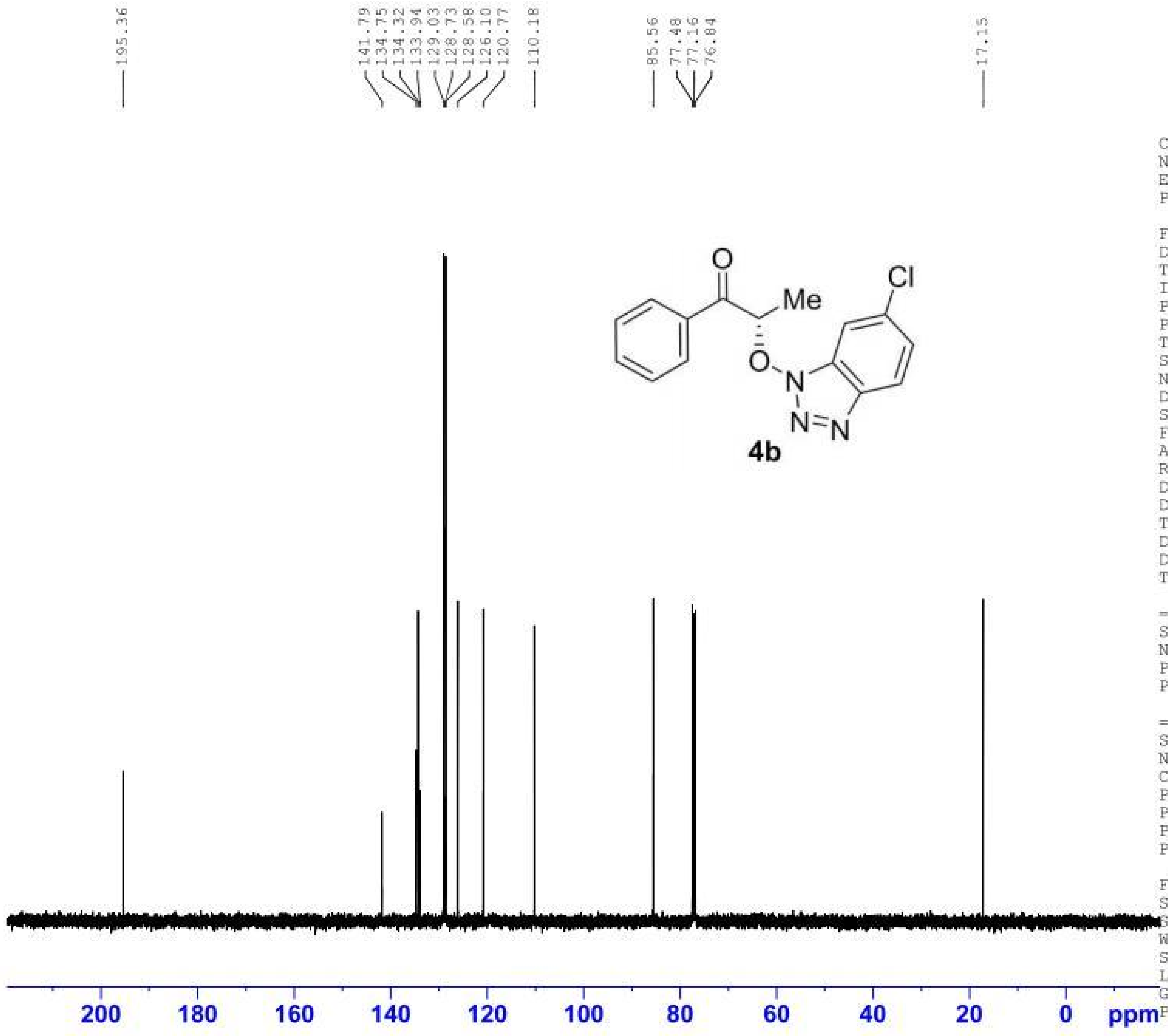

Current Data Parameters
NAME qcx-6-136a
EXPNO 2
PROCNO 1

F2 - Acquisition Parameters
Date_ 20230530
Time 19.28
INSTRUM spect
PROBHD 5 mm PABBO BB/
PULPROG zgpg30
TD 65536
SOLVENT CDCl3
NS 17
DS 2
SWH 24038.461 Hz
FIDRES 0.366798 Hz
AQ 1.3631488 sec
RG 196.92
DW 20.800 usec
DE 6.50 usec
TE 296.8 K
D1 2.00000000 sec
D11 0.03000000 sec
TD0 1

======== CHANNEL f1 ========
SFO1 100.6228298 MHz
NUC1 13C
P1 9.70 usec
PLW1 46.98899841 W

======== CHANNEL f2 ========
SFO2 400.1316005 MHz
NUC2 1H
CPDPRG[2 waltz16
PCPD2 90.00 usec
PLW2 11.99499989 W
PLW12 0.34213999 W
PLW13 0.27713001 W

F2 - Processing parameters
SI 32768
SF 100.6127666 MHz
WDW EM
SSB 0
LB 1.00 Hz
GB 0
PC 1.40

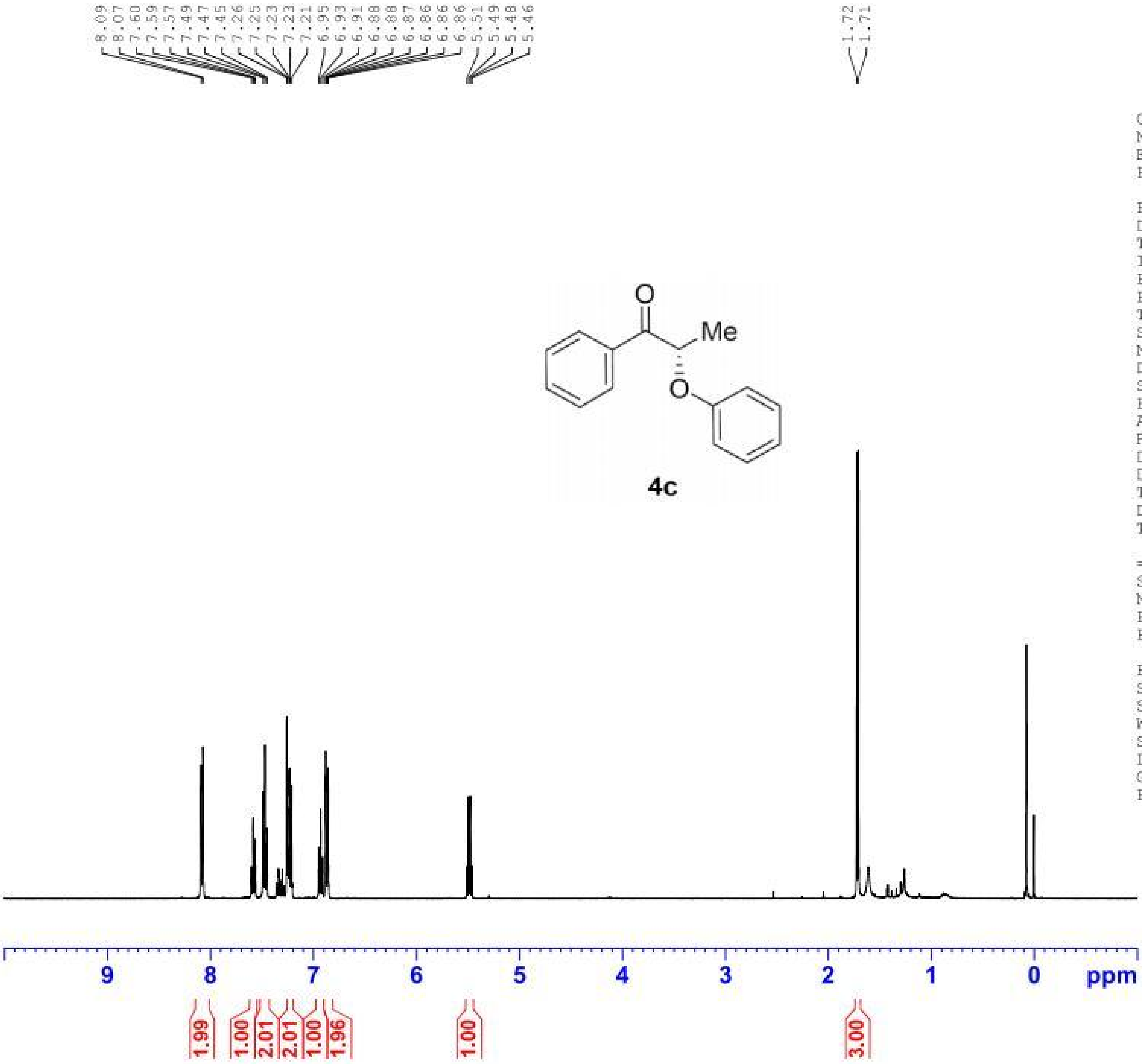

4c

Current Data Parameters
NAME qcx-phenol
EXPNO 1
PROCNO 1

F2 - Acquisition Parameters
Date_ 20250723
Time 22.46
INSTRUM spect
PROBHD 5 mm PABBO BB/
PULPROG zg30
TD 65536
SOLVENT CDCl3
NS 64
DS 2
SWH 8012.820 Hz
FIDRES 0.122266 Hz
AQ 4.0894465 sec
RG 112.31
DW 62.400 usec
DE 6.50 usec
TE 296.8 K
D1 1.00000000 sec
TD0 1

======== CHANNEL f1 ========
SFO1 400.1324710 MHz
NUC1 1H
P1 14.50 usec
PLW1 11.99499989 W

F2 - Processing parameters
SI 65536
SF 400.1300098 MHz
WDW EM
SSB 0
LB 0.30 Hz
GB 0
PC 1.00

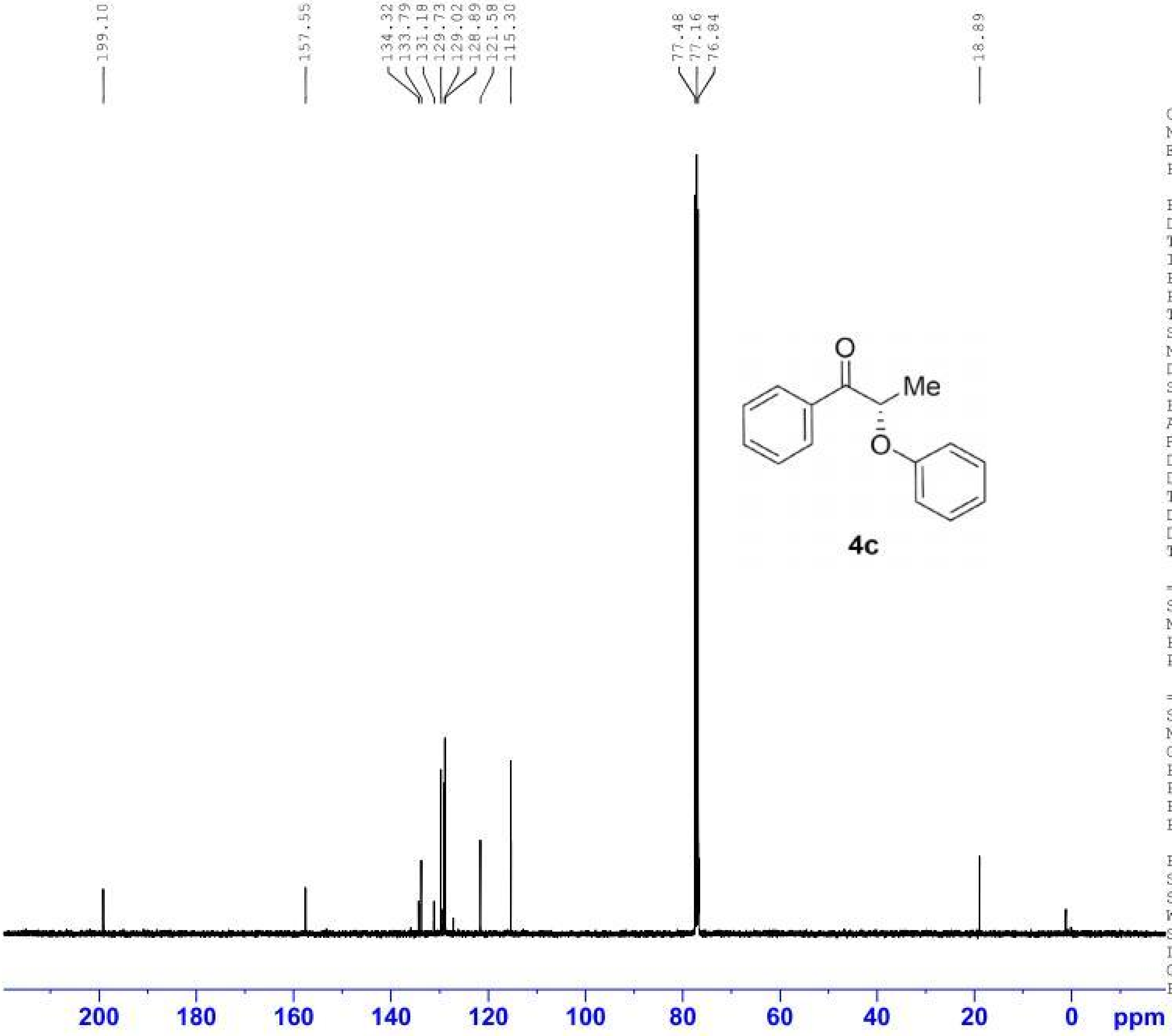

4c

```
Current Data Parameters
NAME          qcx-phenol
EXPNO                  2
PROCNO                 1

F2 - Acquisition Parameters
Date_           20250723
Time               23.48
INSTRUM            spect
PROBHD   5 mm PABBO BB/
PULPROG          zgpg30
TD                 65536
SOLVENT            CDCl3
NS                   989
DS                     2
SWH            24038.461 Hz
FIDRES          0.366798 Hz
AQ             1.3631488 sec
RG                196.92
DW                20.800 usec
DE                  6.50 usec
TE                 298.2 K
D1            2.00000000 sec
D11           0.03000000 sec
TD0                    1

======== CHANNEL f1 ========
SFO1         100.6228298 MHz
NUC1                 13C
P1                  9.70 usec
PLW1         46.98899841 W

======== CHANNEL f2 ========
SFO2         400.1316005 MHz
NUC2                  1H
CPDPRG[2         waltz16
PCPD2              90.00 usec
PLW2         11.99499989 W
PLW12         0.34213999 W
PLW13         0.27713001 W

F2 - Processing parameters
SI                 32768
SF           100.6127556 MHz
WDW                   EM
SSB                    0
LB                  1.00 Hz
GB                     0
PC                  1.40
```

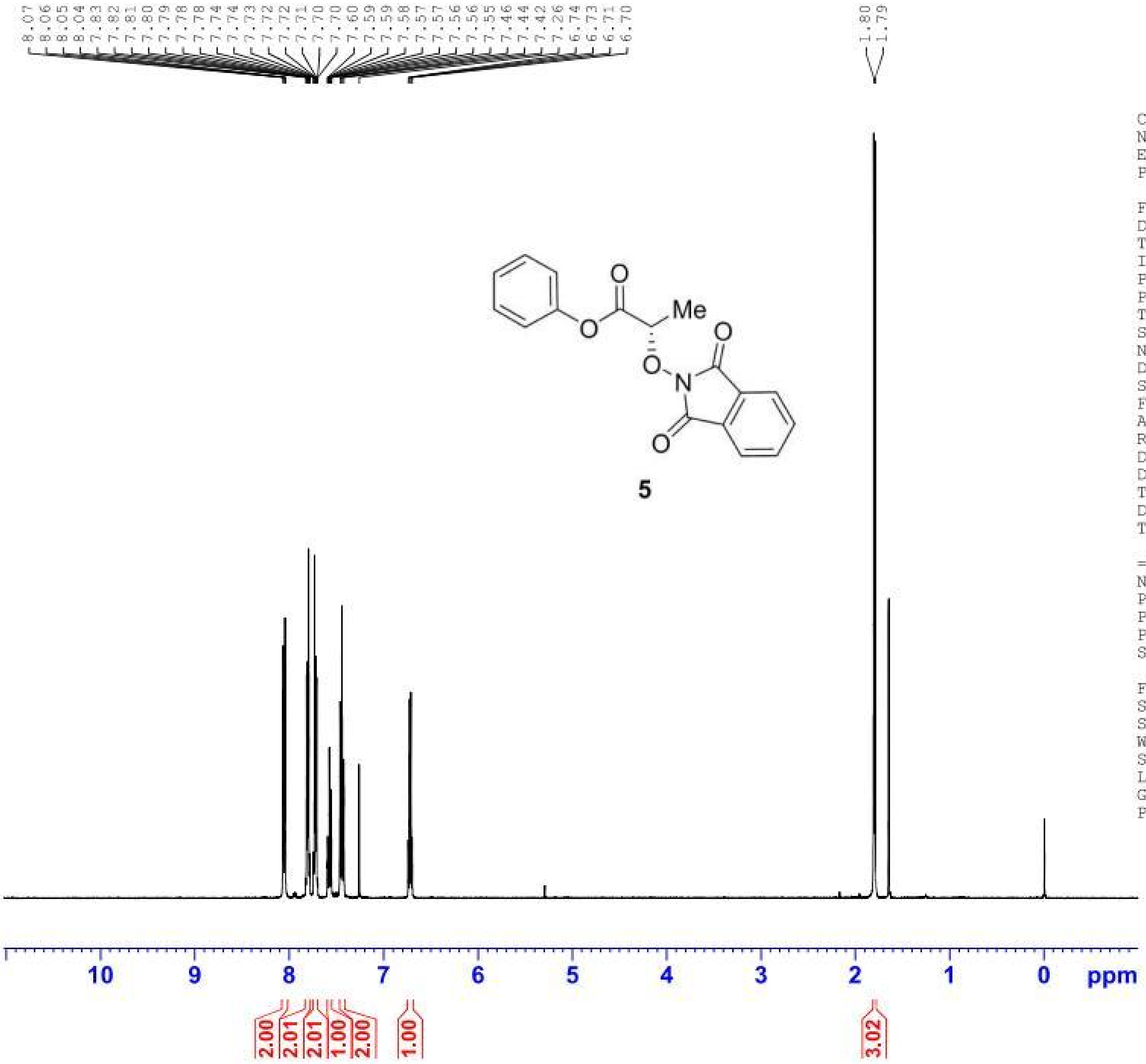

Current Data Parameters
NAME qcx-6-76b-new
EXPNO 1
PROCNO 1

F2 - Acquisition Parameters
Date_ 20230324
Time 21.44
INSTRUM spect
PROBHD 5 mm DUL 13C-1
PULPROG zg30
TD 65536
SOLVENT CDCl3
NS 4
DS 2
SWH 8223.685 Hz
FIDRES 0.125483 Hz
AQ 3.9845889 sec
RG 362
DW 60.800 usec
DE 6.00 usec
TE 293.3 K
D1 1.00000000 sec
TD0 1

======== CHANNEL f1 ========
NUC1 1H
P1 15.80 usec
PL1 -1.00 dB
PL1W 12.17476940 W
SFO1 400.1324710 MHz

F2 - Processing parameters
SI 32768
SF 400.1300098 MHz
WDW EM
SSB 0
LB 0.30 Hz
GB 0
PC 1.00

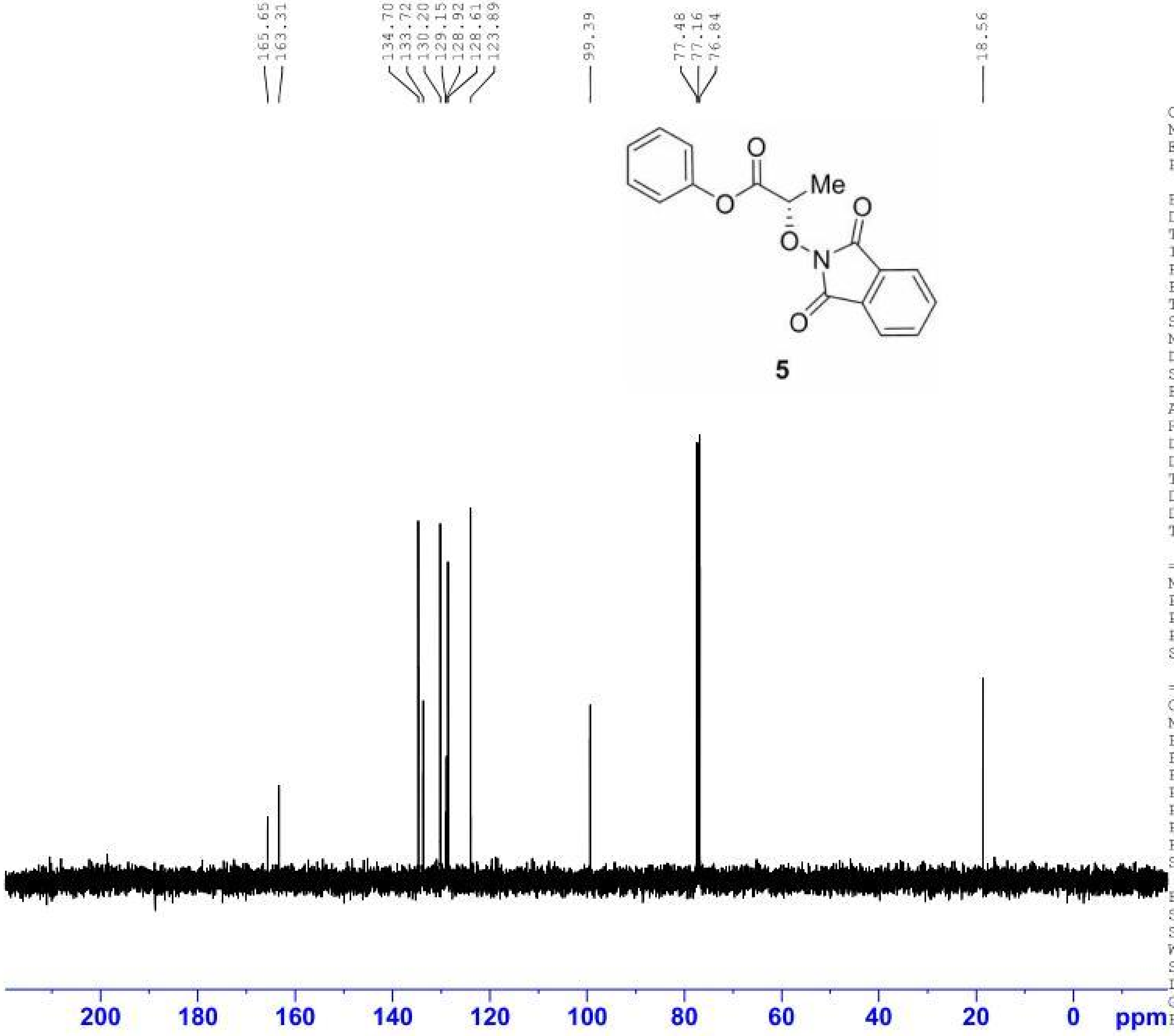


```
Current Data Parameters
NAME          qcx-6-76b-new
EXPNO                     2
PROCNO                    1

F2 - Acquisition Parameters
Date_              20230324
Time                  21.45
INSTRUM               spect
PROBHD     5 mm DUL 13C-1
PULPROG             zgpg30
TD                   65536
SOLVENT              CDCl3
NS                      76
DS                       1
SWH              24038.461 Hz
FIDRES            0.366798 Hz
AQ               1.3631488 sec
RG                    2050
DW                  20.800 usec
DE                    6.00 usec
TE                   293.4 K
D1              2.00000000 sec
D11             0.03000000 sec
TD0                      1

======== CHANNEL f1 ========
NUC1                   13C
P1                   40.00 usec
PL1                  -3.00 dB
PL1W           60.64365387 W
SFO1           100.6228298 MHz

======== CHANNEL f2 ========
CPDPRG[2           waltz16
NUC2                    1H
PCPD2                80.00 usec
PL2                  -1.00 dB
PL12                 14.39 dB
PL13                 18.00 dB
PL2W           12.17476940 W
PL12W           0.35193357 W
PL13W           0.15327126 W
SFO2           400.1316005 MHz

F2 - Processing parameters
SI                   32768
SF             100.6127582 MHz
WDW                     EM
SSB                      0
LB                    1.00 Hz
GB                       0
PC                    1.40
```

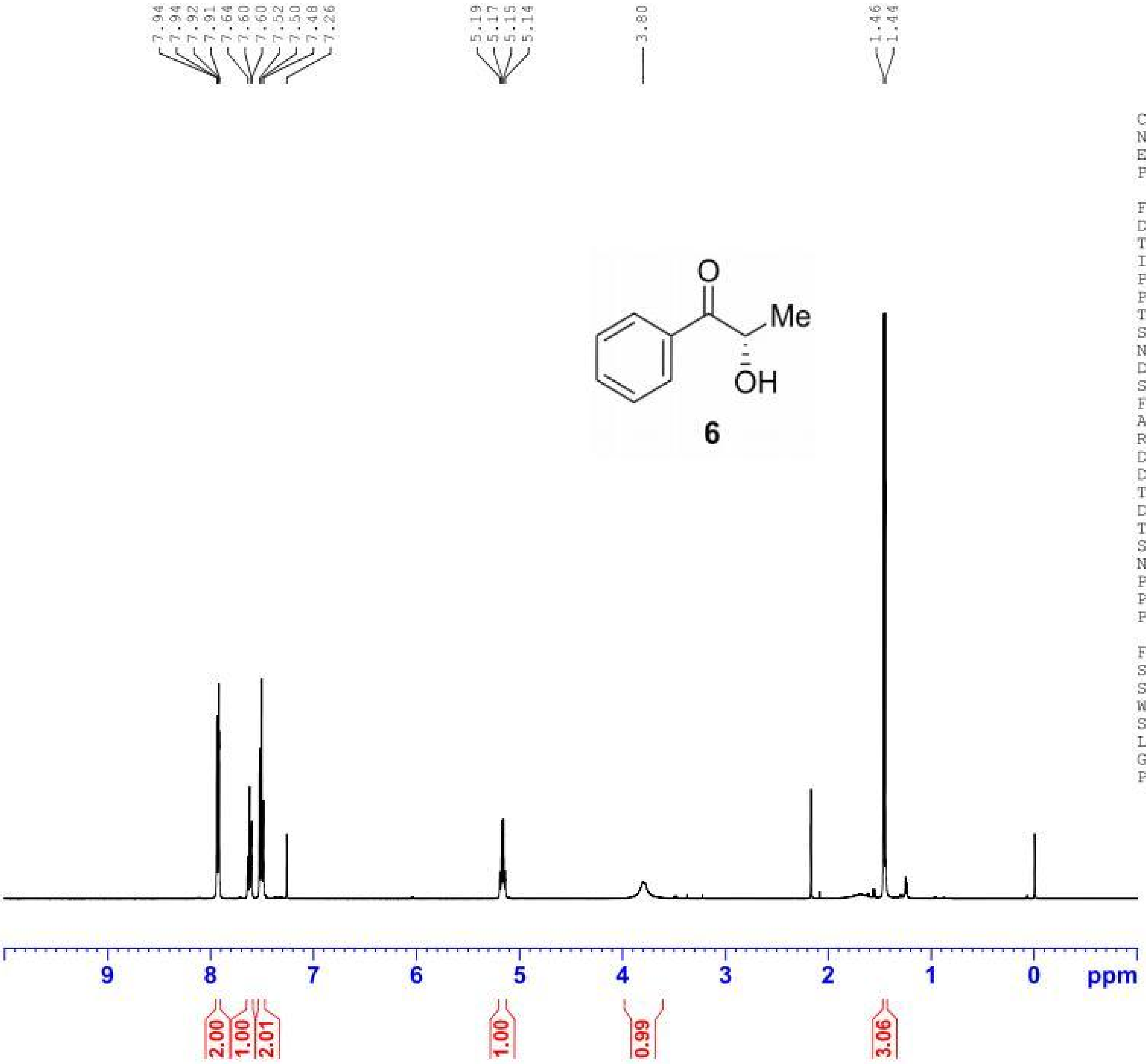

Current Data Parameters
NAME qcx-6-90-new-2
EXPNO 1
PROCNO 1

F2 - Acquisition Parameters
Date_ 20230411
Time 21.06 h
INSTRUM AvanceNeo 400MHz
PROBHD Z163739_0629 (
PULPROG zg30
TD 65536
SOLVENT CDCl3
NS 8
DS 2
SWH 8196.722 Hz
FIDRES 0.250144 Hz
AQ 3.9976959 sec
RG 101
DW 61.000 usec
DE 13.89 usec
TE 296.4 K
D1 1.00000000 sec
TD0 1
SFO1 400.1824711 MHz
NUC1 1H
P0 2.67 usec
P1 8.00 usec
PLW1 21.26700020 W

F2 - Processing parameters
SI 65536
SF 400.1800093 MHz
WDW EM
SSB 0
LB 0.30 Hz
GB 0
PC 1.00

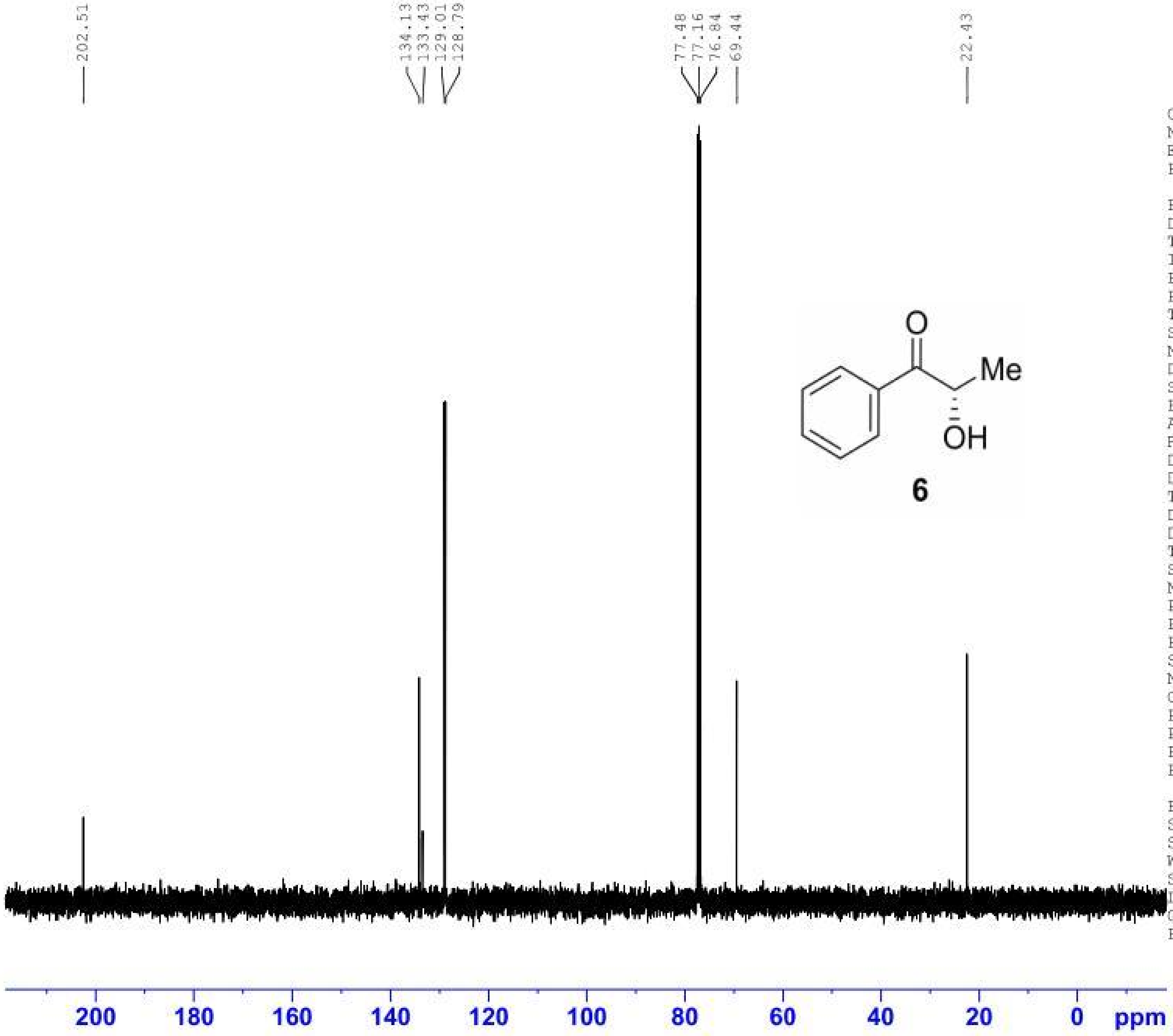

Current Data Parameters
NAME qcx-6-90-new-2
EXPNO 2
PROCNO 1

F2 - Acquisition Parameters
Date_ 20230411
Time 21.10 h
INSTRUM AvanceNeo 400MHz
PROBHD Z163739_0629 (
PULPROG zgpg30
TD 65536
SOLVENT CDCl3
NS 29
DS 4
SWH 23809.523 Hz
FIDRES 0.726609 Hz
AQ 1.3762560 sec
RG 10
DW 21.000 usec
DE 6.50 usec
TE 296.8 K
D1 2.00000000 sec
D11 0.03000000 sec
TD0 1
SFO1 100.6354036 MHz
NUC1 13C
P0 2.67 usec
P1 8.00 usec
PLW1 85.25399780 W
SFO2 400.1816007 MHz
NUC2 1H
CPDPRG[2 waltz65
PCPD2 90.00 usec
PLW2 21.26700020 W
PLW12 0.16802999 W
PLW13 0.08452000 W

F2 - Processing parameters
SI 32768
SF 100.6253293 MHz
WDW EM
SSB 0
LB 1.00 Hz
GB 0
PC 1.40

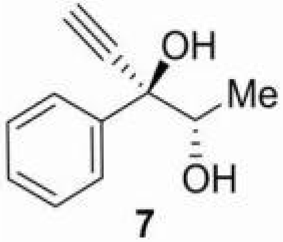

7

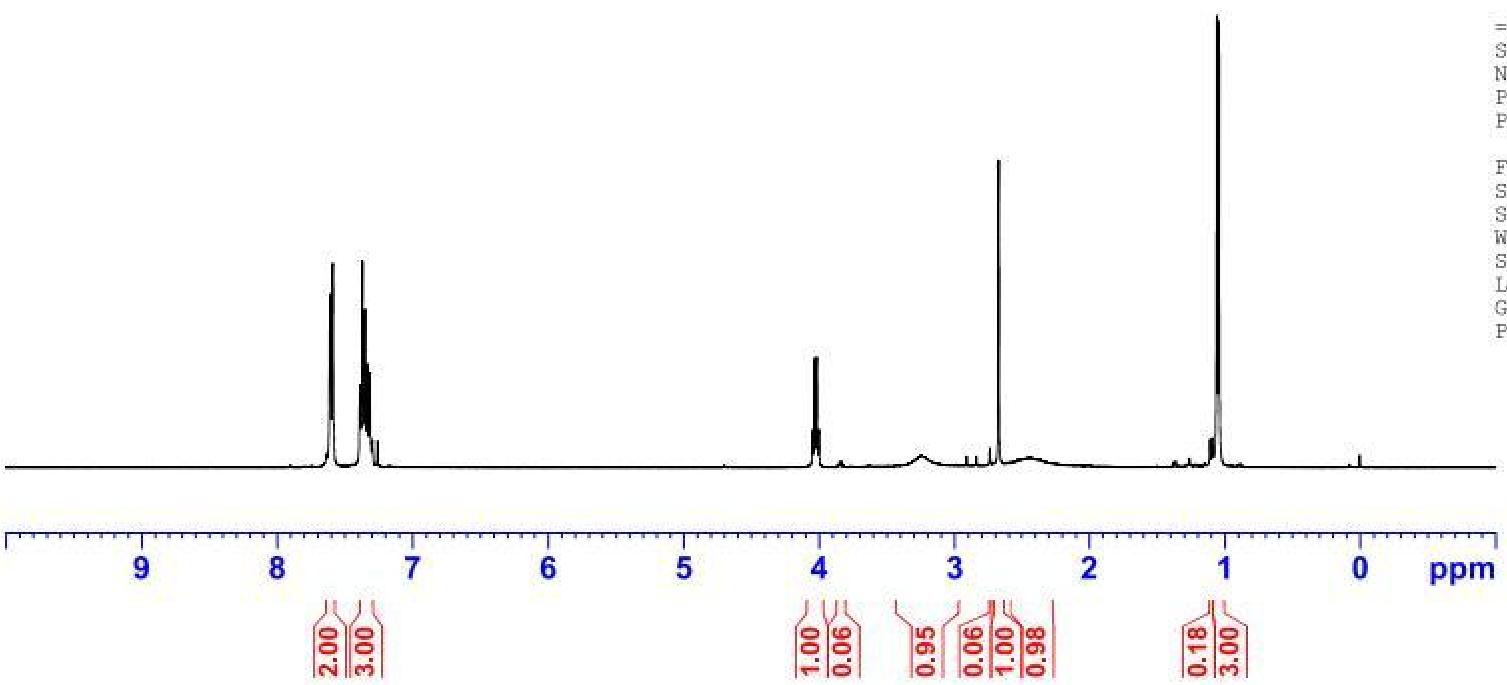

Current Data Parameters
NAME qcx-6-97-new-H
EXPNO 1
PROCNO 1

F2 - Acquisition Parameters
Date_ 20230527
Time 21.17
INSTRUM spect
PROBHD 5 mm PABBO BB/
PULPROG zg30
TD 65536
SOLVENT CDCl3
NS 6
DS 2
SWH 8012.820 Hz
FIDRES 0.122266 Hz
AQ 4.0894465 sec
RG 62.93
DW 62.400 usec
DE 6.50 usec
TE 296.6 K
D1 1.00000000 sec
TD0 1

======== CHANNEL f1 ========
SFO1 400.1324710 MHz
NUC1 1H
P1 14.50 usec
PLW1 11.99499989 W

F2 - Processing parameters
SI 65536
SF 400.1300102 MHz
WDW EM
SSB 0
LB 0.30 Hz
GB 0
PC 1.00

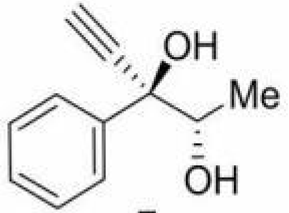

7

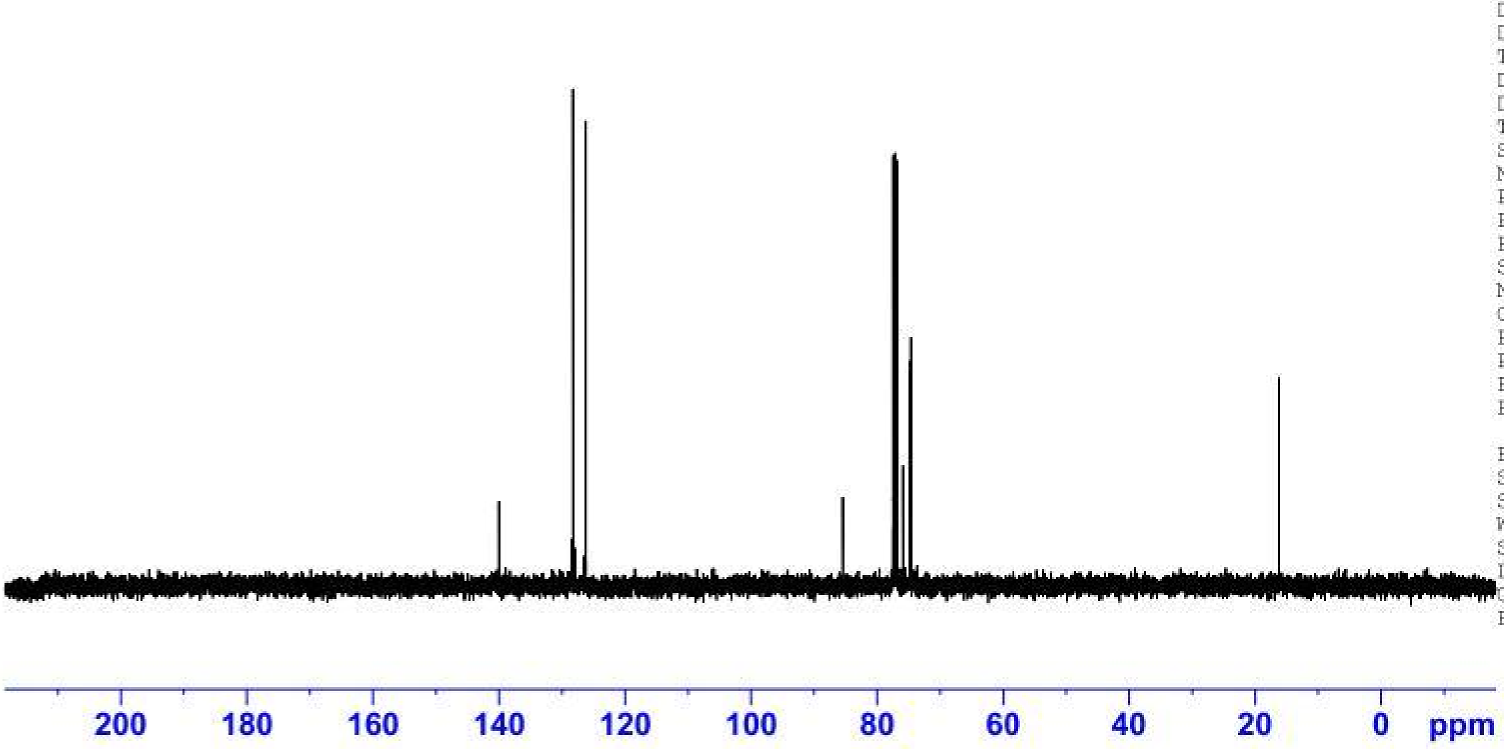


```
Current Data Parameters
NAME               qcx-6-97
EXPNO                     2
PROCNO                    1

F2 - Acquisition Parameters
Date_              20230411
Time                19.44 h
INSTRUM   AvanceNeo 400MHz
PROBHD    Z163739_0629 (
PULPROG             zgpg30
TD                   65536
SOLVENT              CDCl3
NS                      32
DS                       4
SWH              23809.523 Hz
FIDRES            0.726609 Hz
AQ               1.3762560 sec
RG                      10
DW                  21.000 usec
DE                    6.50 usec
TE                   296.5 K
D1              2.00000000 sec
D11             0.03000000 sec
TD0                      1
SFO1           100.6354036 MHz
NUC1                   13C
P0                    2.67 usec
P1                    8.00 usec
PLW1           85.25399780 W
SFO2           400.1816007 MHz
NUC2                    1H
CPDPRG[2           waltz65
PCPD2                90.00 usec
PLW2           21.26700020 W
PLW12           0.16802999 W
PLW13           0.08452000 W

F2 - Processing parameters
SI                   32768
SF             100.6253316 MHz
WDW                     EM
SSB                      0
LB                    1.00 Hz
GB                       0
PC                    1.40
```

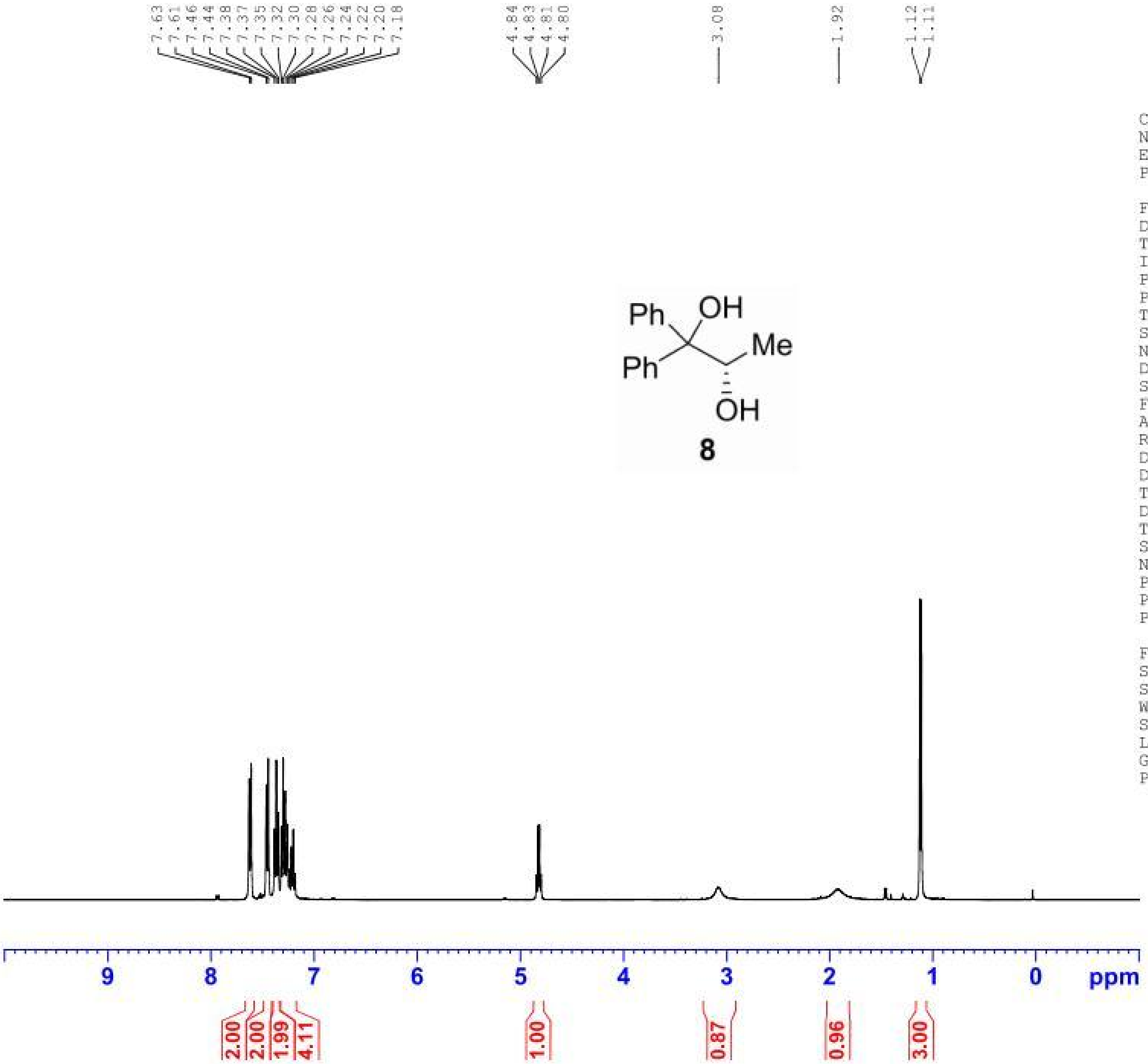

8

Current Data Parameters
NAME qcx-6-100a-new-H
EXPNO 1
PROCNO 1

F2 - Acquisition Parameters
Date_ 20230527
Time 22.38 h
INSTRUM AvanceNeo 400MHz
PROBHD Z163739_0629 (
PULPROG zg30
TD 65536
SOLVENT CDCl3
NS 13
DS 2
SWH 8196.722 Hz
FIDRES 0.250144 Hz
AQ 3.9976959 sec
RG 101
DW 61.000 usec
DE 13.89 usec
TE 298.6 K
D1 1.00000000 sec
TD0 1
SFO1 400.1824711 MHz
NUC1 1H
P0 2.67 usec
P1 8.00 usec
PLW1 21.26700020 W

F2 - Processing parameters
SI 65536
SF 400.1800075 MHz
WDW EM
SSB 0
LB 0.30 Hz
GB 0
PC 1.00

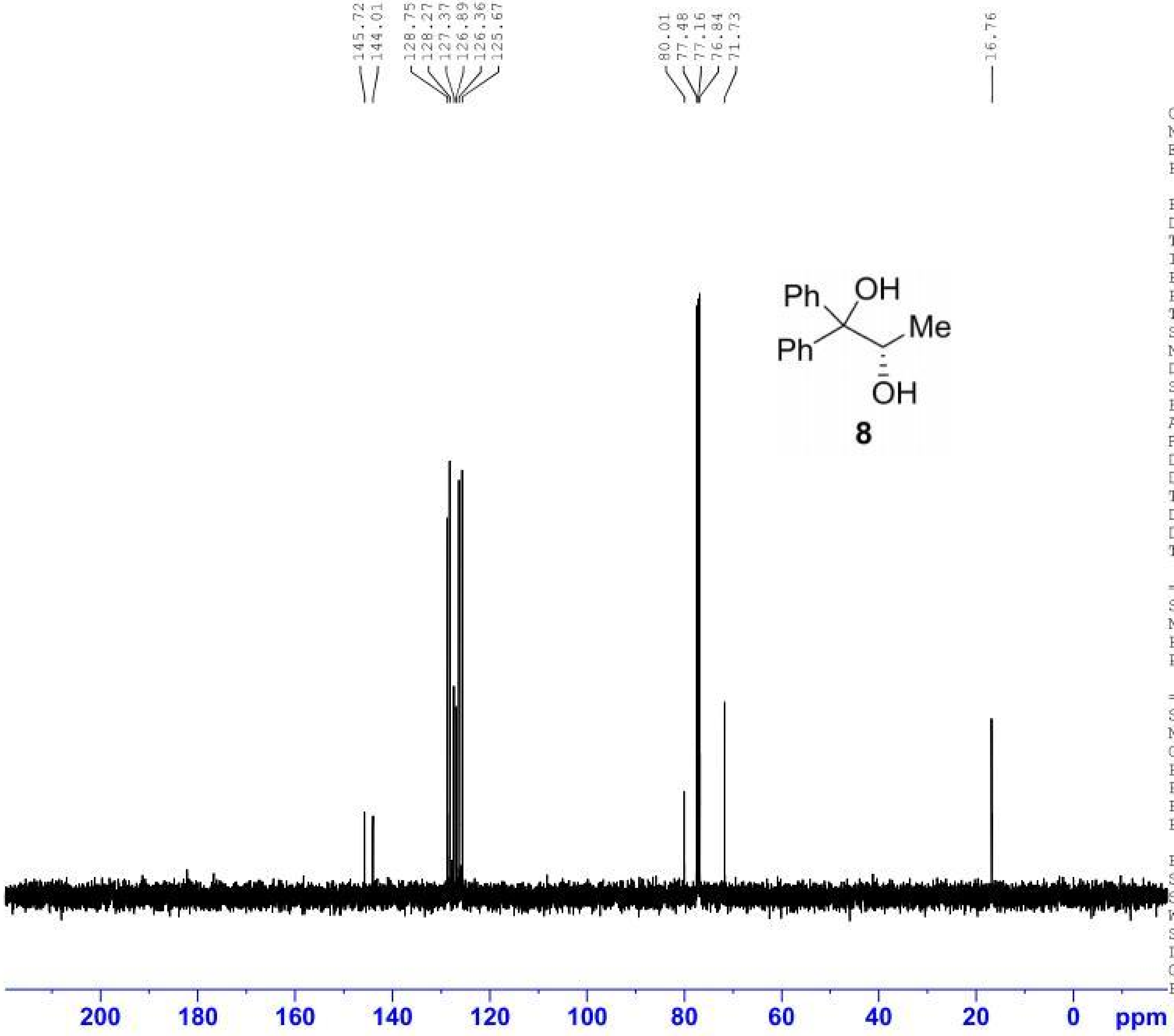


```
Current Data Parameters
NAME             qcx-6-100a
EXPNO                     2
PROCNO                    1

F2 - Acquisition Parameters
Date_              20230413
Time                  22.47
INSTRUM               spect
PROBHD   5 mm PABBO BB/
PULPROG             zgpg30
TD                    65536
SOLVENT               CDCl3
NS                       15
DS                        2
SWH               24038.461 Hz
FIDRES             0.366798 Hz
AQ                1.3631488 sec
RG                   196.92
DW                   20.800 usec
DE                     6.50 usec
TE                    296.4 K
D1               2.00000000 sec
D11              0.03000000 sec
TD0                       1

======== CHANNEL f1 ========
SFO1            100.6228298 MHz
NUC1                    13C
P1                     9.70 usec
PLW1            46.98899841 W

======== CHANNEL f2 ========
SFO2            400.1316005 MHz
NUC2                     1H
CPDPRG[2            waltz16
PCPD2                 90.00 usec
PLW2            11.99499989 W
PLW12            0.34213999 W
PLW13            0.27713001 W

F2 - Processing parameters
SI                    32768
SF              100.6127595 MHz
WDW                      EM
SSB                       0
LB                     1.00 Hz
GB                        0
PC                     1.40
```

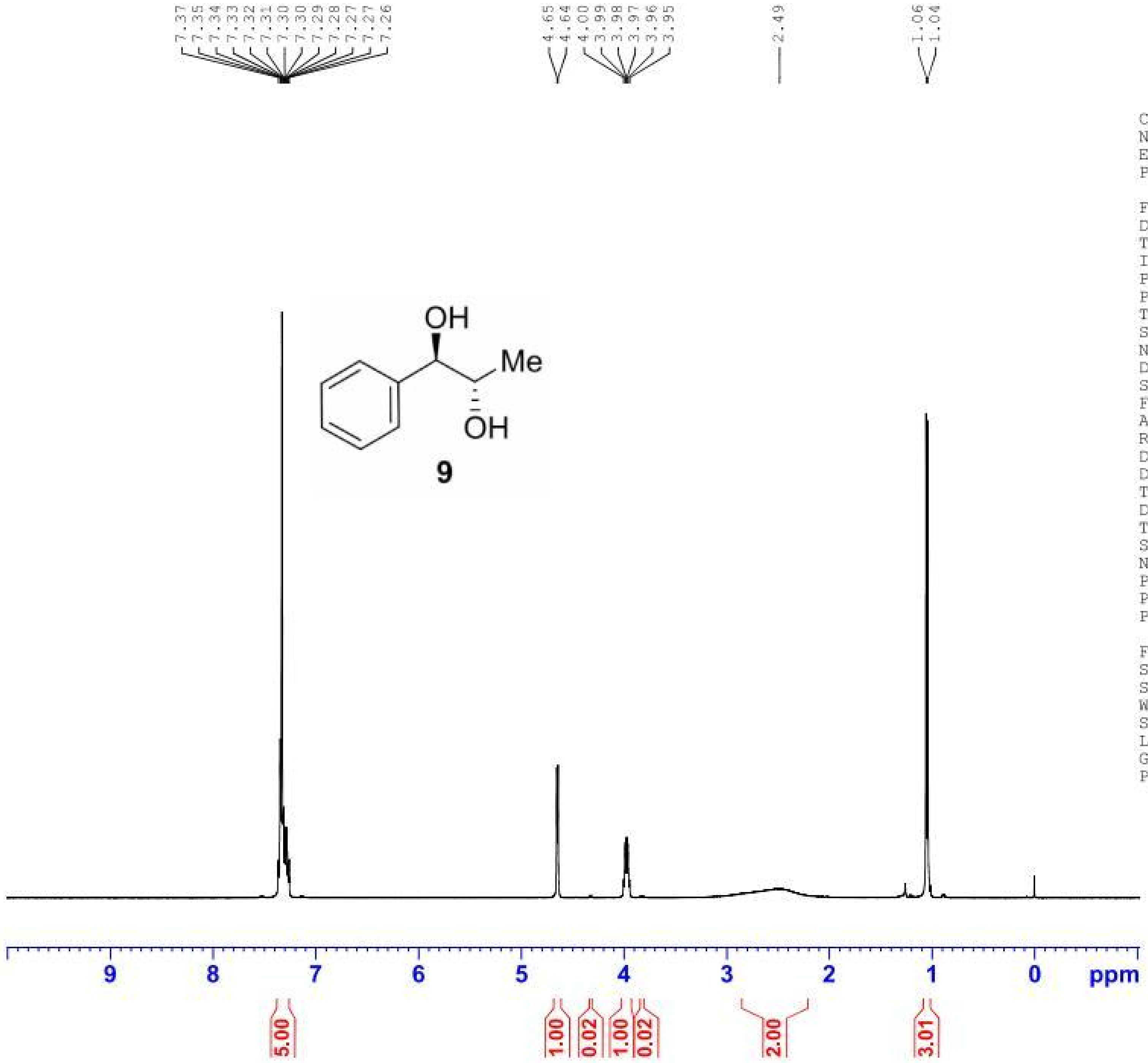


```
Current Data Parameters
NAME          qcx-6-104a
EXPNO                  1
PROCNO                 1

F2 - Acquisition Parameters
Date_           20230416
Time               18.26 h
INSTRUM   AvanceNeo 400MHz
PROBHD    Z163739_0629 (
PULPROG             zg30
TD                 65536
SOLVENT            CDCl3
NS                    11
DS                     2
SWH             8196.722 Hz
FIDRES          0.250144 Hz
AQ             3.9976959 sec
RG                   101
DW                61.000 usec
DE                 13.89 usec
TE                 296.6 K
D1            1.00000000 sec
TD0                    1
SFO1         400.1824711 MHz
NUC1                  1H
P0                  2.67 usec
P1                  8.00 usec
PLW1         21.26700020 W

F2 - Processing parameters
SI                 65536
SF           400.1800095 MHz
WDW                   EM
SSB                    0
LB                  0.30 Hz
GB                     0
PC                  1.00
```

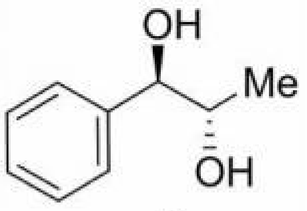

9

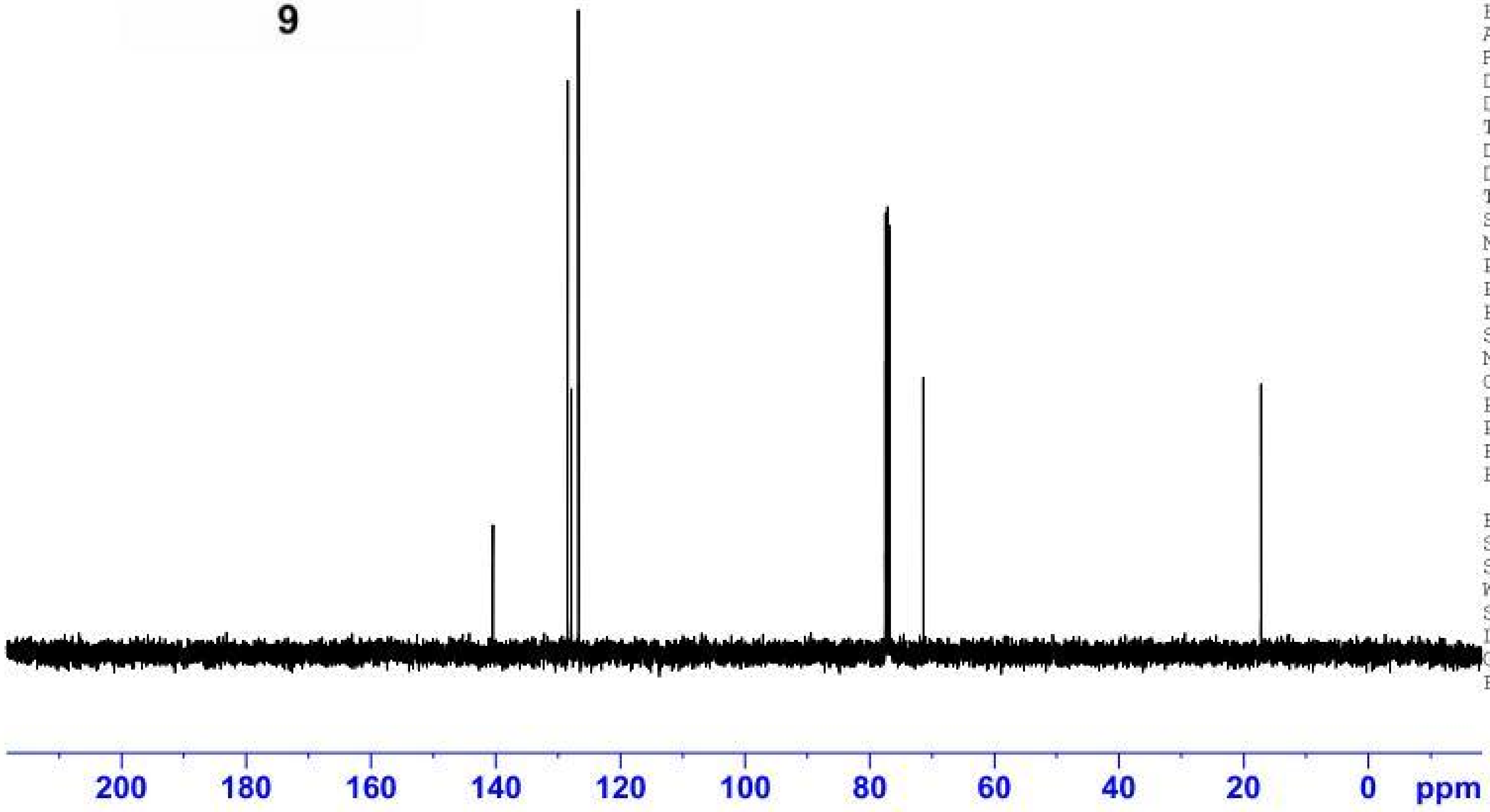

Current Data Parameters
NAME qcx-6-104a
EXPNO 2
PROCNO 1

F2 - Acquisition Parameters
Date_ 20230416
Time 18.29 h
INSTRUM AvanceNeo 400MHz
PROBHD Z163739_0629 (
PULPROG zgpg30
TD 65536
SOLVENT CDCl3
NS 10
DS 4
SWH 23809.523 Hz
FIDRES 0.726609 Hz
AQ 1.3762560 sec
RG 10
DW 21.000 usec
DE 6.50 usec
TE 297.0 K
D1 2.00000000 sec
D11 0.03000000 sec
TD0 1
SFO1 100.6354036 MHz
NUC1 13C
P0 2.67 usec
P1 8.00 usec
PLW1 85.25399780 W
SFO2 400.1816007 MHz
NUC2 1H
CPDPRG[2 waltz65
PCPD2 90.00 usec
PLW2 21.26700020 W
PLW12 0.16802999 W
PLW13 0.08452000 W

F2 - Processing parameters
SI 32768
SF 100.6253324 MHz
WDW EM
SSB 0
LB 1.00 Hz
GB 0
PC 1.40

```
=====================================================================
Acq. Operator   : SYSTEM                    Seq. Line :   6
Sample Operator : SYSTEM
Acq. Instrument : HPLC                       Location :   P2-B-01
Injection Date  : 28/2/2023 2:29:08 pm            Inj :   1
                                          Inj Volume : 2.000 µl
Different Inj Volume from Sample Entry!  Actual Inj Volume : 10.000 µl
Acq. Method     : C:\Users\Public\Documents\ChemStation\1\Data\SUN\SUN 2023-02-28 12-42-46
                  \IC3-30-60.M
Last changed    : 10/11/2022 9:42:07 am by SYSTEM
Analysis Method : C:\Users\Public\Documents\ChemStation\1\Data\SUN\SUN 2023-02-28 12-42-46
                  \IC3-30-60.M (Sequence Method)
Last changed    : 28/2/2023 4:23:41 pm by SYSTEM
                  (modified after loading)
Additional Info : Peak(s) manually integrated
```

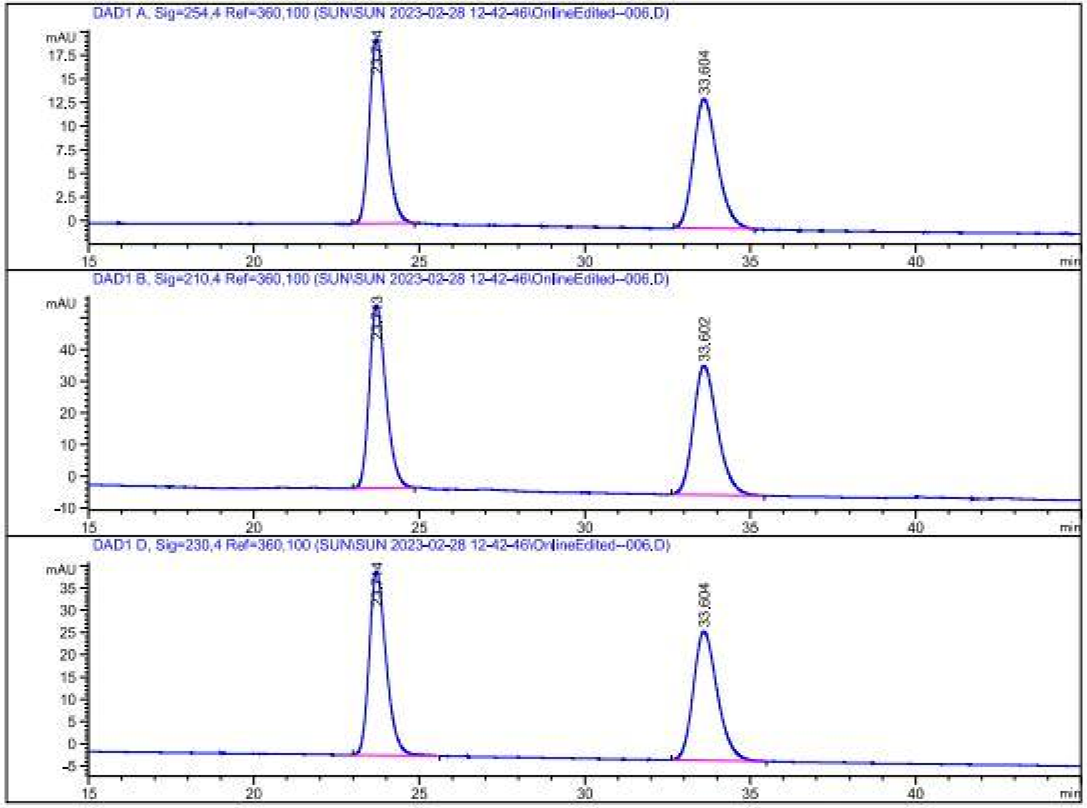

Data File C:\Users\P...\ChemStation\1\Data\SUN\SUN 2023-02-28 12-42-46\OnlineEdited--006.D
Sample Name: qcx-6-35a

```
=====================================================================
                         Area Percent Report
=====================================================================

Sorted By             :      Signal
Multiplier            :      1.0000
Dilution              :      1.0000
Use Multiplier & Dilution Factor with ISTDs
```

Signal 1: DAD1 A, Sig=254,4 Ref=360,100

Peak #	RetTime [min]	Type	Width [min]	Area [mAU*s]	Height [mAU]	Area %
1	23.714	BB	0.4239	686.31152	19.50930	50.0017
2	33.604	BB	0.5908	686.26587	13.67903	49.9983
Totals :				1372.57739	33.18834	

Signal 2: DAD1 B, Sig=210,4 Ref=360,100

Peak #	RetTime [min]	Type	Width [min]	Area [mAU*s]	Height [mAU]	Area %
1	23.713	BB	0.5024	2031.73486	57.82708	49.5852
2	33.602	BB	0.6121	2065.72363	40.80764	50.4148
Totals :				4097.45850	98.63472	

Signal 3: DAD1 D, Sig=230,4 Ref=360,100

Peak #	RetTime [min]	Type	Width [min]	Area [mAU*s]	Height [mAU]	Area %
1	23.714	BB	0.5266	1473.50720	41.31514	50.1553
2	33.604	BB	0.5993	1464.38367	28.96428	49.8447
Totals :				2937.89087	70.27942	

```
=====================================================================
                          *** End of Report ***
```

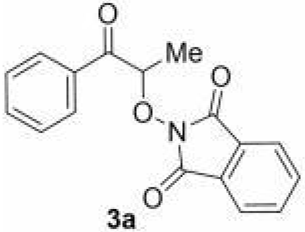

3a
racemic

===

Acq. Operator : SYSTEM Seq. Line : 7
Sample Operator : SYSTEM
Acq. Instrument : HPLC Location : P2-B-02
Injection Date : 28/2/2023 3:30:15 pm Inj : 1
Inj Volume : 2.000 µl
Different Inj Volume from Sample Entry! Actual Inj Volume : 6.000 µl
Acq. Method : C:\Users\Public\Documents\ChemStation\1\Data\SUN\SUN 2023-02-28 12-42-46
\IC3-30-60.M
Last changed : 28/2/2023 4:22:54 pm by SYSTEM
(modified after loading)
Analysis Method : C:\Users\Public\Documents\ChemStation\1\Data\SUN\SUN 2023-02-28 12-42-46
\IC3-30-60.M (Sequence Method)
Last changed : 28/2/2023 4:23:41 pm by SYSTEM
(modified after loading)
Additional Info : Peak(s) manually integrated

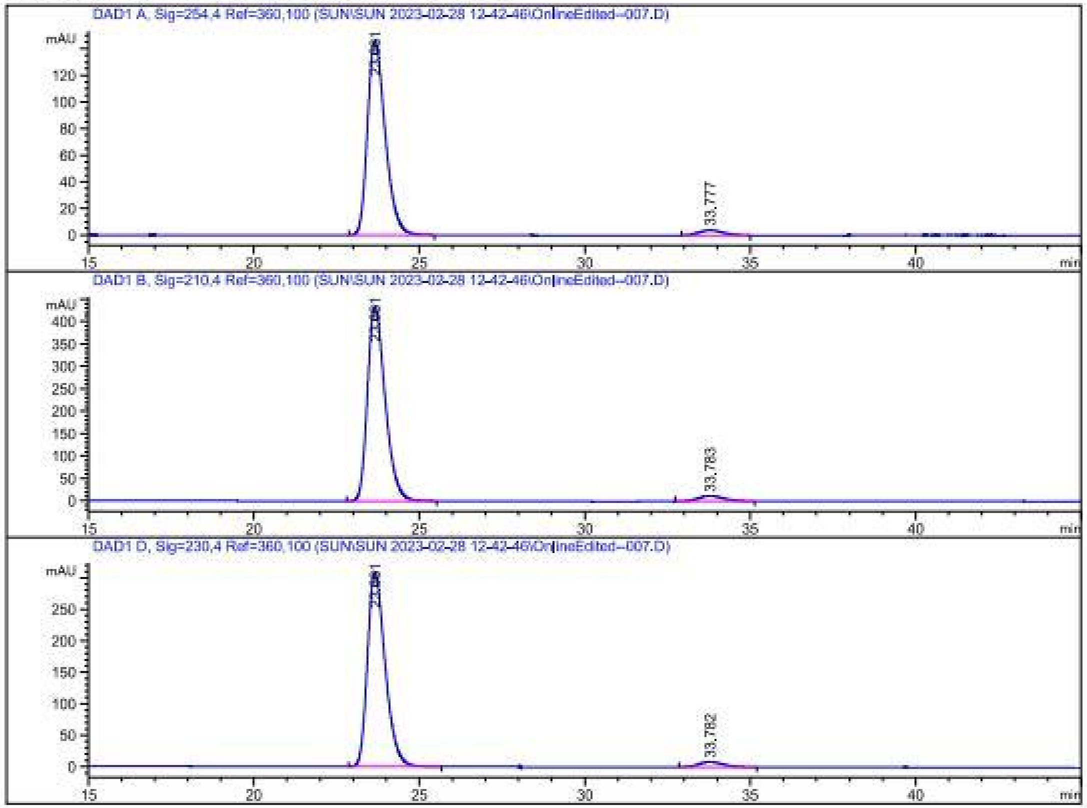

Data File C:\Users\P...\ChemStation\1\Data\SUN\SUN 2023-02-28 12-42-46\OnlineEdited--007.D
Sample Name: qcx-6-35b

===

Area Percent Report

===

Sorted By : Signal
Multiplier : 1.0000
Dilution : 1.0000
Use Multiplier & Dilution Factor with ISTDs

Signal 1: DAD1 A, Sig=254,4 Ref=360,100

Peak #	RetTime [min]	Type	Width [min]	Area [mAU*s]	Height [mAU]	Area %
1	23.661	BB	0.5603	5458.26172	146.13448	96.5504
2	33.777	BB	0.5940	195.01726	3.84635	3.4496

Totals : 5653.27898 149.98083

Signal 2: DAD1 B, Sig=210,4 Ref=360,100

Peak #	RetTime [min]	Type	Width [min]	Area [mAU*s]	Height [mAU]	Area %
1	23.661	BB	0.5709	1.61989e4	434.04980	96.3004
2	33.783	BB	0.6213	622.32770	11.75307	3.6996

Totals : 1.68213e4 445.80287

Signal 3: DAD1 D, Sig=230,4 Ref=360,100

Peak #	RetTime [min]	Type	Width [min]	Area [mAU*s]	Height [mAU]	Area %
1	23.661	BB	0.5752	1.15120e4	308.24149	96.4477
2	33.782	BB	0.6053	423.99478	8.20668	3.5523

Totals : 1.19359e4 316.44817

===

*** End of Report ***

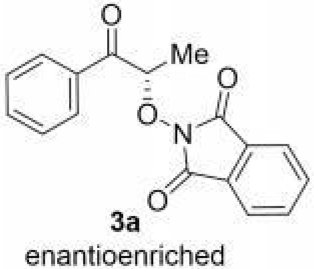

3a
enantioenriched

```
=====================================================================
Acq. Operator   : SYSTEM                    Seq. Line :  77
Sample Operator : SYSTEM
Acq. Instrument : HPLC                       Location :  P1-F-05
Injection Date  : 4/3/2023 8:51:45 am             Inj :  1
                                           Inj Volume : 2.000 µl
Different Inj Volume from Sample Entry!  Actual Inj Volume : 3.000 µl
Acq. Method     : C:\Users\Public\Documents\ChemStation\1\Data\SUN\SUN 2023-03-02 14-47-18
                  \IC3-50-60.M
Last changed    : 12/2/2023 2:10:14 pm by SYSTEM
Analysis Method : C:\Users\Public\Documents\ChemStation\1\Data\SUN\SUN 2023-03-02 14-47-18
                  \IC3-50-60.M (Sequence Method)
Last changed    : 10/3/2023 1:40:16 pm by SYSTEM
                  (modified after loading)
Additional Info : Peak(s) manually integrated
```

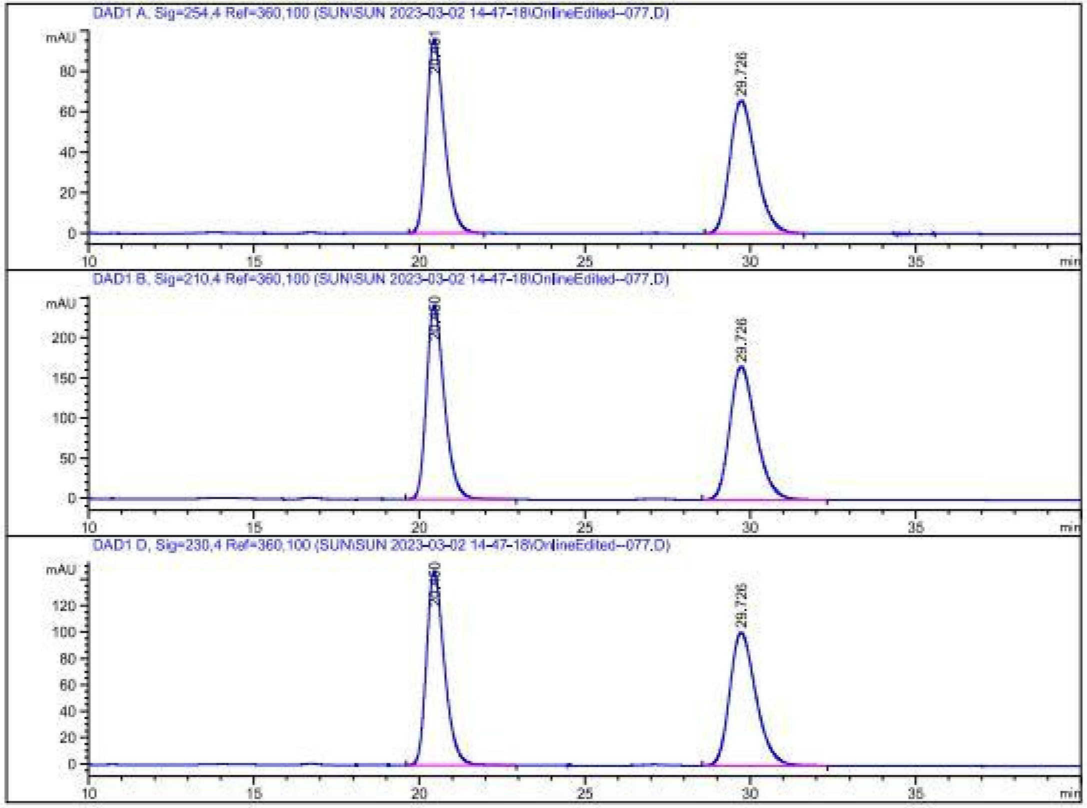

Data File C:\Users\P...\ChemStation\1\Data\SUN\SUN 2023-03-02 14-47-18\OnlineEdited--077.D
Sample Name: qcx-6-11a

===
Area Percent Report
===

```
Sorted By             :      Signal
Multiplier            :      1.0000
Dilution              :      1.0000
Use Multiplier & Dilution Factor with ISTDs
```

Signal 1: DAD1 A, Sig=254,4 Ref=360,100

Peak #	RetTime [min]	Type	Width [min]	Area [mAU*s]	Height [mAU]	Area %
1	20.451	BB	0.5572	3579.14966	95.73296	49.8788
2	29.726	BB	0.6736	3596.54663	65.65215	50.1212

Totals : 7175.69629 161.38511

Signal 2: DAD1 B, Sig=210,4 Ref=360,100

Peak #	RetTime [min]	Type	Width [min]	Area [mAU*s]	Height [mAU]	Area %
1	20.450	BB	0.5762	9145.46973	242.35677	49.9888
2	29.726	BB	0.8142	9149.58594	166.22124	50.0112

Totals : 1.82951e4 408.57800

Signal 3: DAD1 D, Sig=230,4 Ref=360,100

Peak #	RetTime [min]	Type	Width [min]	Area [mAU*s]	Height [mAU]	Area %
1	20.450	BB	0.5743	5553.15723	147.14096	49.9609
2	29.726	BB	0.8165	5561.85547	100.98696	50.0391

Totals : 1.11150e4 248.12792

===
*** End of Report ***

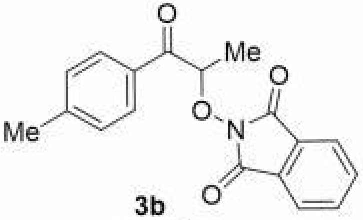

3b
racemic

```
Acq. Operator   : SYSTEM                        Seq. Line : 126
Sample Operator : SYSTEM
Acq. Instrument : HPLC                           Location :   P1-F-07
Injection Date  : 10/3/2023 4:46:31 am                Inj :   1
                                              Inj Volume : 2.000 µl
Different Inj Volume from Sample Entry!  Actual Inj Volume : 8.000 µl
Acq. Method     : C:\Users\Public\Documents\ChemStation\1\Data\SUN\SUN 2023-03-07 15-14-32
                  \IC3-50-40.M
Last changed    : 7/2/2023 9:36:14 am by SYSTEM
Analysis Method : C:\Users\Public\Documents\ChemStation\1\Data\SUN\SUN 2023-03-07 15-14-32
                  \IC3-50-40.M (Sequence Method)
Last changed    : 10/3/2023 1:38:15 pm by SYSTEM
                  (modified after loading)
Additional Info : Peak(s) manually integrated
```

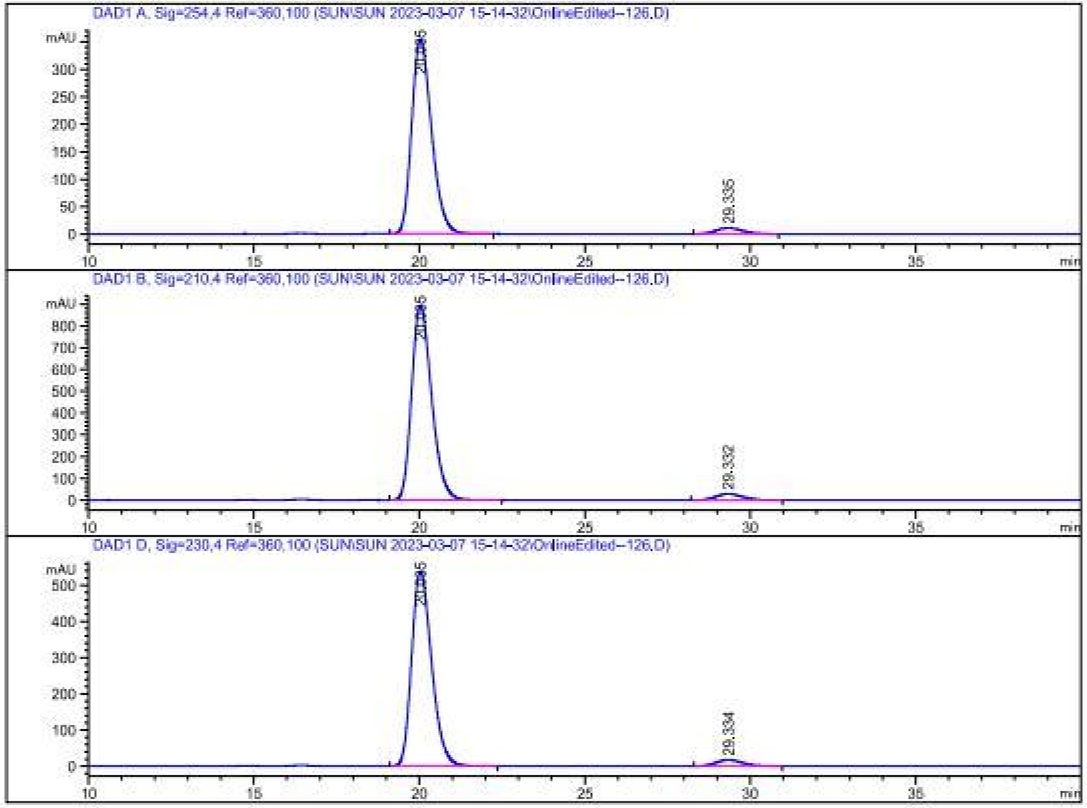

Data File C:\Users\P...\ChemStation\1\Data\SUN\SUN 2023-03-07 15-14-32\OnlineEdited--126.D
Sample Name: qcx-6-48d

Area Percent Report

```
Sorted By             :      Signal
Multiplier            :      1.0000
Dilution              :      1.0000
Use Multiplier & Dilution Factor with ISTDs
```

Signal 1: DAD1 A, Sig=254,4 Ref=360,100

Peak #	RetTime [min]	Type	Width [min]	Area [mAU*s]	Height [mAU]	Area %
1	20.035	BB	0.6373	1.48382e4	354.85660	95.8766
2	29.335	BB	0.6617	638.15253	11.35379	4.1234

Totals : 1.54764e4 366.21039

Signal 2: DAD1 B, Sig=210,4 Ref=360,100

Peak #	RetTime [min]	Type	Width [min]	Area [mAU*s]	Height [mAU]	Area %
1	20.035	BB	0.6391	3.74738e4	894.81512	95.8340
2	29.332	BB	0.6651	1629.04077	28.79013	4.1660

Totals : 3.91028e4 923.60525

Signal 3: DAD1 D, Sig=230,4 Ref=360,100

Peak #	RetTime [min]	Type	Width [min]	Area [mAU*s]	Height [mAU]	Area %
1	20.035	BB	0.6474	2.25972e4	538.69861	95.8411
2	29.334	BB	0.6637	980.58301	17.40869	4.1589

Totals : 2.35778e4 556.10729

*** End of Report ***

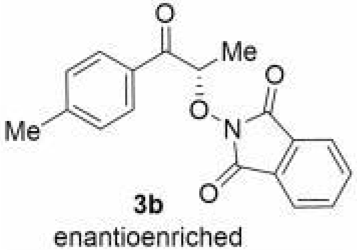

3b
enantioenriched

```
=====================================================================
Acq. Operator   : SYSTEM                    Seq. Line : 124
Sample Operator : SYSTEM
Acq. Instrument : HPLC                       Location :   P2-B-01
Injection Date  : 10/3/2023 2:44:15 am            Inj :   1
                                           Inj Volume : 2.000 µl
Different Inj Volume from Sample Entry!  Actual Inj Volume : 20.000 µl
Acq. Method     : C:\Users\Public\Documents\ChemStation\1\Data\SUN\SUN 2023-03-07 15-14-32
                  \IC3-50-60.M
Last changed    : 12/2/2023 2:10:14 pm by SYSTEM
Analysis Method : C:\Users\Public\Documents\ChemStation\1\Data\SUN\SUN 2023-03-07 15-14-32
                  \IC3-50-60.M (Sequence Method)
Last changed    : 10/3/2023 1:35:15 pm by SYSTEM
                  (modified after loading)
Additional Info : Peak(s) manually integrated
```

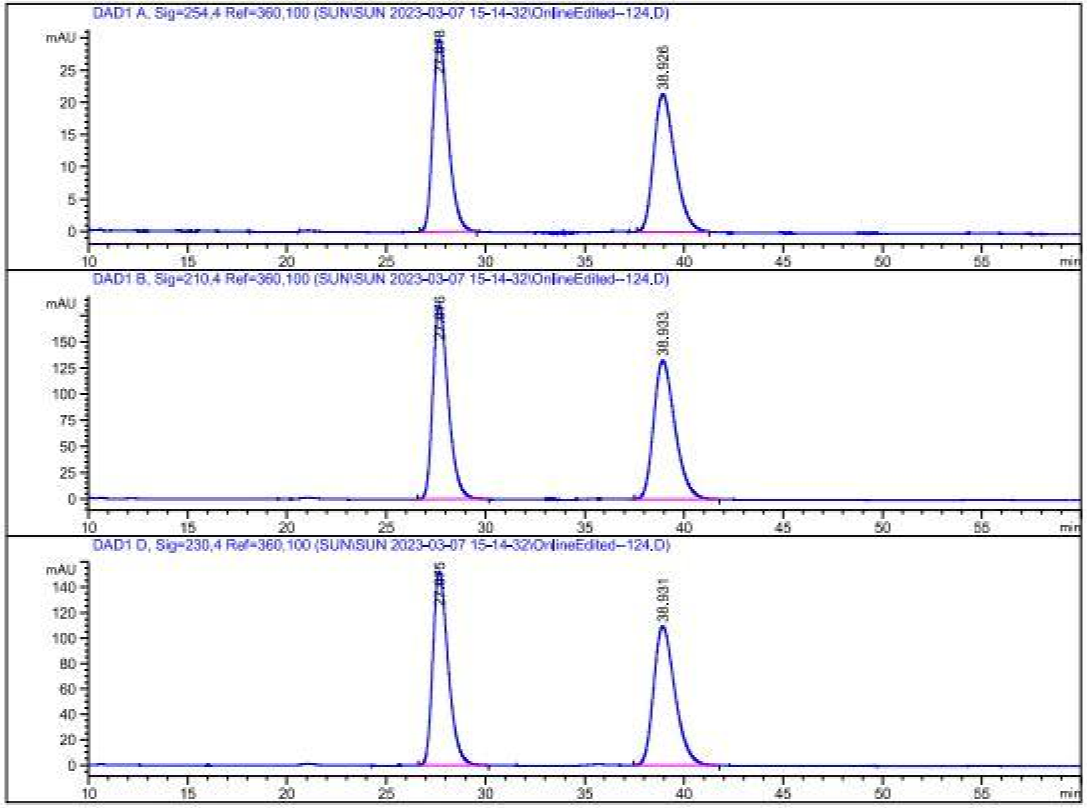

Data File C:\Users\P...\ChemStation\1\Data\SUN\SUN 2023-03-07 15-14-32\OnlineEdited--124.D
Sample Name: qcx-6-54a

===
Area Percent Report
===

```
Sorted By             :      Signal
Multiplier            :      1.0000
Dilution              :      1.0000
Use Multiplier & Dilution Factor with ISTDs
```

Signal 1: DAD1 A, Sig=254,4 Ref=360,100

Peak #	RetTime [min]	Type	Width [min]	Area [mAU*s]	Height [mAU]	Area %
1	27.678	BB	0.6322	1590.48340	29.87294	50.1043
2	38.926	BB	0.8685	1583.85901	21.34921	49.8957
Totals :				3174.34241	51.22215	

Signal 2: DAD1 B, Sig=210,4 Ref=360,100

Peak #	RetTime [min]	Type	Width [min]	Area [mAU*s]	Height [mAU]	Area %
1	27.676	BB	0.7652	9936.65332	185.41924	50.1336
2	38.933	BB	0.8872	9883.71094	132.51120	49.8664
Totals :				1.98204e4	317.93044	

Signal 3: DAD1 D, Sig=230,4 Ref=360,100

Peak #	RetTime [min]	Type	Width [min]	Area [mAU*s]	Height [mAU]	Area %
1	27.675	BB	0.7818	8195.55469	152.78271	50.0432
2	38.931	BB	0.9870	8181.39307	109.45634	49.9568
Totals :				1.63769e4	262.23905	

===
*** End of Report ***

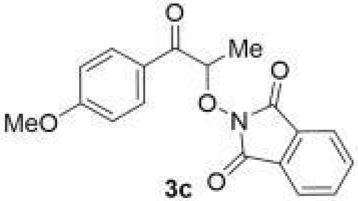

3c
racemic

Acq. Operator : SYSTEM   Seq. Line : 125
Sample Operator : SYSTEM
Acq. Instrument : HPLC   Location : P1-F-06
Injection Date : 10/3/2023 3:45:24 am   Inj : 1
Inj Volume : 2.000 µl
Different Inj Volume from Sample Entry! Actual Inj Volume : 8.000 µl
Acq. Method : C:\Users\Public\Documents\ChemStation\1\Data\SUN\SUN 2023-03-07 15-14-32\IC3-50-60.M
Last changed : 12/2/2023 2:10:14 pm by SYSTEM
Analysis Method : C:\Users\Public\Documents\ChemStation\1\Data\SUN\SUN 2023-03-07 15-14-32\IC3-50-60.M (Sequence Method)
Last changed : 10/3/2023 1:35:15 pm by SYSTEM
(modified after loading)
Additional Info : Peak(s) manually integrated

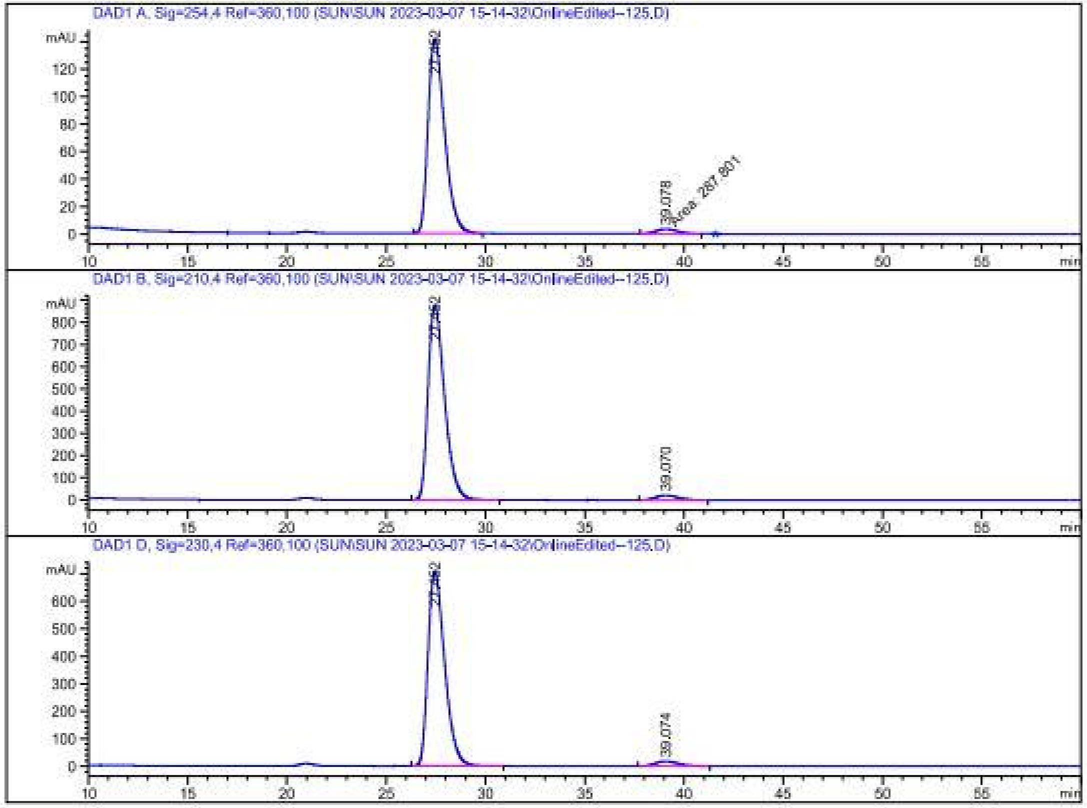

Data File C:\Users\P...\ChemStation\1\Data\SUN\SUN 2023-03-07 15-14-32\OnlineEdited--125.D
Sample Name: qcx-6-48c

Area Percent Report

Sorted By : Signal
Multiplier : 1.0000
Dilution : 1.0000
Use Multiplier & Dilution Factor with ISTDs

Signal 1: DAD1 A, Sig=254,4 Ref=360,100

Peak #	RetTime [min]	Type	Width [min]	Area [mAU*s]	Height [mAU]	Area %
1	27.452	BB	0.7981	8169.65186	141.39999	96.5971
2	39.078	MM	1.3176	287.80081	3.64035	3.4029

Totals : 8457.45267 145.04034

Signal 2: DAD1 B, Sig=210,4 Ref=360,100

Peak #	RetTime [min]	Type	Width [min]	Area [mAU*s]	Height [mAU]	Area %
1	27.452	BB	0.8372	5.07369e4	876.03339	96.7869
2	39.070	BB	0.8992	1684.33960	21.95100	3.2131

Totals : 5.24212e4 897.98439

Signal 3: DAD1 D, Sig=230,4 Ref=360,100

Peak #	RetTime [min]	Type	Width [min]	Area [mAU*s]	Height [mAU]	Area %
1	27.452	BB	0.8848	4.13465e4	708.58319	96.7145
2	39.074	BB	0.9039	1404.59131	18.21030	3.2855

Totals : 4.27511e4 726.79349

*** End of Report ***

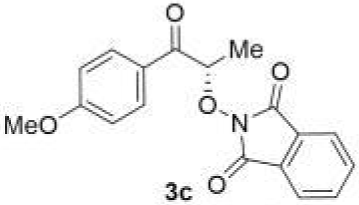

3c
enantioenriched

===

```
Acq. Operator   : SYSTEM                    Seq. Line :   6
Sample Operator : SYSTEM
Acq. Instrument : HPLC                       Location :   P2-B-03
Injection Date  : 27/3/2023 4:01:34 pm            Inj :   1
                                           Inj Volume : 2.000 µl
Different Inj Volume from Sample Entry!  Actual Inj Volume : 3.000 µl
Acq. Method     : C:\Users\Public\Documents\ChemStation\1\Data\SUN\SUN 2023-03-27 13-30-18
                  \IC3-50-60.M
Last changed    : 12/2/2023 2:10:14 pm by SYSTEM
Analysis Method : C:\Users\Public\Documents\ChemStation\1\Data\SUN\SUN 2023-03-27 13-30-18
                  \IC3-50-60.M (Sequence Method)
Last changed    : 2/6/2023 3:04:44 pm by SYSTEM
                  (modified after loading)
Additional Info : Peak(s) manually integrated
```

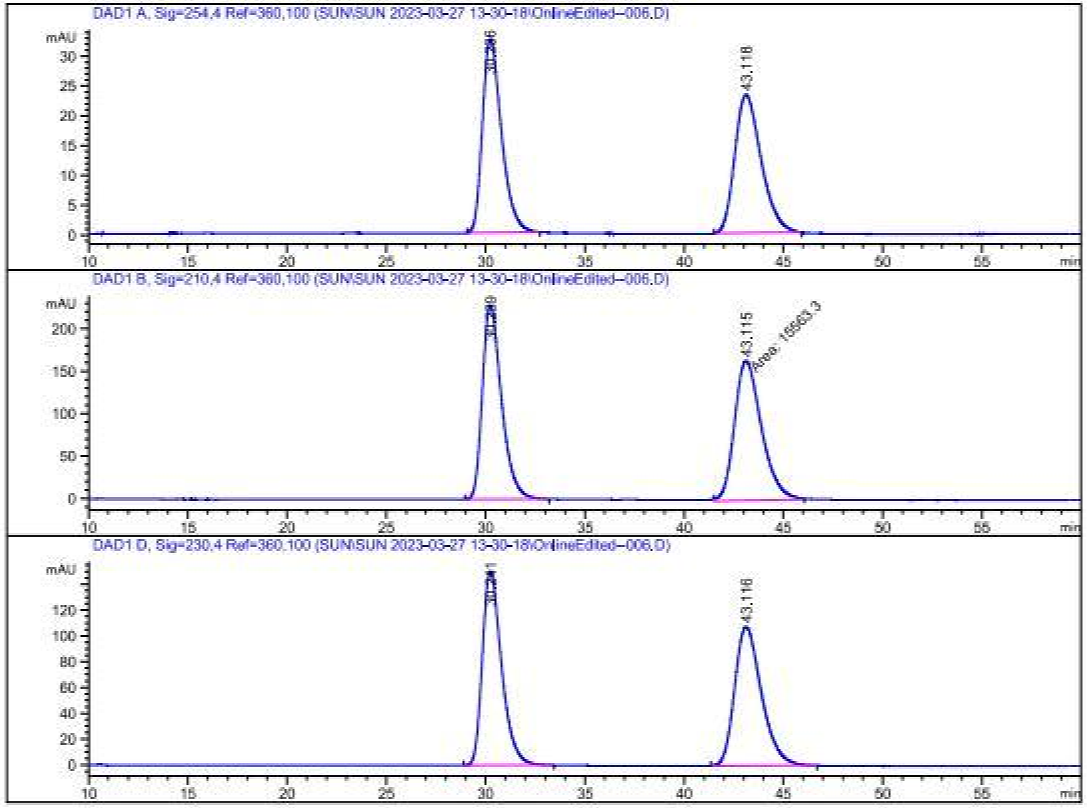

Data File C:\Users\P...\ChemStation\1\Data\SUN\SUN 2023-03-27 13-30-18\OnlineEdited--006.D
Sample Name: qcx-6-77a-rac

===
Area Percent Report
===

```
Sorted By          :      Signal
Multiplier         :      1.0000
Dilution           :      1.0000
Use Multiplier & Dilution Factor with ISTDs
```

Signal 1: DAD1 A, Sig=254,4 Ref=360,100

Peak #	RetTime [min]	Type	Width [min]	Area [mAU*s]	Height [mAU]	Area %
1	30.236	BB	0.7851	2165.58716	32.44888	50.1831
2	43.118	BB	1.0853	2149.78613	23.15330	49.8169

Totals : 4315.37329 55.60218

Signal 2: DAD1 B, Sig=210,4 Ref=360,100

Peak #	RetTime [min]	Type	Width [min]	Area [mAU*s]	Height [mAU]	Area %
1	30.239	BB	0.9577	1.53264e4	228.60298	49.6165
2	43.115	MM	1.5766	1.55633e4	164.52223	50.3835

Totals : 3.08897e4 393.12521

Signal 3: DAD1 D, Sig=230,4 Ref=360,100

Peak #	RetTime [min]	Type	Width [min]	Area [mAU*s]	Height [mAU]	Area %
1	30.241	BB	0.9260	1.01321e4	150.67506	50.0216
2	43.116	BB	1.1037	1.01234e4	107.79493	49.9784

Totals : 2.02555e4 258.46999

===
*** End of Report ***

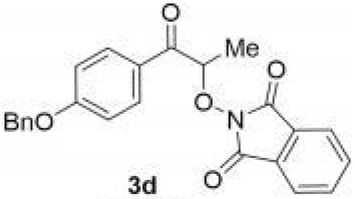

3d
racemic

===

Acq. Operator : SYSTEM Seq. Line : 7
Sample Operator : SYSTEM
Acq. Instrument : HPLC Location : P2-B-04
Injection Date : 27/3/2023 5:02:36 pm Inj : 1
Inj Volume : 2.000 µl
Different Inj Volume from Sample Entry! Actual Inj Volume : 6.000 µl
Acq. Method : C:\Users\Public\Documents\ChemStation\1\Data\SUN\SUN 2023-03-27 13-30-18\IC3-50-60.M
Last changed : 12/2/2023 2:10:14 pm by SYSTEM
Analysis Method : C:\Users\Public\Documents\ChemStation\1\Data\SUN\SUN 2023-03-27 13-30-18\IC3-50-60.M (Sequence Method)
Last changed : 27/3/2023 7:40:00 pm by SYSTEM
(modified after loading)
Additional Info : Peak(s) manually integrated

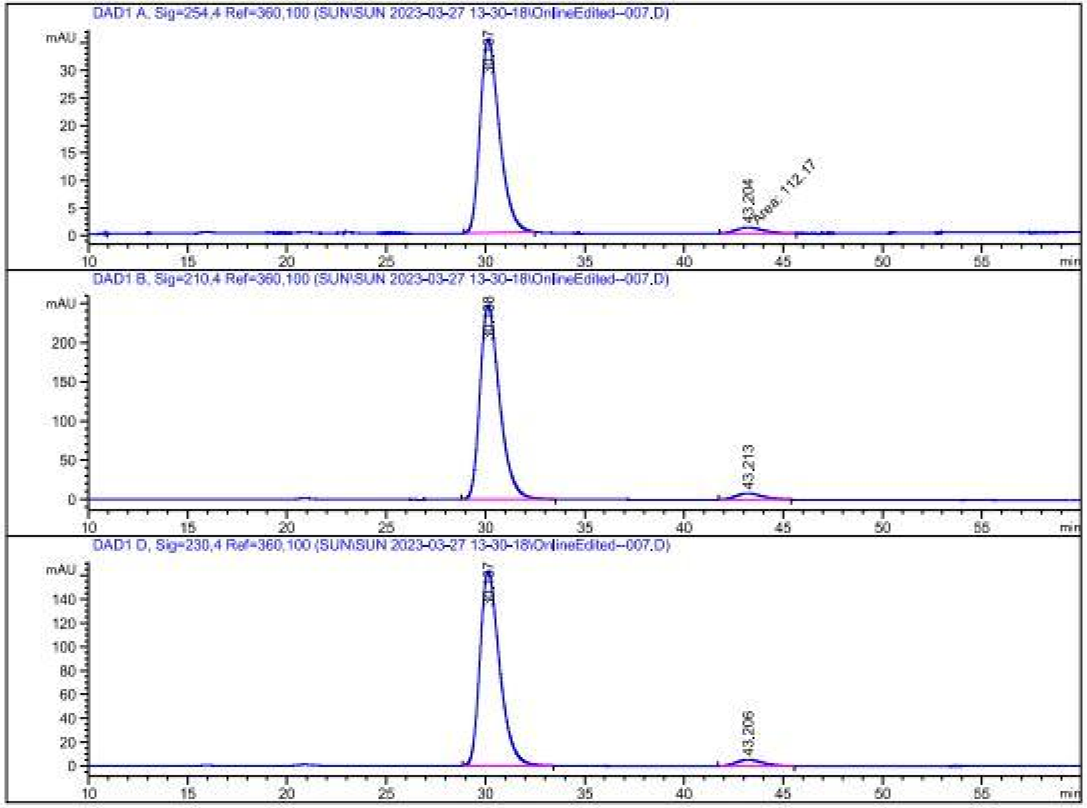

Data File C:\Users\P...\ChemStation\1\Data\SUN\SUN 2023-03-27 13-30-18\OnlineEdited--007.D
Sample Name: qcx-6-77a

===
Area Percent Report
===

Sorted By : Signal
Multiplier : 1.0000
Dilution : 1.0000
Use Multiplier & Dilution Factor with ISTDs

Signal 1: DAD1 A, Sig=254,4 Ref=360,100

Peak #	RetTime [min]	Type	Width [min]	Area [mAU*s]	Height [mAU]	Area %
1	30.137	BB	0.8009	2397.60229	35.18859	95.5307
2	43.204	MM	1.6016	112.16981	1.16728	4.4693

Totals : 2509.77210 36.35587

Signal 2: DAD1 B, Sig=210,4 Ref=360,100

Peak #	RetTime [min]	Type	Width [min]	Area [mAU*s]	Height [mAU]	Area %
1	30.138	BB	0.9598	1.70558e4	248.02928	95.8616
2	43.213	BB	1.0810	736.30603	7.97250	4.1384

Totals : 1.77921e4 256.00178

Signal 3: DAD1 D, Sig=230,4 Ref=360,100

Peak #	RetTime [min]	Type	Width [min]	Area [mAU*s]	Height [mAU]	Area %
1	30.137	BB	0.9831	1.12322e4	163.26813	95.7686
2	43.206	BB	1.0940	496.27493	5.30928	4.2314

Totals : 1.17284e4 168.57740

===
*** End of Report ***

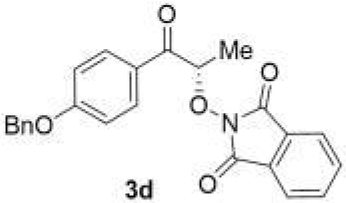

3d
enantioenriched

===

```
Acq. Operator   : SYSTEM                     Seq. Line :   60
Sample Operator : SYSTEM
Acq. Instrument : HPLC                        Location :   P1-F-07
Injection Date  : 12/3/2023 5:34:58 pm             Inj :   1
                                          Inj Volume : 2.000 µl
Different Inj Volume from Sample Entry!  Actual Inj Volume : 3.000 µl
Acq. Method     : C:\Users\Public\Documents\ChemStation\1\Data\SUN\SUN 2023-03-11 14-02-31
                  \IC3-30-60.M
Last changed    : 10/11/2022 9:42:07 am by SYSTEM
Analysis Method : C:\Users\Public\Documents\ChemStation\1\Data\SUN\SUN 2023-03-11 14-02-31
                  \IC3-30-60.M (Sequence Method)
Last changed    : 13/3/2023 7:41:30 pm by SYSTEM
                  (modified after loading)
Additional Info : Peak(s) manually integrated
```

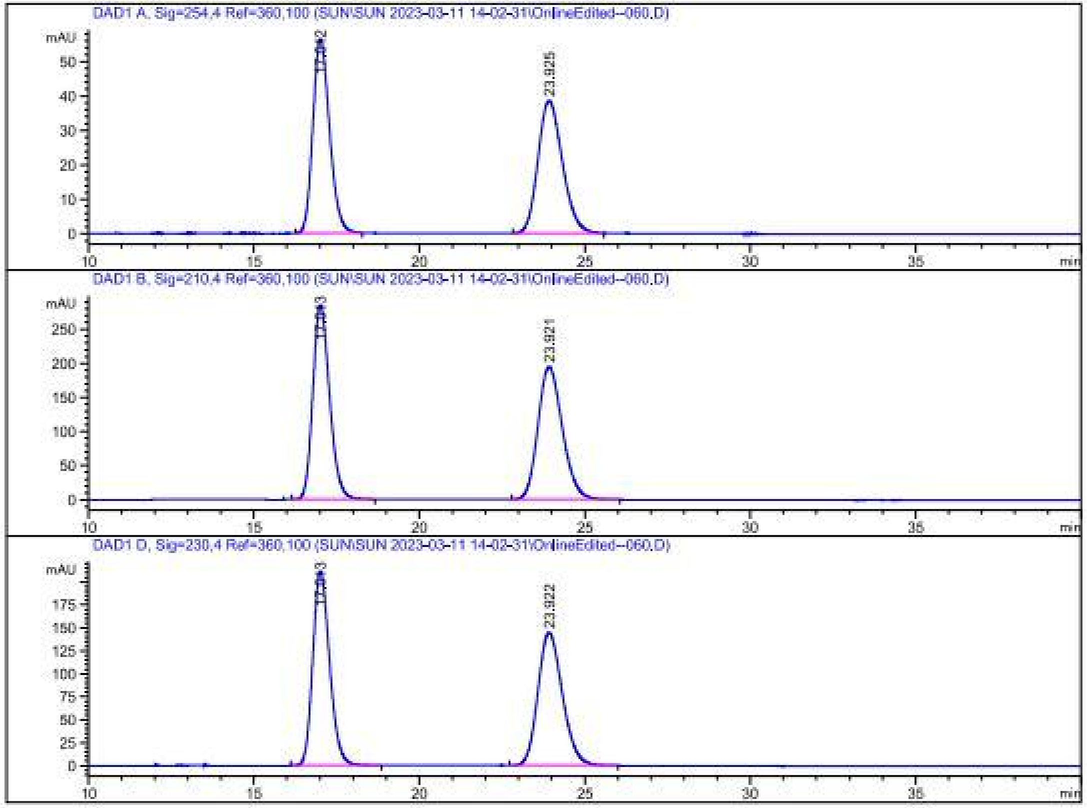

Data File C:\Users\P...\ChemStation\1\Data\SUN\SUN 2023-03-11 14-02-31\OnlineEdited--060.D
Sample Name: qcx-6-56h

===

Area Percent Report

===

```
Sorted By             :      Signal
Multiplier            :      1.0000
Dilution              :      1.0000
Use Multiplier & Dilution Factor with ISTDs
```

Signal 1: DAD1 A, Sig=254,4 Ref=360,100

Peak #	RetTime [min]	Type	Width [min]	Area [mAU*s]	Height [mAU]	Area %
1	17.012	BB	0.5222	1970.72229	56.25536	49.9870
2	23.925	BB	0.6045	1971.74377	38.43311	50.0130

Totals : 3942.46606 94.68847

Signal 2: DAD1 B, Sig=210,4 Ref=360,100

Peak #	RetTime [min]	Type	Width [min]	Area [mAU*s]	Height [mAU]	Area %
1	17.013	BB	0.5299	1.00153e4	285.05939	50.0151
2	23.921	BB	0.7470	1.00093e4	194.63385	49.9849

Totals : 2.00246e4 479.69324

Signal 3: DAD1 D, Sig=230,4 Ref=360,100

Peak #	RetTime [min]	Type	Width [min]	Area [mAU*s]	Height [mAU]	Area %
1	17.013	BB	0.5441	7452.80811	211.51508	49.9953
2	23.922	BB	0.7463	7454.21143	144.74452	50.0047

Totals : 1.49070e4 356.25960

===

*** End of Report ***

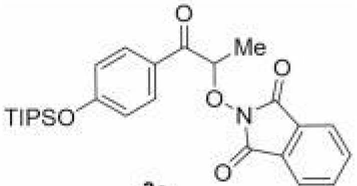

3e
racemic

```
=====================================================================
Acq. Operator   : SYSTEM                      Seq. Line :  66
Sample Operator : SYSTEM
Acq. Instrument : HPLC                         Location :   P2-A-09
Injection Date  : 17/3/2023 3:35:46 am              Inj :   1
                                           Inj Volume : 2.000 µl
Different Inj Volume from Sample Entry!  Actual Inj Volume : 8.000 µl
Acq. Method     : C:\Users\Public\Documents\ChemStation\1\Data\SUN\SUN 2023-03-15 22-36-05
                  \IC3-30-40.M
Last changed    : 27/2/2023 10:13:57 pm by SYSTEM
Analysis Method : C:\Users\Public\Documents\ChemStation\1\Data\SUN\SUN 2023-03-15 22-36-05
                  \IC3-30-40.M (Sequence Method)
Last changed    : 17/3/2023 1:42:44 pm by SYSTEM
                  (modified after loading)
Additional Info : Peak(s) manually integrated
```

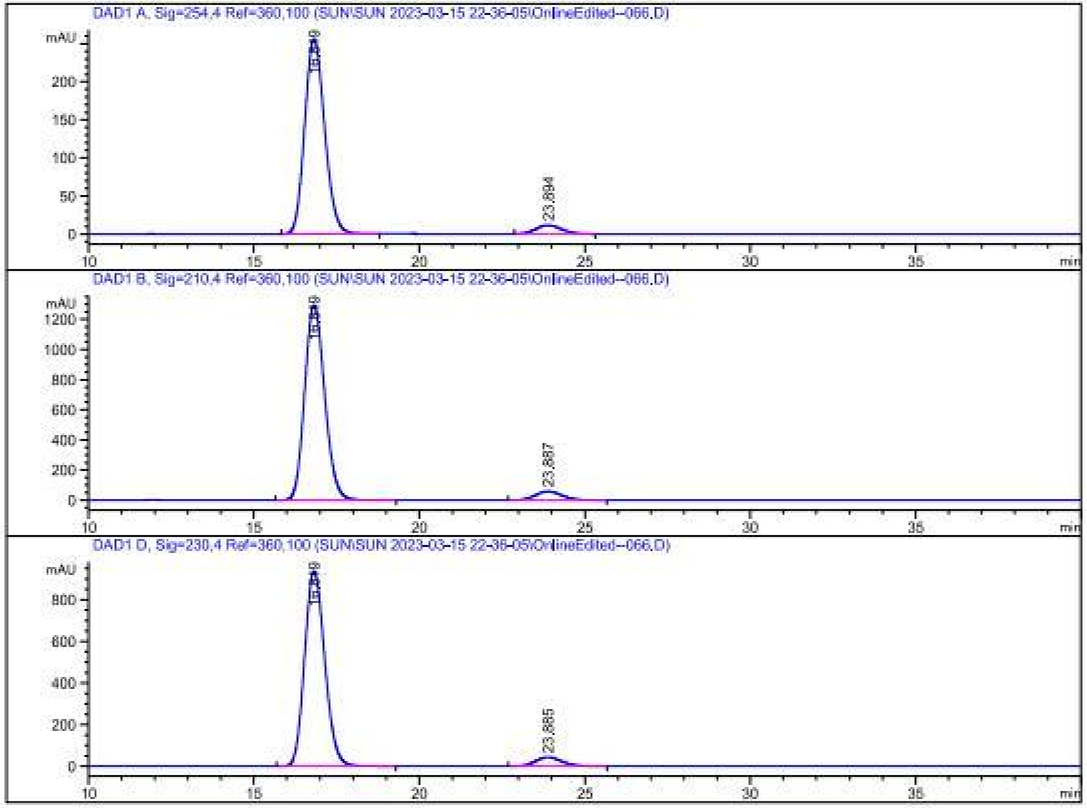

Data File C:\Users\P...\ChemStation\1\Data\SUN\SUN 2023-03-15 22-36-05\OnlineEdited--066.D
Sample Name: qcx-6-56d

Area Percent Report

```
Sorted By             :      Signal
Multiplier            :      1.0000
Dilution              :      1.0000
Use Multiplier & Dilution Factor with ISTDs
```

Signal 1: DAD1 A, Sig=254,4 Ref=360,100

Peak #	RetTime [min]	Type	Width [min]	Area [mAU*s]	Height [mAU]	Area %
1	16.819	BB	0.6435	1.07421e4	256.04315	94.4932
2	23.894	BB	0.6426	626.02094	11.42750	5.5068

Totals : 1.13682e4 267.47065

Signal 2: DAD1 B, Sig=210,4 Ref=360,100

Peak #	RetTime [min]	Type	Width [min]	Area [mAU*s]	Height [mAU]	Area %
1	16.819	BB	0.6397	5.43772e4	1294.07141	94.4357
2	23.887	BB	0.6657	3204.00513	57.84781	5.5643

Totals : 5.75812e4 1351.91922

Signal 3: DAD1 D, Sig=230,4 Ref=360,100

Peak #	RetTime [min]	Type	Width [min]	Area [mAU*s]	Height [mAU]	Area %
1	16.819	BB	0.6617	3.97262e4	937.78119	94.3206
2	23.885	BB	0.6506	2392.05249	43.16193	5.6794

Totals : 4.21183e4 980.94312

*** End of Report ***

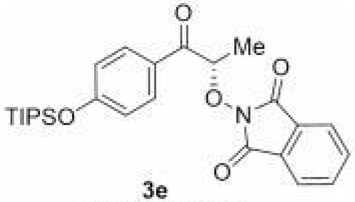

3e
enantioenriched

===

Acq. Operator : SYSTEM Seq. Line : 9
Sample Operator : SYSTEM
Acq. Instrument : HPLC Location : P1-F-09
Injection Date : 7/3/2023 1:46:28 am Inj : 1
Inj Volume : 2.000 µl
Different Inj Volume from Sample Entry! Actual Inj Volume : 4.000 µl
Acq. Method : C:\Users\Public\Documents\ChemStation\1\Data\SUN\SUN 2023-03-06 22-12-22 \IC3-30-40.M
Last changed : 27/2/2023 10:13:57 pm by SYSTEM
Analysis Method : C:\Users\Public\Documents\ChemStation\1\Data\SUN\SUN 2023-03-06 22-12-22 \IC3-30-40.M (Sequence Method)
Last changed : 7/3/2023 1:29:31 pm by SYSTEM
(modified after loading)
Additional Info : Peak(s) manually integrated

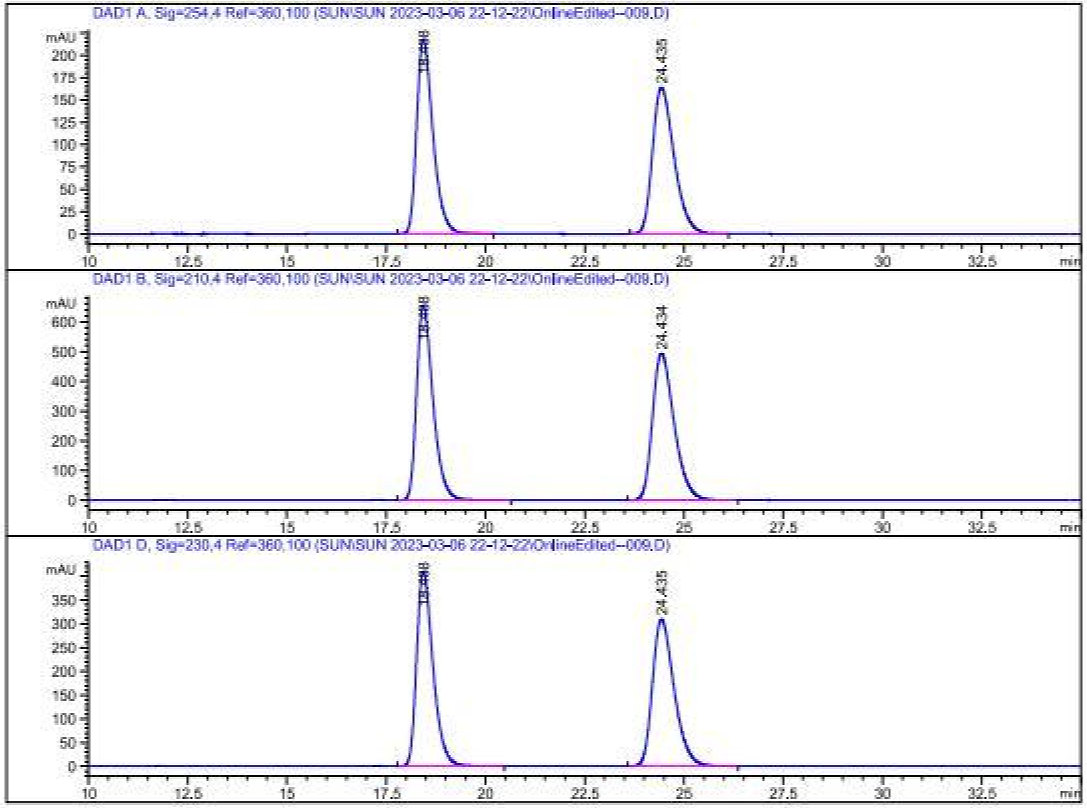

Data File C:\Users\P...\ChemStation\1\Data\SUN\SUN 2023-03-06 22-12-22\OnlineEdited--009.D
Sample Name: qcx-6-47a

===

Area Percent Report

===

Sorted By : Signal
Multiplier : 1.0000
Dilution : 1.0000
Use Multiplier & Dilution Factor with ISTDs

Signal 1: DAD1 A, Sig=254,4 Ref=360,100

Peak #	RetTime [min]	Type	Width [min]	Area [mAU*s]	Height [mAU]	Area %
1	18.438	BB	0.4340	6231.84326	218.02031	50.0845
2	24.435	BB	0.5666	6210.81006	163.91071	49.9155

Totals : 1.24427e4 381.93102

Signal 2: DAD1 B, Sig=210,4 Ref=360,100

Peak #	RetTime [min]	Type	Width [min]	Area [mAU*s]	Height [mAU]	Area %
1	18.438	BB	0.4342	1.87525e4	655.62915	50.0696
2	24.434	BB	0.5693	1.87003e4	493.24551	49.9304

Totals : 3.74528e4 1148.87466

Signal 3: DAD1 D, Sig=230,4 Ref=360,100

Peak #	RetTime [min]	Type	Width [min]	Area [mAU*s]	Height [mAU]	Area %
1	18.438	BB	0.4363	1.17344e4	410.17886	50.0364
2	24.435	BB	0.5766	1.17174e4	308.86047	49.9636

Totals : 2.34518e4 719.03934

===

*** End of Report ***

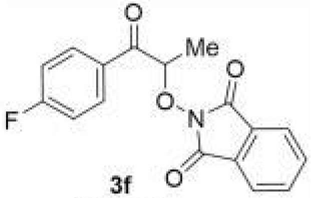

3f
racemic

Acq. Operator : SYSTEM Seq. Line : 7
Sample Operator : SYSTEM
Acq. Instrument : HPLC Location : P1-F-05
Injection Date : 5/3/2023 11:10:15 pm Inj : 1
Inj Volume : 2.000 µl
Different Inj Volume from Sample Entry! Actual Inj Volume : 3.000 µl
Acq. Method : C:\Users\Public\Documents\ChemStation\1\Data\SUN\SUN 2023-03-05 19-17-20 \IC3-30-60.M
Last changed : 10/11/2022 9:42:07 am by SYSTEM
Analysis Method : C:\Users\Public\Documents\ChemStation\1\Data\SUN\SUN 2023-03-05 19-17-20 \IC3-30-60.M (Sequence Method)
Last changed : 6/3/2023 1:35:43 pm by SYSTEM
(modified after loading)
Additional Info : Peak(s) manually integrated

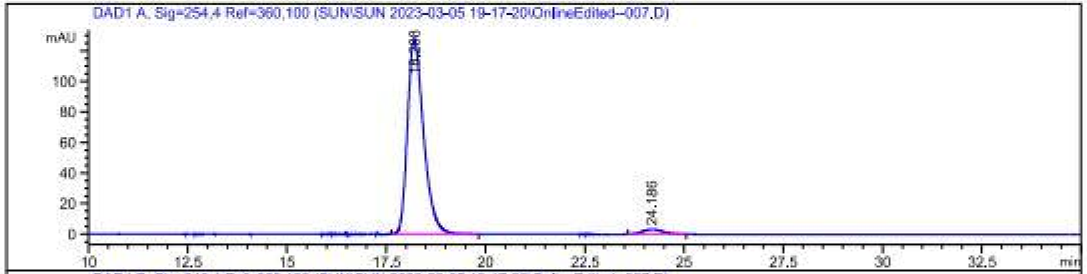

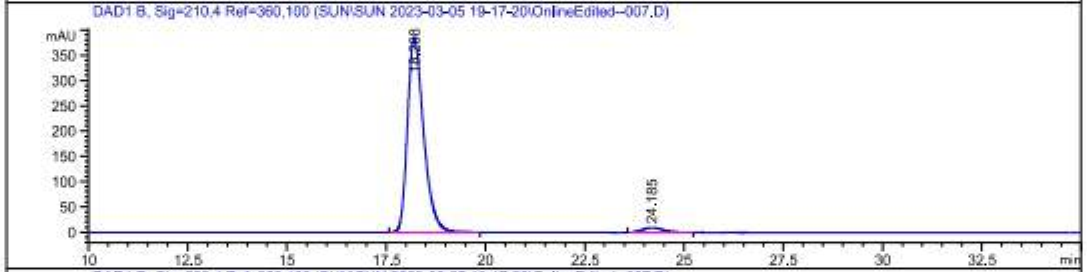

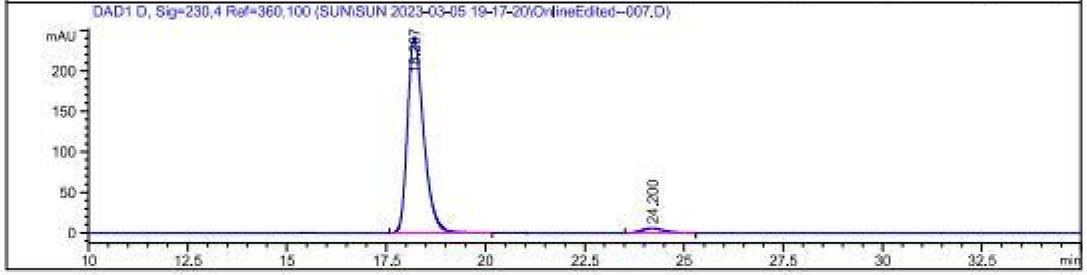

Data File C:\Users\P...\ChemStation\1\Data\SUN\SUN 2023-03-05 19-17-20\OnlineEdited--007.D
Sample Name: qcx-6-41a

Area Percent Report

Sorted By : Signal
Multiplier : 1.0000
Dilution : 1.0000
Use Multiplier & Dilution Factor with ISTDs

Signal 1: DAD1 A, Sig=254,4 Ref=360,100

Peak #	RetTime [min]	Type	Width [min]	Area [mAU*s]	Height [mAU]	Area %
1	18.208	BB	0.4310	3620.41992	127.80489	96.9010
2	24.186	BB	0.4260	115.78641	3.19385	3.0990

Totals : 3736.20633 130.99875

Signal 2: DAD1 B, Sig=210,4 Ref=360,100

Peak #	RetTime [min]	Type	Width [min]	Area [mAU*s]	Height [mAU]	Area %
1	18.208	BB	0.4319	1.09033e4	385.01703	96.8993
2	24.185	BB	0.4325	348.89804	9.47617	3.1007

Totals : 1.12522e4 394.49320

Signal 3: DAD1 D, Sig=230,4 Ref=360,100

Peak #	RetTime [min]	Type	Width [min]	Area [mAU*s]	Height [mAU]	Area %
1	18.207	BB	0.4310	6837.38916	241.02115	96.8117
2	24.200	BB	0.4402	225.17293	6.06448	3.1883

Totals : 7062.56209 247.08562

*** End of Report ***

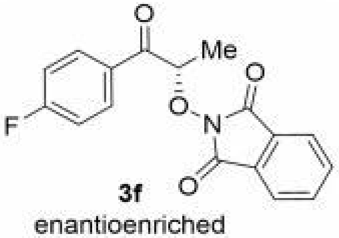

3f
enantioenriched

===

```
Acq. Operator   : SYSTEM                       Seq. Line :  70
Sample Operator : SYSTEM
Acq. Instrument : HPLC                          Location :  P1-F-06
Injection Date  : 4/3/2023 2:59:00 am                Inj :  1
                                              Inj Volume : 2.000 µl
Different Inj Volume from Sample Entry!  Actual Inj Volume : 3.000 µl
Acq. Method     : C:\Users\Public\Documents\ChemStation\1\Data\SUN\SUN 2023-03-02 14-47-18
                  \IC3-30-60.M
Last changed    : 10/11/2022 9:42:07 am by SYSTEM
Analysis Method : C:\Users\Public\Documents\ChemStation\1\Data\SUN\SUN 2023-03-02 14-47-18
                  \IC3-30-60.M (Sequence Method)
Last changed    : 6/3/2023 1:39:46 pm by SYSTEM
                  (modified after loading)
Additional Info : Peak(s) manually integrated
```

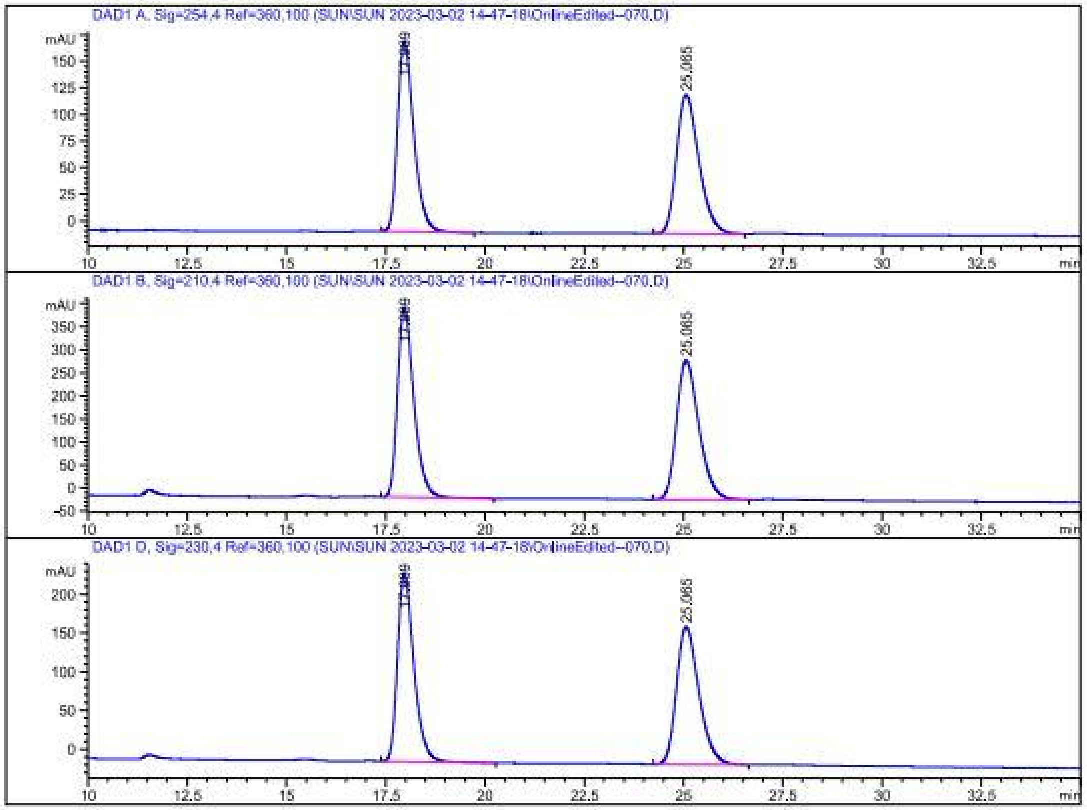

Data File C:\Users\P...\ChemStation\1\Data\SUN\SUN 2023-03-02 14-47-18\OnlineEdited--070.D
Sample Name: qcx-6-11e

===

Area Percent Report

===

```
Sorted By             :      Signal
Multiplier            :      1.0000
Dilution              :      1.0000
Use Multiplier & Dilution Factor with ISTDs
```

Signal 1: DAD1 A, Sig=254,4 Ref=360,100

Peak #	RetTime [min]	Type	Width [min]	Area [mAU*s]	Height [mAU]	Area %
1	17.969	BB	0.4324	5095.20117	179.11096	49.9686
2	25.065	BB	0.5804	5101.60547	130.41063	50.0314

Totals : 1.01968e4 309.52159

Signal 2: DAD1 B, Sig=210,4 Ref=360,100

Peak #	RetTime [min]	Type	Width [min]	Area [mAU*s]	Height [mAU]	Area %
1	17.969	BB	0.4346	1.17577e4	412.37796	49.9188
2	25.065	BB	0.6022	1.17960e4	301.02698	50.0812

Totals : 2.35537e4 713.40494

Signal 3: DAD1 D, Sig=230,4 Ref=360,100

Peak #	RetTime [min]	Type	Width [min]	Area [mAU*s]	Height [mAU]	Area %
1	17.969	BB	0.4366	6944.85107	243.25029	49.9499
2	25.065	BB	0.5997	6958.77539	177.55354	50.0501

Totals : 1.39036e4 420.80383

===

*** End of Report ***

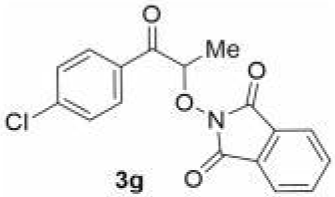

3g
racemic

===

Acq. Operator : SYSTEM Seq. Line : 5
Sample Operator : SYSTEM
Acq. Instrument : HPLC Location : P1-F-06
Injection Date : 5/3/2023 9:08:07 pm Inj : 1
Inj Volume : 2.000 µl
Different Inj Volume from Sample Entry! Actual Inj Volume : 3.000 µl
Acq. Method : C:\Users\Public\Documents\ChemStation\1\Data\SUN\SUN 2023-03-05 19-17-20\IC3-30-60.M
Last changed : 10/11/2022 9:42:07 am by SYSTEM
Analysis Method : C:\Users\Public\Documents\ChemStation\1\Data\SUN\SUN 2023-03-05 19-17-20\IC3-30-60.M (Sequence Method)
Last changed : 6/3/2023 1:35:43 pm by SYSTEM
(modified after loading)
Additional Info : Peak(s) manually integrated

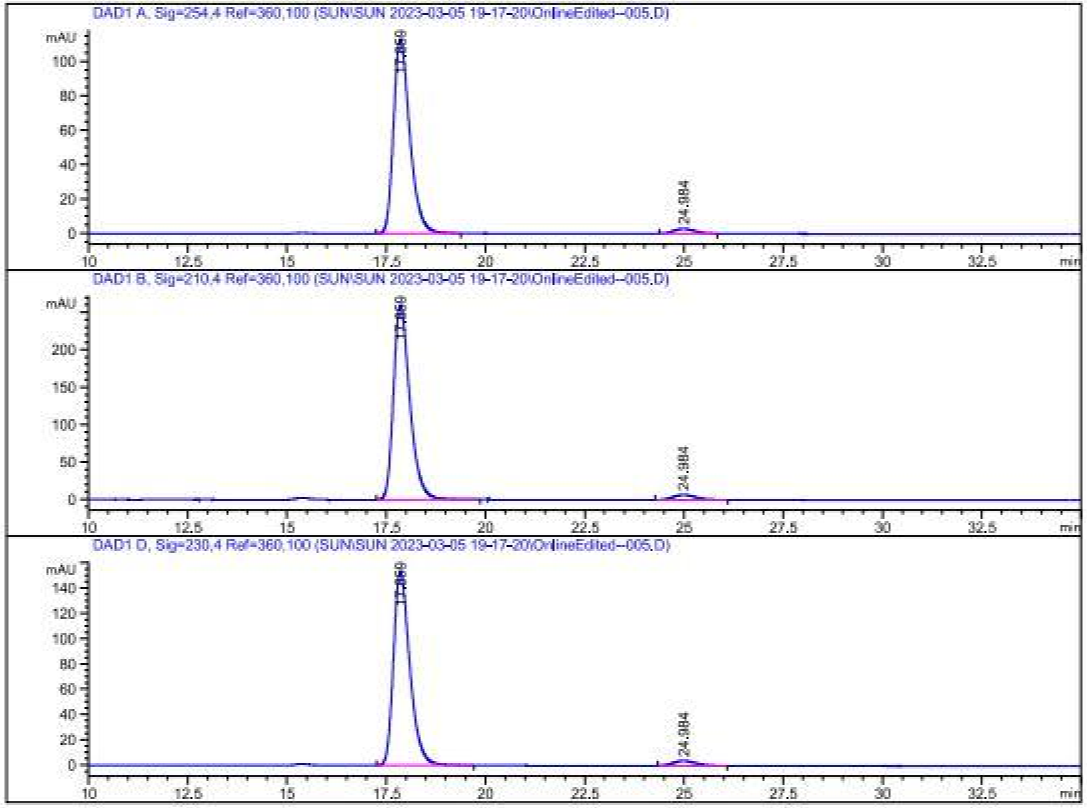

Data File C:\Users\P...\ChemStation\1\Data\SUN\SUN 2023-03-05 19-17-20\OnlineEdited--005.D
Sample Name: qcx-6-41b

===

Area Percent Report

===

Sorted By : Signal
Multiplier : 1.0000
Dilution : 1.0000
Use Multiplier & Dilution Factor with ISTDs

Signal 1: DAD1 A, Sig=254,4 Ref=360,100

Peak #	RetTime [min]	Type	Width [min]	Area [mAU*s]	Height [mAU]	Area %
1	17.859	BB	0.4335	3225.67529	113.18884	96.8285
2	24.984	BB	0.4373	105.65167	2.83846	3.1715

Totals : 3331.32697 116.02730

Signal 2: DAD1 B, Sig=210,4 Ref=360,100

Peak #	RetTime [min]	Type	Width [min]	Area [mAU*s]	Height [mAU]	Area %
1	17.859	BB	0.4338	7425.48486	259.89862	96.5734
2	24.984	BB	0.4550	263.47302	6.77878	3.4266

Totals : 7688.95789 266.67740

Signal 3: DAD1 D, Sig=230,4 Ref=360,100

Peak #	RetTime [min]	Type	Width [min]	Area [mAU*s]	Height [mAU]	Area %
1	17.859	BB	0.4346	4388.13770	153.69606	96.6785
2	24.984	BB	0.4487	150.75790	3.94162	3.3215

Totals : 4538.89560 157.63768

===

*** End of Report ***

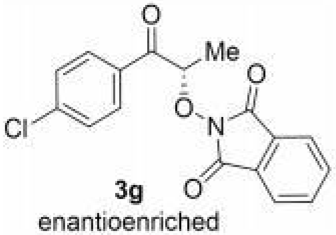

3g
enantioenriched

===

Acq. Operator : SYSTEM Seq. Line : 71
Sample Operator : SYSTEM
Acq. Instrument : HPLC Location : P1-F-07
Injection Date : 4/3/2023 4:00:06 am Inj : 1
Inj Volume : 2.000 µl
Different Inj Volume from Sample Entry! Actual Inj Volume : 3.000 µl
Acq. Method : C:\Users\Public\Documents\ChemStation\1\Data\SUN\SUN 2023-03-02 14-47-18\IC3-30-60.M
Last changed : 10/11/2022 9:42:07 am by SYSTEM
Analysis Method : C:\Users\Public\Documents\ChemStation\1\Data\SUN\SUN 2023-03-02 14-47-18\IC3-30-60.M (Sequence Method)
Last changed : 6/3/2023 1:39:46 pm by SYSTEM
(modified after loading)
Additional Info : Peak(s) manually integrated

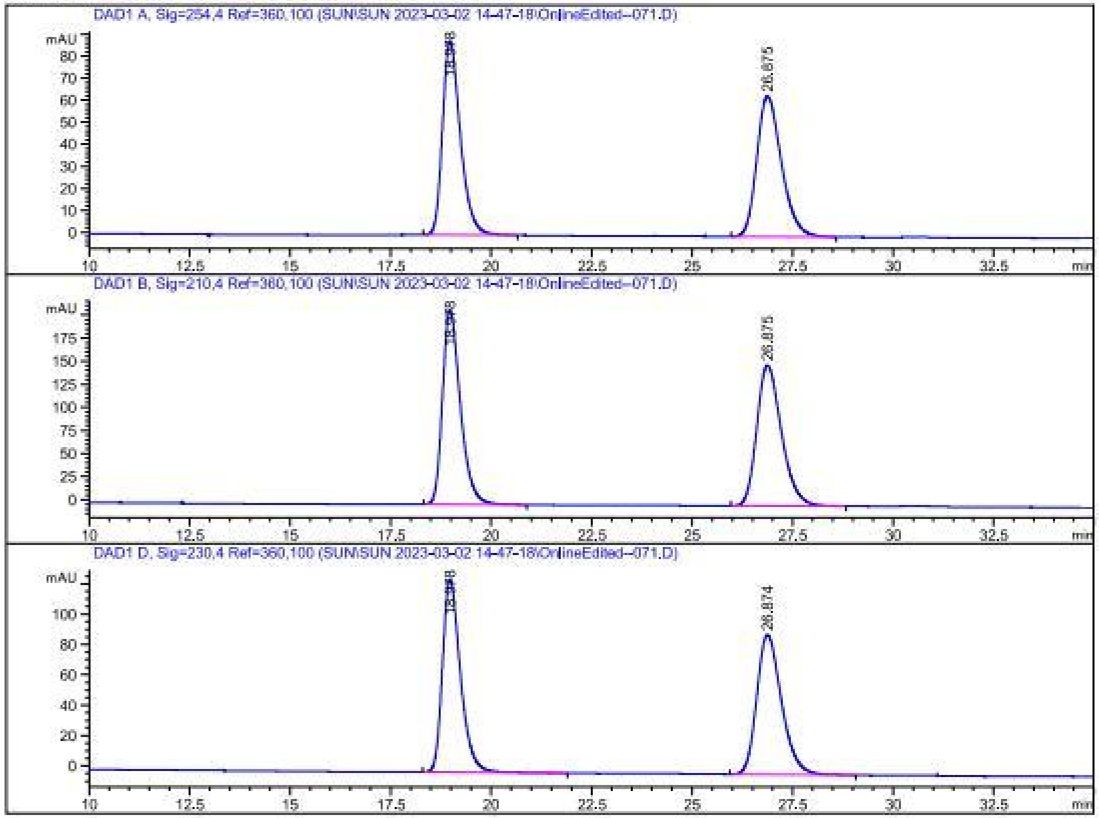

Data File C:\Users\P...\ChemStation\1\Data\SUN\SUN 2023-03-02 14-47-18\OnlineEdited--071.D
Sample Name: qcx-6-12b

===

Area Percent Report

===

Sorted By : Signal
Multiplier : 1.0000
Dilution : 1.0000
Use Multiplier & Dilution Factor with ISTDs

Signal 1: DAD1 A, Sig=254,4 Ref=360,100

Peak #	RetTime [min]	Type	Width [min]	Area [mAU*s]	Height [mAU]	Area %
1	18.978	BB	0.4611	2721.84009	88.18568	49.9437
2	26.875	BB	0.6139	2727.98193	63.92424	50.0563

Totals : 5449.82202 152.10992

Signal 2: DAD1 B, Sig=210,4 Ref=360,100

Peak #	RetTime [min]	Type	Width [min]	Area [mAU*s]	Height [mAU]	Area %
1	18.978	BB	0.4618	6499.84277	210.18872	49.9233
2	26.875	BB	0.6251	6519.80420	152.39612	50.0767

Totals : 1.30196e4 362.58484

Signal 3: DAD1 D, Sig=230,4 Ref=360,100

Peak #	RetTime [min]	Type	Width [min]	Area [mAU*s]	Height [mAU]	Area %
1	18.978	BB	0.4691	3939.93628	126.93650	49.9996
2	26.874	BB	0.6388	3940.00464	92.05877	50.0004

Totals : 7879.94092 218.99527

===

*** End of Report ***

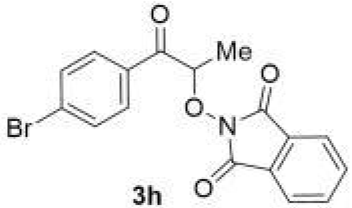

3h
racemic

```
=====================================================================
Acq. Operator   : SYSTEM                        Seq. Line :    6
Sample Operator : SYSTEM
Acq. Instrument : HPLC                           Location :   P1-F-07
Injection Date  : 5/3/2023 10:09:10 pm                Inj :    1
                                               Inj Volume : 2.000 µl
Different Inj Volume from Sample Entry!  Actual Inj Volume : 3.000 µl
Acq. Method     : C:\Users\Public\Documents\ChemStation\1\Data\SUN\SUN 2023-03-05 19-17-20
                  \IC3-30-60.M
Last changed    : 10/11/2022 9:42:07 am by SYSTEM
Analysis Method : C:\Users\Public\Documents\ChemStation\1\Data\SUN\SUN 2023-03-05 19-17-20
                  \IC3-30-60.M (Sequence Method)
Last changed    : 6/3/2023 1:35:43 pm by SYSTEM
                  (modified after loading)
Additional Info : Peak(s) manually integrated
```

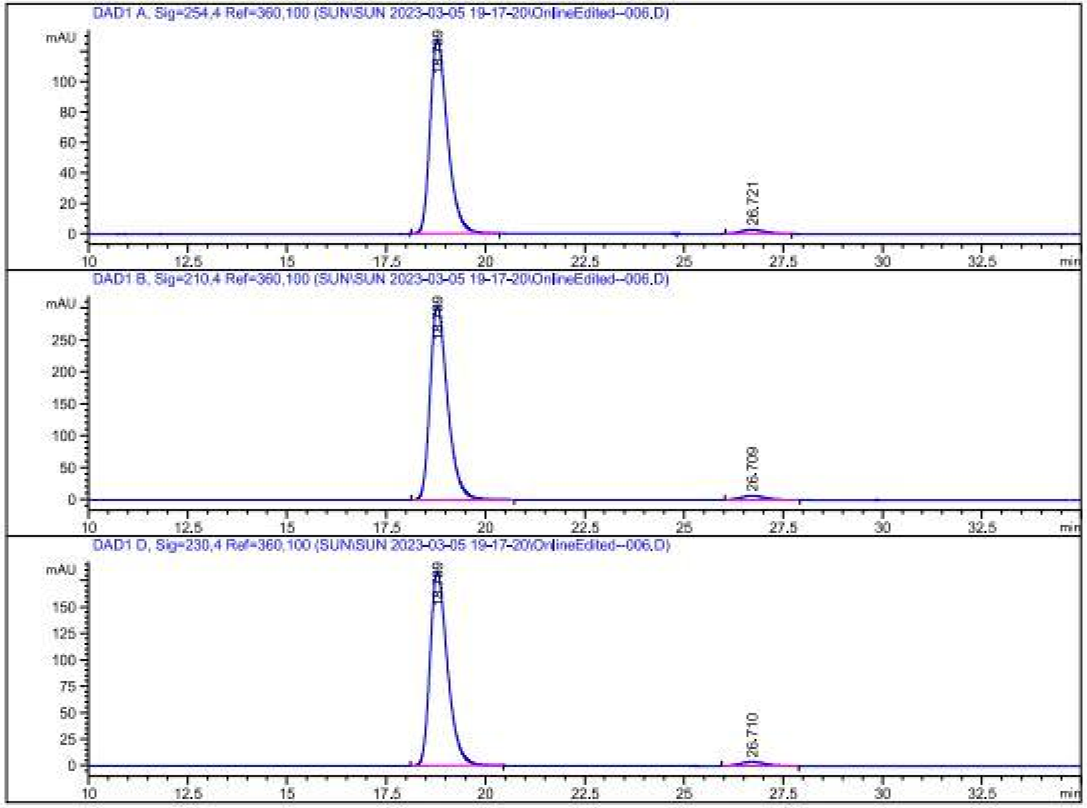

Data File C:\Users\P...\ChemStation\1\Data\SUN\SUN 2023-03-05 19-17-20\OnlineEdited--006.D
Sample Name: qcx-6-41c

===
Area Percent Report
===

```
Sorted By             :      Signal
Multiplier            :      1.0000
Dilution              :      1.0000
Use Multiplier & Dilution Factor with ISTDs
```

Signal 1: DAD1 A, Sig=254,4 Ref=360,100

Peak #	RetTime [min]	Type	Width [min]	Area [mAU*s]	Height [mAU]	Area %
1	18.789	BB	0.4584	3901.63770	128.07159	97.2342
2	26.721	BB	0.4803	110.98286	2.70427	2.7658

Totals : 4012.62056 130.77587

Signal 2: DAD1 B, Sig=210,4 Ref=360,100

Peak #	RetTime [min]	Type	Width [min]	Area [mAU*s]	Height [mAU]	Area %
1	18.789	BB	0.4631	9299.95898	304.71368	97.1476
2	26.709	BB	0.4926	273.05978	6.50690	2.8524

Totals : 9573.01877 311.22059

Signal 3: DAD1 D, Sig=230,4 Ref=360,100

Peak #	RetTime [min]	Type	Width [min]	Area [mAU*s]	Height [mAU]	Area %
1	18.789	BB	0.4608	5612.72559	184.02553	97.0970
2	26.710	BB	0.4956	167.81119	3.97448	2.9030

Totals : 5780.53677 188.00001

===
*** End of Report ***

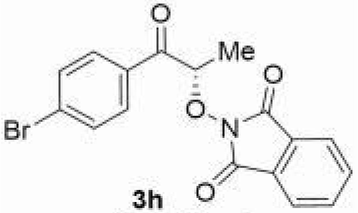

3h
enantioenriched

Acq. Operator : SYSTEM Seq. Line : 39
Sample Operator : SYSTEM
Acq. Instrument : HPLC Location : P1-F-03
Injection Date : 8/3/2023 6:55:11 am Inj : 1
Inj Volume : 2.000 µl
Different Inj Volume from Sample Entry! Actual Inj Volume : 15.000 µl
Acq. Method : C:\Users\Public\Documents\ChemStation\1\Data\SUN\SUN 2023-03-07 15-14-32\IC3-30-60.M
Last changed : 10/11/2022 9:42:07 am by SYSTEM
Analysis Method : C:\Users\Public\Documents\ChemStation\1\Data\SUN\SUN 2023-03-07 15-14-32\IC3-30-60.M (Sequence Method)
Last changed : 8/3/2023 1:15:39 pm by SYSTEM
(modified after loading)
Additional Info : Peak(s) manually integrated

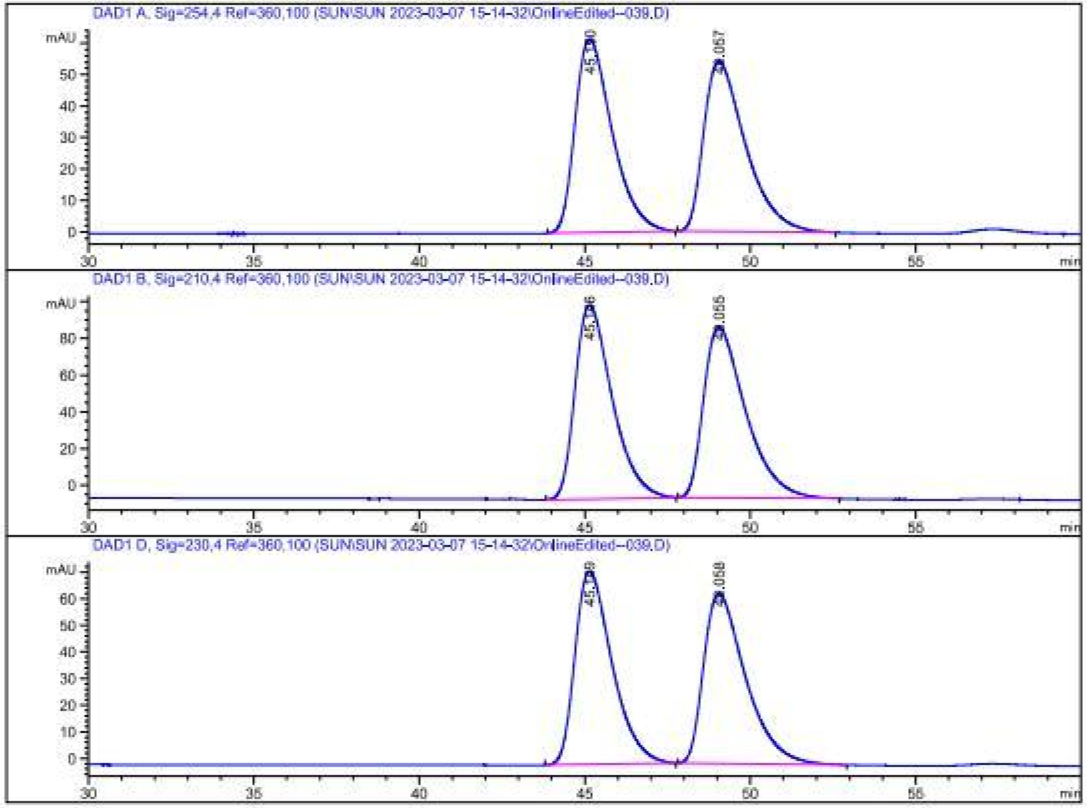

Data File C:\Users\P...\ChemStation\1\Data\SUN\SUN 2023-03-07 15-14-32\OnlineEdited--039.D
Sample Name: qcx-6-47b-2

Area Percent Report

Sorted By : Signal
Multiplier : 1.0000
Dilution : 1.0000
Use Multiplier & Dilution Factor with ISTDs

Signal 1: DAD1 A, Sig=254,4 Ref=360,100

Peak #	RetTime [min]	Type	Width [min]	Area [mAU*s]	Height [mAU]	Area %
1	45.150	BB	0.9129	4794.28711	61.47468	50.1827
2	49.057	BB	1.0328	4759.37256	54.02447	49.8173

Totals : 9553.65967 115.49915

Signal 2: DAD1 B, Sig=210,4 Ref=360,100

Peak #	RetTime [min]	Type	Width [min]	Area [mAU*s]	Height [mAU]	Area %
1	45.146	BB	0.9243	8222.77246	105.49851	50.0836
2	49.055	BB	1.0474	8195.30566	92.92587	49.9164

Totals : 1.64181e4 198.42438

Signal 3: DAD1 D, Sig=230,4 Ref=360,100

Peak #	RetTime [min]	Type	Width [min]	Area [mAU*s]	Height [mAU]	Area %
1	45.149	BB	0.9131	5657.91455	72.56789	50.1352
2	49.058	BB	1.0315	5627.40771	63.80194	49.8648

Totals : 1.12853e4 136.36983

*** End of Report ***

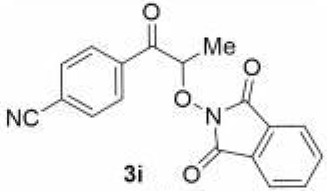

3i
racemic

Acq. Operator : SYSTEM Seq. Line : 4
Sample Operator : SYSTEM
Acq. Instrument : HPLC Location : P1-F-10
Injection Date : 7/3/2023 4:08:35 pm Inj : 1
Inj Volume : 2.000 µl
Different Inj Volume from Sample Entry! Actual Inj Volume : 3.000 µl
Acq. Method : C:\Users\Public\Documents\ChemStation\1\Data\SUN\SUN 2023-03-07 15-14-32\IC3-30-60.M
Last changed : 10/11/2022 9:42:07 am by SYSTEM
Analysis Method : C:\Users\Public\Documents\ChemStation\1\Data\SUN\SUN 2023-03-07 15-14-32\IC3-30-60.M (Sequence Method)
Last changed : 8/3/2023 1:20:48 pm by SYSTEM
(modified after loading)
Additional Info : Peak(s) manually integrated

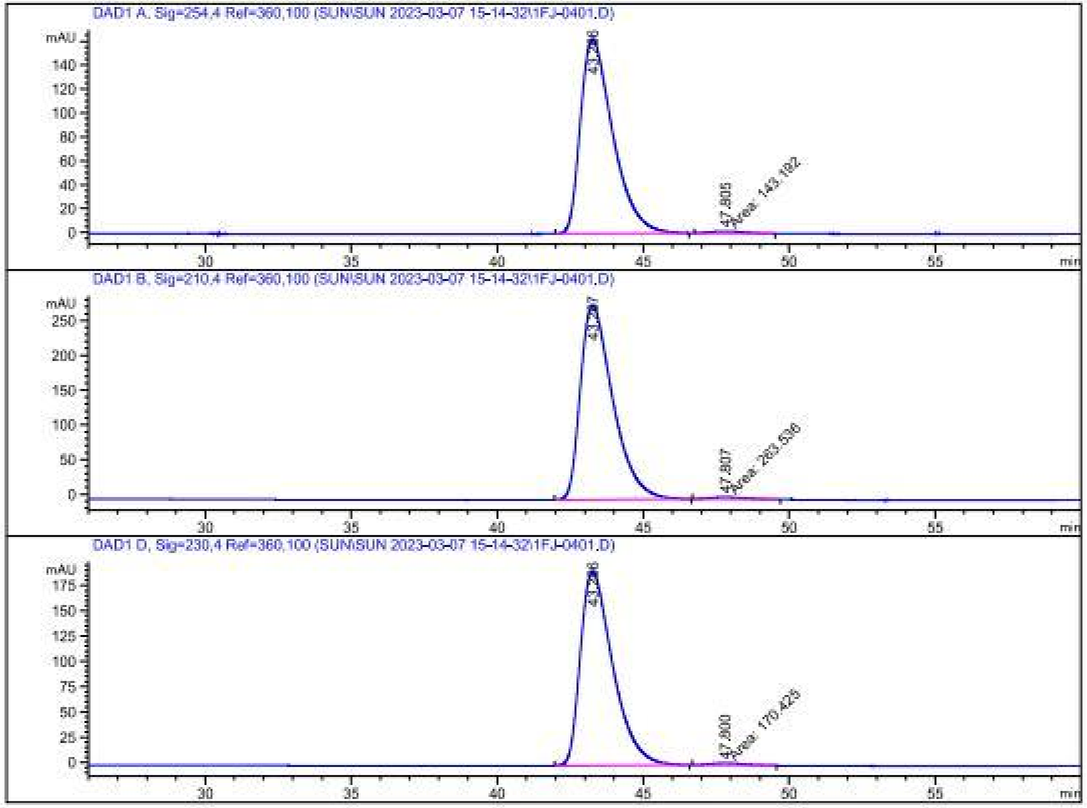

Data File C:\Users\P...Documents\ChemStation\1\Data\SUN\SUN 2023-03-07 15-14-32\1FJ-0401.D
Sample Name: qcx-6-41d

Area Percent Report

Sorted By : Signal
Multiplier : 1.0000
Dilution : 1.0000
Use Multiplier & Dilution Factor with ISTDs

Signal 1: DAD1 A, Sig=254,4 Ref=360,100

Peak #	RetTime [min]	Type	Width [min]	Area [mAU*s]	Height [mAU]	Area %
1	43.266	BB	1.0548	1.25474e4	162.68942	98.8717
2	47.805	MM	1.3510	143.19194	1.76653	1.1283

Totals : 1.26906e4 164.45595

Signal 2: DAD1 B, Sig=210,4 Ref=360,100

Peak #	RetTime [min]	Type	Width [min]	Area [mAU*s]	Height [mAU]	Area %
1	43.267	BB	1.0608	2.15745e4	279.73993	98.7932
2	47.807	MM	1.4014	263.53555	3.13427	1.2068

Totals : 2.18380e4 282.87420

Signal 3: DAD1 D, Sig=230,4 Ref=360,100

Peak #	RetTime [min]	Type	Width [min]	Area [mAU*s]	Height [mAU]	Area %
1	43.266	BB	1.0961	1.48027e4	192.07928	98.8618
2	47.800	MM	1.3610	170.42494	2.08699	1.1382

Totals : 1.49731e4 194.16628

*** End of Report ***

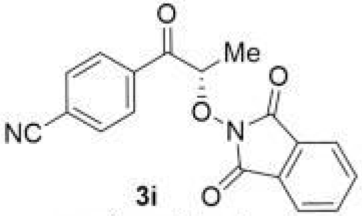

3i
enantioenriched

===

```
Acq. Operator   : SYSTEM                     Seq. Line :  11
Sample Operator : SYSTEM
Acq. Instrument : HPLC                        Location :   P1-F-05
Injection Date  : 13/3/2023 9:03:51 pm             Inj :   1
                                           Inj Volume : 2.000 µl
Different Inj Volume from Sample Entry!  Actual Inj Volume : 25.000 µl
Acq. Method     : C:\Users\Public\Documents\ChemStation\1\Data\SUN\SUN 2023-03-13 17-26-17
                  \IC3-50-60.M
Last changed    : 12/2/2023 2:10:14 pm by SYSTEM
Analysis Method : C:\Users\Public\Documents\ChemStation\1\Data\SUN\SUN 2023-03-13 17-26-17
                  \IC3-50-60.M (Sequence Method)
Last changed    : 16/3/2023 6:06:35 pm by SYSTEM
                  (modified after loading)
Additional Info : Peak(s) manually integrated
```

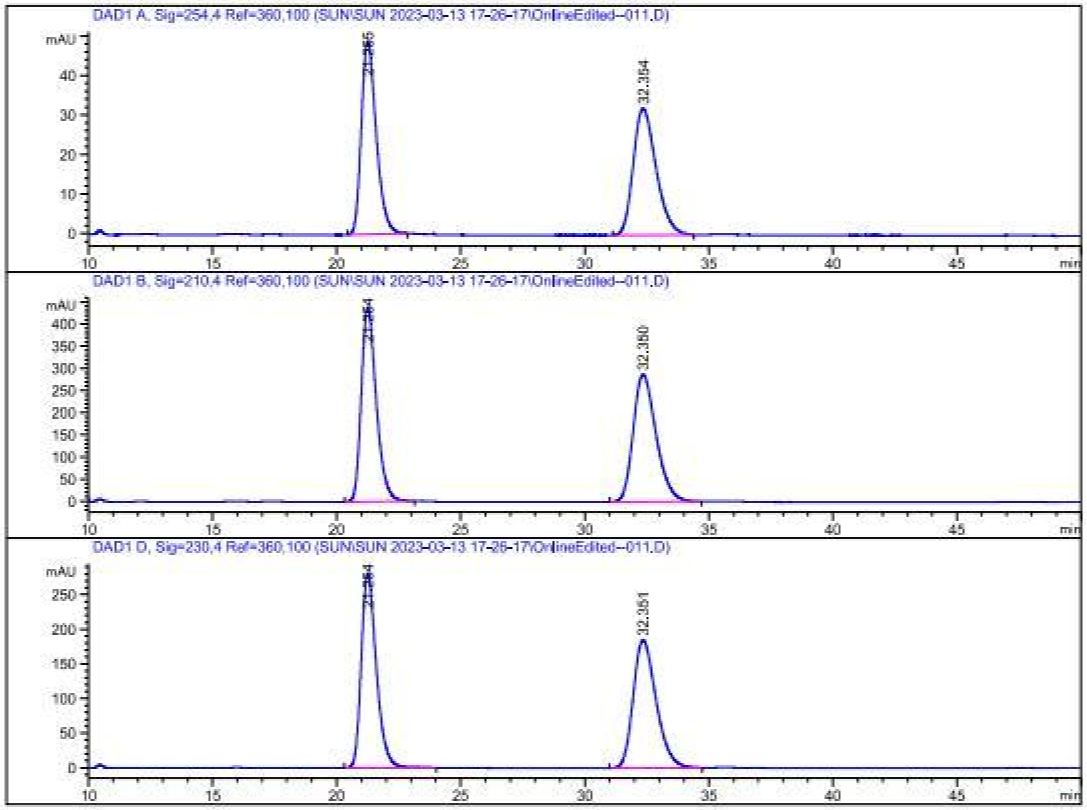

Data File C:\Users\P...\ChemStation\1\Data\SUN\SUN 2023-03-13 17-26-17\OnlineEdited--011.D
Sample Name: qcx-6-56f-2

===

Area Percent Report

===

```
Sorted By             :      Signal
Multiplier            :      1.0000
Dilution              :      1.0000
Use Multiplier & Dilution Factor with ISTDs
```

Signal 1: DAD1 A, Sig=254,4 Ref=360,100

Peak #	RetTime [min]	Type	Width [min]	Area [mAU*s]	Height [mAU]	Area %
1	21.255	BB	0.5417	2046.55212	48.90737	49.9782
2	32.354	BB	0.7550	2048.33350	32.01363	50.0218
Totals :				4094.88562	80.92100	

Signal 2: DAD1 B, Sig=210,4 Ref=360,100

Peak #	RetTime [min]	Type	Width [min]	Area [mAU*s]	Height [mAU]	Area %
1	21.254	BB	0.6299	1.84070e4	438.82529	49.9815
2	32.350	BB	0.8482	1.84206e4	287.21878	50.0185
Totals :				3.68276e4	726.04407	

Signal 3: DAD1 D, Sig=230,4 Ref=360,100

Peak #	RetTime [min]	Type	Width [min]	Area [mAU*s]	Height [mAU]	Area %
1	21.254	BB	0.6483	1.19392e4	281.52731	50.1248
2	32.351	BB	0.9368	1.18797e4	184.72586	49.8752
Totals :				2.38189e4	466.25317	

===

*** End of Report ***

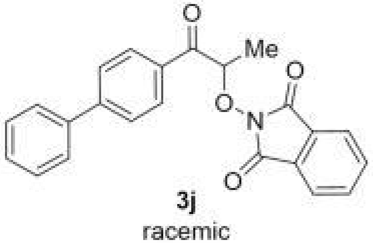

3j
racemic

===

Acq. Operator : SYSTEM   Seq. Line : 63
Sample Operator : SYSTEM
Acq. Instrument : HPLC   Location : P1-F-04
Injection Date : 17/3/2023 1:22:34 am   Inj : 1
Inj Volume : 2.000 µl
Different Inj Volume from Sample Entry! Actual Inj Volume : 3.000 µl
Acq. Method : C:\Users\Public\Documents\ChemStation\1\Data\SUN\SUN 2023-03-15 22-36-05\IC3-50-60.M
Last changed : 12/2/2023 2:10:14 pm by SYSTEM
Analysis Method : C:\Users\Public\Documents\ChemStation\1\Data\SUN\SUN 2023-03-15 22-36-05\IC3-50-60.M (Sequence Method)
Last changed : 17/3/2023 1:40:56 pm by SYSTEM
(modified after loading)
Additional Info : Peak(s) manually integrated

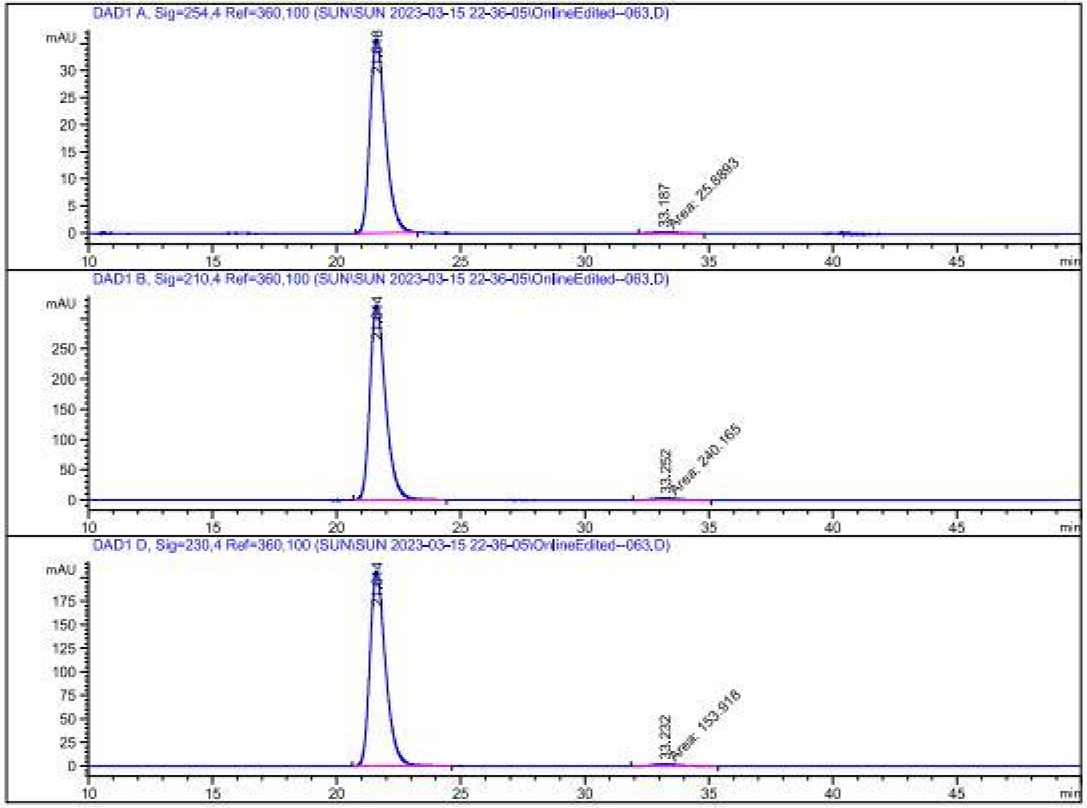

Data File C:\Users\P...\ChemStation\1\Data\SUN\SUN 2023-03-15 22-36-05\OnlineEdited--063.D
Sample Name: qcx-6-56b

===

Area Percent Report

===

Sorted By : Signal
Multiplier : 1.0000
Dilution : 1.0000
Use Multiplier & Dilution Factor with ISTDs

Signal 1: DAD1 A, Sig=254,4 Ref=360,100

Peak #	RetTime [min]	Type	Width [min]	Area [mAU*s]	Height [mAU]	Area %
1	21.618	BB	0.5839	1562.47681	35.89071	98.3701
2	33.187	MM	1.1345	25.88934	3.80327e-1	1.6299

Totals : 1588.36615 36.27103

Signal 2: DAD1 B, Sig=210,4 Ref=360,100

Peak #	RetTime [min]	Type	Width [min]	Area [mAU*s]	Height [mAU]	Area %
1	21.614	BB	0.6600	1.41570e4	322.24496	98.3319
2	33.252	MM	1.1755	240.16466	3.40525	1.6681

Totals : 1.43971e4 325.65022

Signal 3: DAD1 D, Sig=230,4 Ref=360,100

Peak #	RetTime [min]	Type	Width [min]	Area [mAU*s]	Height [mAU]	Area %
1	21.614	BB	0.6647	9115.45508	206.82506	98.3395
2	33.232	MM	1.1780	153.91779	2.17772	1.6605

Totals : 9269.37286 209.00278

===

*** End of Report ***

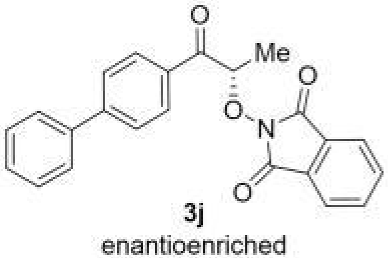

3j
enantioenriched

```
=====================================================================
Acq. Operator   : SYSTEM                       Seq. Line :  59
Sample Operator : SYSTEM
Acq. Instrument : HPLC                          Location :   P1-F-06
Injection Date  : 12/3/2023 4:33:55 pm               Inj :   1
                                             Inj Volume : 2.000 µl
Different Inj Volume from Sample Entry!  Actual Inj Volume : 3.000 µl
Acq. Method     : C:\Users\Public\Documents\ChemStation\1\Data\SUN\SUN 2023-03-11 14-02-31
                  \IC3-30-60.M
Last changed    : 10/11/2022 9:42:07 am by SYSTEM
Analysis Method : C:\Users\Public\Documents\ChemStation\1\Data\SUN\SUN 2023-03-11 14-02-31
                  \IC3-30-60.M (Sequence Method)
Last changed    : 13/3/2023 7:40:48 pm by SYSTEM
                  (modified after loading)
Additional Info : Peak(s) manually integrated
```

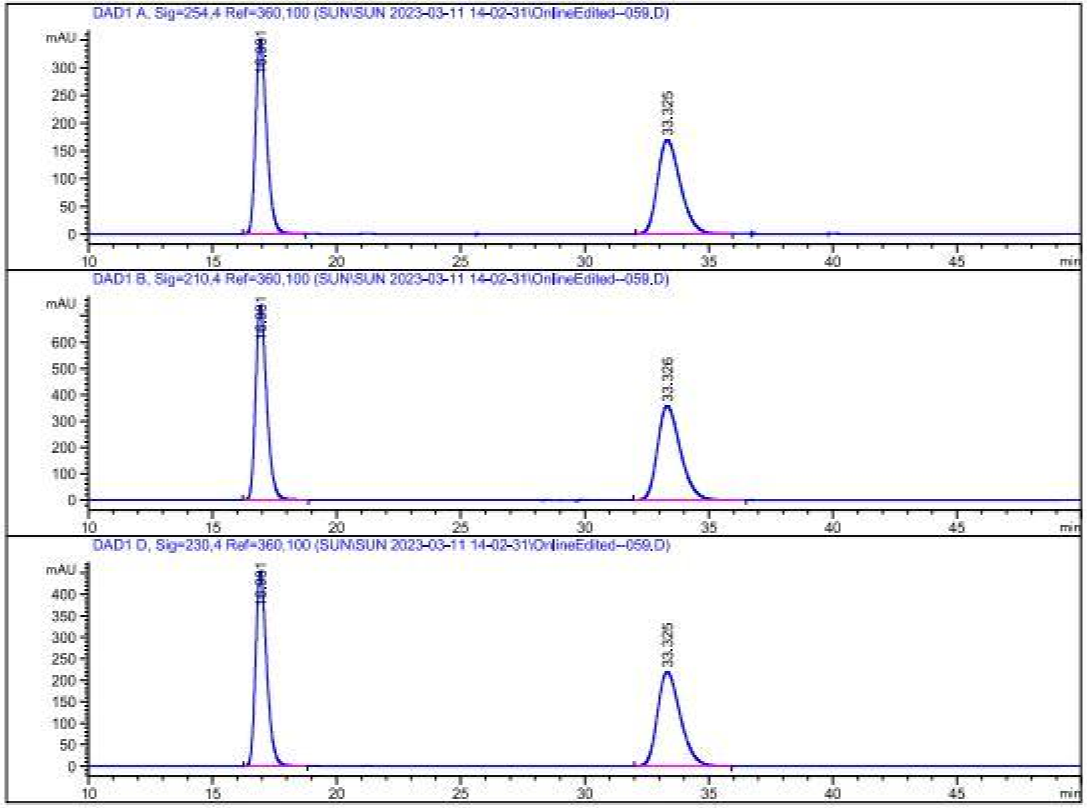

Data File C:\Users\P...\ChemStation\1\Data\SUN\SUN 2023-03-11 14-02-31\OnlineEdited--059.D
Sample Name: qcx-6-56g

```
=====================================================================
                         Area Percent Report
=====================================================================

Sorted By             :      Signal
Multiplier            :      1.0000
Dilution              :      1.0000
Use Multiplier & Dilution Factor with ISTDs
```

Signal 1: DAD1 A, Sig=254,4 Ref=360,100

Peak #	RetTime [min]	Type	Width [min]	Area [mAU*s]	Height [mAU]	Area %
1	16.931	BB	0.4693	1.08053e4	351.32669	49.9838
2	33.325	BB	0.9123	1.08123e4	169.47864	50.0162

Totals : 2.16176e4 520.80533

Signal 2: DAD1 B, Sig=210,4 Ref=360,100

Peak #	RetTime [min]	Type	Width [min]	Area [mAU*s]	Height [mAU]	Area %
1	16.931	BB	0.4725	2.27708e4	739.96222	49.9306
2	33.326	BB	0.9396	2.28341e4	357.31244	50.0694

Totals : 4.56049e4 1097.27466

Signal 3: DAD1 D, Sig=230,4 Ref=360,100

Peak #	RetTime [min]	Type	Width [min]	Area [mAU*s]	Height [mAU]	Area %
1	16.931	BB	0.4734	1.39763e4	453.67584	49.9414
2	33.325	BB	0.9291	1.40091e4	219.51077	50.0586

Totals : 2.79854e4 673.18661

===

*** End of Report ***

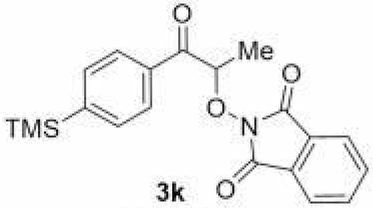

3k
racemic

Acq. Operator : SYSTEM Seq. Line : 65
Sample Operator : SYSTEM
Acq. Instrument : HPLC Location : P2-A-08
Injection Date : 17/3/2023 2:34:41 am Inj : 1
Inj Volume : 2.000 µl
Different Inj Volume from Sample Entry! Actual Inj Volume : 8.000 µl
Acq. Method : C:\Users\Public\Documents\ChemStation\1\Data\SUN\SUN 2023-03-15 22-36-05\IC3-30-60.M
Last changed : 10/11/2022 9:42:07 am by SYSTEM
Analysis Method : C:\Users\Public\Documents\ChemStation\1\Data\SUN\SUN 2023-03-15 22-36-05\IC3-30-60.M (Sequence Method)
Last changed : 17/3/2023 1:41:37 pm by SYSTEM
(modified after loading)
Additional Info : Peak(s) manually integrated

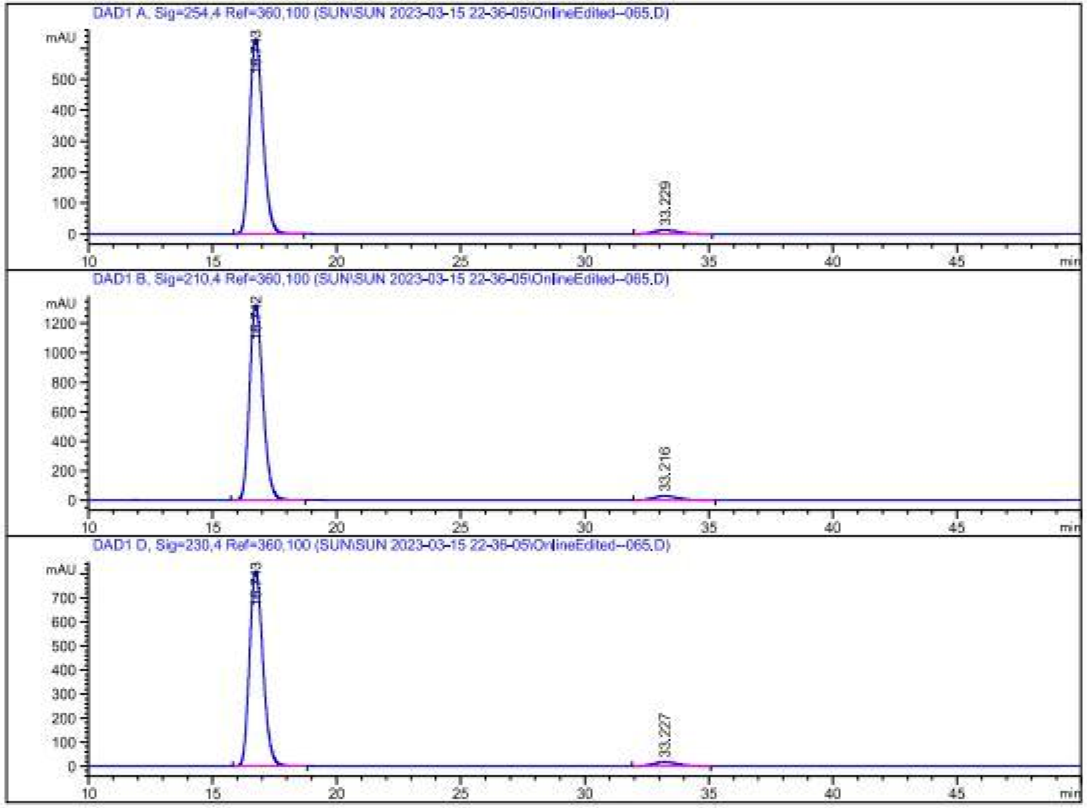

Data File C:\Users\P...\ChemStation\1\Data\SUN\SUN 2023-03-15 22-36-05\OnlineEdited--065.D
Sample Name: qcx-6-56c

Area Percent Report

Sorted By : Signal
Multiplier : 1.0000
Dilution : 1.0000
Use Multiplier & Dilution Factor with ISTDs

Signal 1: DAD1 A, Sig=254,4 Ref=360,100

Peak #	RetTime [min]	Type	Width [min]	Area [mAU*s]	Height [mAU]	Area %
1	16.743	BB	0.5825	2.35838e4	630.27155	96.3400
2	33.229	BB	0.7723	895.94946	13.59633	3.6600

Totals : 2.44797e4 643.86787

Signal 2: DAD1 B, Sig=210,4 Ref=360,100

Peak #	RetTime [min]	Type	Width [min]	Area [mAU*s]	Height [mAU]	Area %
1	16.742	BB	0.5778	4.96250e4	1323.99316	96.3214
2	33.216	BB	0.7768	1895.21375	28.64955	3.6786

Totals : 5.15202e4 1352.64271

Signal 3: DAD1 D, Sig=230,4 Ref=360,100

Peak #	RetTime [min]	Type	Width [min]	Area [mAU*s]	Height [mAU]	Area %
1	16.743	BB	0.5839	3.04140e4	810.38721	96.3225
2	33.227	BB	0.7715	1161.18787	17.62908	3.6775

Totals : 3.15752e4 828.01629

*** End of Report ***

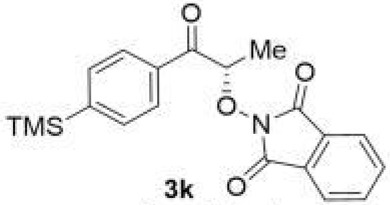

3k
enantioenriched

```
=====================================================================
Acq. Operator   : SYSTEM                    Seq. Line :  74
Sample Operator : SYSTEM
Acq. Instrument : HPLC                       Location :  P1-C-10
Injection Date  : 4/3/2023 6:43:23 am             Inj :  1
                                           Inj Volume : 2.000 µl
Different Inj Volume from Sample Entry!  Actual Inj Volume : 3.000 µl
Acq. Method     : C:\Users\Public\Documents\ChemStation\1\Data\SUN\SUN 2023-03-02 14-47-18
                  \IC3-30-60.M
Last changed    : 10/11/2022 9:42:07 am by SYSTEM
Analysis Method : C:\Users\Public\Documents\ChemStation\1\Data\SUN\SUN 2023-03-02 14-47-18
                  \IC3-30-60.M (Sequence Method)
Last changed    : 6/3/2023 1:49:19 pm by SYSTEM
                  (modified after loading)
Additional Info : Peak(s) manually integrated
```

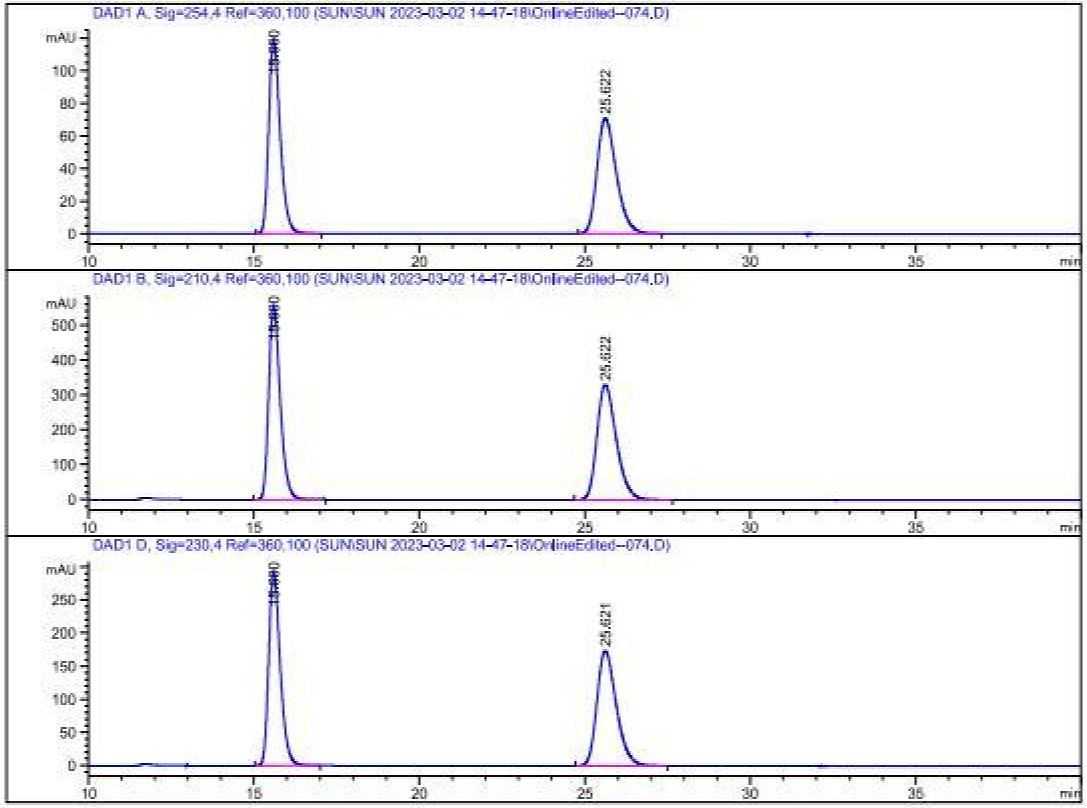

Data File C:\Users\P...\ChemStation\1\Data\SUN\SUN 2023-03-02 14-47-18\OnlineEdited--074.D
Sample Name: qcx-6-32c

Area Percent Report

```
Sorted By             :      Signal
Multiplier            :      1.0000
Dilution              :      1.0000
Use Multiplier & Dilution Factor with ISTDs
```

Signal 1: DAD1 A, Sig=254,4 Ref=360,100

Peak #	RetTime [min]	Type	Width [min]	Area [mAU*s]	Height [mAU]	Area %
1	15.600	BB	0.3661	2875.51538	119.54694	49.9593
2	25.622	BB	0.6042	2880.20239	70.78899	50.0407
Totals :				5755.71777	190.33593	

Signal 2: DAD1 B, Sig=210,4 Ref=360,100

Peak #	RetTime [min]	Type	Width [min]	Area [mAU*s]	Height [mAU]	Area %
1	15.600	BB	0.3662	1.33837e4	557.26147	49.9435
2	25.622	BB	0.6124	1.34140e4	329.86823	50.0565
Totals :				2.67977e4	887.12970	

Signal 3: DAD1 D, Sig=230,4 Ref=360,100

Peak #	RetTime [min]	Type	Width [min]	Area [mAU*s]	Height [mAU]	Area %
1	15.600	BB	0.3661	7045.32471	293.42111	49.9325
2	25.621	BB	0.6049	7064.36914	173.71695	50.0675
Totals :				1.41097e4	467.13806	

*** End of Report ***

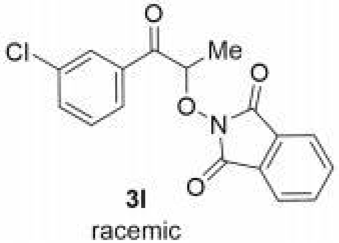

3l
racemic

===

Acq. Operator	: SYSTEM	Seq. Line :	201
Sample Operator	: SYSTEM		
Acq. Instrument	: HPLC	Location :	P2-B-03
Injection Date	: 11/3/2023 11:11:09 am	Inj :	1
		Inj Volume :	2.000 µl

Different Inj Volume from Sample Entry! Actual Inj Volume : 5.000 µl

Acq. Method : C:\Users\Public\Documents\ChemStation\1\Data\SUN\SUN 2023-03-07 15-14-32\IC3-30-40.M
Last changed : 27/2/2023 10:13:57 pm by SYSTEM
Analysis Method : C:\Users\Public\Documents\ChemStation\1\Data\SUN\SUN 2023-03-07 15-14-32\IC3-30-40.M (Sequence Method)
Last changed : 11/3/2023 7:28:27 pm by SYSTEM
(modified after loading)
Additional Info : Peak(s) manually integrated

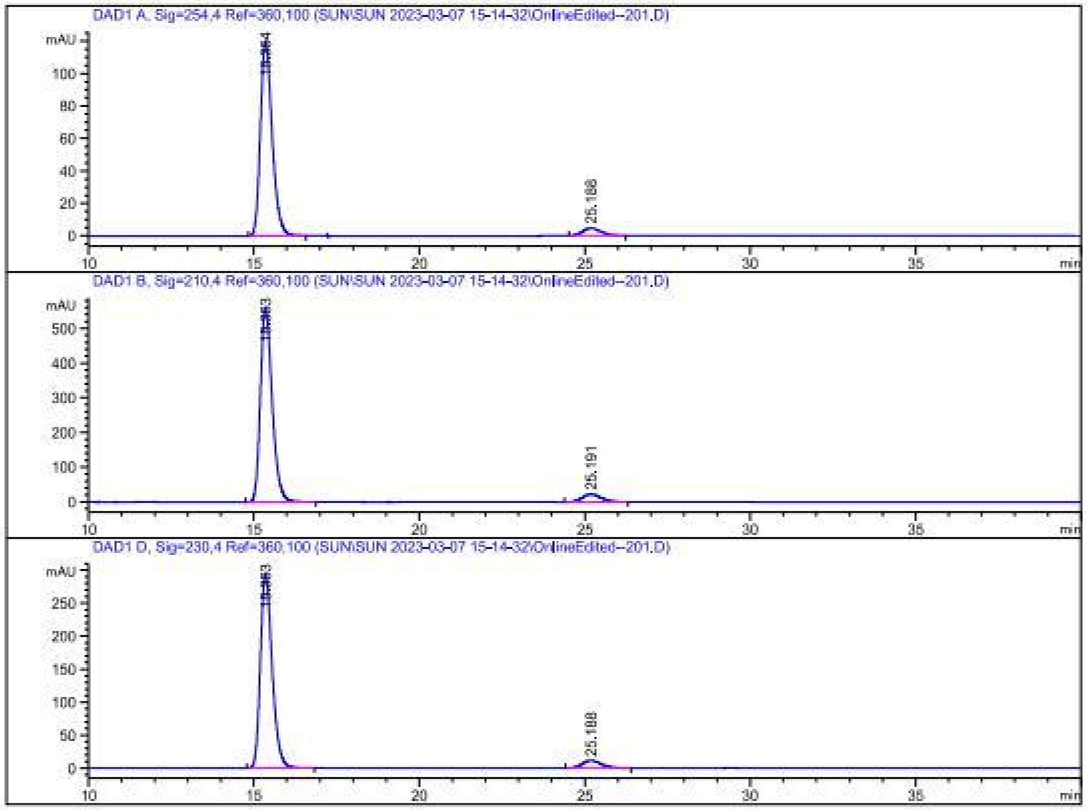

Data File C:\Users\P...\ChemStation\1\Data\SUN\SUN 2023-03-07 15-14-32\OnlineEdited--201.D
Sample Name: qcx-6-50c

===

Area Percent Report

===

Sorted By : Signal
Multiplier : 1.0000
Dilution : 1.0000
Use Multiplier & Dilution Factor with ISTDs

Signal 1: DAD1 A, Sig=254,4 Ref=360,100

Peak #	RetTime [min]	Type	Width [min]	Area [mAU*s]	Height [mAU]	Area %
1	15.354	BB	0.3715	2958.85449	120.08479	94.1280
2	25.188	BB	0.4623	184.58345	4.69919	5.8720

Totals : 3143.43794 124.78397

Signal 2: DAD1 B, Sig=210,4 Ref=360,100

Peak #	RetTime [min]	Type	Width [min]	Area [mAU*s]	Height [mAU]	Area %
1	15.353	BB	0.3742	1.38586e4	562.01129	93.9986
2	25.191	BB	0.4747	884.81879	22.21616	6.0014

Totals : 1.47434e4 584.22746

Signal 3: DAD1 D, Sig=230,4 Ref=360,100

Peak #	RetTime [min]	Type	Width [min]	Area [mAU*s]	Height [mAU]	Area %
1	15.353	BB	0.3777	7284.77393	295.37161	94.0061
2	25.188	BB	0.4834	464.47925	11.65519	5.9939

Totals : 7749.25317 307.02680

===

*** End of Report ***

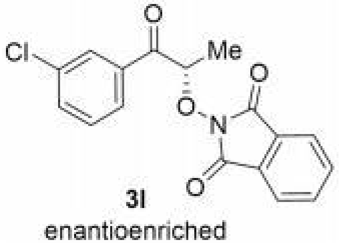

3l
enantioenriched

Acq. Operator : SYSTEM Seq. Line : 78
Sample Operator : SYSTEM
Acq. Instrument : HPLC Location : P1-C-08
Injection Date : 4/3/2023 9:52:53 am Inj : 1
Inj Volume : 2.000 µl
Different Inj Volume from Sample Entry! Actual Inj Volume : 3.000 µl
Acq. Method : C:\Users\Public\Documents\ChemStation\1\Data\SUN\SUN 2023-03-02 14-47-18\IC3-50-60.M
Last changed : 12/2/2023 2:10:14 pm by SYSTEM
Analysis Method : C:\Users\Public\Documents\ChemStation\1\Data\SUN\SUN 2023-03-02 14-47-18\IC3-50-60.M (Sequence Method)
Last changed : 6/3/2023 1:47:26 pm by SYSTEM
(modified after loading)
Additional Info : Peak(s) manually integrated

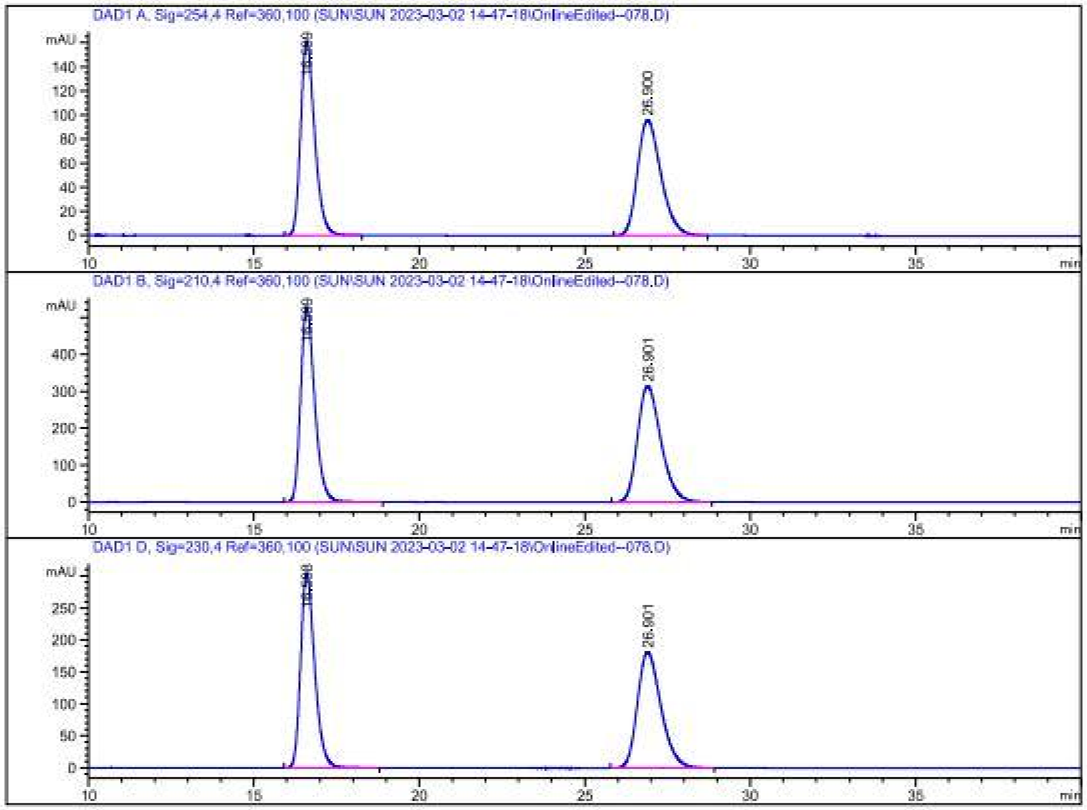

Data File C:\Users\P...\ChemStation\1\Data\SUN\SUN 2023-03-02 14-47-18\OnlineEdited--078.D
Sample Name: qcx-6-32a

Area Percent Report

Sorted By : Signal
Multiplier : 1.0000
Dilution : 1.0000
Use Multiplier & Dilution Factor with ISTDs

Signal 1: DAD1 A, Sig=254,4 Ref=360,100

Peak #	RetTime [min]	Type	Width [min]	Area [mAU*s]	Height [mAU]	Area %
1	16.599	BB	0.4525	4754.15430	161.22090	50.1708
2	26.900	BB	0.7368	4721.79199	95.83613	49.8292

Totals : 9475.94629 257.05703

Signal 2: DAD1 B, Sig=210,4 Ref=360,100

Peak #	RetTime [min]	Type	Width [min]	Area [mAU*s]	Height [mAU]	Area %
1	16.599	BB	0.4508	1.56068e4	528.08820	50.2031
2	26.901	BB	0.7386	1.54806e4	314.07037	49.7969

Totals : 3.10874e4 842.15857

Signal 3: DAD1 D, Sig=230,4 Ref=360,100

Peak #	RetTime [min]	Type	Width [min]	Area [mAU*s]	Height [mAU]	Area %
1	16.598	BB	0.4517	8994.22949	304.39835	50.1180
2	26.901	BB	0.7478	8951.88574	181.36696	49.8820

Totals : 1.79461e4 485.76530

*** End of Report ***

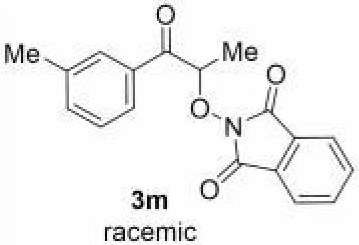

3m
racemic

===

Acq. Operator : SYSTEM   Seq. Line : 6
Sample Operator : SYSTEM
Acq. Instrument : HPLC   Location : P2-B-01
Injection Date : 11/3/2023 4:34:53 pm   Inj : 1
Inj Volume : 2.000 µl
Different Inj Volume from Sample Entry! Actual Inj Volume : 5.000 µl
Acq. Method : C:\Users\Public\Documents\ChemStation\1\Data\SUN\SUN 2023-03-11 14-02-31 \IC3-50-40.M
Last changed : 7/2/2023 9:36:14 am by SYSTEM
Analysis Method : C:\Users\Public\Documents\ChemStation\1\Data\SUN\SUN 2023-03-11 14-02-31 \IC3-50-40.M (Sequence Method)
Last changed : 11/3/2023 7:24:09 pm by SYSTEM
(modified after loading)
Additional Info : Peak(s) manually integrated

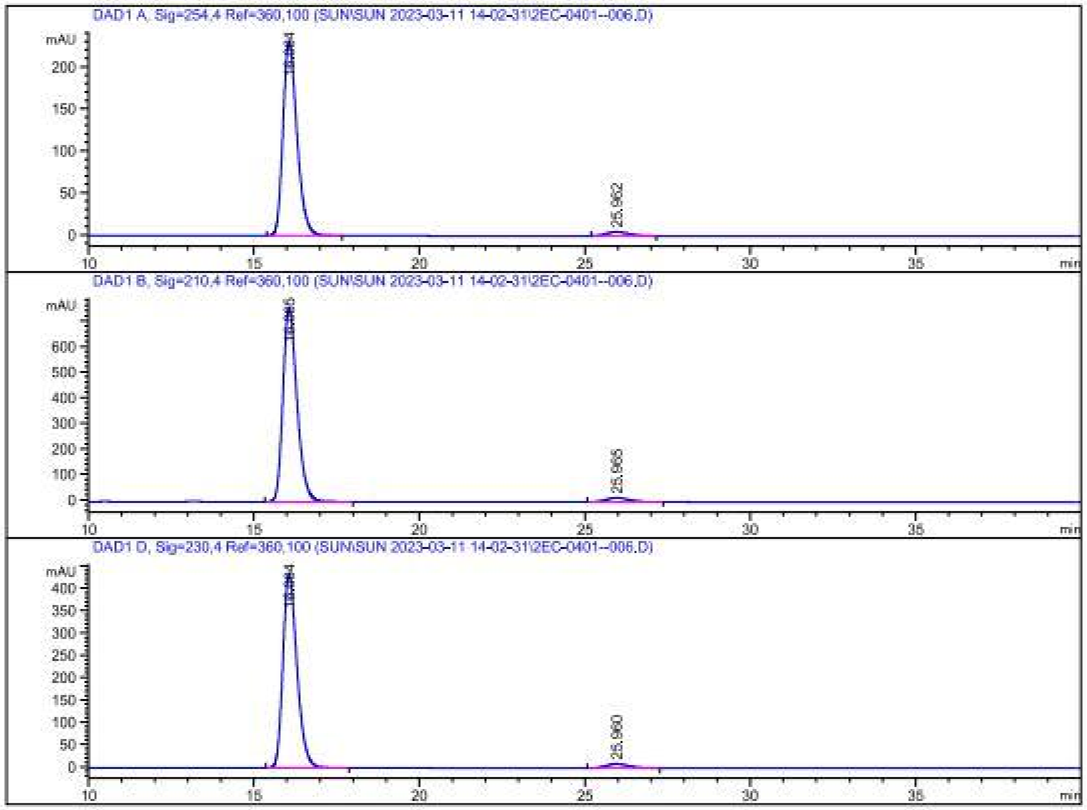

Data File C:\Users\P...ents\ChemStation\1\Data\SUN\SUN 2023-03-11 14-02-31\2EC-0401--006.D
Sample Name: qcx-6-50a

===

Area Percent Report

===

Sorted By : Signal
Multiplier : 1.0000
Dilution : 1.0000
Use Multiplier & Dilution Factor with ISTDs

Signal 1: DAD1 A, Sig=254,4 Ref=360,100

Peak #	RetTime [min]	Type	Width [min]	Area [mAU*s]	Height [mAU]	Area %
1	16.064	BB	0.4476	6790.89893	231.91322	96.7506
2	25.962	BB	0.5428	228.07607	4.93370	3.2494

Totals : 7018.97499 236.84693

Signal 2: DAD1 B, Sig=210,4 Ref=360,100

Peak #	RetTime [min]	Type	Width [min]	Area [mAU*s]	Height [mAU]	Area %
1	16.065	BB	0.4494	2.22628e4	759.74152	96.6391
2	25.965	BB	0.5542	774.25140	16.40354	3.3609

Totals : 2.30370e4 776.14506

Signal 3: DAD1 D, Sig=230,4 Ref=360,100

Peak #	RetTime [min]	Type	Width [min]	Area [mAU*s]	Height [mAU]	Area %
1	16.064	BB	0.4502	1.27956e4	436.19165	96.6518
2	25.960	BB	0.5511	443.26202	9.43480	3.3482

Totals : 1.32389e4 445.62645

===

*** End of Report ***

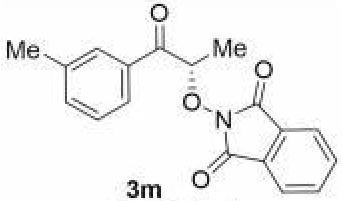

3m
enantioenriched

Area Percent Report

sample OH insertion

Data file: C:\Users\Public\Documents\ChemStation\1\Data\2025-07-12\qcx 2025-07-25 13-44-53\002-P2-A1-qcx-9-210a.D

Acquisition Data:

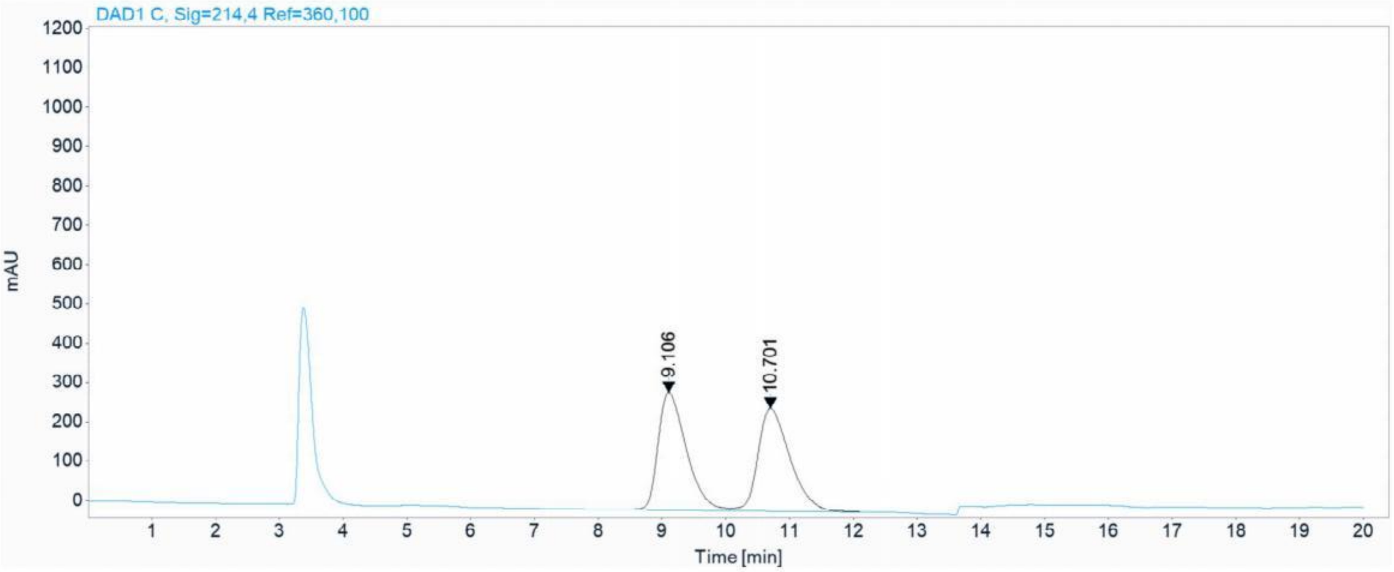

Signal: DAD1 C, Sig=214,4 Ref=360,100

RT [min]	Width [min]	Height	Area	Area%
9.106	0.4773	299.9527	9185.3564	49.9627
10.701	0.5358	261.9041	9199.0801	50.0373
		Sum	18384.4365	100.0000

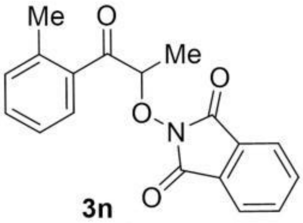

3n
racemic

Area Percent Report

sample OH insertion

Data file: C:\Users\Public\Documents\ChemStation\1\Data\2025-07-12\qcx 2025-07-25 13-44-53\006-P2-A2-qcx-9-210b.D

Acquisition Data:

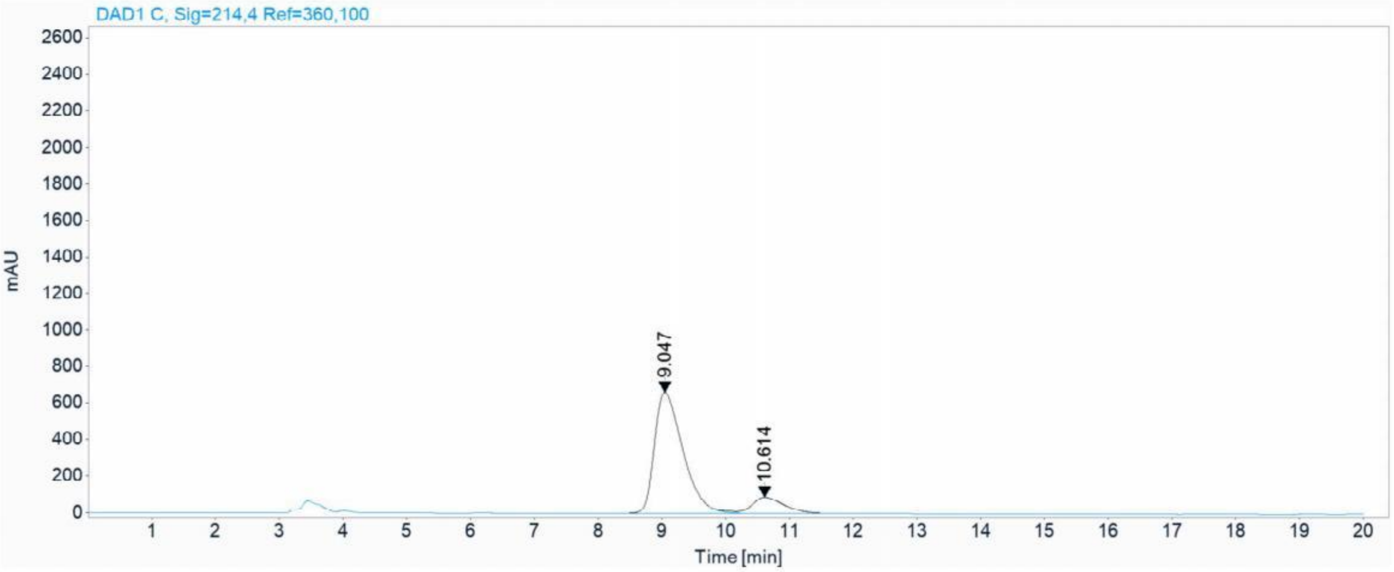

Signal: DAD1 C, Sig=214,4 Ref=360,100

RT [min]	Width [min]	Height	Area	Area%
9.047	0.4793	663.0869	20535.2617	86.4675
10.614	0.5165	87.9162	3213.8433	13.5325
		Sum	23749.1050	100.0000

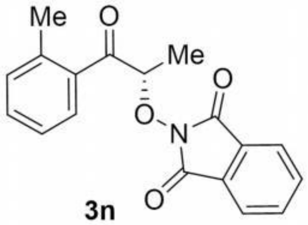

3n
enantioenriched

Acq. Operator : SYSTEM Seq. Line : 122
Sample Operator : SYSTEM
Acq. Instrument : HPLC Location : P2-B-02
Injection Date : 10/3/2023 12:42:01 am Inj : 1
Inj Volume : 2.000 µl
Different Inj Volume from Sample Entry! Actual Inj Volume : 12.000 µl
Acq. Method : C:\Users\Public\Documents\ChemStation\1\Data\SUN\SUN 2023-03-07 15-14-32\IC3-50-60.M
Last changed : 12/2/2023 2:10:14 pm by SYSTEM
Analysis Method : C:\Users\Public\Documents\ChemStation\1\Data\SUN\SUN 2023-03-07 15-14-32\IC3-50-60.M (Sequence Method)
Last changed : 10/3/2023 1:32:23 pm by SYSTEM
(modified after loading)
Additional Info : Peak(s) manually integrated

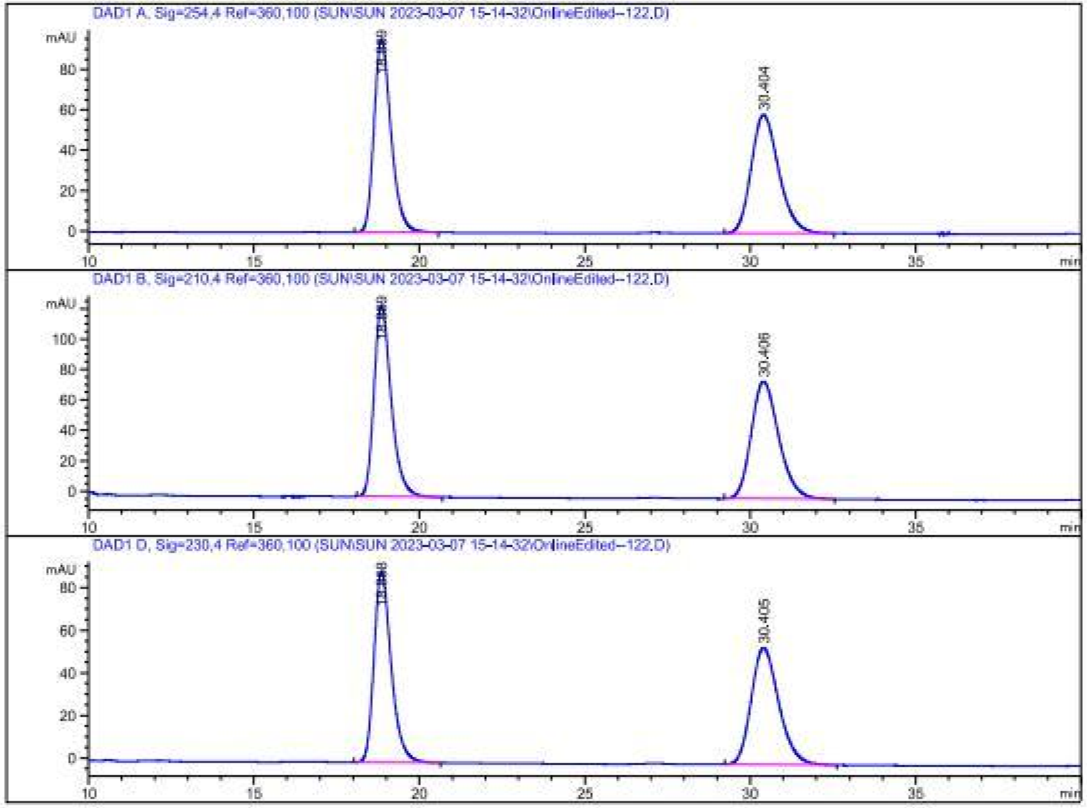

Data File C:\Users\P...\ChemStation\1\Data\SUN\SUN 2023-03-07 15-14-32\OnlineEdited--122.D
Sample Name: qcx-6-54b

Area Percent Report

Sorted By : Signal
Multiplier : 1.0000
Dilution : 1.0000
Use Multiplier & Dilution Factor with ISTDs

Signal 1: DAD1 A, Sig=254,4 Ref=360,100

Peak #	RetTime [min]	Type	Width [min]	Area [mAU*s]	Height [mAU]	Area %
1	18.859	BB	0.5292	3430.74829	95.71107	50.1240
2	30.404	BB	0.6977	3413.77808	58.68036	49.8760

Totals : 6844.52637 154.39143

Signal 2: DAD1 B, Sig=210,4 Ref=360,100

Peak #	RetTime [min]	Type	Width [min]	Area [mAU*s]	Height [mAU]	Area %
1	18.859	BB	0.5286	4546.93848	125.83208	50.3362
2	30.406	BB	0.7577	4486.19385	77.17481	49.6638

Totals : 9033.13232 203.00689

Signal 3: DAD1 D, Sig=230,4 Ref=360,100

Peak #	RetTime [min]	Type	Width [min]	Area [mAU*s]	Height [mAU]	Area %
1	18.858	BB	0.5387	3244.44336	89.75050	50.3922
2	30.405	BB	0.6917	3193.93506	54.94676	49.6078

Totals : 6438.37842 144.69726

*** End of Report ***

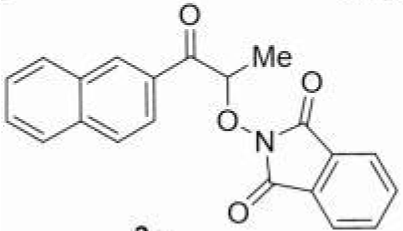

3o
racemic

===

Acq. Operator : SYSTEM   Seq. Line : 123
Sample Operator : SYSTEM
Acq. Instrument : HPLC   Location : P1-F-05
Injection Date : 10/3/2023 1:43:10 am   Inj : 1
Inj Volume : 2.000 µl
Different Inj Volume from Sample Entry! Actual Inj Volume : 8.000 µl
Acq. Method : C:\Users\Public\Documents\ChemStation\1\Data\SUN\SUN 2023-03-07 15-14-32\IC3-50-60.M
Last changed : 12/2/2023 2:10:14 pm by SYSTEM
Analysis Method : C:\Users\Public\Documents\ChemStation\1\Data\SUN\SUN 2023-03-07 15-14-32\IC3-50-60.M (Sequence Method)
Last changed : 10/3/2023 1:32:23 pm by SYSTEM
(modified after loading)
Additional Info : Peak(s) manually integrated

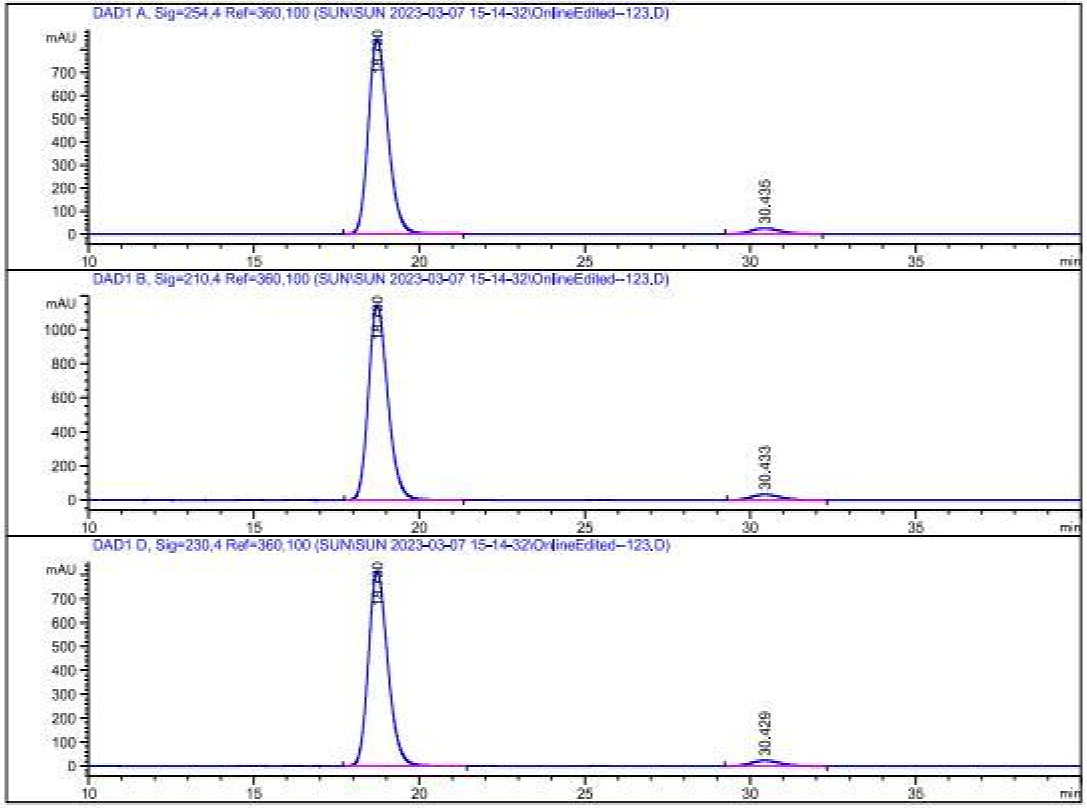

Data File C:\Users\P...\ChemStation\1\Data\SUN\SUN 2023-03-07 15-14-32\OnlineEdited--123.D
Sample Name: qcx-6-48a

===

Area Percent Report

===

Sorted By : Signal
Multiplier : 1.0000
Dilution : 1.0000
Use Multiplier & Dilution Factor with ISTDs

Signal 1: DAD1 A, Sig=254,4 Ref=360,100

Peak #	RetTime [min]	Type	Width [min]	Area [mAU*s]	Height [mAU]	Area %
1	18.730	BB	0.6308	3.47852e4	845.85919	95.7313
2	30.435	BB	0.7183	1551.10107	25.38738	4.2687
Totals :				3.63363e4	871.24657	

Signal 2: DAD1 B, Sig=210,4 Ref=360,100

Peak #	RetTime [min]	Type	Width [min]	Area [mAU*s]	Height [mAU]	Area %
1	18.730	BB	0.6151	4.65898e4	1143.96985	95.8030
2	30.433	BB	0.7165	2041.02917	33.37521	4.1970
Totals :				4.86308e4	1177.34505	

Signal 3: DAD1 D, Sig=230,4 Ref=360,100

Peak #	RetTime [min]	Type	Width [min]	Area [mAU*s]	Height [mAU]	Area %
1	18.730	BB	0.6240	3.32093e4	815.67664	95.7954
2	30.429	BB	0.7193	1457.60132	23.79004	4.2046
Totals :				3.46669e4	839.46667	

===

*** End of Report ***

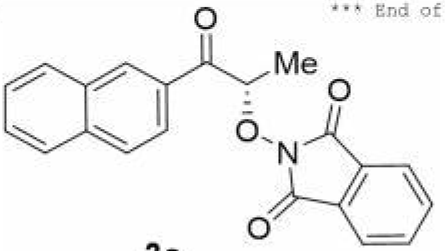

3o
enantioenriched

Acq. Operator : SYSTEM Seq. Line : 73
Sample Operator : SYSTEM
Acq. Instrument : HPLC Location : P1-F-09
Injection Date : 4/3/2023 5:42:21 am Inj : 1
Inj Volume : 2.000 µl
Different Inj Volume from Sample Entry! Actual Inj Volume : 3.000 µl
Acq. Method : C:\Users\Public\Documents\ChemStation\1\Data\SUN\SUN 2023-03-02 14-47-18\IC3-30-60.M
Last changed : 10/11/2022 9:42:07 am by SYSTEM
Analysis Method : C:\Users\Public\Documents\ChemStation\1\Data\SUN\SUN 2023-03-02 14-47-18\IC3-30-60.M (Sequence Method)
Last changed : 10/3/2023 1:30:41 pm by SYSTEM
(modified after loading)
Additional Info : Peak(s) manually integrated

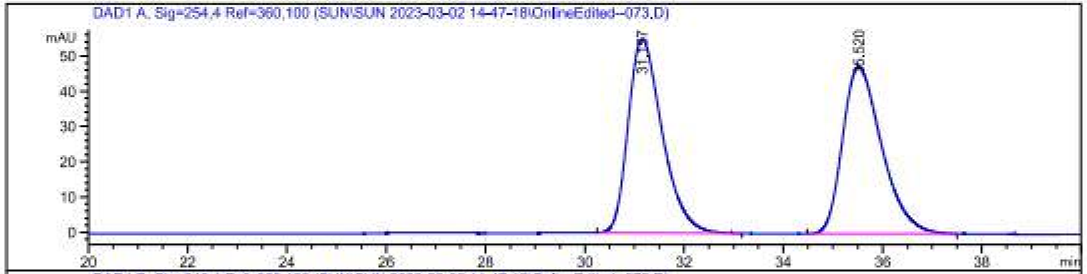

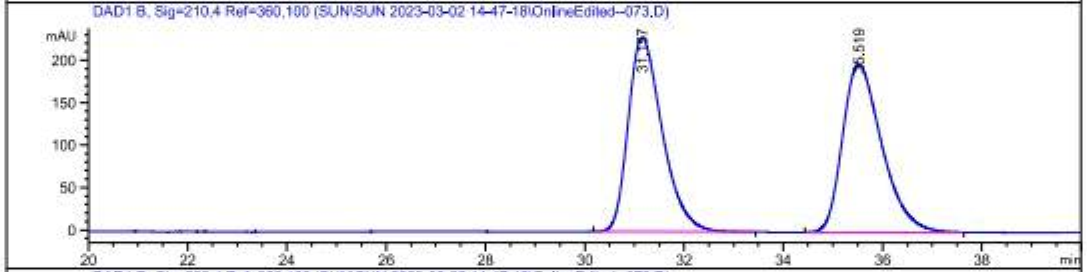

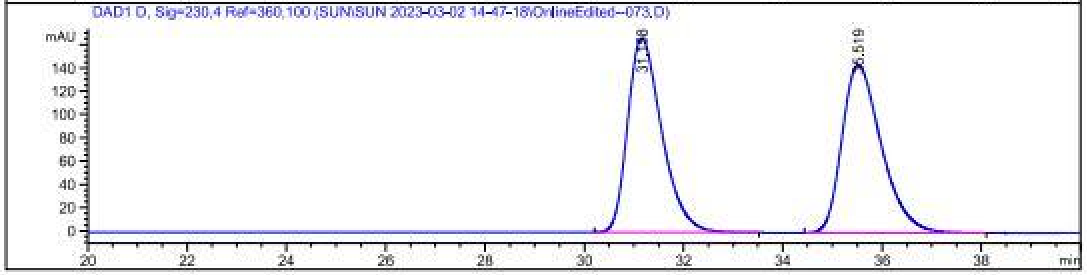

Area Percent Report

Sorted By : Signal
Multiplier : 1.0000
Dilution : 1.0000
Use Multiplier & Dilution Factor with ISTDs

Signal 1: DAD1 A, Sig=254,4 Ref=360,100

Peak #	RetTime [min]	Type	Width [min]	Area [mAU*s]	Height [mAU]	Area %
1	31.157	BB	0.5995	2623.24561	54.94096	50.1665
2	35.520	BB	0.7222	2605.83423	47.44803	49.8335
Totals :				5229.07983	102.38899	

Signal 2: DAD1 B, Sig=210,4 Ref=360,100

Peak #	RetTime [min]	Type	Width [min]	Area [mAU*s]	Height [mAU]	Area %
1	31.157	BB	0.7056	1.09386e4	228.85098	50.1370
2	35.519	BB	0.7915	1.08788e4	197.84859	49.8630
Totals :				2.18173e4	426.69957	

Signal 3: DAD1 D, Sig=230,4 Ref=360,100

Peak #	RetTime [min]	Type	Width [min]	Area [mAU*s]	Height [mAU]	Area %
1	31.158	BB	0.7165	7976.72607	166.76875	50.0401
2	35.519	BB	0.7873	7963.94873	144.32182	49.9599
Totals :				1.59407e4	311.09058	

*** End of Report ***

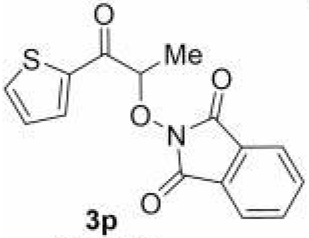

3p
racemic

Acq. Operator : SYSTEM   Seq. Line : 120
Sample Operator : SYSTEM
Acq. Instrument : HPLC   Location : P1-F-04
Injection Date : 9/3/2023 11:54:57 pm   Inj : 1
Inj Volume : 2.000 µl
Different Inj Volume from Sample Entry! Actual Inj Volume : 8.000 µl
Acq. Method : C:\Users\Public\Documents\ChemStation\1\Data\SUN\SUN 2023-03-07 15-14-32\IC3-30-40.M
Last changed : 27/2/2023 10:13:57 pm by SYSTEM
Analysis Method : C:\Users\Public\Documents\ChemStation\1\Data\SUN\SUN 2023-03-07 15-14-32\IC3-30-40.M (Sequence Method)
Last changed : 10/3/2023 1:27:47 pm by SYSTEM
(modified after loading)
Additional Info : Peak(s) manually integrated

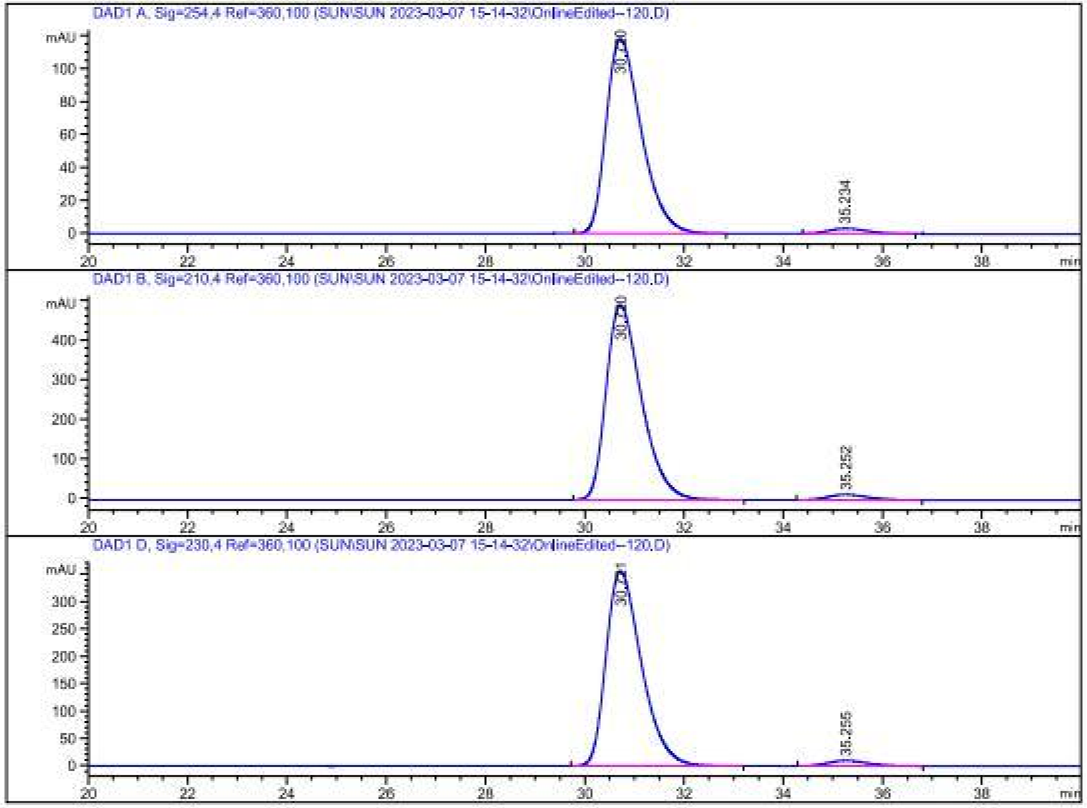

Data File C:\Users\P...\ChemStation\1\Data\SUN\SUN 2023-03-07 15-14-32\OnlineEdited--120.D
Sample Name: qcx-6-48b

Area Percent Report

Sorted By : Signal
Multiplier : 1.0000
Dilution : 1.0000
Use Multiplier & Dilution Factor with ISTDs

Signal 1: DAD1 A, Sig=254,4 Ref=360,100

Peak #	RetTime [min]	Type	Width [min]	Area [mAU*s]	Height [mAU]	Area %
1	30.720	BB	0.6976	5832.63086	118.21059	97.0658
2	35.234	BB	0.6383	176.31177	3.23524	2.9342
Totals :				6008.94263	121.44583	

Signal 2: DAD1 B, Sig=210,4 Ref=360,100

Peak #	RetTime [min]	Type	Width [min]	Area [mAU*s]	Height [mAU]	Area %
1	30.720	BB	0.7270	2.43238e4	491.77792	96.9509
2	35.252	BB	0.6524	764.99194	13.74291	3.0491
Totals :				2.50888e4	505.52083	

Signal 3: DAD1 D, Sig=230,4 Ref=360,100

Peak #	RetTime [min]	Type	Width [min]	Area [mAU*s]	Height [mAU]	Area %
1	30.721	BB	0.7472	1.76183e4	355.80621	96.9275
2	35.255	BB	0.6537	558.49072	10.04498	3.0725
Totals :				1.81768e4	365.85119	

*** End of Report ***

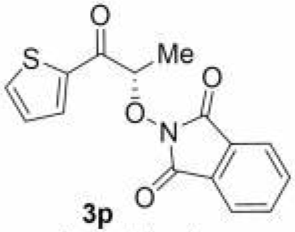

3p
enantioenriched

Acq. Operator	: SYSTEM	Seq. Line :	75
Sample Operator	: SYSTEM		
Acq. Instrument	: HPLC	Location :	P1-F-10
Injection Date	: 4/3/2023 7:44:31 am	Inj :	1
		Inj Volume :	2.000 µl

Different Inj Volume from Sample Entry! Actual Inj Volume : 3.000 µl

Acq. Method : C:\Users\Public\Documents\ChemStation\1\Data\SUN\SUN 2023-03-02 14-47-18\IC3-30-60.M
Last changed : 10/11/2022 9:42:07 am by SYSTEM
Analysis Method : C:\Users\Public\Documents\ChemStation\1\Data\SUN\SUN 2023-03-02 14-47-18\IC3-30-60.M (Sequence Method)
Last changed : 6/3/2023 1:45:03 pm by SYSTEM
(modified after loading)
Additional Info : Peak(s) manually integrated

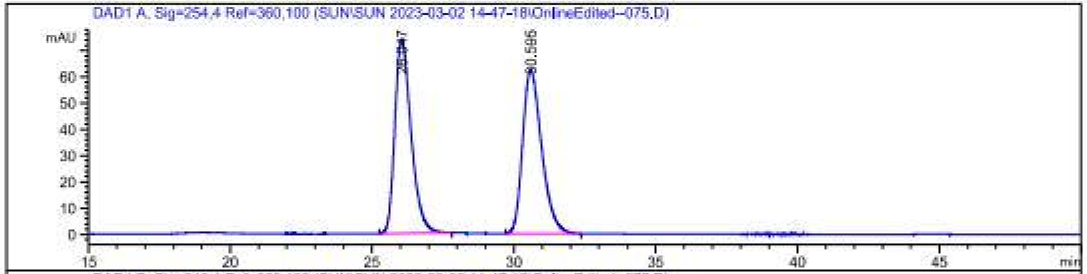

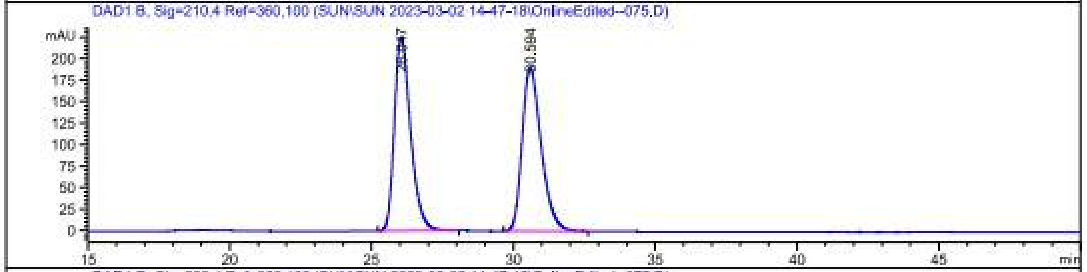

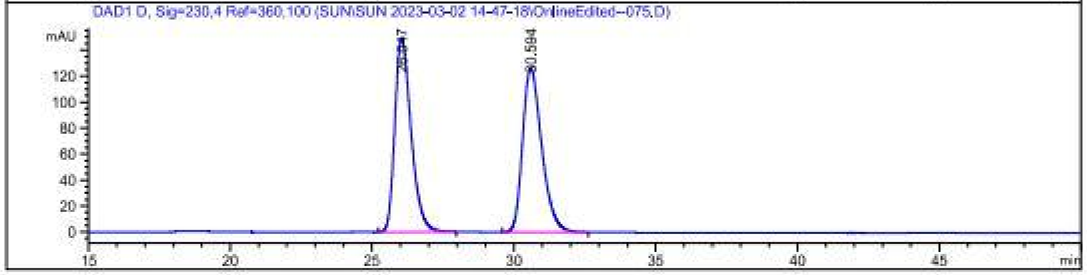

Area Percent Report

Sorted By : Signal
Multiplier : 1.0000
Dilution : 1.0000
Use Multiplier & Dilution Factor with ISTDs

Signal 1: DAD1 A, Sig=254,4 Ref=360,100

Peak #	RetTime [min]	Type	Width [min]	Area [mAU*s]	Height [mAU]	Area %
1	26.047	BB	0.5716	2938.99194	73.88852	50.2648
2	30.595	BB	0.6235	2908.03052	62.13552	49.7352

Totals : 5847.02246 136.02404

Signal 2: DAD1 B, Sig=210,4 Ref=360,100

Peak #	RetTime [min]	Type	Width [min]	Area [mAU*s]	Height [mAU]	Area %
1	26.047	BB	0.5992	8965.89355	225.08868	50.1733
2	30.594	BB	0.6758	8903.94336	189.63184	49.8267

Totals : 1.78698e4 414.72052

Signal 3: DAD1 D, Sig=230,4 Ref=360,100

Peak #	RetTime [min]	Type	Width [min]	Area [mAU*s]	Height [mAU]	Area %
1	26.047	BB	0.6000	5942.96582	149.41449	50.0656
2	30.594	BB	0.6939	5927.39844	126.10099	49.9344

Totals : 1.18704e4 275.51548

*** End of Report ***

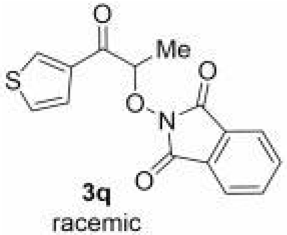

3q
racemic

Acq. Operator : SYSTEM Seq. Line : 33
Sample Operator : SYSTEM
Acq. Instrument : HPLC Location : P2-B-01
Injection Date : 19/3/2023 12:57:36 am Inj : 1
Inj Volume : 2.000 µl
Different Inj Volume from Sample Entry! Actual Inj Volume : 3.000 µl
Acq. Method : C:\Users\Public\Documents\ChemStation\1\Data\SUN\SUN 2023-03-18 13-24-33\IC3-30-60.M
Last changed : 10/11/2022 9:42:07 am by SYSTEM
Analysis Method : C:\Users\Public\Documents\ChemStation\1\Data\SUN\SUN 2023-03-18 13-24-33\IC3-30-60.M (Sequence Method)
Last changed : 19/3/2023 8:48:33 pm by SYSTEM
(modified after loading)
Additional Info : Peak(s) manually integrated

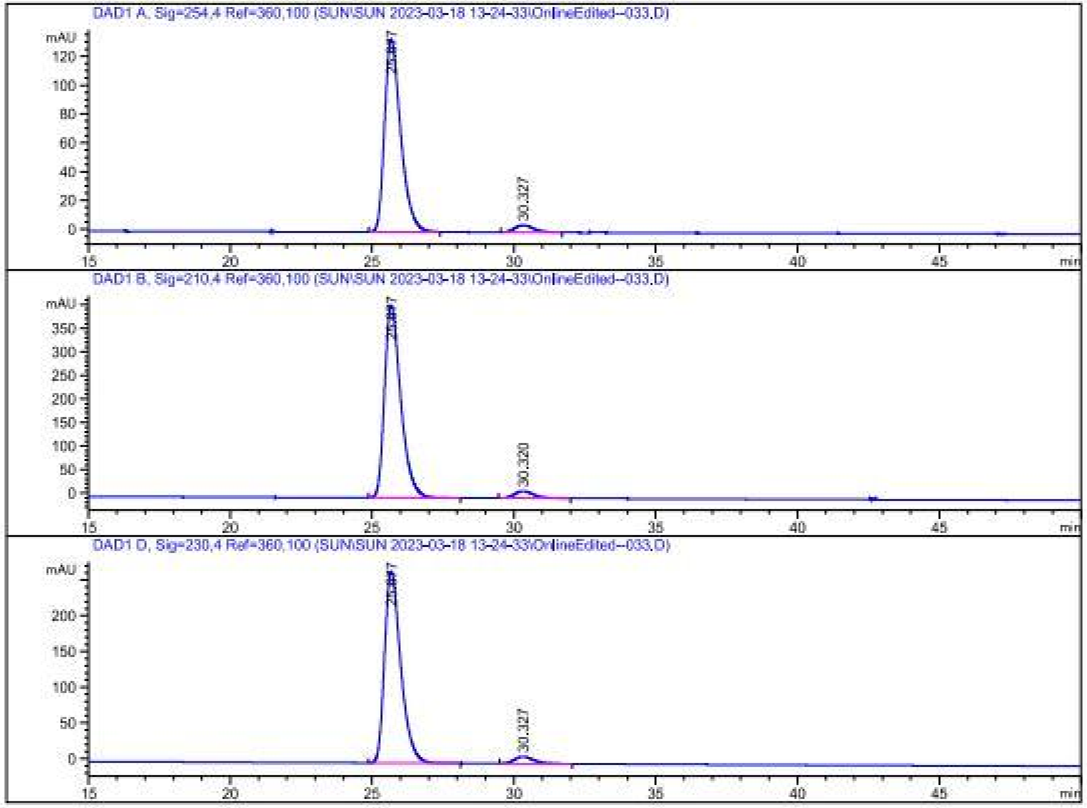

Data File C:\Users\P...\ChemStation\1\Data\SUN\SUN 2023-03-18 13-24-33\OnlineEdited--033.D
Sample Name: qcx-6-60a

Area Percent Report

Sorted By : Signal
Multiplier : 1.0000
Dilution : 1.0000
Use Multiplier & Dilution Factor with ISTDs

Signal 1: DAD1 A, Sig=254,4 Ref=360,100

Peak #	RetTime [min]	Type	Width [min]	Area [mAU*s]	Height [mAU]	Area %
1	25.677	BB	0.5893	5280.27002	133.82410	95.8389
2	30.327	BB	0.5487	229.25746	4.90559	4.1611

Totals : 5509.52748 138.72969

Signal 2: DAD1 B, Sig=210,4 Ref=360,100

Peak #	RetTime [min]	Type	Width [min]	Area [mAU*s]	Height [mAU]	Area %
1	25.677	BB	0.5995	1.61906e4	408.85776	95.7618
2	30.320	BB	0.5571	716.55060	15.12763	4.2382

Totals : 1.69071e4 423.98539

Signal 3: DAD1 D, Sig=230,4 Ref=360,100

Peak #	RetTime [min]	Type	Width [min]	Area [mAU*s]	Height [mAU]	Area %
1	25.677	BB	0.5973	1.06487e4	268.46844	95.7419
2	30.327	BB	0.5581	473.59833	9.98116	4.2581

Totals : 1.11223e4 278.44960

*** End of Report ***

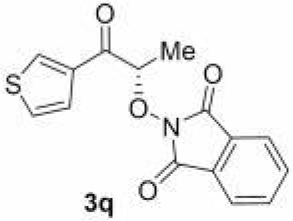

3q
enantioenriched

===

Acq. Operator : SYSTEM Seq. Line : 128
Sample Operator : SYSTEM
Acq. Instrument : HPLC Location : P2-B-03
Injection Date : 10/3/2023 5:38:32 am Inj : 1
Inj Volume : 2.000 µl
Different Inj Volume from Sample Entry! Actual Inj Volume : 6.000 µl
Acq. Method : C:\Users\Public\Documents\ChemStation\1\Data\SUN\SUN 2023-03-07 15-14-32\IC3-20-40.M
Last changed : 4/1/2023 3:55:34 pm by SYSTEM
Analysis Method : C:\Users\Public\Documents\ChemStation\1\Data\SUN\SUN 2023-03-07 15-14-32\IC3-20-40.M (Sequence Method)
Last changed : 10/3/2023 1:41:31 pm by SYSTEM
(modified after loading)
Additional Info : Peak(s) manually integrated

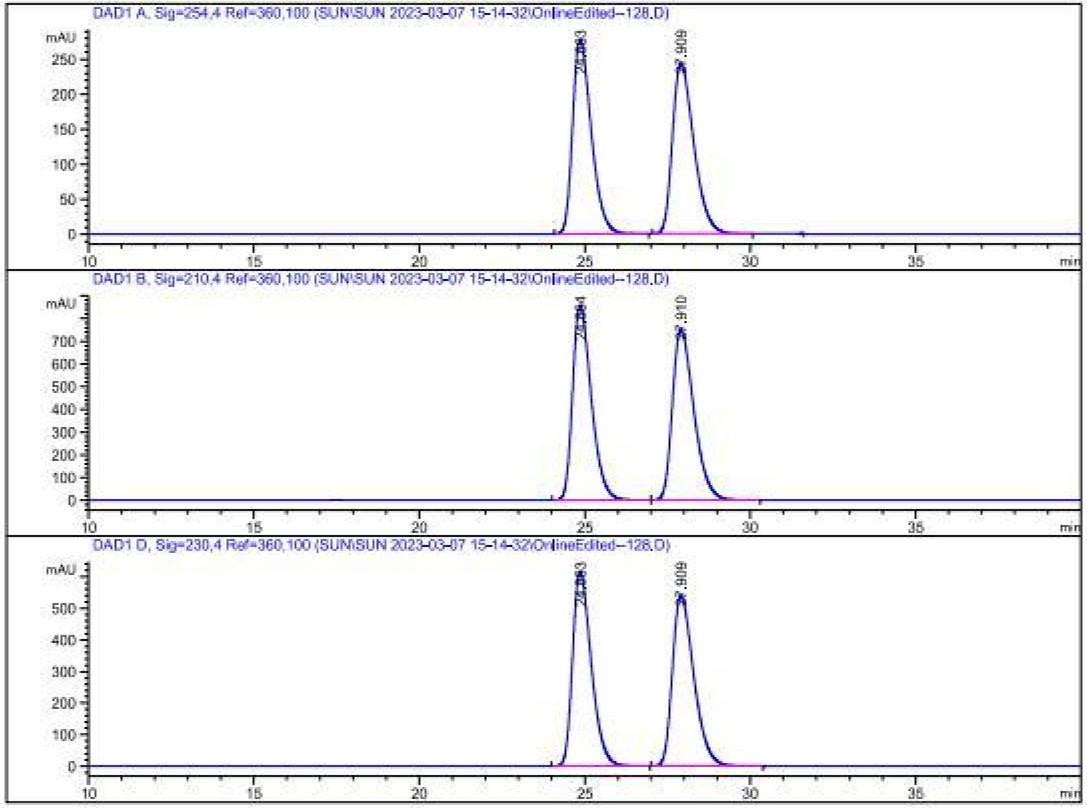

Data File C:\Users\P...\ChemStation\1\Data\SUN\SUN 2023-03-07 15-14-32\OnlineEdited--128.D
Sample Name: qcx-6-54c

===

Area Percent Report

===

Sorted By : Signal
Multiplier : 1.0000
Dilution : 1.0000
Use Multiplier & Dilution Factor with ISTDs

Signal 1: DAD1 A, Sig=254,4 Ref=360,100

Peak #	RetTime [min]	Type	Width [min]	Area [mAU*s]	Height [mAU]	Area %
1	24.863	BB	0.5961	1.09551e4	278.38577	50.0196
2	27.909	BB	0.6770	1.09465e4	243.04114	49.9804

Totals : 2.19017e4 521.42691

Signal 2: DAD1 B, Sig=210,4 Ref=360,100

Peak #	RetTime [min]	Type	Width [min]	Area [mAU*s]	Height [mAU]	Area %
1	24.864	BB	0.5941	3.36736e4	856.69476	50.0033
2	27.910	BB	0.6762	3.36691e4	747.96661	49.9967

Totals : 6.73427e4 1604.66138

Signal 3: DAD1 D, Sig=230,4 Ref=360,100

Peak #	RetTime [min]	Type	Width [min]	Area [mAU*s]	Height [mAU]	Area %
1	24.863	BB	0.6031	2.42185e4	616.17798	50.0015
2	27.909	BB	0.6846	2.42170e4	538.00739	49.9985

Totals : 4.84355e4 1154.18536

===

*** End of Report ***

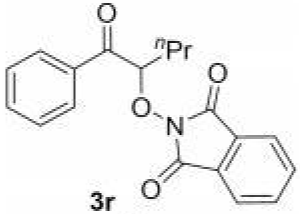

3r
racemic

===

Acq. Operator : SYSTEM Seq. Line : 40
Sample Operator : SYSTEM
Acq. Instrument : HPLC Location : P2-B-01
Injection Date : 25/2/2023 12:49:19 pm Inj : 1
Inj Volume : 2.000 µl
Different Inj Volume from Sample Entry! Actual Inj Volume : 5.000 µl
Acq. Method : C:\Users\Public\Documents\ChemStation\1\Data\SUN\SUN 2023-02-24 20-02-45\IC3-20-40.M
Last changed : 4/1/2023 3:55:34 pm by SYSTEM
Analysis Method : C:\Users\Public\Documents\ChemStation\1\Data\SUN\SUN 2023-02-24 20-02-45\IC3-20-40.M (Sequence Method)
Last changed : 10/3/2023 1:43:19 pm by SYSTEM
(modified after loading)
Additional Info : Peak(s) manually integrated

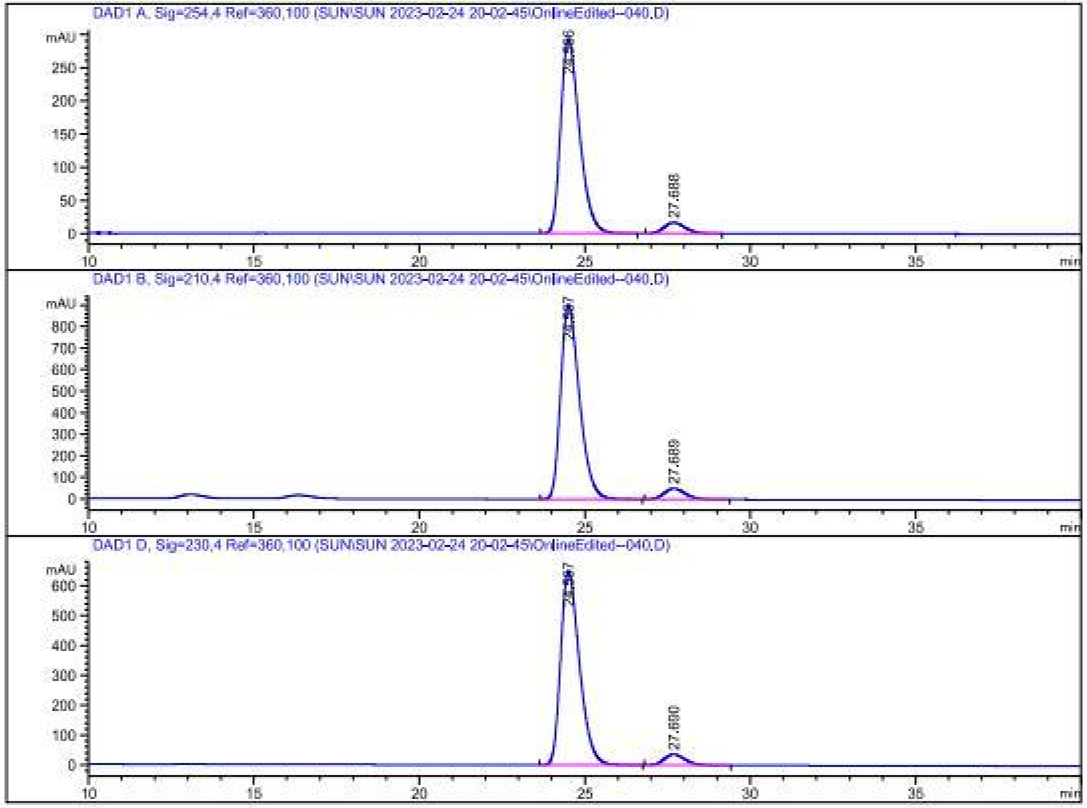

Data File C:\Users\P...\ChemStation\1\Data\SUN\SUN 2023-02-24 20-02-45\OnlineEdited--040.D
Sample Name: qcx-6-28b

===

Area Percent Report

===

Sorted By : Signal
Multiplier : 1.0000
Dilution : 1.0000
Use Multiplier & Dilution Factor with ISTDs

Signal 1: DAD1 A, Sig=254,4 Ref=360,100

Peak #	RetTime [min]	Type	Width [min]	Area [mAU*s]	Height [mAU]	Area %
1	24.506	BB	0.6017	1.15838e4	292.07068	94.1418
2	27.688	BB	0.5205	720.83380	16.31447	5.8582

Totals : 1.23046e4 308.38515

Signal 2: DAD1 B, Sig=210,4 Ref=360,100

Peak #	RetTime [min]	Type	Width [min]	Area [mAU*s]	Height [mAU]	Area %
1	24.507	BB	0.6037	3.57370e4	901.13293	94.1112
2	27.689	BB	0.5503	2236.16626	50.33469	5.8888

Totals : 3.79732e4 951.46762

Signal 3: DAD1 D, Sig=230,4 Ref=360,100

Peak #	RetTime [min]	Type	Width [min]	Area [mAU*s]	Height [mAU]	Area %
1	24.507	BB	0.6085	2.57160e4	648.62738	94.1030
2	27.690	BB	0.6038	1611.50452	36.27115	5.8970

Totals : 2.73275e4 684.89853

===

*** End of Report ***

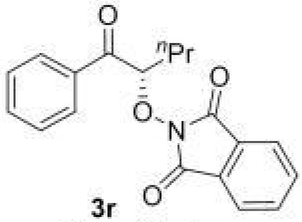

3r
enantioenriched

===

Acq. Operator : SYSTEM   Seq. Line : 14
Sample Operator : SYSTEM
Acq. Instrument : HPLC   Location : P1-F-02
Injection Date : 2/5/2023 5:08:43 pm   Inj : 1
Inj Volume : 2.000 µl
Different Inj Volume from Sample Entry! Actual Inj Volume : 1.000 µl
Acq. Method : C:\Users\Public\Documents\ChemStation\1\Data\SUN\SUN 2023-05-02 10-28-58\IC3-30-60.M
Last changed : 10/11/2022 9:42:07 am by SYSTEM
Analysis Method : C:\Users\Public\Documents\ChemStation\1\Data\SUN\SUN 2023-05-02 10-28-58\IC3-30-60.M (Sequence Method)
Last changed : 2/5/2023 10:10:35 pm by SYSTEM
(modified after loading)
Additional Info : Peak(s) manually integrated

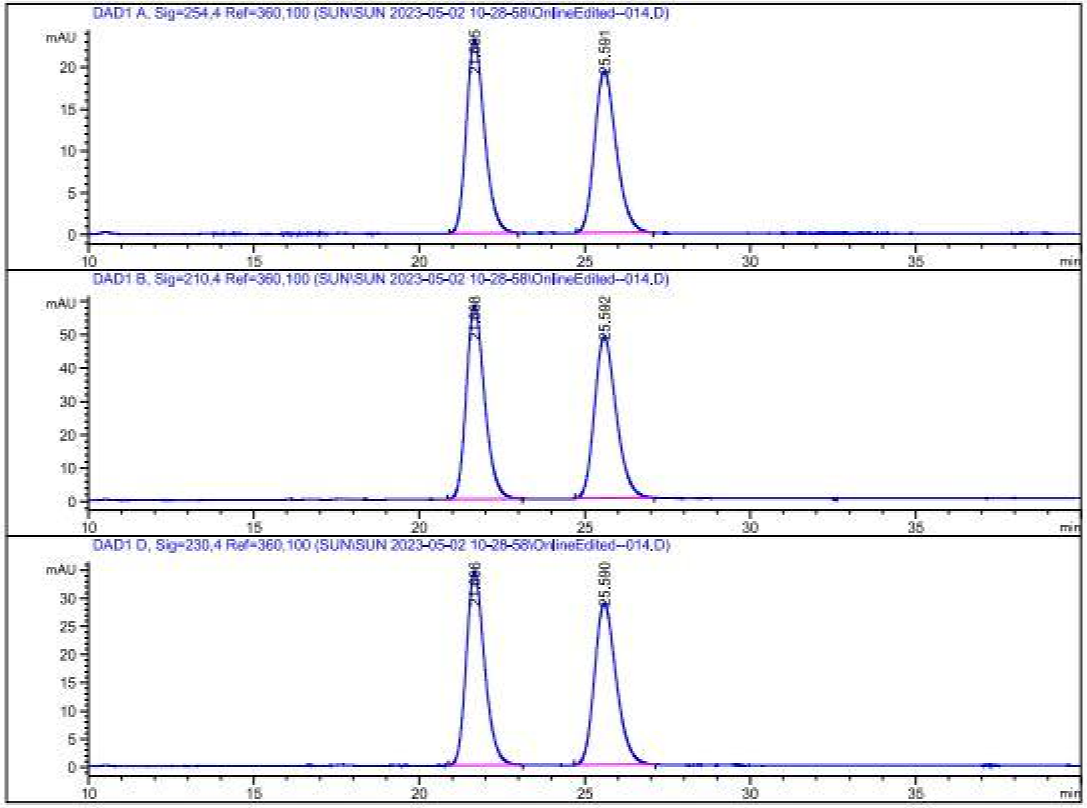

Data File C:\Users\P...\ChemStation\1\Data\SUN\SUN 2023-05-02 10-28-58\OnlineEdited--014.D
Sample Name: qcx-6-118a

===

Area Percent Report

===

Sorted By : Signal
Multiplier : 1.0000
Dilution : 1.0000
Use Multiplier & Dilution Factor with ISTDs

Signal 1: DAD1 A, Sig=254,4 Ref=360,100

Peak #	RetTime [min]	Type	Width [min]	Area [mAU*s]	Height [mAU]	Area %
1	21.665	BB	0.4810	889.00830	23.20498	50.1808
2	25.591	BB	0.5484	882.60364	19.32432	49.8192

Totals : 1771.61194 42.52930

Signal 2: DAD1 B, Sig=210,4 Ref=360,100

Peak #	RetTime [min]	Type	Width [min]	Area [mAU*s]	Height [mAU]	Area %
1	21.668	BB	0.5380	2230.33984	58.01522	50.2188
2	25.592	BB	0.5667	2210.90161	48.28942	49.7812

Totals : 4441.24146 106.30464

Signal 3: DAD1 D, Sig=230,4 Ref=360,100

Peak #	RetTime [min]	Type	Width [min]	Area [mAU*s]	Height [mAU]	Area %
1	21.666	BB	0.5153	1327.05945	34.53632	50.1465
2	25.590	BB	0.5784	1319.30688	28.77653	49.8535

Totals : 2646.36633 63.31285

===

*** End of Report ***

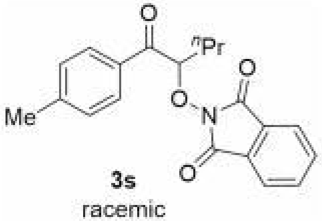

3s
racemic

Acq. Operator : SYSTEM Seq. Line : 2
Sample Operator : SYSTEM
Acq. Instrument : HPLC Location : P1-F-02
Injection Date : 1/5/2023 4:52:45 pm Inj : 1
Inj Volume : 2.000 µl
Different Inj Volume from Sample Entry! Actual Inj Volume : 1.000 µl
Acq. Method : C:\Users\Public\Documents\ChemStation\1\Data\SUN\SUN 2023-05-01 15-50-06\IC3-30-60.M
Last changed : 10/11/2022 9:42:07 am by SYSTEM
Analysis Method : C:\Users\Public\Documents\ChemStation\1\Data\SUN\SUN 2023-05-01 15-50-06\IC3-30-60.M (Sequence Method)
Last changed : 2/5/2023 10:09:13 pm by SYSTEM
(modified after loading)
Additional Info : Peak(s) manually integrated

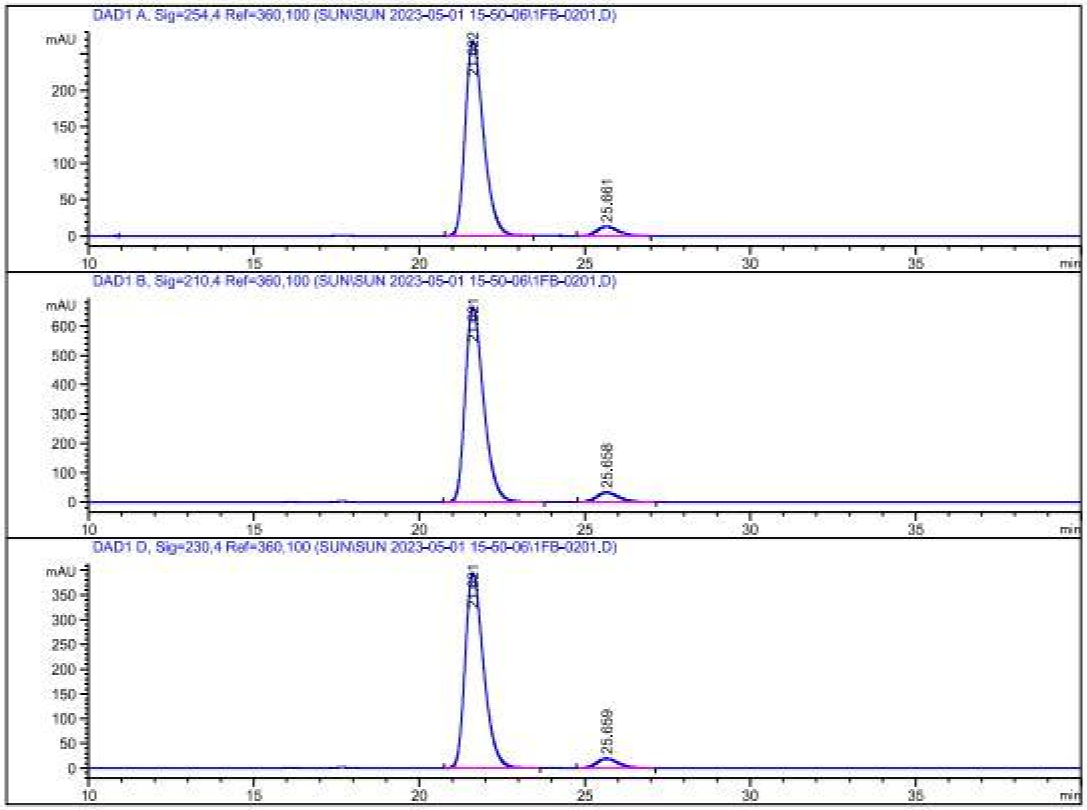

Data File C:\Users\P...Documents\ChemStation\1\Data\SUN\SUN 2023-05-01 15-50-06\1FB-0201.D
Sample Name: qcx-6-117a

Area Percent Report

Sorted By : Signal
Multiplier : 1.0000
Dilution : 1.0000
Use Multiplier & Dilution Factor with ISTDs

Signal 1: DAD1 A, Sig=254,4 Ref=360,100

Peak #	RetTime [min]	Type	Width [min]	Area [mAU*s]	Height [mAU]	Area %
1	21.622	BB	0.5846	1.03012e4	267.29343	94.5736
2	25.661	BB	0.5377	591.05487	12.98012	5.4264

Totals : 1.08922e4 280.27354

Signal 2: DAD1 B, Sig=210,4 Ref=360,100

Peak #	RetTime [min]	Type	Width [min]	Area [mAU*s]	Height [mAU]	Area %
1	21.621	BB	0.5804	2.56990e4	664.91931	94.5386
2	25.658	BB	0.5705	1484.61548	32.43724	5.4614

Totals : 2.71836e4 697.35655

Signal 3: DAD1 D, Sig=230,4 Ref=360,100

Peak #	RetTime [min]	Type	Width [min]	Area [mAU*s]	Height [mAU]	Area %
1	21.621	BB	0.5869	1.52268e4	394.01727	94.5186
2	25.659	BB	0.5373	883.03894	19.29879	5.4814

Totals : 1.61099e4 413.31606

*** End of Report ***

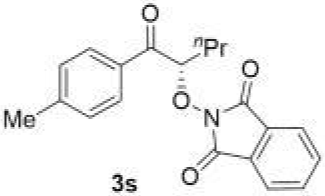

3s
enantioenriched

```
=====================================================================
Acq. Operator   : SYSTEM                         Seq. Line :  72
Sample Operator : SYSTEM
Acq. Instrument : HPLC                            Location :   P1-F-08
Injection Date  : 4/3/2023 5:01:15 am                  Inj :   1
                                              Inj Volume : 2.000 µl
Different Inj Volume from Sample Entry!  Actual Inj Volume : 3.000 µl
Acq. Method     : C:\Users\Public\Documents\ChemStation\1\Data\SUN\SUN 2023-03-02 14-47-18
                  \IC3-30-40.M
Last changed    : 27/2/2023 10:13:57 pm by SYSTEM
Analysis Method : C:\Users\Public\Documents\ChemStation\1\Data\SUN\SUN 2023-03-02 14-47-18
                  \IC3-30-40.M (Sequence Method)
Last changed    : 6/3/2023 1:42:15 pm by SYSTEM
                  (modified after loading)
Additional Info : Peak(s) manually integrated
```

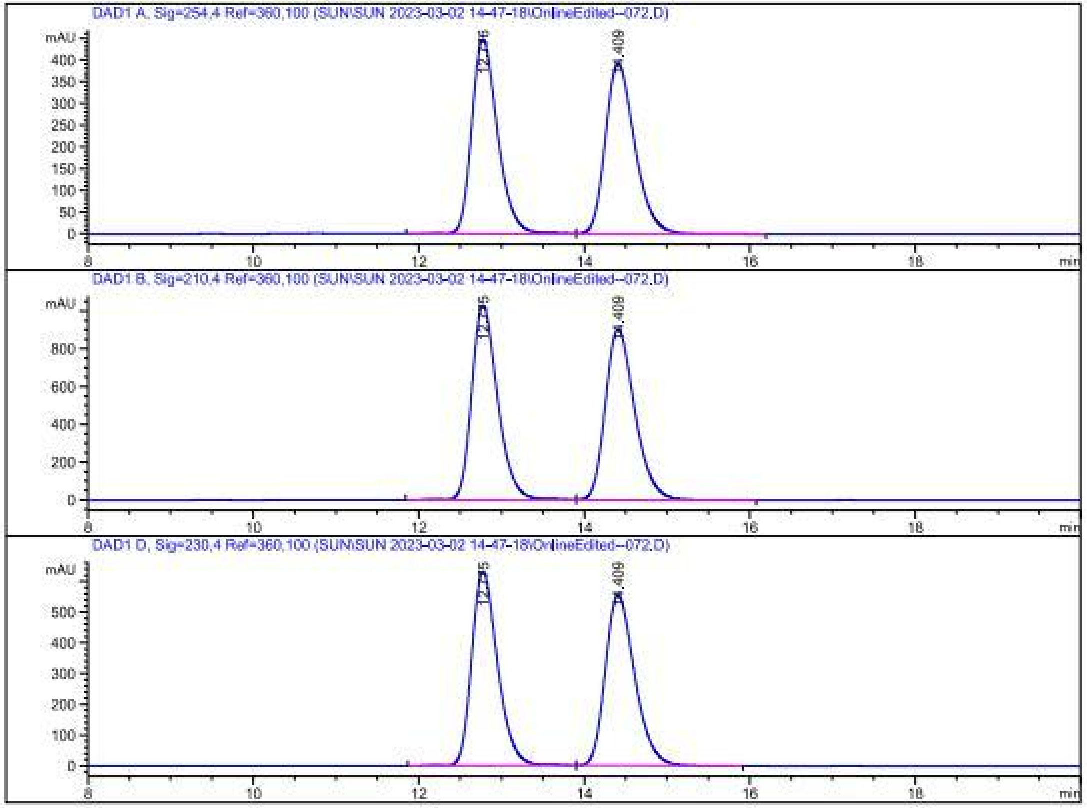

Data File C:\Users\P...\ChemStation\1\Data\SUN\SUN 2023-03-02 14-47-18\OnlineEdited--072.D
Sample Name: qcx-6-12c

```
=====================================================================
                         Area Percent Report
=====================================================================

Sorted By             :      Signal
Multiplier            :      1.0000
Dilution              :      1.0000
Use Multiplier & Dilution Factor with ISTDs
```

Signal 1: DAD1 A, Sig=254,4 Ref=360,100

Peak #	RetTime [min]	Type	Width [min]	Area [mAU*s]	Height [mAU]	Area %
1	12.776	VV R	0.3336	9788.07324	447.77429	50.1908
2	14.409	VB	0.3819	9713.66895	392.27051	49.8092

Totals : 1.95017e4 840.04480

Signal 2: DAD1 B, Sig=210,4 Ref=360,100

Peak #	RetTime [min]	Type	Width [min]	Area [mAU*s]	Height [mAU]	Area %
1	12.775	VV R	0.3332	2.25923e4	1031.09814	50.1701
2	14.409	VB	0.3795	2.24391e4	904.39636	49.8299

Totals : 4.50314e4 1935.49451

Signal 3: DAD1 D, Sig=230,4 Ref=360,100

Peak #	RetTime [min]	Type	Width [min]	Area [mAU*s]	Height [mAU]	Area %
1	12.775	VB R	0.3343	1.38172e4	633.11310	50.1436
2	14.409	BB	0.3821	1.37380e4	555.44611	49.8564

Totals : 2.75552e4 1188.55920

===
*** End of Report ***

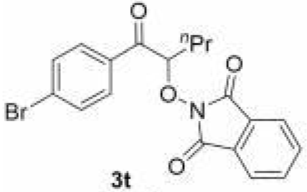

3t
racemic

```
=====================================================================
Acq. Operator   : SYSTEM                        Seq. Line :  15
Sample Operator : SYSTEM
Acq. Instrument : HPLC                           Location :   P2-B-01
Injection Date  : 27/3/2023 9:01:10 pm                Inj :   1
                                               Inj Volume : 2.000 µl
Different Inj Volume from Sample Entry!  Actual Inj Volume : 6.000 µl
Acq. Method     : C:\Users\Public\Documents\ChemStation\1\Data\SUN\SUN 2023-03-27 13-30-18
                  \IC3-30-40.M
Last changed    : 27/3/2023 9:31:50 pm by SYSTEM
                  (modified after loading)
Analysis Method : C:\Users\Public\Documents\ChemStation\1\Data\SUN\SUN 2023-03-27 13-30-18
                  \IC3-30-40.M (Sequence Method)
Last changed    : 27/3/2023 9:33:24 pm by SYSTEM
                  (modified after loading)
Additional Info : Peak(s) manually integrated
```

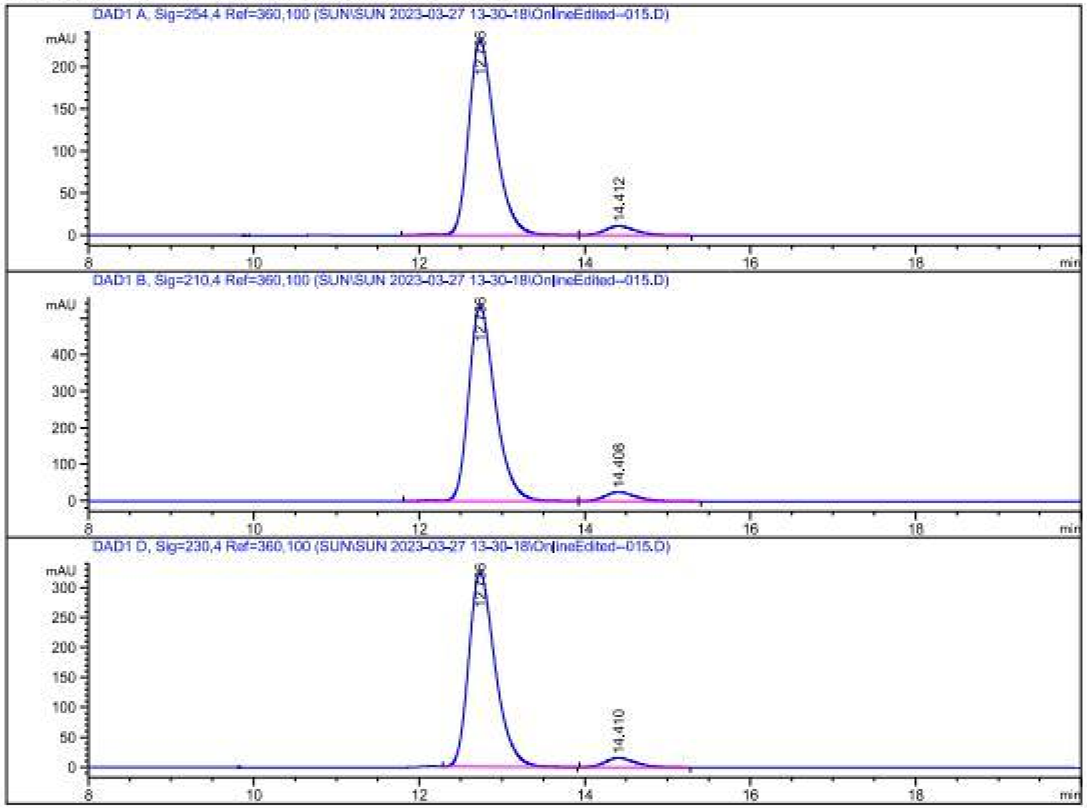

Data File C:\Users\P...\ChemStation\1\Data\SUN\SUN 2023-03-27 13-30-18\OnlineEdited--015.D
Sample Name: qcx-6-74d

Area Percent Report

```
Sorted By             :      Signal
Multiplier            :      1.0000
Dilution              :      1.0000
Use Multiplier & Dilution Factor with ISTDs
```

Signal 1: DAD1 A, Sig=254,4 Ref=360,100

Peak #	RetTime [min]	Type	Width [min]	Area [mAU*s]	Height [mAU]	Area %
1	12.736	BB	0.3408	5169.62012	231.23766	94.9711
2	14.412	BB	0.3307	273.74072	10.85390	5.0289

Totals : 5443.36084 242.09155

Signal 2: DAD1 B, Sig=210,4 Ref=360,100

Peak #	RetTime [min]	Type	Width [min]	Area [mAU*s]	Height [mAU]	Area %
1	12.736	VB R	0.3402	1.19449e4	533.59827	94.9181
2	14.408	BB	0.3648	639.53198	25.26444	5.0819

Totals : 1.25845e4 558.86271

Signal 3: DAD1 D, Sig=230,4 Ref=360,100

Peak #	RetTime [min]	Type	Width [min]	Area [mAU*s]	Height [mAU]	Area %
1	12.736	BB	0.3394	7217.59912	324.62729	94.8682
2	14.410	BB	0.3601	390.43066	15.46375	5.1318

Totals : 7608.02979 340.09104

*** End of Report ***

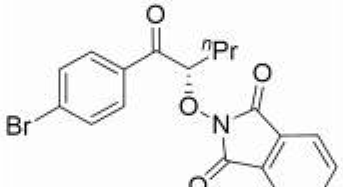

3t
enantioenriched

Acq. Operator : SYSTEM Seq. Line : 15
Sample Operator : SYSTEM
Acq. Instrument : HPLC Location : P1-F-03
Injection Date : 2/5/2023 6:09:42 pm Inj : 1
Inj Volume : 2.000 µl
Different Inj Volume from Sample Entry! Actual Inj Volume : 1.000 µl
Acq. Method : C:\Users\Public\Documents\ChemStation\1\Data\SUN\SUN 2023-05-02 10-28-58\IC3-30-60.M
Last changed : 10/11/2022 9:42:07 am by SYSTEM
Analysis Method : C:\Users\Public\Documents\ChemStation\1\Data\SUN\SUN 2023-05-02 10-28-58\IC3-30-60.M (Sequence Method)
Last changed : 2/5/2023 10:10:35 pm by SYSTEM
(modified after loading)
Additional Info : Peak(s) manually integrated

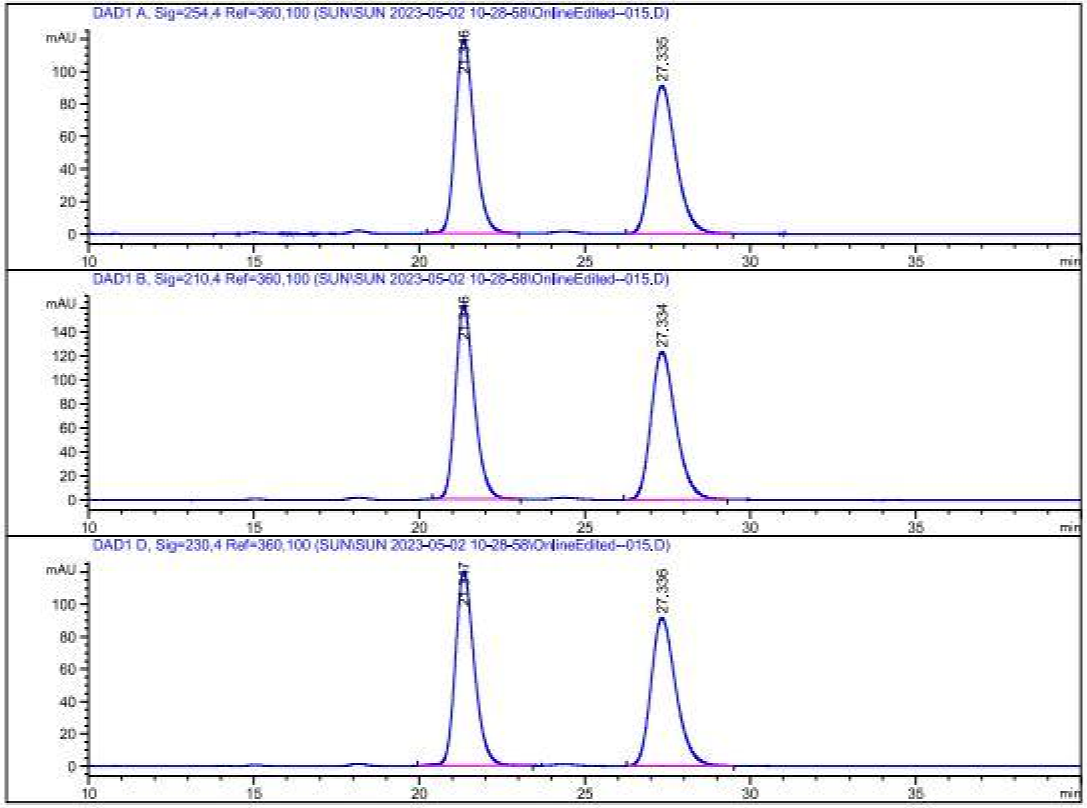

Data File C:\Users\P...\ChemStation\1\Data\SUN\SUN 2023-05-02 10-28-58\OnlineEdited--015.D
Sample Name: qcx-6-118b

Area Percent Report

Sorted By : Signal
Multiplier : 1.0000
Dilution : 1.0000
Use Multiplier & Dilution Factor with ISTDs

Signal 1: DAD1 A, Sig=254,4 Ref=360,100

Peak #	RetTime [min]	Type	Width [min]	Area [mAU*s]	Height [mAU]	Area %
1	21.346	BB	0.6007	4732.01611	119.18471	49.8567
2	27.335	BB	0.7334	4759.22119	91.04464	50.1433

Totals : 9491.23730 210.22935

Signal 2: DAD1 B, Sig=210,4 Ref=360,100

Peak #	RetTime [min]	Type	Width [min]	Area [mAU*s]	Height [mAU]	Area %
1	21.346	BB	0.5810	6386.45850	161.46416	49.8729
2	27.334	BB	0.7393	6419.00977	123.20667	50.1271

Totals : 1.28055e4 284.67082

Signal 3: DAD1 D, Sig=230,4 Ref=360,100

Peak #	RetTime [min]	Type	Width [min]	Area [mAU*s]	Height [mAU]	Area %
1	21.347	BB	0.5880	4811.69580	120.19372	50.2062
2	27.336	BB	0.7023	4772.16650	91.45810	49.7938

Totals : 9583.86230 211.65182

*** End of Report ***

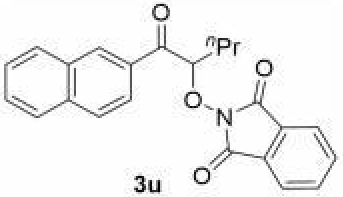

3u
racemic

Acq. Operator : SYSTEM Seq. Line : 3
Sample Operator : SYSTEM
Acq. Instrument : HPLC Location : P1-F-03
Injection Date : 1/5/2023 5:53:49 pm Inj : 1
Inj Volume : 2.000 µl
Different Inj Volume from Sample Entry! Actual Inj Volume : 1.000 µl
Acq. Method : C:\Users\Public\Documents\ChemStation\1\Data\SUN\SUN 2023-05-01 15-50-06 \IC3-30-60.M
Last changed : 10/11/2022 9:42:07 am by SYSTEM
Analysis Method : C:\Users\Public\Documents\ChemStation\1\Data\SUN\SUN 2023-05-01 15-50-06 \IC3-30-60.M (Sequence Method)
Last changed : 2/5/2023 10:07:43 pm by SYSTEM
(modified after loading)
Additional Info : Peak(s) manually integrated

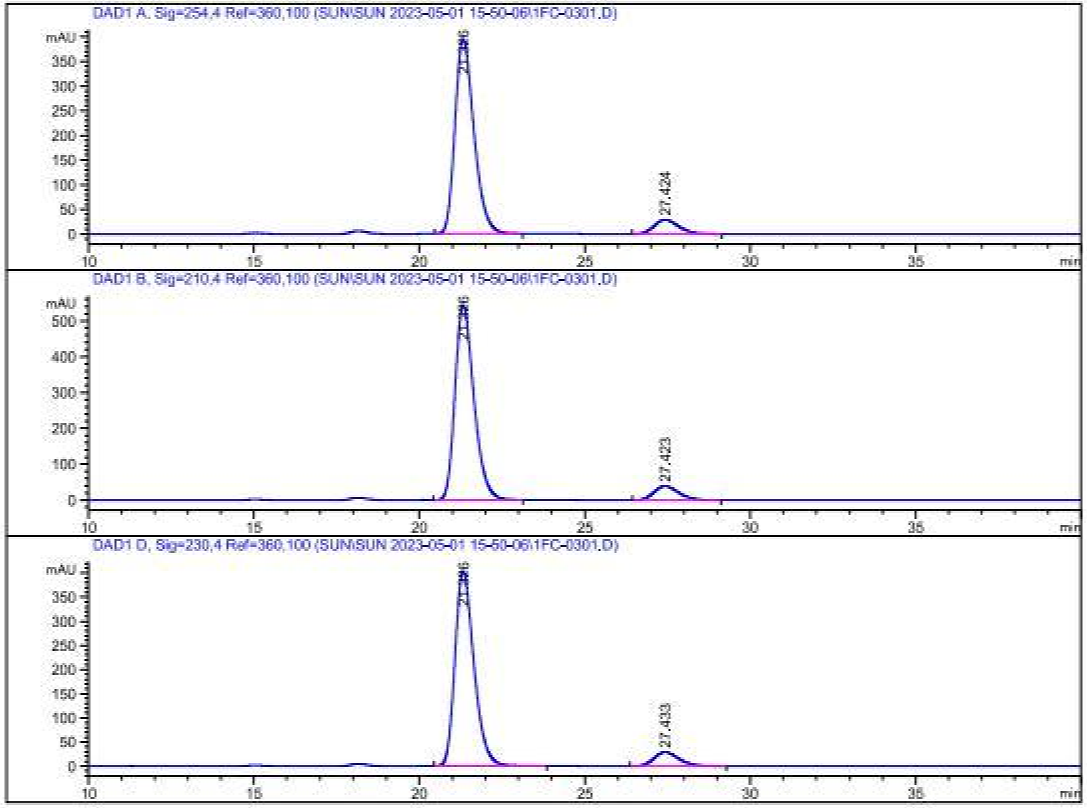

Data File C:\Users\P...Documents\ChemStation\1\Data\SUN\SUN 2023-05-01 15-50-06\1FC-0301.D
Sample Name: qcx-6-117b

Area Percent Report

Sorted By : Signal
Multiplier : 1.0000
Dilution : 1.0000
Use Multiplier & Dilution Factor with ISTDs

Signal 1: DAD1 A, Sig=254,4 Ref=360,100

Peak #	RetTime [min]	Type	Width [min]	Area [mAU*s]	Height [mAU]	Area %
1	21.326	BB	0.6073	1.57302e4	394.38947	91.1827
2	27.424	BB	0.6192	1521.09875	28.93819	8.8173

Totals : 1.72513e4 423.32766

Signal 2: DAD1 B, Sig=210,4 Ref=360,100

Peak #	RetTime [min]	Type	Width [min]	Area [mAU*s]	Height [mAU]	Area %
1	21.326	BB	0.6015	2.15594e4	543.30060	91.3181
2	27.423	BB	0.6175	2049.72437	39.14071	8.6819

Totals : 2.36091e4 582.44130

Signal 3: DAD1 D, Sig=230,4 Ref=360,100

Peak #	RetTime [min]	Type	Width [min]	Area [mAU*s]	Height [mAU]	Area %
1	21.326	BB	0.6076	1.60384e4	403.21442	91.2753
2	27.433	BB	0.6210	1533.05823	29.08266	8.7247

Totals : 1.75715e4 432.29708

*** End of Report ***

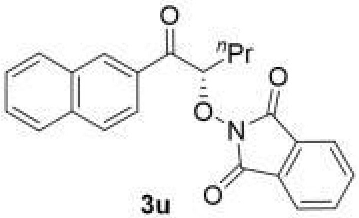

3u
enantioenriched

===

Acq. Operator : SYSTEM   Seq. Line : 2
Sample Operator : SYSTEM
Acq. Instrument : HPLC   Location : P1-F-04
Injection Date : 28/4/2023 7:08:08 pm   Inj : 1
Inj Volume : 2.000 µl
Different Inj Volume from Sample Entry! Actual Inj Volume : 12.000 µl
Acq. Method : C:\Users\Public\Documents\ChemStation\1\Data\SUN\SUN 2023-04-28 19-01-04\IC3-50-80.M
Last changed : 28/4/2023 6:59:37 pm by SYSTEM
Analysis Method : C:\Users\Public\Documents\ChemStation\1\Data\SUN\SUN 2023-04-28 19-01-04\IC3-50-80.M (Sequence Method)
Last changed : 2/5/2023 5:51:13 pm by SYSTEM
(modified after loading)
Additional Info : Peak(s) manually integrated

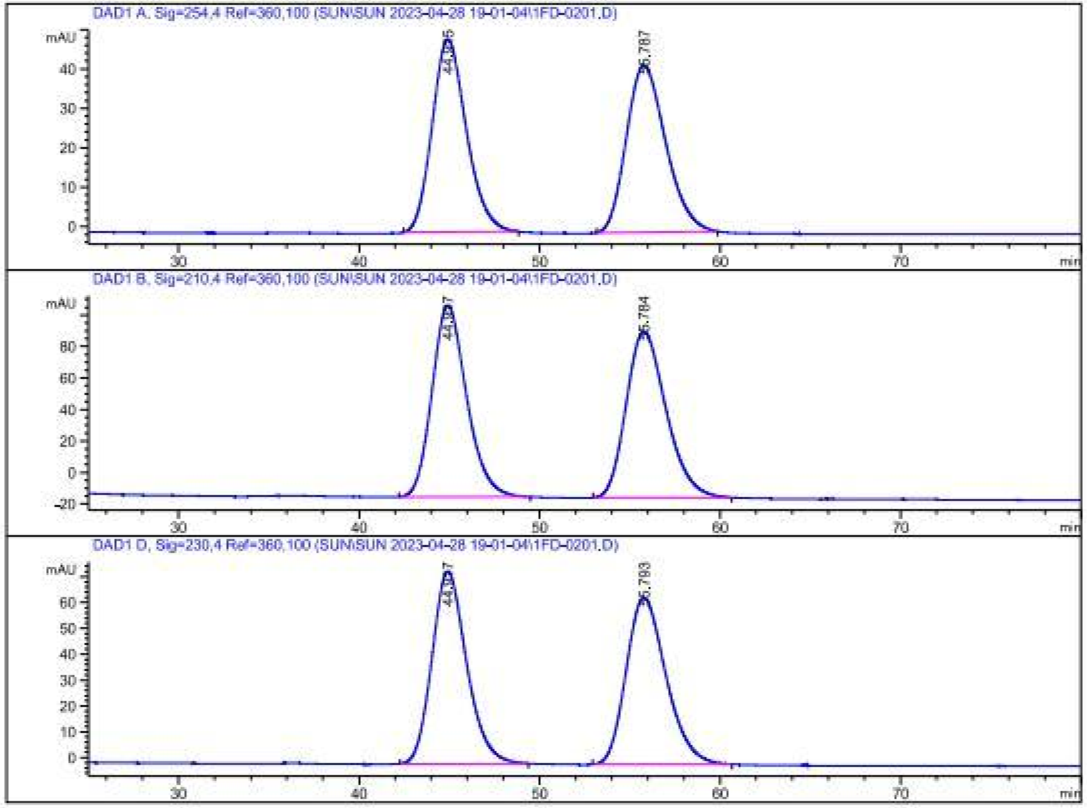

Data File C:\Users\P...Documents\ChemStation\1\Data\SUN\SUN 2023-04-28 19-01-04\1FD-0201.D
Sample Name: qcx-6-118c

===

Area Percent Report

===

Sorted By : Signal
Multiplier : 1.0000
Dilution : 1.0000
Use Multiplier & Dilution Factor with ISTDs

Signal 1: DAD1 A, Sig=254,4 Ref=360,100

Peak #	RetTime [min]	Type	Width [min]	Area [mAU*s]	Height [mAU]	Area %
1	44.915	BB	1.5440	6467.75439	48.98223	50.1796
2	55.787	BB	1.7736	6421.44824	42.32279	49.8204

Totals : 1.28892e4 91.30502

Signal 2: DAD1 B, Sig=210,4 Ref=360,100

Peak #	RetTime [min]	Type	Width [min]	Area [mAU*s]	Height [mAU]	Area %
1	44.917	BB	1.5605	1.62197e4	121.72761	50.0259
2	55.784	BB	1.7958	1.62029e4	105.43838	49.9741

Totals : 3.24226e4 227.16599

Signal 3: DAD1 D, Sig=230,4 Ref=360,100

Peak #	RetTime [min]	Type	Width [min]	Area [mAU*s]	Height [mAU]	Area %
1	44.917	BB	1.5567	9919.76367	74.46222	49.9985
2	55.793	BB	1.7977	9920.36621	64.50819	50.0015

Totals : 1.98401e4 138.97041

===

*** End of Report ***

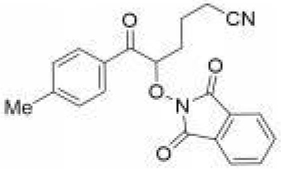

3v
racemic

===

Acq. Operator : SYSTEM Seq. Line : 11
Sample Operator : SYSTEM
Acq. Instrument : HPLC Location : P1-F-04
Injection Date : 2/5/2023 2:15:42 pm Inj : 1
Inj Volume : 2.000 µl
Different Inj Volume from Sample Entry! Actual Inj Volume : 0.500 µl
Acq. Method : C:\Users\Public\Documents\ChemStation\1\Data\SUN\SUN 2023-05-02 10-28-58\IC3-50-80.M
Last changed : 28/4/2023 6:59:37 pm by SYSTEM
Analysis Method : C:\Users\Public\Documents\ChemStation\1\Data\SUN\SUN 2023-05-02 10-28-58\IC3-50-80.M (Sequence Method)
Last changed : 2/5/2023 5:53:30 pm by SYSTEM
(modified after loading)
Additional Info : Peak(s) manually integrated

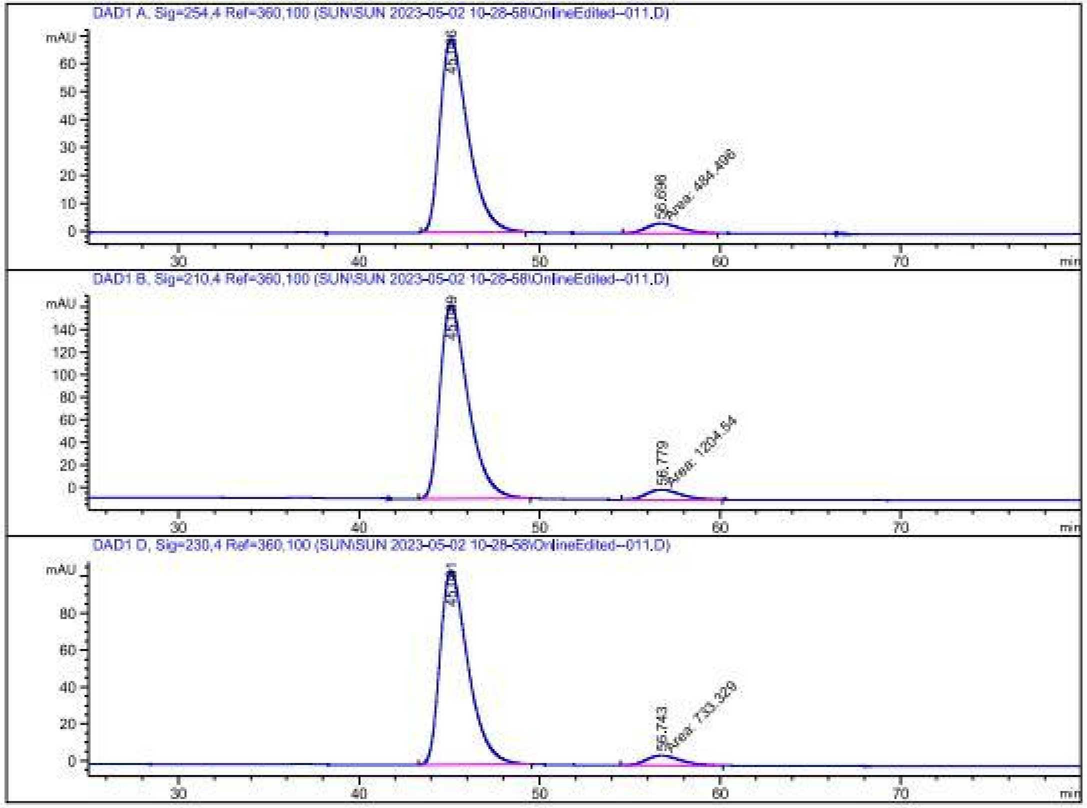

Data File C:\Users\P...\ChemStation\1\Data\SUN\SUN 2023-05-02 10-28-58\OnlineEdited--011.D
Sample Name: qcx-6-117c

===

Area Percent Report

===

Sorted By : Signal
Multiplier : 1.0000
Dilution : 1.0000
Use Multiplier & Dilution Factor with ISTDs

Signal 1: DAD1 A, Sig=254,4 Ref=360,100

Peak #	RetTime [min]	Type	Width [min]	Area [mAU*s]	Height [mAU]	Area %
1	45.086	BB	1.2591	7450.19238	69.19897	93.8940
2	56.696	MM	2.2260	484.49561	3.62748	6.1060

Totals : 7934.68799 72.82645

Signal 2: DAD1 B, Sig=210,4 Ref=360,100

Peak #	RetTime [min]	Type	Width [min]	Area [mAU*s]	Height [mAU]	Area %
1	45.089	BB	1.2680	1.85164e4	171.38390	93.8921
2	56.779	MM	2.2301	1204.54016	9.00195	6.1079

Totals : 1.97209e4 180.38585

Signal 3: DAD1 D, Sig=230,4 Ref=360,100

Peak #	RetTime [min]	Type	Width [min]	Area [mAU*s]	Height [mAU]	Area %
1	45.091	BB	1.2680	1.13433e4	104.90681	93.9277
2	56.743	MM	2.2365	733.32935	5.46492	6.0723

Totals : 1.20767e4 110.37174

===

*** End of Report ***

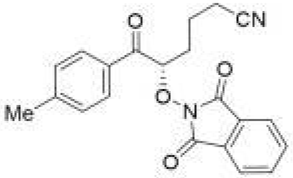

3v
enantioenriched

Acq. Operator : SYSTEM Seq. Line : 60
Sample Operator : SYSTEM
Acq. Instrument : HPLC Location : P2-A-05
Injection Date : 16/3/2023 10:19:40 pm Inj : 1
Inj Volume : 2.000 µl
Different Inj Volume from Sample Entry! Actual Inj Volume : 3.000 µl
Acq. Method : C:\Users\Public\Documents\ChemStation\1\Data\SUN\SUN 2023-03-15 22-36-05\IC3-50-60.M
Last changed : 12/2/2023 2:10:14 pm by SYSTEM
Analysis Method : C:\Users\Public\Documents\ChemStation\1\Data\SUN\SUN 2023-03-15 22-36-05\IC3-50-60.M (Sequence Method)
Last changed : 23/3/2023 9:00:29 pm by SYSTEM
(modified after loading)
Additional Info : Peak(s) manually integrated

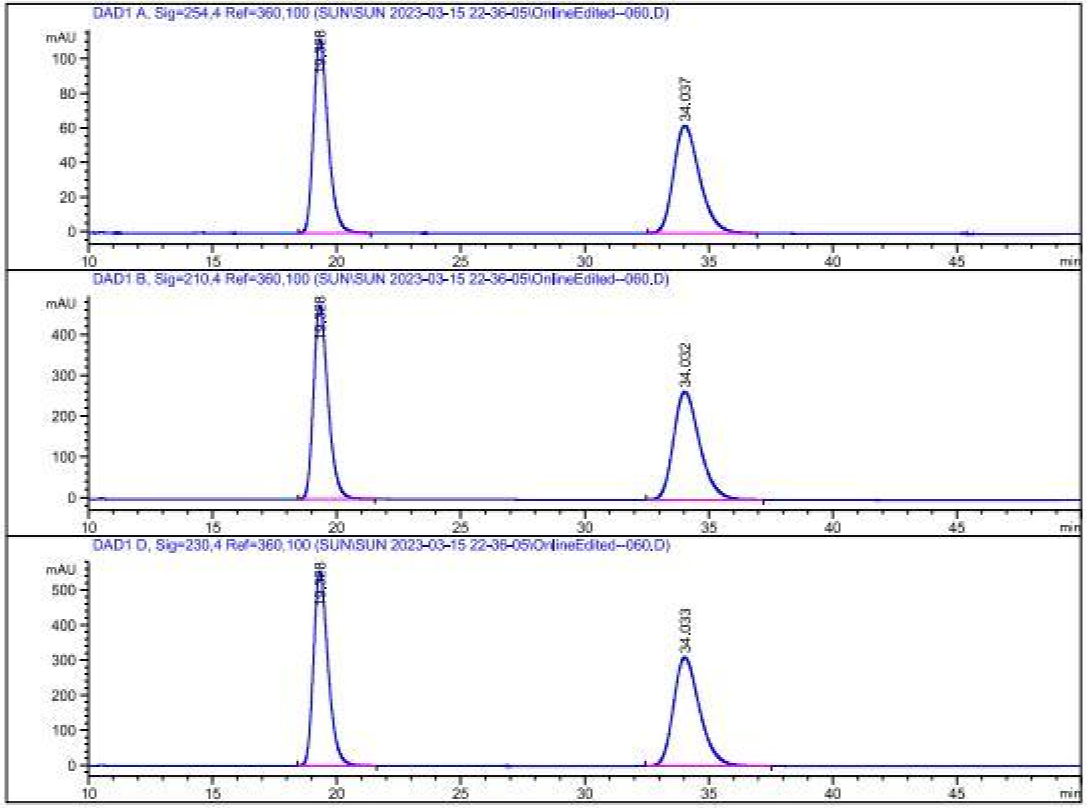

Data File C:\Users\P...\ChemStation\1\Data\SUN\SUN 2023-03-15 22-36-05\OnlineEdited--060.D
Sample Name: qcx-6-61c

Area Percent Report

Sorted By : Signal
Multiplier : 1.0000
Dilution : 1.0000
Use Multiplier & Dilution Factor with ISTDs

Signal 1: DAD1 A, Sig=254,4 Ref=360,100

Peak #	RetTime [min]	Type	Width [min]	Area [mAU*s]	Height [mAU]	Area %
1	19.328	BB	0.6139	4547.14063	112.06439	50.0347
2	34.037	BB	0.9251	4540.82959	62.20716	49.9653

Totals : 9087.97021 174.27156

Signal 2: DAD1 B, Sig=210,4 Ref=360,100

Peak #	RetTime [min]	Type	Width [min]	Area [mAU*s]	Height [mAU]	Area %
1	19.328	BB	0.6097	1.93138e4	476.16357	49.9637
2	34.032	BB	0.9823	1.93418e4	264.92584	50.0363

Totals : 3.86556e4 741.08942

Signal 3: DAD1 D, Sig=230,4 Ref=360,100

Peak #	RetTime [min]	Type	Width [min]	Area [mAU*s]	Height [mAU]	Area %
1	19.328	BB	0.6181	2.25343e4	554.46692	49.8839
2	34.033	BB	1.0595	2.26391e4	309.47491	50.1161

Totals : 4.51734e4 863.94183

*** End of Report ***

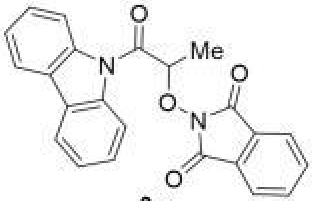

3w
racemic

Acq. Operator : SYSTEM Seq. Line : 55
Sample Operator : SYSTEM
Acq. Instrument : HPLC Location : P1-F-04
Injection Date : 18/4/2023 9:52:59 am Inj : 1
Inj Volume : 2.000 µl
Different Inj Volume from Sample Entry! Actual Inj Volume : 1.000 µl
Acq. Method : C:\Users\Public\Documents\ChemStation\1\Data\SUN\SUN 2023-04-17 10-34-33\IC3-50-60.M
Last changed : 12/2/2023 2:10:14 pm by SYSTEM
Analysis Method : C:\Users\Public\Documents\ChemStation\1\Data\SUN\SUN 2023-04-17 10-34-33\IC3-50-60.M (Sequence Method)
Last changed : 18/4/2023 2:20:50 pm by SYSTEM
(modified after loading)
Additional Info : Peak(s) manually integrated

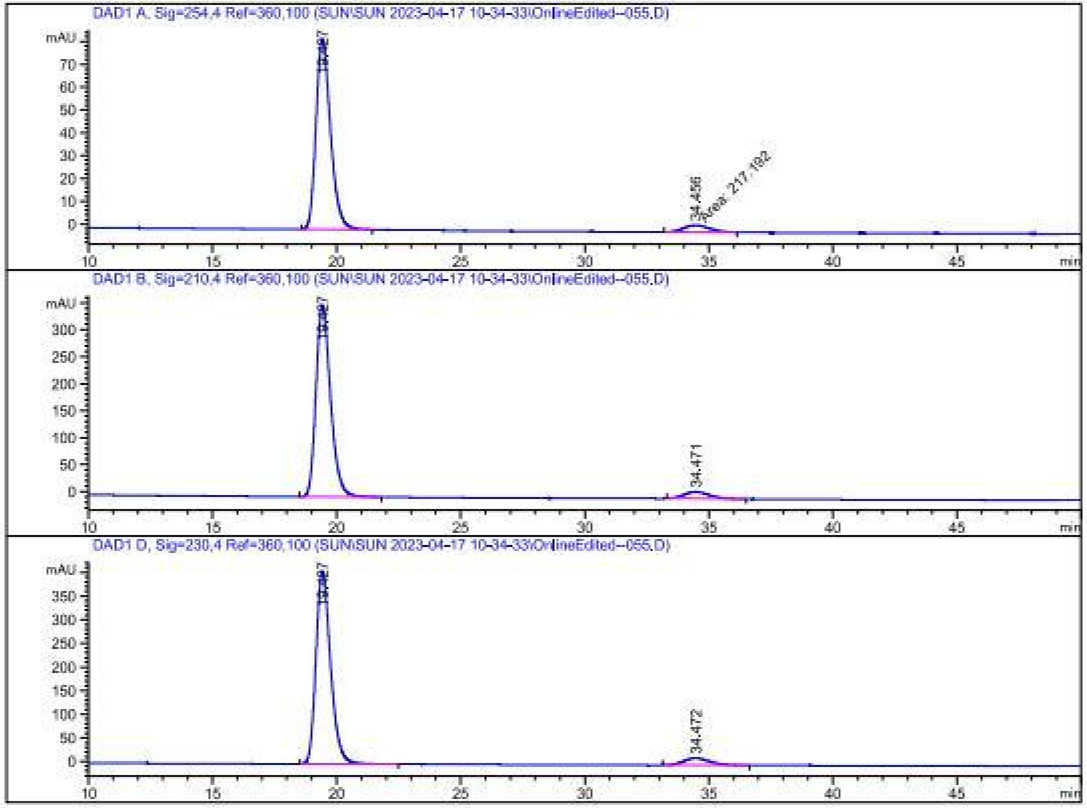

Data File C:\Users\P...\ChemStation\1\Data\SUN\SUN 2023-04-17 10-34-33\OnlineEdited--055.D
Sample Name: qcx-6-82a

Area Percent Report

Sorted By : Signal
Multiplier : 1.0000
Dilution : 1.0000
Use Multiplier & Dilution Factor with ISTDs

Signal 1: DAD1 A, Sig=254,4 Ref=360,100

Peak #	RetTime [min]	Type	Width [min]	Area [mAU*s]	Height [mAU]	Area %
1	19.427	BB	0.5914	3389.72705	83.36713	93.9784
2	34.456	MM	1.2015	217.19249	3.01290	6.0216
Totals :				3606.91954	86.38002	

Signal 2: DAD1 B, Sig=210,4 Ref=360,100

Peak #	RetTime [min]	Type	Width [min]	Area [mAU*s]	Height [mAU]	Area %
1	19.427	BB	0.6181	1.44579e4	355.01663	94.0106
2	34.471	BB	0.8420	921.11041	12.80893	5.9894
Totals :				1.53790e4	367.82556	

Signal 3: DAD1 D, Sig=230,4 Ref=360,100

Peak #	RetTime [min]	Type	Width [min]	Area [mAU*s]	Height [mAU]	Area %
1	19.427	BB	0.6171	1.66484e4	407.54837	93.8660
2	34.472	BB	0.8553	1087.93884	14.89248	6.1340
Totals :				1.77363e4	422.44085	

*** End of Report ***

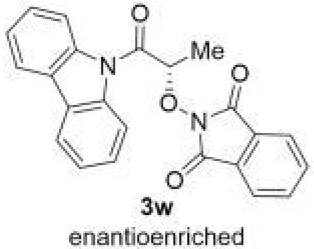

3w
enantioenriched

Acq. Operator : SYSTEM Seq. Line : 12
Sample Operator : SYSTEM
Acq. Instrument : HPLC Location : P2-A-04
Injection Date : 20/5/2023 1:42:36 pm Inj : 1
Inj Volume : 2.000 µl
Different Inj Volume from Sample Entry! Actual Inj Volume : 8.000 µl
Acq. Method : C:\Users\Public\Documents\ChemStation\1\Data\SUN\SUN 2023-05-20 10-09-26\IC3-50-60.M
Last changed : 12/2/2023 2:10:14 pm by SYSTEM
Analysis Method : C:\Users\Public\Documents\ChemStation\1\Data\SUN\SUN 2023-05-20 10-09-26\IC3-50-60.M (Sequence Method)
Last changed : 27/5/2023 4:32:34 pm by SYSTEM
(modified after loading)
Additional Info : Peak(s) manually integrated

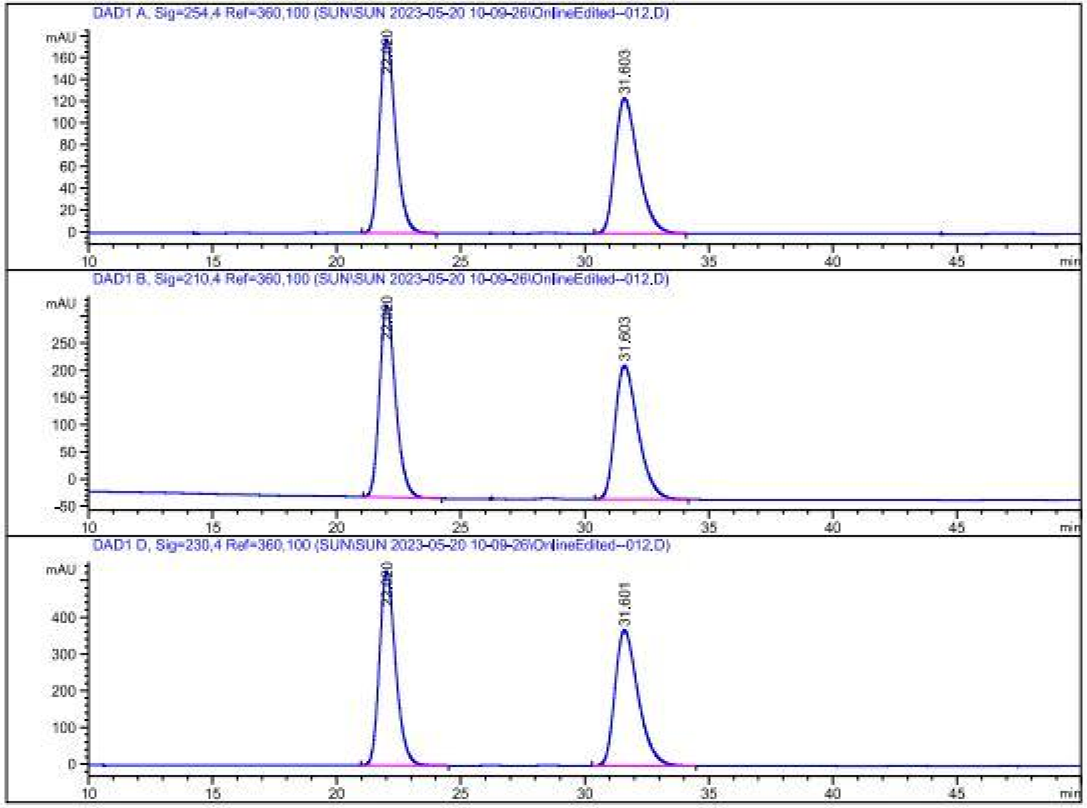

Data File C:\Users\P...\ChemStation\1\Data\SUN\SUN 2023-05-20 10-09-26\OnlineEdited--012.D
Sample Name: qcx-6-130a

Area Percent Report

Sorted By : Signal
Multiplier : 1.0000
Dilution : 1.0000
Use Multiplier & Dilution Factor with ISTDs

Signal 1: DAD1 A, Sig=254,4 Ref=360,100

Peak #	RetTime [min]	Type	Width [min]	Area [mAU*s]	Height [mAU]	Area %
1	22.020	BB	0.6635	8045.64160	178.11681	49.9917
2	31.603	BB	0.8317	8048.32373	124.24981	50.0083

Totals : 1.60940e4 302.36662

Signal 2: DAD1 B, Sig=210,4 Ref=360,100

Peak #	RetTime [min]	Type	Width [min]	Area [mAU*s]	Height [mAU]	Area %
1	22.020	BB	0.6593	1.59254e4	353.69705	49.9546
2	31.603	BB	0.9139	1.59544e4	246.55710	50.0454

Totals : 3.18798e4 600.25415

Signal 3: DAD1 D, Sig=230,4 Ref=360,100

Peak #	RetTime [min]	Type	Width [min]	Area [mAU*s]	Height [mAU]	Area %
1	22.020	BB	0.6774	2.38908e4	528.50366	49.9702
2	31.601	BB	0.9725	2.39193e4	368.64725	50.0298

Totals : 4.78101e4 897.15091

*** End of Report ***

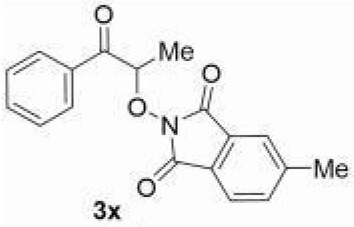

3x
racemic

===

Acq. Operator : SYSTEM   Seq. Line : 45
Sample Operator : SYSTEM
Acq. Instrument : HPLC   Location : P2-A-01
Injection Date : 26/5/2023 6:33:46 am   Inj : 1
Inj Volume : 2.000 µl
Different Inj Volume from Sample Entry! Actual Inj Volume : 5.000 µl
Acq. Method : C:\Users\Public\Documents\ChemStation\1\Data\SUN\SUN 2023-05-25 15-12-14
\IC3-50-60.M
Last changed : 12/2/2023 2:10:14 pm by SYSTEM
Analysis Method : C:\Users\Public\Documents\ChemStation\1\Data\SUN\SUN 2023-05-25 15-12-14
\IC3-50-60.M (Sequence Method)
Last changed : 27/5/2023 4:29:51 pm by SYSTEM
(modified after loading)
Additional Info : Peak(s) manually integrated

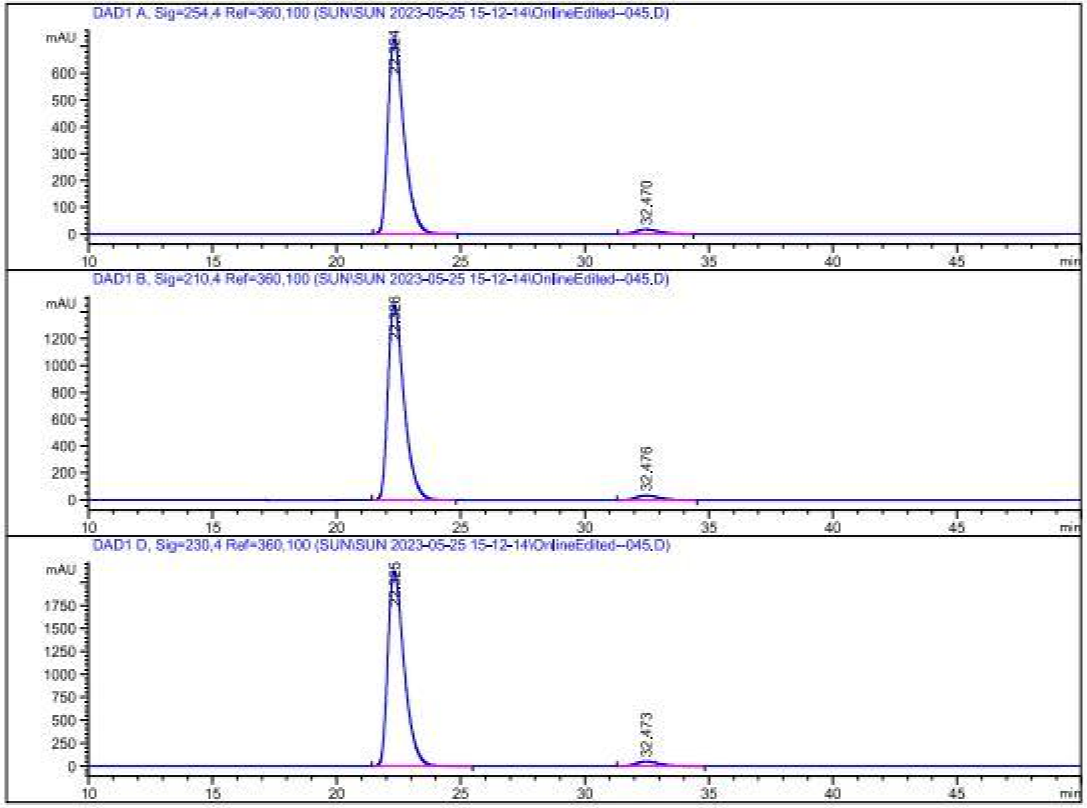

Data File C:\Users\P...\ChemStation\1\Data\SUN\SUN 2023-05-25 15-12-14\OnlineEdited--045.D
Sample Name: qcx-6-131a

===

Area Percent Report

===

Sorted By : Signal
Multiplier : 1.0000
Dilution : 1.0000
Use Multiplier & Dilution Factor with ISTDs

Signal 1: DAD1 A, Sig=254,4 Ref=360,100

Peak #	RetTime [min]	Type	Width [min]	Area [mAU*s]	Height [mAU]	Area %
1	22.324	BB	0.6812	3.28334e4	727.25348	96.6890
2	32.470	BB	0.7460	1124.32886	17.71380	3.3110

Totals : 3.39578e4 744.96728

Signal 2: DAD1 B, Sig=210,4 Ref=360,100

Peak #	RetTime [min]	Type	Width [min]	Area [mAU*s]	Height [mAU]	Area %
1	22.326	BB	0.6467	6.54258e4	1453.35046	96.6869
2	32.476	BB	0.7470	2241.89624	35.15664	3.3131

Totals : 6.76677e4 1488.50711

Signal 3: DAD1 D, Sig=230,4 Ref=360,100

Peak #	RetTime [min]	Type	Width [min]	Area [mAU*s]	Height [mAU]	Area %
1	22.325	BB	0.6294	9.65870e4	2122.34839	96.6259
2	32.473	BB	0.7615	3372.69263	52.67359	3.3741

Totals : 9.99597e4 2175.02198

===

*** End of Report ***

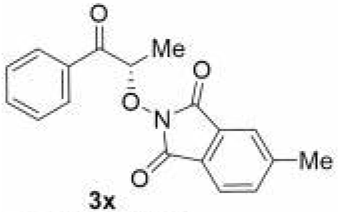

3x
enantioenriched

===

Acq. Operator : SYSTEM Seq. Line : 46
Sample Operator : SYSTEM
Acq. Instrument : HPLC Location : P2-A-05
Injection Date : 20/5/2023 6:48:18 am Inj : 1
Inj Volume : 2.000 µl
Different Inj Volume from Sample Entry! Actual Inj Volume : 8.000 µl
Acq. Method : C:\Users\Public\Documents\ChemStation\1\Data\SUN\SUN 2023-05-19 09-28-34\IC3-30-60.M
Last changed : 10/11/2022 9:42:07 am by SYSTEM
Analysis Method : C:\Users\Public\Documents\ChemStation\1\Data\SUN\SUN 2023-05-19 09-28-34\IC3-30-60.M (Sequence Method)
Last changed : 27/5/2023 2:58:56 pm by SYSTEM
(modified after loading)
Additional Info : Peak(s) manually integrated

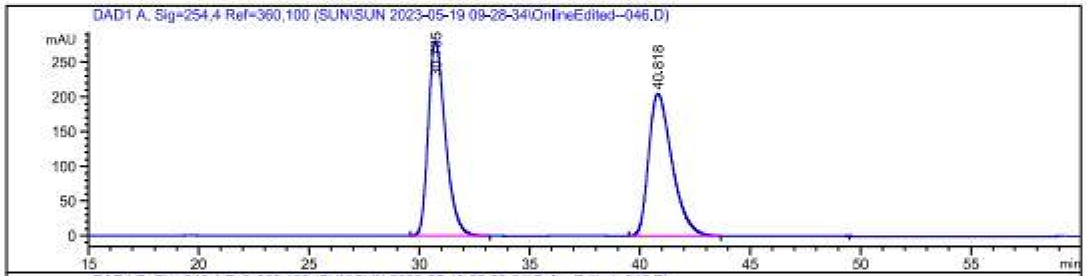

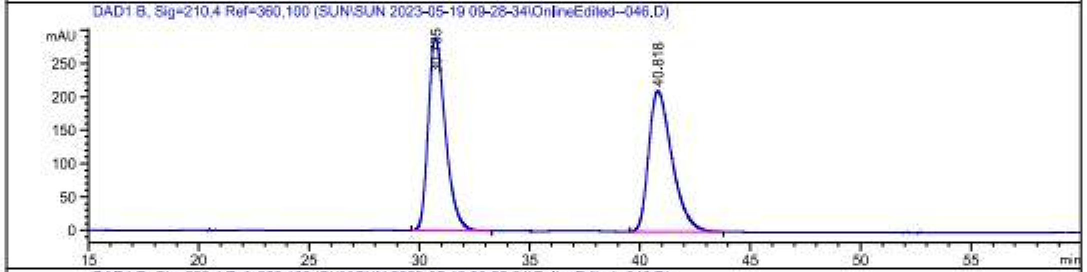

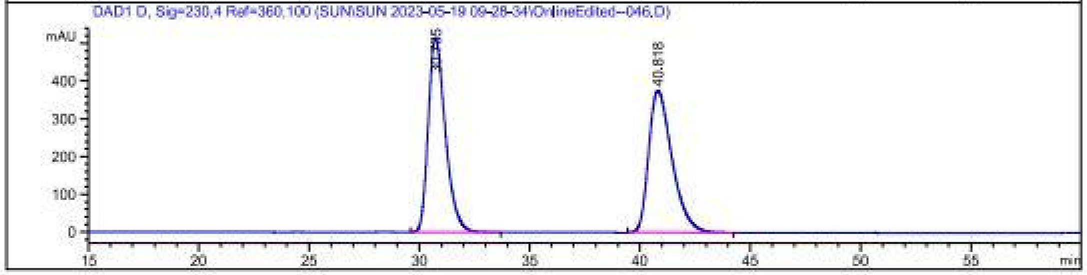

Data File C:\Users\P...\ChemStation\1\Data\SUN\SUN 2023-05-19 09-28-34\OnlineEdited--046.D
Sample Name: qcx-6-130b

===

Area Percent Report

===

Sorted By : Signal
Multiplier : 1.0000
Dilution : 1.0000
Use Multiplier & Dilution Factor with ISTDs

Signal 1: DAD1 A, Sig=254,4 Ref=360,100

Peak #	RetTime [min]	Type	Width [min]	Area [mAU*s]	Height [mAU]	Area %
1	30.735	BB	0.7914	1.51448e4	280.30093	50.0549
2	40.818	BB	1.0016	1.51116e4	204.85355	49.9451

Totals : 3.02564e4 485.15448

Signal 2: DAD1 B, Sig=210,4 Ref=360,100

Peak #	RetTime [min]	Type	Width [min]	Area [mAU*s]	Height [mAU]	Area %
1	30.735	BB	0.7925	1.55921e4	288.96167	50.1165
2	40.818	BB	0.9891	1.55197e4	210.63760	49.8835

Totals : 3.11118e4 499.59927

Signal 3: DAD1 D, Sig=230,4 Ref=360,100

Peak #	RetTime [min]	Type	Width [min]	Area [mAU*s]	Height [mAU]	Area %
1	30.735	BB	0.8176	2.78140e4	515.39532	50.0329
2	40.818	BB	1.0638	2.77774e4	376.24643	49.9671

Totals : 5.55914e4 891.64175

===

*** End of Report ***

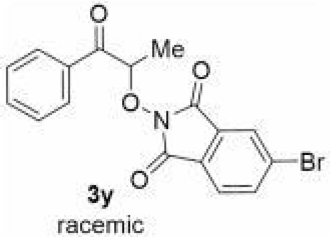

3y
racemic

===

Acq. Operator : SYSTEM Seq. Line : 43
Sample Operator : SYSTEM
Acq. Instrument : HPLC Location : P2-A-02
Injection Date : 26/5/2023 5:21:43 am Inj : 1
Inj Volume : 2.000 µl
Different Inj Volume from Sample Entry! Actual Inj Volume : 5.000 µl
Acq. Method : C:\Users\Public\Documents\ChemStation\1\Data\SUN\SUN 2023-05-25 15-12-14 \IC3-30-60.M
Last changed : 10/11/2022 9:42:07 am by SYSTEM
Analysis Method : C:\Users\Public\Documents\ChemStation\1\Data\SUN\SUN 2023-05-25 15-12-14 \IC3-30-60.M (Sequence Method)
Last changed : 27/5/2023 4:29:04 pm by SYSTEM
(modified after loading)
Additional Info : Peak(s) manually integrated

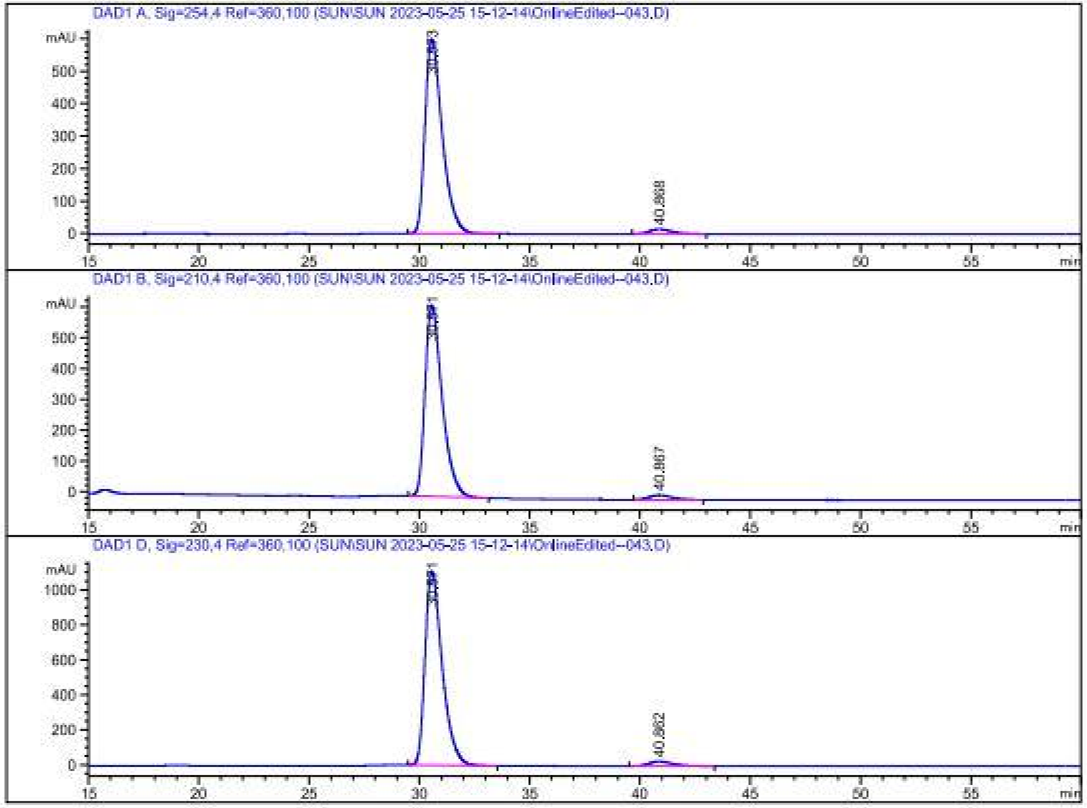

Data File C:\Users\P...\ChemStation\1\Data\SUN\SUN 2023-05-25 15-12-14\OnlineEdited--043.D
Sample Name: qcx-6-131b

===
Area Percent Report
===

Sorted By : Signal
Multiplier : 1.0000
Dilution : 1.0000
Use Multiplier & Dilution Factor with ISTDs

Signal 1: DAD1 A, Sig=254,4 Ref=360,100

Peak #	RetTime [min]	Type	Width [min]	Area [mAU*s]	Height [mAU]	Area %
1	30.573	BB	0.8103	3.25258e4	599.15204	96.8367
2	40.868	BB	0.8345	1062.50793	14.90840	3.1633
Totals :				3.35883e4	614.06044	

Signal 2: DAD1 B, Sig=210,4 Ref=360,100

Peak #	RetTime [min]	Type	Width [min]	Area [mAU*s]	Height [mAU]	Area %
1	30.571	BB	0.7743	3.34924e4	621.39526	96.9148
2	40.867	BB	0.8242	1066.20715	15.14835	3.0852
Totals :				3.45586e4	636.54361	

Signal 3: DAD1 D, Sig=230,4 Ref=360,100

Peak #	RetTime [min]	Type	Width [min]	Area [mAU*s]	Height [mAU]	Area %
1	30.571	BB	0.8071	5.98164e4	1107.47510	96.8103
2	40.862	BB	0.8439	1970.84448	27.34414	3.1897
Totals :				6.17873e4	1134.81923	

===
*** End of Report ***

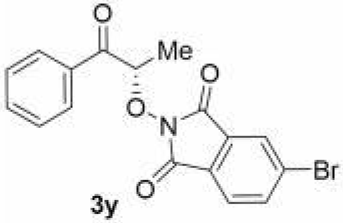

3y
enantioenriched

Acq. Operator : SYSTEM Seq. Line : 2
Sample Operator : SYSTEM
Acq. Instrument : HPLC Location : P1-F-06
Injection Date : 19/4/2023 10:06:13 pm Inj : 1
Inj Volume : 2.000 µl
Different Inj Volume from Sample Entry! Actual Inj Volume : 3.000 µl
Acq. Method : C:\Users\Public\Documents\ChemStation\1\Data\SUN\SUN 2023-04-19 21-58-27\IC3-50-60.M
Last changed : 12/2/2023 2:10:14 pm by SYSTEM
Analysis Method : C:\Users\Public\Documents\ChemStation\1\Data\SUN\SUN 2023-04-19 21-58-27\IC3-50-60.M (Sequence Method)
Last changed : 20/4/2023 1:44:32 pm by SYSTEM
(modified after loading)
Additional Info : Peak(s) manually integrated

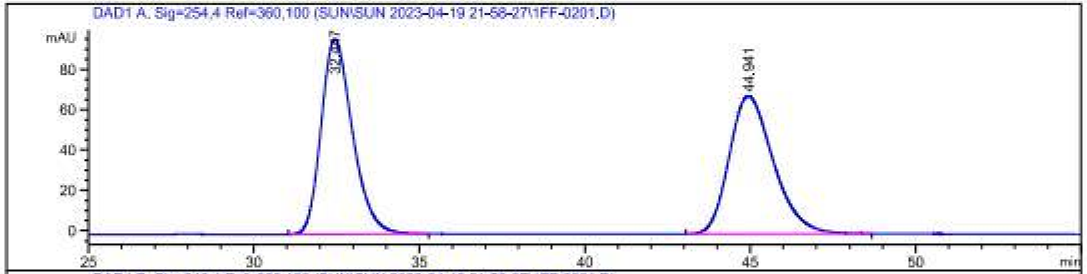

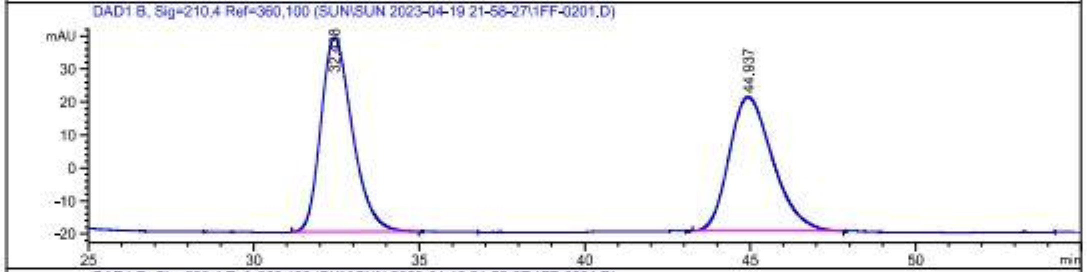

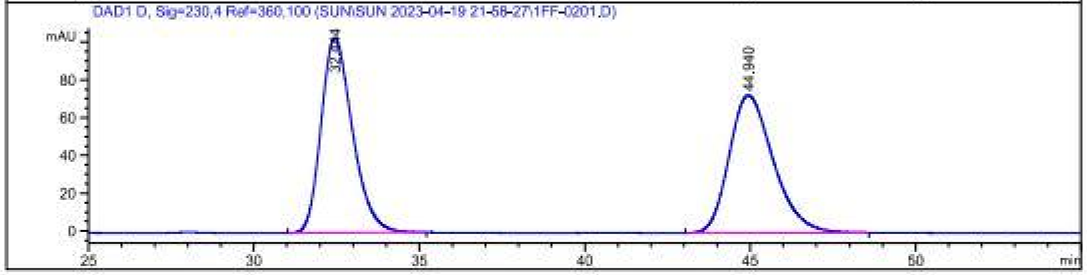

Data File C:\Users\P...Documents\ChemStation\1\Data\SUN\SUN 2023-04-19 21-58-27\1FF-0201.D
Sample Name: qcx-6-107b

Area Percent Report

Sorted By : Signal
Multiplier : 1.0000
Dilution : 1.0000
Use Multiplier & Dilution Factor with ISTDs

Signal 1: DAD1 A, Sig=254,4 Ref=360,100

Peak #	RetTime [min]	Type	Width [min]	Area [mAU*s]	Height [mAU]	Area %
1	32.447	BB	0.9263	6393.29736	96.70048	50.0941
2	44.941	BB	1.0959	6369.28955	68.43258	49.9059

Totals : 1.27626e4 165.13306

Signal 2: DAD1 B, Sig=210,4 Ref=360,100

Peak #	RetTime [min]	Type	Width [min]	Area [mAU*s]	Height [mAU]	Area %
1	32.438	BB	0.8342	3906.78979	58.94890	51.0904
2	44.937	BB	1.0810	3740.02417	40.74208	48.9096

Totals : 7646.81396 99.69099

Signal 3: DAD1 D, Sig=230,4 Ref=360,100

Peak #	RetTime [min]	Type	Width [min]	Area [mAU*s]	Height [mAU]	Area %
1	32.444	BB	0.9641	6827.01855	103.22570	50.1967
2	44.940	BB	1.0950	6773.50146	72.83440	49.8033

Totals : 1.36005e4 176.06010

*** End of Report ***

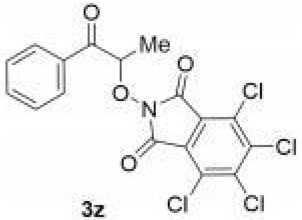

3z
racemic

===

Acq. Operator : SYSTEM Seq. Line : 20
Sample Operator : SYSTEM
Acq. Instrument : HPLC Location : P1-F-03
Injection Date : 23/4/2023 4:38:38 am Inj : 1
Inj Volume : 2.000 µl
Different Inj Volume from Sample Entry! Actual Inj Volume : 3.000 µl
Acq. Method : C:\Users\Public\Documents\ChemStation\1\Data\SUN\SUN 2023-04-22 20-30-51\IC3-50-60.M
Last changed : 12/2/2023 2:10:14 pm by SYSTEM
Analysis Method : C:\Users\Public\Documents\ChemStation\1\Data\SUN\SUN 2023-04-22 20-30-51\IC3-50-60.M (Sequence Method)
Last changed : 24/4/2023 12:44:25 pm by SYSTEM
(modified after loading)
Additional Info : Peak(s) manually integrated

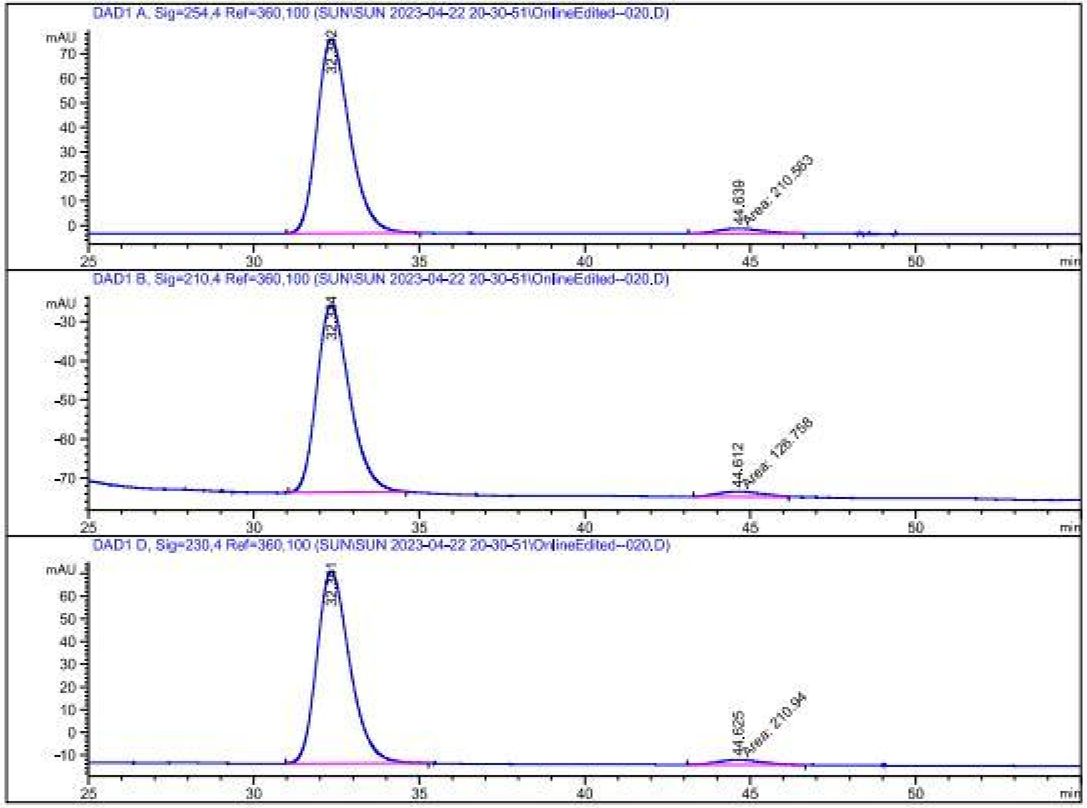

Data File C:\Users\P...\ChemStation\1\Data\SUN\SUN 2023-04-22 20-30-51\OnlineEdited--020.D
Sample Name: qcx-6-107a

===
Area Percent Report
===

Sorted By : Signal
Multiplier : 1.0000
Dilution : 1.0000
Use Multiplier & Dilution Factor with ISTDs

Signal 1: DAD1 A, Sig=254,4 Ref=360,100

Peak #	RetTime [min]	Type	Width [min]	Area [mAU*s]	Height [mAU]	Area %
1	32.342	BB	0.8571	5453.69385	79.22380	96.2826
2	44.639	MM	1.6289	210.56343	2.15449	3.7174

Totals : 5664.25728 81.37829

Signal 2: DAD1 B, Sig=210,4 Ref=360,100

Peak #	RetTime [min]	Type	Width [min]	Area [mAU*s]	Height [mAU]	Area %
1	32.334	BB	0.8117	3300.59766	47.79354	96.3016
2	44.612	MM	1.5804	126.75761	1.33680	3.6984

Totals : 3427.35526 49.13035

Signal 3: DAD1 D, Sig=230,4 Ref=360,100

Peak #	RetTime [min]	Type	Width [min]	Area [mAU*s]	Height [mAU]	Area %
1	32.341	BB	0.8770	5863.07764	84.81594	96.5272
2	44.625	MM	1.5730	210.94017	2.23499	3.4728

Totals : 6074.01781 87.05093

===
*** End of Report ***

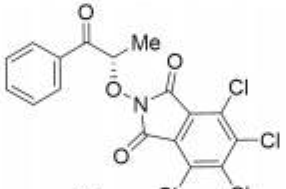

3z
enantioenriched

===

Acq. Operator : SYSTEM Seq. Line : 40
Sample Operator : SYSTEM
Acq. Instrument : HPLC Location : P2-B-06
Injection Date : 19/3/2023 5:59:43 am Inj : 1
Inj Volume : 2.000 µl
Different Inj Volume from Sample Entry! Actual Inj Volume : 8.000 µl
Acq. Method : C:\Users\Public\Documents\ChemStation\1\Data\SUN\SUN 2023-03-18 13-24-33 \IC3-70-60.M
Last changed : 17/3/2023 4:30:22 pm by SYSTEM
Analysis Method : C:\Users\Public\Documents\ChemStation\1\Data\SUN\SUN 2023-03-18 13-24-33 \IC3-70-60.M (Sequence Method)
Last changed : 19/3/2023 8:51:01 pm by SYSTEM
(modified after loading)
Additional Info : Peak(s) manually integrated

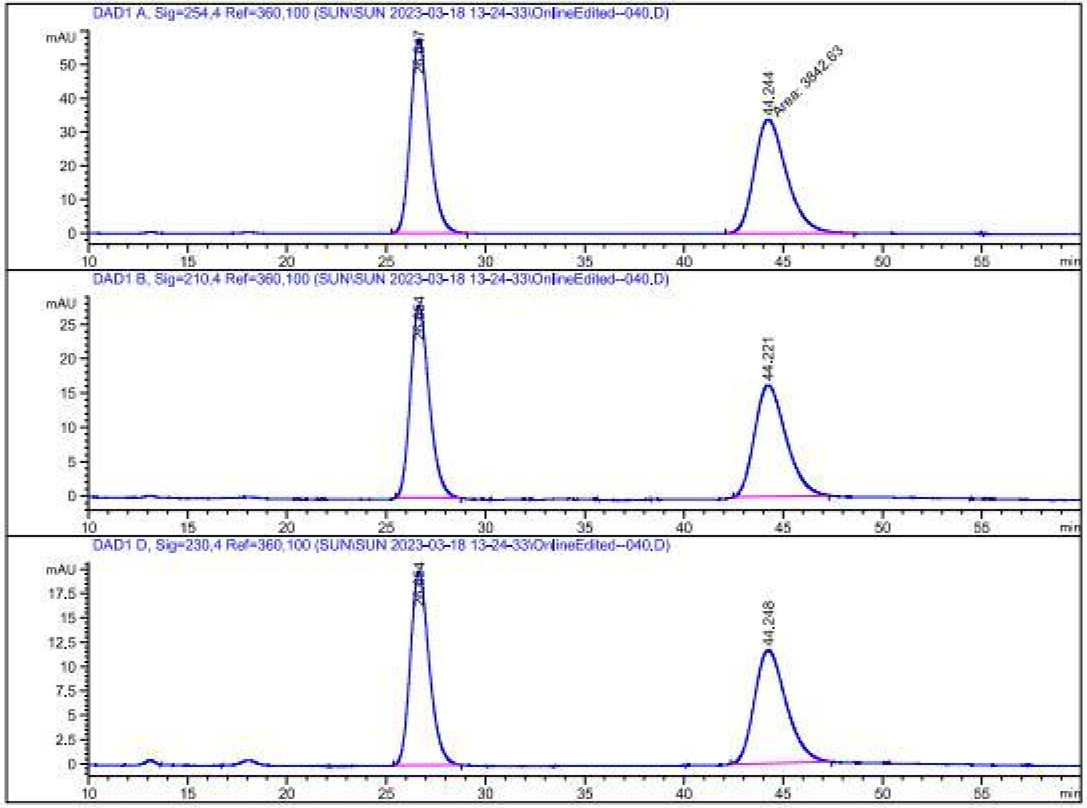

Data File C:\Users\P...\ChemStation\1\Data\SUN\SUN 2023-03-18 13-24-33\OnlineEdited--040.D
Sample Name: qcx-6-67b

===

Area Percent Report

===

Sorted By : Signal
Multiplier : 1.0000
Dilution : 1.0000
Use Multiplier & Dilution Factor with ISTDs

Signal 1: DAD1 A, Sig=254,4 Ref=360,100

Peak #	RetTime [min]	Type	Width [min]	Area [mAU*s]	Height [mAU]	Area %
1	26.647	BB	0.8062	3815.98511	57.58124	49.8260
2	44.244	MM	1.8949	3842.63428	33.79873	50.1740

Totals : 7658.61938 91.37997

Signal 2: DAD1 B, Sig=210,4 Ref=360,100

Peak #	RetTime [min]	Type	Width [min]	Area [mAU*s]	Height [mAU]	Area %
1	26.654	BB	0.7700	1839.98511	28.07950	50.6876
2	44.221	BB	1.2876	1790.06750	16.27000	49.3124

Totals : 3630.05261 44.34951

Signal 3: DAD1 D, Sig=230,4 Ref=360,100

Peak #	RetTime [min]	Type	Width [min]	Area [mAU*s]	Height [mAU]	Area %
1	26.654	BB	0.7741	1314.20203	19.93640	50.3978
2	44.248	BB	1.3058	1293.45715	11.62333	49.6022

Totals : 2607.65918 31.55973

===

*** End of Report ***

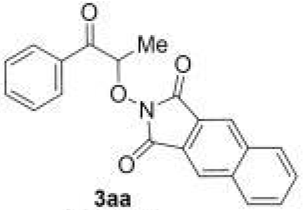

3aa
racemic

===

Acq. Operator : SYSTEM   Seq. Line : 6
Sample Operator : SYSTEM
Acq. Instrument : HPLC   Location : P2-B-05
Injection Date : 19/3/2023 5:00:46 pm   Inj : 1
Inj Volume : 2.000 µl
Different Inj Volume from Sample Entry! Actual Inj Volume : 1.000 µl
Acq. Method : C:\Users\Public\Documents\ChemStation\1\Data\SUN\SUN 2023-03-19 14-33-28\IC3-70-60.M
Last changed : 17/3/2023 4:30:22 pm by SYSTEM
Analysis Method : C:\Users\Public\Documents\ChemStation\1\Data\SUN\SUN 2023-03-19 14-33-28\IC3-70-60.M (Sequence Method)
Last changed : 19/3/2023 8:46:46 pm by SYSTEM
(modified after loading)
Additional Info : Peak(s) manually integrated

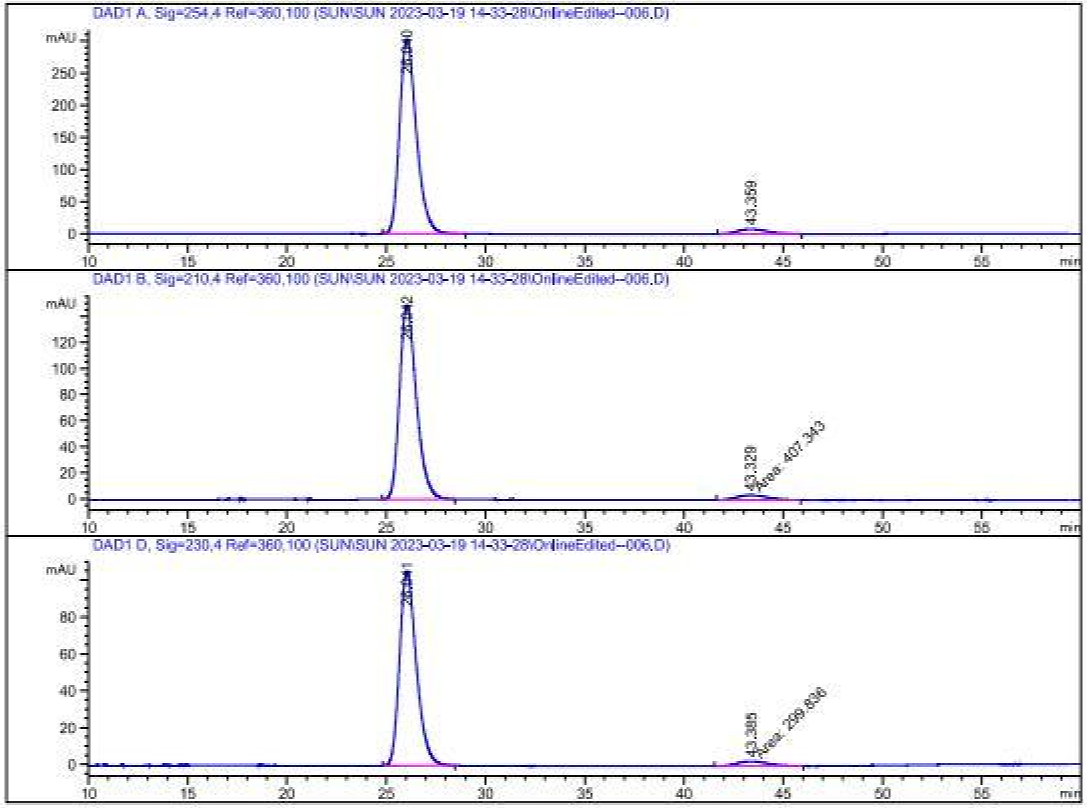

Data File C:\Users\P...\ChemStation\1\Data\SUN\SUN 2023-03-19 14-33-28\OnlineEdited--006.D
Sample Name: qcx-6-59a

===

Area Percent Report

===

Sorted By : Signal
Multiplier : 1.0000
Dilution : 1.0000
Use Multiplier & Dilution Factor with ISTDs

Signal 1: DAD1 A, Sig=254,4 Ref=360,100

Peak #	RetTime [min]	Type	Width [min]	Area [mAU*s]	Height [mAU]	Area %
1	26.040	BB	0.8778	1.79242e4	303.14230	95.6913
2	43.359	BB	1.2211	807.07910	7.73981	4.3087

Totals : 1.87312e4 310.88211

Signal 2: DAD1 B, Sig=210,4 Ref=360,100

Peak #	RetTime [min]	Type	Width [min]	Area [mAU*s]	Height [mAU]	Area %
1	26.042	BB	0.8146	8772.21289	148.84674	95.5625
2	43.329	MM	1.7745	407.34314	3.82584	4.4375

Totals : 9179.55603 152.67258

Signal 3: DAD1 D, Sig=230,4 Ref=360,100

Peak #	RetTime [min]	Type	Width [min]	Area [mAU*s]	Height [mAU]	Area %
1	26.041	BB	0.8201	6212.20459	105.39937	95.3957
2	43.385	MM	1.8147	299.83618	2.75374	4.6043

Totals : 6512.04077 108.15311

===

*** End of Report ***

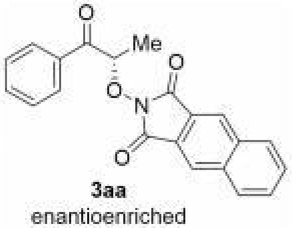

3aa
enantioenriched

```
=====================================================================
Acq. Operator   : SYSTEM                         Seq. Line :  34
Sample Operator : SYSTEM
Acq. Instrument : HPLC                            Location :   P2-B-02
Injection Date  : 19/3/2023 1:58:36 am                 Inj :   1
                                               Inj Volume : 2.000 µl
Different Inj Volume from Sample Entry!  Actual Inj Volume : 5.000 µl
Acq. Method     : C:\Users\Public\Documents\ChemStation\1\Data\SUN\SUN 2023-03-18 13-24-33
                  \IC3-30-40.M
Last changed    : 27/2/2023 10:13:57 pm by SYSTEM
Analysis Method : C:\Users\Public\Documents\ChemStation\1\Data\SUN\SUN 2023-03-18 13-24-33
                  \IC3-30-40.M (Sequence Method)
Last changed    : 19/3/2023 8:53:59 pm by SYSTEM
                  (modified after loading)
Additional Info : Peak(s) manually integrated
```

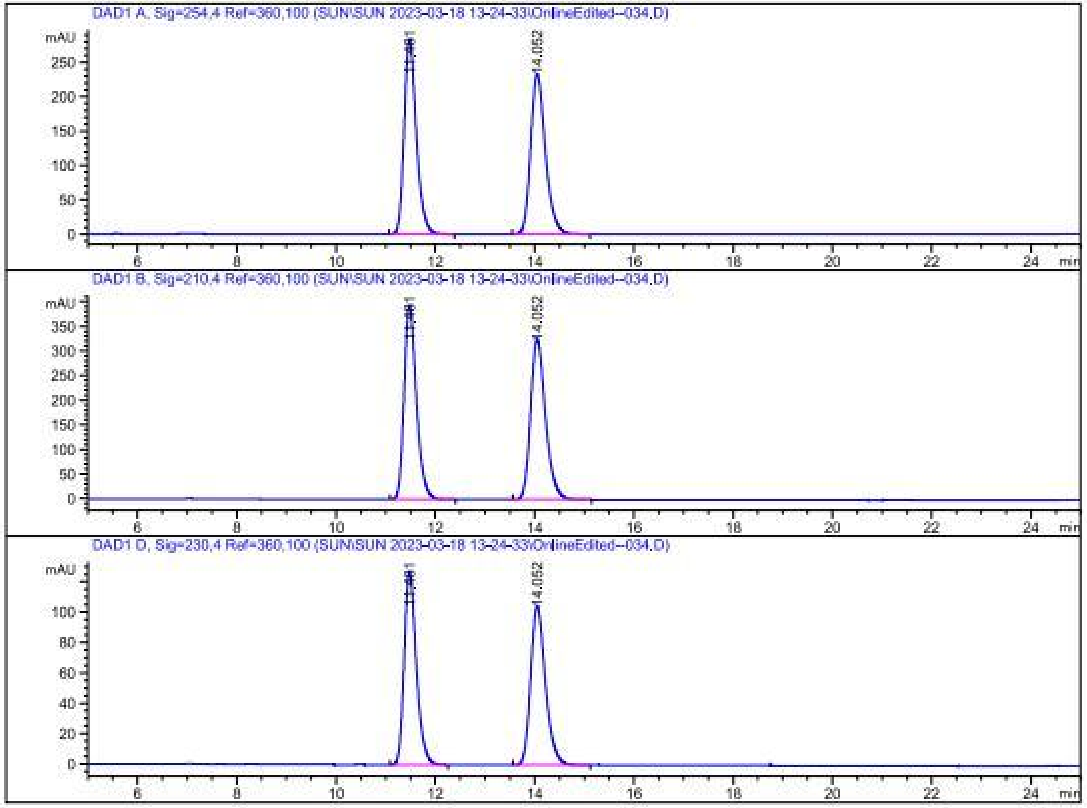

Data File C:\Users\P...\ChemStation\1\Data\SUN\SUN 2023-03-18 13-24-33\OnlineEdited--034.D
Sample Name: qcx-6-67a

```
=====================================================================
                         Area Percent Report
=====================================================================

Sorted By             :      Signal
Multiplier            :      1.0000
Dilution              :      1.0000
Use Multiplier & Dilution Factor with ISTDs
```

Signal 1: DAD1 A, Sig=254,4 Ref=360,100

Peak #	RetTime [min]	Type	Width [min]	Area [mAU*s]	Height [mAU]	Area %
1	11.481	BB	0.2595	4816.78027	283.87601	49.9871
2	14.052	BB	0.3140	4819.25879	233.32179	50.0129

Totals : 9636.03906 517.19780

Signal 2: DAD1 B, Sig=210,4 Ref=360,100

Peak #	RetTime [min]	Type	Width [min]	Area [mAU*s]	Height [mAU]	Area %
1	11.481	BB	0.2630	6774.79297	394.27856	49.8677
2	14.052	BB	0.3190	6810.73682	326.35312	50.1323

Totals : 1.35855e4 720.63168

Signal 3: DAD1 D, Sig=230,4 Ref=360,100

Peak #	RetTime [min]	Type	Width [min]	Area [mAU*s]	Height [mAU]	Area %
1	11.481	BB	0.2600	2170.30542	127.57659	49.9391
2	14.052	BB	0.3152	2175.59814	105.01630	50.0609

Totals : 4345.90356 232.59289

```
=====================================================================
                          *** End of Report ***
```

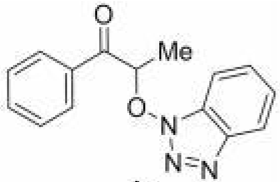

4a
racemic

```
=====================================================================
Acq. Operator   : SYSTEM                       Seq. Line :  21
Sample Operator : SYSTEM
Acq. Instrument : HPLC                          Location :   P2-A-06
Injection Date  : 17/3/2023 11:15:42 pm              Inj :   1
                                              Inj Volume : 2.000 µl
Different Inj Volume from Sample Entry!  Actual Inj Volume : 8.000 µl
Acq. Method     : C:\Users\Public\Documents\ChemStation\1\Data\SUN\SUN 2023-03-17 16-32-11
                  \IC3-30-40.M
Last changed    : 27/2/2023 10:13:57 pm by SYSTEM
Analysis Method : C:\Users\Public\Documents\ChemStation\1\Data\SUN\SUN 2023-03-17 16-32-11
                  \IC3-30-40.M (Sequence Method)
Last changed    : 19/3/2023 8:53:01 pm by SYSTEM
                  (modified after loading)
Additional Info : Peak(s) manually integrated
```

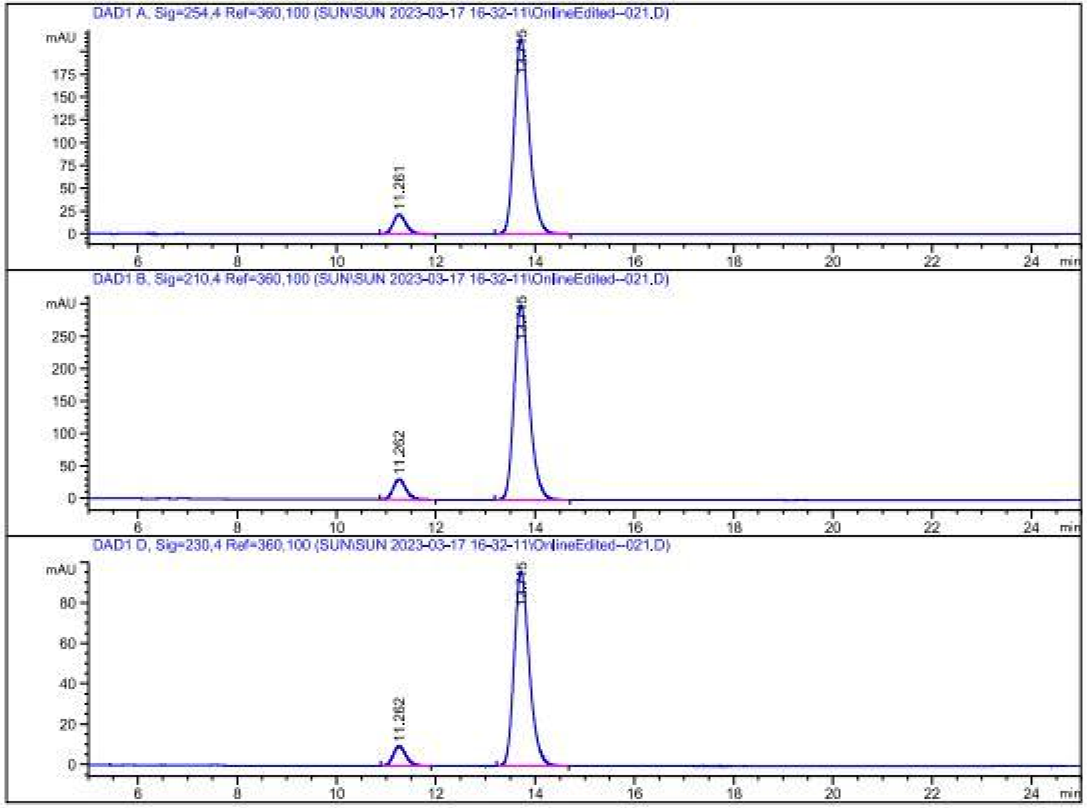

Data File C:\Users\P...\ChemStation\1\Data\SUN\SUN 2023-03-17 16-32-11\OnlineEdited--021.D
Sample Name: qcx-6-59b

Area Percent Report

```
Sorted By             :      Signal
Multiplier            :      1.0000
Dilution              :      1.0000
Use Multiplier & Dilution Factor with ISTDs
```

Signal 1: DAD1 A, Sig=254,4 Ref=360,100

Peak #	RetTime [min]	Type	Width [min]	Area [mAU*s]	Height [mAU]	Area %
1	11.261	BB	0.2771	388.37723	21.56706	7.9632
2	13.715	BB	0.3222	4488.76611	213.99385	92.0368
Totals :				4877.14334	235.56091	

Signal 2: DAD1 B, Sig=210,4 Ref=360,100

Peak #	RetTime [min]	Type	Width [min]	Area [mAU*s]	Height [mAU]	Area %
1	11.262	BB	0.2752	559.83032	31.07396	8.0887
2	13.715	BB	0.3255	6361.32373	300.48511	91.9113
Totals :				6921.15405	331.55906	

Signal 3: DAD1 D, Sig=230,4 Ref=360,100

Peak #	RetTime [min]	Type	Width [min]	Area [mAU*s]	Height [mAU]	Area %
1	11.262	BB	0.2601	175.54662	9.74773	7.9847
2	13.715	BB	0.3228	2022.97717	96.22204	92.0153
Totals :				2198.52379	105.96977	

*** End of Report ***

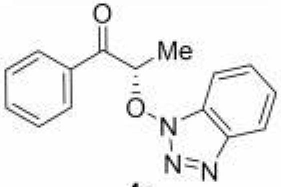

4a
enantioenriched

```
=====================================================================
Acq. Operator   : SYSTEM                        Seq. Line :  10
Sample Operator : SYSTEM
Acq. Instrument : HPLC                           Location :   P2-A-03
Injection Date  : 30/5/2023 7:33:34 pm                Inj :   1
                                               Inj Volume : 2.000 µl
Different Inj Volume from Sample Entry!  Actual Inj Volume : 8.000 µl
Acq. Method     : C:\Users\Public\Documents\ChemStation\1\Data\SUN\SUN 2023-05-30 17-01-04
                  \IC3-20-40.M
Last changed    : 4/1/2023 3:55:34 pm by SYSTEM
Analysis Method : C:\Users\Public\Documents\ChemStation\1\Data\SUN\SUN 2023-05-30 17-01-04
                  \IC3-20-40.M (Sequence Method)
Last changed    : 31/5/2023 5:22:58 pm by SYSTEM
                  (modified after loading)
Additional Info : Peak(s) manually integrated
```

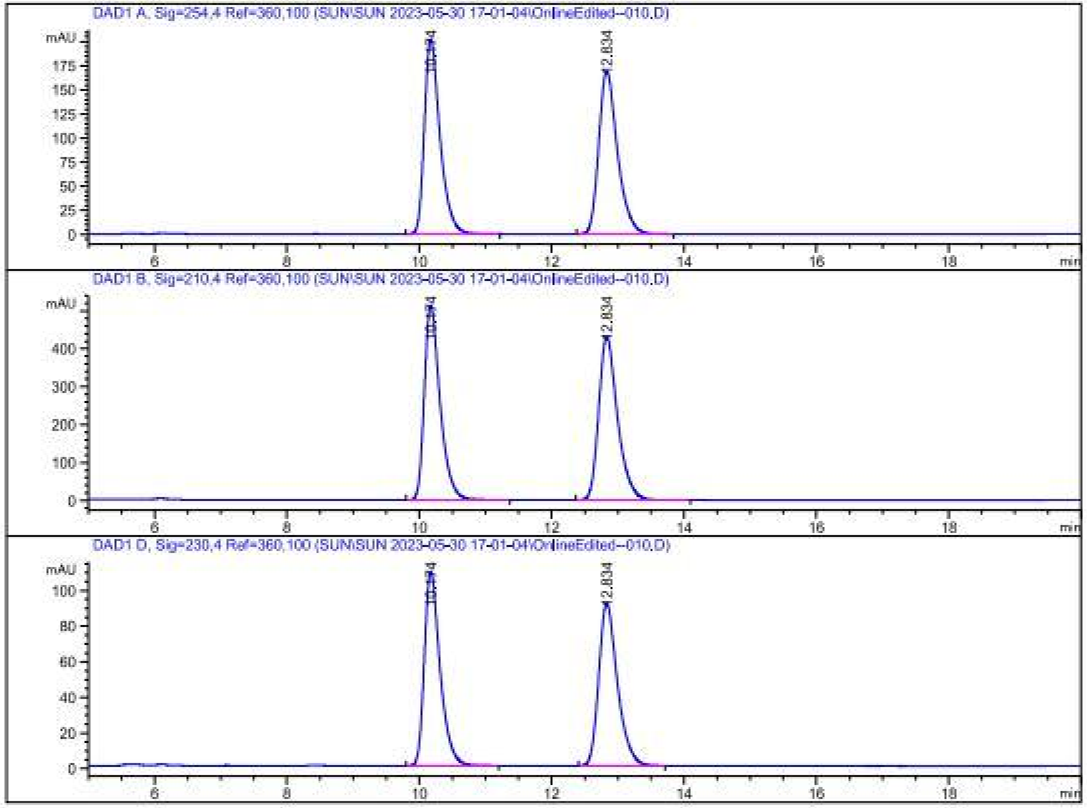

Data File C:\Users\P...\ChemStation\1\Data\SUN\SUN 2023-05-30 17-01-04\OnlineEdited--010.D
Sample Name: qcx-6-136c

```
=====================================================================
                         Area Percent Report
=====================================================================

Sorted By             :      Signal
Multiplier            :      1.0000
Dilution              :      1.0000
Use Multiplier & Dilution Factor with ISTDs
```

Signal 1: DAD1 A, Sig=254,4 Ref=360,100

Peak #	RetTime [min]	Type	Width [min]	Area [mAU*s]	Height [mAU]	Area %
1	10.174	BB	0.2468	3317.80420	202.34300	49.9836
2	12.834	BB	0.2975	3319.98364	169.13940	50.0164
Totals :				6637.78784	371.48241	

Signal 2: DAD1 B, Sig=210,4 Ref=360,100

Peak #	RetTime [min]	Type	Width [min]	Area [mAU*s]	Height [mAU]	Area %
1	10.174	BB	0.2480	8470.92871	513.29932	49.9343
2	12.834	BB	0.2979	8493.23340	430.11377	50.0657
Totals :				1.69642e4	943.41309	

Signal 3: DAD1 D, Sig=230,4 Ref=360,100

Peak #	RetTime [min]	Type	Width [min]	Area [mAU*s]	Height [mAU]	Area %
1	10.174	BB	0.2492	1799.18677	109.19703	50.1051
2	12.834	BB	0.2983	1791.63538	91.14478	49.8949
Totals :				3590.82214	200.34181	

```
=====================================================================
                          *** End of Report ***
```

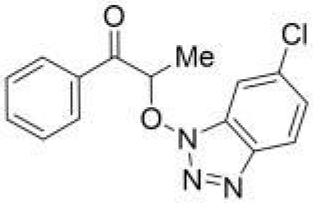

4b
racemic

===

Acq. Operator : SYSTEM Seq. Line : 51
Sample Operator : SYSTEM
Acq. Instrument : HPLC Location : P2-A-05
Injection Date : 31/5/2023 2:01:26 pm Inj : 1
Inj Volume : 2.000 µl
Different Inj Volume from Sample Entry! Actual Inj Volume : 1.000 µl
Acq. Method : C:\Users\Public\Documents\ChemStation\1\Data\SUN\SUN 2023-05-30 17-01-04 \IC3-20-20.M
Last changed : 16/5/2023 11:01:35 am by SYSTEM
Analysis Method : C:\Users\Public\Documents\ChemStation\1\Data\SUN\SUN 2023-05-30 17-01-04 \IC3-20-20.M (Sequence Method)
Last changed : 31/5/2023 5:24:26 pm by SYSTEM
(modified after loading)
Additional Info : Peak(s) manually integrated

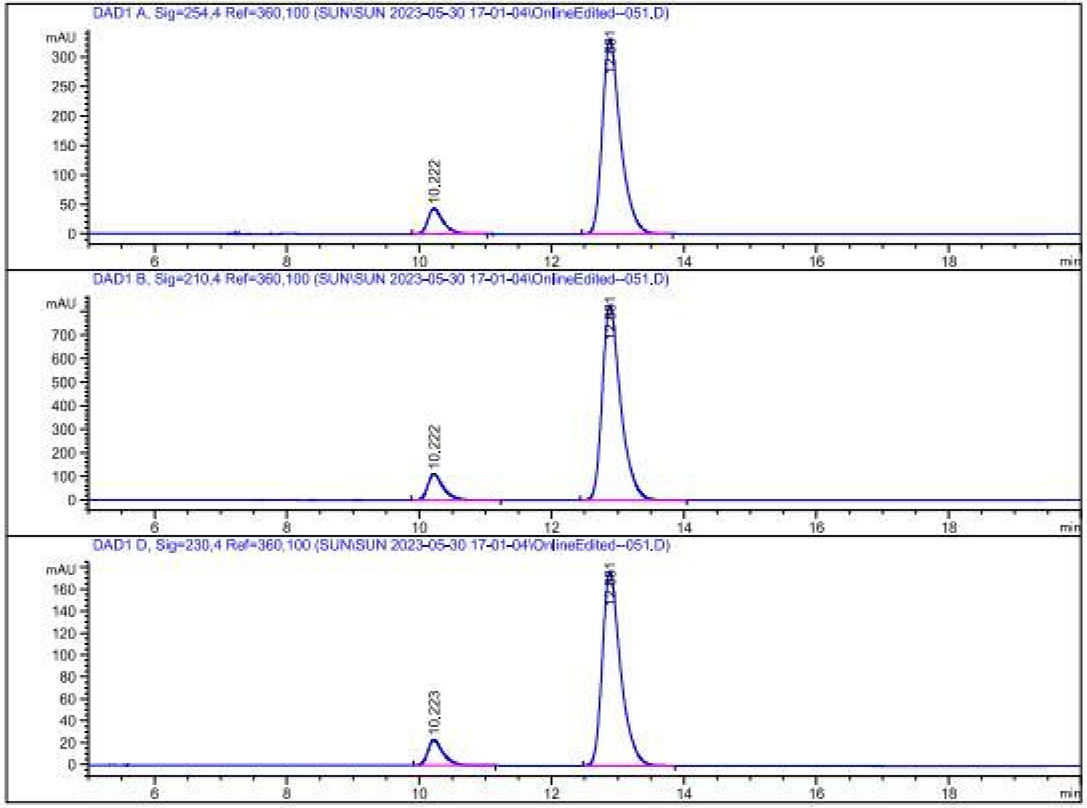

Data File C:\Users\P...\ChemStation\1\Data\SUN\SUN 2023-05-30 17-01-04\OnlineEdited--051.D
Sample Name: qcx-6-136a

===

Area Percent Report

===

Sorted By : Signal
Multiplier : 1.0000
Dilution : 1.0000
Use Multiplier & Dilution Factor with ISTDs

Signal 1: DAD1 A, Sig=254,4 Ref=360,100

Peak #	RetTime [min]	Type	Width [min]	Area [mAU*s]	Height [mAU]	Area %
1	10.222	BB	0.2383	692.12158	43.23661	9.9125
2	12.881	BB	0.2851	6290.21387	330.37418	90.0875

Totals : 6982.33545 373.61079

Signal 2: DAD1 B, Sig=210,4 Ref=360,100

Peak #	RetTime [min]	Type	Width [min]	Area [mAU*s]	Height [mAU]	Area %
1	10.222	BB	0.2381	1792.09070	111.15955	10.1027
2	12.881	BB	0.2900	1.59467e4	828.88251	89.8973

Totals : 1.77388e4 940.04205

Signal 3: DAD1 D, Sig=230,4 Ref=360,100

Peak #	RetTime [min]	Type	Width [min]	Area [mAU*s]	Height [mAU]	Area %
1	10.223	BB	0.2329	378.20682	23.48167	10.0289
2	12.881	BB	0.2872	3392.95630	177.32022	89.9711

Totals : 3771.16312 200.80189

===

*** End of Report ***

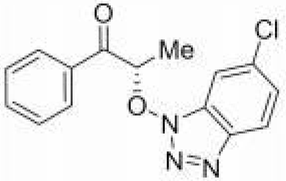

4b
enantioenriched

```
=====================================================================
Acq. Operator   : SYSTEM                    Seq. Line :    5
Acq. Instrument : LC1260                     Location :   P1-A-03
Injection Date  : 7/24/2025 11:15:27 PM           Inj :    1
                                           Inj Volume : 5.000 µl
Different Inj Volume from Sample Entry!  Actual Inj Volume : 30.000 µl
Acq. Method     : C:\Users\Public\Documents\ChemStation\1\Data\SUN\SUN 2025-07-24 22-08-59
                  \IG3-10-30.M
Last changed    : 11/28/2024 8:36:39 PM by SYSTEM
Analysis Method : C:\Users\Public\Documents\ChemStation\1\Data\SUN\SUN 2025-07-24 22-08-59
                  \IG3-10-30.M (Sequence Method)
Last changed    : 7/25/2025 1:29:30 PM by SYSTEM
                  (modified after loading)
Additional Info : Peak(s) manually integrated
```

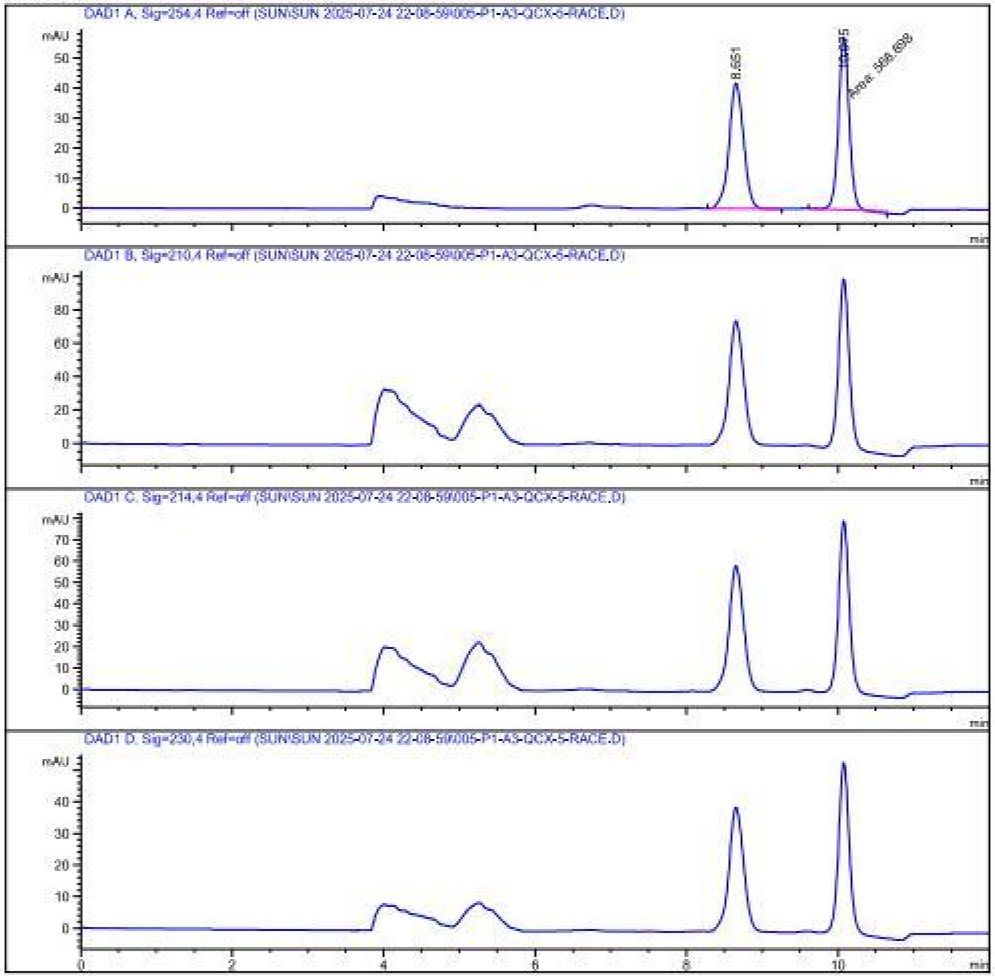

Data File C:\Users\P...emStation\1\Data\SUN\SUN 2025-07-24 22-08-59\005-P1-A3-QCX-5-RACE.D
Sample Name: QCX-5-RACE

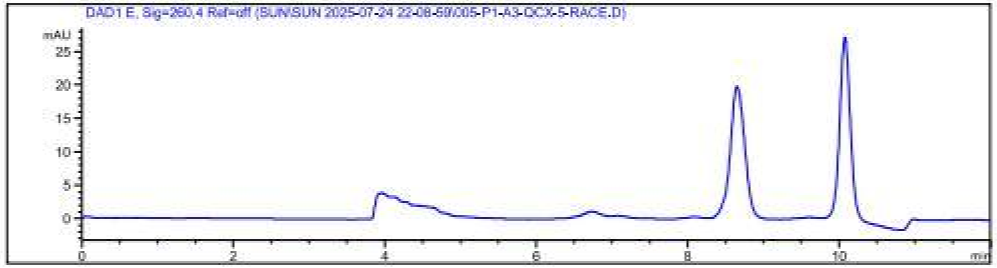


```
=====================================================================
                         Area Percent Report
=====================================================================

Sorted By             :      Signal
Multiplier            :      1.0000
Dilution              :      1.0000
Use Multiplier & Dilution Factor with ISTDs


Signal 1: DAD1 A, Sig=254,4 Ref=off

Peak RetTime Type  Width     Area      Height     Area
  #   [min]        [min]   [mAU*s]     [mAU]        %
----|-------|----|-------|----------|----------|--------|
   1   8.651 BB    0.2091  562.36920   41.77102  49.7114
   2  10.075 MM    0.1646  568.89813   57.60205  50.2886

Totals :                   1131.26733   99.37308


Signal 2: DAD1 B, Sig=210,4 Ref=off

Signal 3: DAD1 C, Sig=214,4 Ref=off

Signal 4: DAD1 D, Sig=230,4 Ref=off

Signal 5: DAD1 E, Sig=260,4 Ref=off

=====================================================================
                         *** End of Report ***
```

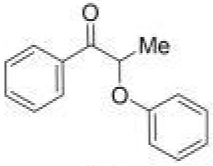

4c
racemic

===

Acq. Operator : SYSTEM Seq. Line : 6
Acq. Instrument : LC1260 Location : P1-A-04
Injection Date : 7/24/2025 11:46:37 PM Inj : 1
Inj Volume : 5.000 µl
Different Inj Volume from Sample Entry! Actual Inj Volume : 30.000 µl
Acq. Method : C:\Users\Public\Documents\ChemStation\1\Data\SUN\SUN 2025-07-24 22-08-59\IG3-10-30.M
Last changed : 11/28/2024 8:36:39 PM by SYSTEM
Analysis Method : C:\Users\Public\Documents\ChemStation\1\Data\SUN\SUN 2025-07-24 22-08-59\IG3-10-30.M (Sequence Method)
Last changed : 7/25/2025 1:36:22 PM by SYSTEM
(modified after loading)
Additional Info : Peak(s) manually integrated

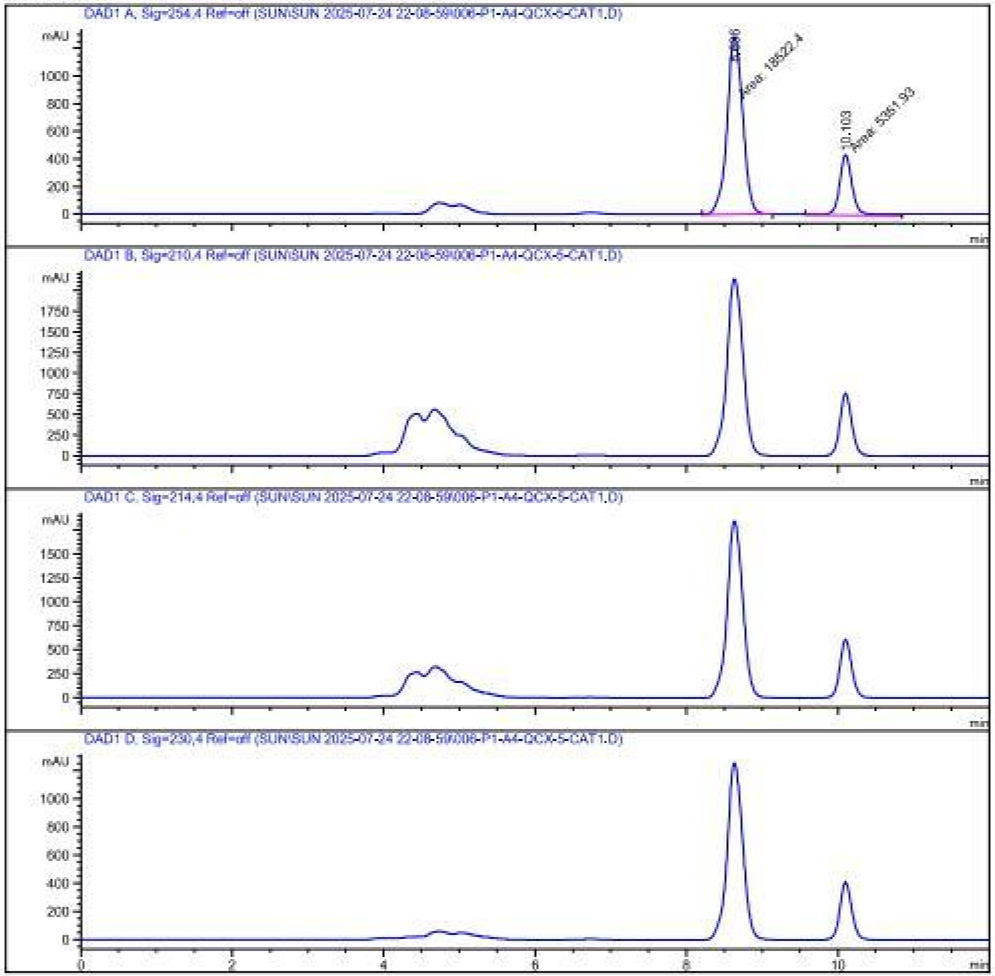

Data File C:\Users\P...emStation\1\Data\SUN\SUN 2025-07-24 22-08-59\006-P1-A4-QCX-5-CAT1.D
Sample Name: QCX-5-CAT1

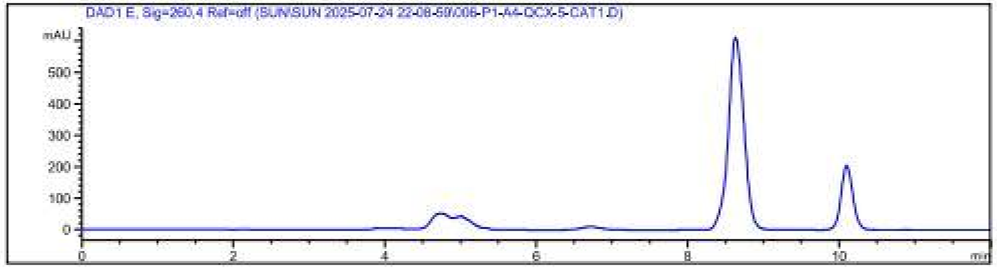

===
Area Percent Report
===

Sorted By : Signal
Multiplier : 1.0000
Dilution : 1.0000
Use Multiplier & Dilution Factor with ISTDs

Signal 1: DAD1 A, Sig=254,4 Ref=off

Peak #	RetTime [min]	Type	Width [min]	Area [mAU*s]	Height [mAU]	Area %
1	8.636	MM	0.2409	1.85224e4	1281.70337	77.5829
2	10.103	MM	0.2039	5351.93359	437.41751	22.4171

Totals : 2.38743e4 1719.12088

Signal 2: DAD1 B, Sig=210,4 Ref=off

Signal 3: DAD1 C, Sig=214,4 Ref=off

Signal 4: DAD1 D, Sig=230,4 Ref=off

Signal 5: DAD1 E, Sig=260,4 Ref=off

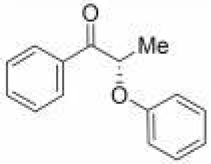

4c
enantioenriched

===
*** End of Report ***

===

Acq. Operator : SYSTEM Seq. Line : 17
Sample Operator : SYSTEM
Acq. Instrument : HPLC Location : P2-A-05
Injection Date : 17/3/2023 9:40:35 pm Inj : 1
Inj Volume : 2.000 µl
Different Inj Volume from Sample Entry! Actual Inj Volume : 8.000 µl
Acq. Method : C:\Users\Public\Documents\ChemStation\1\Data\SUN\SUN 2023-03-17 16-32-11\IC3-20-40.M
Last changed : 4/1/2023 3:55:34 pm by SYSTEM
Analysis Method : C:\Users\Public\Documents\ChemStation\1\Data\SUN\SUN 2023-03-17 16-32-11\IC3-20-40.M (Sequence Method)
Last changed : 24/3/2023 7:56:20 pm by SYSTEM
(modified after loading)
Additional Info : Peak(s) manually integrated

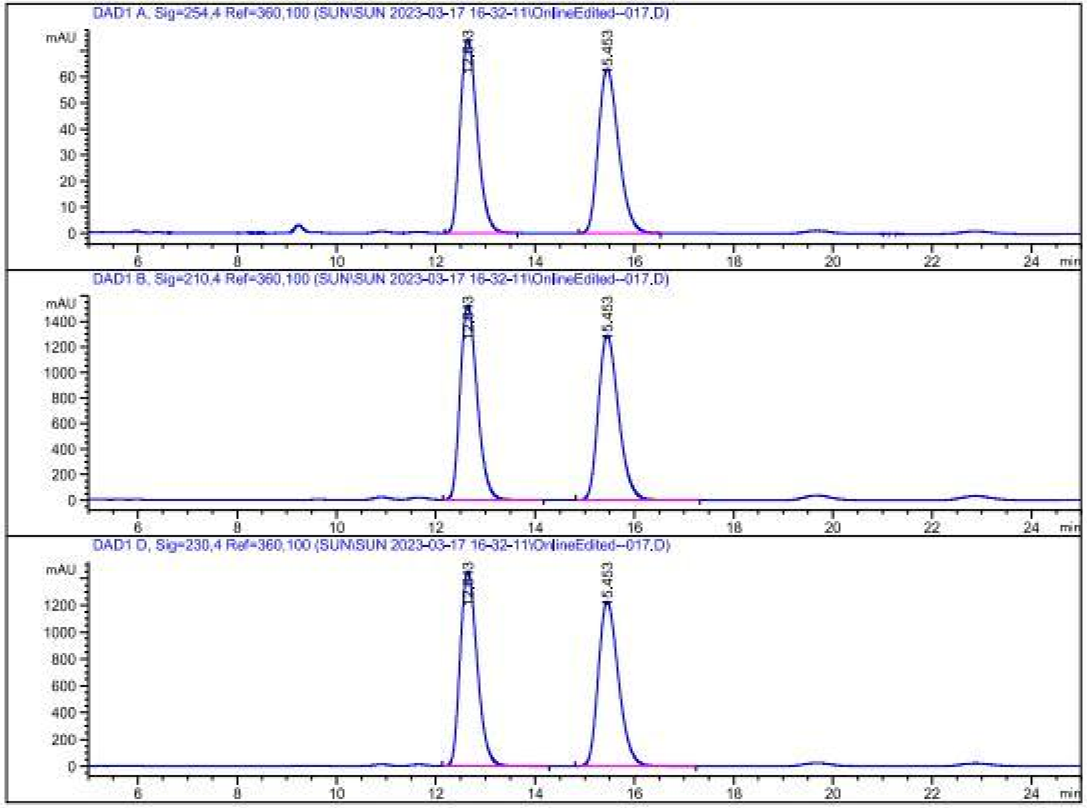

Data File C:\Users\P...\ChemStation\1\Data\SUN\SUN 2023-03-17 16-32-11\OnlineEdited--017.D
Sample Name: qcx-6-62b

===

Area Percent Report

===

Sorted By : Signal
Multiplier : 1.0000
Dilution : 1.0000
Use Multiplier & Dilution Factor with ISTDs

Signal 1: DAD1 A, Sig=254,4 Ref=360,100

Peak #	RetTime [min]	Type	Width [min]	Area [mAU*s]	Height [mAU]	Area %
1	12.643	BB	0.3715	1773.22229	74.41598	49.8173
2	15.453	BB	0.4360	1786.23181	63.04772	50.1827

Totals : 3559.45410 137.46370

Signal 2: DAD1 B, Sig=210,4 Ref=360,100

Peak #	RetTime [min]	Type	Width [min]	Area [mAU*s]	Height [mAU]	Area %
1	12.643	BB	0.3730	3.61593e4	1522.71240	49.9146
2	15.453	BB	0.4356	3.62831e4	1288.20996	50.0854

Totals : 7.24424e4 2810.92236

Signal 3: DAD1 D, Sig=230,4 Ref=360,100

Peak #	RetTime [min]	Type	Width [min]	Area [mAU*s]	Height [mAU]	Area %
1	12.643	BB	0.3730	3.44355e4	1452.74988	49.9297
2	15.453	BB	0.4375	3.45325e4	1228.25696	50.0703

Totals : 6.89681e4 2681.00684

===

*** End of Report ***

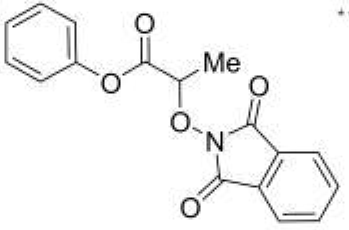

5
racemic

Acq. Operator : SYSTEM Seq. Line : 7
Sample Operator : SYSTEM
Acq. Instrument : HPLC Location : P1-E-03
Injection Date : 24/3/2023 6:45:34 pm Inj : 1
Inj Volume : 2.000 µl
Different Inj Volume from Sample Entry! Actual Inj Volume : 4.000 µl
Acq. Method : C:\Users\Public\Documents\ChemStation\1\Data\SUN\SUN 2023-03-24 15-54-54 \IC3-20-40.M
Last changed : 4/1/2023 3:55:34 pm by SYSTEM
Analysis Method : C:\Users\Public\Documents\ChemStation\1\Data\SUN\SUN 2023-03-24 15-54-54 \IC3-20-40.M (Sequence Method)
Last changed : 24/3/2023 7:54:53 pm by SYSTEM
(modified after loading)
Additional Info : Peak(s) manually integrated

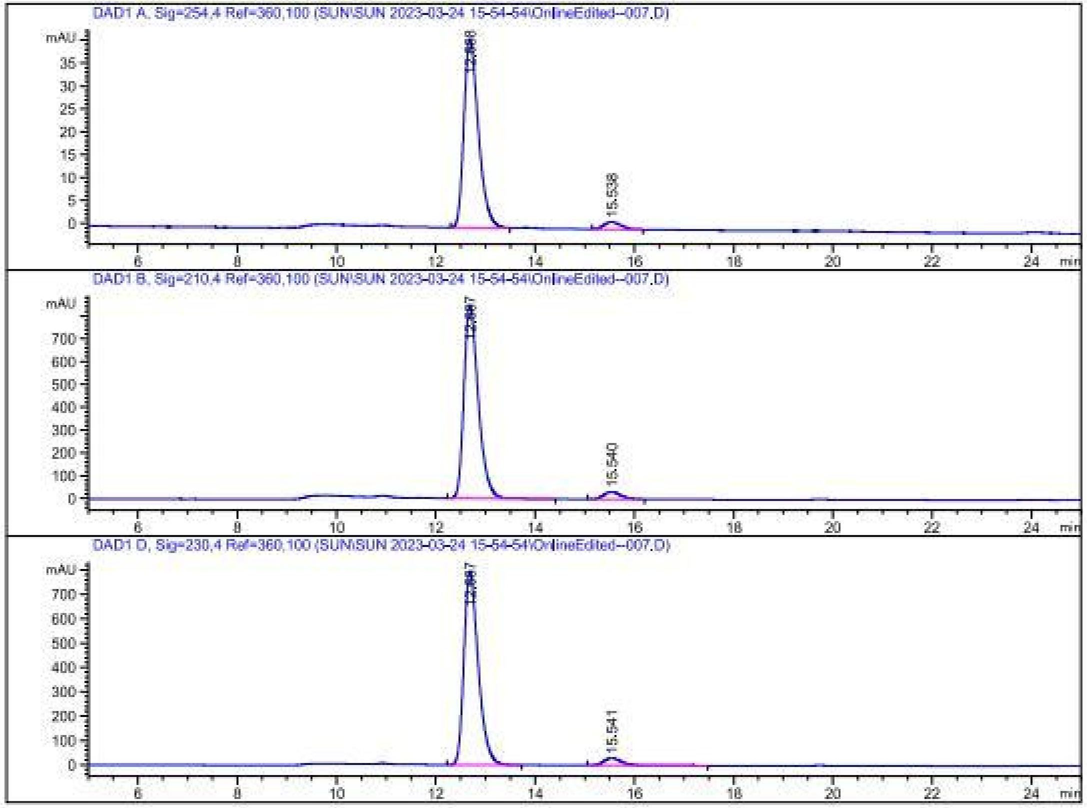

Data File C:\Users\P...\ChemStation\1\Data\SUN\SUN 2023-03-24 15-54-54\OnlineEdited--007.D
Sample Name: qcx-6-76b-new-2

Area Percent Report

Sorted By : Signal
Multiplier : 1.0000
Dilution : 1.0000
Use Multiplier & Dilution Factor with ISTDs

Signal 1: DAD1 A, Sig=254,4 Ref=360,100

Peak #	RetTime [min]	Type	Width [min]	Area [mAU*s]	Height [mAU]	Area %
1	12.688	BB	0.3061	831.28479	41.16321	95.5495
2	15.538	BB	0.2838	38.71957	1.62033	4.4505

Totals : 870.00436 42.78354

Signal 2: DAD1 B, Sig=210,4 Ref=360,100

Peak #	RetTime [min]	Type	Width [min]	Area [mAU*s]	Height [mAU]	Area %
1	12.687	BB	0.3039	1.70049e4	846.41376	95.6670
2	15.540	BB	0.3529	770.18738	32.05084	4.3330

Totals : 1.77751e4 878.46460

Signal 3: DAD1 D, Sig=230,4 Ref=360,100

Peak #	RetTime [min]	Type	Width [min]	Area [mAU*s]	Height [mAU]	Area %
1	12.687	BB	0.3023	1.59205e4	796.19250	95.4113
2	15.541	BB	0.3766	765.68701	30.42228	4.5887

Totals : 1.66862e4 826.61478

*** End of Report ***

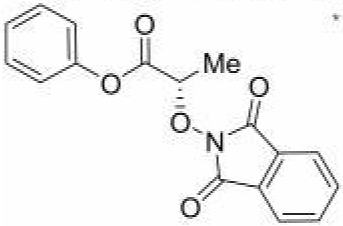

5
enantioenriched

Area Percent Report

sample OH insertion

Data file: C:\Users\Public\Documents\ChemStation\1\Data\2025-07-24\czq 2025-07-24 11-34-43\020-P2-A1-qcx-9-207c.D

Acquisition Data:

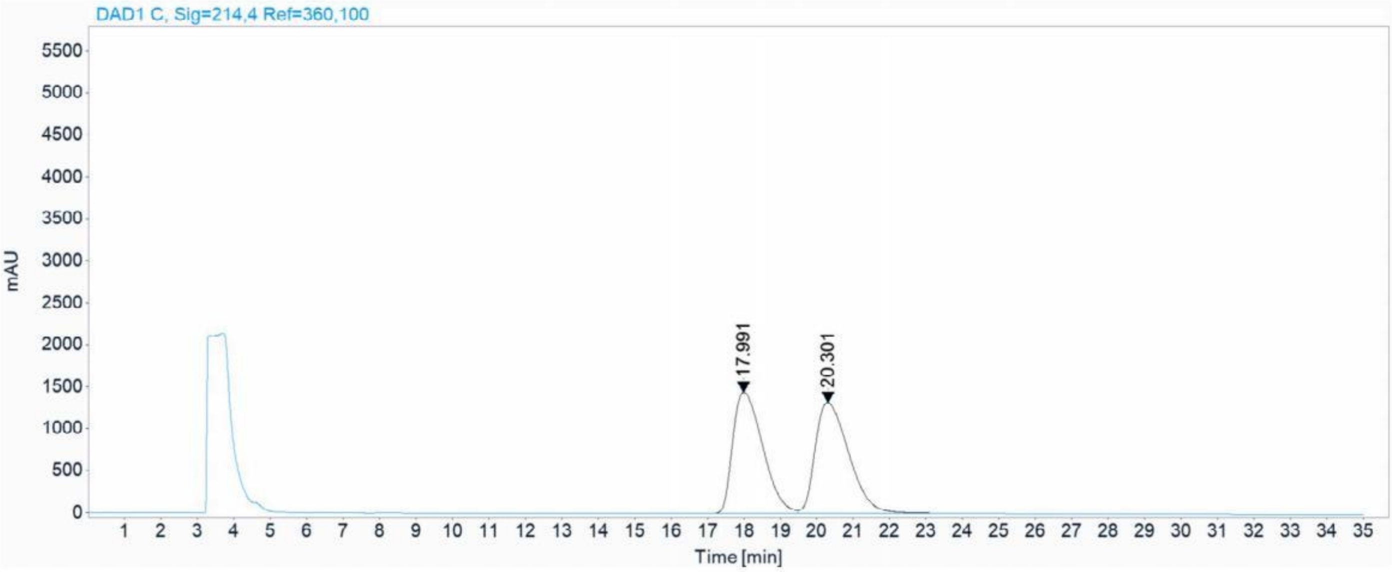

Signal: DAD1 C, Sig=214,4 Ref=360,100

RT [min]	Width [min]	Height	Area	Area%
17.991	0.7764	1444.5223	82512.0469	49.3070
20.301	0.9091	1321.1910	84831.3203	50.6930
		Sum	167343.3672	100.0000

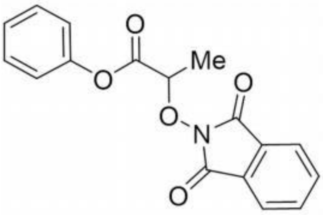

5
racemic
from ylide

Area Percent Report

sample OH insertion

Data file: C:\Users\Public\Documents\ChemStation\1\Data\2025-07-24\czq 2025-07-24 11-34-43\022-P2-A3-qcx-9-207b.D

Acquisition Data:

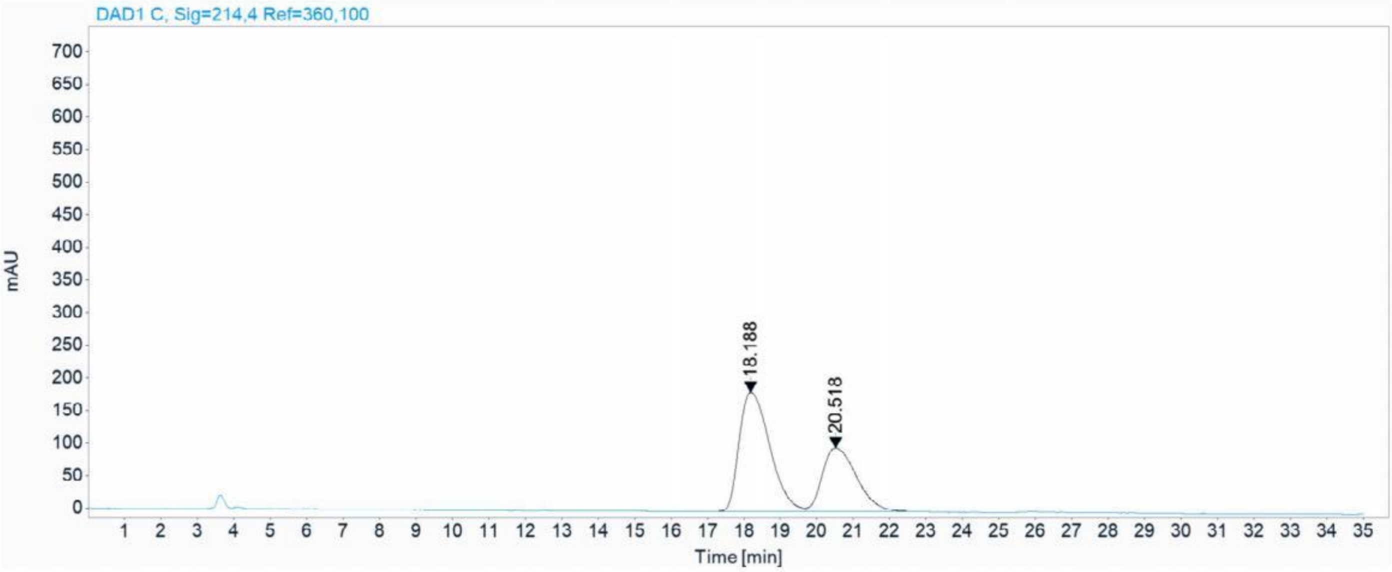

Signal: DAD1 C, Sig=214,4 Ref=360,100

RT [min]	Width [min]	Height	Area	Area%
18.188	0.9158	182.3887	10627.2168	62.4506
20.518	0.8944	97.5285	6389.7720	37.5494
		Sum	17016.9888	100.0000

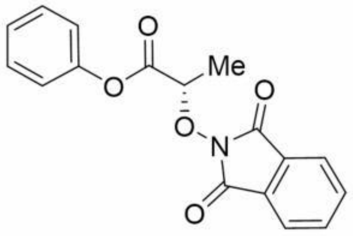

5
enantioenriched
from ylide

Acq. Operator : SYSTEM Seq. Line : 2
Sample Operator : SYSTEM
Acq. Instrument : HPLC Location : P1-F-02
Injection Date : 24/3/2023 11:36:48 pm Inj : 1
Inj Volume : 2.000 µl
Different Inj Volume from Sample Entry! Actual Inj Volume : 8.000 µl
Acq. Method : C:\Users\Public\Documents\ChemStation\1\Data\SUN\SUN 2023-03-24 23-24-36\OD3-10-20.M
Last changed : 15/8/2022 10:27:52 pm by SYSTEM
Analysis Method : C:\Users\Public\Documents\ChemStation\1\Data\SUN\SUN 2023-03-24 23-24-36\OD3-10-20.M (Sequence Method)
Last changed : 27/3/2023 10:08:48 pm by SYSTEM
(modified after loading)
Additional Info : Peak(s) manually integrated

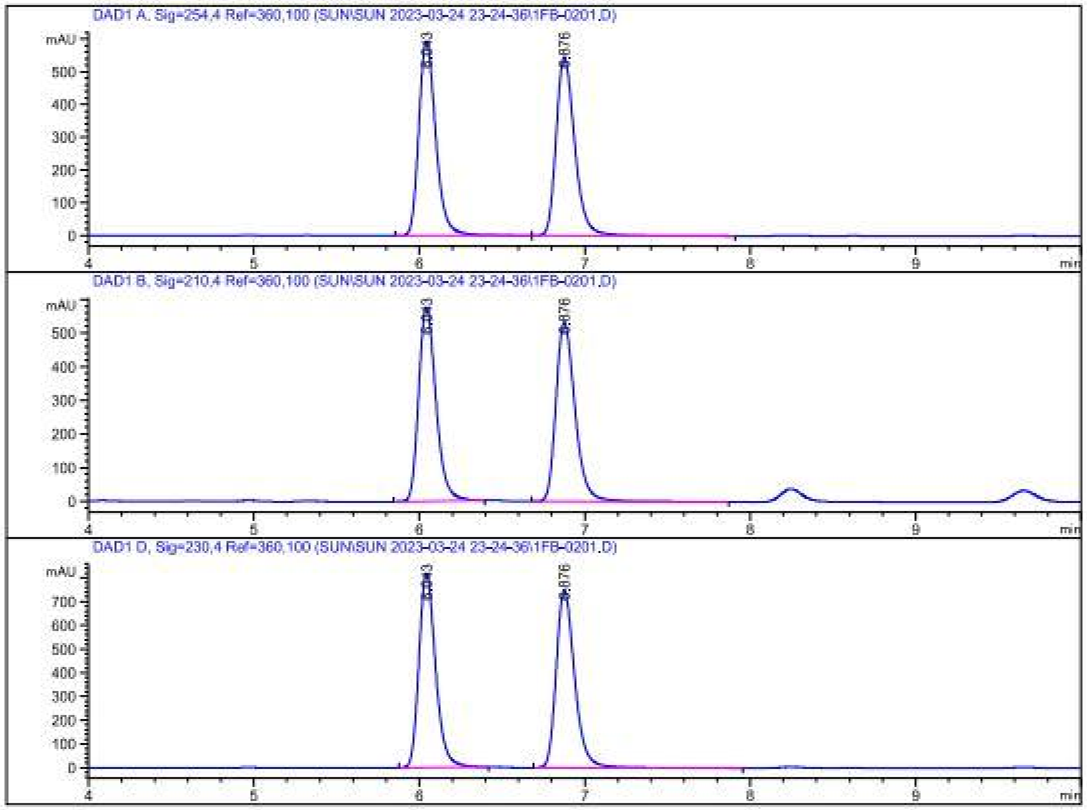

Data File C:\Users\P...Documents\ChemStation\1\Data\SUN\SUN 2023-03-24 23-24-36\1FB-0201.D
Sample Name: qcx-6-76a

Area Percent Report

Sorted By : Signal
Multiplier : 1.0000
Dilution : 1.0000
Use Multiplier & Dilution Factor with ISTDs

Signal 1: DAD1 A, Sig=254,4 Ref=360,100

Peak #	RetTime [min]	Type	Width [min]	Area [mAU*s]	Height [mAU]	Area %
1	6.043	BB	0.1111	4259.46338	594.22845	49.8987
2	6.876	BB	0.1210	4276.76074	545.34674	50.1013

Totals : 8536.22412 1139.57520

Signal 2: DAD1 B, Sig=210,4 Ref=360,100

Peak #	RetTime [min]	Type	Width [min]	Area [mAU*s]	Height [mAU]	Area %
1	6.043	BB	0.1131	4181.06445	576.43054	49.4040
2	6.876	BB	0.1252	4281.93750	532.87781	50.5960

Totals : 8463.00195 1109.30835

Signal 3: DAD1 D, Sig=230,4 Ref=360,100

Peak #	RetTime [min]	Type	Width [min]	Area [mAU*s]	Height [mAU]	Area %
1	6.043	BB	0.1082	5740.65625	819.86353	49.6053
2	6.876	BB	0.1191	5832.00049	751.06561	50.3947

Totals : 1.15727e4 1570.92914

*** End of Report ***

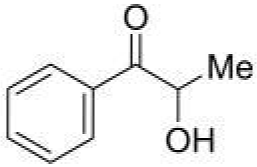

6
racemic

Acq. Operator : SYSTEM Seq. Line : 20
Sample Operator : SYSTEM
Acq. Instrument : HPLC Location : P2-B-03
Injection Date : 27/3/2023 11:47:07 pm Inj : 1
Inj Volume : 2.000 µl
Different Inj Volume from Sample Entry! Actual Inj Volume : 10.000 µl
Acq. Method : C:\Users\Public\Documents\ChemStation\1\Data\SUN\SUN 2023-03-27 13-30-18\OD3-10-20.M
Last changed : 15/8/2022 10:27:52 pm by SYSTEM
Analysis Method : C:\Users\Public\Documents\ChemStation\1\Data\SUN\SUN 2023-03-27 13-30-18\OD3-10-20.M (Sequence Method)
Last changed : 28/3/2023 1:30:04 pm by SYSTEM
(modified after loading)
Additional Info : Peak(s) manually integrated

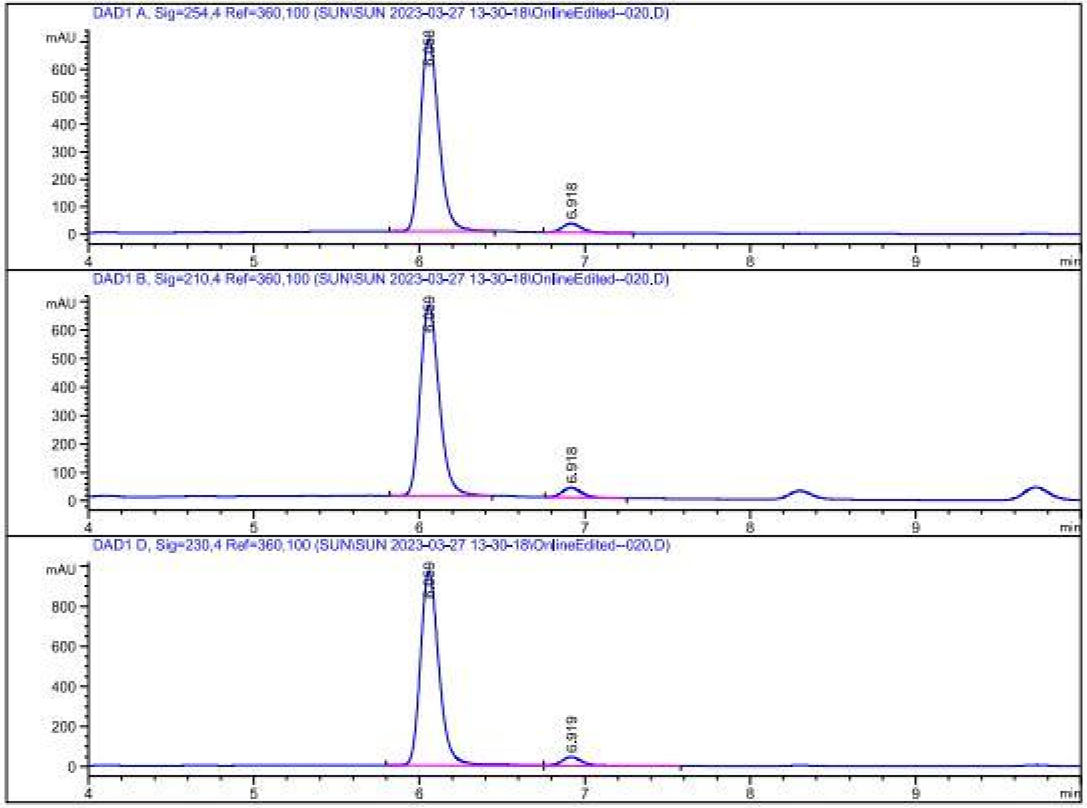

Data File C:\Users\P...\ChemStation\1\Data\SUN\SUN 2023-03-27 13-30-18\OnlineEdited--020.D
Sample Name: qcx-6-80a

Area Percent Report

Sorted By : Signal
Multiplier : 1.0000
Dilution : 1.0000
Use Multiplier & Dilution Factor with ISTDs

Signal 1: DAD1 A, Sig=254,4 Ref=360,100

Peak #	RetTime [min]	Type	Width [min]	Area [mAU*s]	Height [mAU]	Area %
1	6.058	BB	0.1203	5421.05957	700.22693	95.3694
2	6.918	BB	0.1237	263.21899	32.77559	4.6306

Totals : 5684.27856 733.00251

Signal 2: DAD1 B, Sig=210,4 Ref=360,100

Peak #	RetTime [min]	Type	Width [min]	Area [mAU*s]	Height [mAU]	Area %
1	6.059	BB	0.1243	5322.44482	669.44495	95.0842
2	6.918	BB	0.1249	275.16638	34.17027	4.9158

Totals : 5597.61121 703.61522

Signal 3: DAD1 D, Sig=230,4 Ref=360,100

Peak #	RetTime [min]	Type	Width [min]	Area [mAU*s]	Height [mAU]	Area %
1	6.059	BV R	0.1192	7529.49512	969.68341	95.2523
2	6.919	VB	0.1292	375.29553	44.34919	4.7477

Totals : 7904.79065 1014.03260

*** End of Report ***

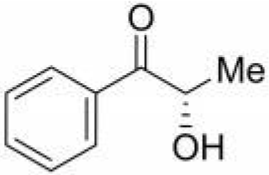

6
enantioenriched

Acq. Operator : SYSTEM Seq. Line : 2
Sample Operator : SYSTEM
Acq. Instrument : HPLC Location : P2-F-02
Injection Date : 12/4/2023 3:24:06 pm Inj : 1
Inj Volume : 2.000 µl
Different Inj Volume from Sample Entry! Actual Inj Volume : 10.000 µl
Acq. Method : C:\Users\Public\Documents\ChemStation\1\Data\SUN\SUN 2023-04-12 14-32-01 \OD3-3-30.M
Last changed : 12/4/2023 3:03:56 pm by SYSTEM
Analysis Method : C:\Users\Public\Documents\ChemStation\1\Data\SUN\SUN 2023-04-12 14-32-01 \OD3-3-30.M (Sequence Method)
Last changed : 14/4/2023 6:58:22 pm by SYSTEM
(modified after loading)
Additional Info : Peak(s) manually integrated

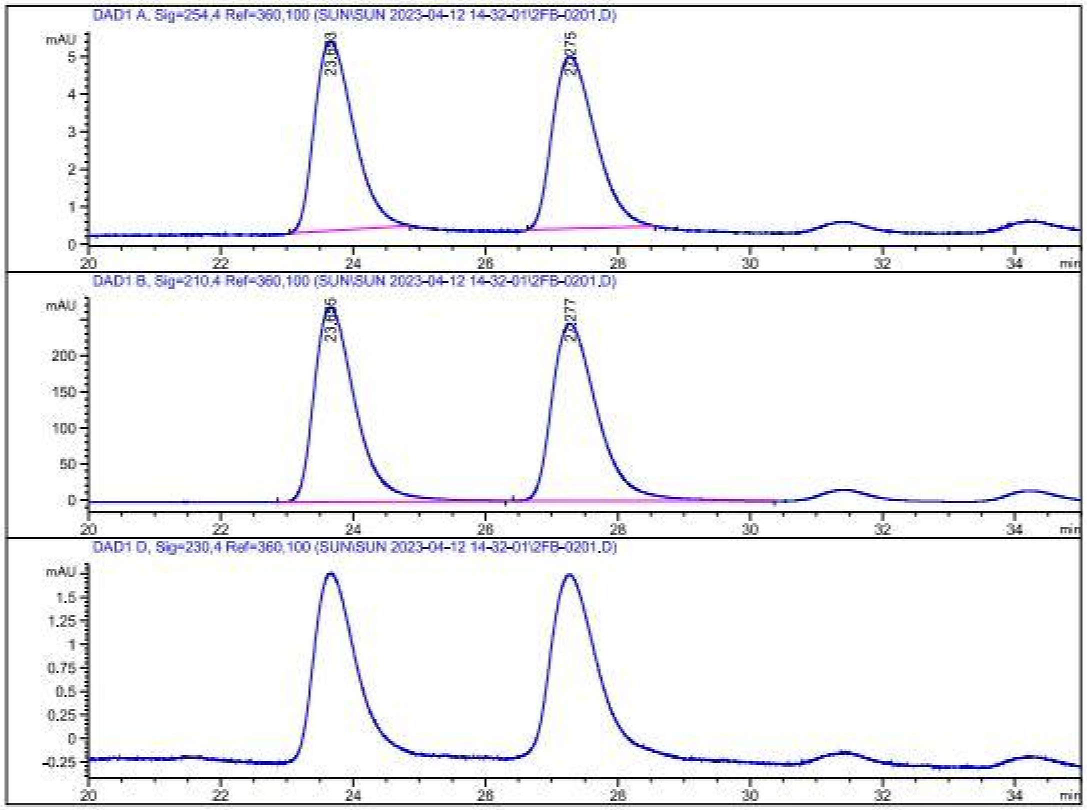

Data File C:\Users\P...Documents\ChemStation\1\Data\SUN\SUN 2023-04-12 14-32-01\2FB-0201.D
Sample Name: qcx-6-92b-3

Area Percent Report

Sorted By : Signal
Multiplier : 1.0000
Dilution : 1.0000
Use Multiplier & Dilution Factor with ISTDs

Signal 1: DAD1 A, Sig=254,4 Ref=360,100

Peak #	RetTime [min]	Type	Width [min]	Area [mAU*s]	Height [mAU]	Area %
1	23.653	BB	0.4837	208.44455	5.05901	50.1730
2	27.275	BB	0.5287	207.00706	4.58951	49.8270

Totals : 415.45161 9.64852

Signal 2: DAD1 B, Sig=210,4 Ref=360,100

Peak #	RetTime [min]	Type	Width [min]	Area [mAU*s]	Height [mAU]	Area %
1	23.655	BB	0.6500	1.16279e4	269.41333	49.8934
2	27.277	BB	0.7085	1.16776e4	245.48015	50.1066

Totals : 2.33054e4 514.89348

Signal 3: DAD1 D, Sig=230,4 Ref=360,100

*** End of Report ***

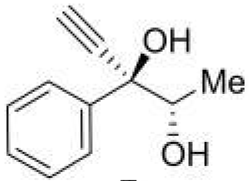

7
racemic

```
=====================================================================
Acq. Operator   : SYSTEM                        Seq. Line :   1
Sample Operator : SYSTEM
Acq. Instrument : HPLC                           Location :   P2-F-01
Injection Date  : 12/4/2023 2:33:04 pm                Inj :   1
                                               Inj Volume : 2.000 µl
Different Inj Volume from Sample Entry!  Actual Inj Volume : 10.000 µl
Acq. Method     : C:\Users\Public\Documents\ChemStation\1\Data\SUN\SUN 2023-04-12 14-32-01
                  \OD3-3-30.M
Last changed    : 12/4/2023 3:03:56 pm by SYSTEM
                  (modified after loading)
Analysis Method : C:\Users\Public\Documents\ChemStation\1\Data\SUN\SUN 2023-04-12 14-32-01
                  \OD3-3-30.M (Sequence Method)
Last changed    : 14/4/2023 6:58:22 pm by SYSTEM
                  (modified after loading)
Additional Info : Peak(s) manually integrated
```

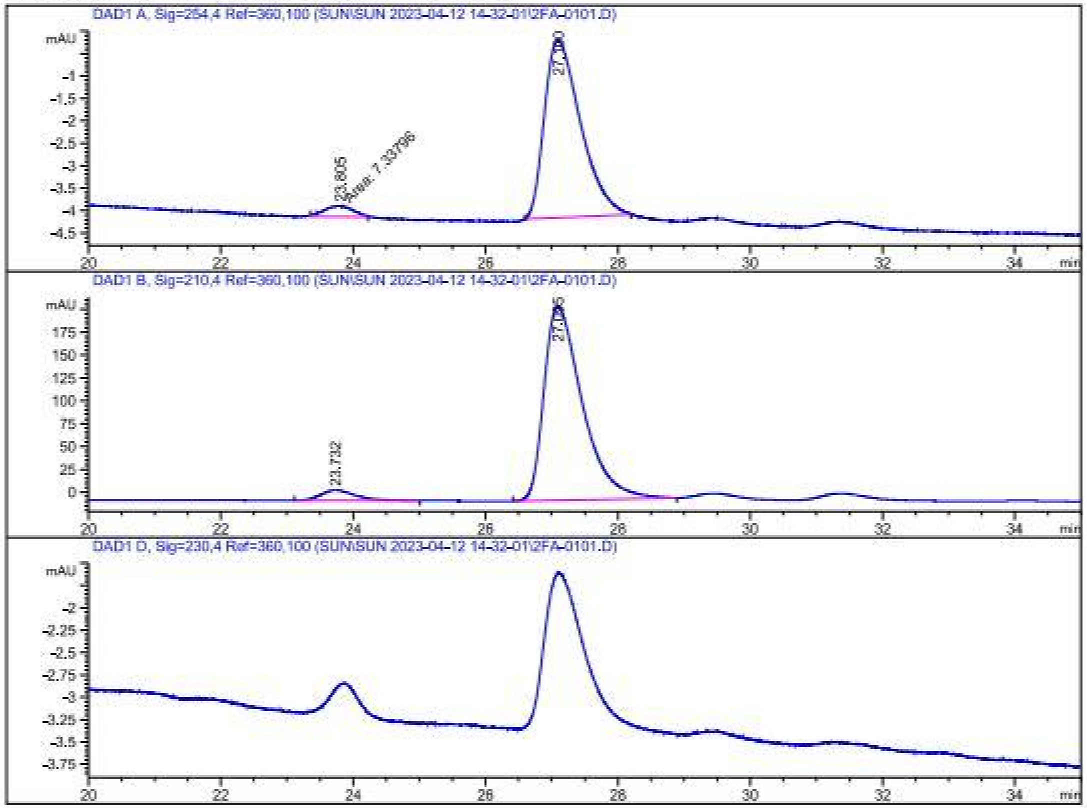

Data File C:\Users\P...Documents\ChemStation\1\Data\SUN\SUN 2023-04-12 14-32-01\2FA-0101.D
Sample Name: qcx-6-97

```
=====================================================================
                         Area Percent Report
=====================================================================

Sorted By             :      Signal
Multiplier            :      1.0000
Dilution              :      1.0000
Use Multiplier & Dilution Factor with ISTDs


Signal 1: DAD1 A, Sig=254,4 Ref=360,100

Peak RetTime Type  Width     Area      Height     Area
  #   [min]        [min]   [mAU*s]     [mAU]        %
----|-------|----|-------|----------|----------|--------|
   1  23.805 MM    0.4979    7.33796 2.45654e-1   4.6536
   2  27.100 BB    0.4474  150.34511    3.94713  95.3464

Totals :                   157.68306    4.19278


Signal 2: DAD1 B, Sig=210,4 Ref=360,100

Peak RetTime Type  Width     Area      Height     Area
  #   [min]        [min]   [mAU*s]     [mAU]        %
----|-------|----|-------|----------|----------|--------|
   1  23.732 BB    0.4320  414.46185   11.28441   4.6524
   2  27.095 BB    0.5840 8494.01953  211.73776  95.3476

Totals :                  8908.48138  223.02218


Signal 3: DAD1 D, Sig=230,4 Ref=360,100

=====================================================================
                          *** End of Report ***
```

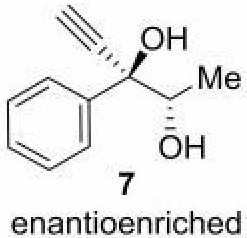

7
enantioenriched

Acq. Operator : SYSTEM Seq. Line : 6
Sample Operator : SYSTEM
Acq. Instrument : HPLC Location : P1-F-02
Injection Date : 13/4/2023 8:49:09 pm Inj : 1
Inj Volume : 2.000 µl
Different Inj Volume from Sample Entry! Actual Inj Volume : 1.000 µl
Acq. Method : C:\Users\Public\Documents\ChemStation\1\Data\SUN\SUN 2023-04-13 19-28-39 \OD3-10-20.M
Last changed : 15/8/2022 10:27:52 pm by SYSTEM
Analysis Method : C:\Users\Public\Documents\ChemStation\1\Data\SUN\SUN 2023-04-13 19-28-39 \OD3-10-20.M (Sequence Method)
Last changed : 15/4/2023 1:49:49 pm by SYSTEM
(modified after loading)
Additional Info : Peak(s) manually integrated

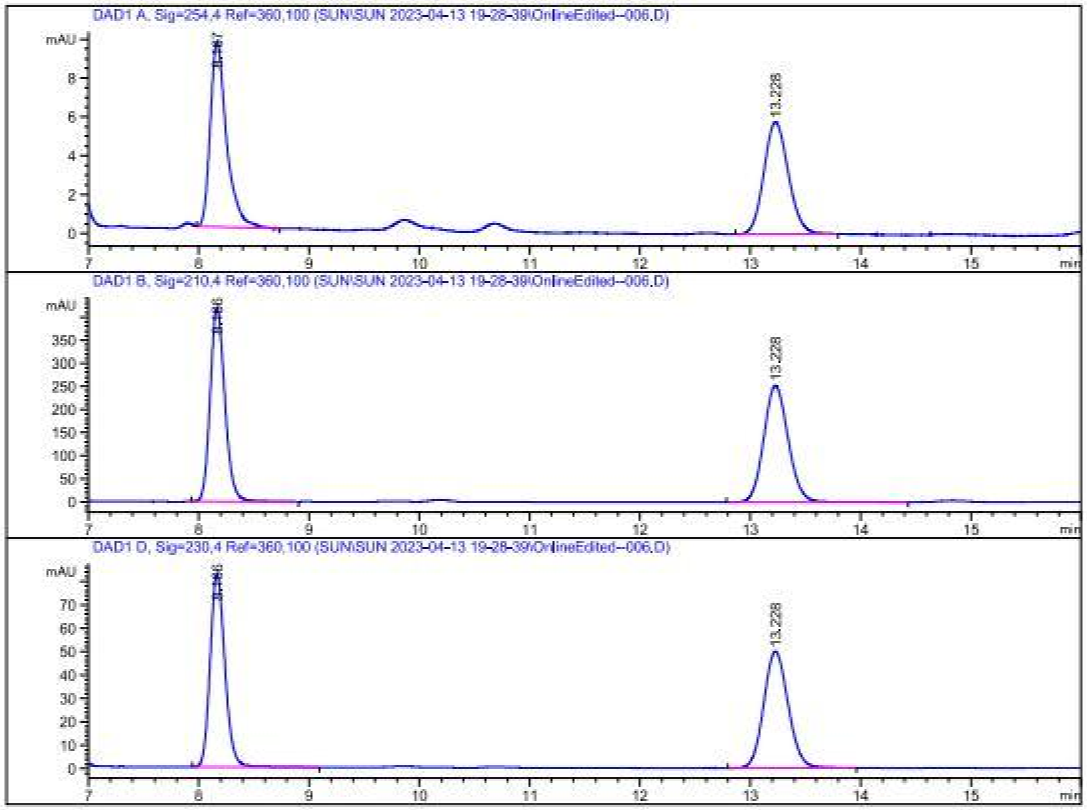

Data File C:\Users\P...\ChemStation\1\Data\SUN\SUN 2023-04-13 19-28-39\OnlineEdited--006.D
Sample Name: qcx-6-99a-2

Area Percent Report

Sorted By : Signal
Multiplier : 1.0000
Dilution : 1.0000
Use Multiplier & Dilution Factor with ISTDs

Signal 1: DAD1 A, Sig=254,4 Ref=360,100

Peak #	RetTime [min]	Type	Width [min]	Area [mAU*s]	Height [mAU]	Area %
1	8.167	BB	0.1502	98.10477	9.56174	52.2205
2	13.228	BB	0.2307	89.76163	5.77869	47.7795

Totals : 187.86640 15.34043

Signal 2: DAD1 B, Sig=210,4 Ref=360,100

Peak #	RetTime [min]	Type	Width [min]	Area [mAU*s]	Height [mAU]	Area %
1	8.166	BB	0.1428	3885.25830	420.37338	49.9640
2	13.228	BB	0.2402	3890.86377	251.96671	50.0360

Totals : 7776.12207 672.34009

Signal 3: DAD1 D, Sig=230,4 Ref=360,100

Peak #	RetTime [min]	Type	Width [min]	Area [mAU*s]	Height [mAU]	Area %
1	8.166	BB	0.1433	768.21466	82.76736	49.9961
2	13.228	BB	0.2400	768.33374	49.81039	50.0039

Totals : 1536.54840 132.57775

*** End of Report ***

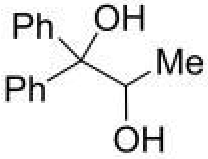

8
racemic

```
=====================================================================
Acq. Operator   : SYSTEM                          Seq. Line :  16
Sample Operator : SYSTEM
Acq. Instrument : HPLC                             Location :   P1-F-02
Injection Date  : 13/4/2023 11:11:34 pm                 Inj :   1
                                               Inj Volume : 2.000 µl
Different Inj Volume from Sample Entry!  Actual Inj Volume : 10.000 µl
Acq. Method     : C:\Users\Public\Documents\ChemStation\1\Data\SUN\SUN 2023-04-13 19-28-39
                  \OD3-10-20.M
Last changed    : 15/8/2022 10:27:52 pm by SYSTEM
Analysis Method : C:\Users\Public\Documents\ChemStation\1\Data\SUN\SUN 2023-04-13 19-28-39
                  \OD3-10-20.M (Sequence Method)
Last changed    : 15/4/2023 1:49:49 pm by SYSTEM
                  (modified after loading)
Additional Info : Peak(s) manually integrated
```

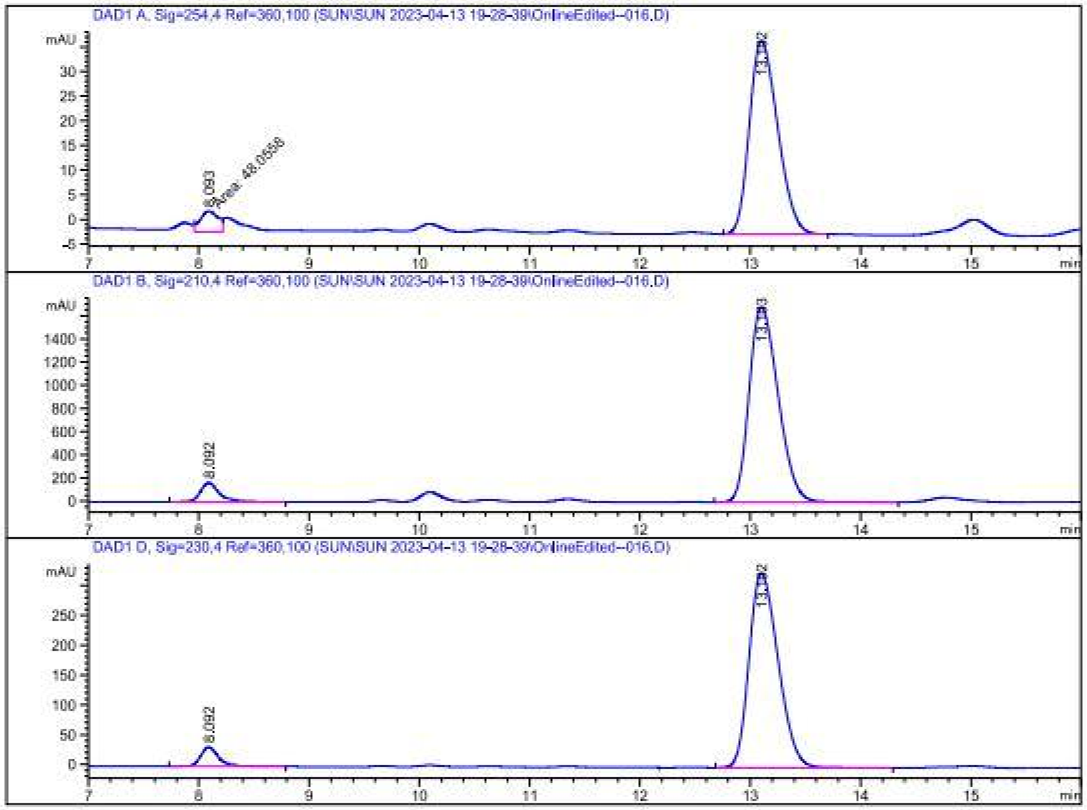

Data File C:\Users\P...\ChemStation\1\Data\SUN\SUN 2023-04-13 19-28-39\OnlineEdited--016.D
Sample Name: qcx-6-100a

===

Area Percent Report

===

```
Sorted By             :      Signal
Multiplier            :      1.0000
Dilution              :      1.0000
Use Multiplier & Dilution Factor with ISTDs
```

Signal 1: DAD1 A, Sig=254,4 Ref=360,100

Peak #	RetTime [min]	Type	Width [min]	Area [mAU*s]	Height [mAU]	Area %
1	8.093	MF	0.1904	48.05576	4.20688	6.4135
2	13.102	BB	0.2779	701.23822	39.15988	93.5865
Totals :				749.29398	43.36676	

Signal 2: DAD1 B, Sig=210,4 Ref=360,100

Peak #	RetTime [min]	Type	Width [min]	Area [mAU*s]	Height [mAU]	Area %
1	8.092	BB	0.1780	1935.17798	163.56900	5.9257
2	13.103	BB	0.2871	3.07224e4	1681.92529	94.0743
Totals :				3.26576e4	1845.49429	

Signal 3: DAD1 D, Sig=230,4 Ref=360,100

Peak #	RetTime [min]	Type	Width [min]	Area [mAU*s]	Height [mAU]	Area %
1	8.092	BB	0.1750	374.73514	32.38246	5.8960
2	13.102	BB	0.2869	5981.06348	326.18256	94.1040
Totals :				6355.79861	358.56501	

===

*** End of Report ***

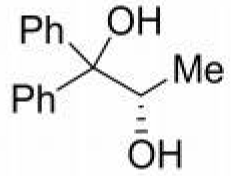

8

enantioenriched

===

Acq. Operator : SYSTEM Seq. Line : 2
Sample Operator : SYSTEM
Acq. Instrument : HPLC Location : P2-D-01
Injection Date : 14/4/2023 3:11:01 pm Inj : 1
Inj Volume : 2.000 µl
Different Inj Volume from Sample Entry! Actual Inj Volume : 8.000 µl
Acq. Method : C:\Users\Public\Documents\ChemStation\1\Data\SUN\SUN 2023-04-14 14-38-25 \ID3-10-20.M
Last changed : 19/8/2022 10:53:06 pm by SYSTEM
Analysis Method : C:\Users\Public\Documents\ChemStation\1\Data\SUN\SUN 2023-04-14 14-38-25 \ID3-10-20.M (Sequence Method)
Last changed : 17/4/2023 1:17:27 pm by SYSTEM
(modified after loading)
Additional Info : Peak(s) manually integrated

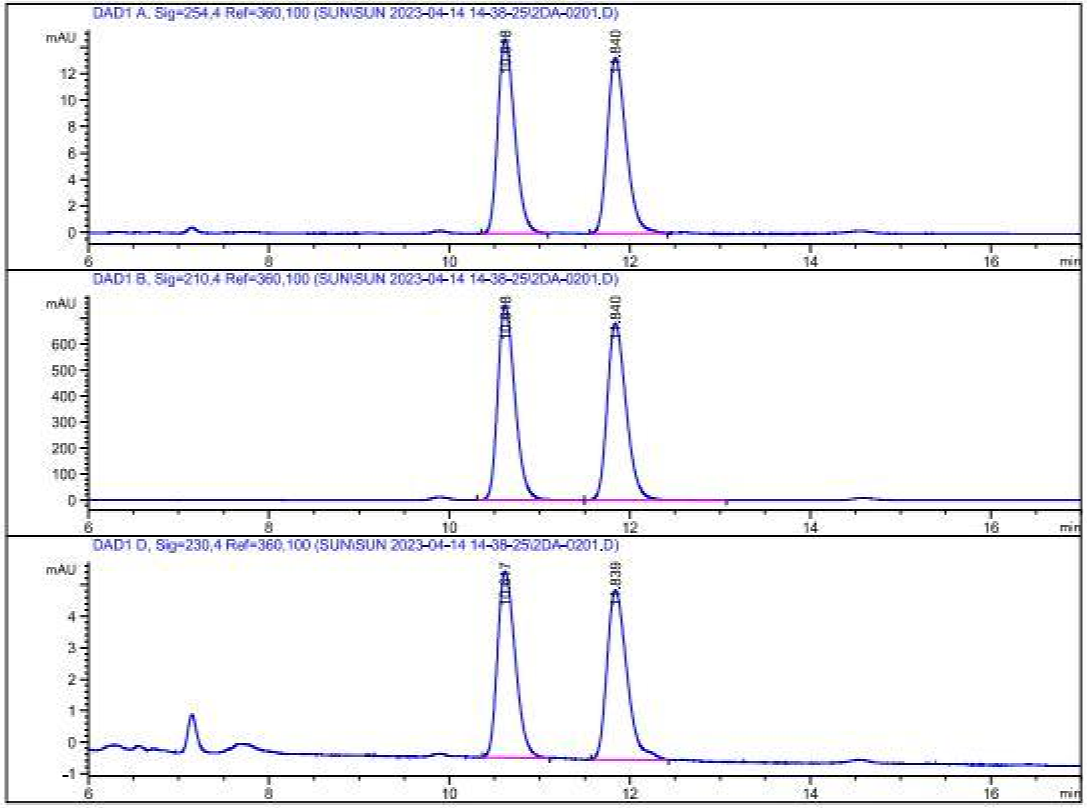

Data File C:\Users\P...Documents\ChemStation\1\Data\SUN\SUN 2023-04-14 14-38-25\2DA-0201.D
Sample Name: qcx-6-100b

===
Area Percent Report
===

Sorted By : Signal
Multiplier : 1.0000
Dilution : 1.0000
Use Multiplier & Dilution Factor with ISTDs

Signal 1: DAD1 A, Sig=254,4 Ref=360,100

Peak #	RetTime [min]	Type	Width [min]	Area [mAU*s]	Height [mAU]	Area %
1	10.618	BB	0.1992	191.13289	14.69606	49.9074
2	11.840	BB	0.2185	191.84195	13.28018	50.0926

Totals : 382.97484 27.97623

Signal 2: DAD1 B, Sig=210,4 Ref=360,100

Peak #	RetTime [min]	Type	Width [min]	Area [mAU*s]	Height [mAU]	Area %
1	10.618	BB	0.2039	9834.62207	750.18097	49.9567
2	11.840	BB	0.2244	9851.68359	680.44843	50.0433

Totals : 1.96863e4 1430.62939

Signal 3: DAD1 D, Sig=230,4 Ref=360,100

Peak #	RetTime [min]	Type	Width [min]	Area [mAU*s]	Height [mAU]	Area %
1	10.617	BB	0.1909	80.61909	5.91956	49.4327
2	11.839	BB	0.2182	82.46934	5.38381	50.5673

Totals : 163.08842 11.30337

===
*** End of Report ***

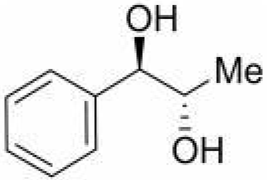

9
racemic

Acq. Operator : SYSTEM Seq. Line : 2
Sample Operator : SYSTEM
Acq. Instrument : HPLC Location : P1-F-04
Injection Date : 16/4/2023 7:33:22 pm Inj : 1
Inj Volume : 2.000 µl
Different Inj Volume from Sample Entry! Actual Inj Volume : 3.000 µl
Acq. Method : C:\Users\Public\Documents\ChemStation\1\Data\SUN\SUN 2023-04-16 19-01-04\ID3-10-30.M
Last changed : 16/8/2022 10:05:02 am by SYSTEM
Analysis Method : C:\Users\Public\Documents\ChemStation\1\Data\SUN\SUN 2023-04-16 19-01-04\ID3-10-30.M (Sequence Method)
Last changed : 17/4/2023 1:12:22 pm by SYSTEM
(modified after loading)
Additional Info : Peak(s) manually integrated

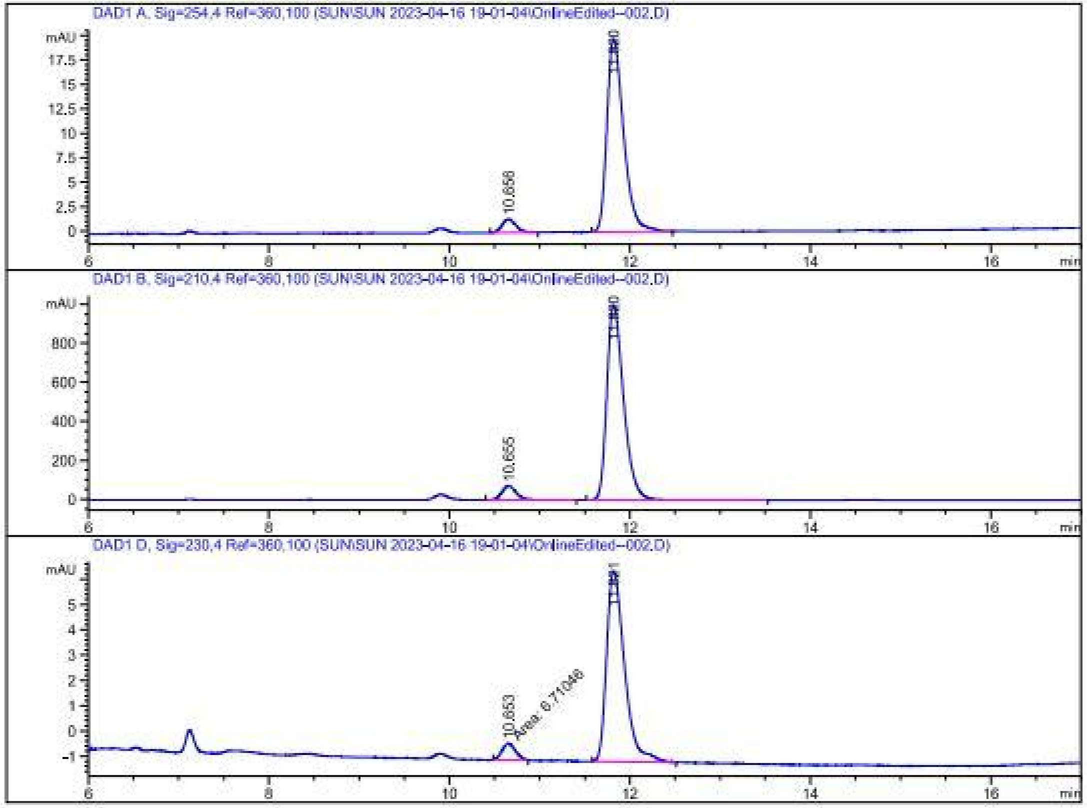

Data File C:\Users\P...\ChemStation\1\Data\SUN\SUN 2023-04-16 19-01-04\OnlineEdited--002.D
Sample Name: qcx-6-104a-2

Area Percent Report

Sorted By : Signal
Multiplier : 1.0000
Dilution : 1.0000
Use Multiplier & Dilution Factor with ISTDs

Signal 1: DAD1 A, Sig=254,4 Ref=360,100

Peak #	RetTime [min]	Type	Width [min]	Area [mAU*s]	Height [mAU]	Area %
1	10.656	BB	0.1547	15.16069	1.39619	5.6553
2	11.820	BB	0.1979	252.91689	19.68853	94.3447

Totals : 268.07758 21.08472

Signal 2: DAD1 B, Sig=210,4 Ref=360,100

Peak #	RetTime [min]	Type	Width [min]	Area [mAU*s]	Height [mAU]	Area %
1	10.655	BB	0.1695	788.45172	71.60142	5.7928
2	11.820	BB	0.1967	1.28224e4	999.27399	94.2072

Totals : 1.36109e4 1070.87540

Signal 3: DAD1 D, Sig=230,4 Ref=360,100

Peak #	RetTime [min]	Type	Width [min]	Area [mAU*s]	Height [mAU]	Area %
1	10.653	MM	0.1698	6.71046	6.58755e-1	6.0708
2	11.821	BB	0.2023	103.82688	7.53209	93.9292

Totals : 110.53734 8.19085

*** End of Report ***

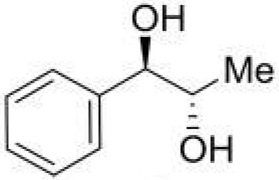

9
enantioenriched
